# Dysregulated methylation-ubiquitination crosstalk accelerates intervertebral disc degeneration via MED12 destabilization and cGAS/STING activation

**DOI:** 10.1172/JCI197274

**Published:** 2026-06-30

**Authors:** Huaizhen Liang, Dingchao Zhu, Du, Xinyu Li, Rui Shi, Jie Lei, Bide Tong, Hanpeng Xu, Di Wu, Xingyu Zhou, Yifan Du, Zixuan Ou, Junyu Wei, Shuchang Peng, Wencan Ke, Zhiwei Liao, Bingjin Wang, Kun Wang, Xiaobo Feng, Yu Song, Cao Yang

**Affiliations:** 1 Department of Orthopaedics, Union Hospital, Tongji Medical College, Huazhong University of Science and Technology, Wuhan, China.; 2 Hubei JiangXia Laboratory, Wuhan, China.

**Keywords:** Aging, Bone biology, Cellular senescence, Orthopedics, Ubiquitin-proteosome system

## Abstract

Intervertebral disc degeneration (IVDD) is a leading cause of low back pain, yet there remains no effective therapeutic approach to reverse its progression, imposing a substantial socioeconomic burden. While multiple factors contribute to IVDD pathogenesis, cellular senescence has emerged as a critical risk factor associated with both the incidence and progression of IVDD. Aging and other damage factors drive nucleus pulposus cells (NPCs) toward a senescent phenotype characterized by increased secretion of proinflammatory factors, resulting in NPC dysfunction and tissue degeneration, which are hallmarks of IVDD. In this study, we demonstrated that PRMT2 deficiency disrupted arginine methylation-ubiquitination crosstalk, driving NPC inflammatory senescence and accelerating IVDD progression. Mechanistically, PRMT2 loss reduced FBXO7 methylation at Arg504, promoting the FBXO7–MED12 interaction to facilitate MED12 ubiquitination and subsequent proteasomal degradation. MED12 deficiency induced pathological R-loop accumulation, which activated the cytosolic DNA–sensing cGAS/STING axis, triggering inflammatory response cascades. Notably, engineered extracellular vesicles delivering MED12-overexpressing plasmids significantly inhibited NPC senescence and attenuated IVDD progression. Together, our findings establish that dysregulated methylation-ubiquitination crosstalk critically drives IVDD progression and reveal MED12 as a promising therapeutic target for ameliorating the impact of IVDD.

## Abstract 2

PRMT2 deficiency‒mediated methylation-ubiquitination crosstalk of MED12 promotes R-loop/cGAS/STING axis-dependent IVDD progression.

## Graphical abstract

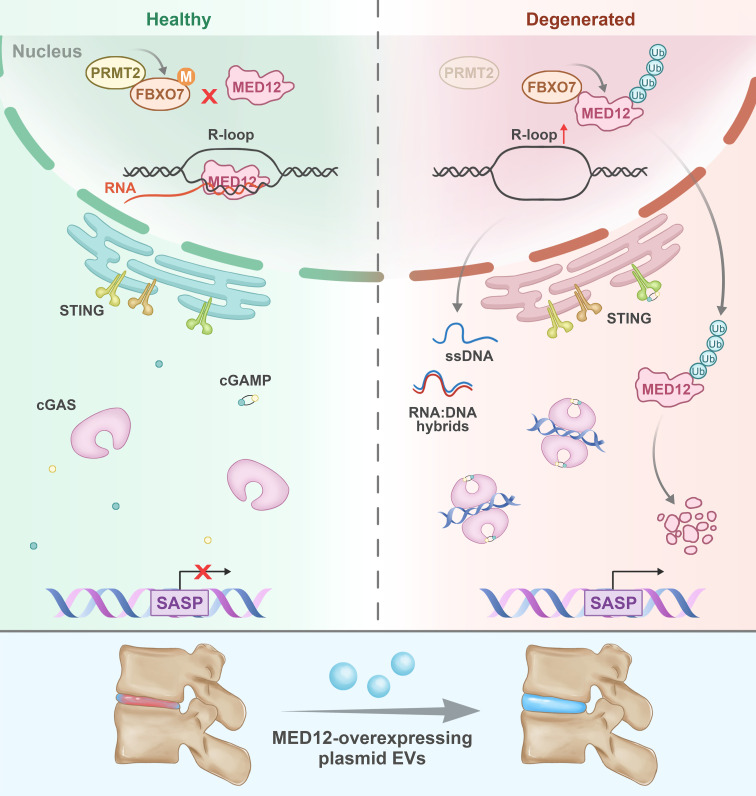

## Introduction

Intervertebral disc degeneration (IVDD) is a geriatric disease that is becoming increasingly prevalent in modern society (1). It manifests itself primarily as low back pain, which severely affects a patient’s daily life and ability to work (2, 3). The incidence of IVDD continues to rise as the global population ages and sedentary lifestyles become more common. Aging and various injury factors contribute to the conversion of nucleus pulposus cells (NPCs) to a proinflammatory and hypersecretory senescent phenotype, leading to dysfunction of NPCs and tissue inflammation and degeneration, which can cause the development of IVDD (4–7). An in-depth analysis of the regulatory mechanisms of nucleus pulposus (NP) senescence is important for exploring clinical therapeutic strategies for IVDD.

Posttranslational modifications (PTMs) play pivotal roles in the intricate cellular signaling pathways (8, 9). PTMs do not act in isolation, and the interplay between different types of PTMs is emerging as an important dimension of cellular regulation that fine-tunes cellular responses to environmental cues (10, 11). Among the various PTMs, arginine methylation has received much attention because of its prevalence and importance in cellular processes (9, 12). Arginine methylation involves the addition of a methyl group to the nitrogen atom of an arginine residue, a modification that affects protein–protein interactions, protein stability, and enzyme activity (13–15). However, the interplay between arginine methylation and other PTMs remains poorly understood. Interestingly, our mass spectral analysis revealed significant changes in the arginine methylation levels of FBXO7 during IVDD progression. FBXO7 is a member of the F-box protein family and constitutes the substrate-recruiting subunit of the Skp1-Cullin1-F-box protein (SCF) ubiquitin E3 ligase complex (16, 17). The arginine methylation of the ubiquitin substrate aptamer FBXO7 observed in our study prompted us to explore whether this PTM interplay is involved in IVDD progression and to identify the underlying molecular mechanisms involved.

Surprisingly, our proteomic analysis revealed MED12 as the most prominent target of methylation-ubiquitination crosstalk, which involves ubiquitin-dependent protein degradation during IVDD development. MED12, a member of the Mediator complex, is a multifunctional protein that acts as a platform to interact with histone-modifying enzymes, coactivators, transcription factors, and the Mediator complex to regulate epigenetic landscapes and gene expression (18–21). Given that MED12 is a universally required coordinator of nuclear homeostasis regulation, its role in cellular senescence and the development of age-related IVDD warrants further exploration.

In this study, we report that during IVDD progression, the PRMT2 deficiency–mediated loss of arginine methylation of FBXO7 leads to aberrant R-loop formation and promotes inflammatory senescence in NPCs. Mechanistically, loss of PRMT2 leads to reduced arginine methylation of FBXO7, which promotes ubiquitin-dependent protein degradation of MED12 by increasing the interaction of FBXO7 with MED12. Thus, MED12 deficiency causes R-loop accumulation, triggering an inflammatory cascade response through the activation of DNA sensing–related signaling pathways. Notably, extracellular vesicles (EVs) loaded with MED12-overexpressing plasmids significantly alleviated NPC senescence and the progression of IVDD.

## Results

### FBXO7 methylation deficiency promotes NPC senescence and IVDD progression.

**Figure 1:**
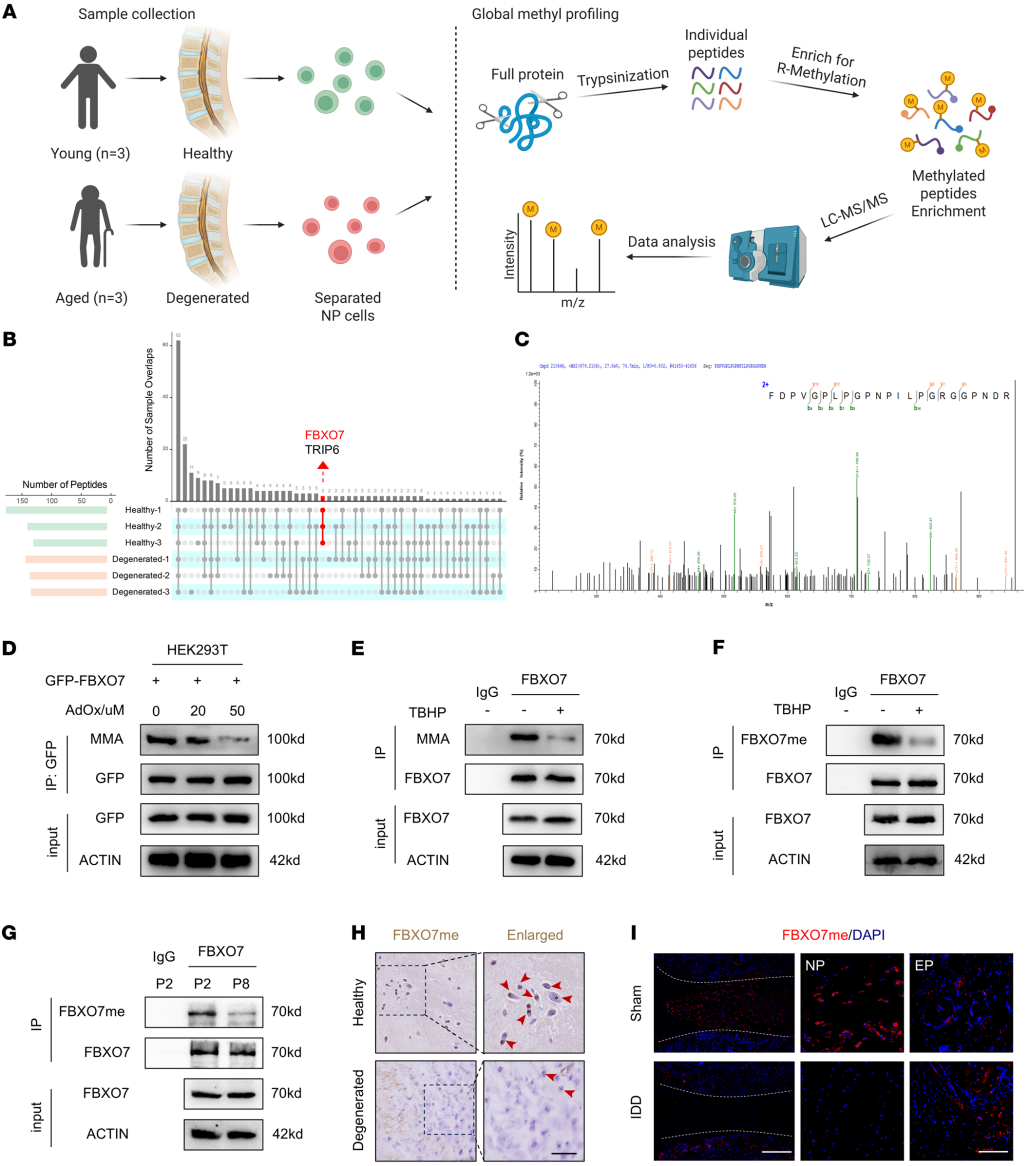
FBXO7 methylation is downregulated in IVDD. (**A**) Schematic representation of the experimental design to quantify the arginine methylproteome in IVDD. (**B**) Upset plot indicating the presence of arginine methylated peptides identified in each sample. (**C**) Liquid chromatography–tandem mass spectrometry of the tryptic peptide FDPVGPLPGPNPILPGRGGPNDR identified a monomethylated residue at R504. (**D**) Methyltransferase inhibitors decrease FBXO7 methylation. GFP-tagged FBXO7 was expressed in HEK293cells, followed by treatment with increasing concentrations of the methyltransferase inhibitor AdOx. (**E**) Co-IP analysis of methylated FBXO7 in NPCs with the indicated treatment. (**F**) Co-IP analysis of FBXO7-R504me in NPCs with the indicated treatment. (**G**) Co-IP analysis of FBXO7-R504me in P2 and P8 NPCs. (**H**) IHC of the FBXO7-R504me in human NP tissue. Scale bar: 100 μm. (**I**) IF staining of FBXO7-R504me in coccygeal vertebrae from rats with the indicated treatment. Scale bars: 1 mm (left), 250 μm (right). At least 3 independent experiments were performed.

To comprehensively analyze the dynamics of methylated proteins during IVDD, we employed a label-free quantitative proteomics approach using a pan-methylated antibody to enrich methylated peptides from total protein extracts of healthy and degenerated NP tissues (Figure 1A). We quantified 158 monomethylarginine (MMA) sites in more than half of the biological replicates, with 125 methylated sites quantified in degenerated samples and 131 methylated sites quantified in healthy samples. Strikingly, we observed MMA at position 504 of FBXO7 in all the biological replicates of the healthy samples, yet it was consistently absent in the degenerated samples (Figure 1, B and C, and Supplemental Figure 1A; supplemental material available online with this article; https://doi.org/10.1172/JCI197274DS1). Next, we verified the presence of arginine methylation in FBXO7. We exogenously expressed the FBXO7 protein fused with a GFP tag in HEK293T cells, which were immunoprecipitated and then subjected to Western blotting using a pan–anti-MMA antibody, which revealed MMA of FBXO7. Moreover, its methylation level decreased in a dose-dependent manner following treatment with the protein arginine methyltransferase (PRMT) inhibitor adenosine dialdehyde (AdOx) (Figure 1D) (22). Furthermore, we used tert-butyl hydroperoxide (TBHP) to establish an in vitro senescence model of NPCs, and our results showed that treatment with 50 μM TBHP for 5 days induced a stable senescent phenotype (Supplemental Figure 2, A–D). We observed that arginine methylation of endogenous FBXO7 was significantly decreased in TBHP-induced degenerated NPCs (Figure 1E). Next, we introduced arginine-to-lysine (RK) and arginine-to-phenylalanine (RF) substitutions at position 504 of FBXO7. RK substitution achieved methylation deficiency in FBXO7, whereas the RF substitution mimics a constitutive methylation in FBXO7 (23, 24). As expected, the RK FBXO7 mutant presented a marked reduction in methylation levels relative to those of WT FBXO7. Furthermore, TBHP treatment elicited significant inhibition of methylation in WT FBXO7, whereas the RK mutant showed no further reduction in methylation compared with the basal state (Supplemental Figure 1B). To specifically detect arginine methylation at position 504 of FBXO7, we generated a site-specific methylation antibody. Dot blot analysis confirmed its specificity (Supplemental Figure 1, C and D). In both TBHP-induced and replication stress–induced senescence models of NPCs, we consistently observed a marked downregulation of FBXO7-R504me levels (Figure 1, F and G). Furthermore, we validated the decreased levels of FBXO7-R504me in another independent cohort of human NP tissues with IVDD (Figure 1H and Supplemental Figure 1E). Immunofluorescence (IF) staining revealed that FBXO7-R504me was downregulated in the NP tissue concomitant with disc degeneration, whereas its levels remained unchanged in the endplate (Figure 1I). Collectively, these data demonstrated that FBXO7 undergoes methylation at R504.

**Figure 2:**
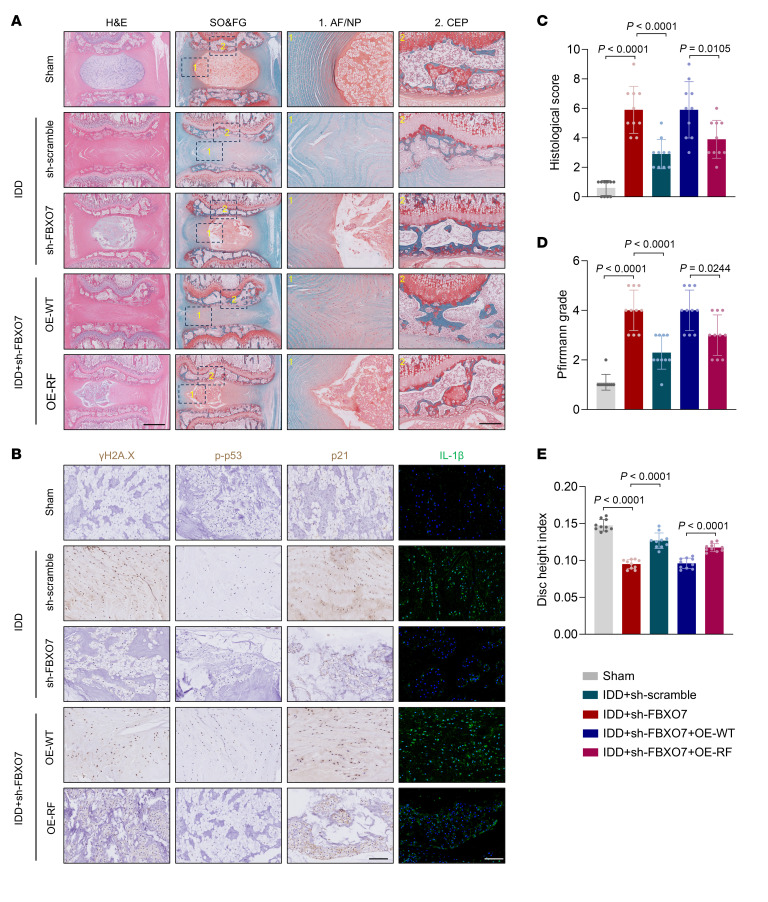
FBXO7 methylation deficiency promotes NPC senescence and IVDD progression. (**A**) H&E staining and SO&FG staining of coccygeal vertebrae from rats with the indicated treatment. Scale bars: 1 mm (left), 250 μm (right). (**B**) IHC staining of p-p53, γH2AX, and p21 and IF staining of IL-1β in rat coccygeal IVDs. Scale bars: 100  μm. (**C**–**E**) Histological score (*n* = 10) (**C**), Pfirrmann degenerative grades (*n* = 10) (**D**), and disc height index (*n* = 10) (**E**) of rat coccygeal IVDs. One-way ANOVA (**C**–**E**); data are presented as mean ± SD. At least 3 independent experiments were performed.

The observed absence of R504 methylation in FBXO7 cells from degenerated NPCs prompted us to investigate its potential causal role in IVDD pathogenesis. We transduced WT FBXO7 or RF substitution FBXO7 mutant plasmids into FBXO7-deficient NPCs through recombinant expression. TBHP treatment induced a marked cellular senescence phenotype in NPCs transduced with WT FBXO7, as evidenced by significant upregulation of senescence-associated markers, accumulation of phosphorylated γH2AX foci, and markedly reduced 5-ethynyl-2′-deoxyuridine (EdU) incorporation rates (25). In contrast, the recombinant RF mutant conferred resistance to the TBHP-triggered senescence phenotype (Supplemental Figure 2, E–H). To systematically investigate the in vivo functional consequences of FBXO7 R504 methylation in IVDD, we established a validated caudal IVDD rat model through needle puncture. In this model, endogenous FBXO7 was first knocked down via intradiscal delivery of adeno-associated virus (AAVs) expressing FBXO7 shRNA, followed by reconstitution with either WT FBXO7 or the RF substitution mutant (Supplemental Figure 2, I–L). MRI evaluation demonstrated that T2-weighted MRI signal intensity significantly decreased with needle puncture in intervertebral discs (IVDs) transduced with WT FBXO7 plasmids, whereas the magnitude of signal loss was significantly attenuated in IVDs transduced with RF mutant plasmids (Figure 2D and Supplemental Figure 2M) (26). Integrated x-ray and �CT analyses revealed that reconstitution with the FBXO7 RF mutant significantly attenuated disc height loss in needle puncture–induced degenerative discs compared with IVDs reconstituted with WT FBXO7 (Figure 2E and Supplemental Figure 2M). Moreover, histological staining indicated that the reconstitution of RF mutants in IVDs attenuated the needle puncture–induced degenerated phenotype, as indicated by improved extracellular matrix organization, distinct anatomical demarcation between the NP and annulus fibrosus regions, and relative preservation of the NP volume compared with those of WT FBXO7–reconstituted discs (Figure 2, A and C) (27). Importantly, IHC and IF staining demonstrated that, compared with WT FBXO7–reconstituted discs, IVDs reconstituted with the RF mutant presented significantly lower proportions of phosphorylated p53^+^ (p-p53^+^), γH2AX^+^, p21^+^, and IL-1β^+^ NPCs (Figure 2B). Collectively, these findings demonstrated that pathological loss of FBXO7 R504 methylation might drive IVDD progression.

### Loss of PRMT2-mediated FBXO7 R504 methylation promoted NPC senescence and IVDD progression.

**Figure 3:**
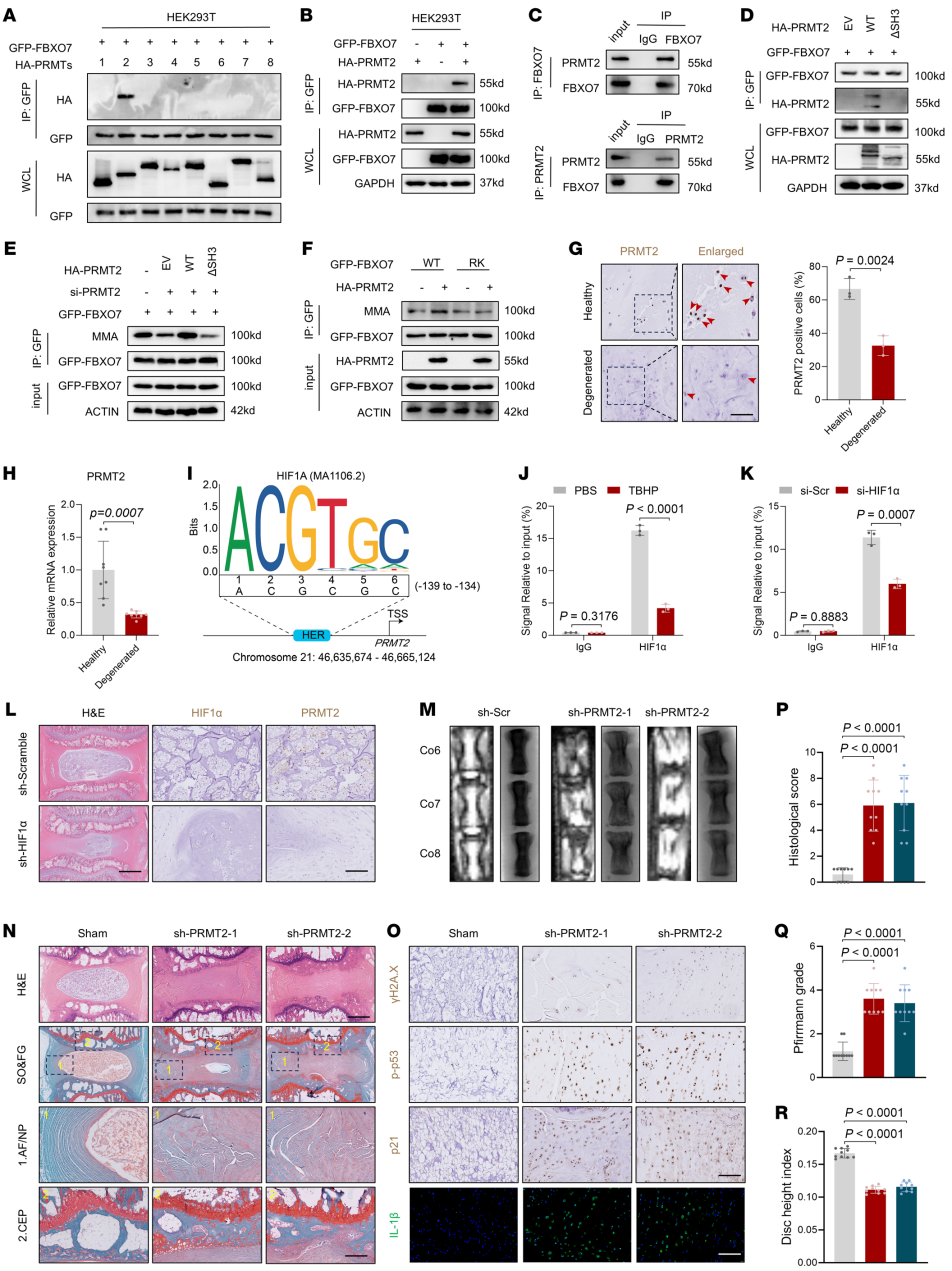
Loss of PRMT2-mediated FBXO7 R504 methylation promoted NPC senescence and IVDD progression. (**A**) Proteins were immunoprecipitated using GFP antibody and analyzed by Western blotting in HEK293T cells transfected with indicated constructs. WCL, whole-cell lysate. (**B**) Exogenous co-IP analysis of the interaction of FBXO7 with PRMT2 in HEK293T cells. (**C**) Endogenous co-IP analysis of the interaction of FBXO7 with PRMT2 in NPCs. (**D**) Proteins were immunoprecipitated using GFP antibody and analyzed by Western blotting in HEK293T cells transfected with indicated constructs. (**E**) Co-IP analysis of methylated FBXO7 in PRMT2-silenced HEK293T cells. (**F**) Co-IP analysis of methylated FBXO7 in HEK293T cells transfected with indicated constructs. (**G**) IHC of the PRMT2 in human NP tissue. Arrowheads indicate PRMT2^+^ cells. Scale bar: 100 μm. (**H**) mRNA expression analysis of PRMT2 in human NP tissue by RT-qPCR. (**I**) Schematic showing that the base sequence represents the consensus HIF1α-binding motif. (**J**) ChIP-PCR analysis of the enrichment of HIF1α in the PRMT2 promoter in NPCs with the indicated treatment. (**K**) ChIP-PCR analysis of the enrichment of HIF1α in the PRMT2 promoter in NPCs with HIF1α silencing. (**L**) H&E staining and IHC staining of coccygeal vertebrae from rats with the indicated treatment. Scale bar: 1 mm (left), 100 μm (right). (**M**) Representative x-ray and MRI images of coccygeal vertebrae from rats with the indicated treatment. (**N**) H&E staining and SO&FG staining of coccygeal vertebrae from rats with the indicated treatment. Scale bar: 1 mm (top), 250 μm (bottom). (**O**) IHC staining of p-p53, γH2AX, and p21 and IF staining of IL-1β in rat coccygeal IVDs. Scale bars: 100 μm. (**P**–**R**) Histological score (**P**), Pfirrmann degenerative grades (**Q**), and disc height index (**R**) of rat coccygeal IVDs. Unpaired 2-tailed Student’s *t* test (**G**, **H**, **J**, and **K**); 1-way ANOVA (**P–R**); data are presented as mean ± SD. At least 3 independent experiments were performed.

To identify methyltransferases capable of methylating FBXO7, we conducted a comprehensive PRMT family screen by coexpressing GFP-FBXO7 with HA-tagged PRMTs (PRMT1-8) in HEK293T cells. Co-IP analysis showed that PRMT2 interacted only with FBXO7 but not with other PRMTs (Figure 3A). The PRMT2–FBXO7 interaction was validated through reciprocal co-IP in HEK293T cells (Figure 3B and Supplemental Figure 3A). Consistently, the binding of endogenous PRMT2 and FBXO7 was detected in NPCs (Figure 3C). FBXO7 contains several domains: a ubiquitin-like domain, an FBXO7-PI31 dimerization domain, an F-box domain, and proline-rich motifs (17). We demonstrated that deletion of the C-terminal proline-rich region abrogated the interaction between FBXO7 and PRMT2 (Supplemental Figure 3, B and C) (28). The N-terminal SH3 domain of PRMT2 is known to mediate specific recognition of proline-rich motifs in binding partners (29, 30). Our findings revealed that the SH3 domain is necessary for the interaction of PRMT2 with FBXO7 (Figure 3D and Supplemental Figure 3B). Together, these findings demonstrate that PRMT2 directly interacts with FBXO7. Furthermore, we explored whether FBXO7 is methylated by PRMT2. Reconstitution of full-length PRMT2 or SH3 domain–deleted PRMT2 (ΔSH3-PRMT2) in endogenous PRMT2-depleted HEK293T cells revealed that PRMT2 depletion or expression of ΔSH3-PRMT2 significantly reduced FBXO7 methylation in contrast with that in control cells or cells reconstituted with full-length PRMT2 (Figure 3E and Supplemental Figure 3D). Moreover, overexpression of PRMT2 significantly increased the methylation level of WT-FBXO7 but not the RK mutant (Figure 3F). These findings indicate that PRMT2 is essential and capable of catalyzing FBXO7 methylation at R504.

We next investigated how PRMT2 is regulated in the context of IVDD. IHC and RT-qPCR revealed that PRMT2 levels were downregulated in degenerated IVDs (Figure 3, G and H). To explore the mechanism underlying PRMT2 downregulation, we used the JASPAR database to predict transcription factors that may bind to the PRMT2 promoter. HIF1α was predicted to bind at a site located −139 to −134 upstream of the PRMT2 transcription start site (Figure 3I). HIF1α, a master transcriptional regulator of cellular adaptation to hypoxia, plays a critical protective role in IVDD by maintaining mitochondrial integrity, promoting glycolysis, and reducing oxidative stress and apoptosis, and its expression is progressively downregulated in degenerated NP tissues, correlating with disease severity. ChIP-PCR analysis demonstrated that both TBHP treatment and HIF1α knockdown reduced HIF1α enrichment at the PRMT2 promoter (Figure 3, J and K). Consistently, knockdown of HIF1α downregulated PRMT2 expression under both normoxic and hypoxic conditions (Supplemental Figure 3, E and F). To validate the functional role of HIF1α in regulating PRMT2 expression and disc degeneration in vivo, we injected AAVs expressing HIF1α shRNA into rat caudal IVDs. H&E and IHC staining revealed that the sh-HIF1α group exhibited more severe disc degeneration and concomitantly lower PRMT2 levels compared with the control group (Figure 3L). Together, these findings demonstrate that HIF1α transcriptionally regulates PRMT2 expression, and the loss of HIF1α during IVDD contributes to PRMT2 downregulation and accelerated disc degeneration.

Next, we explored the functional consequences of PRMT2-mediated FBXO7 R504 methylation on NPC senescence. PRMT2 knockdown triggered a senescence-associated phenotype characterized by elevated expression of classical senescence markers, persistent accumulation of DNA damage foci, and profound inhibition of proliferative capacity. Importantly, reconstitution of WT PRMT2, but not its SH3 domain–deficient variant, effectively attenuated these senescence-associated phenotype changes (Supplemental Figure 3, G–I). Furthermore, intradiscal delivery of PRMT2-targeting shRNA via an AAV into the rat caudal IVDs induced distinct degenerative phenotypes. Radiographic assessment demonstrated significant disc height reduction, paralleled by diminished MRI T2-weighted signal intensity indicative of NP dehydration (Figure 3, M, Q, and R). Histopathological evaluation revealed structural hallmarks of degeneration, including a contracted NP area with extracellular matrix disorganization and disrupted annulus fibrosus lamellar architecture (Figure 3, N and P). Strikingly, PRMT2 knockdown in vivo significantly increased the expression of p-p53, γH2AX, p21, and IL-1β (Figure 3O). Conversely, compared with the AAV-control group, intradiscal delivery of AAV-PRMT2 for PRMT2 overexpression markedly attenuated puncture-induced disc degeneration, as evidenced by preserved disc height, T2 signal intensity, NP matrix integrity, and organized annulus fibrosus architecture, along with reduced expression of p-p53, γH2AX, p21, and IL-1β (Supplemental Figure 4, A–F). Taken together, these results establish that deficiency in the PRMT2-mediated methylation of FBXO7 at R504 promotes both NPC senescence and IVDD progression.

### FBXO7 R504 methylation prevents FBXO7-mediated MED12 recruitment and destabilization.

**Figure 4:**
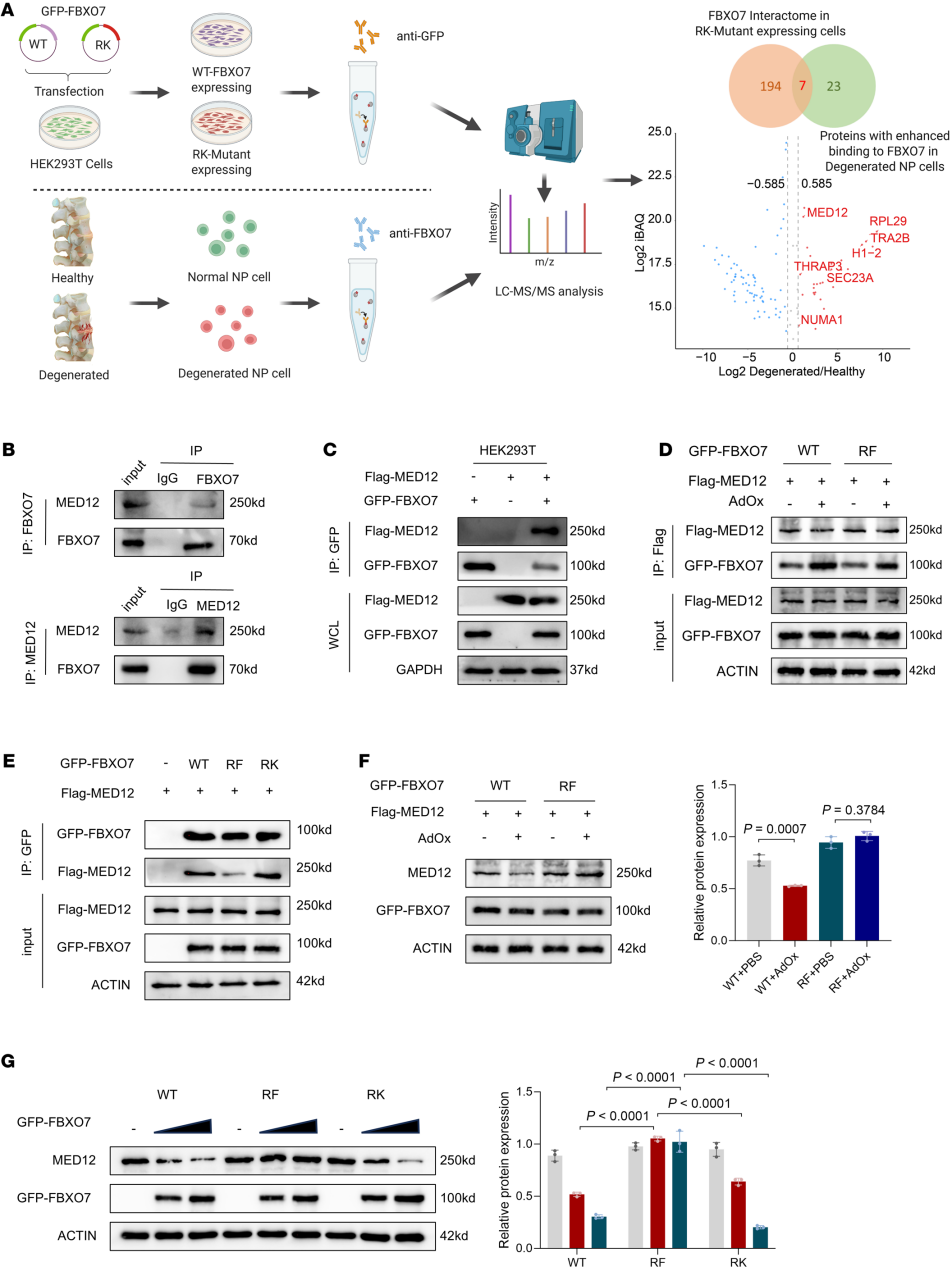
FBXO7 R504 methylation prevents FBXO7-mediated MED12 recruitment and destabilization. (**A**) Schematic workflow showing IP-MS performed to identify FBXO7-interacting proteins in NPCs and HEK293T cells. (**B**) Endogenous co-IP analysis of the interaction of FBXO7 with MED12 in NPCs. (**C**) Exogenous co-IP analysis of the interaction of FBXO7 with MED12 in HEK293T cells cotransfected with GFP-FBXO7 and Flag-MED12. (**D**) Co-IP analysis of the interaction of FBXO7 with MED12 in HEK293T cells cotransfected with Flag-MED12 and GFP-FBXO7 (WT and RF mutants) with the indicated treatment. (**E**) Co-IP analysis of the interaction of FBXO7 with MED12 in HEK293T cells cotransfected with Flag-MED12 and GFP-FBXO7 (WT, RK mutants, and RF mutants). (**F**) Protein level analysis of MED12 in HEK293T cells cotransfected with Flag-MED12 and GFP-FBXO7 (WT and RF mutants) with the indicated treatment by Western blot. (**G**) Protein level analysis of MED12 in NPCs overexpressing FBXO7 by Western blot. One-way ANOVA (**F** and **G**); data are presented as mean ± SD. At least 3 independent experiments were performed.

Building upon the established role of FBXO7 R504 methylation in mitigating IVDD, we next aimed to elucidate the molecular cascade through which this modification regulates disease pathogenesis. FBXO7 functions as the substrate-recruiting subunit of the SCF E3 ubiquitin ligase complex, with its substrate recognition dynamics potentially modulated by PTMs (31, 32). Exogenous co-IP and mass spectrometry were performed to identify the FBXO7-binding protein from WT FBXO7 or RK mutant transduced HEK293T cells. We identified 201 potential FBXO7-binding partners in RK mutant–expressing HEK293T cells and 97 candidates that interact with WT FBXO7 (Figure 4A). Further proteomic profiling of endogenous FBXO7 interactomes through anti-FBXO7 co-IP coupled with mass spectrometry revealed substantial remodeling of binding partners between normal and degenerated NPCs. Notably, 7 proteins within the FBXO7 RK mutant interactome exhibited enhanced interactions with FBXO7 in degenerated NPCs. Among them, MED12 was identified as the most significantly enriched protein among the FBXO7-binding proteins in degenerated NPCs (Figure 4A). Consistently, co-IP assays confirmed that MED12 exhibited the strongest interaction with FBXO7 among these 7 candidate proteins (Supplemental Figure 5A). As a critical component of the Mediator complex, MED12 plays a role in transcriptional initiation and epigenetic regulation, either in a Mediator-dependent or -independent manner (33, 34). Based on these findings, we proceeded to investigate the mode of interaction between FBXO7 and MED12. Reciprocal co-IP assays demonstrated a direct interaction between endogenous FBXO7 and MED12 in NPCs (Figure 4B). To further validate this interaction, exogenous coexpression experiments in HEK293T cells revealed specific coprecipitation of GFP-tagged FBXO7 with FLAG-tagged MED12 and vice versa (Figure 4C and Supplemental Figure 5B). To map the interaction domains between FBXO7 and MED12, we performed co-IP assays in HEK293T cells expressing truncated variants of both proteins. Domain mapping analysis revealed that the C-terminal region of MED12 and the ubiquitin-like domain coupled with the FBXO7-PI31 dimerization domain of FBXO7 are essential for their physical interaction (Supplemental Figure 5, C–E) (35). To investigate how FBXO7 R504 methylation regulates its interaction with MED12, we performed co-IP assays in HEK293T cells expressing WT FBXO7 or a constitutively methylated RF mutant. Treatment with the methylation inhibitor AdOx markedly enhanced the interaction between WT FBXO7 and MED12, whereas the RF mutant was less sensitive to AdOx (Figure 4D). Furthermore, we performed co-IP assays in HEK293T cells expressing WT FBXO7, methylation-resistant RK mutants, or constitutively methylated RF mutants. Strikingly, the RF mutant markedly reduced the FBXO7–MED12 interaction, whereas the WT and RK mutants retained MED12 binding capacity (Figure 4E). Moreover, Western blot analysis demonstrated reduced MED12 protein levels in degenerated NP tissues, showing an inverse correlation with the senescence phenotype of disc degeneration (Supplemental Figure 5, F and G). To determine whether FBXO7-mediated recruitment of MED12 facilitates its proteolytic turnover, we assessed MED12 protein levels in NPCs overexpressing WT FBXO7 or RK/RF mutants. WT and RK mutants markedly decreased MED12 levels in a dose-dependent manner, whereas the RF mutant failed to reduce MED12 levels (Figure 4G). Furthermore, pharmacological inhibition of methylation using AdOx reduced MED12 protein levels and destabilized MED12 in HEK293T cells expressing WT FBXO7 but not the RF mutant (Figure 4F and Supplemental Figure 5H). Collectively, these findings demonstrated that pathological loss of FBXO7 R504 methylation increased the FBXO7–MED12 interaction, thereby leading to increased MED12 destabilization.

### FBXO7 R504 methylation impedes FBXO7-mediated K48-linked ubiquitination of MED12.

**Figure 5:**
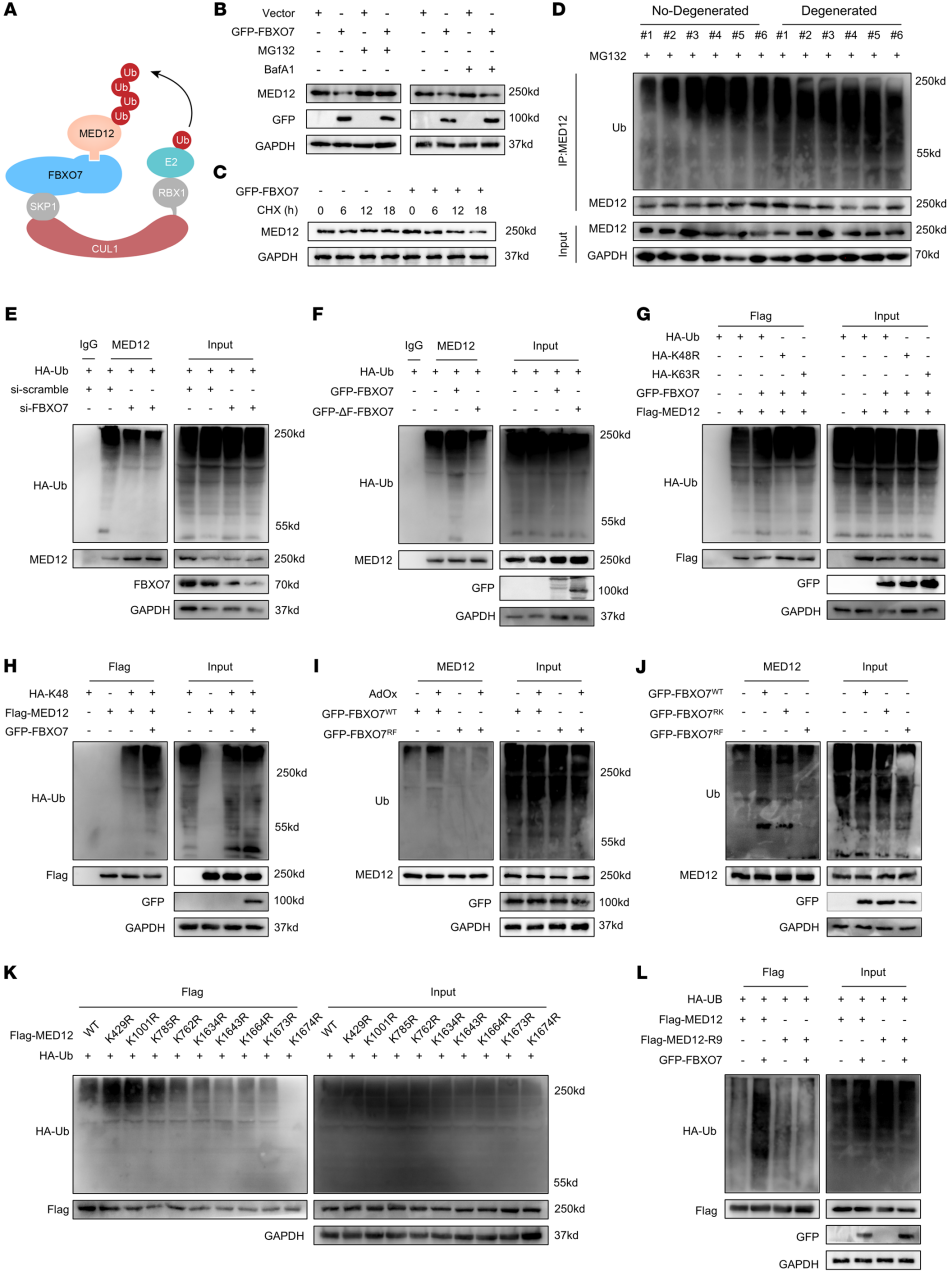
FBXO7 R504 methylation impedes FBXO7-mediated K48-linked ubiquitination of MED12. (**A**) A model of MED12 protein polyubiquitination by the FBXO7 E3 ligase complex. (**B**) Protein level analysis of MED12 in NPCs overexpressing FBXO7 with indicated treatment. (**C**) Half-life analysis of MED12 protein in NPCs with or without FBXO7 overexpression. (**D**) Co-IP analysis of MED12 ubiquitination in human samples. (**E**) Co-IP analysis of endogenous MED12 ubiquitination in NPCs treated with si-FBXO7. (**F**) Co-IP analysis of endogenous MED12 ubiquitination in NPCs overexpressing WT FBXO7 or F-box domain–deleted mutant (ΔF-FBXO7). (**G**) Co-IP analysis of exogenous MED12 ubiquitination in HEK293T cells transfected with various combinations of plasmids. (**H**) Co-IP analysis of exogenous MED12 ubiquitination in HEK293T cells transfected with various combinations of plasmids. (**I**) Co-IP analysis of endogenous MED12 ubiquitination in NPCs transfected with WT FBXO7 or FBXO7 RF mutant with the indicated treatment. (**J**) Co-IP analysis of endogenous MED12 ubiquitination in NPCs overexpressed with FBXO7 (WT, RF mutants, and RK mutants) with the indicated treatment. (**K**) Co-IP analysis of exogenous MED12 ubiquitination of WT MED12 and mutants in HEK293T cells. (**L**) Co-IP analysis of exogenous MED12 ubiquitination of WT MED12 and K1674R mutants in HEK293T cells. At least 3 independent experiments were performed.

Given the demonstrated physical interaction between FBXO7 and MED12 and their functional linkage in promoting MED12 destabilization, we propose a mechanistic model in which FBXO7 directly catalyzes MED12 polyubiquitination, thereby leading to its proteasomal degradation (Figure 5A). The decrease in MED12 protein levels induced by FBXO7 overexpression was blocked by the proteasome inhibitor MG132 but not by the lysosomal inhibitor BafA1 (Figure 5B). Consistently, the half-life of the endogenous MED12 protein was significantly reduced under FBXO7 overexpression, as demonstrated by protein stability assays using cycloheximide treatment (Figure 5C). Furthermore, co-IP experiments demonstrated significantly elevated ubiquitination levels of MED12 in degenerated NPCs from pairs of human samples (Figure 5D). We observed that MED12 ubiquitination was decreased upon FBXO7 knockdown (Figure 5E). However, overexpression of WT FBXO7, but not the catalytically inactive F-box domain–deleted ΔF-FBXO7 mutant, strongly increased MED12 ubiquitination (Figure 5F) (17). Given that K48-linked ubiquitin chains are canonical signals for proteasomal recognition and degradation, we next sought to characterize the type of FBXO7-catalyzed ubiquitin chains on MED12 (36). In HEK293T cells reconstituted with the ubiquitin-K48R mutant (defective in the formation of K48-linked ubiquitin chains), FBXO7 overexpression failed to increase MED12 ubiquitination levels, whereas this impairment was not observed with the ubiquitin-K63R mutant (Figure 5G). Furthermore, FBXO7 overexpression markedly promoted K48-linked polyubiquitin chain formation in MED12 (Figure 5H). Collectively, these findings establish that FBXO7 selectively targets MED12 for proteasomal degradation through K48-linked ubiquitination.

We subsequently investigated the impact of FBXO7 methylation on MED12 K48-linked ubiquitination. Co-IP assays demonstrated that AdOx treatment markedly elevated MED12 ubiquitination levels, whereas HEK293T cells expressing the constitutively methylation-mimetic RF mutant exhibited resistance to AdOx-induced ubiquitination (Figure 5I). Furthermore, transfection of HEK293T cells with WT FBXO7 or RF or RK mutants revealed that the methylation-mimetic RF mutant significantly reduced MED12 ubiquitination levels compared with those of both the WT and RK variants (Figure 5J). Collectively, these findings demonstrate that FBXO7 R504 methylation suppresses the K48-linked ubiquitination of MED12, thereby attenuating its proteasomal degradation. To predict potential ubiquitination sites on MED12, GPS-Uber — a hybrid learning framework integrating deep neural networks and logistic regression for E3-specific ubiquitination site prediction — was employed (37). With a confidence cutoff score of 0.6, the following lysine residues were identified as candidate ubiquitination sites: K429, K762, K785, K1001, K219, K1634, K1643, K1664, K1673, and K1674 (Supplemental Figure 6A). To validate the predicted ubiquitination sites in MED12, WT MED12 plasmids and lysine-to-arginine ubiquitination-defective mutants were transfected into HEK293T cells. Co-IP followed by Western blotting revealed that only the K1674R mutation significantly attenuated the K48-linked ubiquitination of MED12, demonstrating that K1674 is a critical ubiquitination site in MED12 (Figure 5K). Consistently, compared with WT MED12, FBXO7 overexpression did not increase the ubiquitination level of the K1674 mutant (Figure 5L). Taken together, these findings demonstrate that R504 methylation of FBXO7 suppresses MED12 K1674 ubiquitination and proteasomal degradation.

### MED12 deficiency promoted cGAS/STING axis-dependent NPC senescence during IVDD progression.

**Figure 6:**
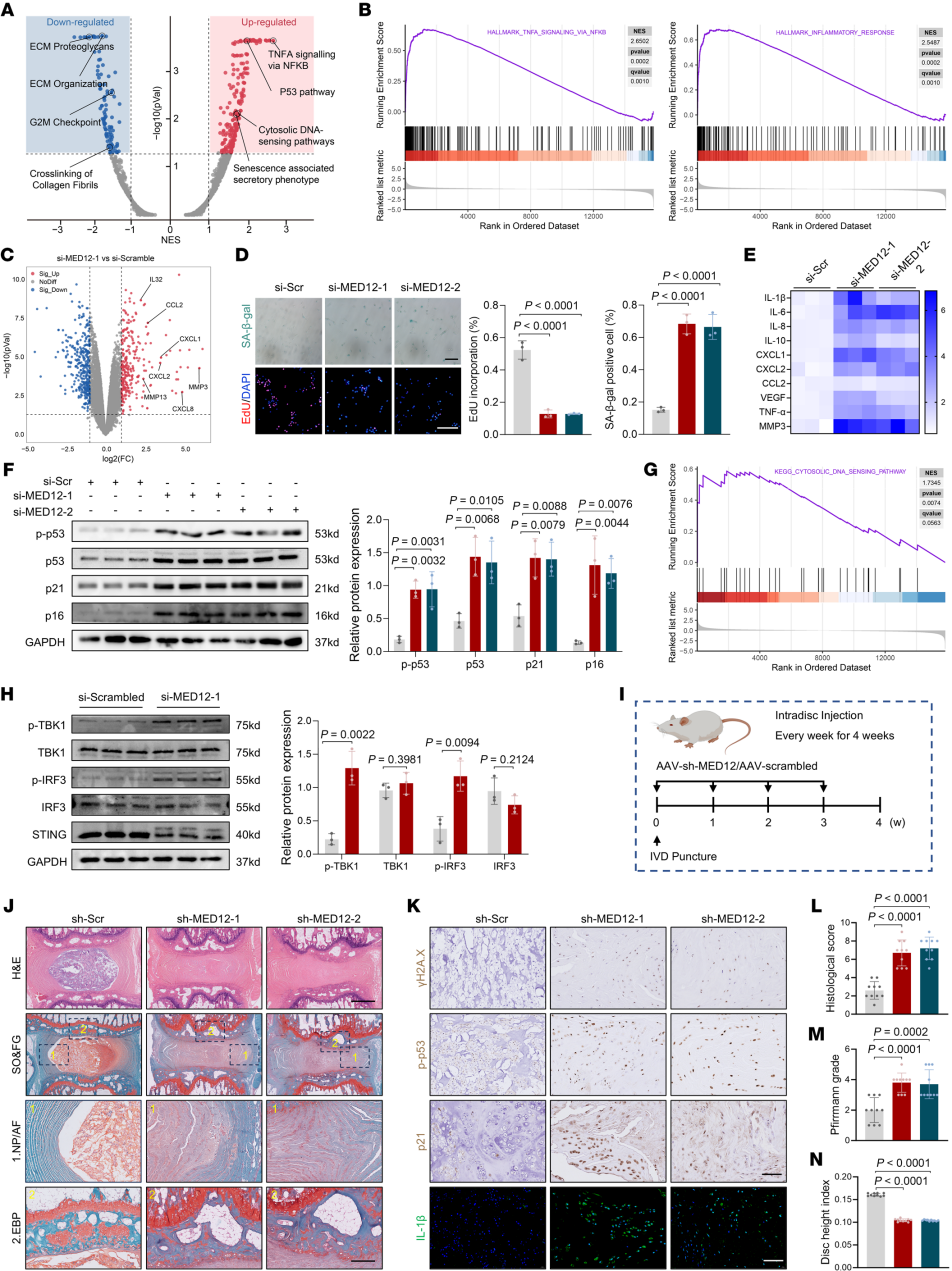
MED12 deficiency promoted cGAS/STING axis–dependent senescence of NPCs during IVDD progression. (**A**) Volcano plot showing enriched downregulated and upregulated pathways of transcriptional profiles in NPCs transfected with si-MED12-1. NES, normalized enrichment score. (**B**) GSEA showing “inflammatory response” and “TNFA signaling via NF-κB” enriched in NPCs transfected with si-MED12-1. (**C**) Volcano plot analysis of the RNA-seq dataset illustrating the top upregulated and downregulated genes in MED12-silenced NPCs compared with control NPCs. (**D**) SA-β-gal activity staining and EdU incorporation assay of NPCs transfected with si-MED12. Scale bars: 100 μm. (**E**) Heatmap of differential SASP in NPCs transfected with si-MED12. (**F**). Protein level analysis of p-p53, p53, p21, and p16 in NPCs transfected with si-MED12 by Western blot. (**G**) GSEA showing “cytosolic DNA-sensing pathway” genes enriched in NPCs transfected with si-MED12-1. (**H**) Protein-level analysis of p-TBK1, TBK1, p-IRF3, IRF3, and STING in NPCs transfected with si-MED12-1 by Western blot. (**I**) Schematic of the experiment design (**J**). H&E and SO&FG staining of coccygeal vertebrae from rats with the indicated treatment. Scale bars: 1 mm (top), 250 μm (bottom). (**K**) IHC staining of p-p53, γH2AX, and p21 and IF staining of IL-1β in rat coccygeal IVDs. Scale bar: 100 μm. (**L**–**N**) Histological score (*n* = 10) (**L**), Pfirrmann degenerative grades (*n* = 10) (**M**), and disc height index (*n* = 10) (**N**) of rat coccygeal IVDs. Unpaired 2-tailed Student’s *t* test (**H**); data are presented as mean ± SD. One-way ANOVA (**D**, **F**, **L**, **M**, and **N**); data are presented as mean ± SD. At least 3 independent experiments were performed.

On the basis of the observed characteristic loss of MED12 during IVDD, we next sought to systematically investigate its functional role in IVD pathophysiology. RNA-seq was performed on NPCs treated with si-Scramble or si-MED12-1 (Supplemental Figure 7A). GSEA revealed that genes upregulated in the siMED12-1 group were significantly enriched in DNA damage–related pathways and inflammatory response pathways, including “senescence-associated secretory phenotype,” “TNFA signaling via NF-κB,” “P53 pathway,” and “inflammatory response.” In contrast, genes upregulated in the si-Scramble group were enriched in extracellular matrix–related pathways, including “ECM proteoglycans,” “ECM organization,” and “G2M checkpoint” (Figure 6, A and B). Volcano plot analysis showed widespread upregulation of NF-κB–related genes in MED12-knockdown NPCs (Figure 6C). In line with transcriptional dysregulation, genetic depletion of MED12 in normal NPCs caused a senescence-associated inflammatory phenotype, as evidenced by the upregulation of classical senescence markers, concomitant increase in senescence-associated β-galactosidase (SA-β-gal) activity, pronounced γH2AX foci formation indicative of DNA damage, and increased secretion of proinflammatory cytokines (Figure 6, D–F, and Supplemental Figure 7B). Collectively, these results establish that MED12 deficiency induces a proinflammatory NPC phenotype.

The upstream signaling mechanisms of senescence-associated secretory phenotype (SASP) activation are multifactorial and context dependent and vary across senescence-inducing stimuli (38–42). In MED12-knockdown NPCs, GSEA of RNA-seq data showed aberrant activation of cytosolic DNA–sensing pathways (Figure 6G). Consistent with the transcriptomic findings, Western blot analysis revealed that MED12 knockdown in NPCs activated the cGAS/STING pathway, as evidenced by increased p-TBK1 and p-IRF3 levels and a characteristic STING gel mobility shift, compared with scramble control cells (Figure 6H). Strikingly, STING knockdown abrogated the senescence-associated phenotypes induced by MED12 depletion (Supplemental Figure 7C). Furthermore, MED12 depletion mediated by shRNA targeting MED12 (sh-MED12) delivered via AAVs to rat tail IVDs induced progressive disc degeneration, which is characterized by structural and cellular pathologies (Figure 6I and Supplemental Figure 7D). MRI T2-weighted imaging revealed reduced signal intensity in the NP region, concomitant with disc height loss observed via x-ray analysis, collectively reflecting structural compromise (Figure 6, M and N, and Supplemental Figure 7E). Histological assessment revealed disrupted extracellular matrix organization in MED12-deficient discs, in contrast with the well-preserved matrix architecture in controls (Figure 6, J and L). Importantly, our results showed that in sh-MED12 rat caudal IVDs, MED12 expression was reduced, accompanied by enhanced expression of senescence-associated markers (Figure 6K). These findings demonstrate that MED12 depletion promoted NPC senescence in IVDs through a cGAS/STING axis-dependent mechanism.

### MED12 loss induces R-loop accumulation, which drives NPC senescence.

**Figure 7:**
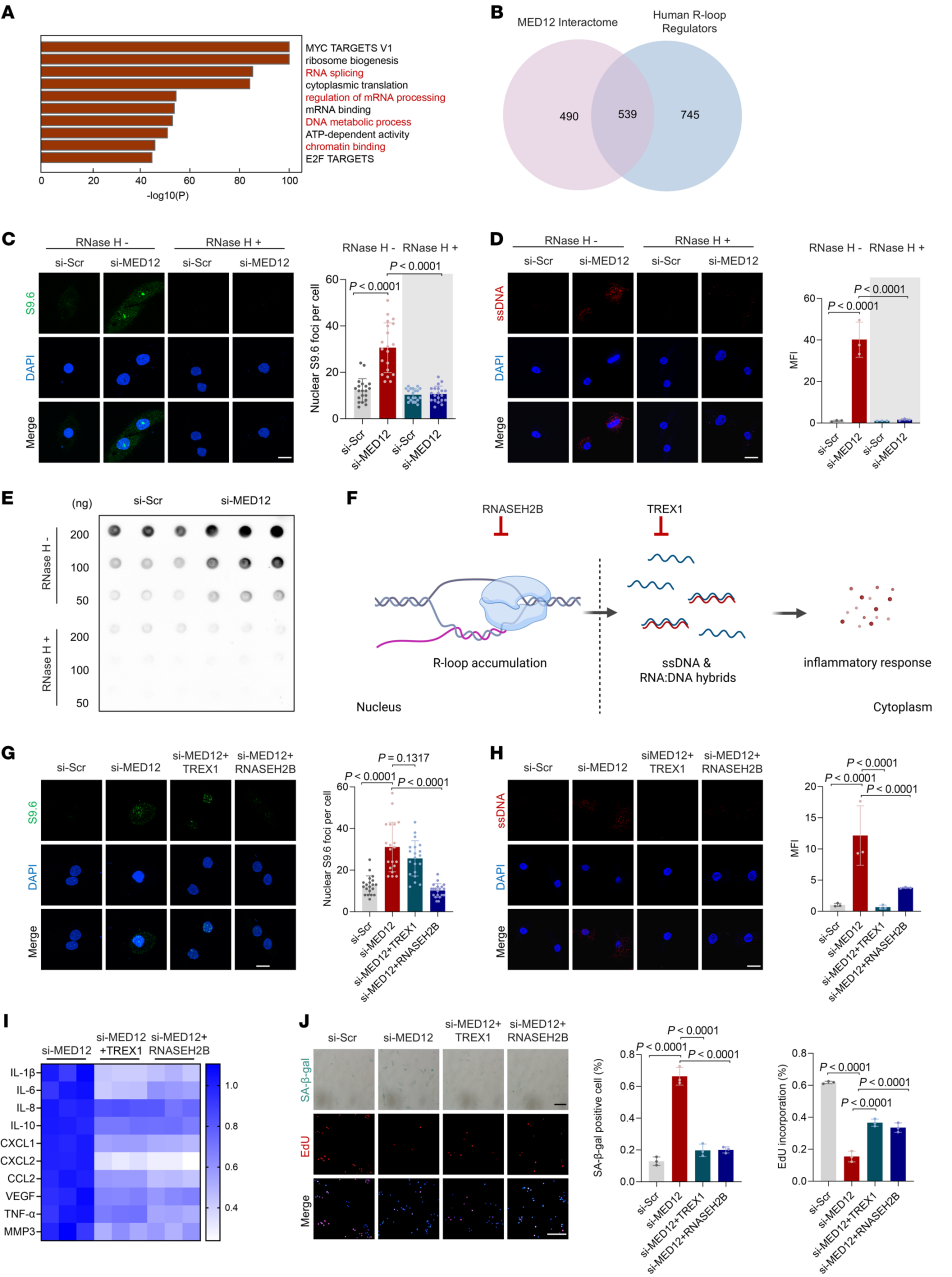
MED12 loss induces R-loop accumulation, which drives NPC senescence. (**A**) Enrichment analysis with Metascape showing terms for protein immunoprecipitated with MED12. (**B**) Venn diagram showing MED12 interactome overlapped with known R-loop regulators. (**C**) Representative IF image of MED12-depleted NPCs stained with S9.6 (green) and DAPI (blue). Scale bar: 10 μm. (**D**) Representative IF image of MED12-depleted NPCs stained with ssDNA (red) and DAPI (blue). Scale bar: 10 μm. (**E**) Dot blot analysis to quantify R-loops in NPCs transfected with si-MED12. RNaseH1 treatment was included as a negative control. (**F**) Schematic of the functional role of abnormal accumulation of pathologic R-loops in chronic inflammation. (**G**) IF staining of R-loop in NPCs with the indicated treatment. Scale bar: 10 μm. (**H**) IF staining of ssDNA in NPCs with the indicated treatment. Scale bar: 10 μm. (**I**) Heatmap of differential SASP in NPCs with the indicated treatment. (**J**) SA-β-gal activity staining and EdU incorporation assay of NPCs transfected with si-MED12. Scale bars: 100 μm. One-way ANOVA (**C**, **D**, **G**, **H**, and **J**); data are presented as mean ± SD. At least 3 independent experiments were performed.

To investigate the upstream mechanisms driving SASP activation in MED12-depleted NPCs, we performed immunoprecipitation–mass spectrometry to profile the MED12 interactome. The analysis identified 1,029 candidate MED12-associated proteins, with enrichment analysis revealing significant clustering in RNA processing and DNA metabolic pathways (Figure 7A). Notably, 539 of these interactors overlapped with known R-loop regulators (Figure 7B) (43). Intriguingly, pathological R-loop accumulation triggers cytoplasmic leakage of self-derived ssDNA and RNA:DNA hybrids, which function as endogenous damage-associated molecular patterns. These aberrant nucleic acids activate cytosolic pattern recognition receptors, including cGAS, triggering STING-dependent inflammatory cascades (41, 44–46). Thus, we hypothesize that cGAS/STING axis–driven inflammatory cascade activation following MED12 depletion could be attributed to pathological R-loop accumulation. Dot blot and IF analyses revealed that MED12 knockdown in NPCs triggered significant R-loop accumulation, as evidenced by increased S9.6 antibody signals. Importantly, administration of RNase H, an enzyme that specifically targets the RNA:DNA hybrid, resulted in a significant reduction in R-loop levels, precluding nonspecific S9.6 cross-reactivity and clearly attributing the observed signals to RNA:DNA hybrid structures (Figure 7, C and E) (47). We next assessed whether R-loop accumulation was accompanied by cytoplasmic ssDNA accumulation. Consistently, elevated cytoplasmic pathological R-loop accumulation and ssDNA levels were observed in MED12-depleted NPCs (Figure 7D).

To mechanistically dissect the interplay between R-loops, cytosolic ssDNA accumulation, and the MED12-associated inflammatory signature, we ectopically expressed the DNA exonuclease TREX1 and the RNA:DNA hybrid–resolving nuclease RNASEH2B in MED12-depleted NPCs (Figure 7F) (46). Consistent with expectations, TREX1 overexpression selectively reduced the number of cytoplasmic RNA:DNA hybrids, whereas RNASEH2B overexpression significantly depleted these hybrid structures in both the nuclear and the cytoplasmic compartments (Figure 7G). Ectopic expression of both TREX1 and RNASEH2B substantially attenuated cytoplasmic ssDNA leakage in MED12-depleted NPCs (Figure 7H). Importantly, overexpression of TREX1 and RNase H1 alleviated the senescence phenotype induced by MED12 depletion, as evidenced by reduced expression of senescence-associated markers, diminished β-galactosidase activity, attenuated SASP activation, and restored cellular proliferative capacity (Figure 7, I and J, and Supplemental Figure 8, A and B). These results demonstrate that MED12 deficiency triggers R-loop accumulation, which in turn activates the cGAS/STING axis–dependent inflammatory cascade.

### PRMT2 deficiency promotes FBXO7/MED12/R-loop axis–dependent NPC senescence.

**Figure 8:**
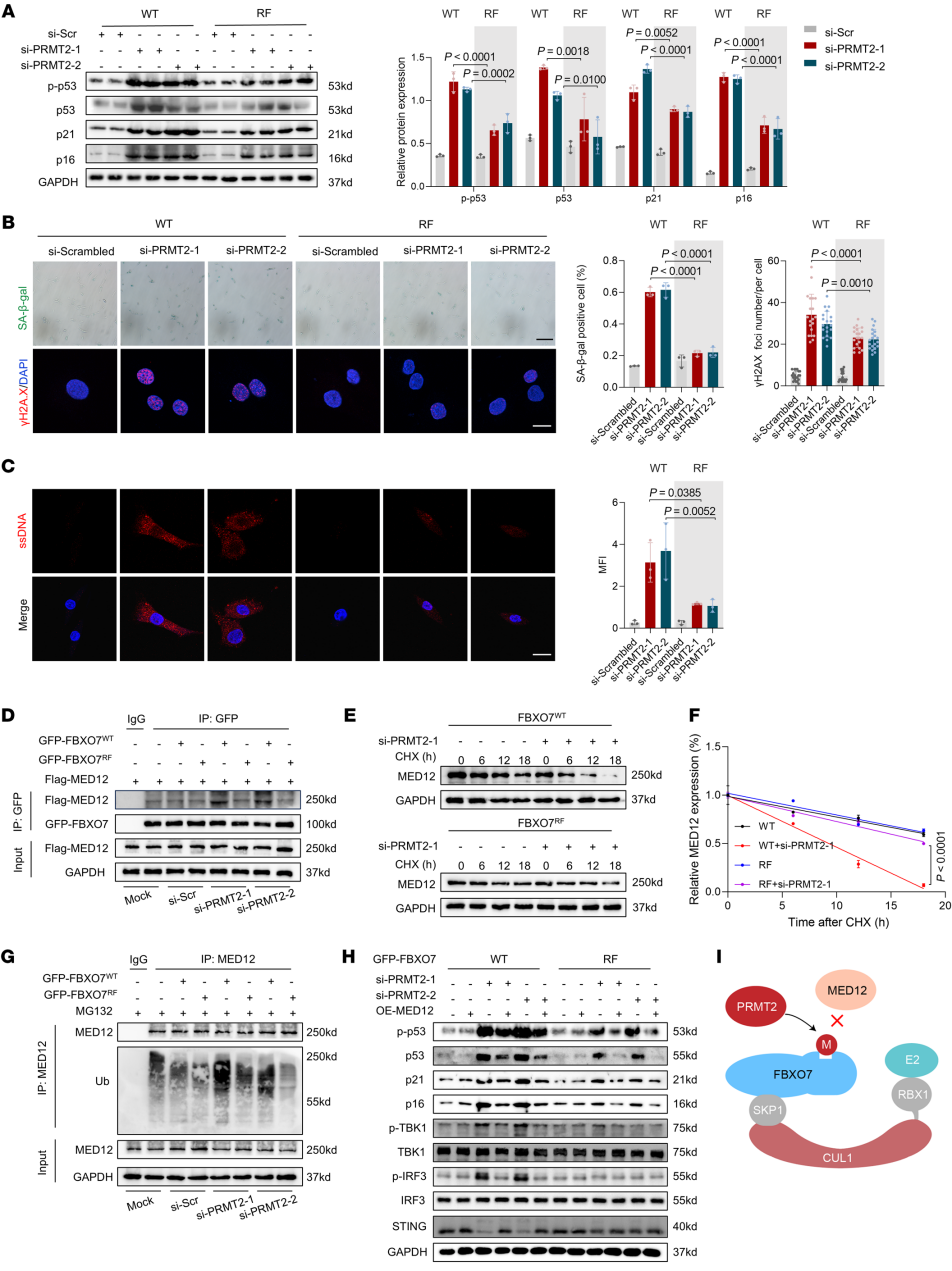
Loss of PRMT2 promotes FBXO7/MED12/R-loop axis–dependent NPC senescence. (**A**) Protein level analysis of p-p53, p53, p21, and p16 in NPCs with the indicated treatment by Western blot. (**B**) SA-β-gal activity and IF staining of γH2AX in NPCs with the indicated treatment. Scale bars: 100 μm (top), 10 μm (bottom). (**C**) IF staining of ssDNA in NPCs with the indicated treatment. Scale bar: 10 μm. (**D**) Co-IP analysis of the interaction of FBXO7 with MED12 in HEK293T cells cotransfected with Flag-MED12 and GFP-FBXO7 (WT and RF mutants) with or without si-PRMT2 transfection. (**E** and **F**) Half-life analysis of MED12 protein in NPCs cotransfected with Flag-MED12 and GFP-FBXO7 (WT and RF mutants) with or without si-PRMT2 transfection. (**G**) Co-IP analysis of endogenous MED12 ubiquitination in NPCs transfected with WT FBXO7 or FBXO7 RF mutant with or without si-PRMT2 transfection. (**H**) Protein level analysis of p-p53, p53, p21, p16, p-TBK1, TBK1, p-IRF3, IRF3, and STING in NPCs with the indicated treatment by Western blot. (**I**) A model of FBXO7-mediated MED12 protein polyubiquitination disrupted by PRMT2. At least 3 independent experiments were performed.

We depleted endogenous FBXO7 and reconstituted it with either WT FBXO7 or the RF mutant in NPCs. Consistent with previous observations, PRMT2 knockdown induced senescence-associated phenotypes characterized by upregulated senescence-associated marker expression, elevated senescence-associated β-galactosidase activity, and genomic instability. Notably, reconstitution with the RF mutant conferred resistance to PRMT2 deficiency–driven senescence acquisition (Figure 8, A and B, and Supplemental Figure 9A). Consistently, PRMT2 knockdown–induced activation of the cGAS/STING pathway was effectively blocked by reconstitution with the RF mutant (Supplemental Figure 9B). Furthermore, reconstituted expression of the FBXO7 RF mutant specifically ameliorated the PRMT2 knockdown–induced proinflammatory hypersecretory state, as evidenced by normalized cytokine expression profiles (Supplemental Figure 9C). Additionally, the PRMT2 knockdown–induced increase in R-loop formation and cytosolic ssDNA accumulation were significantly suppressed in RF mutant–reconstituted NPCs (Figure 8C and Supplemental Figure 9D). Collectively, these data demonstrated that the PRMT2-mediated methylation of FBXO7 at R504 suppressed R-loop formation and mitigated cGAS/STING axis–dependent inflammatory senescence in NPCs.

Next, we investigated the role of PRMT2 in FBXO7-mediated ubiquitin-proteasome degradation of MED12. Co-IP assays demonstrated that PRMT2 knockdown significantly increased the interaction between WT FBXO7 and MED12 in HEK293T cells. Conversely, PRMT2 depletion did not increase the interaction between the recombinantly expressed RF mutant and MED12 (Figure 8D). Moreover, PRMT2 knockdown significantly shortened the half-life of MED12 and increased its ubiquitination level in HEK293T cells transduced with recombinant WT FBXO7 plasmids, whereas these effects were abolished in cells transduced with RF mutant plasmids (Figure 8, E–G). Importantly, MED12 overexpression abrogated the PRMT2 knockdown–induced upregulation of senescence-associated markers (Figure 8H). Collectively, these findings support a model in which PRMT2-mediated methylation of FBXO7 impedes its interaction with MED12 and subsequent FBXO7-driven ubiquitin-dependent degradation of MED12, thereby suppressing pathological R-loop accumulation and ultimately inhibiting cGAS/STING axis–dependent proinflammatory cascades (Figure 8I).

### EV-based MED12-overexpressing plasmid cargo alleviated NPC senescence and IVDD progression.

**Figure 9:**
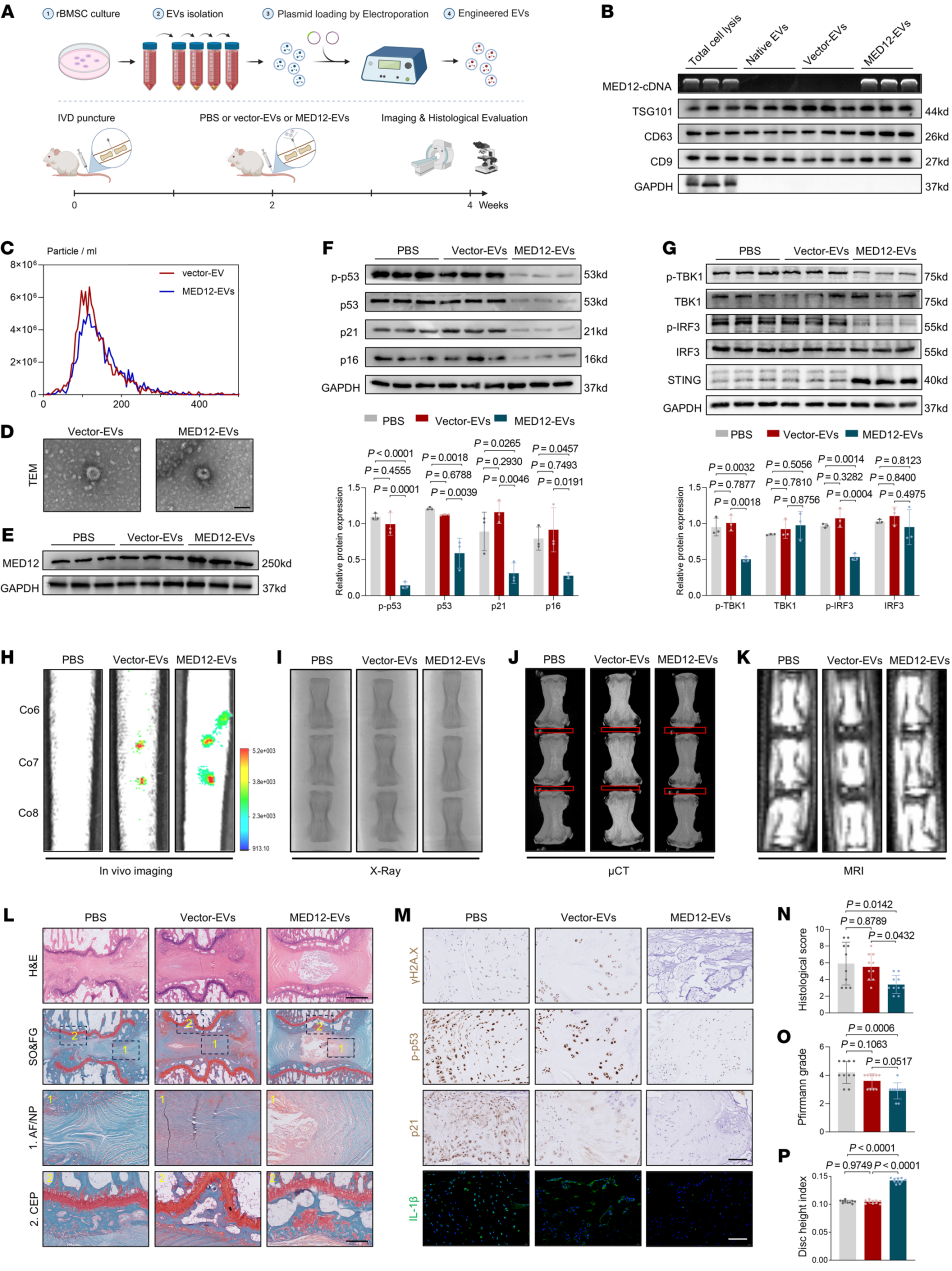
EV-based MED12-overexpressing plasmid cargo alleviated NPC senescence and IVDD progression. (**A**) Schematic showing the experiment design. (**B**) Western blotting analysis of EV markers and cDNA electrophoresis to determine the efficiency of the MED12-overexpressing plasmid loading into rBMSC-derived EVs. (**C**) Nanoparticle tracking analysis of ATR-EVs or vector-EVs. (**D**) Representative transmission electron microscopy (TEM) images of EVs loaded with MED12-EVs or vector-EVs. Scale bar: 100 nm. (**E**) Protein-level analysis of MED12 in rat NPCs with the indicated treatment by Western blot. (**F**) Protein-level analysis of p-p53, p53, p21, and p16 in rat NPCs with the indicated treatment by Western blot. (**G**) Protein-level analysis of p-TBK1, TBK1, p-IRF3, IRF3, and STING in rat NPCs with the indicated treatment by Western blot. (**H**–**K**) Representative in vivo imaging (**H**), x-ray images (**I**), μCT images (**J**), and MRI images (**K**) of coccygeal vertebrae and IVDs from rats treated with the intradiscal injection of MED12-EVs or vector-EVs. (**L**) H&E and SO&FG staining of coccygeal vertebrae from rats with the indicated treatment. Scale bars: 1 mm (top), 250 μm (bottom). (**M**) IHC staining of p-p53, γH2AX, and p21 and IF staining of IL-1β in rat coccygeal IVDs. Scale bar: 100 μm. (**N**–**P**) Histological score (*n* = 10) (**N**), Pfirrmann degenerative grades (*n* = 10) (**O**), and disc height index (*n* = 10) (**P**) of rat coccygeal IVDs. One-way ANOVA (**F**, **G**, **N**, **O**, and **P**); data are presented as mean ± SD. At least 3 independent experiments were performed.

Finally, to explore the translational implications of this discovery, we utilized EVs for MED12 overexpression plasmid delivery. Compared with conventional vectors, EVs present inherent therapeutic advantages, including prolonged extracellular persistence, biological barrier penetrability, low immunogenicity, and abundant tissue availability (48–50). This approach strategically addresses the technical challenges posed by the substantial genomic footprint of the MED12 gene and current packaging limitations, while capitalizing on the demonstrated capacity of EVs for intercellular cargo transfer. We hypothesize that MED12 plasmid-loaded EVs ameliorate NPC senescence and IVDD progression through the restoration of physiological MED12 protein levels. EVs were isolated from rat bone marrow mesenchymal stem cell (rBMSC) culture media via differential ultracentrifugation. Subsequently, MED12-overexpressing plasmids (MED12-EVs) and empty vector plasmids (vector-EVs) were loaded into EVs using optimized electroporation parameters (Figure 9A). Western blot analysis confirmed the presence of canonical EV biomarkers (CD63, CD9, and ALIX), confirming EV identity and purity (Figure 9B) (51). Agarose gel electrophoresis demonstrated efficient loading of MED12-overexpressing plasmids into EVs (Figure 9B). Nanoparticle tracking analysis and transmission electron microscopy showed that MED12-EVs and vector-EVs exhibited irregular spheroidal morphologies with similar sizes (Figure 9, C and D). Notably, MED12-EVs effectively rescued MED12 protein deficiency in degenerated NPCs, as quantified by Western blot analysis (Figure 9E). Consistently, MED12-EVs effectively alleviated senescence-associated phenotypes in degenerated NPCs, which was accompanied by reduced cGAS/STING axis–driven inflammatory cascade activation (Figure 9, F and G, and Supplemental Figure 10, A and B). Next, we evaluated the therapeutic efficacy of MED12-EVs for IVDD in vivo. We established an IVDD model through caudal needle puncture in Sprague-Dawley rats via the intradiscal administration of PBS, vector-EVs, or MED12-EVs (Figure 9A). In vivo fluorescence imaging of DiO-labeled EVs revealed their preferential accumulation at the central region of the IVD, demonstrating effective targeted delivery and confinement to the NP region without extravasation into surrounding tissues (Figure 9H). Furthermore, H&E staining of heart, liver, spleen, lung, and kidney showed no histological alterations or toxicity in the MED12-EV group, supporting its biosafety (Supplemental Figure 10C). Radiological evaluation indicated that MED12-EVs attenuated puncture-induced disc height index reduction and T2-weighted signal loss (Figure 9, I–K, O, and P). Consistently, histological analysis demonstrated the therapeutic effects of MED12-EVs on coccygeal IVDs. Compared with PBS and vector-EV treatment, MED12-EV treatment markedly preserved the proteoglycan content and NP architecture (Figure 9, L and N). Furthermore, the specific localization of pronounced DiO-labeled green fluorescence within NP regions validated the targeted delivery efficiency of EVs, which was functionally correlated with the restoration of MED12 protein levels (Supplemental Figure 10D). To assess long-term retention and the sustained effect of MED12 overexpression, we performed DiO IF and MED12 IHC at 4 and 8 weeks after injection, which revealed persistent EV-derived signal and maintained MED12 expression in the NP region (Supplemental Figure 10, E and F). Furthermore, MED12-EV treatment significantly reduced the number of γH2AX^+^, p-p53^+^, p21^+^, and IL-1β^+^ cells (Figure 9M). In summary, we proposed and validated an EV-based MED12 restoration strategy to mitigate NPC senescence and IVDD progression.

## Discussion

**Figure 10:**
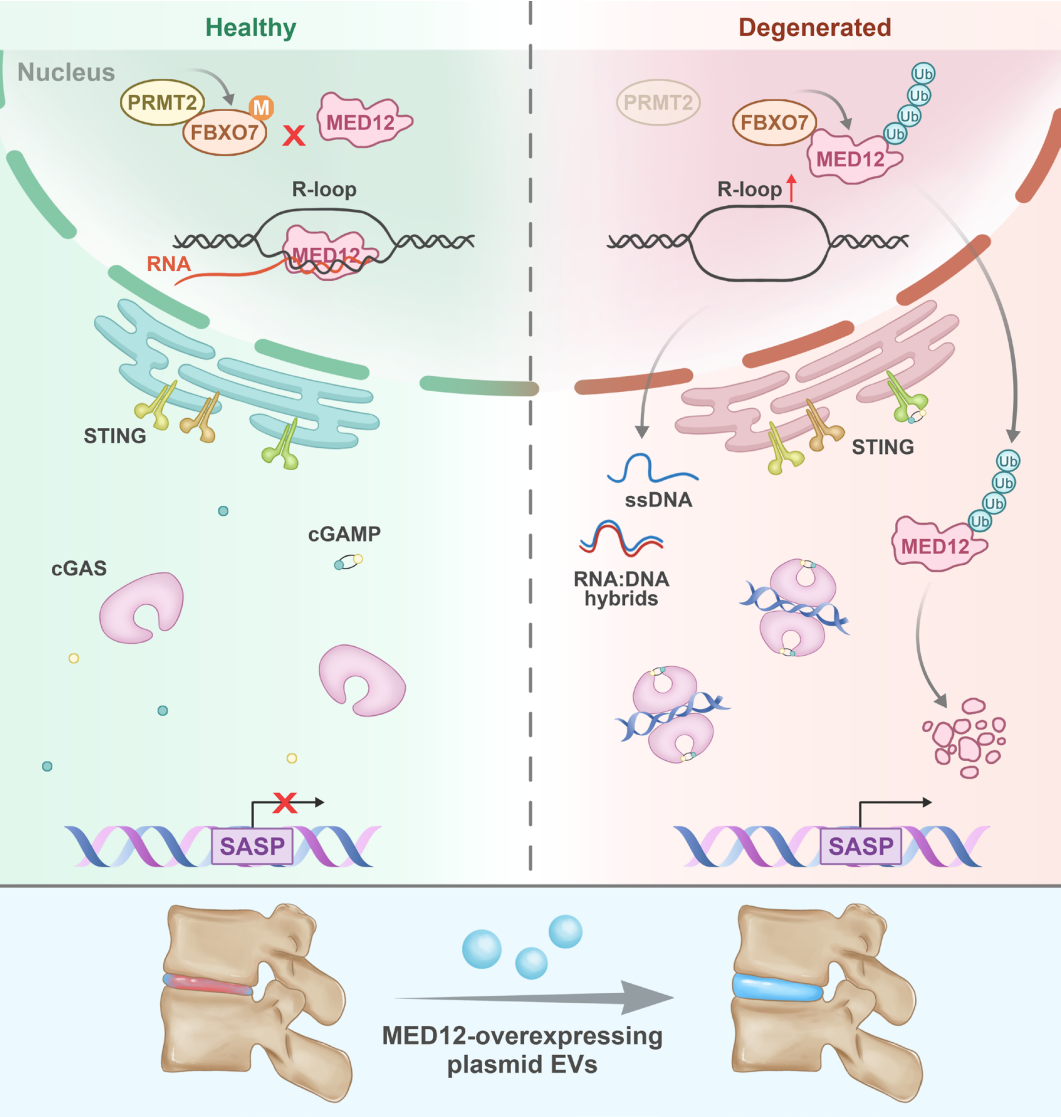
PRMT2 deficiency-mediated FBXO7-MED12 complex assembly promotes MED12 destabilization and R-loop/cGAS/STING axis-dependent IVDD. Schematic illustration showing a mechanism linking methylation-ubiquitination crosstalk of MED12 and R-loop/cGAS/STING axis in NPC senescence and IVDD progression.

In this study, we discovered that dysregulation of the methylation-ubiquitination–mediated destabilization of MED12 could promote abnormal R-loop accumulation, which represents a mechanism driving the inflammatory cascade in IVDD. The loss of MED12 promoted R-loop formation, which activated cytoplasmic DNA sensing–related pathways and aberrantly induced cellular proinflammatory signaling pathways. Targeted restoration of MED12 suppressed senescence in NPCs and alleviated IVDD progression (Figure 10).

Multiple factors may contribute to the development of IVDD, and among these factors, cellular senescence has emerged as a critical risk factor associated with both the incidence and progression of IVDD (2, 4, 52). In-depth investigations of the pathogenesis of IVDD are important for developing targeted treatment strategies. Here, through comparative proteomic profiling of posttranslational events in degenerated versus normal IVDs, we identified PRMT2 deficiency–mediated methylation-ubiquitination as an underlying mechanism driving disc inflammatory degeneration. Previous studies have demonstrated that PRMT2 deficiency promotes inflammatory responses through an NF-κB–dependent pathway (53). In this study, we revealed a mechanism whereby PRMT2 deficiency induced cytoplasmic self-DNA leakage, thereby triggering cGAS/STING-dependent inflammatory cascade activation. Our work identified PRMT2 as a pivotal safeguard against endogenous damage-associated molecular pattern generation, diverging from previous models that focused on the direct interaction of PRMT2 with NF-κB subunits to suppress their transcriptional activity.

IVD aging is accompanied by NPC senescence (52, 54). Senescence limits the self-renewal ability of NPCs, and the limitations of self-renewal potential and the persistence of environmental stresses ultimately lead to tissue inflammation and damage as well as the development of IVDD (55–57). MED12 plays a crucial role in transcriptional regulation, serving as a platform that recruits histone-modifying enzymes, coactivators, and transcription factors while simultaneously acting as a bridge connecting the Mediator complex with RNA polymerase II (34, 35, 58, 59). Intriguingly, the MED12-binding proteome we identified covered a notable portion of R-loop regulators. Coupled with its multifaceted role in transcriptional regulation, this finding prompted a rigorous investigation into how MED12 regulates R-loop homeostasis. Our findings demonstrate that MED12 deficiency drives R-loop accumulation, thereby activating the cGAS/STING axis to promote NPC senescence. Interestingly, previous studies showed that MED12 inhibition derepresses endogenous retrotransposons, which release cytosolic nucleic acids to activate both the type I interferon pathway and the NF-κB signaling pathway (60). Notably, prior work in other cell types has reported that MED12 loss can trigger senescence through alternative mechanisms, such as LIMK2-mediated dysregulation of actin cytoskeleton and cytokinesis failure, or through altered TGF-β signaling (61, 62). These observations indicate that the role of MED12 in senescence is highly context dependent and varies across cell types. In the present study, we propose that MED12 deficiency promotes senescence primarily through dysregulation of R-loop accumulation and activation of the cGAS/STING axis. Thus, our findings reveal a cell type–specific mechanistic insight that extends the known functional repertoire of MED12 into disc degeneration. Future studies elucidating how MED12 scaffolds the assembly of functionally distinct protein complexes are critical to reveal its role in maintaining transcriptional homeostasis and coordinating context-dependent biological programs, offering avenues to target MED12-driven regulatory networks in disease.

Several limitations of this study should be acknowledged. First, due to the difficulty in obtaining age-matched healthy adult disc tissues, we used pediatric scoliosis samples as controls, which introduces a potential age bias. Future studies with larger, properly age-stratified human cohorts are warranted to further dissect the contributions of aging versus degeneration. Second, our in vivo experiments were performed using a rat coccygeal puncture model, which offers excellent accessibility and reproducibility but does not fully replicate the biomechanical environment of the human lumbar spine. Future validation in a lumbar disc degeneration model would enhance translational relevance. Third, although our EV-based MED12 delivery showed efficacy in the rat model, substantial hurdles remain for clinical translation, including large-scale GMP-compliant production, potential immunogenicity, long-term safety, and regulatory approval. Additional efforts to improve EV targeting, reduce off-tissue effects, and optimize manufacturing are essential before any clinical application can be envisioned.

In summary, our findings demonstrated that PRMT2 deficiency promoted MED12 degradation through methylation-ubiquitination crosstalk, driving R-loop/cGAS/STING axis–dependent inflammatory cascades. Furthermore, we engineered EVs encapsulating MED12-overexpressing plasmids, which effectively suppressed NPC senescence and alleviated IVDD progression, identifying MED12 as a potential therapeutic target for IVDD intervention.

## Methods

Additional details may be found in the supplemental materials.

### Sex as a biological variable.

Our study incorporated clinical samples from both male and female donors. Our study exclusively examined female mice, as in our previous studies. It is unknown whether the findings are relevant for male mice.

### Human NP samples.

Human NP tissues were obtained from surgical procedures (lumbar vertebral fracture, idiopathic scoliosis, or IVDD without hereditary diseases) with written informed consent. Clinical medical records and radiographic MRI data were used to evaluate the degenerative grade of the human NP samples according to the Pfirrmann grading system (26). Donor information, including age, sex, clinical diagnosis, and Pfirrmann grade, was anonymized and recorded. The NP samples were subjected to Western blot analysis.

### Cell culture and treatments.

Human NP tissue samples were obtained from surgical disc specimens of patients who underwent lumbar vertebral fracture or idiopathic scoliosis (63). The tissues were minced and digested in 0.4% type II collagenase (Invitrogen) dissolved in DMEM/F12 (Gibco) for 4 hours at 37°C with gentle agitation. The digested cells were centrifuged at 200*g*, rinsed twice with PBS, and resuspended in complete medium containing DMEM/F12 supplemented with 10% FBS (Gibco) and 1% penicillin-streptomycin (Gibco). The cells were cultured at 37°C in a humidified 5% CO_2_ atmosphere. The HEK293T cell line was purchased from the American Type Culture Collection and cultured in DMEM (Gibco) supplemented with 10% FBS (Invitrogen).

To establish an in vitro senescence model, NPCs were treated with 50 μM TBHP (Sigma-Aldrich) for 5 consecutive days (64). To explore the effects of arginine methylation, 20 or 50 μM AdOx (MedChemExpress), a PRMT inhibitor, was used to treat NPCs or HEK293T cells. To explore the dynamic stability of the MED12 protein, NPCs or HEK293T cells were treated with 100 μg/mL cycloheximide (MedChemExpress) to inhibit de novo protein synthesis, 10 μM MG132 (MedChemExpress) for 6 h to block proteasomal function, and 50 μM BafA1 (MedChemExpress) for 6 h to block lysosomal function.

### Animals.

Three-month-old Sprague-Dawley rats (female, 200 ± 20 g) were obtained from the Laboratory Animal Center of Huazhong University of Science and Technology.

### Coccygeal IVD needle puncture IVDD model.

An IVDD model was established in 3-month-old female Sprague-Dawley rats using a validated coccygeal needle puncture protocol. The rats were anesthetized with 3% pentobarbital, and the tail skin was sterilized with iodophor. A 20-gauge needle was inserted perpendicularly into the NP region through the annulus fibrosus under aseptic conditions at the Co6-Co7 and Co7-Co8 coccygeal IVDs. The needle was rotated 180° and held for 10 seconds to induce annular injury (5, 65). Sham-operated controls underwent skin incision without disc puncture. Disc degeneration progression was assessed 4 weeks after injury via radiological analysis (MRI, x-ray, and μCT), histological analysis (H&E and safranin O and fast green [SO&FG] staining), and IHC staining.

### Statistics.

The data in this study are presented as the means ± SD. The data were obtained from at least 3 independent experiments. Unpaired 2-tailed Student’s *t* test was used to analyze the significance between 2 groups, whereas 1-way ANOVA was used to analyze the significance among multiple groups. GraphPad Prism version 9.3.0 was used to analyze the data and statistical results.

### Study approval.

The use of volunteer samples was approved by the Ethics Committee of Tongji Medical College, Huazhong University of Science and Technology (no. S341). All animal experiments were approved by the Laboratory Animal Center of Huazhong University of Science and Technology (no. S2394).

### Data availability.

All individual values represented in graphs are provided in the Supporting Data Values file. The raw sequence data reported in this paper have been deposited in the Genome Sequence Archive (Genomics, Proteomics & Bioinformatics 2025) in the National Genomics Data Center, China National Center for Bioinformation/Beijing Institute of Genomics, Chinese Academy of Sciences (GSA-Human: HRA017941). The mass spectrometry proteomics data have been deposited to the ProteomeXchange Consortium via the iProX partner repository with the dataset identifier PXD0772788. Additional information is available from the corresponding authors upon reasonable request.

## Author contributions

Conceptualization: CY, HL, and YS. Methodology: HL, DZ, ZD, XL, and RS. Investigation: HL, DZ, ZD, XL, RS, DW, XZ, JL, BT, HX, ZO, JW, WK, BW, XF, KW, YS, YD, SP, and ZL. Visualization: HL, DZ, ZD, XL, and RS. Funding acquisition: CY, YS, and HL. Project administration: HL, DZ, and ZD. Supervision: CY and YS. Writing (original draft): YS and HL. Writing (review and editing): CY and YS.

## Conflict of interest

The authors have declared that no conflict of interest exists.

## Funding support

- National Natural Science Foundation of China (82130072, 82072505, and 82472511).
- Creative Research Groups of Hubei Province (2021CFA007).
- Shenzhen Science and Technology Program (SGDX20230116093544006).
- Open Research Project of Hubei Jiangxia Laboratory (2025JXKF014).
- Fundamental Research Funds for the Central Universities, Huazhong University of Science and Technology (YCJJ20242118).

## Supplementary Material

Supplemental data

Unedited blot and gel images

Supporting data values

## Version 1 06/30/2026

In-Press Preview

## Version 2 08/17/2026

Electronic publication
