# Supplementary material for: Dysregulated methylation-ubiquitination crosstalk accelerates intervertebral disc degeneration via MED12 destabilization and cGAS/STING activation
Source: J Clin Invest. 2026 Jun 30;136(16):e197274. doi: 10.1172/JCI197274 (PMC13476149; doi:10.1172/JCI197274)
Supplement: Supplementary file 1 [file jci-136-197274-s185.pdf]

Supplementary Materials for

Dysregulated methylation–ubiquitination crosstalk accelerates intervertebral disc degeneration via MED12 destabilization and cGAS/STING activation

Huaizhen Liang *et al.*

*Corresponding author. Email: caoyangunion@hust.edu.cn

This PDF file includes:

Materials and Methods
Supplemental figure 1 to 10
Supplemental table 1 to 12

Materials and Methods

RNA interference and plasmid transfection

For RNA interference, gene-specific small interfering RNAs (siRNAs) targeting PRMT2, FBXO7, MED12, and STING and nontargeting scrambled siRNAs (as negative controls) were transfected into cells using Lipofectamine 2000 (Invitrogen) following the manufacturer's protocol. The cells were harvested 72 hours posttransfection for subsequent analyses. The knockdown efficiency was validated by western blotting. Further information on the siRNA sequences is provided in Supplementary Table 8.

Human FBXO7 wild type (WT), truncated FBXO7 (amino acids 1-324, 325-522, and Δ332-375), FBXO7 mutants (R504K and R504F) and rat MED12 wild type (WT) were separately tagged with GFP and cloned and inserted into the empty vector plasmid pCDNA3.1 to obtain the overexpression plasmids. Human WT PRMT2 and truncated PRMT2 (amino acids 88-433) were separately tagged with HA and cloned and inserted into the empty vector plasmid pCDNA3.1 to obtain the overexpression plasmids. Human WT MED12 and truncated MED12 (amino acids 1-597, 589-964, 959-1728, and 1616-2177) and mutants (K429R, K762R, K785R, K1001R, K1634R, K1643R, K1664R, K1673R, and K1674R) were separately tagged with Flag and cloned and inserted into the empty vector plasmid pCDNA3.1 to obtain the overexpression plasmids. Human ubiquitin WT, K-only (K48-linked and K63-linked) and K-mutated (K48R or K63R) constructs were cloned and inserted into the empty vector plasmid pmCherry-HA-6×His. For efficient transient transfection, NP cells and HEK293T cells were transfected with the previously described plasmids using Lipofectamine™ 2000 (Invitrogen, USA) for 72 h.

FBXO7-interacting protein sequence analysis by immunoprecipitation mass spectrometry (IP-MS)

Nucleus pulposus cell lysates were subjected to immunoprecipitation using an anti-FBXO7 antibody, and the immunoprecipitated proteins were resolved by SDS-PAGE, excised as a single gel lane, and digested in-gel with trypsin. The resulting peptides were desalted on C18 StageTips and analyzed on a Q-Exactive HF mass spectrometer coupled to an UltiMate 3000 RSLCnano system, using a 60-min gradient and data-dependent acquisition (top 20). Raw MS data were processed with MaxQuant (version 2.2.0.0) and searched against the UniProt human reference proteome (downloaded 20250113) with trypsin/P specificity (up to two missed cleavages), carbamidomethyl (C) as a fixed modification, and oxidation (M), acetyl (protein N-term), and deamidation (NQ) as variable modifications. Mass tolerances were 20 ppm for first search and 4.5 ppm for main search (MS1), and 20 ppm for MS/MS. Peptide-spectrum match, protein, and site false discovery rates were all set to ≤1%.

MED12-interacting protein sequence analysis by IP-MS

Nucleus pulposus cell lysates were subjected to immunoprecipitation using an anti-MED12 antibody, and the immunoprecipitated proteins were resolved by SDS-PAGE, excised as a single gel lane, and digested in-gel with trypsin. The resulting peptides were desalted on C18 StageTips and analyzed on a Q-Exactive Plus mass spectrometer coupled to an Easy-nLC 1200 system, using a 60-min gradient and data-dependent acquisition (top 20). Raw MS data were processed with MaxQuant (version 2.4.14.0) and searched against the UniProt human reference proteome (downloaded 2024-08-02) with trypsin specificity (up to two missed cleavages),

carbamidomethyl (C) as a fixed modification, and oxidation (M) and acetyl (protein N-term) as variable modifications. Mass tolerances were 20 ppm for first search and 4.5 ppm for main search (MS1), and 20 ppm for MS/MS. Peptide-spectrum match, protein, and site false discovery rates were all set to ≤1%.

Western blotting

After the indicated treatments, protein lysates were extracted using RIPA buffer (Boster, China) supplemented with PMSF (MedChemExpress, USA). Protein concentrations were quantified via a BCA assay (Boster, China), and equal amounts of protein (20–50 μg) were resolved by 8–12% SDS–PAGE under reducing conditions. The separated proteins were transferred to PVDF membranes (Millipore), which were blocked with 5% nonfat milk in TBST (Tris-buffered saline with 0.1% Tween-20) for 1 hour at room temperature, followed by incubation with primary antibodies overnight at 4 °C. After washing, the membranes were incubated with HRP-conjugated secondary antibodies for 1 hour at room temperature. The protein bands were visualized using enhanced chemiluminescence (ECL) substrate (Thermo Scientific) and imaged with a ChemiDoc system (Bio-Rad). Densitometric analysis was performed using ImageJ software (NIH), with GAPDH used as a loading control. All experiments included three biological replicates and were repeated at least twice independently.

Reverse transcription quantitative polymerase chain reaction (RT–qPCR)

Total RNA was isolated from cells or tissues using TRIzol reagent (Invitrogen) and quantified via NanoDrop spectrophotometry (Thermo Scientific). Reverse transcription was performed with 1 μg of RNA using the HiScript III RT SuperMix for qPCR (Vazyme, China) according to the manufacturer's protocol. qPCR was carried out in triplicate using SYBR Green Master Mix (Vazyme, China) on a CFX96 Touch sequence detection system (Bio-Rad, USA). Relative mRNA levels were calculated using the 2−ΔΔCt method and normalized to the level of GAPDH. Further information on the primer sequences is provided in Supplementary Table 7.

Chromatin Immunoprecipitation assays (ChIP).

Cells were conducted with corresponding disposal. ChIP was conducted using a Simple ChIP Enzymatic Chromatin IP kit (Cell Signaling Technology), according to the manufacturer's instructions. Antibody of anti-HIF1A was used for immunoprecipitation. Normal rabbit IgG was used as a negative control. Primers of PRMT2 for ChIP-qPCR are listed in Supplementary Table 6.

Generation of Anti- FBXO7-R504me Antibody

The anti-FBXO7-R504me antibody (customized from HUABIO) was generated in rabbits using the peptide GGPNDR(me)FPFRP and ASPNNR(me)FPFRP as antigens. A total of five immunizations were performed. The first immunization dose was 0.75 mg, whereas half of the first dose was used for the next four immunizations. Finally, the serum was obtained for purification.

Bulk-RNA sequencing and Gene set enrichment analysis (GSEA)

Human nucleus pulposus (NP) cells were transfected with si-Scramble or si-MED12-1 (three biological replicates per group) for 48 h. Total RNA was extracted using TRIzol reagent (Thermo Fisher, USA). RNA integrity was assessed using an Agilent 2100 Bioanalyzer, and 1 μg

of total RNA per sample was used for library construction. Libraries were prepared using the MGIEasy RNA Library Prep Kit (MGI Tech, China) and sequenced on an MGISEQ-T7 platform (MGI Tech) with paired-end 150 bp reads, generating approximately 6 G of raw data per sample. Raw reads were quality-controlled using fastp (version 0.23.0). Clean reads were aligned to the human reference genome GRCh38.87. Gene-level read counts were quantified using featureCounts. Differential expression analysis was performed using DESeq2, with genes considered significantly differentially expressed if $|\log_2(\text{fold change})| > 1$ and adjusted p-value < 0.05.
GSEA was conducted using GSEA 4.3.2 software with 1000 permutations and default parameters according to standard procedure. The C2 (curated gene sets) collection and C5 (ontology gene set) collection of the Molecular Signatures Database (MSigDB) version 7.5.1 were used to map overlaps between gene sets in MSigDB and expression set.

Co-immunoprecipitation (Co-IP)
After indicated treatment, treated cells were lysed with NP-40 (Boster, China) supplemented with 1% Cocktail and 1% PMSF (MedChemExpres, USA) for 30 min on the ice, centrifuged at 12000 rpm for 15 min, and the protein content of supernatant was measured by BCA protein assay kit (Boster, China). 1000 µg proteins with the concentration of 1 µg/µl were used to incubate with protein A/G beads (MedChemExpres, USA) conjugated with specific primary antibodies at 4 ºC overnight on a rotator. And the immunoprecipitation was eluted by 1×loading buffer (Boster, USA) and analyzed by western blotting analysis as previous described.

EdU Incorperation Assay
EdU labeling was performed to examine the proliferation status of NPCs. NPCs were exposed to 25µM of 5-ethynyl-2′-deoxyuridine (EdU, RiboBio, C10338, Guangzhou, China) for 12h at 37°C and fixed in 4% paraformaldehyde. NPCs were then permeabilized using 0.5% Triton-X-100 and then reacted with Apollo488 for 30 mins. subsequently, Hoechst 33342 was used to stain the DNA contents of the cells for 30 min, and images were visualized and captured using a microscope (Olympus, BX53). EdU positive cells were analyzed using Image J. The experiments were replicated three times.

R-loop detection using Dot-Blot
Genomic DNA was extracted from NP cells using the TIANamp Genomic DNA Kit (TIANGEN, China) following the manufacturer's protocol. The concentration of DNA was determined using a spectrophotometer. The extracted DNA was then diluted to a standard con centration of 200ng/µl and subjected to further serial dilutions for detailed analysis. As a control, an aliquot of the DNA was also diluted to 200ng/µl in a reaction mixture containing 5units of RNase H (M0297, New England Biolabs) and are action buffer with a water base, to a final volume of 20µl, and then incubated in 37° for 20min. This control setup was similarly serially diluted. Subsequently, 2µl samples from each of these diluted DNA concentrations were carefully spotted onto a nitrocellulose membrane for further experimental processes. The membrane was air-dried for 1h at room temperature and cross-linked using a UV cross-linker (1200µJx100). To block nonspecific binding, the membrane was incubated with 5%nonfatdry milk in Tris-buffered saline with 0.1% Tween 20 (TBST) for 1h at room temperature. Next, the membrane was incubated overnight at 4°C with gentle shaking with an antibody specific to R-loops, S9.6 (Sigma, MABE1095, 1:200) antibody. After washing the membrane three times, it was incubated with a

secondary antibody. R-loop signals were visualized using an enhanced chemiluminescence (ECL) substrate.

SA-β-galactosidase Staining

SA-β-galactosidase staining was performed using a Senescence β-Galactosidase Staining Kit (Beyotime Biotechnology, C0602, Shanghai, China) according to the manufacturer's protocol. Briefly, NPCs were washed with PBS and fixed with the fixative provided in the kit for 20 min at room temperature. After removing the fixative and washing again with PBS, cells were incubated with freshly prepared staining solution at 37 °C for 12 h. Images were captured under a microscope (Olympus, BX53; Melville, NY, USA).

Immunofluorescence

NP cells were planted in the glass coverslip. After the medium was replaced twice, NP cells were exposed to indicated treatment. The cellular coverslip was washed twice with PBS, fixed with 4% paraformaldehyde for 20 min, permeabilized with 0.5% Triton-X-100 for 15 min, blocked with normal goat serum for 30 min, incubated with primary antibodies at 4 ºC overnight, washed twice with PBS supplemented with 0.1% tween-20 and incubated with secondary antibodies of anti-mouse/rabbit Alexa Fluor 488 nm or 568 nm. Cellular nucleus was stained with DAPI (Invitrogen, USA). The fluorescent images were captured using confocal imaging (Olympus, FV3000).

Identification of methylated protein and residues by mass spectrometry

Arginine mono-methylated peptides were enriched by immunoprecipitation (IP) using asymmetric mono-methyl arginine (me-R) antibodies with protein A/G beads (MedChemExpres, USA) at 4 ºC overnight on a rotator. After washed 5 times with pre-cooled NP-40 lysis buffer, immunoprecipitates were eluted with 1×loading buffer (Boster, USA) and separated SDS-PAGE gel. According to the manufacture's protocol, SDS-PAGE gel piece was decolorized, alkylated and enzymatically disintegrated. The samples were analyzed by liquid chromatography–tandem mass spectrometry (LC-MS/MS) on a Q Exactive mass spectrometer (Thermo Fisher Scientific). The LC-MS/MS data were processed using Proteome Discoverer (Thermo Fisher Scientific) and searched against the Swiss-prot Homo sapiens protein sequence database.

EVs isolation and plasmid loading

Rat bone marrow mesenchymal stem cells (rBMSCs) were obtained from the bilateral femurs of four-month-old SD rats, isolated by density gradient centrifugation and adhered to tissue culture plastic. rBMSCs were cultured in the 1:1 DMEM: F12 growth medium combined with 10% FBS and 1:100 penicillin-streptomycin in the incubator with 5% CO2 at 37 °C condition. The medium was changed every three days. After rBMSCs were passaged to second generation, the medium was replaced with 1:1 DMEM: F12 treated with 15% EV-free FBS (System Bioscience, USA) and collected for isolate EVs. EVs were purified according to the differential ultracentrifugation protocols (49, 88, 104, 105). Briefly, collected medium was centrifuged sequentially at 600×g for 10 min, 2000×g for 30 min and 10000×g for 30 min to remove dead cells or cell debris, and ultra-centrifuged at 20000×g for 60 min and washed twice with PBS to isolate EVs. Finally, EVs were suspended with PBS and stored in 4°C for further experiments. BCA protein assay kit (Boster, China) was using to measure the protein concentration of EVs. A total of 4 μg vector or

MED12-overexpressing plasmids were mixed with 200 µl EVs (100 µg/ml). Then the mixtures were transferred into 4 mm electroporation cuvettes and electroporated at 0.150 Kv/100 µF.

EVs identification

For observing the morphology of engineered EVs, a total of 20 µl EVs was loaded onto a formvar/carbon-coated grid for 5 min. after dried on bibulous paper, the grid was immediately negatively stained with 3% aqueous phosphotungstic acid for 1 min, and dried overnight. The grid was pictured using transmission electron microscopy (FEI Tecnai G20 TWIN, USA). For analyzing the concentration and size of engineered EVs, a total of 20 µl EVs were diluted 100- to 500-fold with precooled PBS for measurement. ZetaView Multiple Parameter Particle Tracking Analyzer (Particle Metrix) was using to assess the number and size of EVs via nanoparticle tracking analysis. For identifying protein markers of EVs, western blotting was used to analyze the protein content of EVs involved in EV-markers including CD9, CD63 and TSG101. Briefly, as described previously, protein concentration was measured by BCA protein assay kit and a total of 10 µg protein were separated on 10% SDS-PAGE gel, transferred to 0.45 µm PVDF membranes and incubate with special primary antibodies. Cellular proteins were used as positively condition. For evaluating the loading of MED12-overexpressing plasmids, a total of 200 µl EVs (100 µg/ml) was used to exact DNA cargo in engineered EVs via TIANamp Genomic DNA Kit (TIANGEN, China). Isolated DNA cargo was amplified by using the special primers of MED12 gene, and quantitatively analyzed by performing qPCR as previously described.

Intradiscal injection of EVs

After the initial coccygeal IVDs needle puncture, rats randomly and double-blindly assigned to 3 treatment groups with 5 rats in each group. After anesthesia with 3% pentobarbital, a total of 2 µl solution containing PBS, vector-EVs (100 µg/L) and MED12-EVs (100 µg/L) was slowly injected into the center of NP regions of Co6-7, Co7-8 and Co8-9 coccygeal IVDs by using a 33-gauge needle. Immediately, the rats were sacrificed for in vivo imaging analysis to confirm that engineered EVs were injected into the NP regions of coccygeal IVDs in vivo, and after one month, the rats were sacrificed for radiographic and histological analysis.

Intradiscal injection of AAVs

Three-month-old female Sprague-Dawley rats were randomly divided into multiple groups (n = 5 per group). After anaesthesia with 3% pentobarbital, a total of 2 µL of solution containing the indicated AAVs at a titer of 10^9 plaque-forming units was slowly injected into the centre of the nucleus pulposus region of the Co6-Co7 and Co7-Co8 coccygeal intervertebral discs using a 33-gauge needle (Hamilton) attached to a microliter syringe (Hamilton). An adeno-associated virus (AAV) containing short hairpin RNA targeting PRMT2, MED12, HIF1A, FBXO7, or a scrambled sequence, as well as AAVs for overexpression of FBXO7 (wild-type or R504F mutant) or PRMT2, were generated. For ubiquitous expression, AAVs were constructed using the pAAV5-CMV-EGFP system according to the manufacturer's protocols. For nucleus pulposus (NP)-specific expression, AAVs were constructed using the AAV5-col2a1-T2A-mCherry system, in which the col2a1 promoter drives transgene expression specifically in NP cells. The AAV constructs driven by the CMV promoter included AAV-sh-Scramble, AAV-sh-HIF1A, AAV-sh-PRMT2-1, AAV-sh-PRMT2-2, AAV-sh-MED12-1, AAV-sh-MED12-2, and AAV-PRMT2. The AAV constructs driven by the col2a1 promoter

(NP-specific) included AAV-sh-FBXO7, AAV-FBXO7-WT, and AAV-FBXO7-RF. After one month, the rats were sacrificed for radiographic and histological analysis.

Immunohistochemistry (IHC)
Slides of human NP samples or rat coccygeal IVD specimens were deparaffinized with xylene, rehydrated with ethanol in gradient concentrations, antigen-retrieved with citrate buffer, blocked with normal goat serum, incubated with special primary antibodies overnight. After incubated with secondary antibodies and visualized with DAB peroxidase substrate kit, histological images were analyzed using the BX53 microscope (Olympus).

Histological and Radiographic Evaluation and Analysis
One months later, rats were sacrificed and histological and radiographic evaluation were performed. After X-ray and MRI examination, tails were fixed in 10% neutral-buffered formalin for 1 week and Midsagittal sections were stained with hematoxylin and Safranin O-fast green to evaluate the degeneration level. The histological evaluation was performed according to histologic grading system developed by Ji et al. More specifically, the cellularity and morphology of the AF, NP, and the border between the two structures were examined. The scale is based on 5 categories of degenerative changes with scores ranging from 0 points (0 in each category) for a normal disc to 15 points (3 in each category) for a severely degenerated disc. For morphology of the NP, score 0: round shape and the NP constitutes >75% of the disc area, score 1: round shape and the NP constitutes 50–75% of the disc area, score 2: round shape and the NP constitutes 25–50% of the disc area, score 3: round shape and the NP constitutes <25% of the disc area. For cellularity of the NP, score 0: stellar-shaped cells with a proteoglycan matrix located at the periphery, evenly distributed, score 1: partially stellar and partially round cells, more stellar than round, score 2: mostly large, round cells, separated by dense areas of proteoglycan matrix, score 3: large, round cells, separated by dense areas of proteoglycan matrix. For morphology of the AF, score 0: well-organized collagen lamellae with no ruptures, score 1: inward bulging, ruptured, or serpentine fibers constitute <25% of the AF, score 2: inward bulging, ruptured, or serpentine fibers constitute 25−50% of the AF, score 3: inward bulging, ruptured, or serpentine fibers constitute >50% of the AF. For cellularity of the AF, score 0: fibroblasts comprise >90% of the cells, score 1: fibroblasts comprise >75–90% of the cells, score 2: intermediate, score 3: chondrocytes comprise >75% of the cells. For border between the NP and AF, score 0: normal, without any interruption, score 1: minimal interruption, score 2: moderate interruption, score 3: severe interruption. Radiographs were taken at 4 weeks after the puncture. The change in IVD height was evaluated by the disc height index (DHI) The change in IVD height was evaluated by the disc height index (DHI) Measurements of internal control discs were carried out together with their corresponding punctured discs. Disc height and the adjacent vertebral body heights were measured on the midline and 25% of the disc's width from the midline on either side. The DHI was expressed as the mean of the 3 measurements from midline to the boundary of the central 50% of disc width divided by the mean of the 2 adjacent vertebral body heights. Changes in the DHI of punctured discs were expressed as a percentage (%DHI=post-punctured DHI/pre-punctured DHI ×100). MRI-based Pfirrmann grading system was applied to assess the degenerative grade of the NP specimens.

Supplemental figure 1.

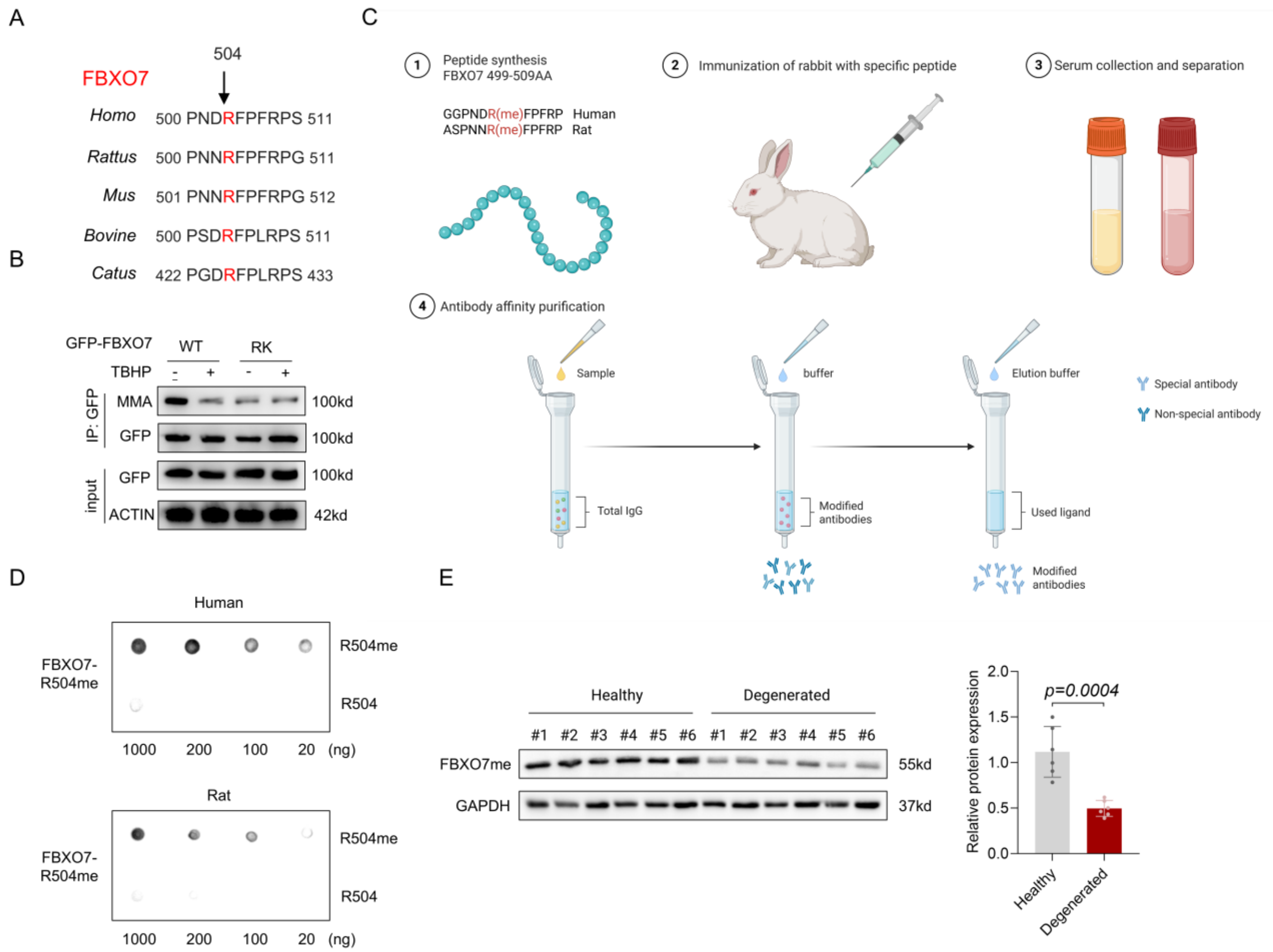

Supplemental figure 1. (A) Alignment of amino acid sequences of FBXO7 across all species showing Arg504 highlighted in red. **(B)** Co-IP analysis of methylated FBXO7 in NPCs transfected with GFP-tagged wild-type FBXO7 or FBXO7 RK mutants. **(C)** Schematic diagram of the experimental design for the generation of anti-FBXO7-R504me antibodies. **(D)** The specificity of the anti-FBXO7-R504me antibody was verified by dot blot. **(E)** Protein level analysis of FBXO7-R504me in human NP tissues by western blot. Unpair students' t test (**E**) was used to performed, and data are represented as mean ± SD. At least 3 independent experiments were performed.

Supplemental figure 2.

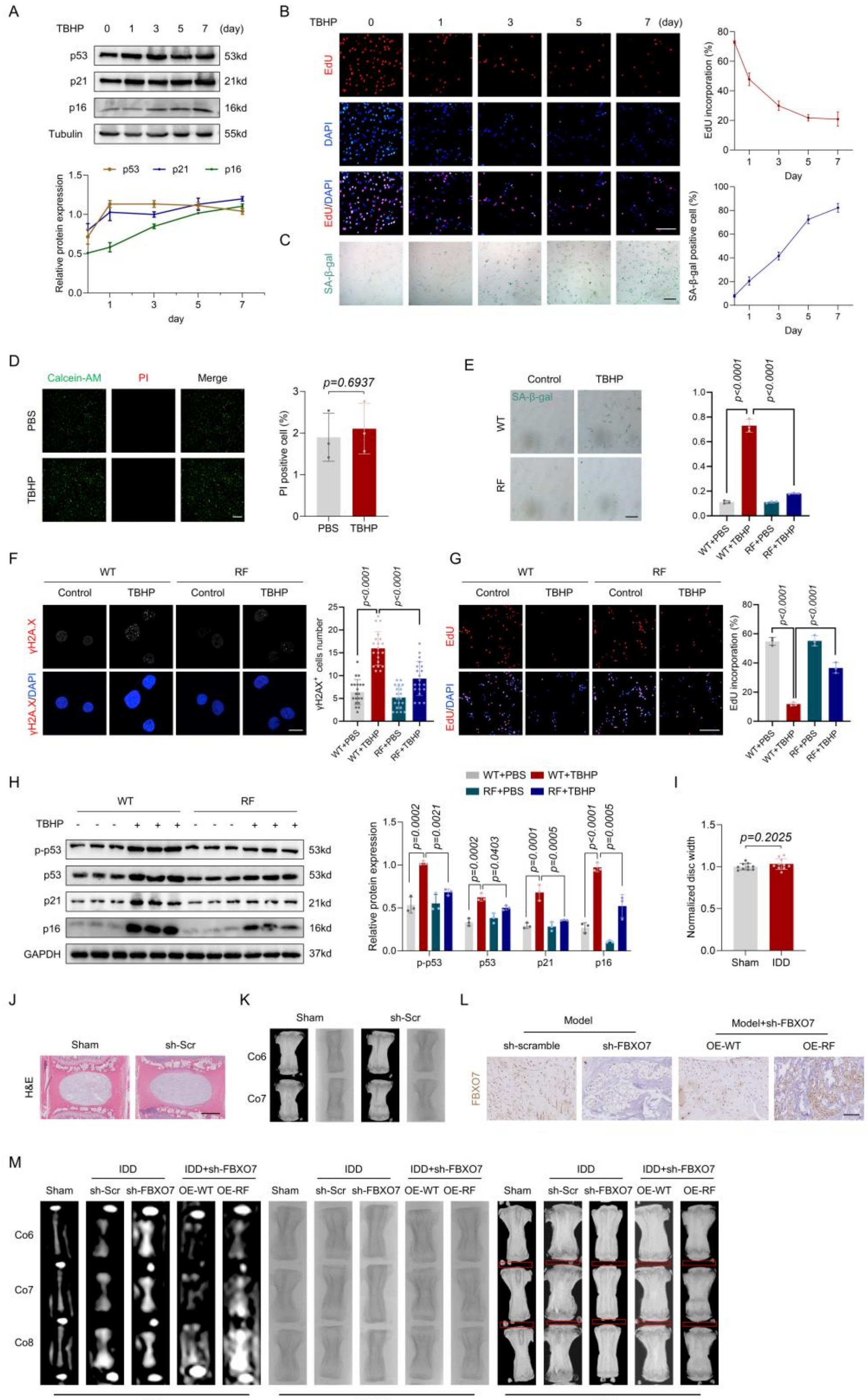

Supplemental figure 2. (A) Western blot analysis of p53, p21, p16 in human NPCs with the indicated treatment. **(B)** EdU incorporation assay and analysis of NPCs with the indicated treatment (Scale bar: 100 μm). **(C)** SA-β-gal activity staining and analysis of NPCs with the indicated treatment (Scale bar: 100 μm). **(D)** NPC viability was evaluated through live/dead fluorescence staining with the indicated treatment (Scale bar: 200 μm). (**E**) SA-β-gal activity staining and analysis of NPCs reconstituted expression of wild-type FBXO7 or FBXO7 RK mutant with the indicated treatment (Scale bar: 100 μm). (**F**) IF staining of γH2A.X foci and analysis of NPCs reconstituted expression of wild-type FBXO7 or FBXO7 RK mutant with the indicated treatment (Scale bar: 10 μm). (**G**) EdU incorporation assay and analysis of NPCs reconstituted expression of wild-type FBXO7 or FBXO7 RK mutant with the indicated treatment (Scale bar: 100 μm). (**H**) Western blot analysis of p-p53, p53, p21, p16 in human NPCs reconstituted expression of wild-type FBXO7 or FBXO7 RK mutant with the indicated treatment. (**I**) Disc width was measured on H&E-stained mid-sagittal sections and normalized to disc width measured on X-ray images. (**J**) H&E staining of coccygeal vertebrae from rats with the indicated treatment (Scale bar: 1 mm). (**K**) Representative X-ray images, μCT images of coccygeal vertebrae and IVDs from rats with the indicated treatment. (**L**) IHC staining analysis of the knockdown efficiency of sh-FBXO7 in NP tissues, and the overexpression efficiency of WT and RF mutant FBXO7 in NP tissues. (**M**)Representative X-ray images, μCT images, MRI images of coccygeal vertebrae and IVDs from rats with the indicated treatment. Unpair students' t test (**D**, **I**) was used to performed. One-way ANOVA (**E**, **F**, **G**, **H**) was used to performed, and data are represented as mean ± SD. At least 3 independent experiments were performed.

Supplemental figure 3.

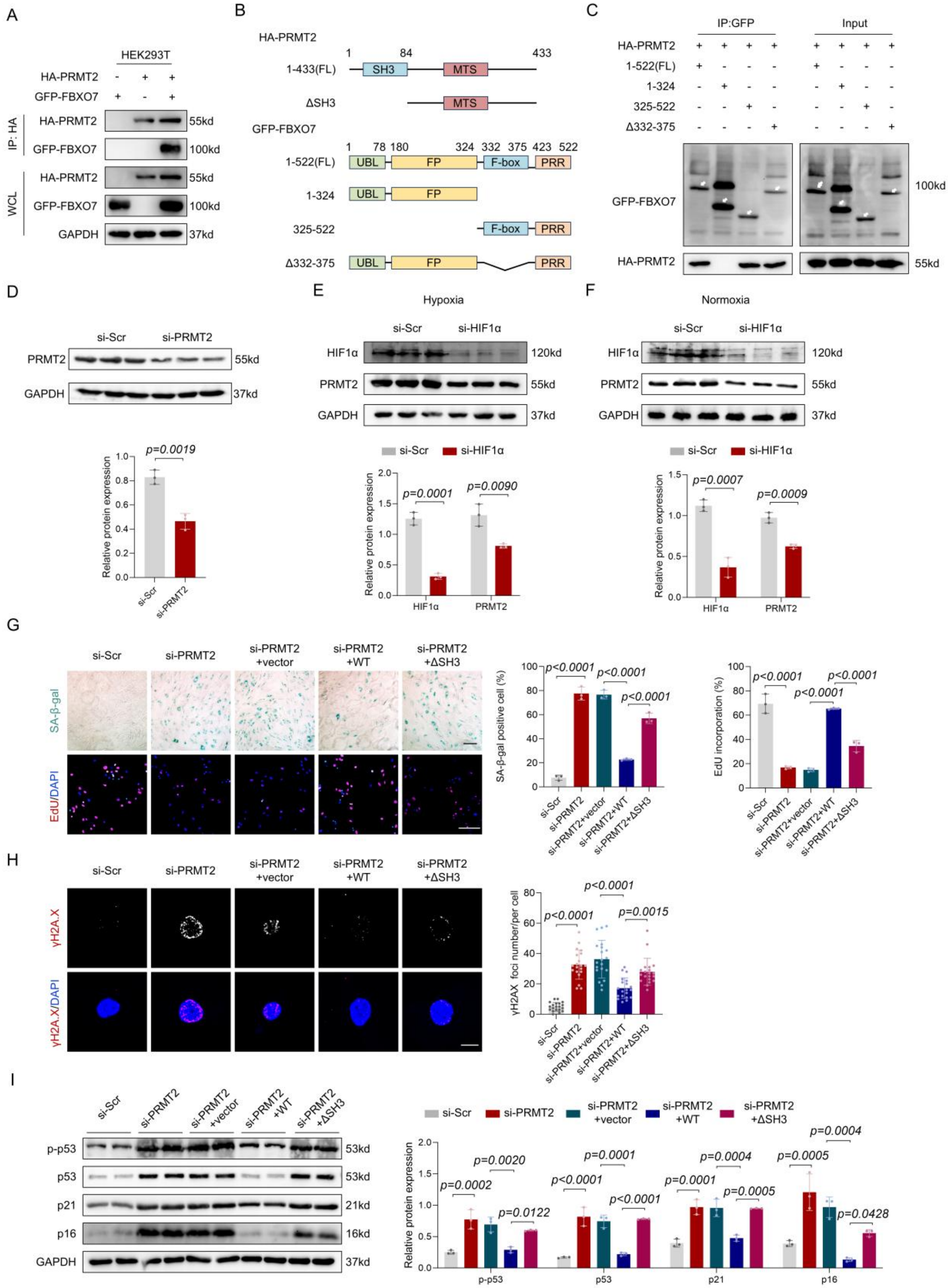

Supplemental figure 3. (**A**) Exogenous Co-IP analysis of the interaction of FBXO7 with PRMT2 in HEK293T cells cotransfected with GFP-FBXO7 and HA-PRMT2. (**B**) A schematic presentation of FBXO7 and PRMT2 domains. (**C**) GFP-FBXO7 FL or indicated truncation mutants were co-expressed with HA-PRMT2 in HEK293T cells. Proteins were immunoprecipitated using GFP antibody and analyzed by western blotting. (**D**) Western blot analysis of PRMT2 in NPCs with PRMT2 silencing. (**E**) Western blot analysis of PRMT2 and HIF1α in NPCs with HIF1α silencing under hypoxia. (**F**) Western blot analysis of PRMT2 and HIF1α in NPCs with HIF1α silencing under normoxia. (**G**) SA-β-gal activity staining and EdU incorporation assay of NPCs with PRMT2 silencing accompanied with overexpression of wild-type or mutant PRMT2 (Scale bar: 100 μm). **(H)** IF staining of γH2A.X foci and analysis of NPCs with PRMT2 silencing accompanied with overexpression of wild-type or mutant PRMT2 (Scale bar: 10 μm). (**I**) Western blot analysis of p-p53, p53, p21, p16 in human NPCs with PRMT2 silencing accompanied with overexpression of wild-type or mutant PRMT2 by western blot. Unpair students' t test (**D, E, F**) were used to performed, and data are represented as mean ± SD. One-way ANOVA (**G**, **H, I**) was used to performed, and data are represented as mean ± SD. At least 3 independent experiments were performed.

Supplemental figure 4.

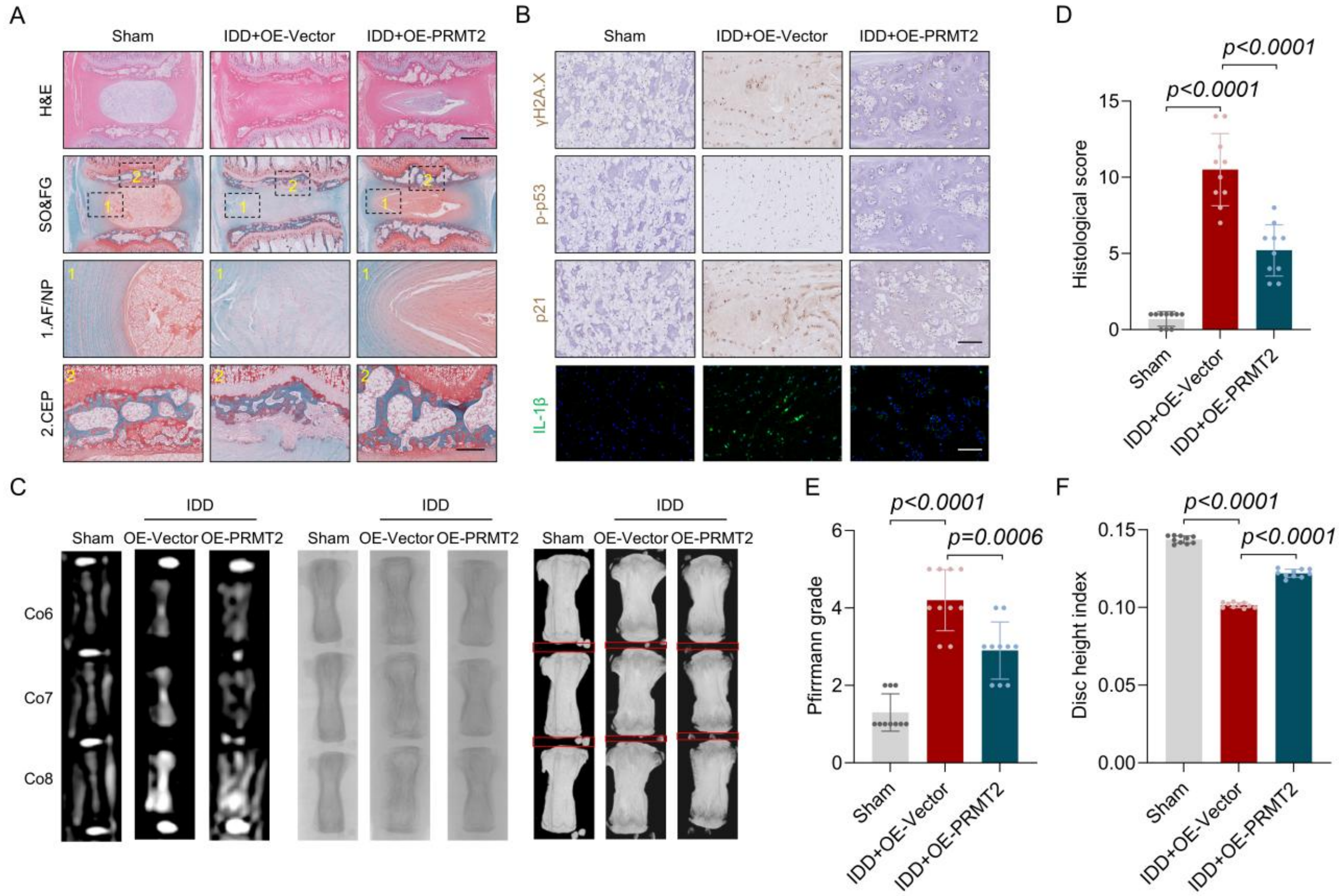

Supplemental figure 4. (**A**) H&E staining SO&FG staining of coccygeal vertebrae from rats with the indicated treatment (Scale bar: 1 mm, 250 μm). (**B**) IHC staining and IF staining of p-p53, γH2A.X, p21 and IL-1β in rat coccygeal IVDs (Scale bar: 100 μm) (**C**) Representative X-ray, μCT images and MRI images of coccygeal vertebrae from rats with the indicated treatment. Representative (**D**) Histological score (n = 10), (**E**) Pfirrmann degenerative grades (n = 10) and (**F**) disc height index (n = 10) of rat coccygeal IVDs. One-way ANOVA (**D**, **E**, **F**) was used to performed, and data are represented as mean ± SD. At least 3 independent experiments were performed.

Supplemental figure 5.

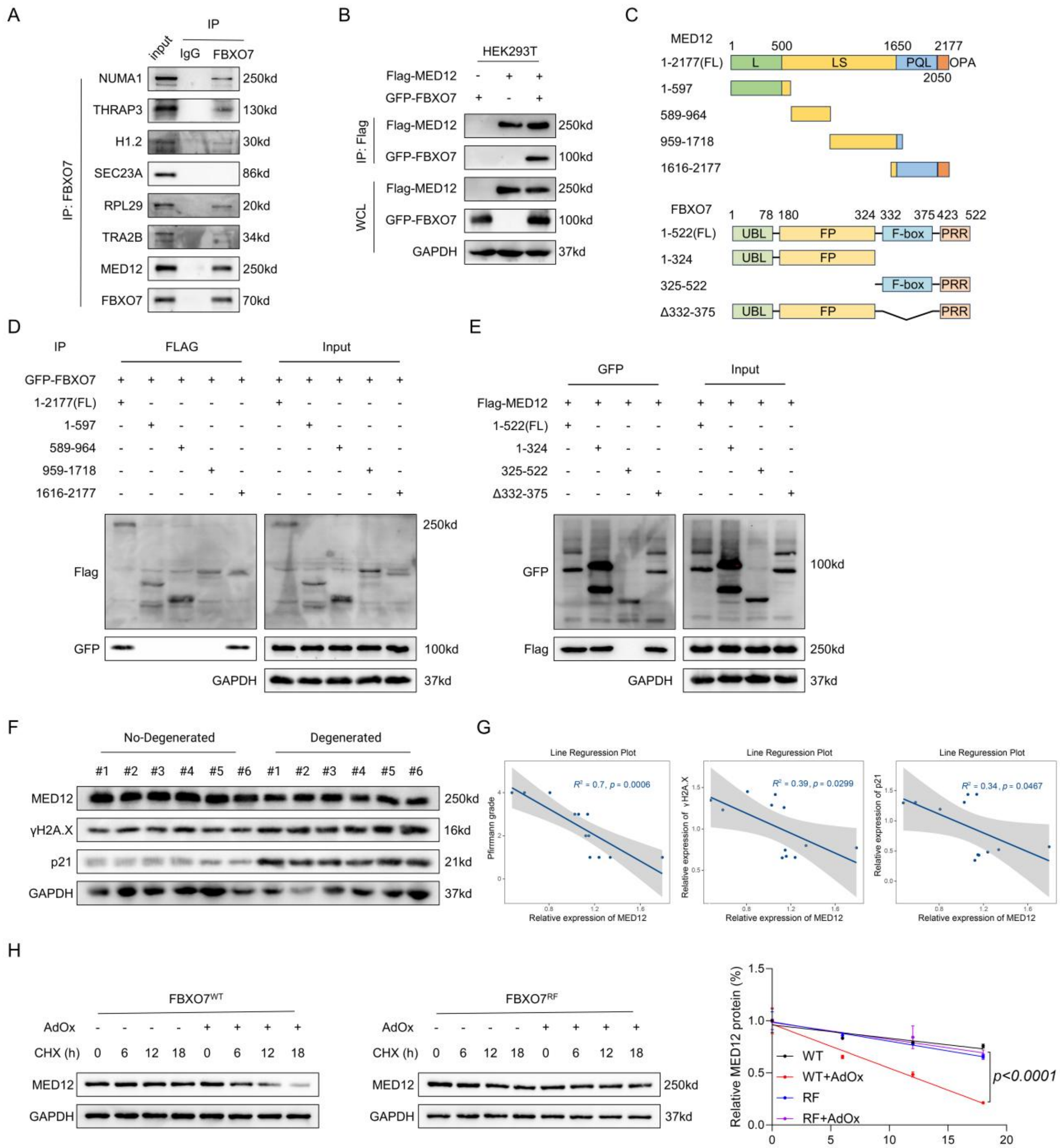

Supplemental figure 5. (**A**) Endogenous Co-IP analysis of the interaction of FBXO7 with 7 protein identified by IP-MS in NPCs. (**B**) Exogenous Co-IP analysis of the interaction of FBXO7 with MED12 in HEK293T cells cotransfected with GFP-FBXO7 and Flag-MED12. (**C**) A schematic presentation of FBXO7 and PRMT2 domains. (**D**) Flag-MED12 FL or indicated truncation mutants were co-expressed with GFP-FBXO7 in HEK293T cells. Proteins were

immunoprecipitated using Flag antibody and analyzed by western blotting. (**E**) GFP-FBXO7 FL or indicated truncation mutants were co-expressed with Flag-MED12 in HEK293T cells. Proteins were immunoprecipitated using GFP antibody and analyzed by western blotting. (**F**) Protein level analysis of MED12, γH2A.X, p21 in human NP tissues by western blot. (**G**) Linear regression analysis between the MED12 protein levels and the Pfirrmann MRI grades, linear regression analysis between the MED12 protein levels and the γH2A.X, p21 protein levels. (**H**) Half-life analysis of MED12 protein in HEK293T cells cotransfected with Flag-MED12 and GFP-FBXO7 (wild-type, RF mutants) with the indicated treatment. Simple linear regression (**G**) was used to performed. One-way ANOVA (**H**) was used to performed, and data are represented as mean ± SD. At least 3 independent experiments were performed.

Supplemental figure 6.

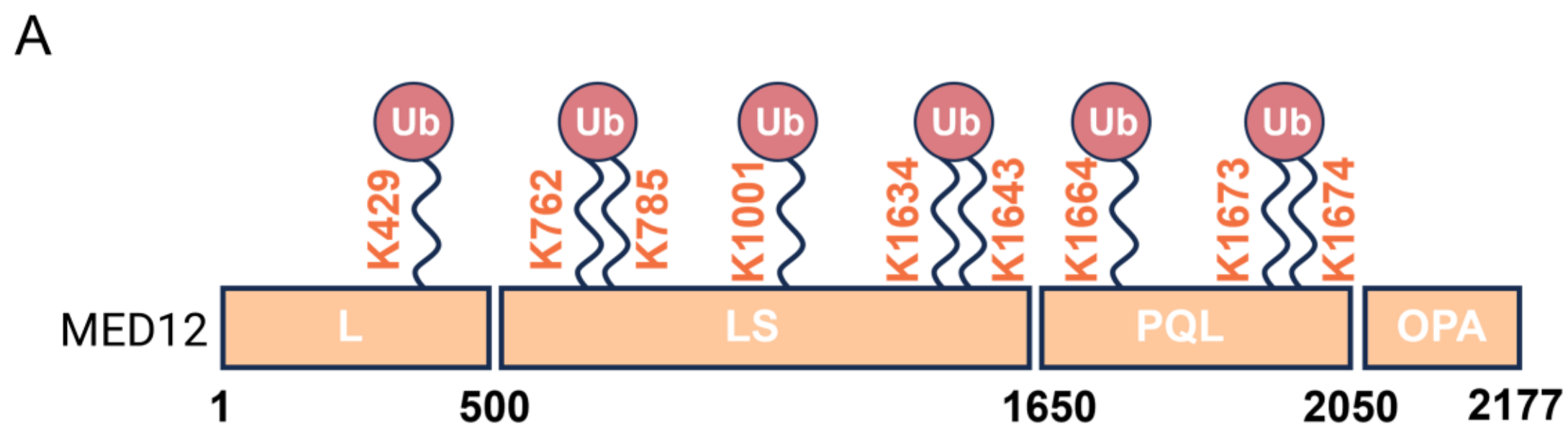

Supplemental figure 6. (A) Ubiquitination sites in MED12 protein predicted by GPS-Uber.

Supplemental figure 7.

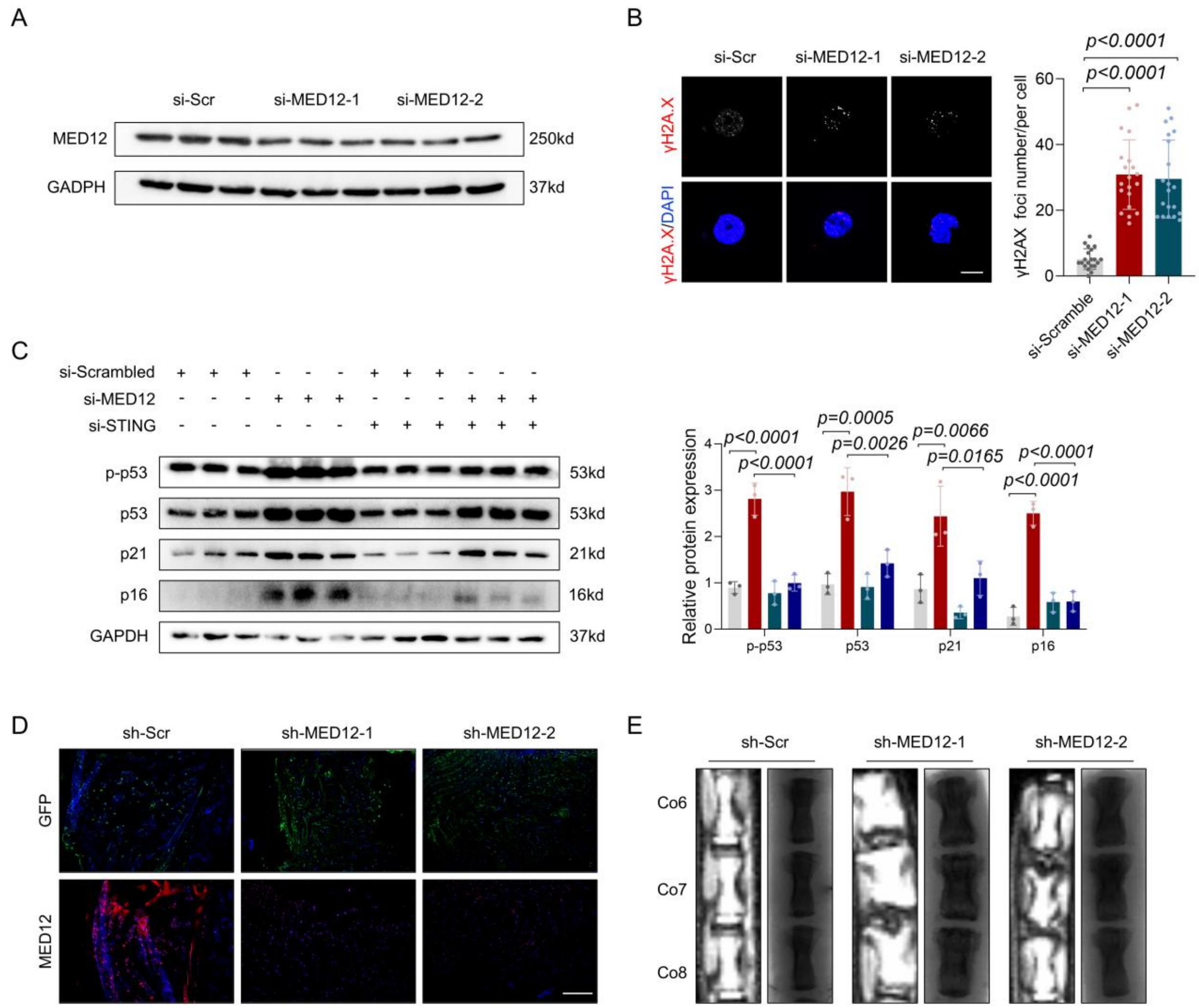

Supplemental figure 7. (A) Protein level analysis of MED12 in NPCs after treatment with specific siRNAs targeting MED12 by western blot. **(B)** γH2A.X foci analysis of NPCs with the indicated treatment (Scale bar: 10 μm). **(C)** Protein level analysis of p-p53, p53, p21, p16 in NP cells with the indicated treatment. **(D)** IF staining of GFP and MED12 of coccygeal vertebrae from rats with the indicated treatment (Scale bar: 100 μm). **(E)** Representative X-ray and MRI images of coccygeal vertebrae from rats with the indicated treatment. One-way ANOVA (**B**, **C**) was used to performed, and data are represented as mean ± SD. At least 3 independent experiments were performed.

Supplemental figure 8.

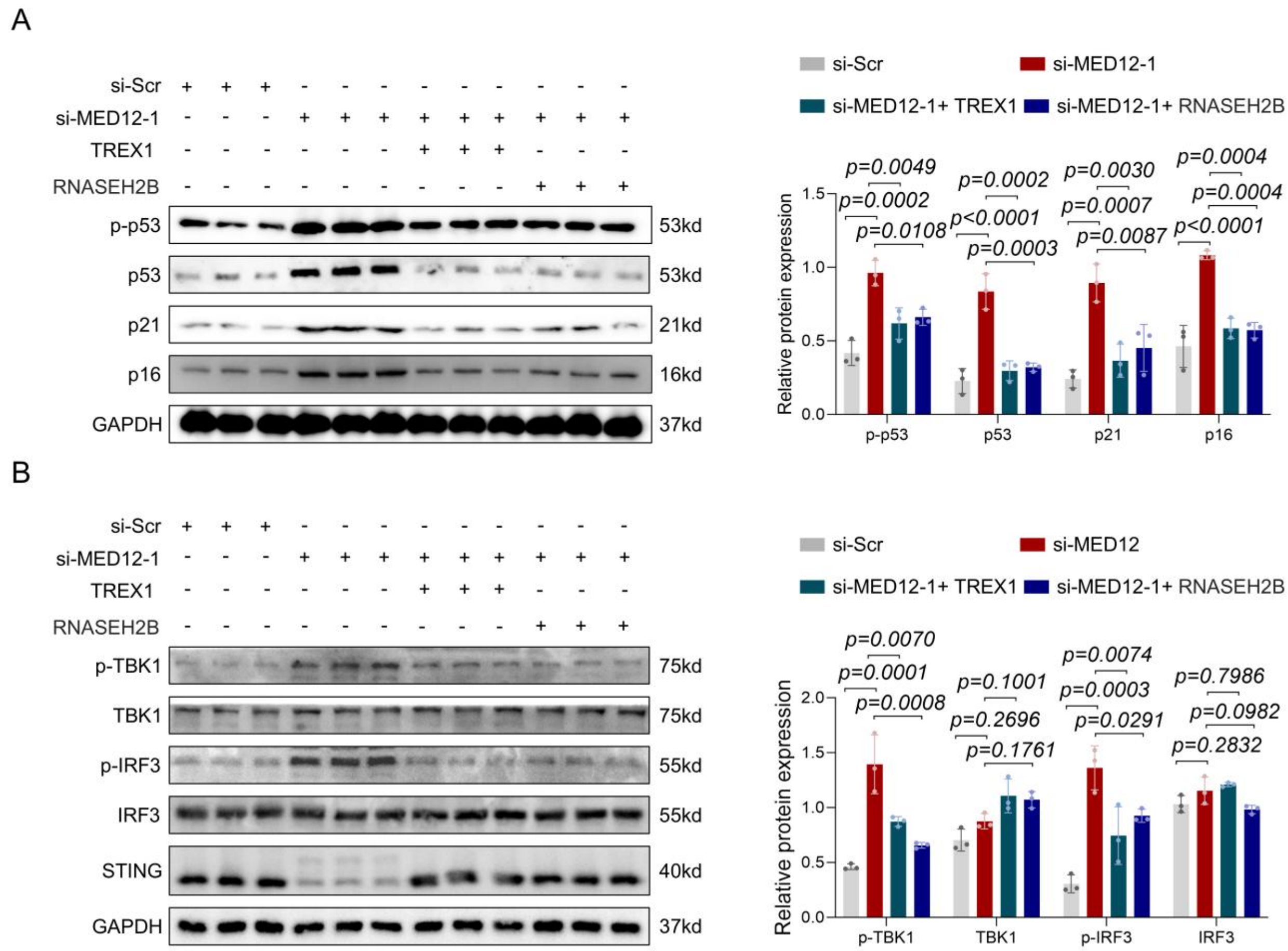

Supplemental figure 8. (A) Protein level analysis of p-p53, p53, p21, p16 in NP cells with the indicated treatment by western blot. **(B)** Protein level analysis of p-TBK1, TBK1, p-IRF3, IRF3, STING in NP cells with the indicated treatment by western blot. One-way ANOVA (**A**, **B**) was used to performed, and data are represented as mean ± SD. At least 3 independent experiments were performed.

Supplemental figure 9.

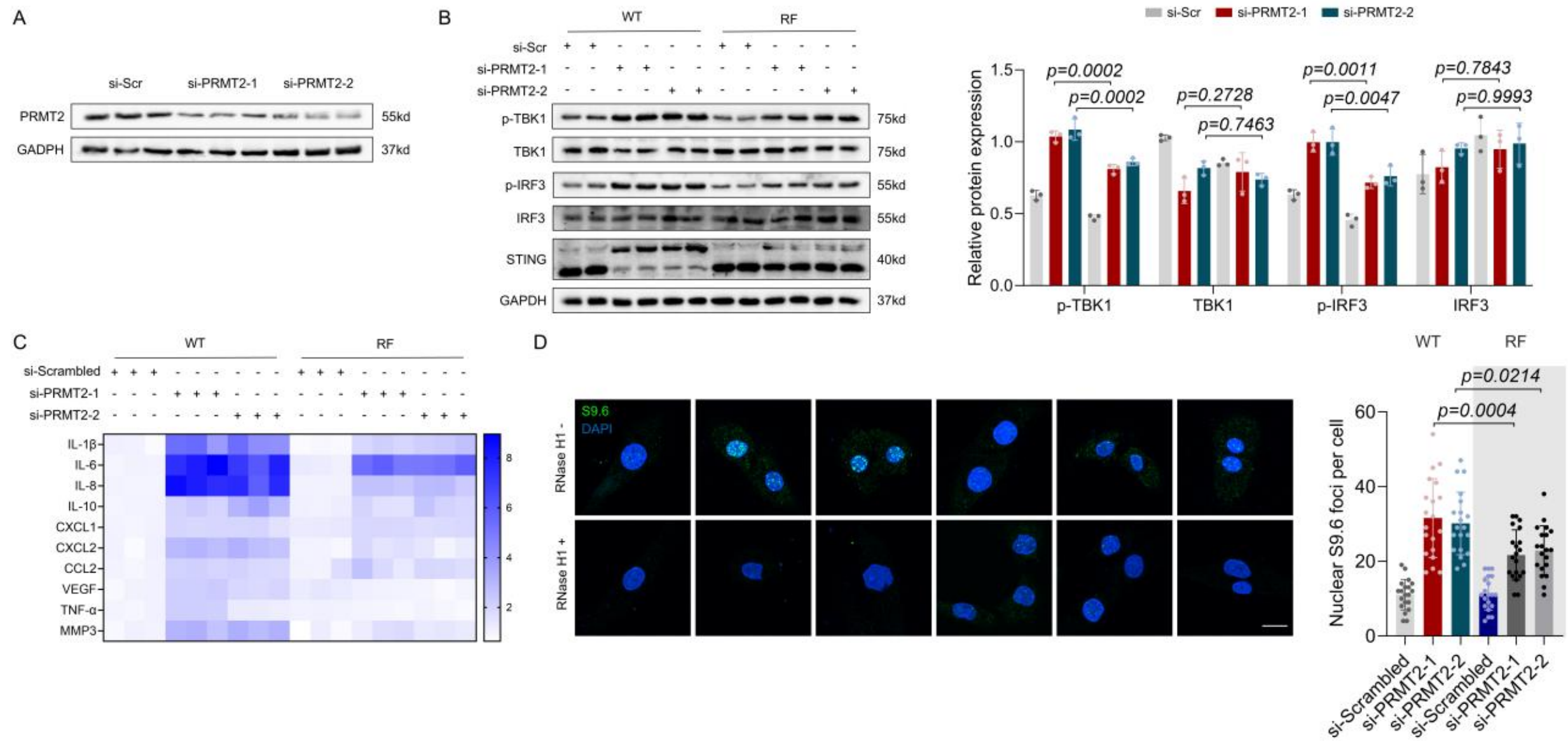

Supplemental figure 9. (**A**) Protein level analysis of PRMT2 in NPCs after treatment with specific siRNAs targeting PRMT2 by western blot. (**B**) Protein level analysis of p-TBK1, TBK1, p-IRF3, IRF3, STING in NP cells with the indicated treatment by western blot. (**C**) Heatmap of differential SASP in NP cells with the indicated treatment. (**D**) IF staining of R-loop in NP cells with the indicated treatment. RNaseH1 treatment was included as a negative control (Scale bar: 10 μm). One-way ANOVA (**B**, **D**) was used to performed, and data are represented as mean ± SD. At least 3 independent experiments were performed.

Supplemental figure 10.

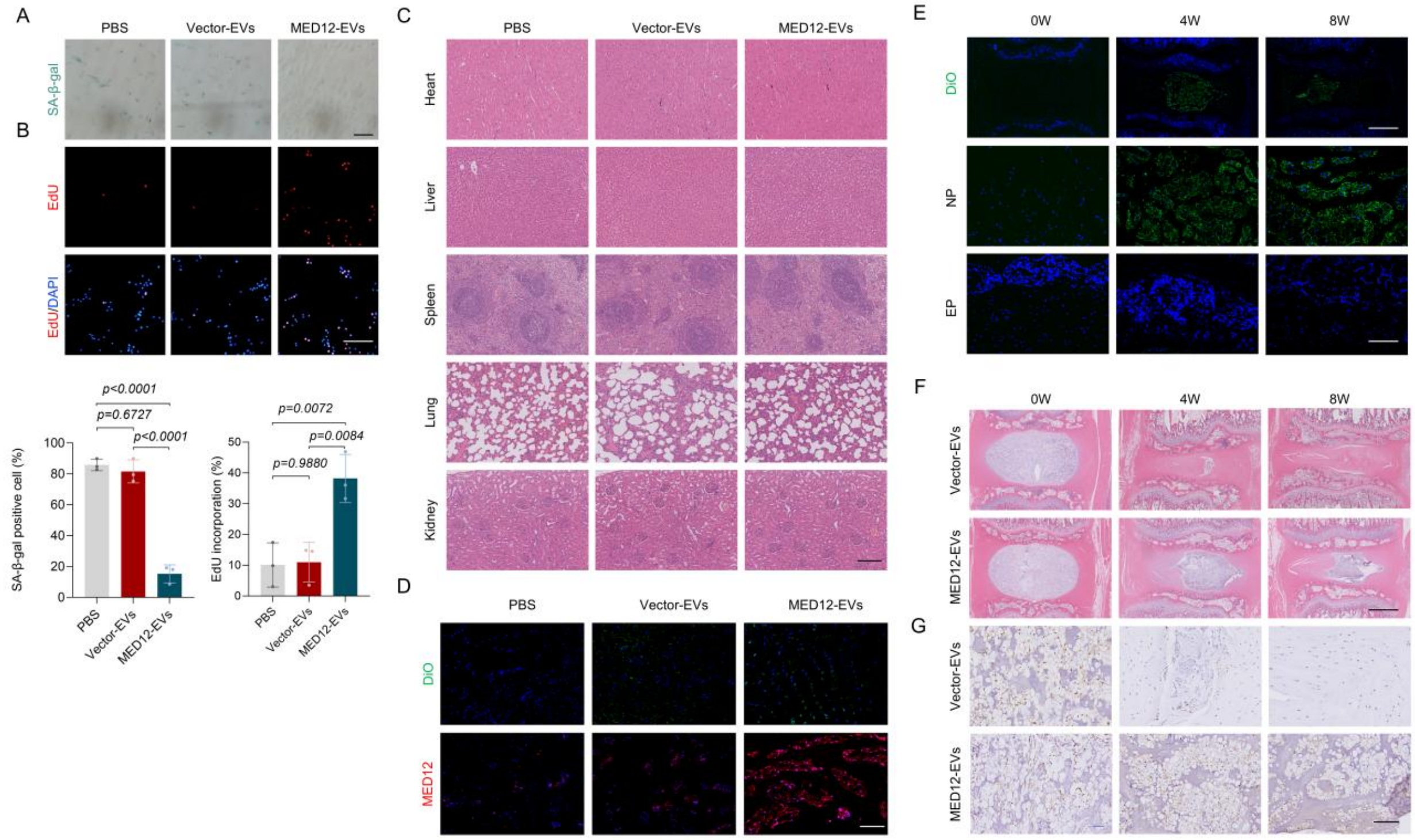

Supplemental figure 10. (**A and B**) SA-β-gal activity staining and EdU incorporation assay of rat NP cells with the indicated treatment (Scale bar: 100 μm). (**C**) H&E staining of lung, liver, spleen, kidney and heart of rats with the indicated treatment (Scale bar: 200 μm). (**D**) IF staining of DiO and MED12 of coccygeal vertebrae from rats with the indicated treatment (Scale bar: 100 μm) (**E**) IF staining of DiO of coccygeal vertebrae from rats with the indicated treatment (1 mm, 250 μm). (**F**) H&E staining of coccygeal vertebrae from rats with the indicated treatment (Scale bar: 1 mm). (**G**) IHC staining of MED12 in coccygeal vertebrae from rats with the indicated treatment (Scale bar: 100 μm). One-way ANOVA (**A**, **B**) was used to performed, and data are represented as mean ± SD. At least 3 independent experiments were performed.

Supplementary Table 1. Characteristics details of the volunteers for label-free quantitative arginine methylated proteomics

Case Number	Gender	Age (years)	Pfirrmann grade	Diagnosis
Case 1	Female	12	I	Idiopathic scolios
Case 2	male	17	I	Idiopathic scolios
Case 3	Female	11	I	Idiopathic scolios
Case 4	male	67	IV	Lumbar disc herniation
Case 5	Female	64	IV	Lumbar disc herniation
Case 6	male	74	IV	Lumbar disc herniation

Supplementary Table 2. Characteristics details of the volunteers for FXBO7-R504me western blotting analysis

Case Number	Gender	Age (years)	Pfirrmann grade	Diagnosis
Case 1	Female	41	II	Lumbar disc herniation
Case 2	male	24	I	Lumbar vertebral fracture
Case 3	Female	31	II	Lumbar disc herniation
Case 4	Female	18	I	Idiopathic scoliosis
Case 5	Female	24	II	Lumbar vertebral fracture
Case 6	male	46	II	Lumbar disc herniation
Case 7	male	41	IV	Lumbar spinal stenosis
Case 8	male	39	IV	Lumbar spinal stenosis
Case 9	Female	32	III	Lumbar disc herniation
Case 10	Female	21	III	Lumbar disc herniation
Case 11	male	47	IV	Lumbar disc herniation
Case 12	male	42	V	Lumbar spondylolisthesis

Supplementary Table 3. Characteristics details of the volunteers for Co-IP analysis

Case Number	Gender	Age (years)	Pfirrmann grade	Diagnosis
Case 1	Female	11	I	Idiopathic scolios
Case 2	male	14	I	Idiopathic scolios
Case 3	Female	11	I	Idiopathic scolios
Case 4	Female	18	I	Idiopathic scolios
Case 5	Female	24	I	Idiopathic scolios
Case 6	male	26	II	Idiopathic scolios
Case 7	male	41	III	Lumbar disc herniation
Case 8	male	49	IV	Lumbar disc herniation
Case 9	Female	52	III	Lumbar disc herniation
Case 10	Female	43	III	Lumbar disc herniation
Case 11	male	47	IV	Lumbar disc herniation
Case 12	male	62	IV	Lumbar disc herniation

Supplementary Table 4. Characteristics details of the volunteers for western blotting analysis

Case Number	Gender	Age (years)	Pfirrmann grade	Diagnosis
Case 1	male	19	I	Idiopathic scolios
Case 2	Female	17	I	Idiopathic scolios
Case 3	Female	11	I	Idiopathic scolios
Case 4	Female	14	I	Idiopathic scolios
Case 5	Female	22	I	Idiopathic scolios
Case 6	Female	21	II	Idiopathic scolios
Case 7	male	51	III	Lumbar disc herniation
Case 8	Female	39	IV	Lumbar disc herniation
Case 9	male	62	III	Lumbar disc herniation
Case 10	Female	78	III	Lumbar disc herniation
Case 11	male	77	IV	Lumbar disc herniation
Case 12	male	52	IV	Lumbar disc herniation

Supplementary Table 5. Classification of Disc Degeneration

Grade	Structure	Distinction of Nucleus and Anulus	Signal Intensity	Height of Intervertebral Disc
I	Homogeneous, bright white	Clear	Hyperintense, isointense to cerebrospinal fluid	Normal
II	Inhomogeneous with or without horizontal bands	Clear	Hyperintense, isointense to cerebrospinal fluid	Normal
III	Inhomogeneous, gray	Unclear	Intermediate	Normal to slightly decreased
IV	Inhomogeneous, gray to black	Lost	Intermediate to hypointense	Normal to moderately decreased
V	Inhomogeneous, black	Lost	Hypointense	Collapsed disc space

Supplementary Table 6. Primer sequences used in Primers used for ChIP

Oligonucleotide	Sense (5'-3')	Antisense (3'-5')
Homo PRMT2	ATTTTGTGGCTCGTAGGGGT	GTGGAGCTCGTCCCCAATC

Supplementary Table 7. Primer sequences used in PCR genotyping

Oligonucleotide	Sense (5'-3')	Antisense (3'-5')
Homo IL-1β	GGTTGAGTTTAAGCCAATCCA	TGCTGACCTAGGCTTGATGA
Homo IL-6	GCCCAGCTATGAACTCCTTCT	GAAGGCAGCAGGCAACAC
Homo IL-8	AGACAGCAGAGCACACAAGC	ATGGTTCCTTCCGGTGGT
Homo IL-10	TGCCTTCAGCAGAGTGAAGA	GCTTGGCAACCCAGGTAA
Homo CXCL1	GCTGAACAGTGACAAATCCAAC	CTTCAGGAACAGCCACCAGT
HomoCXCL2	CCCATGGTTAAGAAAATCATCG	CTTCAGGAACAGCCACCAAT
Homo CCL2	AGTCTCTGCCGCCCTTCT	GTGACTGGGGCATTGATTG
Homo TNF-α	CAGCCTCTTCTCCTTCCTGAT	GCCAGAGGGCTGATTAGAGA
Homo VEGF	AGGGCAGAATCATCACGAAGT	AGGGTCTCGATTGGATGGCA
Homo MMP3	CAAAACATATTTCTTTGTAGAGGACAA	TTCAGCTATTTGCTTGGGAA
Homo GAPDH	CAAGAAGGTGAAGCAGG	TCAAAGGTGGAGGAGTGGGT

Supplementary Table 8. Oligonucleotide sequences used in this study

Sequence name	Forward (5′-3′)	Reverse (5′-3′)
Homo-si-Scrambled	UUCUCCGAACGUGUCACGUTT	ACGUGACACGUUCGGAGAATT
Homo-si-MED12-1	CAGCAUUAUUGCAGAGAAATT	UUUCUCUGCAAUAAUGCUGTT
Homo-si-MED12-2	CCGCAAAGGGACAGCAGAATT	UUCUGCUGUCCCUUUGCGGTT
Homo-si-Scrambled	GGAACUUGGCAACGUGUCUTT	AGACACGUUGCCAAGUUCCTT
Homo-si-PRMT2-1	CAGUUGUGUUGCAGAGAAATT	UUUCUCUGCAACACAACUGTT
Homo-si-PRMT2-2	GUGUCAUCCUGCAGAAUAATT	UUAUUCUGCAGGAUGACACTT
Homo-si-Scrambled	GCAAUAGGUGCAGAUAGAGTT	CUCUAUCUGCACCUAUUGCTT
Homo-si-FBXO7	GAAUGACGACAGUAUGUUATT	UAACAUACUGUCGUCAUUCTT
Homo-si-Scrambled	UUCUCCGAACGUGUCACGUTT	ACGUGACACGUUCGGAGAATT
Homo-si-STING	GCAUUACAACAACCUGCUATT	UAGCAGGUUGUUGUAAUGCTT

Supplementary Table 9. Sequences of the shRNAs targeting rat mRNA transcripts

Target Gene	Clone name name	Sequence
Scrambled	Rat-sh-Scrambled	5′-GACGGAATATGCGACTATGGT-3′
PRMT2	Rat-sh-PRMT2-1	5′-CGGTGGTGTTACAGAGAAATC-3′
	Rat-sh-PRMT2-2	5′-TAGCAATCAAGGAGTTCTTTTCA-3′
Scrambled	Rat-sh-Scrambled	5′-GAGCACTAGCGCTGTTAATTA-3′
MED12	Rat-sh-MED12-1	5′-GCAAGATCTCAGAGTGCTATT-3′
	Rat-sh-MED12-2	5′-CGACTGTTGCTCTACCATACC-3′
Scrambled	Rat-sh-Scrambled	5′-GAGCACTAGCGCTGTTAATTA-3′
FBXO7	Rat-sh-FBXO7	5′-CATTCCTCTCTCCAGAATAAT-3′
Scrambled	Rat-sh-Scrambled	5′-GTCTCCGAACGTGTCACGT-3′
HIF1A	Rat-sh-HIF1A	5′-GCAGACCCAGTTACAGAAACC-3′

Supplementary Table 10. Antibody information

Antibody	Company	Catalog#	Application/ Dilution
anti-p16INK4a	Affinity	AF5484	WB (1: 500)
anti-p21	CST	2947	WB (1: 500)
anti-Phospho-p53 (Ser15)	Abcam	ab278683	WB (1: 1000); IHC (1:150)
anti-p53	CST	9282	WB (1: 1000)
anti-Phospho-γH2A.X (S139)	Abcam	ab81299	IHC (1:150); WB (1:500); IF (1:250)
anti-STING	Proteintech	19851-1-AP	WB (1:1000)
anti-ubiquitin	Abcam	ab134953	WB (1: 500)
anti-mono-Methyl Arginine	CST	8711	WB (1:1000)
FBXO7	CST	57981	WB (1: 1000); IP (4 μg/1000 μg)
PRMT2	Proteintech	66885-1-Ig	WB (1: 1000); IP (4 μg/1000 μg)
anti-GAPDH	Proteintech	60004-1-Ig	WB (1: 2000)
anti-β-actin	Proteintech	66009-1-Ig	WB (1: 2000)
anti-MED12	Proteintech	20028-1-AP	WB (1: 1000); IP (4 μg/1000 μg)
anti-S9.6	Sigma-Aldrich	MABE1095	Dot blot (1: 200); IF: (1: 100)
anti-ssDNA	Sigma-Aldrich	ZMS1042	IF (1:100)
anti-CD63	Proteintech	25682-1-AP	WB (1: 500)
anti-CD9	Proteintech	20597-1-AP	WB (1: 2000)
anti-TSG101	Proteintech	28283-1-AP	WB (1: 1000)
anti-Flag tag epitope	Proteintech	66008-4-Ig	WB (1: 20000); IP (4 μg/1000 μg)
anti-HA tag	Proteintech	51064-2-AP	WB (1: 5000); IP (4 μg/1000 μg)
anti-GFP tag	Proteintech	50430-2-AP	WB (1: 1000); IP (4 μg/1000 μg)
anti- NUMA1	Proteintech	16607-1-AP	WB (1: 500)
anti-THRAP3	Proteintech	19744-1-AP	WB (1: 500)
anti-Histone H1.2	Proteintech	19649-1-AP	WB (1: 500)
anti-TRA2B	Proteintech	29627-1-AP	WB (1: 1000)
anti-SEC23A	Proteintech	30465-1-AP	WB (1: 500)
anti-RPL29	Proteintech	15799-1-AP	WB (1: 2000)
anti-TBK1	Proteintech	67211-1-Ig	WB (1: 500)
anti-Phospho-TBK1(Ser172)	CST	5483T	WB (1: 1000)
anti-FBXO7-R504me	HUABIO	N/A	WB (1: 1000)
anti-IRF3	HUABIO	ET1612-14	WB (1: 2000)
anti-Phospho-IRF3 (S396)	HUABIO	HA722772	WB (1: 1000)
anti-HIF1a	Proteintech	20960-1-AP	WB (1: 1000); IP (4 μg/1000 μg)

Supplementary Table 11. FBXO7 interactome in RK-mutant expressing cells.

Accession	GeneName	Description

A0A075B6R9	IGKV2D-24	Probable non-functional immunoglobulin kappa variable 2D-24 OS=Homo sapiens OX=9606 GN=IGKV2D-24 PE=1 SV=1
A0A087WW87	IGKV2-40	Immunoglobulin kappa variable 2-40 OS=Homo sapiens OX=9606 GN=IGKV2-40 PE=3 SV=2
A7MCY6	TBKBP1	TANK-binding kinase 1-binding protein 1 OS=Homo sapiens OX=9606 GN=TBKBP1 PE=1 SV=1
A8MTJ3	GNAT3	Guanine nucleotide-binding protein G(t) subunit alpha-3 OS=Homo sapiens OX=9606 GN=GNAT3 PE=2 SV=2
O00423	EML1	Echinoderm microtubule-associated protein-like 1 OS=Homo sapiens OX=9606 GN=EML1 PE=1 SV=3
O00567	NOP56	Nucleolar protein 56 OS=Homo sapiens OX=9606 GN=NOP56 PE=1 SV=4
O14654	IRS4	Insulin receptor substrate 4 OS=Homo sapiens OX=9606 GN=IRS4 PE=1 SV=1
O14732	IMPA2	Inositol monophosphatase 2 OS=Homo sapiens OX=9606 GN=IMPA2 PE=1 SV=1
O14950	MYL12B	Myosin regulatory light chain 12B OS=Homo sapiens OX=9606 GN=MYL12B PE=1 SV=2
O15144	ARPC2	Actin-related protein 2/3 complex subunit 2 OS=Homo sapiens OX=9606 GN=ARPC2 PE=1 SV=1
O15371	EIF3D	Eukaryotic translation initiation factor 3 subunit D OS=Homo sapiens OX=9606 GN=EIF3D PE=1 SV=1
O43795	MYO1B	Unconventional myosin-Ib OS=Homo sapiens OX=9606 GN=MYO1B PE=1 SV=3
O60244	MED14	Mediator of RNA polymerase II transcription subunit 14 OS=Homo sapiens OX=9606 GN=MED14 PE=1 SV=2
O75592	MYCBP2	E3 ubiquitin-protein ligase MYCBP2 OS=Homo sapiens OX=9606 GN=MYCBP2 PE=1 SV=4
O76015	KRT38	Keratin, type I cuticular Ha8 OS=Homo sapiens OX=9606 GN=KRT38 PE=1 SV=3
O94826	TOMM70	Mitochondrial import receptor subunit TOM70 OS=Homo sapiens OX=9606 GN=TOMM70 PE=1 SV=1
O94905	ERLIN2	Erlin-2 OS=Homo sapiens OX=9606 GN=ERLIN2 PE=1 SV=1
O95071	UBR5	E3 ubiquitin-protein ligase UBR5 OS=Homo sapiens OX=9606 GN=UBR5 PE=1 SV=2
O95163	ELP1	Elongator complex protein 1 OS=Homo sapiens OX=9606 GN=ELP1 PE=1 SV=3
O95714	HERC2	E3 ubiquitin-protein ligase HERC2 OS=Homo sapiens OX=9606 GN=HERC2 PE=1 SV=2
O95835	LATS1	Serine/threonine-protein kinase LATS1 OS=Homo sapiens OX=9606 GN=LATS1 PE=1 SV=1
P00441	SOD1	Superoxide dismutase [Cu-Zn] OS=Homo sapiens OX=9606 GN=SOD1 PE=1 SV=2
P00505	GOT2	Aspartate aminotransferase, mitochondrial OS=Homo sapiens OX=9606 GN=GOT2 PE=1 SV=3
P00558	PGK1	Phosphoglycerate kinase 1 OS=Homo sapiens OX=9606 GN=PGK1 PE=1 SV=3
P01860	IGHG3	Immunoglobulin heavy constant gamma 3 OS=Homo sapiens OX=9606 GN=IGHG3 PE=1 SV=2

P02461	COL3A1	Collagen alpha-1(III) chain OS=Homo sapiens OX=9606 GN=COL3A1 PE=1 SV=4
P02656	APOC3	Apolipoprotein C-III OS=Homo sapiens OX=9606 GN=APOC3 PE=1 SV=1
P02787	TF	Serotransferrin OS=Homo sapiens OX=9606 GN=TF PE=1 SV=3
P04279	SEMG1	Semenogelin-1 OS=Homo sapiens OX=9606 GN=SEMG1 PE=1 SV=2
P05141	SLC25A5	ADP/ATP translocase 2 OS=Homo sapiens OX=9606 GN=SLC25A5 PE=1 SV=7
P05164	MPO	Myeloperoxidase OS=Homo sapiens OX=9606 GN=MPO PE=1 SV=1
P05388	RPLP0	60S acidic ribosomal protein P0 OS=Homo sapiens OX=9606 GN=RPLP0 PE=1 SV=1
P06454	PTMA	Prothymosin alpha OS=Homo sapiens OX=9606 GN=PTMA PE=1 SV=2
P06703	S100A6	Protein S100-A6 OS=Homo sapiens OX=9606 GN=S100A6 PE=1 SV=1
P06744	GPI	Glucose-6-phosphate isomerase OS=Homo sapiens OX=9606 GN=GPI PE=1 SV=4
P06748	NPM1	Nucleophosmin OS=Homo sapiens OX=9606 GN=NPM1 PE=1 SV=2
P07305	H1-0	Histone H1.0 OS=Homo sapiens OX=9606 GN=H1-0 PE=1 SV=3
P07477	PRSS1	Serine protease 1 OS=Homo sapiens OX=9606 GN=PRSS1 PE=1 SV=1
P07910	HNRNPC	Heterogeneous nuclear ribonucleoproteins C1/C2 OS=Homo sapiens OX=9606 GN=HNRNPC PE=1 SV=4
P08246	ELANE	Neutrophil elastase OS=Homo sapiens OX=9606 GN=ELANE PE=1 SV=1
P08311	CTSG	Cathepsin G OS=Homo sapiens OX=9606 GN=CTSG PE=1 SV=2
P08758	ANXA5	Annexin A5 OS=Homo sapiens OX=9606 GN=ANXA5 PE=1 SV=2
P09211	GSTP1	Glutathione S-transferase P OS=Homo sapiens OX=9606 GN=GSTP1 PE=1 SV=2
P09622	DLD	Dihydrolipoyl dehydrogenase, mitochondrial OS=Homo sapiens OX=9606 GN=DLD PE=1 SV=2
P0DP04	IGHV3-43D	Immunoglobulin heavy variable 3-43D OS=Homo sapiens OX=9606 GN=IGHV3-43D PE=3 SV=1
P11387	TOP1	DNA topoisomerase 1 OS=Homo sapiens OX=9606 GN=TOP1 PE=1 SV=2
P11940	PABPC1	Polyadenylate-binding protein 1 OS=Homo sapiens OX=9606 GN=PABPC1 PE=1 SV=2
P12109	COL6A1	Collagen alpha-1(VI) chain OS=Homo sapiens OX=9606 GN=COL6A1 PE=1 SV=3
P12111	COL6A3	Collagen alpha-3(VI) chain OS=Homo sapiens OX=9606 GN=COL6A3 PE=1 SV=5
P12814	ACTN1	Alpha-actinin-1 OS=Homo sapiens OX=9606 GN=ACTN1 PE=1 SV=2

P16403	H1-2	Histone H1.2 OS=Homo sapiens OX=9606 GN=H1-2 PE=1 SV=2
P16615	ATP2A2	Sarcoplasmic/endoplasmic reticulum calcium ATPase 2 OS=Homo sapiens OX=9606 GN=ATP2A2 PE=1 SV=1
P17844	DDX5	Probable ATP-dependent RNA helicase DDX5 OS=Homo sapiens OX=9606 GN=DDX5 PE=1 SV=1
P18077	RPL35A	60S ribosomal protein L35a OS=Homo sapiens OX=9606 GN=RPL35A PE=1 SV=2
P18583	SON	Protein SON OS=Homo sapiens OX=9606 GN=SON PE=1 SV=4
P18621	RPL17	60S ribosomal protein L17 OS=Homo sapiens OX=9606 GN=RPL17 PE=1 SV=3
P19075	TSPAN8	Tetraspanin-8 OS=Homo sapiens OX=9606 GN=TSPAN8 PE=1 SV=1
P21796	VDAC1	Voltage-dependent anion-selective channel protein 1 OS=Homo sapiens OX=9606 GN=VDAC1 PE=1 SV=2
P22061	PCMT1	Protein-L-isoaspartate(D-aspartate) O-methyltransferase OS=Homo sapiens OX=9606 GN=PCMT1 PE=1 SV=4
P22532	SPRR2D	Small proline-rich protein 2D OS=Homo sapiens OX=9606 GN=SPRR2D PE=2 SV=2
P23435	CBLN1	Cerebellin-1 OS=Homo sapiens OX=9606 GN=CBLN1 PE=1 SV=1
P24278	ZBTB25	Zinc finger and BTB domain-containing protein 25 OS=Homo sapiens OX=9606 GN=ZBTB25 PE=1 SV=2
P25786	PSMA1	Proteasome subunit alpha type-1 OS=Homo sapiens OX=9606 GN=PSMA1 PE=1 SV=1
P25788	PSMA3	Proteasome subunit alpha type-3 OS=Homo sapiens OX=9606 GN=PSMA3 PE=1 SV=2
P27105	STOM	Stomatin OS=Homo sapiens OX=9606 GN=STOM PE=1 SV=3
P30050	RPL12	60S ribosomal protein L12 OS=Homo sapiens OX=9606 GN=RPL12 PE=1 SV=1
P31930	UQCRC1	Cytochrome b-c1 complex subunit 1, mitochondrial OS=Homo sapiens OX=9606 GN=UQCRC1 PE=1 SV=3
P35268	RPL22	60S ribosomal protein L22 OS=Homo sapiens OX=9606 GN=RPL22 PE=1 SV=2
P35914	HMGCL	Hydroxymethylglutaryl-CoA lyase, mitochondrial OS=Homo sapiens OX=9606 GN=HMGCL PE=1 SV=2
P42704	LRPPRC	Leucine-rich PPR motif-containing protein, mitochondrial OS=Homo sapiens OX=9606 GN=LRPPRC PE=1 SV=3
P46977	STT3A	Dolichyl-diphosphooligosaccharide--protein glycosyltransferase subunit STT3A OS=Homo sapiens OX=9606 GN=STT3A PE=1 SV=2
P47755	CAPZA2	F-actin-capping protein subunit alpha-2 OS=Homo sapiens OX=9606 GN=CAPZA2 PE=1 SV=3
P47756	CAPZB	F-actin-capping protein subunit beta OS=Homo sapiens OX=9606 GN=CAPZB PE=1 SV=5
P47914	RPL29	60S ribosomal protein L29 OS=Homo sapiens OX=9606 GN=RPL29 PE=1 SV=2
P48047	ATP5PO	ATP synthase subunit O, mitochondrial OS=Homo sapiens OX=9606 GN=ATP5PO PE=1 SV=1

P48594	SERPINB4	Serpin B4 OS=Homo sapiens OX=9606 GN=SERPINB4 PE=1 SV=2
P49368	CCT3	T-complex protein 1 subunit gamma OS=Homo sapiens OX=9606 GN=CCT3 PE=1 SV=4
P49411	TUFM	Elongation factor Tu, mitochondrial OS=Homo sapiens OX=9606 GN=TUFM PE=1 SV=2
P49750	YLPM1	YLP motif-containing protein 1 OS=Homo sapiens OX=9606 GN=YLPM1 PE=1 SV=4
P50402	EMD	Emerin OS=Homo sapiens OX=9606 GN=EMD PE=1 SV=1
P50991	CCT4	T-complex protein 1 subunit delta OS=Homo sapiens OX=9606 GN=CCT4 PE=1 SV=4
P51114	FXR1	RNA-binding protein FXR1 OS=Homo sapiens OX=9606 GN=FXR1 PE=1 SV=3
P51148	RAB5C	Ras-related protein Rab-5C OS=Homo sapiens OX=9606 GN=RAB5C PE=1 SV=2
P51532	SMARCA4	Transcription activator BRG1 OS=Homo sapiens OX=9606 GN=SMARCA4 PE=1 SV=2
P51572	BCAP31	B-cell receptor-associated protein 31 OS=Homo sapiens OX=9606 GN=BCAP31 PE=1 SV=3
P51991	HNRNPA3	Heterogeneous nuclear ribonucleoprotein A3 OS=Homo sapiens OX=9606 GN=HNRNPA3 PE=1 SV=2
P52907	CAPZA1	F-actin-capping protein subunit alpha-1 OS=Homo sapiens OX=9606 GN=CAPZA1 PE=1 SV=3
P55060	CSE1L	Exportin-2 OS=Homo sapiens OX=9606 GN=CSE1L PE=1 SV=3
P55735	SEC13	Protein SEC13 homolog OS=Homo sapiens OX=9606 GN=SEC13 PE=1 SV=3
P61158	ACTR3	Actin-related protein 3 OS=Homo sapiens OX=9606 GN=ACTR3 PE=1 SV=3
P61160	ACTR2	Actin-related protein 2 OS=Homo sapiens OX=9606 GN=ACTR2 PE=1 SV=1
P62244	RPS15A	40S ribosomal protein S15a OS=Homo sapiens OX=9606 GN=RPS15A PE=1 SV=2
P62266	RPS23	40S ribosomal protein S23 OS=Homo sapiens OX=9606 GN=RPS23 PE=1 SV=3
P62277	RPS13	40S ribosomal protein S13 OS=Homo sapiens OX=9606 GN=RPS13 PE=1 SV=2
P62424	RPL7A	60S ribosomal protein L7a OS=Homo sapiens OX=9606 GN=RPL7A PE=1 SV=2
P62701	RPS4X	40S ribosomal protein S4, X isoform OS=Homo sapiens OX=9606 GN=RPS4X PE=1 SV=2
P62750	RPL23A	60S ribosomal protein L23a OS=Homo sapiens OX=9606 GN=RPL23A PE=1 SV=1
P62753	RPS6	40S ribosomal protein S6 OS=Homo sapiens OX=9606 GN=RPS6 PE=1 SV=1
P62847	RPS24	40S ribosomal protein S24 OS=Homo sapiens OX=9606 GN=RPS24 PE=1 SV=1
P62861	FAU	FAU ubiquitin-like and ribosomal protein S30 OS=Homo sapiens OX=9606 GN=FAU PE=1 SV=2

P62873	GNB1	Guanine nucleotide-binding protein G(I)/G(S)/G(T) subunit beta-1 OS=Homo sapiens OX=9606 GN=GNB1 PE=1 SV=3
P62917	RPL8	60S ribosomal protein L8 OS=Homo sapiens OX=9606 GN=RPL8 PE=1 SV=2
P62995	TRA2B	Transformer-2 protein homolog beta OS=Homo sapiens OX=9606 GN=TRA2B PE=1 SV=1
P63104	YWHAZ	14-3-3 protein zeta/delta OS=Homo sapiens OX=9606 GN=YWHAZ PE=1 SV=1
P63244	RACK1	Receptor of activated protein C kinase 1 OS=Homo sapiens OX=9606 GN=RACK1 PE=1 SV=3
P67809	YBX1	Y-box-binding protein 1 OS=Homo sapiens OX=9606 GN=YBX1 PE=1 SV=3
P78371	CCT2	T-complex protein 1 subunit beta OS=Homo sapiens OX=9606 GN=CCT2 PE=1 SV=4
P82094	TMF1	TATA element modulatory factor OS=Homo sapiens OX=9606 GN=TMF1 PE=1 SV=2
P83881	RPL36A	60S ribosomal protein L36a OS=Homo sapiens OX=9606 GN=RPL36A PE=1 SV=2
Q00610	CLTC	Clathrin heavy chain 1 OS=Homo sapiens OX=9606 GN=CLTC PE=1 SV=5
Q01081	U2AF1	Splicing factor U2AF 35 kDa subunit OS=Homo sapiens OX=9606 GN=U2AF1 PE=1 SV=3
Q02383	SEMG2	Semenogelin-2 OS=Homo sapiens OX=9606 GN=SEMG2 PE=1 SV=1
Q04637	EIF4G1	Eukaryotic translation initiation factor 4 gamma 1 OS=Homo sapiens OX=9606 GN=EIF4G1 PE=1 SV=4
Q05639	EEF1A2	Elongation factor 1-alpha 2 OS=Homo sapiens OX=9606 GN=EEF1A2 PE=1 SV=1
Q07666	KHDRBS1	KH domain-containing, RNA-binding, signal transduction-associated protein 1 OS=Homo sapiens OX=9606 GN=KHDRBS1 PE=1 SV=1
Q08379	GOLGA2	Golgin subfamily A member 2 OS=Homo sapiens OX=9606 GN=GOLGA2 PE=1 SV=3
Q10570	CPSF1	Cleavage and polyadenylation specificity factor subunit 1 OS=Homo sapiens OX=9606 GN=CPSF1 PE=1 SV=2
Q13085	ACACA	Acetyl-CoA carboxylase 1 OS=Homo sapiens OX=9606 GN=ACACA PE=1 SV=2
Q13185	CBX3	Chromobox protein homolog 3 OS=Homo sapiens OX=9606 GN=CBX3 PE=1 SV=4
Q13310	PABPC4	Polyadenylate-binding protein 4 OS=Homo sapiens OX=9606 GN=PABPC4 PE=1 SV=1
Q13510	ASAH1	Acid ceramidase OS=Homo sapiens OX=9606 GN=ASAH1 PE=1 SV=5
Q13885	TUBB2A	Tubulin beta-2A chain OS=Homo sapiens OX=9606 GN=TUBB2A PE=1 SV=1
Q14498	RBM39	RNA-binding protein 39 OS=Homo sapiens OX=9606 GN=RBM39 PE=1 SV=2
Q14533	KRT81	Keratin, type II cuticular Hb1 OS=Homo sapiens OX=9606 GN=KRT81 PE=1 SV=3

Q14686	NCOA6	Nuclear receptor coactivator 6 OS=Homo sapiens OX=9606 GN=NCOA6 PE=1 SV=3
Q14974	KPNB1	Importin subunit beta-1 OS=Homo sapiens OX=9606 GN=KPNB1 PE=1 SV=2
Q14980	NUMA1	Nuclear mitotic apparatus protein 1 OS=Homo sapiens OX=9606 GN=NUMA1 PE=1 SV=2
Q15029	EFTUD2	116 kDa U5 small nuclear ribonucleoprotein component OS=Homo sapiens OX=9606 GN=EFTUD2 PE=1 SV=1
Q15233	NONO	Non-POU domain-containing octamer-binding protein OS=Homo sapiens OX=9606 GN=NONO PE=1 SV=4
Q15424	SAFB	Scaffold attachment factor B1 OS=Homo sapiens OX=9606 GN=SAFB PE=1 SV=4
Q15436	SEC23A	Protein transport protein Sec23A OS=Homo sapiens OX=9606 GN=SEC23A PE=1 SV=2
Q15582	TGFBI	Transforming growth factor-beta-induced protein ig-h3 OS=Homo sapiens OX=9606 GN=TGFBI PE=1 SV=1
Q15637	SF1	Splicing factor 1 OS=Homo sapiens OX=9606 GN=SF1 PE=1 SV=4
Q15911	ZFHX3	Zinc finger homeobox protein 3 OS=Homo sapiens OX=9606 GN=ZFHX3 PE=1 SV=2
Q16658	FSCN1	Fascin OS=Homo sapiens OX=9606 GN=FSCN1 PE=1 SV=3
Q16778	H2BC21	Histone H2B type 2-E OS=Homo sapiens OX=9606 GN=H2BC21 PE=1 SV=3
Q5T481	RBM20	RNA-binding protein 20 OS=Homo sapiens OX=9606 GN=RBM20 PE=1 SV=4
Q5T4S7	UBR4	E3 ubiquitin-protein ligase UBR4 OS=Homo sapiens OX=9606 GN=UBR4 PE=1 SV=1
Q63HN8	RNF213	E3 ubiquitin-protein ligase RNF213 OS=Homo sapiens OX=9606 GN=RNF213 PE=1 SV=3
Q6E0U4	DMKN	Dermokine OS=Homo sapiens OX=9606 GN=DMKN PE=1 SV=3
Q6GMR7	FAAH2	Fatty-acid amide hydrolase 2 OS=Homo sapiens OX=9606 GN=FAAH2 PE=1 SV=1
Q6NZI2	CAVIN1	Caveolae-associated protein 1 OS=Homo sapiens OX=9606 GN=CAVIN1 PE=1 SV=1
Q6ZVX7	NCCRP1	F-box only protein 50 OS=Homo sapiens OX=9606 GN=NCCRP1 PE=1 SV=1
Q7Z3Y8	KRT27	Keratin, type I cytoskeletal 27 OS=Homo sapiens OX=9606 GN=KRT27 PE=1 SV=2
Q7Z417	NUFIP2	FMR1-interacting protein NUFIP2 OS=Homo sapiens OX=9606 GN=NUFIP2 PE=1 SV=1
Q7Z6Z7	HUWE1	E3 ubiquitin-protein ligase HUWE1 OS=Homo sapiens OX=9606 GN=HUWE1 PE=1 SV=3
Q86T82	USP37	Ubiquitin carboxyl-terminal hydrolase 37 OS=Homo sapiens OX=9606 GN=USP37 PE=1 SV=2
Q8IUD2	ERC1	ELKS/Rab6-interacting/CAST family member 1 OS=Homo sapiens OX=9606 GN=ERC1 PE=1 SV=1
Q8IW75	SERPINA12	Serpin A12 OS=Homo sapiens OX=9606 GN=SERPINA12 PE=1 SV=1

Q8IWA0	WDR75	WD repeat-containing protein 75 OS=Homo sapiens OX=9606 GN=WDR75 PE=1 SV=1
Q8IYB3	SRRM1	Serine/arginine repetitive matrix protein 1 OS=Homo sapiens OX=9606 GN=SRRM1 PE=1 SV=2
Q8N3X1	FNBP4	Formin-binding protein 4 OS=Homo sapiens OX=9606 GN=FNBP4 PE=1 SV=3
Q8NC51	SERBP1	Plasminogen activator inhibitor 1 RNA-binding protein OS=Homo sapiens OX=9606 GN=SERBP1 PE=1 SV=2
Q8NCA5	FAM98A	Protein FAM98A OS=Homo sapiens OX=9606 GN=FAM98A PE=1 SV=2
Q8ND56	LSM14A	Protein LSM14 homolog A OS=Homo sapiens OX=9606 GN=LSM14A PE=1 SV=3
Q8NEX9	SDR9C7	Short-chain dehydrogenase/reductase family 9C member 7 OS=Homo sapiens OX=9606 GN=SDR9C7 PE=1 SV=1
Q8NEZ4	KMT2C	Histone-lysine N-methyltransferase 2C OS=Homo sapiens OX=9606 GN=KMT2C PE=1 SV=3
Q8NI35	PATJ	InaD-like protein OS=Homo sapiens OX=9606 GN=PATJ PE=1 SV=3
Q8WWM7	ATXN2L	Ataxin-2-like protein OS=Homo sapiens OX=9606 GN=ATXN2L PE=1 SV=2
Q8WXX0	DNAH7	Dynein axonemal heavy chain 7 OS=Homo sapiens OX=9606 GN=DNAH7 PE=1 SV=2
Q92616	GCN1	eIF-2-alpha kinase activator GCN1 OS=Homo sapiens OX=9606 GN=GCN1 PE=1 SV=7
Q92794	KAT6A	Histone acetyltransferase KAT6A OS=Homo sapiens OX=9606 GN=KAT6A PE=1 SV=2
Q92900	UPF1	Regulator of nonsense transcripts 1 OS=Homo sapiens OX=9606 GN=UPF1 PE=1 SV=2
Q93074	MED12	Mediator of RNA polymerase II transcription subunit 12 OS=Homo sapiens OX=9606 GN=MED12 PE=1 SV=4
Q96CW1	AP2M1	AP-2 complex subunit mu OS=Homo sapiens OX=9606 GN=AP2M1 PE=1 SV=2
Q96EK7	FAM120B	Constitutive coactivator of peroxisome proliferator-activated receptor gamma OS=Homo sapiens OX=9606 GN=FAM120B PE=1 SV=1
Q96IZ0	PAWR	PRKC apoptosis WT1 regulator protein OS=Homo sapiens OX=9606 GN=PAWR PE=1 SV=1
Q96JH8	RADIL	Ras-associating and dilute domain-containing protein OS=Homo sapiens OX=9606 GN=RADIL PE=1 SV=5
Q96N67	DOCK7	Dedicator of cytokinesis protein 7 OS=Homo sapiens OX=9606 GN=DOCK7 PE=1 SV=4
Q99578	RIT2	GTP-binding protein Rit2 OS=Homo sapiens OX=9606 GN=RIT2 PE=1 SV=1
Q99848	EBNA1BP2	Probable rRNA-processing protein EBP2 OS=Homo sapiens OX=9606 GN=EBNA1BP2 PE=1 SV=2
Q9BQG0	MYBBP1A	Myb-binding protein 1A OS=Homo sapiens OX=9606 GN=MYBBP1A PE=1 SV=2
Q9BRL6	SRSF8	Serine/arginine-rich splicing factor 8 OS=Homo sapiens OX=9606 GN=SRSF8 PE=1 SV=1

Q9C0H5	ARHGAP39	Rho GTPase-activating protein 39 OS=Homo sapiens OX=9606 GN=ARHGAP39 PE=1 SV=2
Q9H0U3	MAGT1	Magnesium transporter protein 1 OS=Homo sapiens OX=9606 GN=MAGT1 PE=1 SV=1
Q9H1E1	RNASE7	Ribonuclease 7 OS=Homo sapiens OX=9606 GN=RNASE7 PE=1 SV=2
Q9H307	PNN	Pinin OS=Homo sapiens OX=9606 GN=PNN PE=1 SV=5
Q9H583	HEATR1	HEAT repeat-containing protein 1 OS=Homo sapiens OX=9606 GN=HEATR1 PE=1 SV=3
Q9H6D7	HAUS4	HAUS augmin-like complex subunit 4 OS=Homo sapiens OX=9606 GN=HAUS4 PE=1 SV=1
Q9HC35	EML4	Echinoderm microtubule-associated protein-like 4 OS=Homo sapiens OX=9606 GN=EML4 PE=1 SV=3
Q9HCE1	MOV10	Helicase MOV-10 OS=Homo sapiens OX=9606 GN=MOV10 PE=1 SV=2
Q9NQ29	LUC7L	Putative RNA-binding protein Luc7-like 1 OS=Homo sapiens OX=9606 GN=LUC7L PE=1 SV=1
Q9NR30	DDX21	Nucleolar RNA helicase 2 OS=Homo sapiens OX=9606 GN=DDX21 PE=1 SV=5
Q9NTK1	DEPP1	Protein DEPP1 OS=Homo sapiens OX=9606 GN=DEPP1 PE=1 SV=2
Q9NU22	MDN1	Midasin OS=Homo sapiens OX=9606 GN=MDN1 PE=1 SV=2
Q9NUG4	CCM2L	Cerebral cavernous malformations 2 protein-like OS=Homo sapiens OX=9606 GN=CCM2L PE=1 SV=3
Q9NZ01	TECR	Very-long-chain enoyl-CoA reductase OS=Homo sapiens OX=9606 GN=TECR PE=1 SV=1
Q9NZB2	FAM120A	Constitutive coactivator of PPAR-gamma-like protein 1 OS=Homo sapiens OX=9606 GN=FAM120A PE=1 SV=2
Q9NZD8	SPG21	Maspardin OS=Homo sapiens OX=9606 GN=SPG21 PE=1 SV=1
Q9NZI8	IGF2BP1	Insulin-like growth factor 2 mRNA-binding protein 1 OS=Homo sapiens OX=9606 GN=IGF2BP1 PE=1 SV=2
Q9P219	CCDC88C	Protein Daple OS=Homo sapiens OX=9606 GN=CCDC88C PE=1 SV=3
Q9P2I0	CPSF2	Cleavage and polyadenylation specificity factor subunit 2 OS=Homo sapiens OX=9606 GN=CPSF2 PE=1 SV=2
Q9UBG3	CRNN	Cornulin OS=Homo sapiens OX=9606 GN=CRNN PE=1 SV=1
Q9UBM7	DHCR7	7-dehydrocholesterol reductase OS=Homo sapiens OX=9606 GN=DHCR7 PE=1 SV=1
Q9ULX6	AKAP8L	A-kinase anchor protein 8-like OS=Homo sapiens OX=9606 GN=AKAP8L PE=1 SV=4
Q9UN86	G3BP2	Ras GTPase-activating protein-binding protein 2 OS=Homo sapiens OX=9606 GN=G3BP2 PE=1 SV=2
Q9UQ35	SRRM2	Serine/arginine repetitive matrix protein 2 OS=Homo sapiens OX=9606 GN=SRRM2 PE=1 SV=2
Q9Y2W1	THRAP3	Thyroid hormone receptor-associated protein 3 OS=Homo sapiens OX=9606 GN=THRAP3 PE=1 SV=2
Q9Y3R4	NEU2	Sialidase-2 OS=Homo sapiens OX=9606 GN=NEU2 PE=1 SV=2
Q9Y4L1	HYOU1	Hypoxia up-regulated protein 1 OS=Homo sapiens OX=9606 GN=HYOU1 PE=1 SV=1

Q9Y5B9	SUPT16H	FACT complex subunit SPT16 OS=Homo sapiens OX=9606 GN=SUPT16H PE=1 SV=1

Supplementary Table 12. Protein with enhanced binding to FBXO7 in degenerated NP cells.

Accession	GeneName	Description
Q93074	MED12	Mediator of RNA polymerase II transcription subunit 12 OS=Homo sapiens OX=9606 GN=MED12 PE=1 SV=4
Q9UKV3	ACIN1	Apoptotic chromatin condensation inducer in the nucleus OS=Homo sapiens OX=9606 GN=ACIN1 PE=1 SV=2
P16403	H1-2	Histone H1.2 OS=Homo sapiens OX=9606 GN=H1-2 PE=1 SV=2
Q15436	SEC23A	Protein transport protein Sec23A OS=Homo sapiens OX=9606 GN=SEC23A PE=1 SV=2
Q5UIP0	RIF1	Telomere-associated protein RIF1 OS=Homo sapiens OX=9606 GN=RIF1 PE=1 SV=2
Q9Y2W1	THRAP3	Thyroid hormone receptor-associated protein 3 OS=Homo sapiens OX=9606 GN=THRAP3 PE=1 SV=2
Q8NHV4	NEDD1	Protein NEDD1 OS=Homo sapiens OX=9606 GN=NEDD1 PE=1 SV=1
P47914	RPL29	Large ribosomal subunit protein eL29 OS=Homo sapiens OX=9606 GN=RPL29 PE=1 SV=2
A0A2R8Y4L2	HNRNPA1L3	Heterogeneous nuclear ribonucleoprotein A1-like 3 OS=Homo sapiens OX=9606 GN=HNRNPA1L3 PE=1 SV=2
P48634	PRRC2A	Protein PRRC2A OS=Homo sapiens OX=9606 GN=PRRC2A PE=1 SV=3
Q99878	H2AC14	Histone H2A type 1-J OS=Homo sapiens OX=9606 GN=H2AC14 PE=1 SV=3
Q9ULJ3	ZBTB21	Zinc finger and BTB domain-containing protein 21 OS=Homo sapiens OX=9606 GN=ZBTB21 PE=1 SV=2
Q29980	MICB	MHC class I polypeptide-related sequence B OS=Homo sapiens OX=9606 GN=MICB PE=1 SV=2
Q16629	SRSF7	Serine/arginine-rich splicing factor 7 OS=Homo sapiens OX=9606 GN=SRSF7 PE=1 SV=1
Q15437	SEC23B	Protein transport protein Sec23B OS=Homo sapiens OX=9606 GN=SEC23B PE=1 SV=2

O00425	IGF2BP3	Insulin-like growth factor 2 mRNA-binding protein 3 OS=Homo sapiens OX=9606 GN=IGF2BP3 PE=1 SV=2
Q9Y3Y2	CHTOP	Chromatin target of PRMT1 protein OS=Homo sapiens OX=9606 GN=CHTOP PE=1 SV=2
P62851	RPS25	Small ribosomal subunit protein eS25 OS=Homo sapiens OX=9606 GN=RPS25 PE=1 SV=1
P62995	TRA2B	Transformer-2 protein homolog beta OS=Homo sapiens OX=9606 GN=TRA2B PE=1 SV=1
P84098	RPL19	Large ribosomal subunit protein eL19 OS=Homo sapiens OX=9606 GN=RPL19 PE=1 SV=1
Q86U42	PABPN1	Polyadenylate-binding protein 2 OS=Homo sapiens OX=9606 GN=PABPN1 PE=1 SV=3
Q14839	CHD4	Chromodomain-helicase-DNA-binding protein 4 OS=Homo sapiens OX=9606 GN=CHD4 PE=1 SV=2
Q14980	NUMA1	Nuclear mitotic apparatus protein 1 OS=Homo sapiens OX=9606 GN=NUMA1 PE=1 SV=2
P10809	HSPD1	60 kDa heat shock protein, mitochondrial OS=Homo sapiens OX=9606 GN=HSPD1 PE=1 SV=2
Q8IX01	SUGP2	SURP and G-patch domain-containing protein 2 OS=Homo sapiens OX=9606 GN=SUGP2 PE=1 SV=2
Q8NI36	WDR36	WD repeat-containing protein 36 OS=Homo sapiens OX=9606 GN=WDR36 PE=1 SV=1
Q9BPU6	DPYSL5	Dihydropyrimidinase-related protein 5 OS=Homo sapiens OX=9606 GN=DPYSL5 PE=1 SV=1
Q5TEC6	H3-7	Histone H3-7 OS=Homo sapiens OX=9606 GN=H3-7 PE=1 SV=1
Q9Y623	MYH4	Myosin-4 OS=Homo sapiens OX=9606 GN=MYH4 PE=1 SV=2
A4UGR9	XIRP2	Xin actin-binding repeat-containing protein 2 OS=Homo sapiens OX=9606 GN=XIRP2 PE=1 SV=2
