# Supplementary material for: Dysregulated methylation-ubiquitination crosstalk accelerates intervertebral disc degeneration via MED12 destabilization and cGAS/STING activation
Source: J Clin Invest. 2026 Jun 30;136(16):e197274. doi: 10.1172/JCI197274 (PMC13476149; doi:10.1172/JCI197274)

Fig1D

MMA-IP

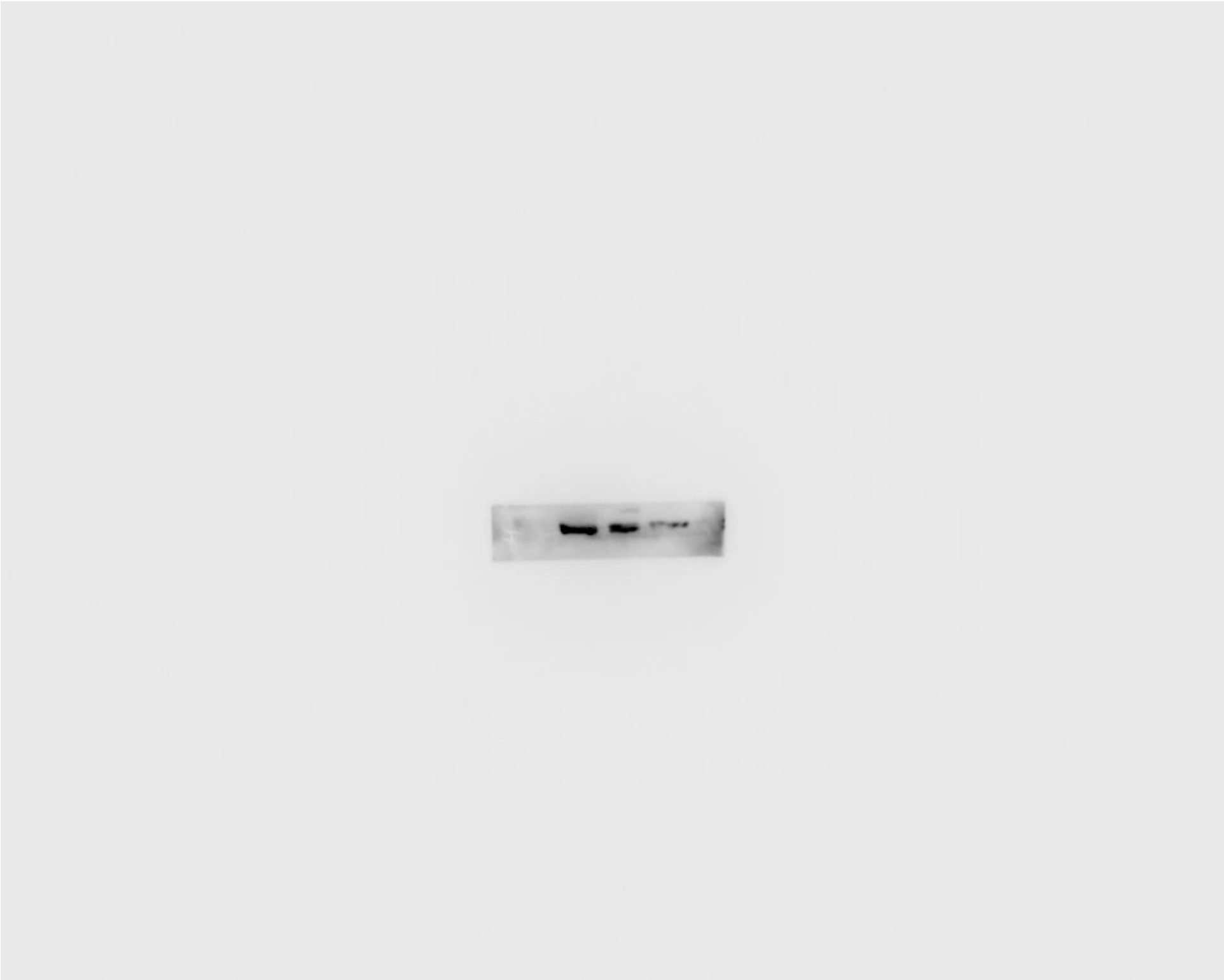

GFP-IP

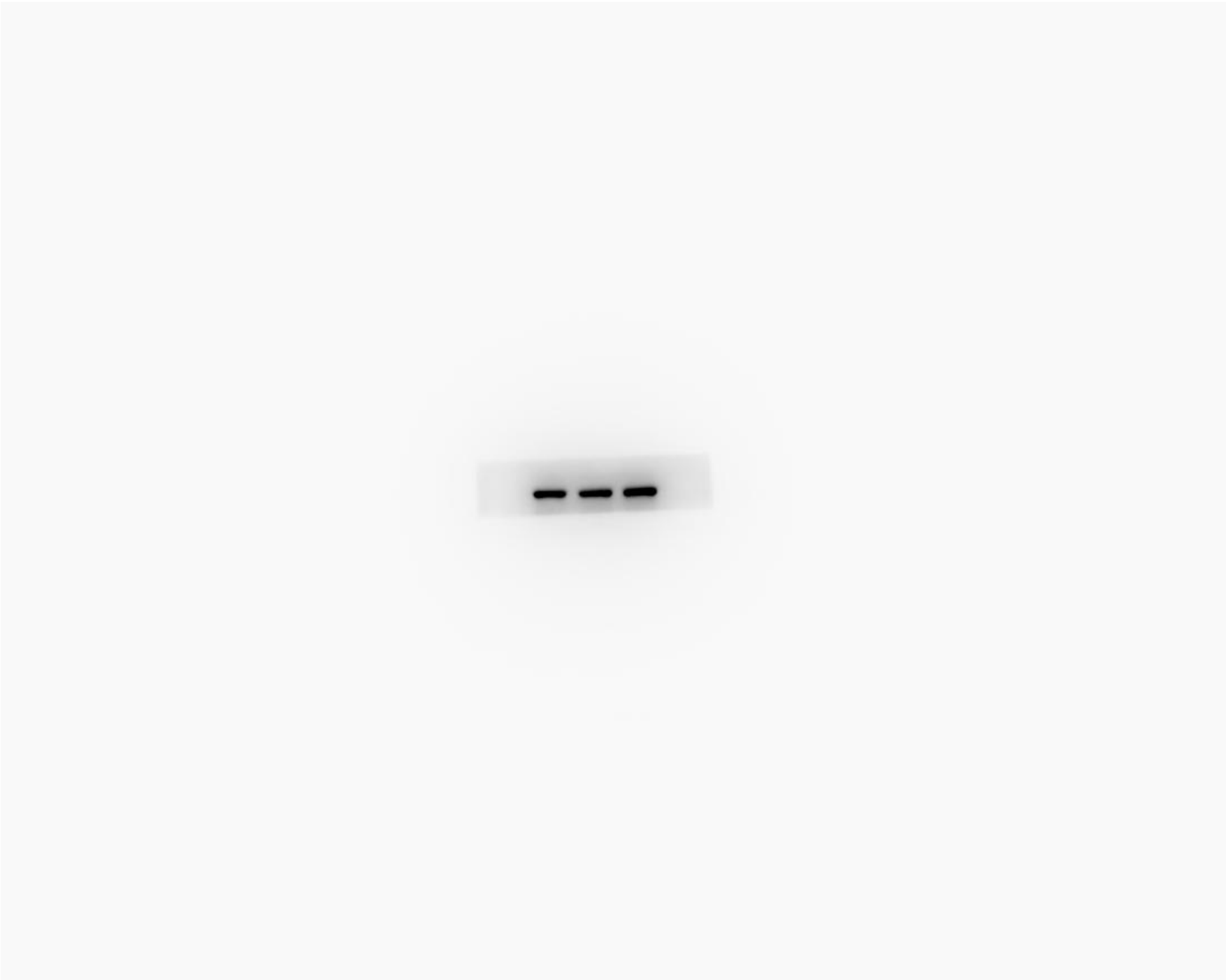

GFP-Input

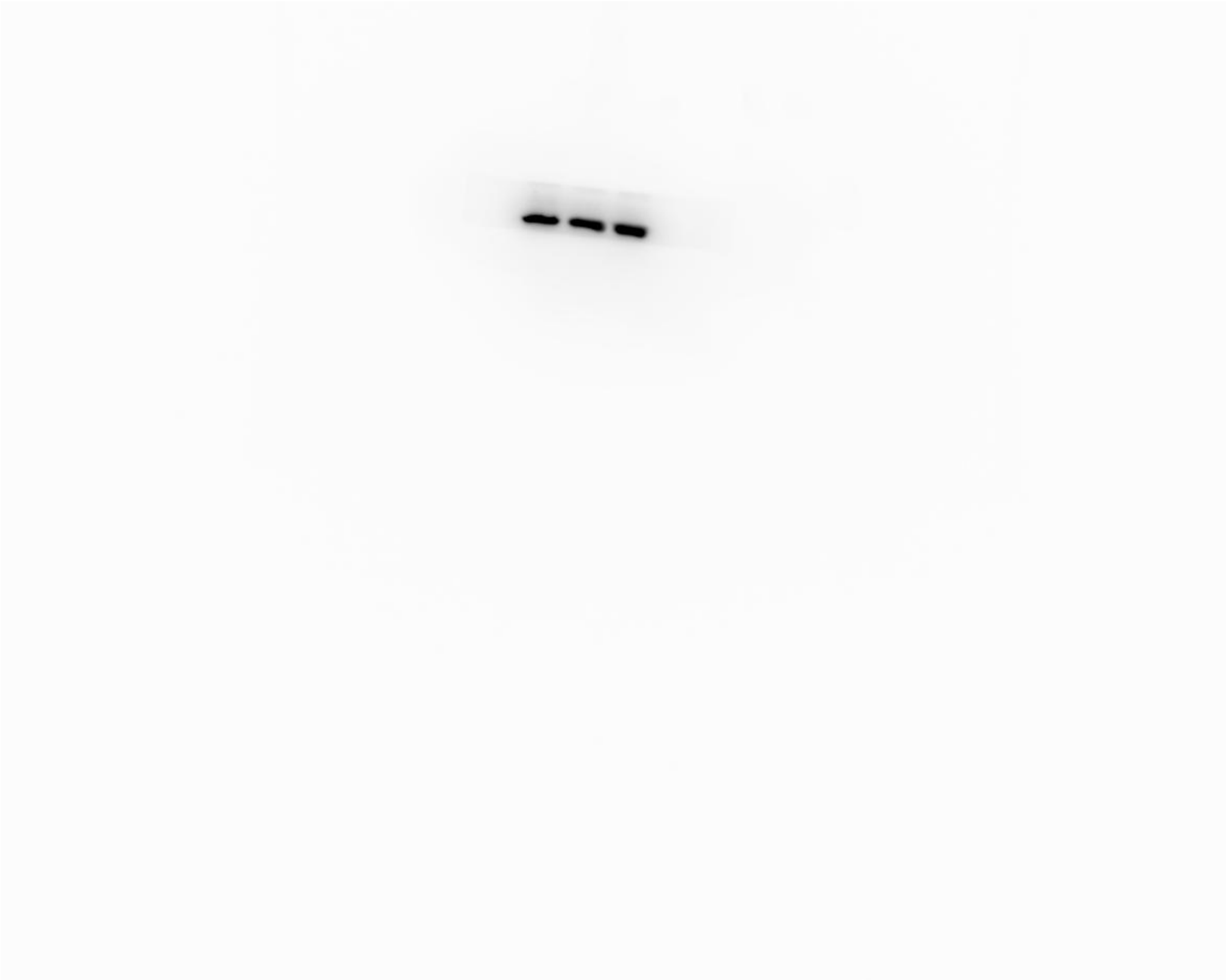

ACTIN-Input

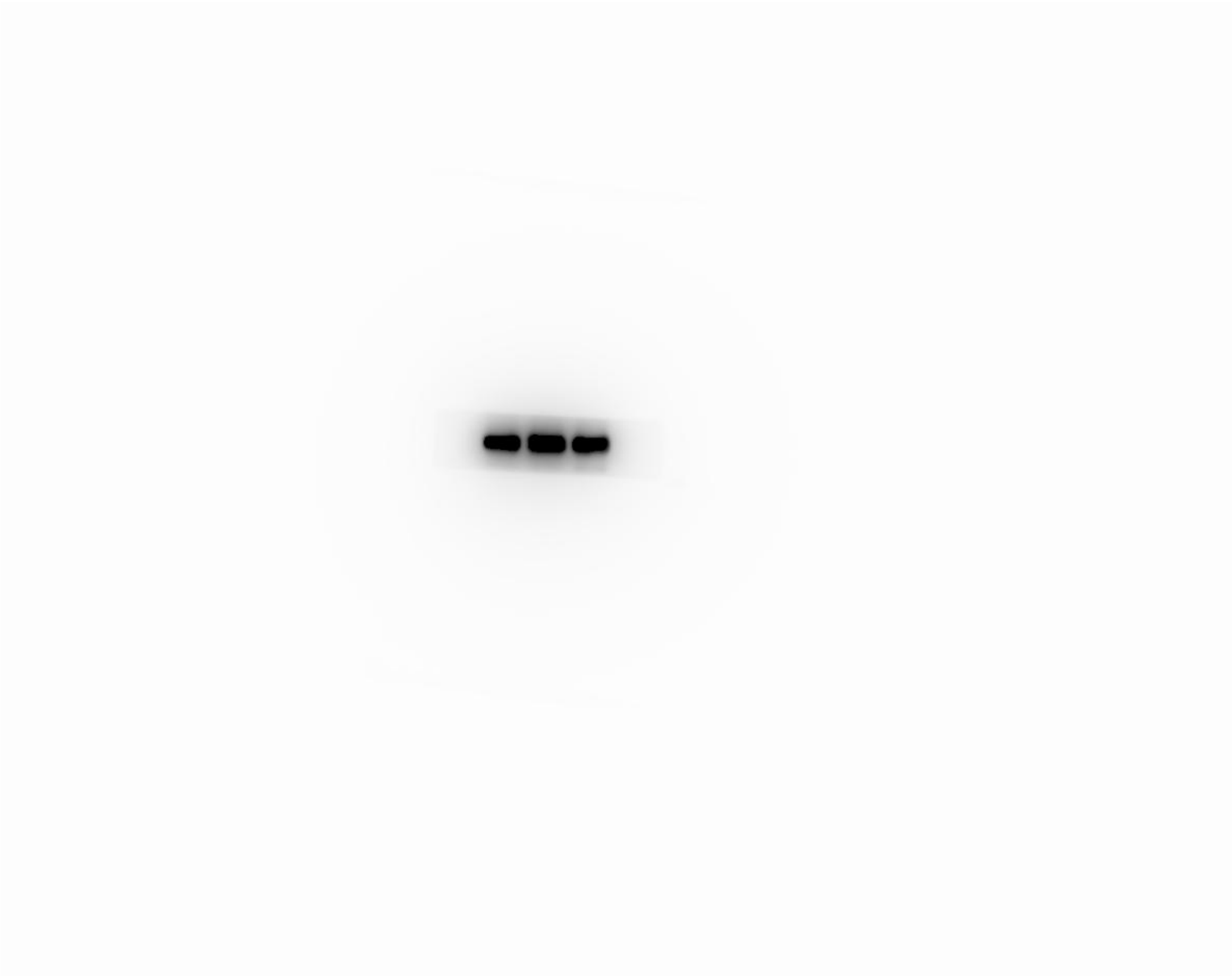

Fig1E

MMA-IP

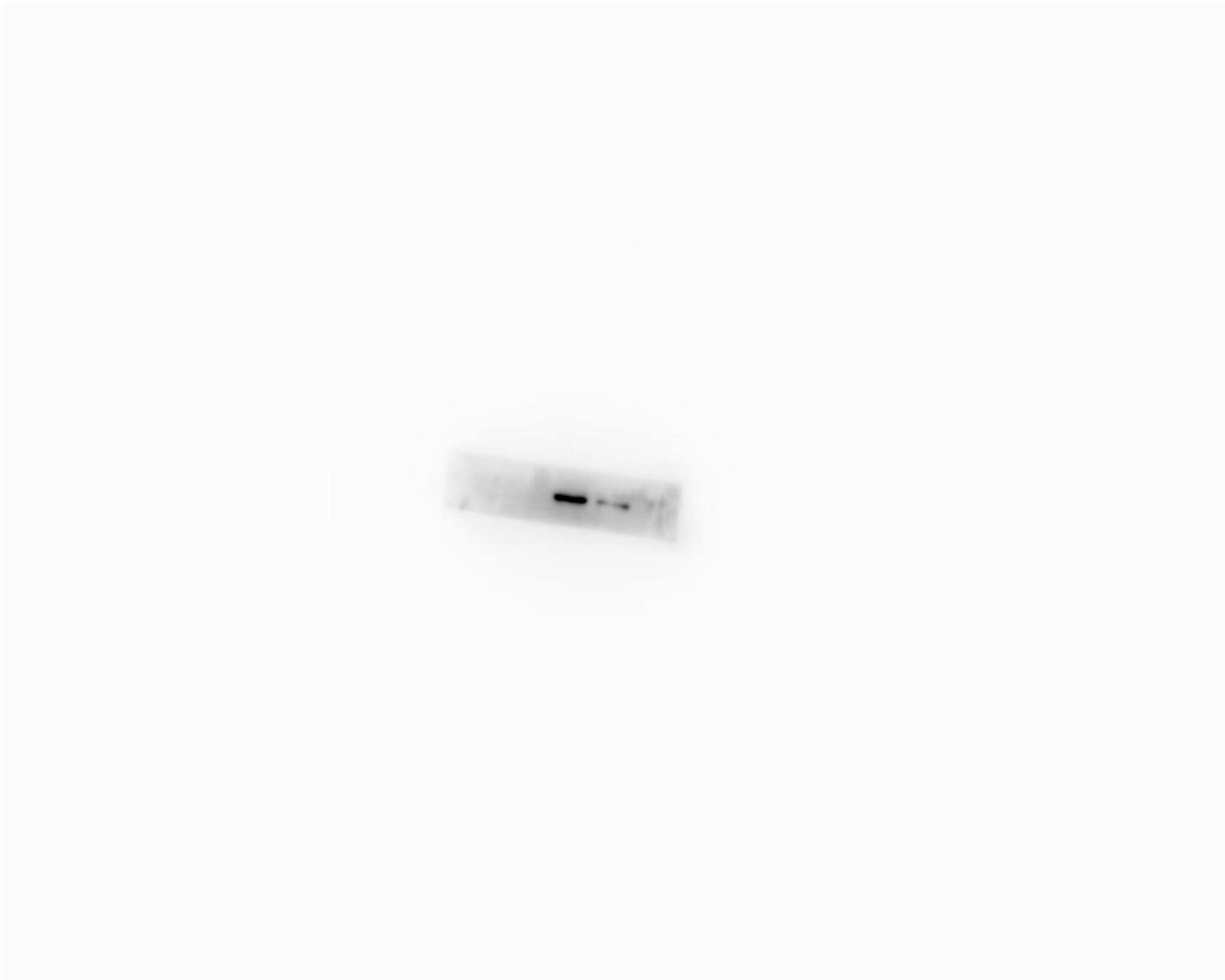

FBXO7-IP

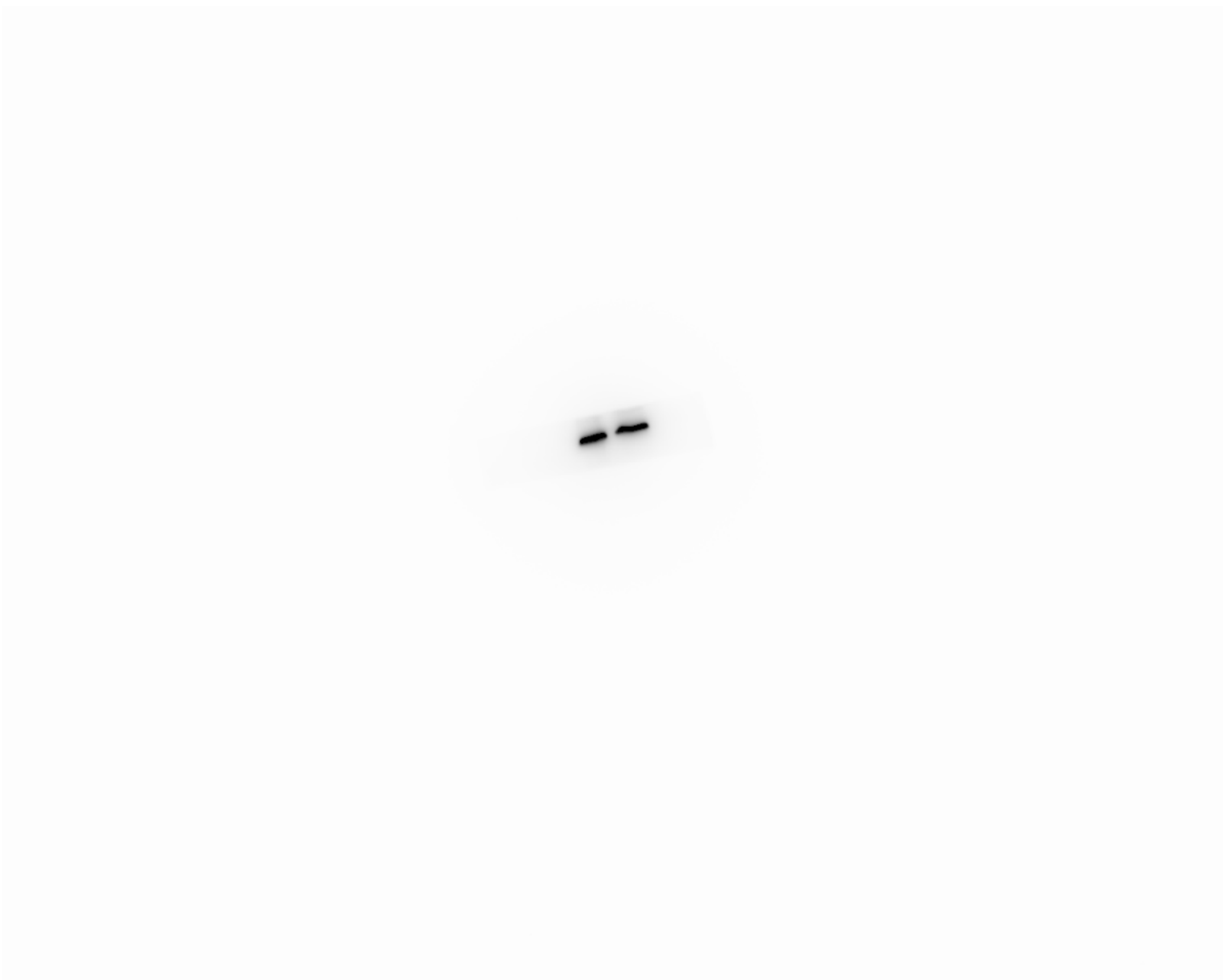

FBXO7-Input

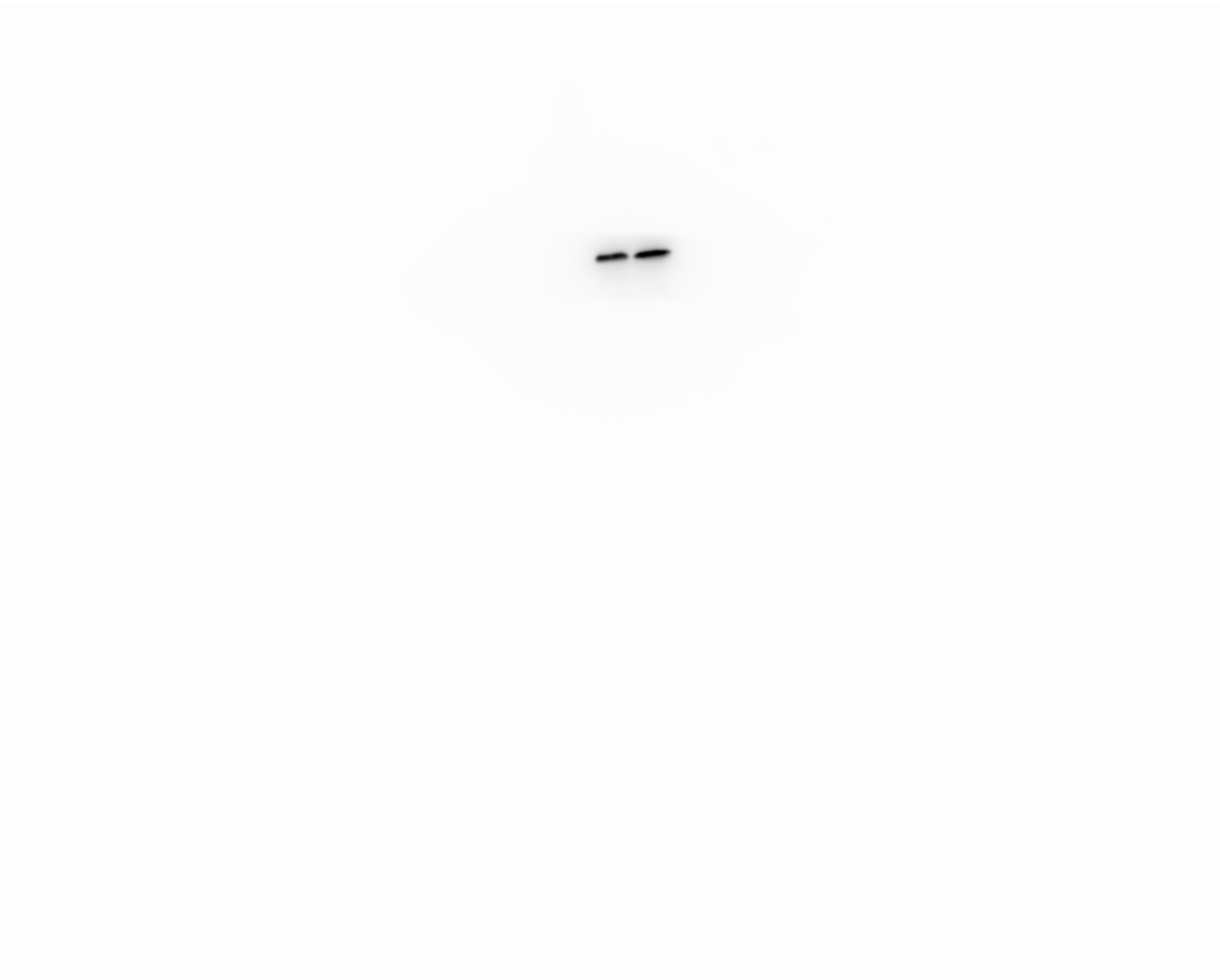

ACTIN-Input

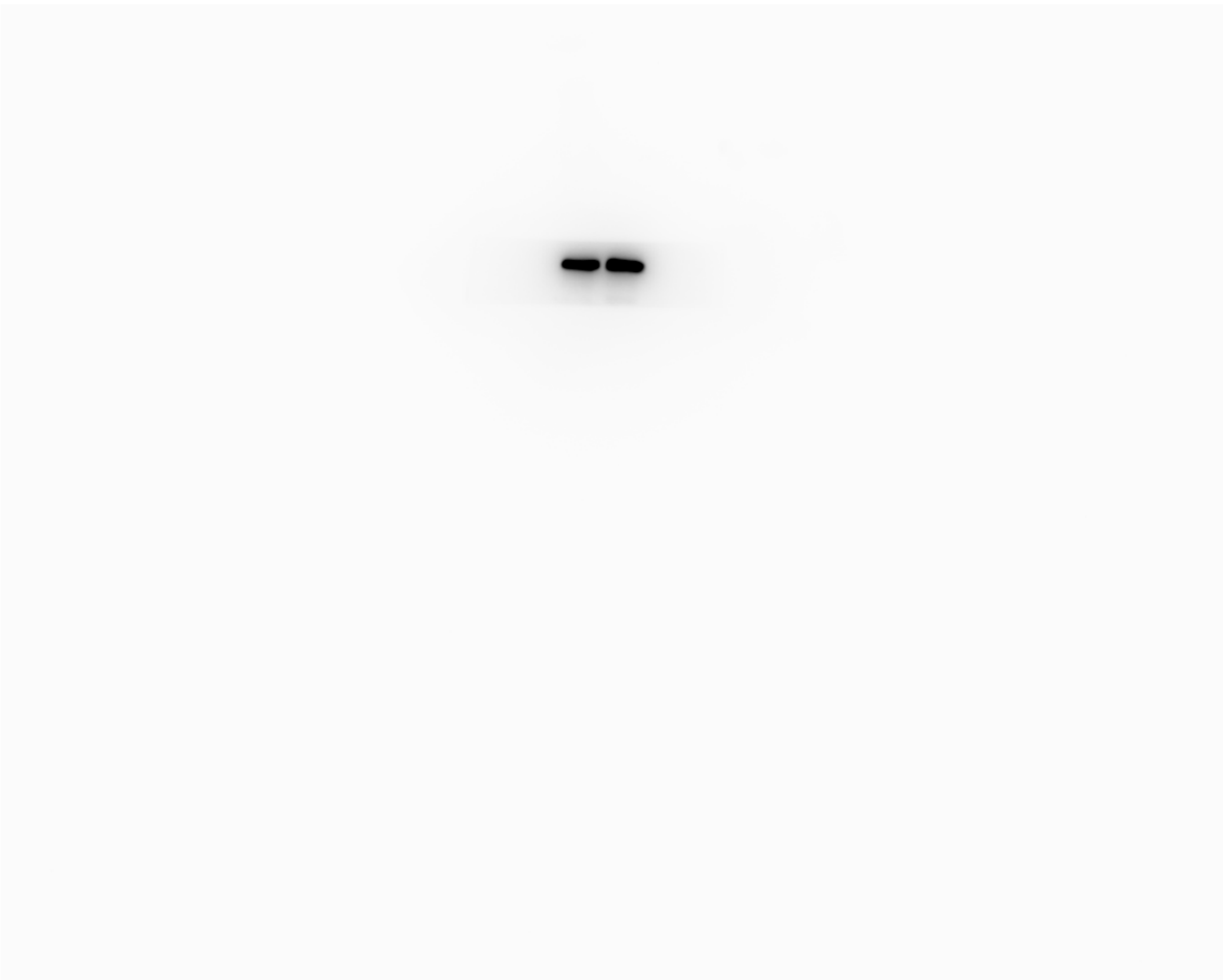

Fig1F

FBXO7me-IP

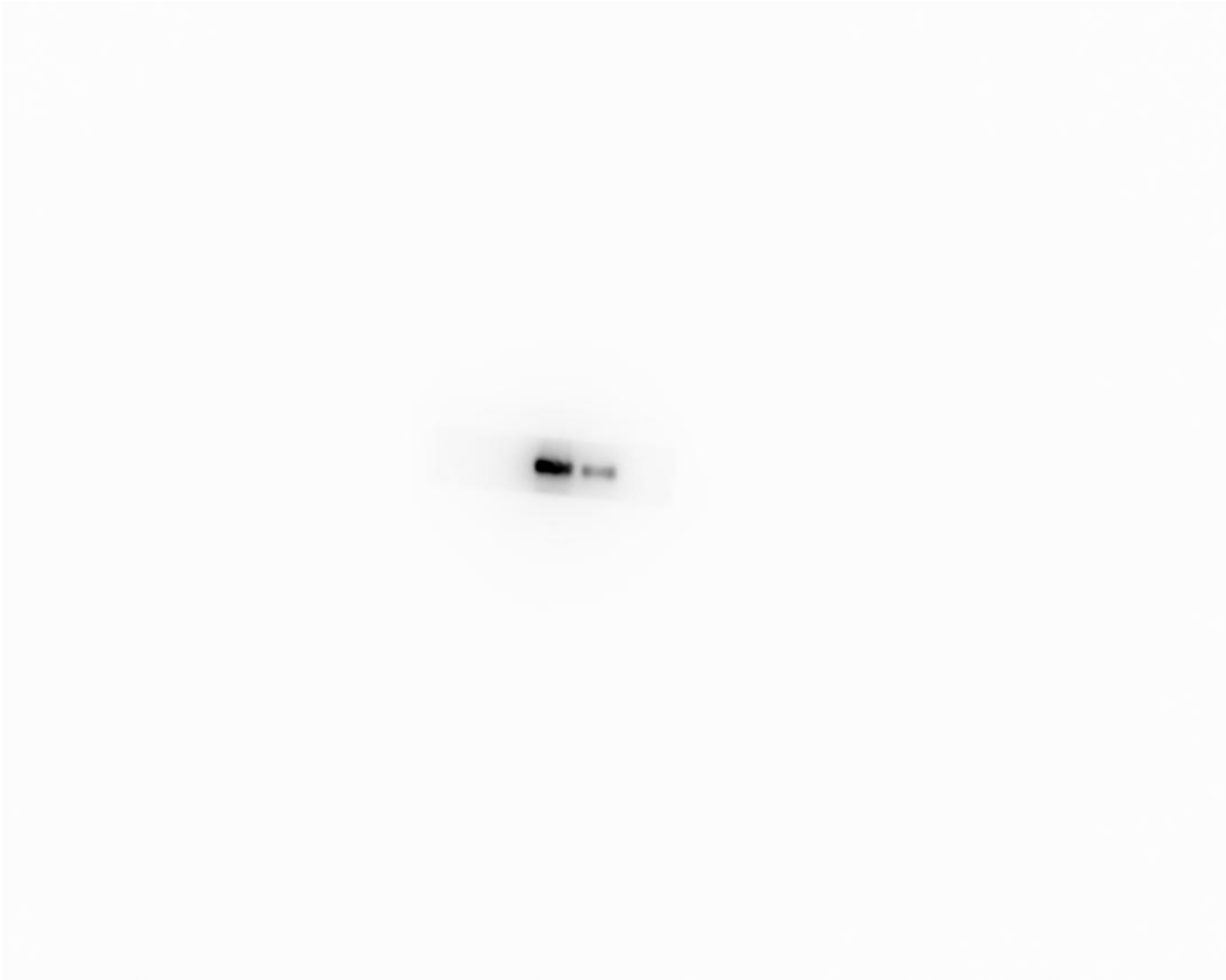

FBXO7-IP

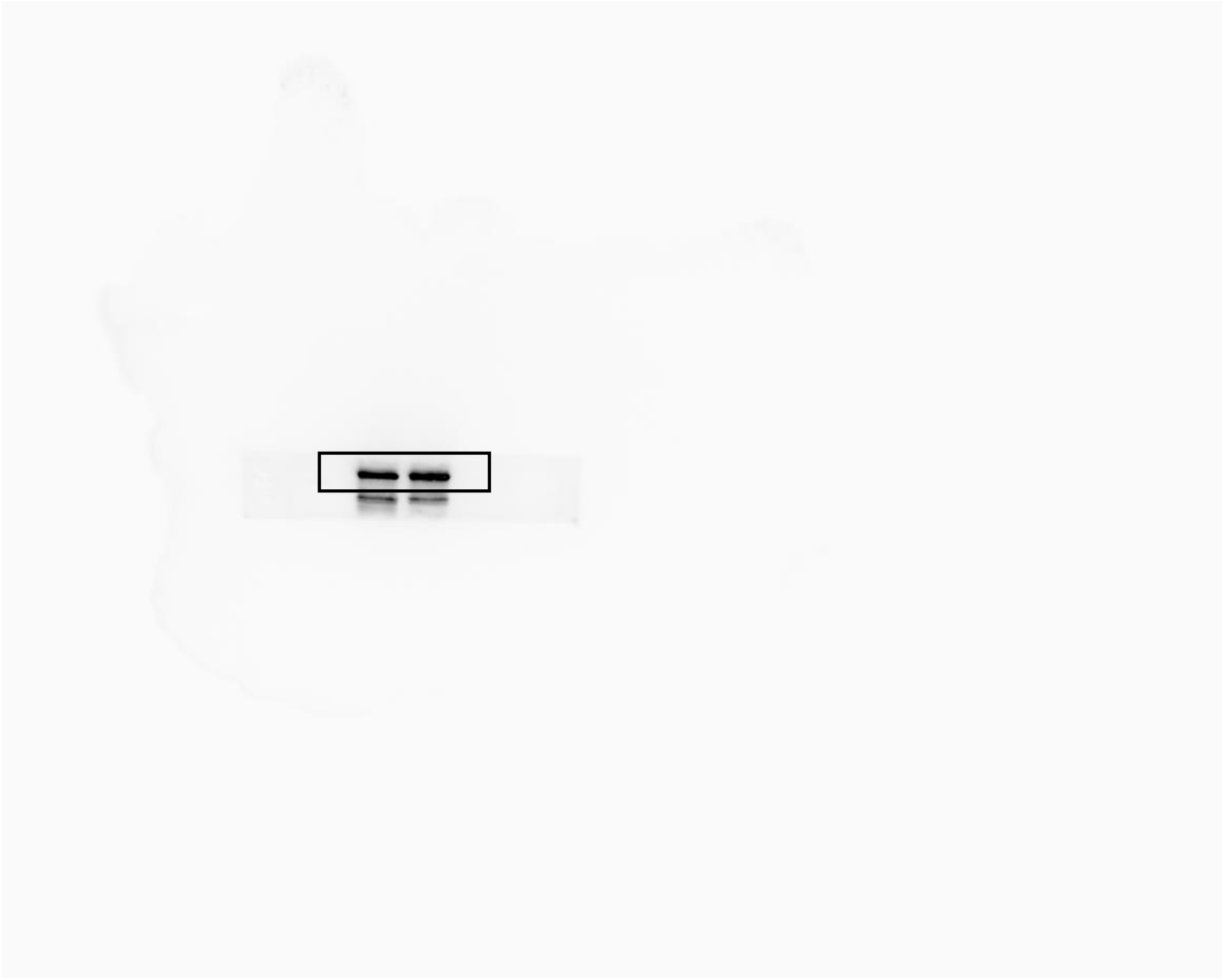

FBXO7-Input

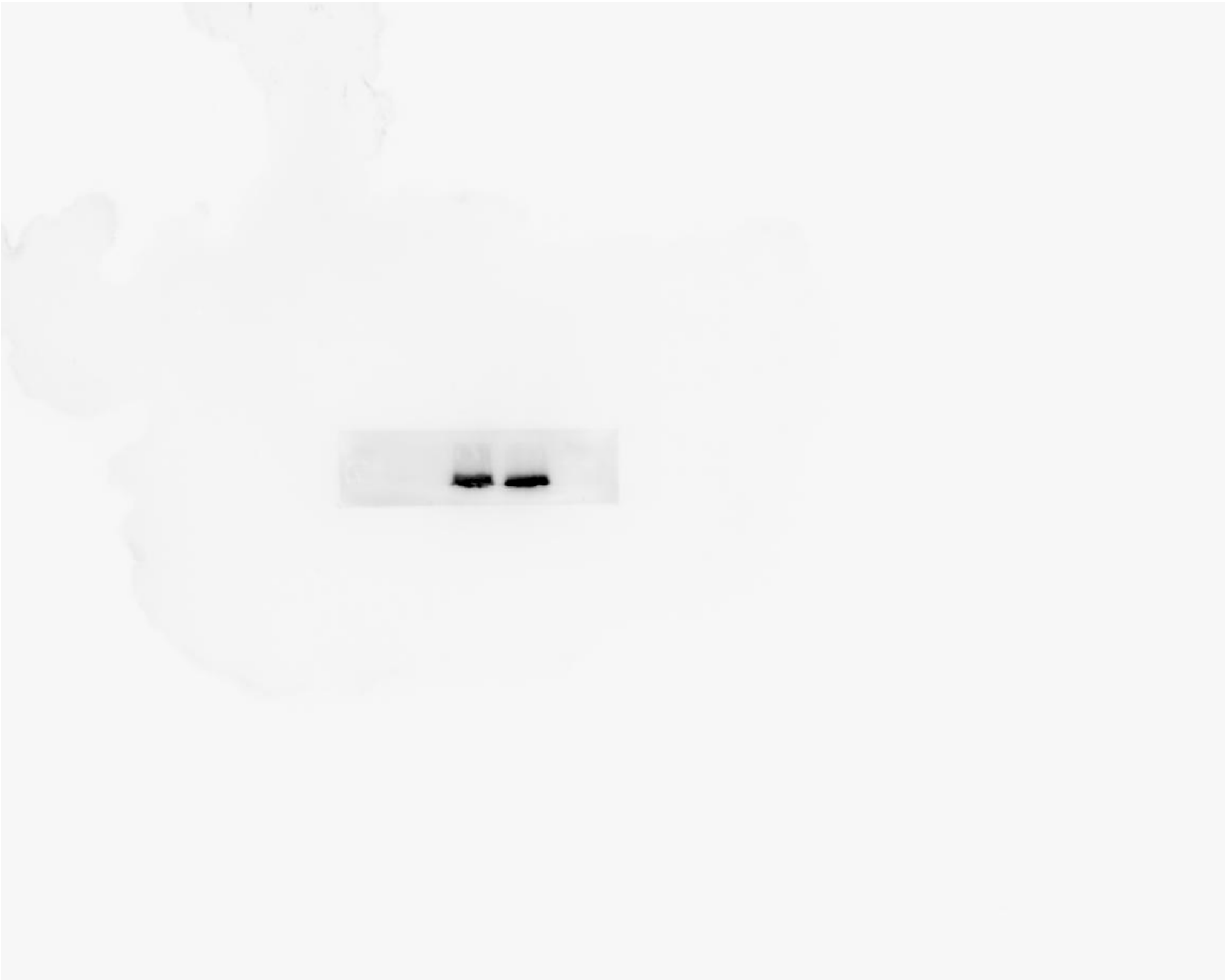

ACTIN-Input

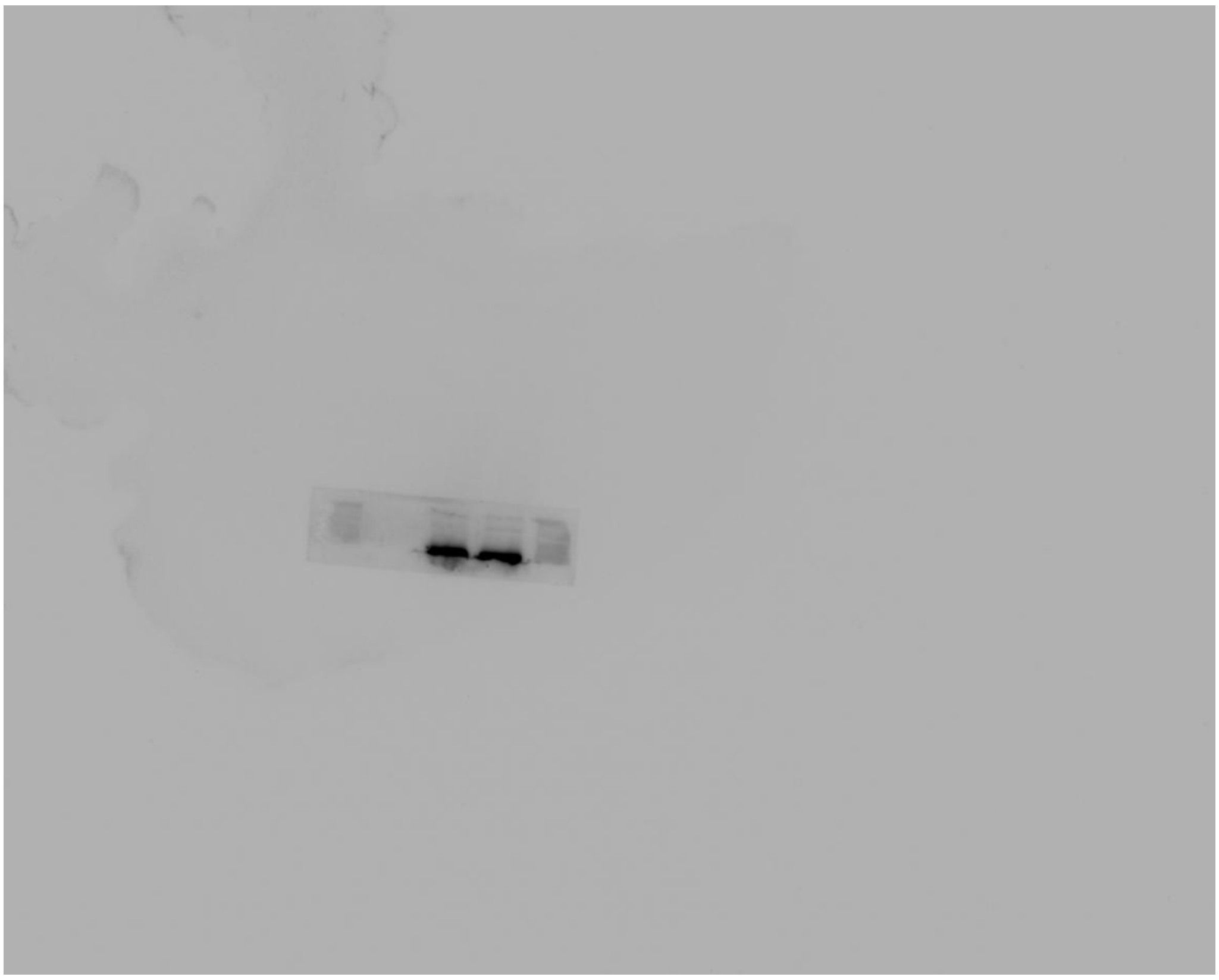

Fig1G

FBXO7me-IP

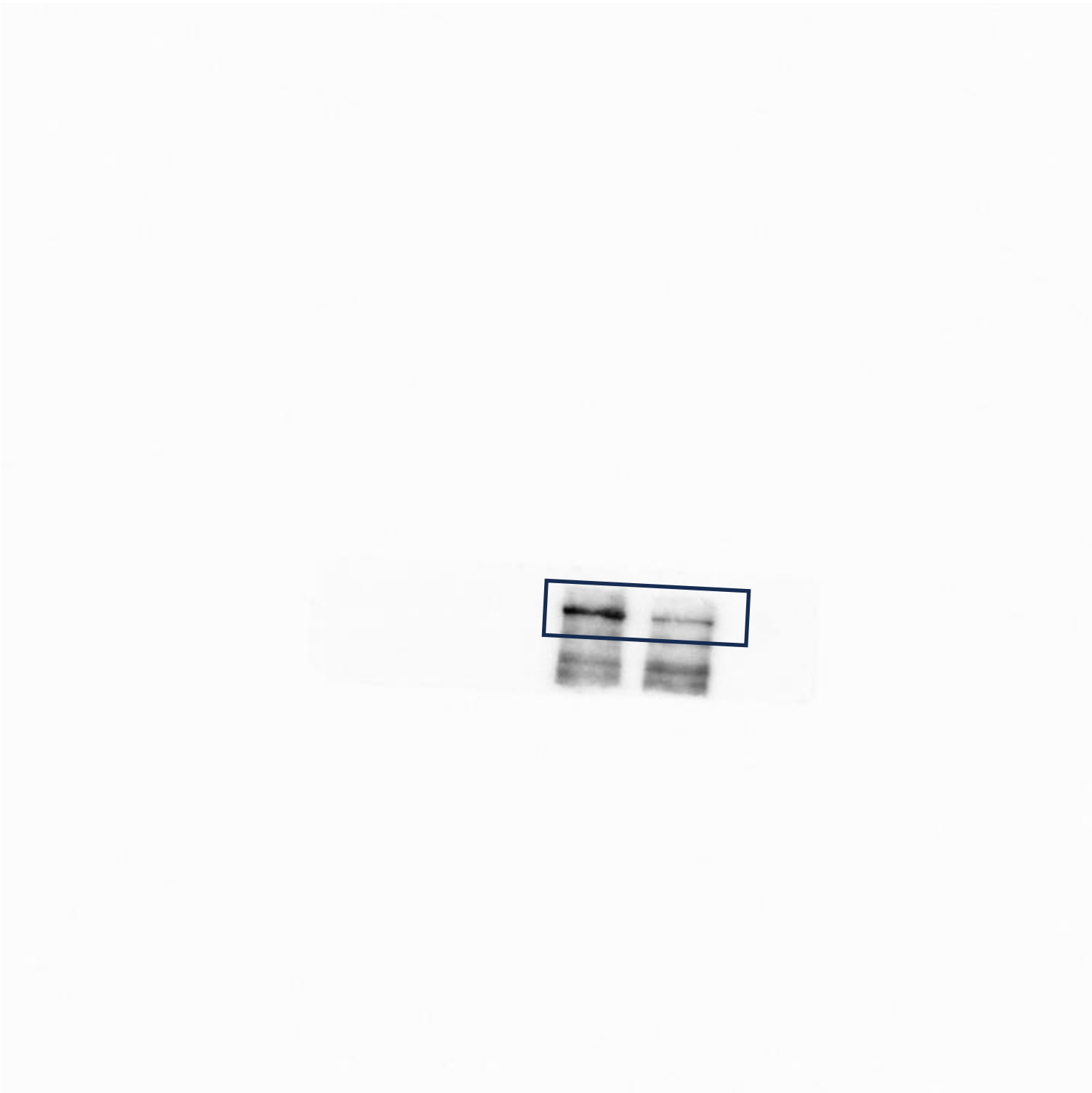

FBXO7-IP

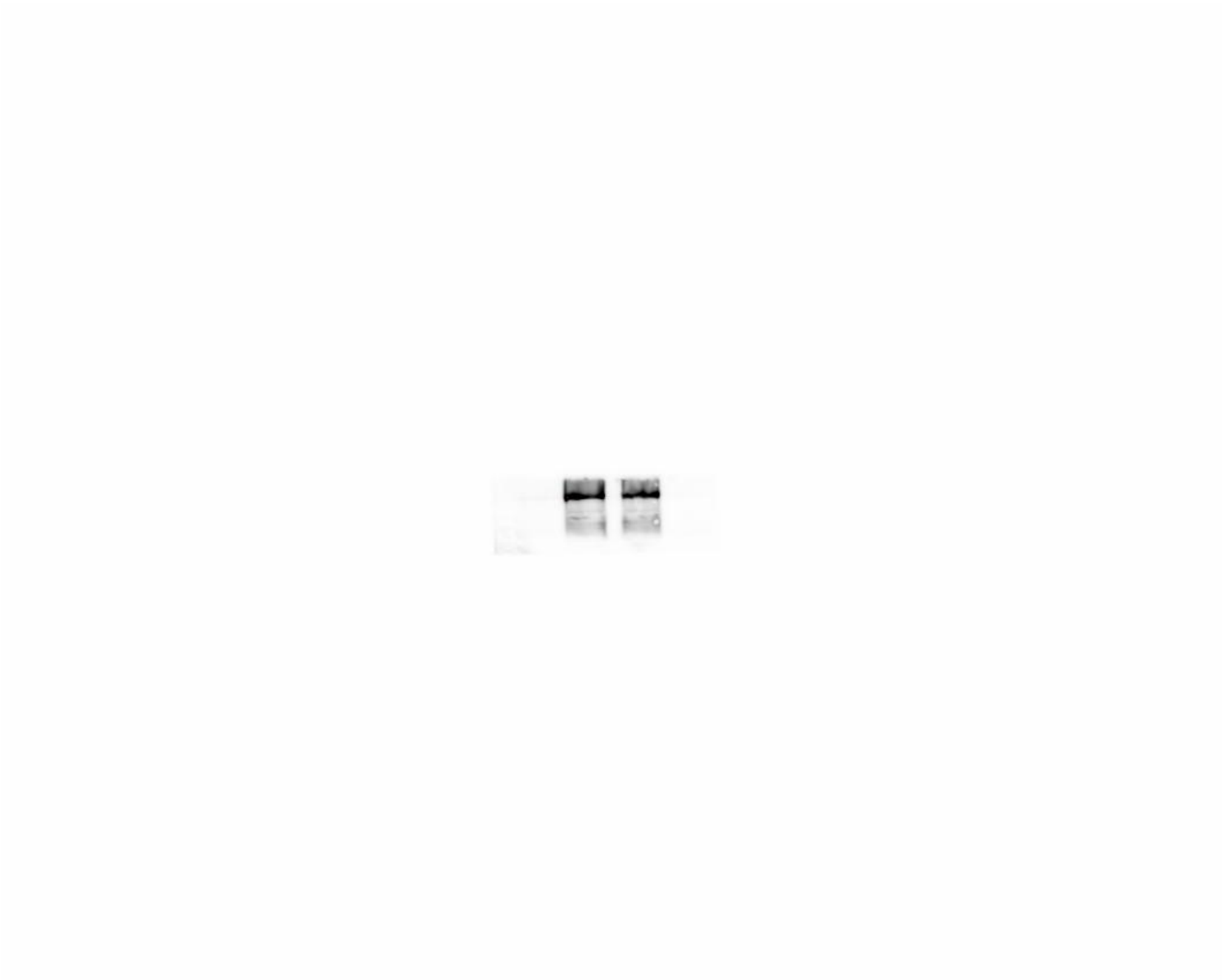

FBXO7-Input

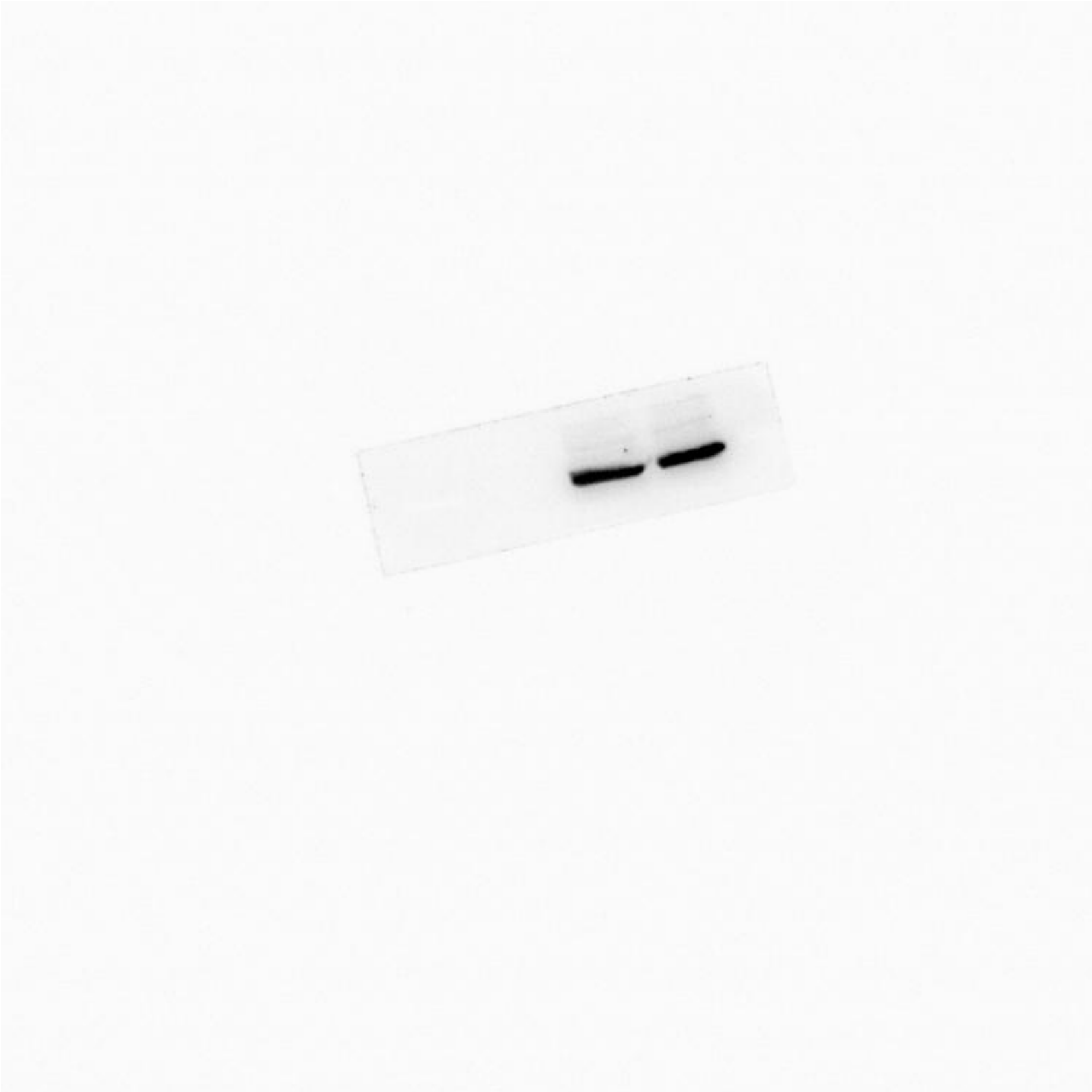

ACTIN-Input

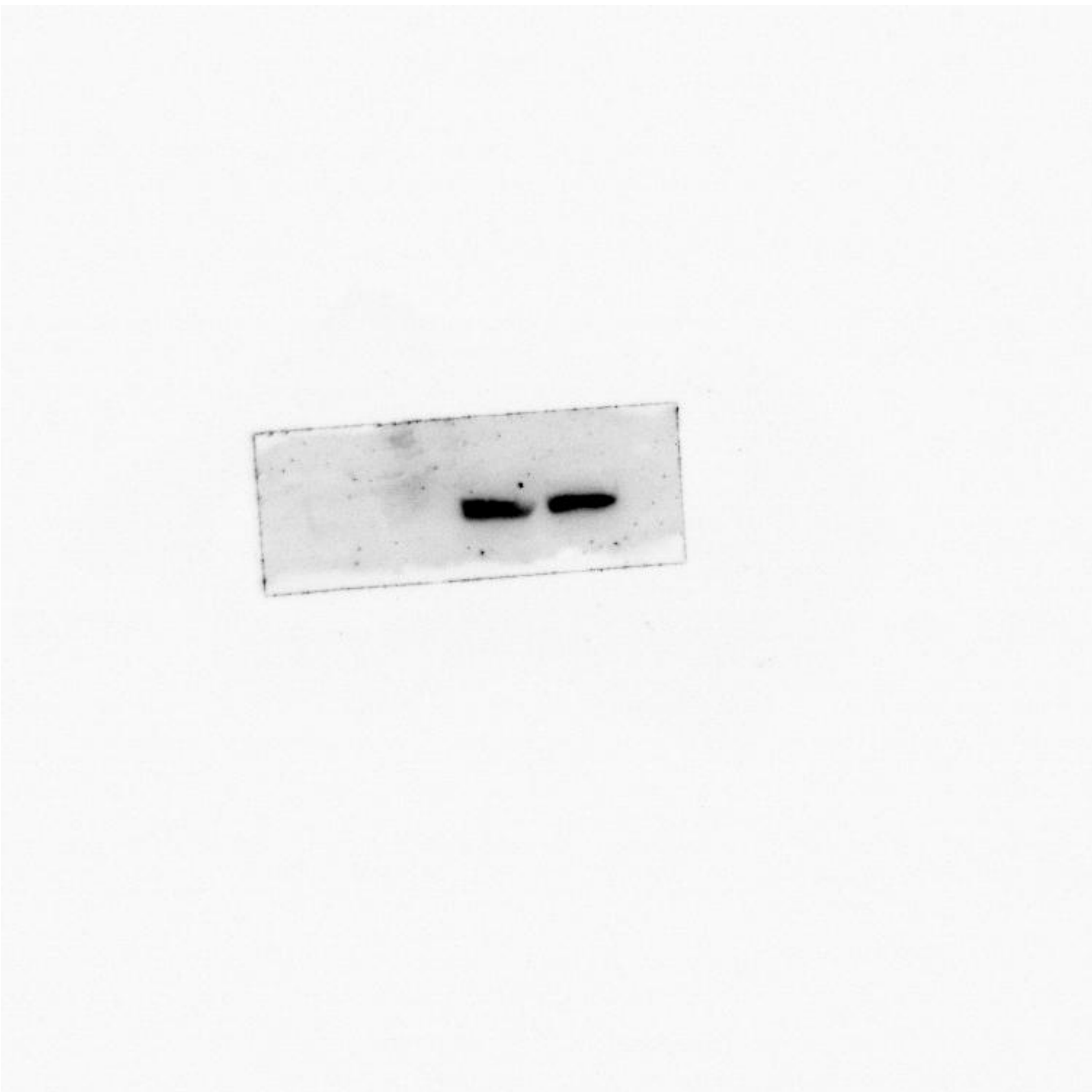

Fig3A

HA-IP

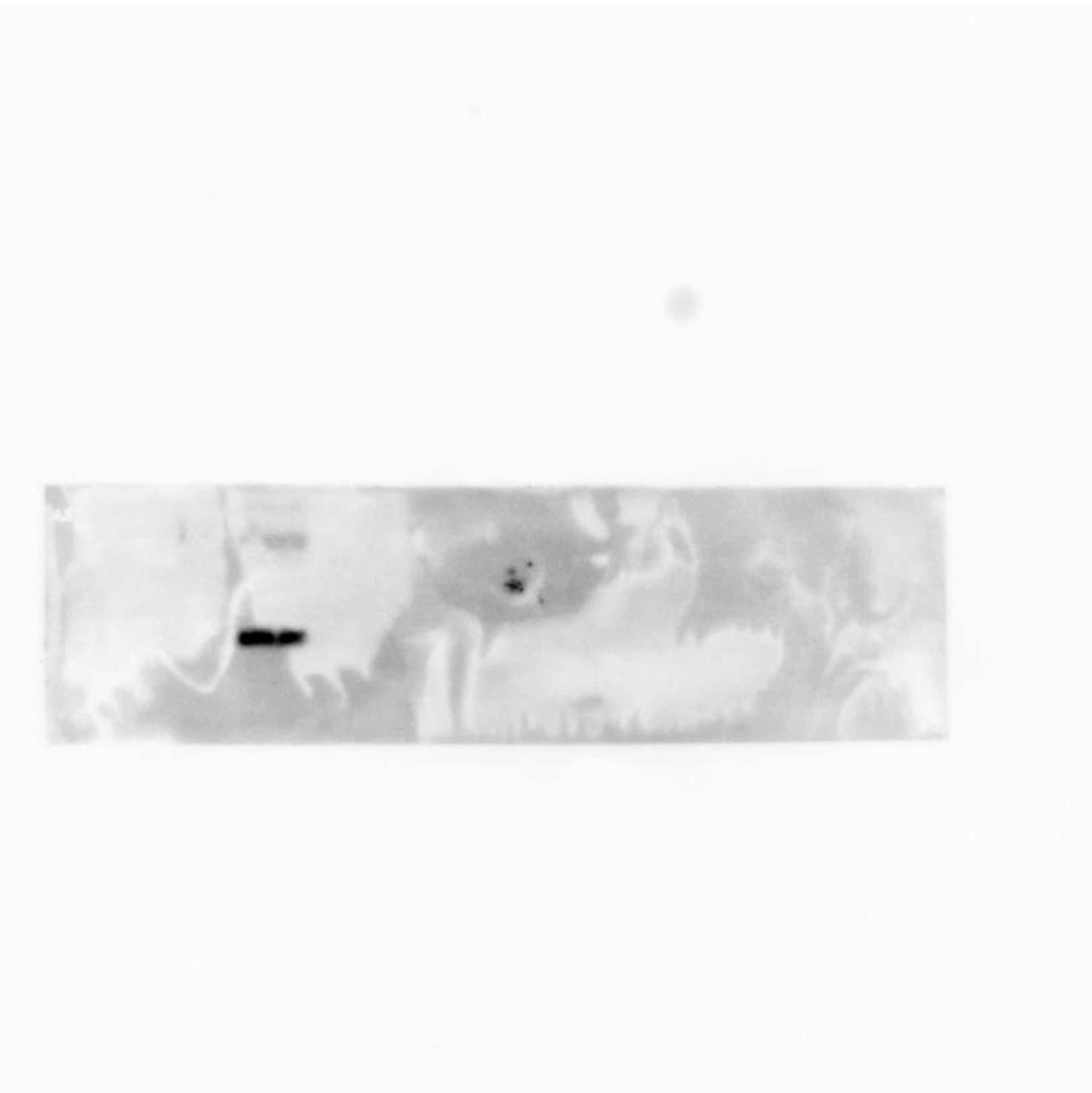

GFP-IP

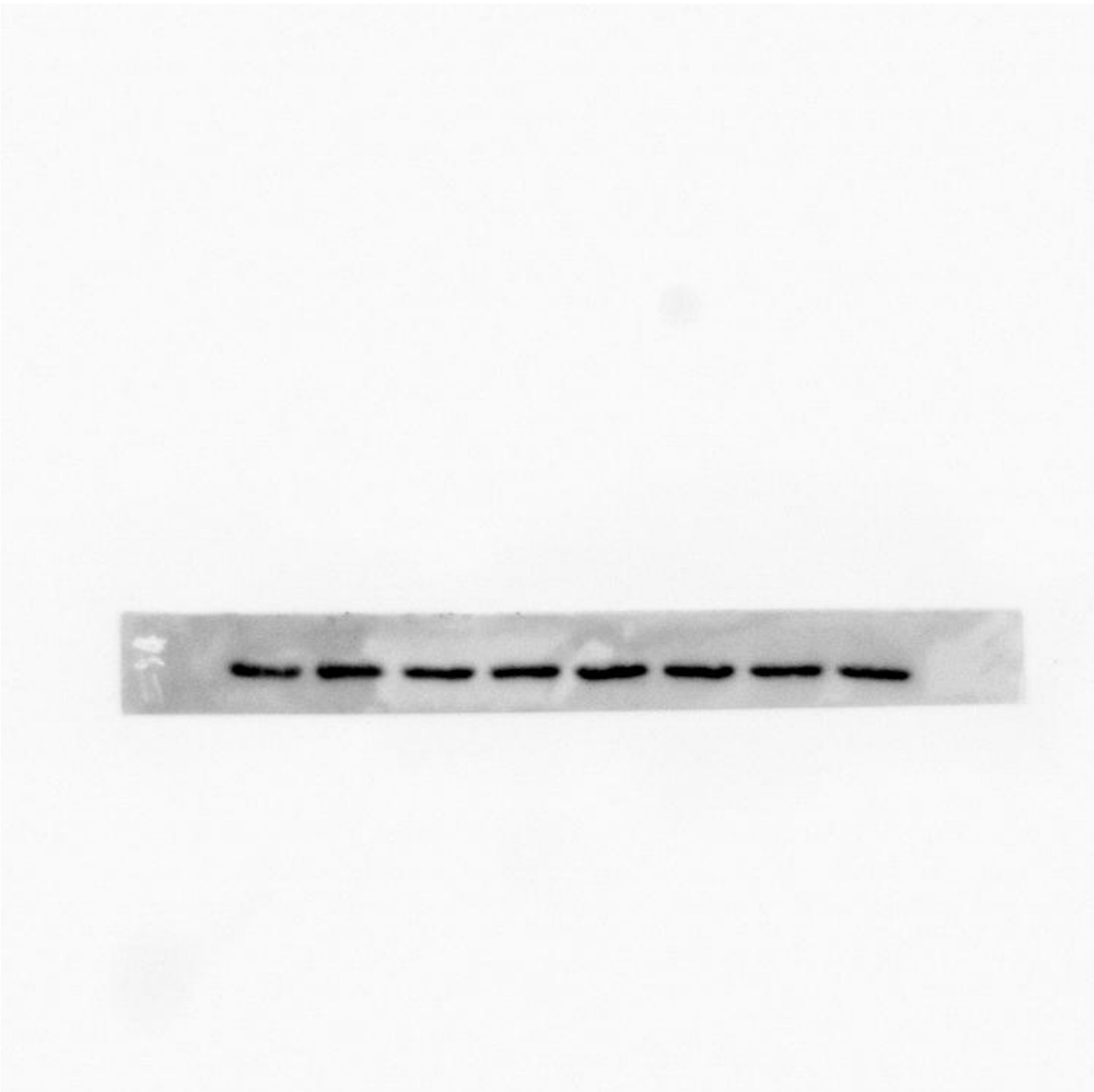

HA-Input

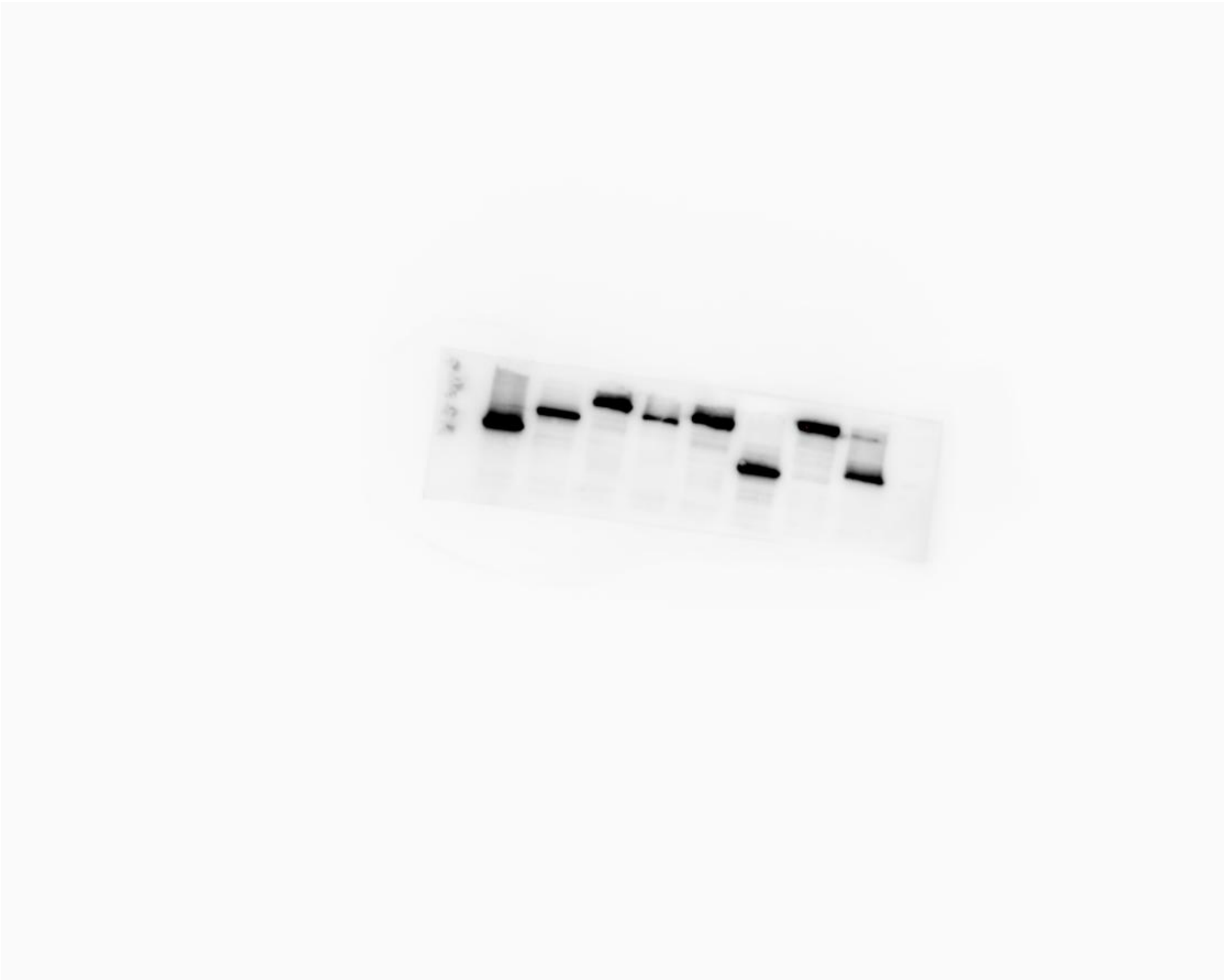

GFP-Input

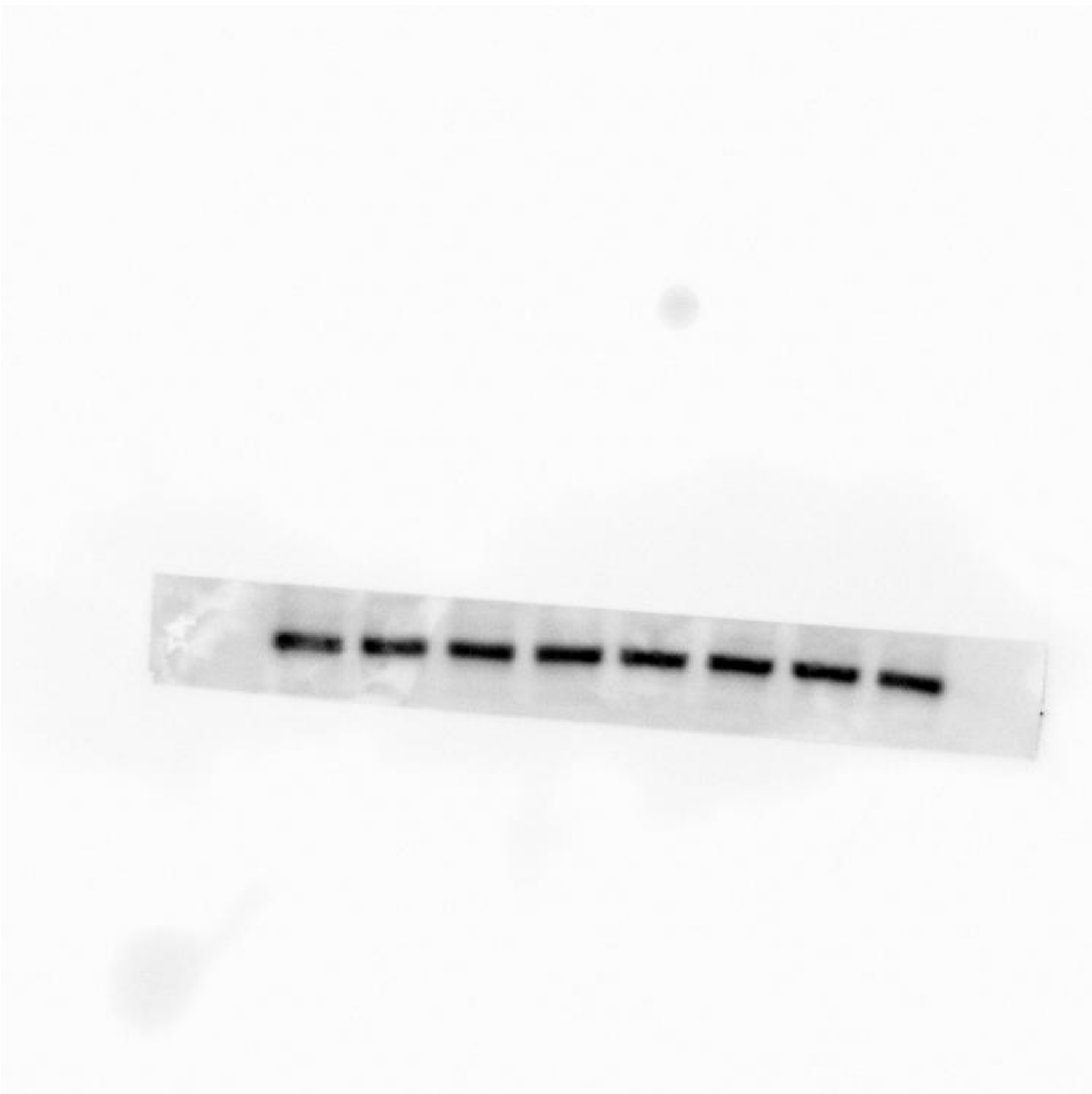

Fig3B

HA-IP

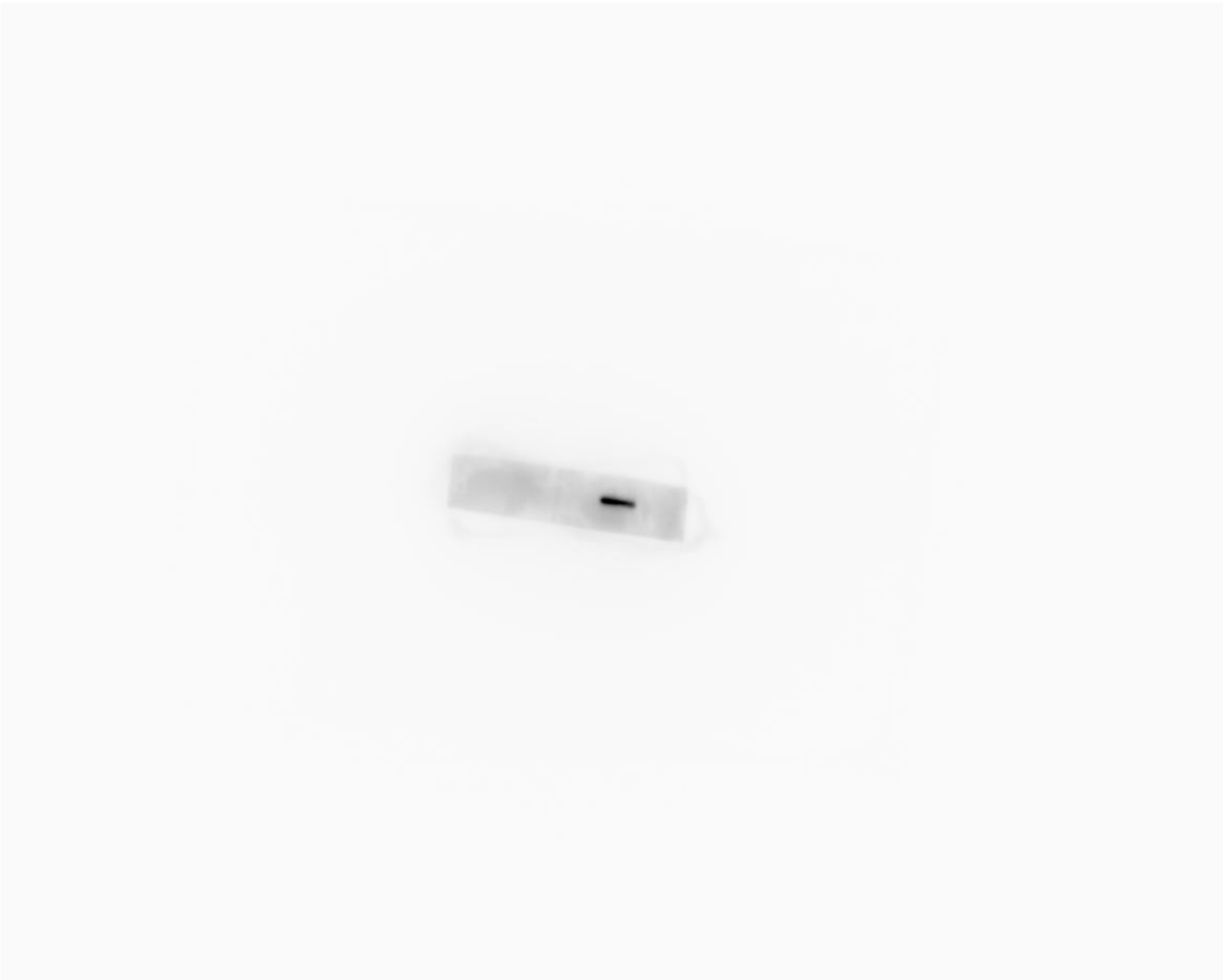

GFP-IP

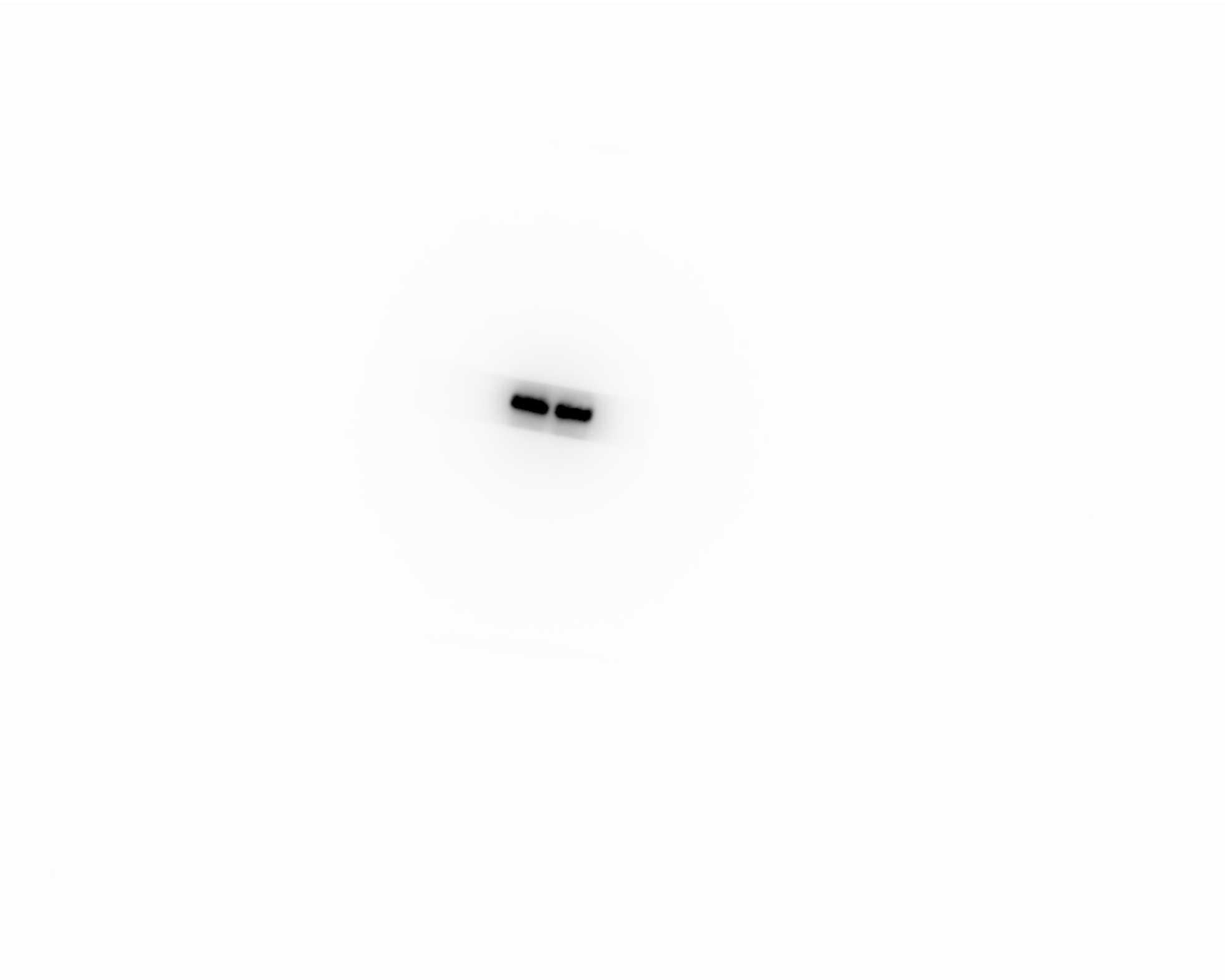

HA-Input

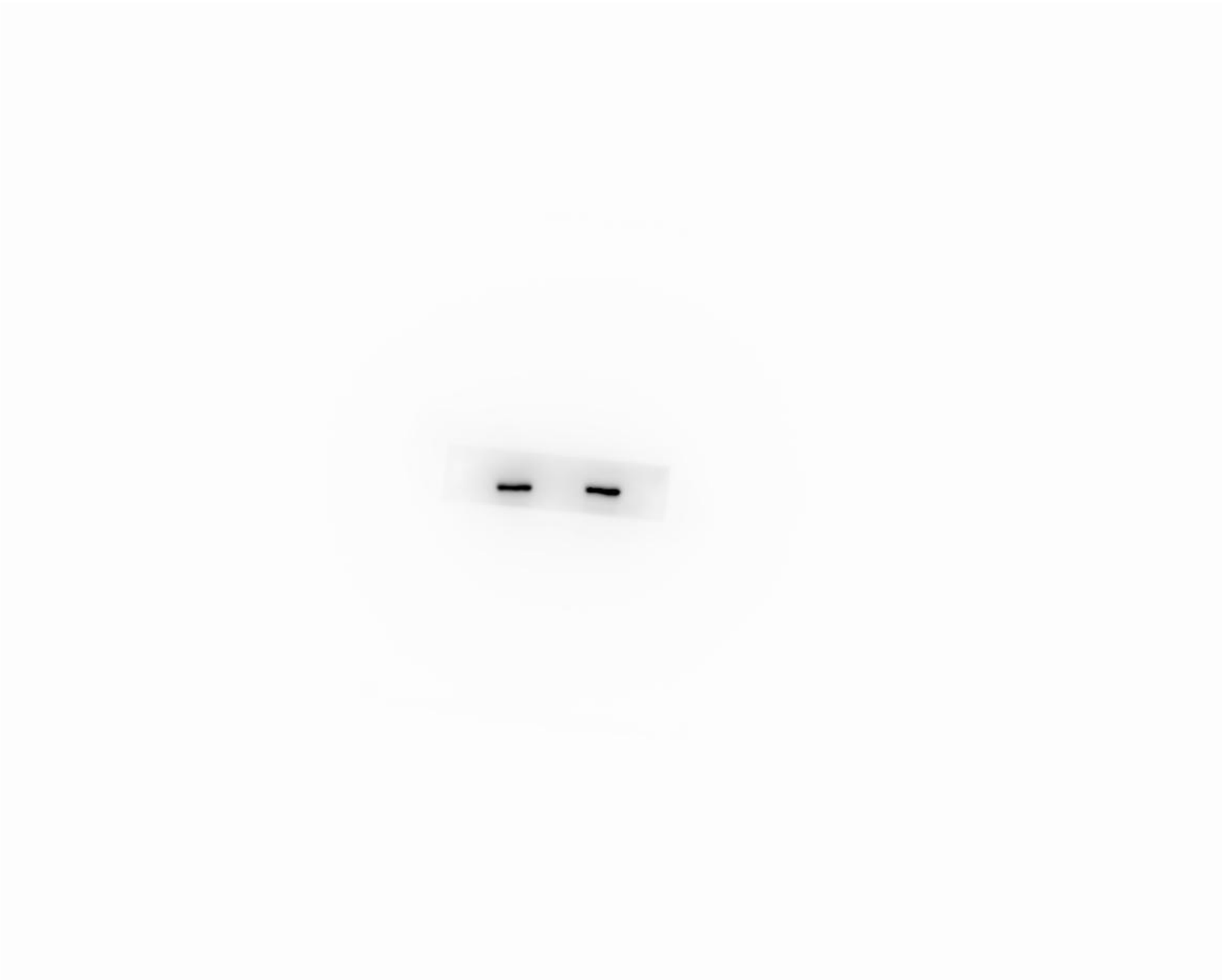

GFP-Input

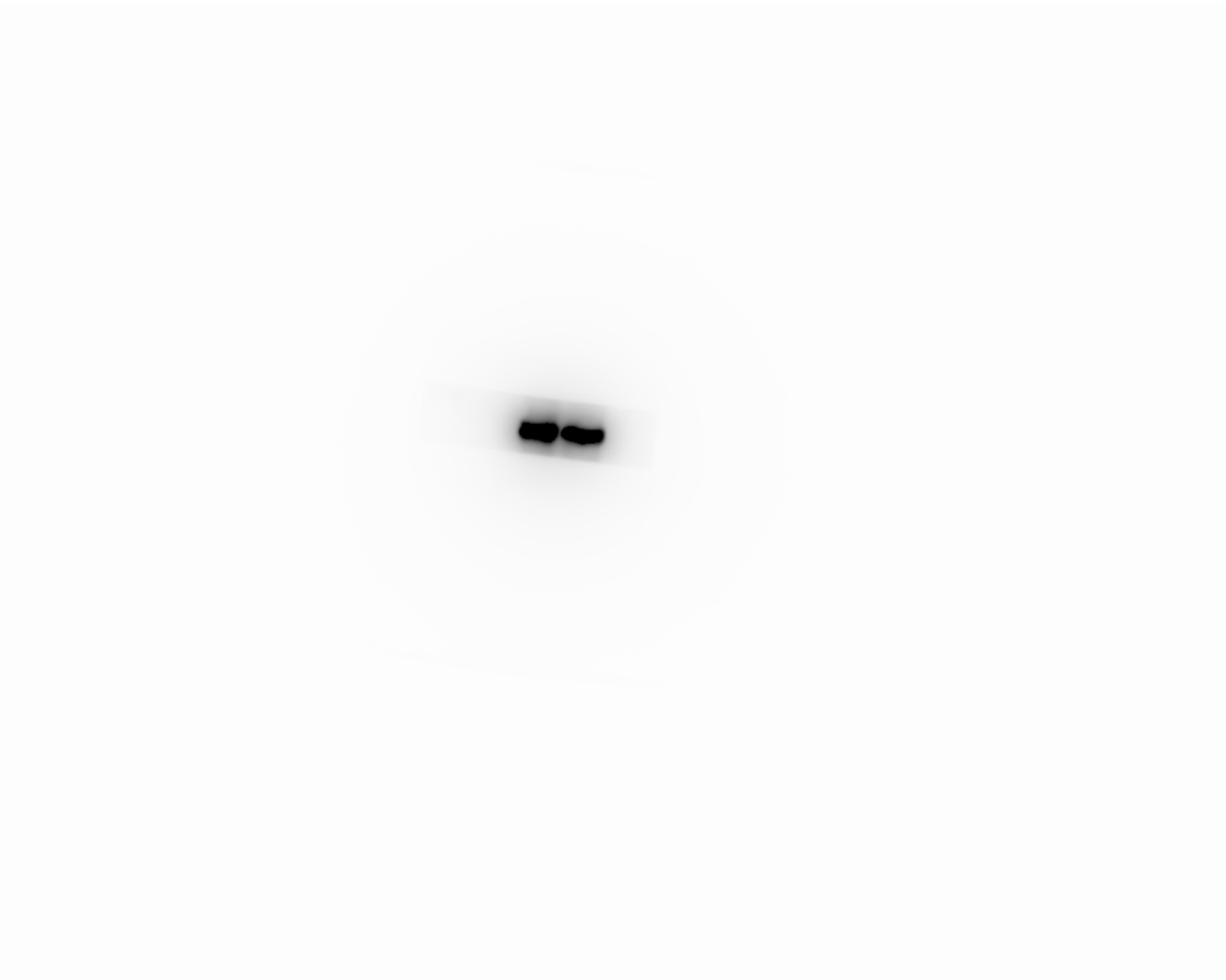

GADPH-Input

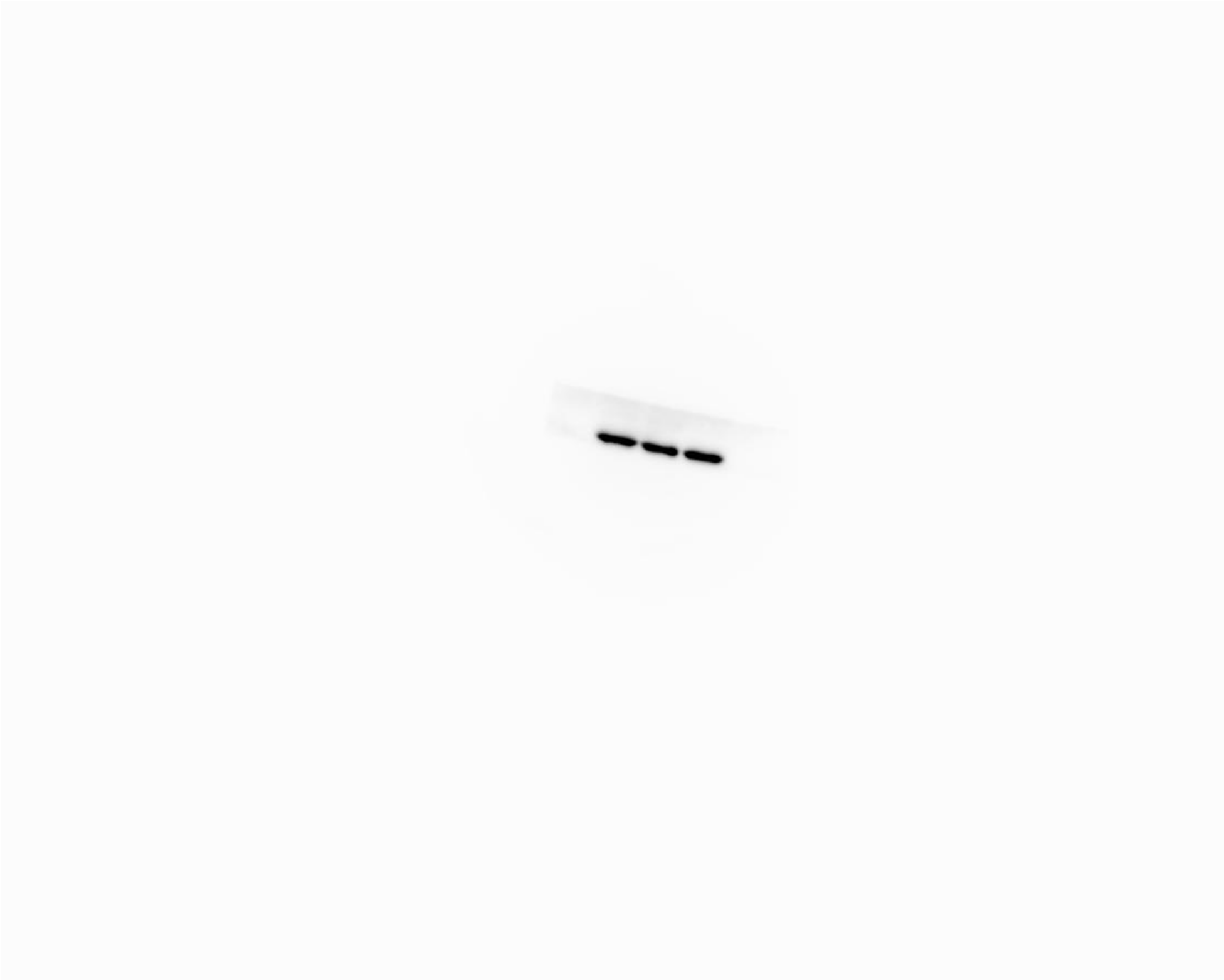

Fig3C

PRMT2

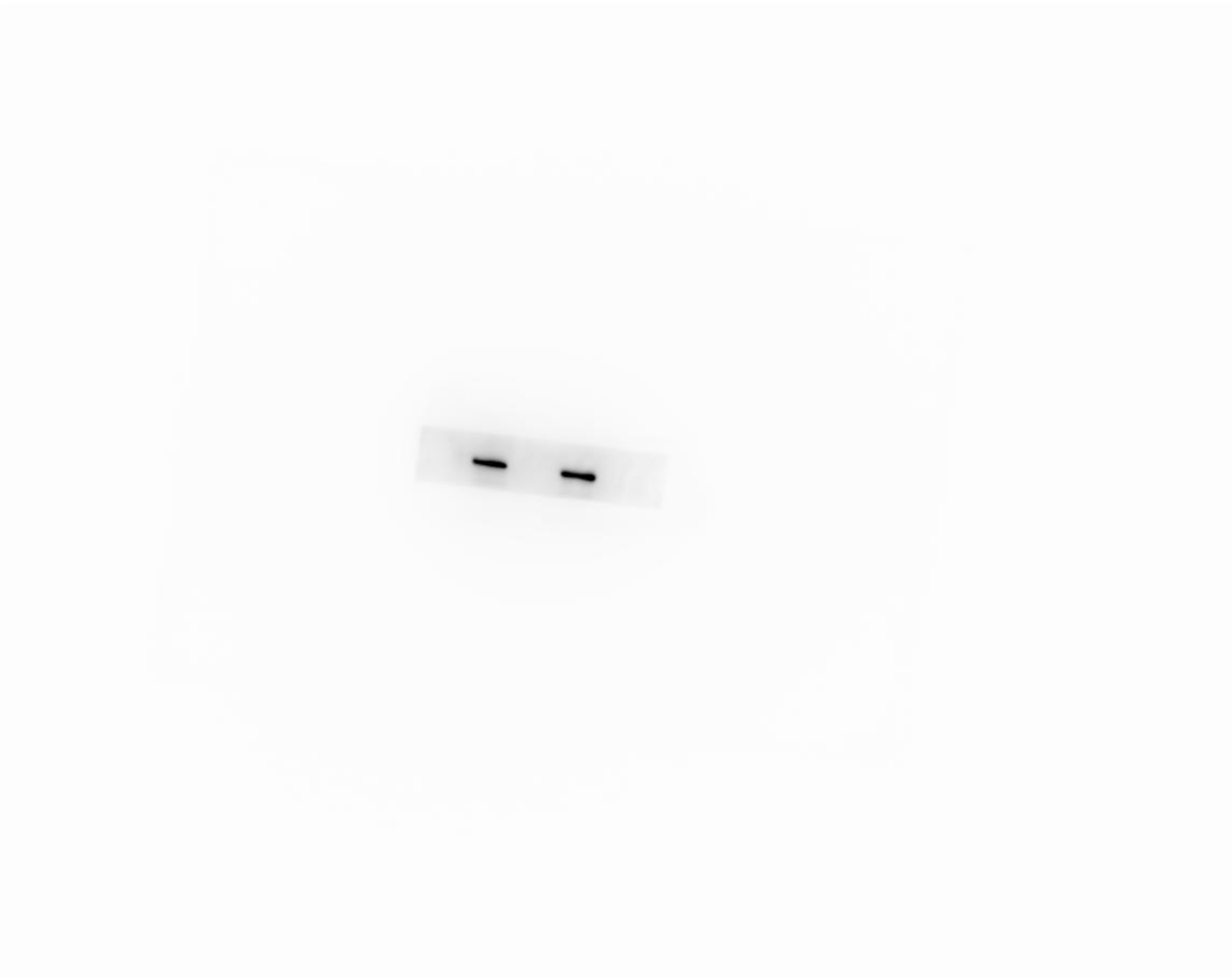

FBXO7

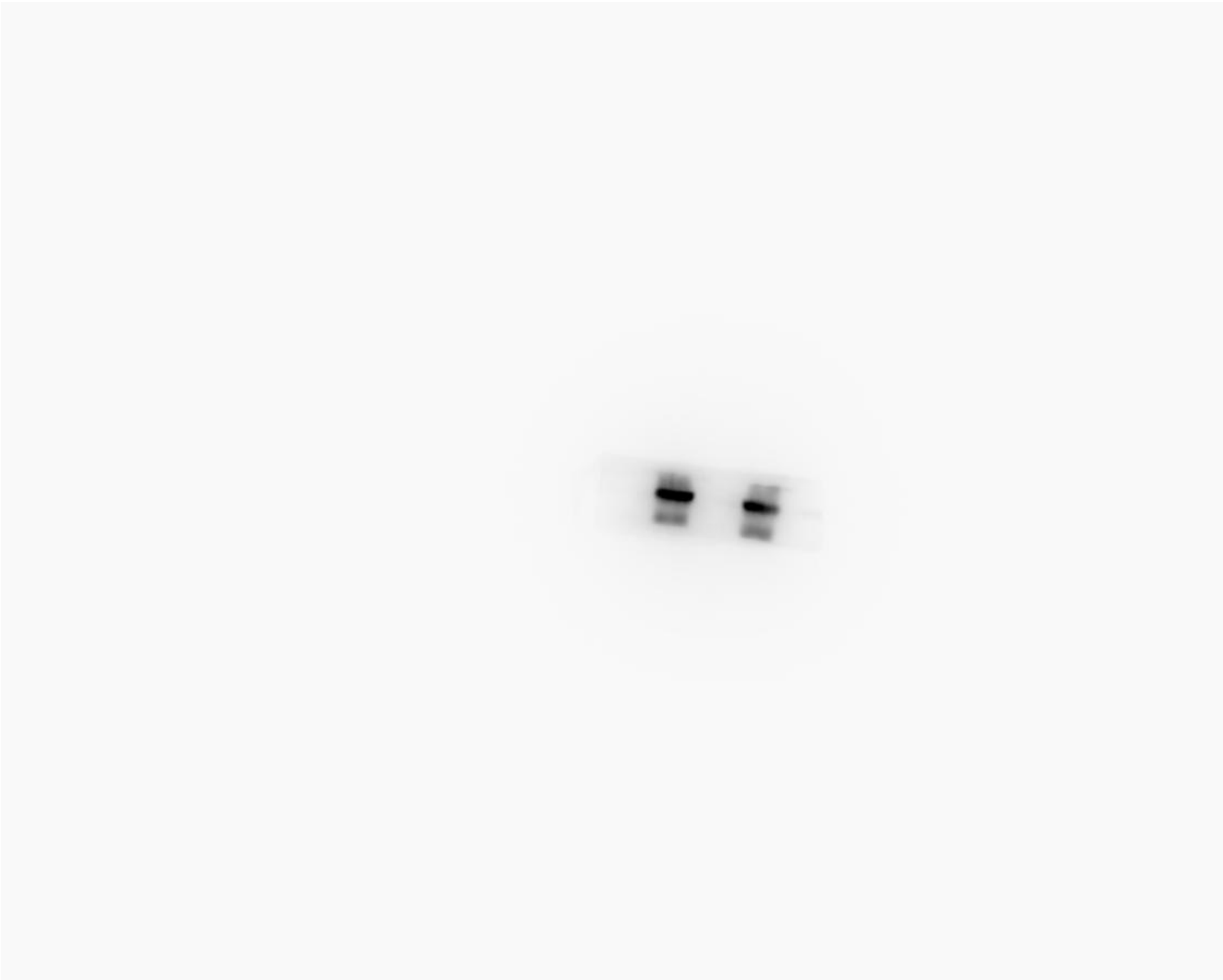

PRMT2

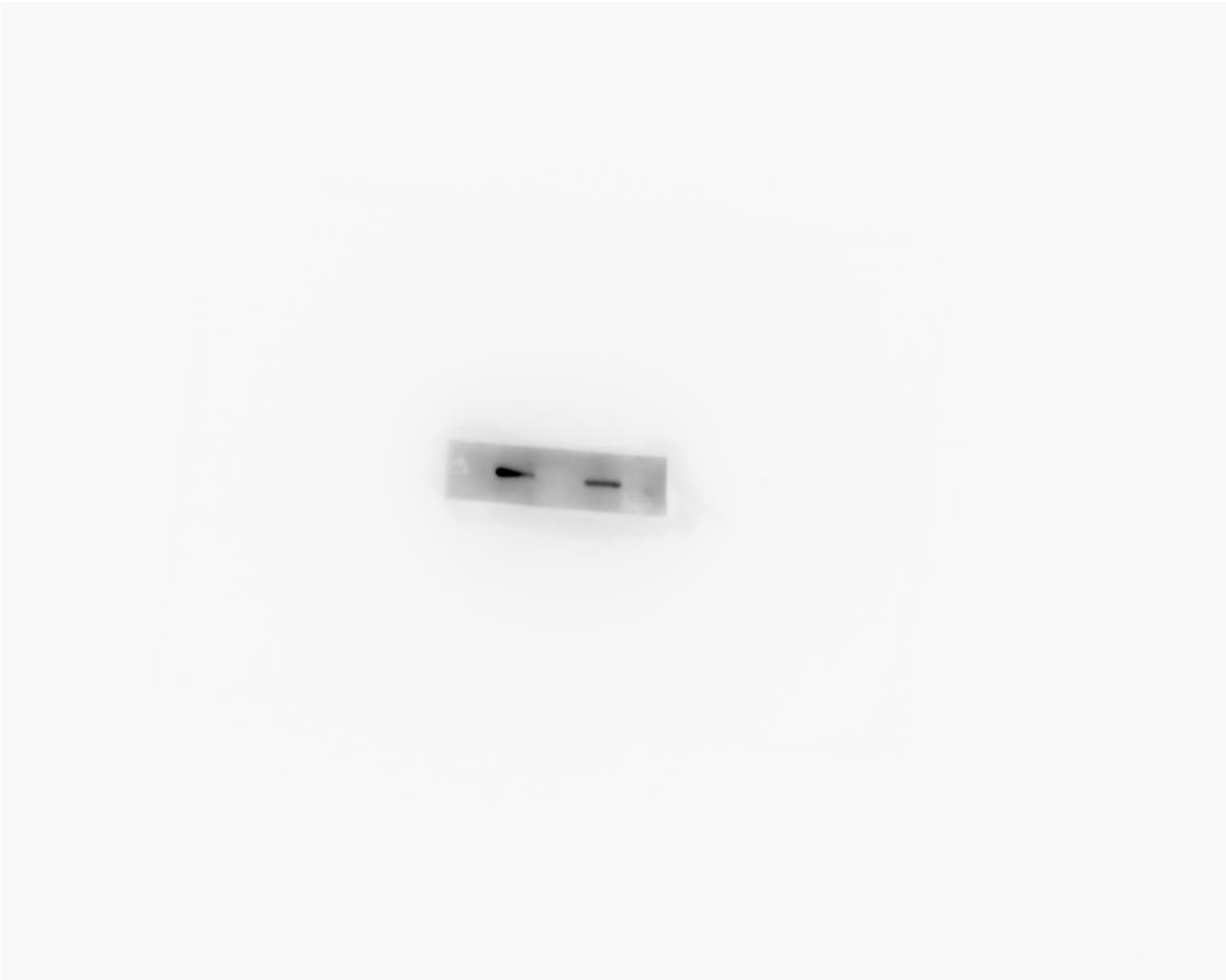

FBXO7

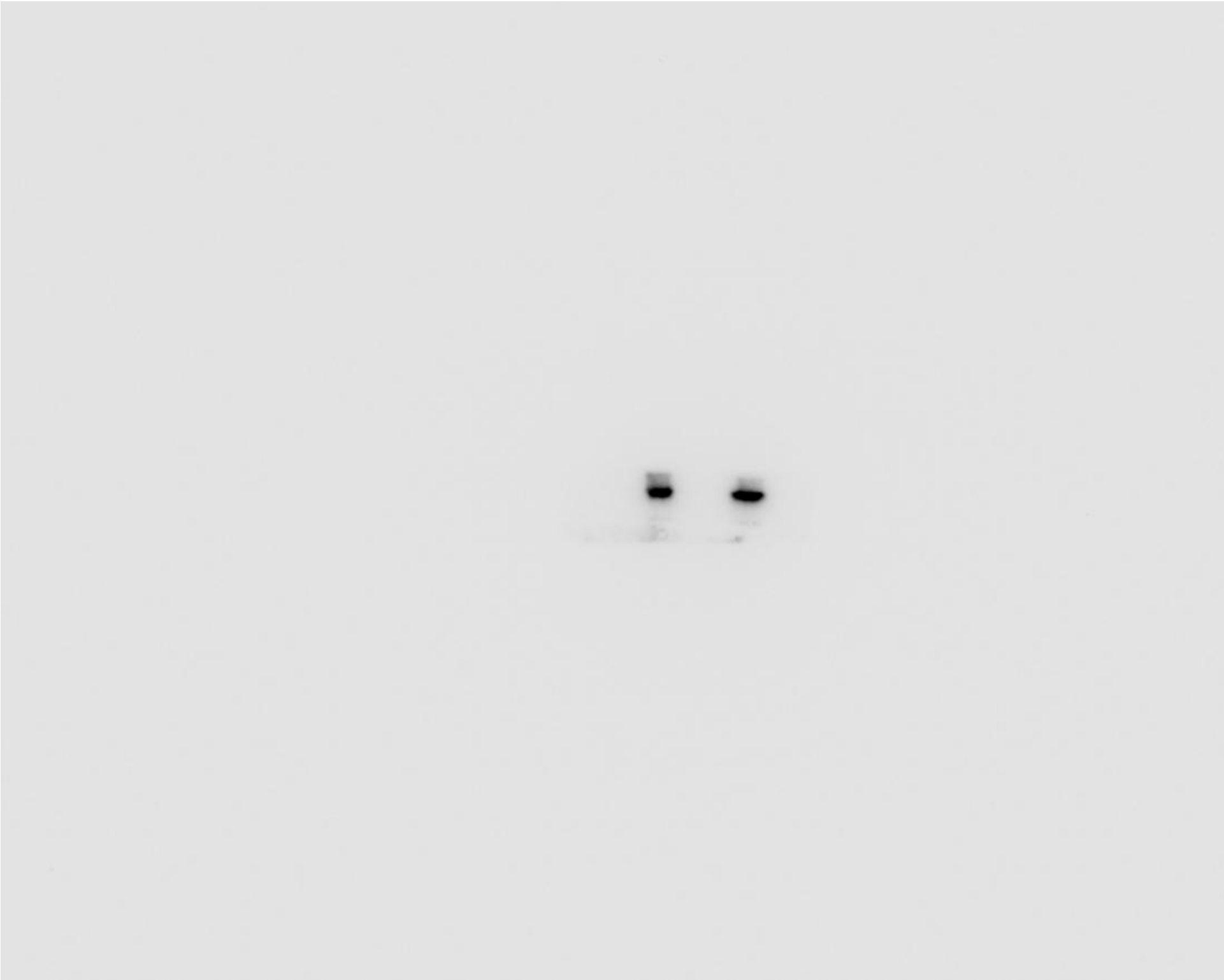

Fig3D

GFP-IP

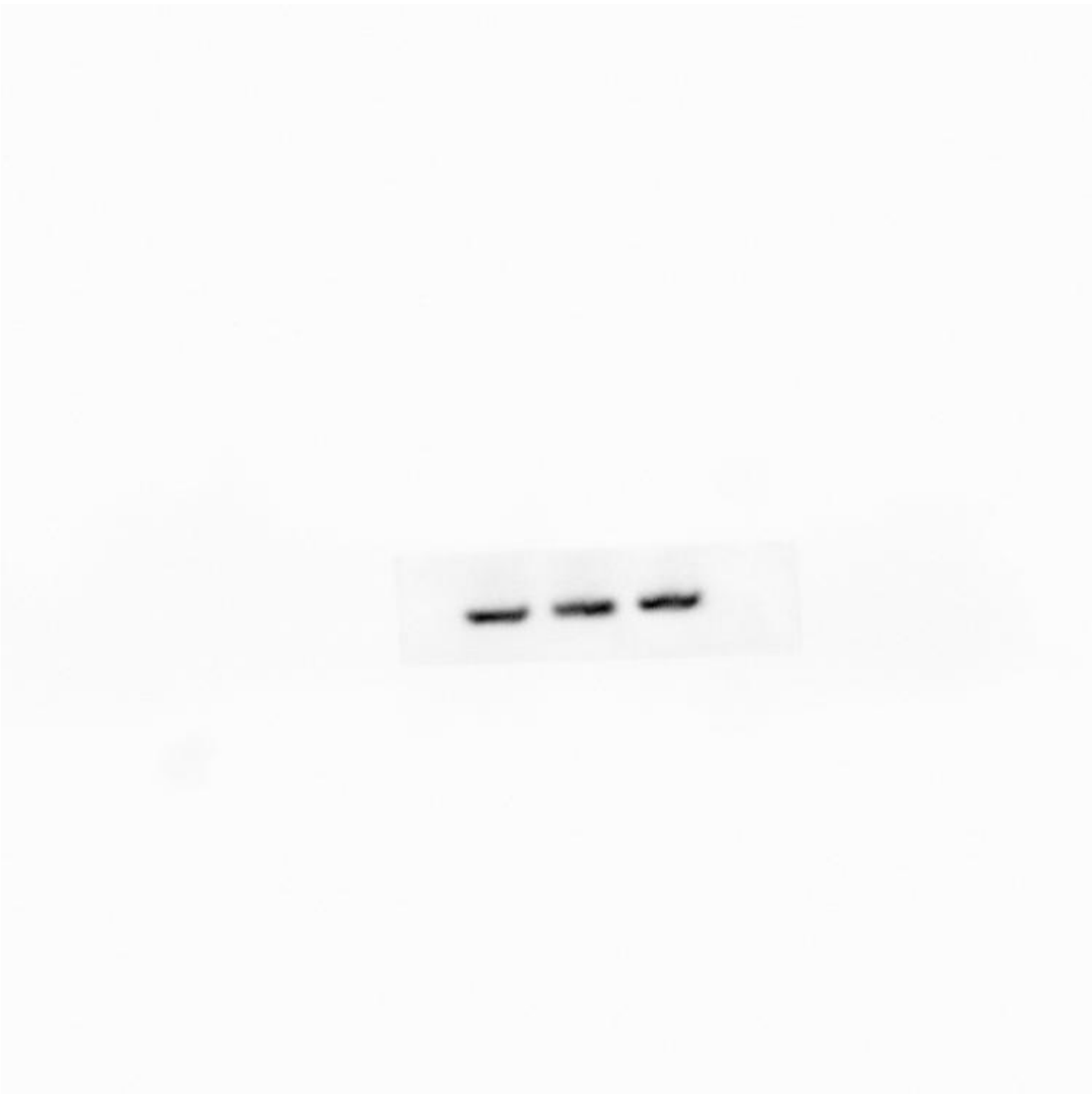

HA-IP

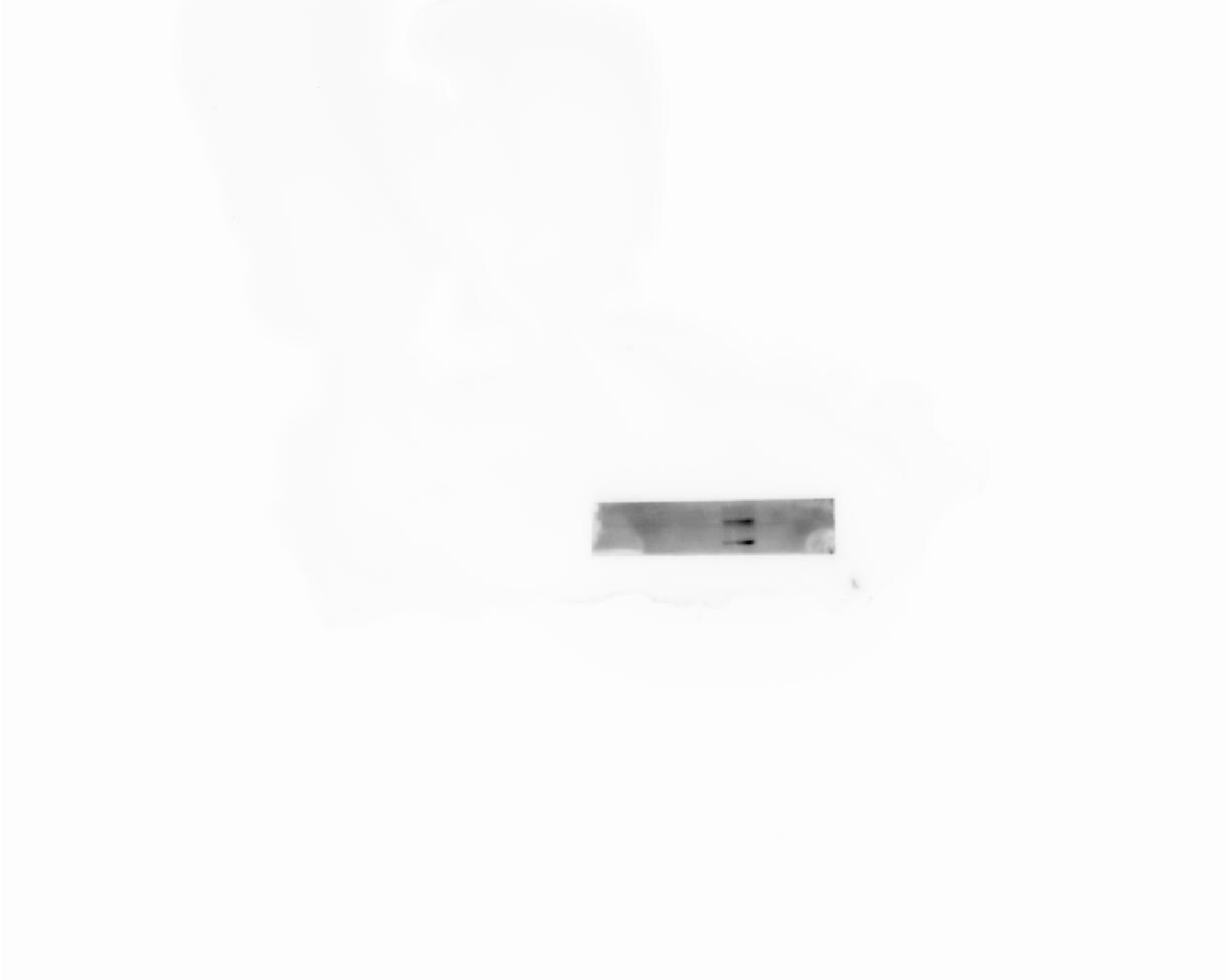

GFP-Input

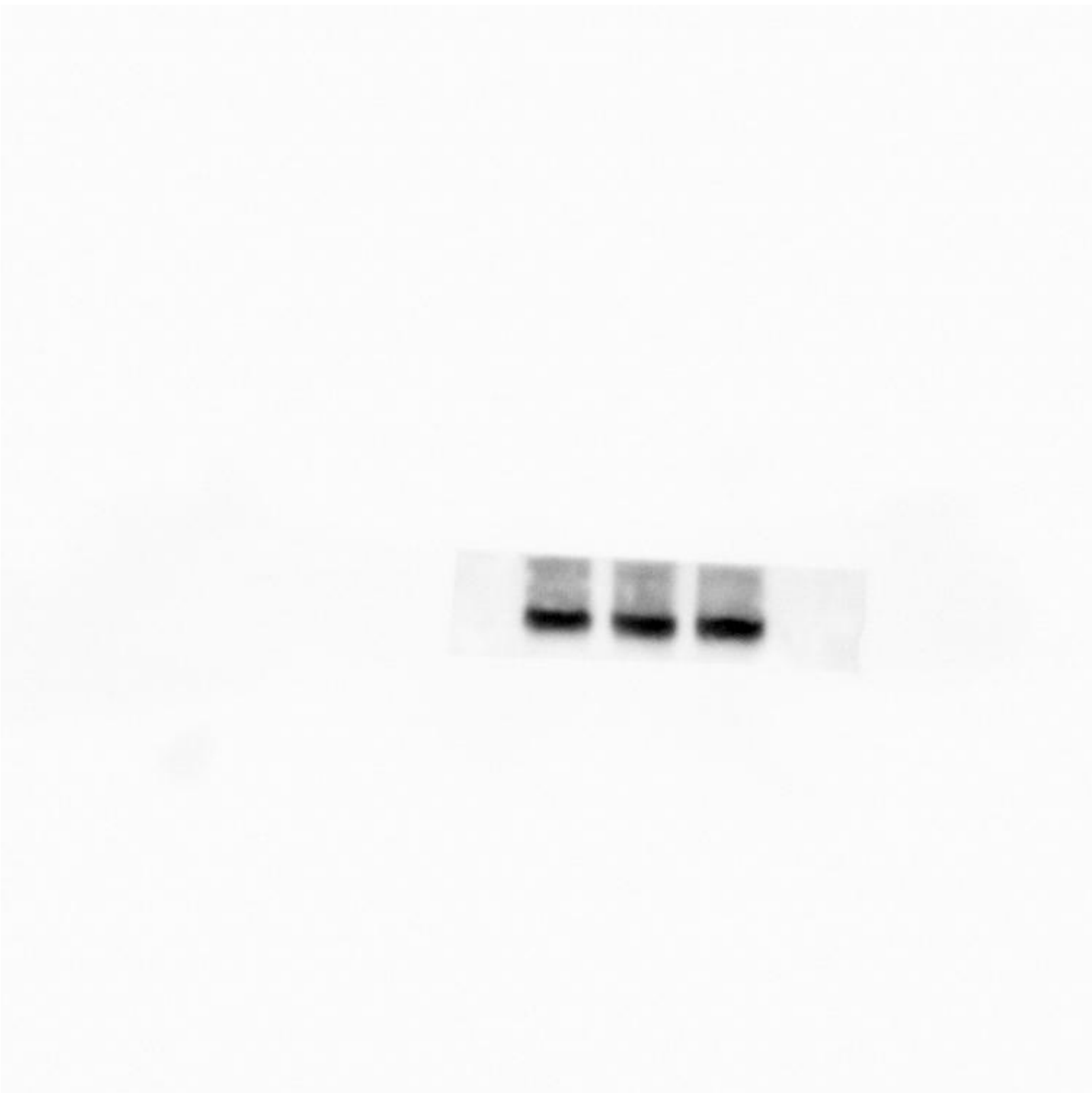

HA-Input

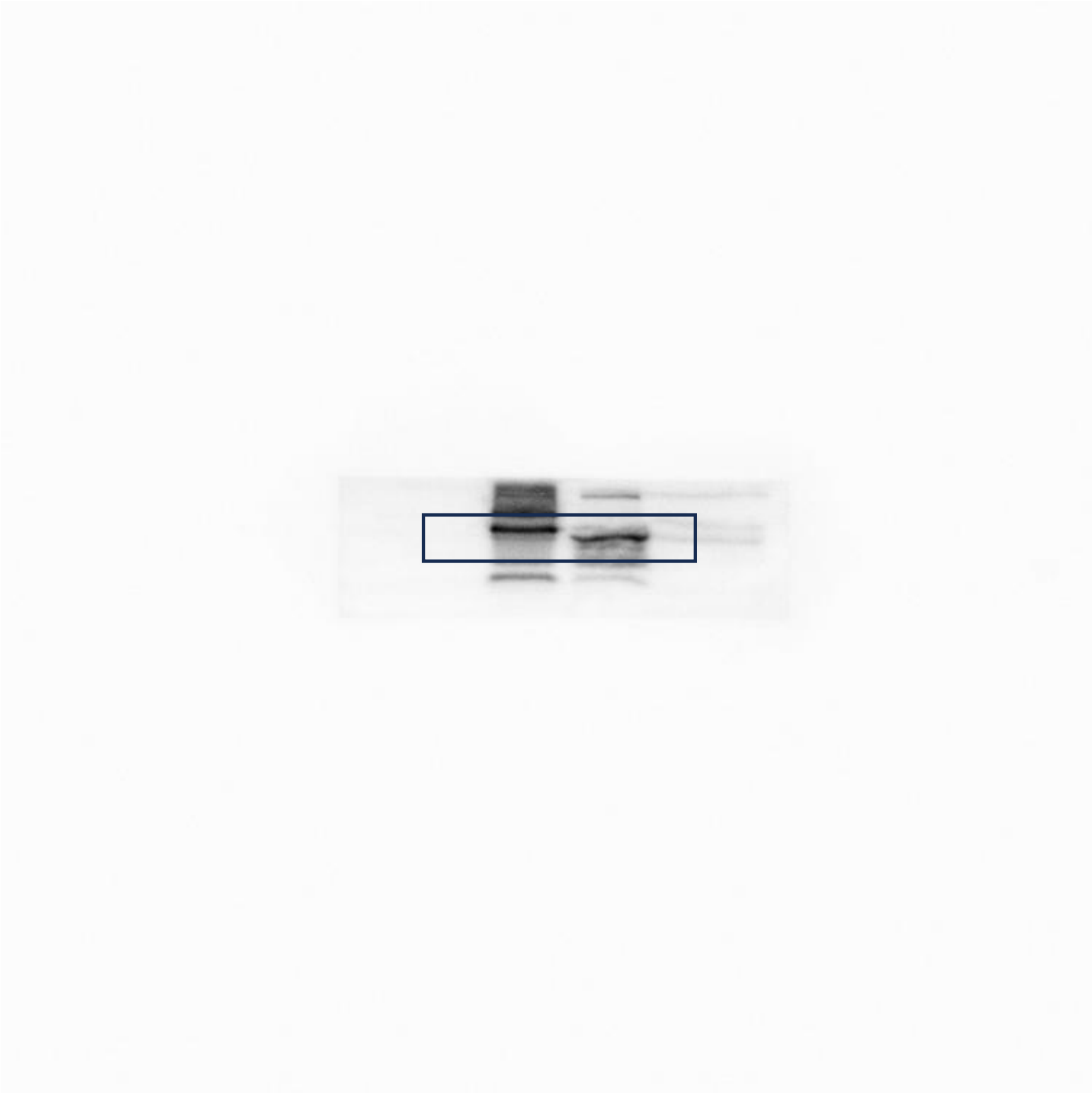

GAPDH-Input

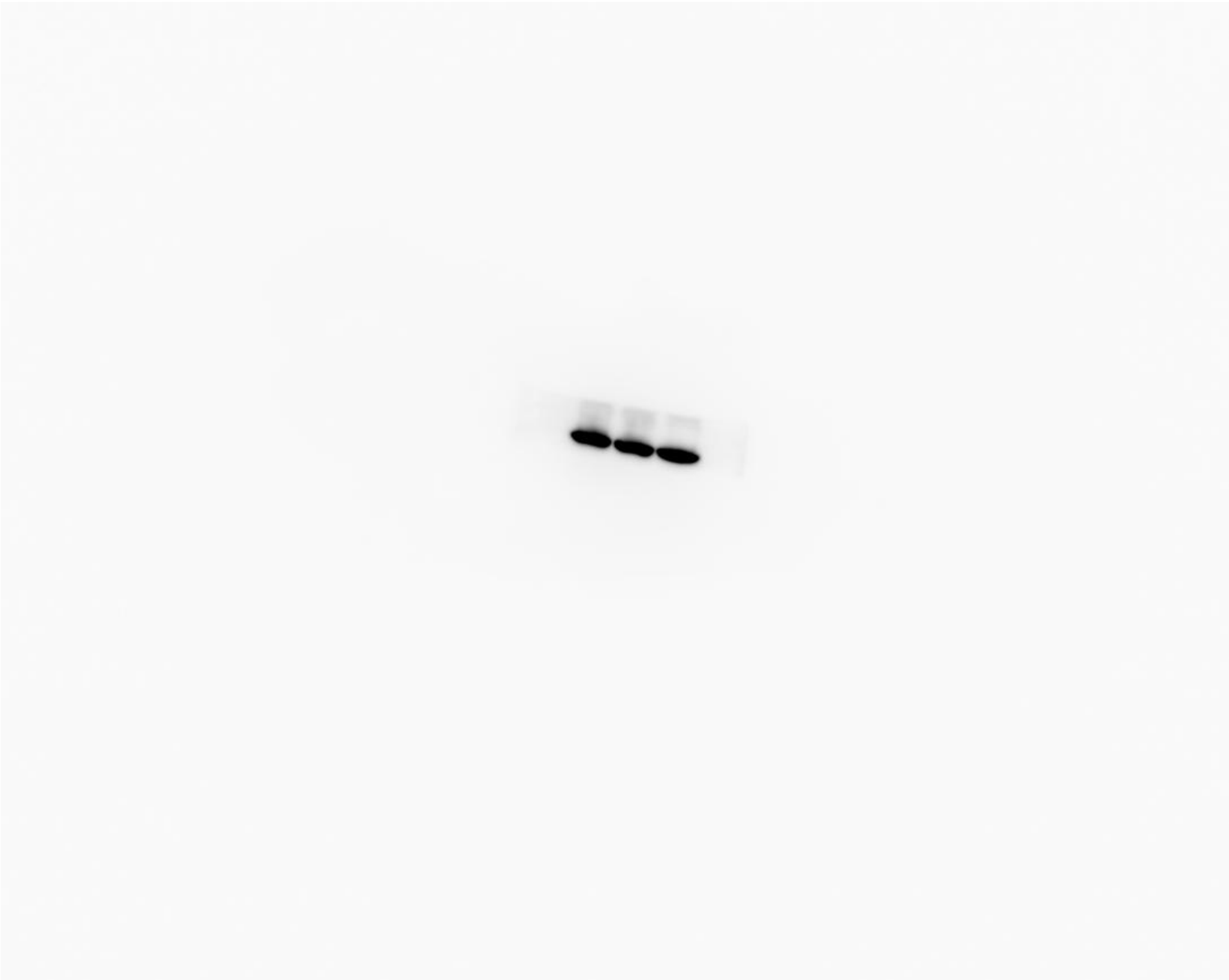

Fig3E

MMA-IP

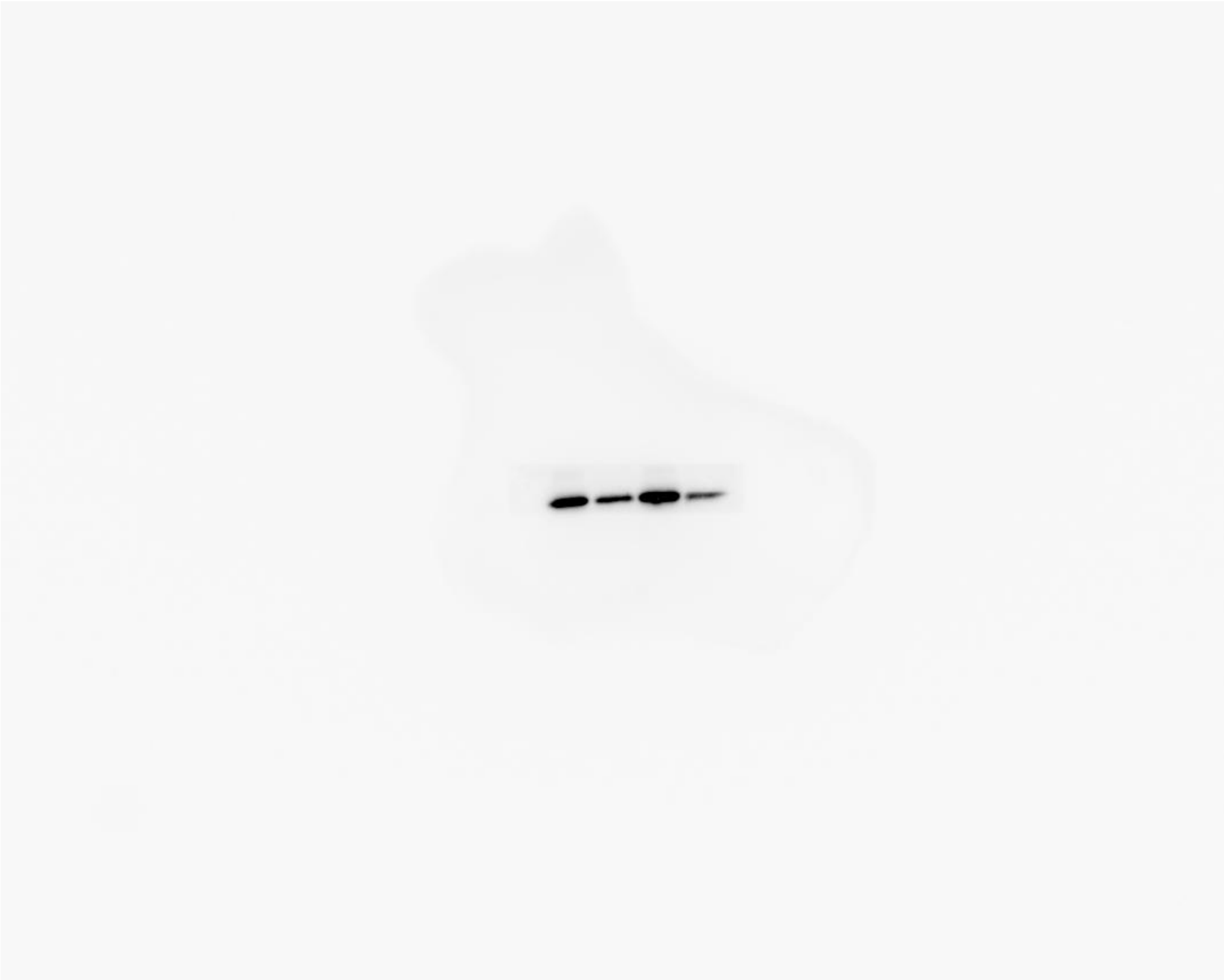

GFP-IP

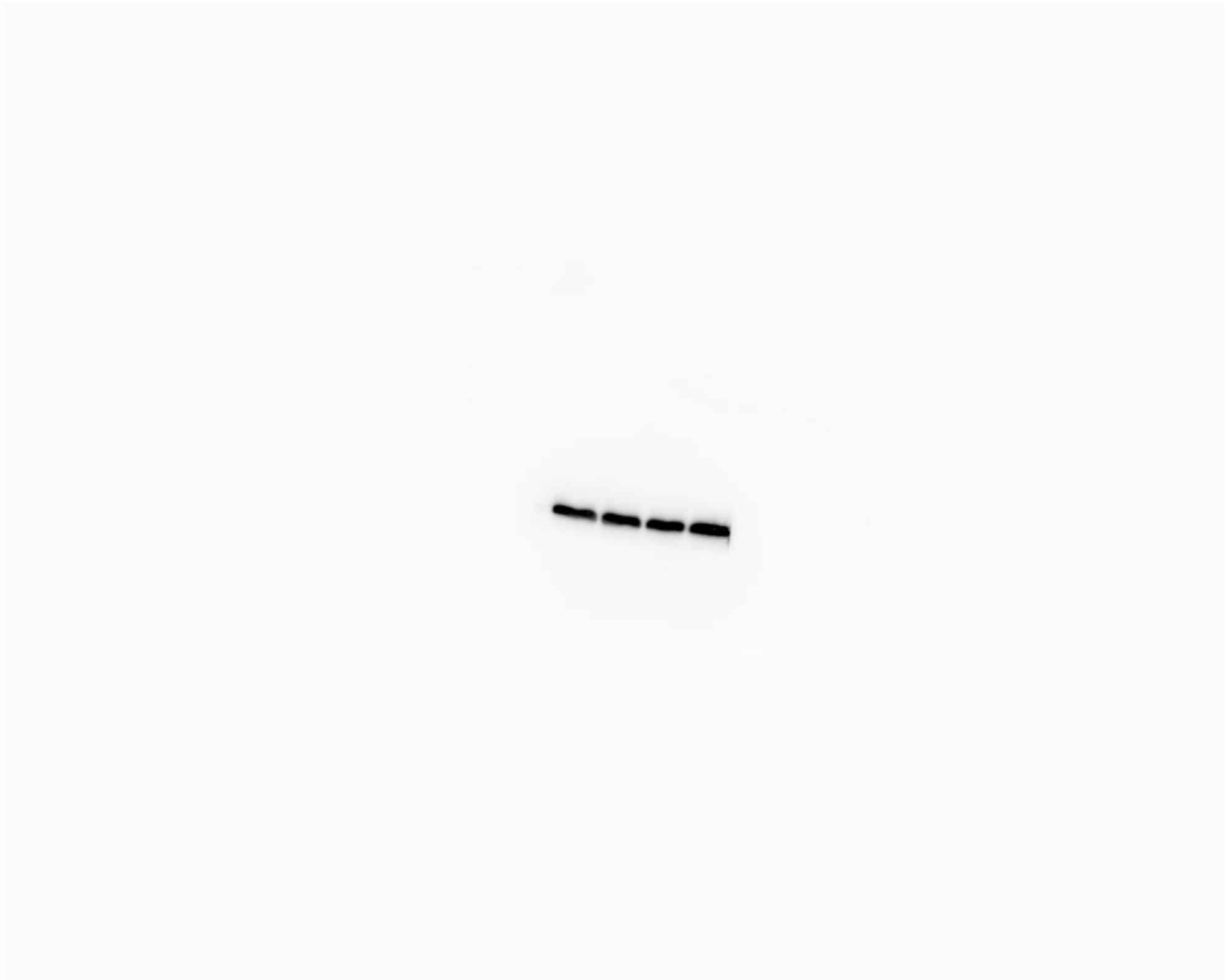

GFP-Input

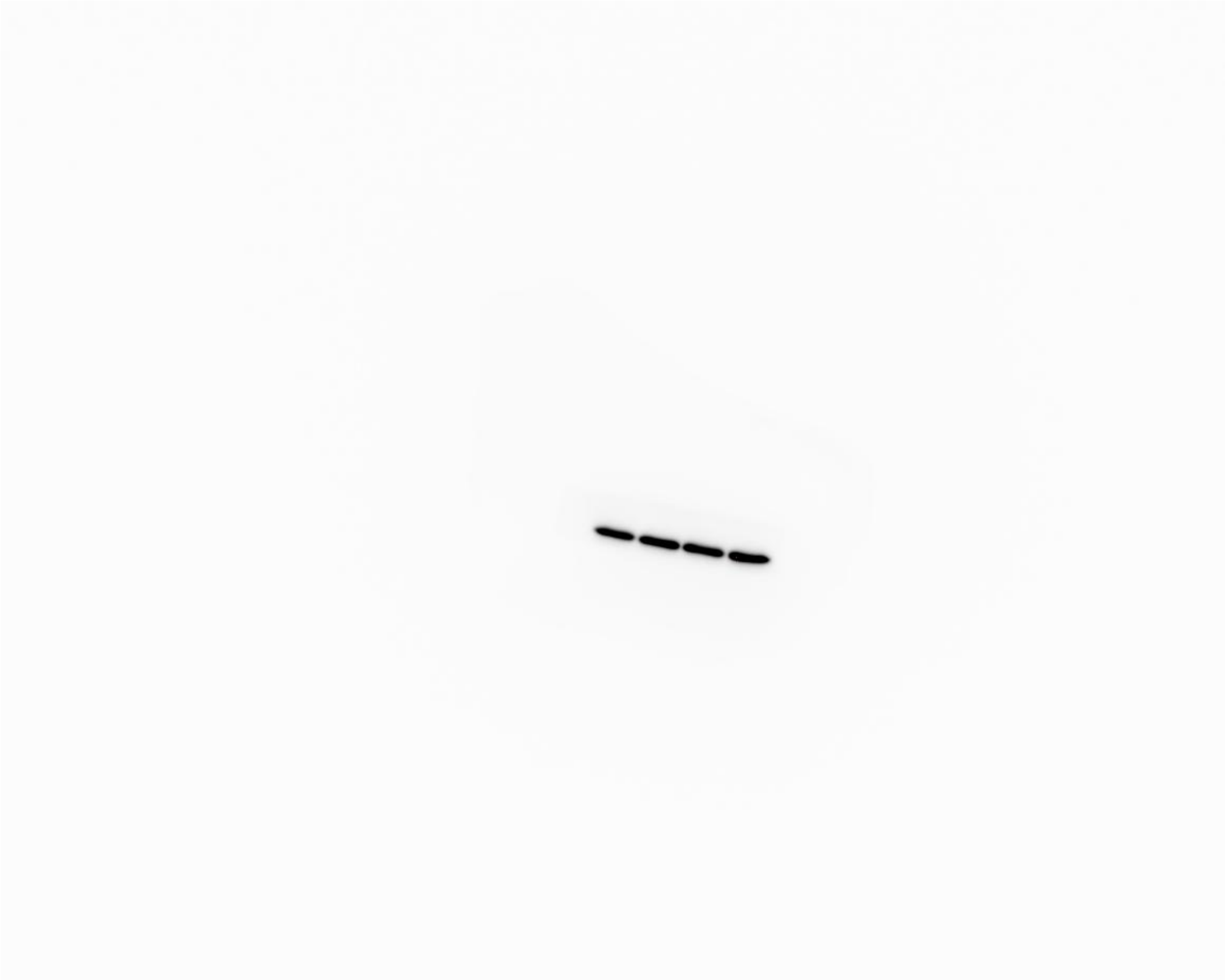

ACTIN-Input

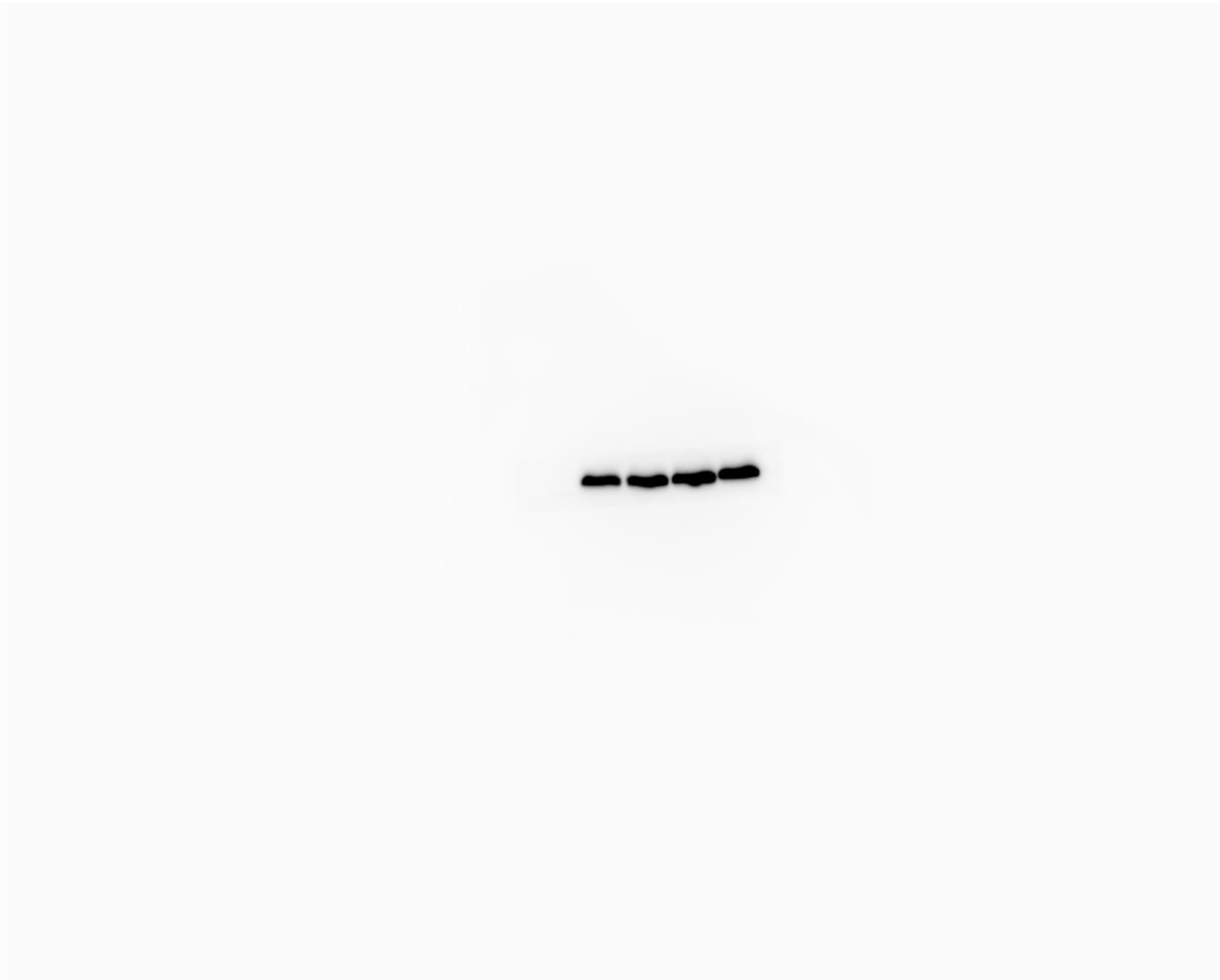

Fig3F

MMA-IP

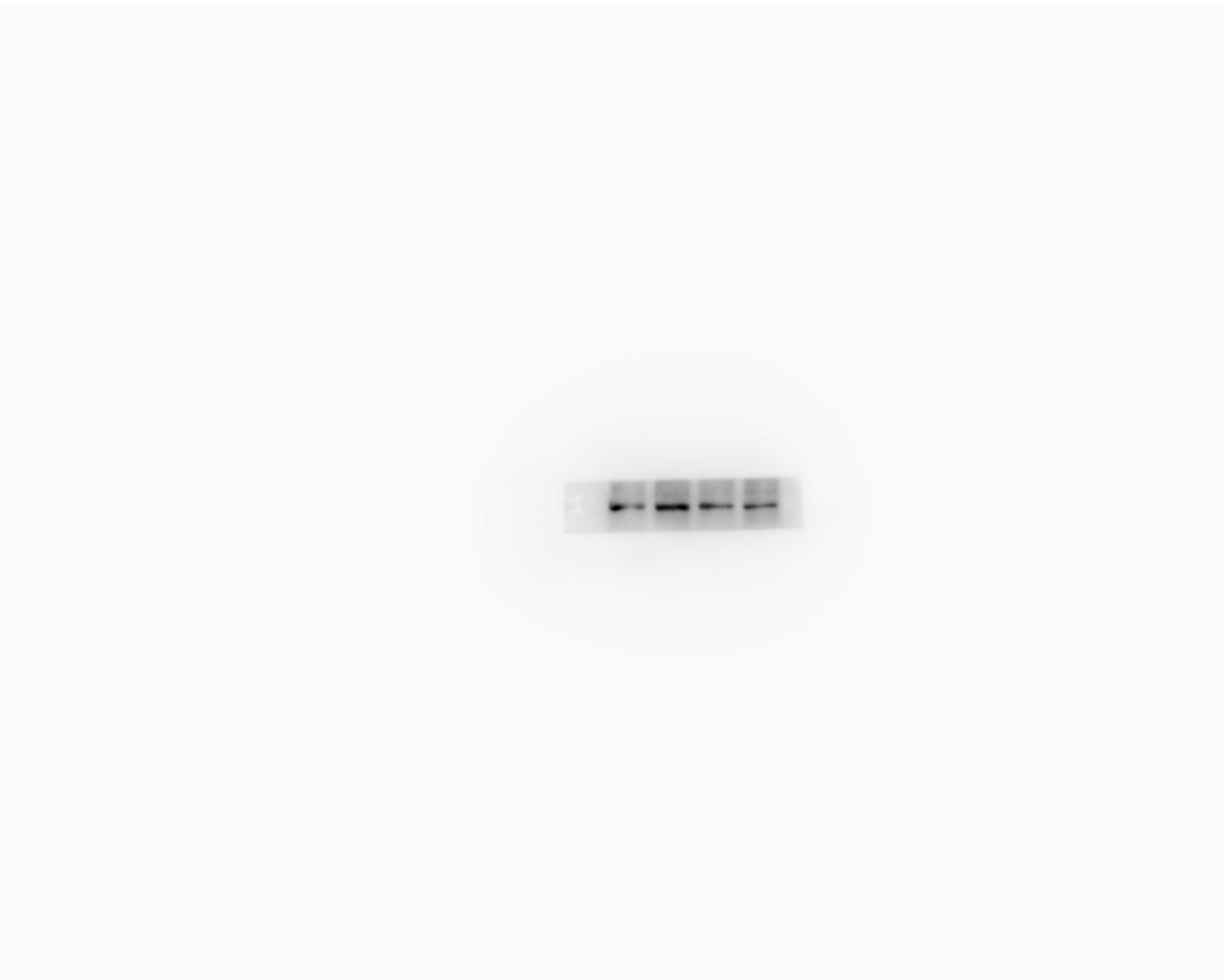

GFP-IP

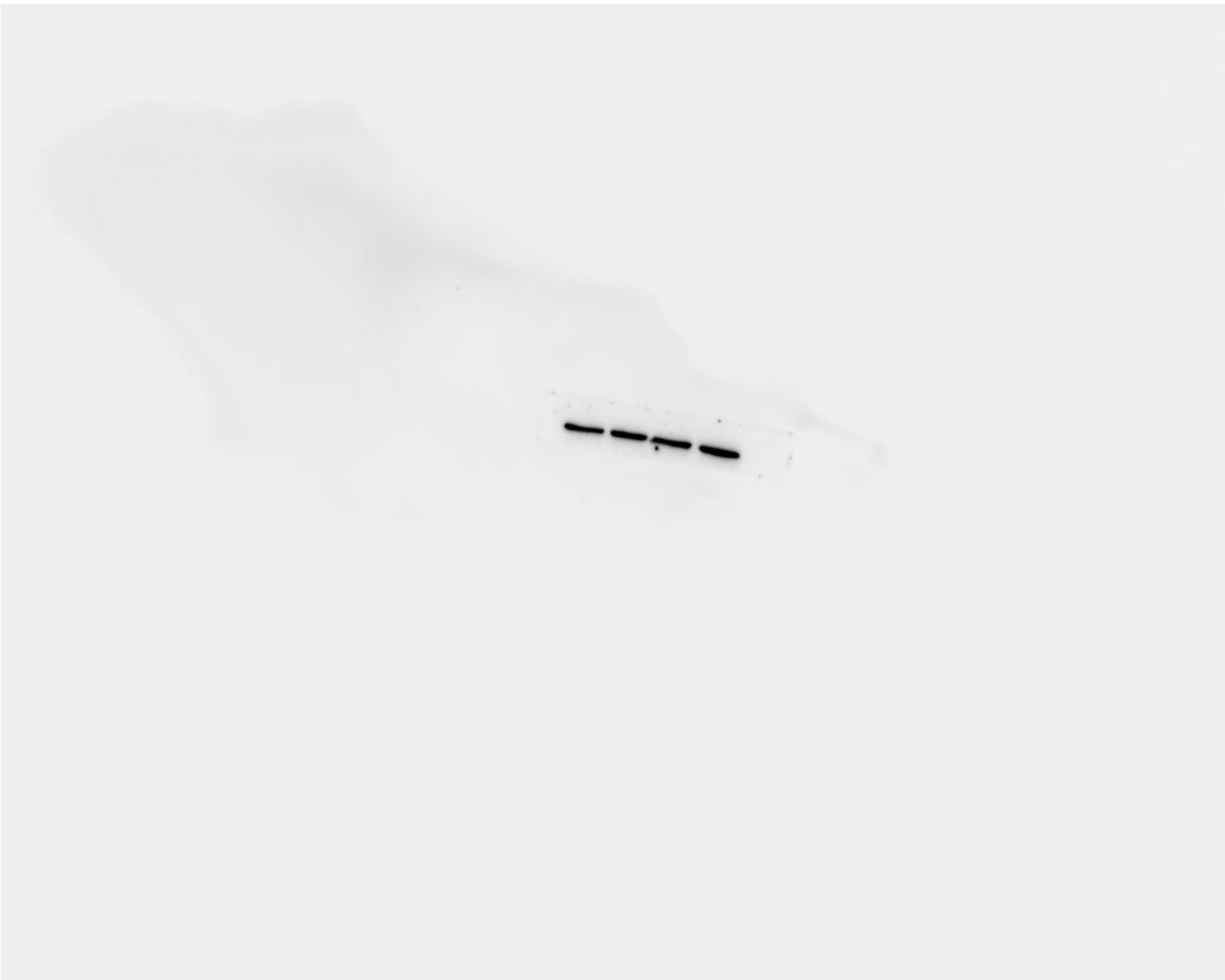

HA-Input

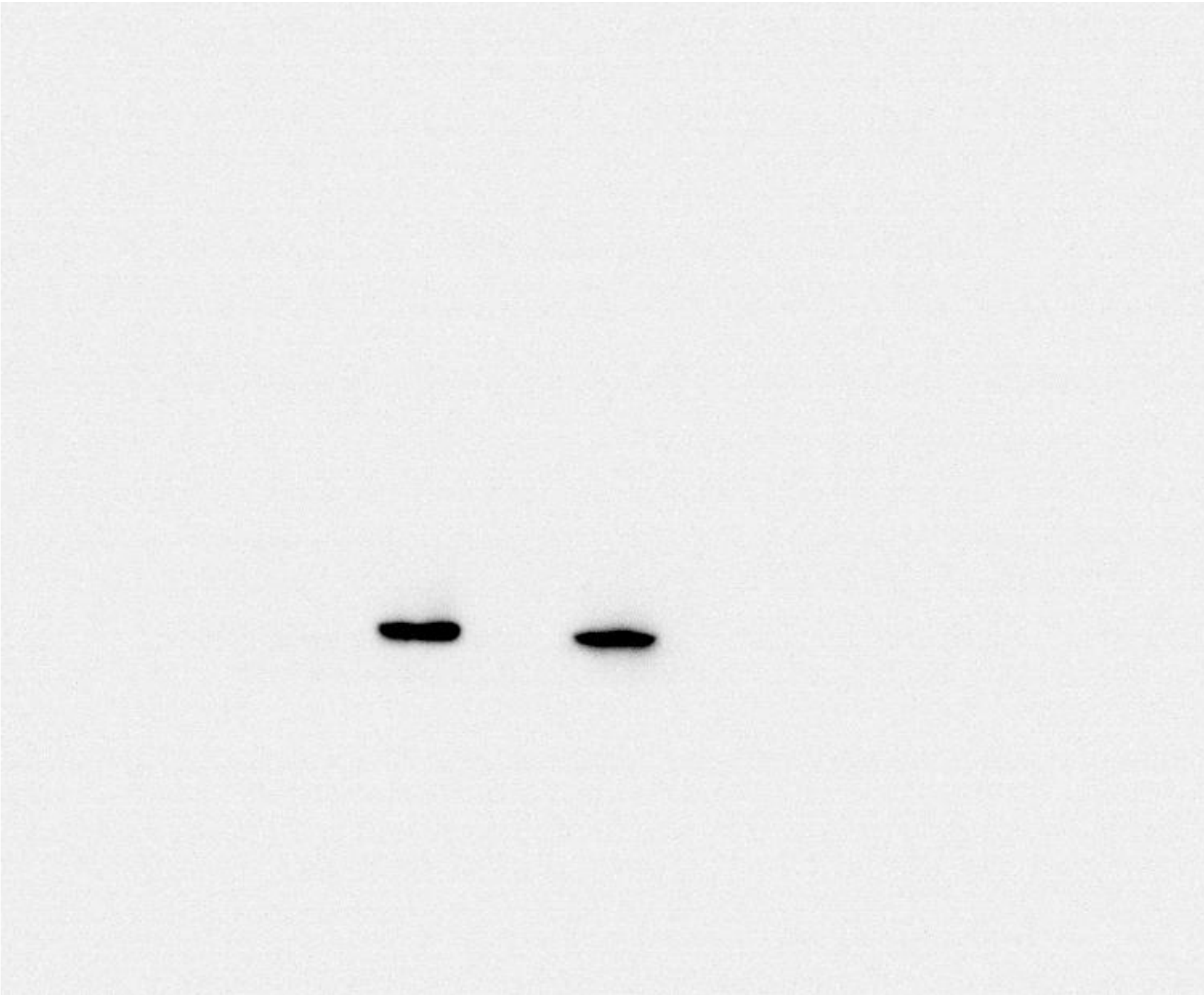

GFP-Input

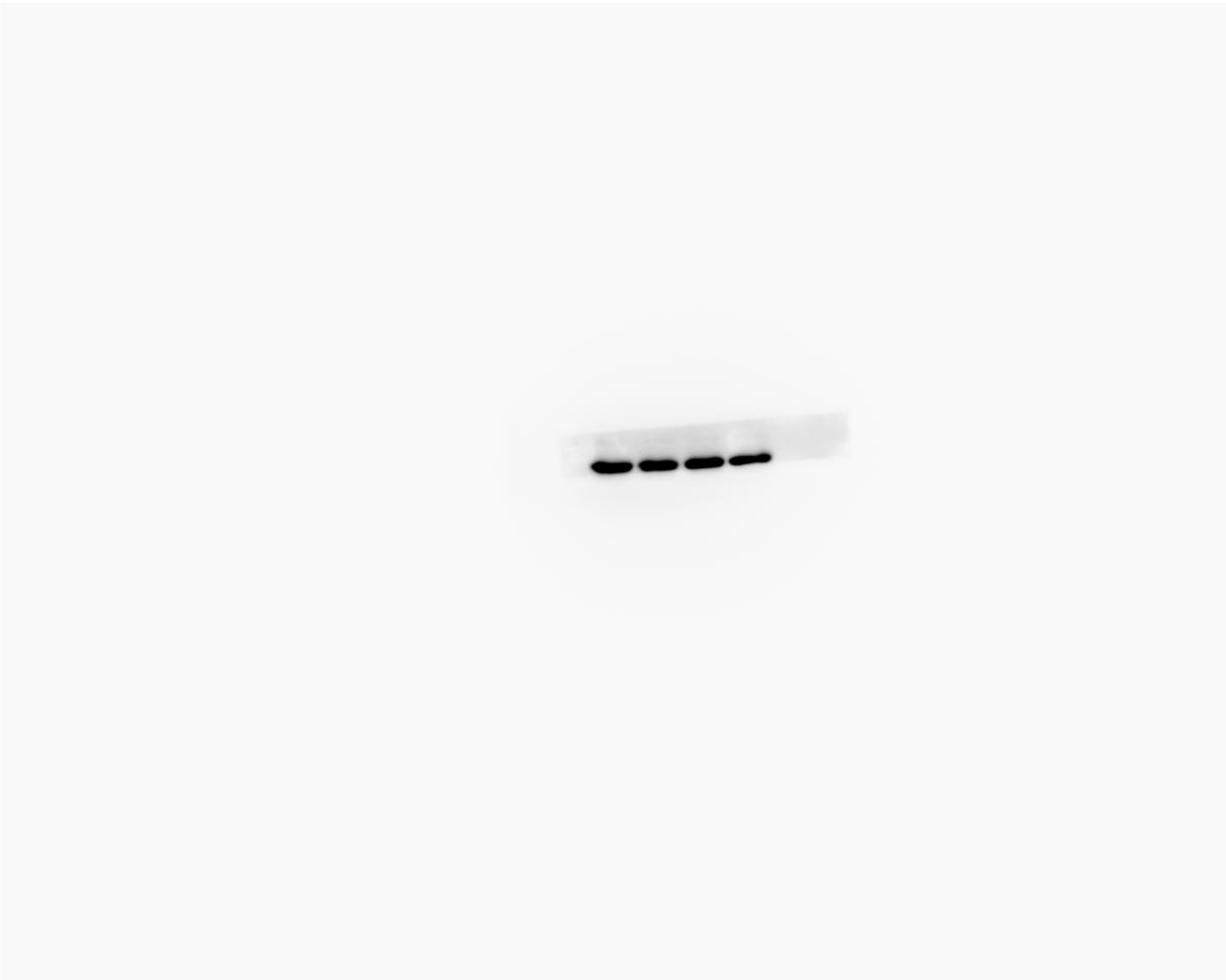

ACTIN-Input

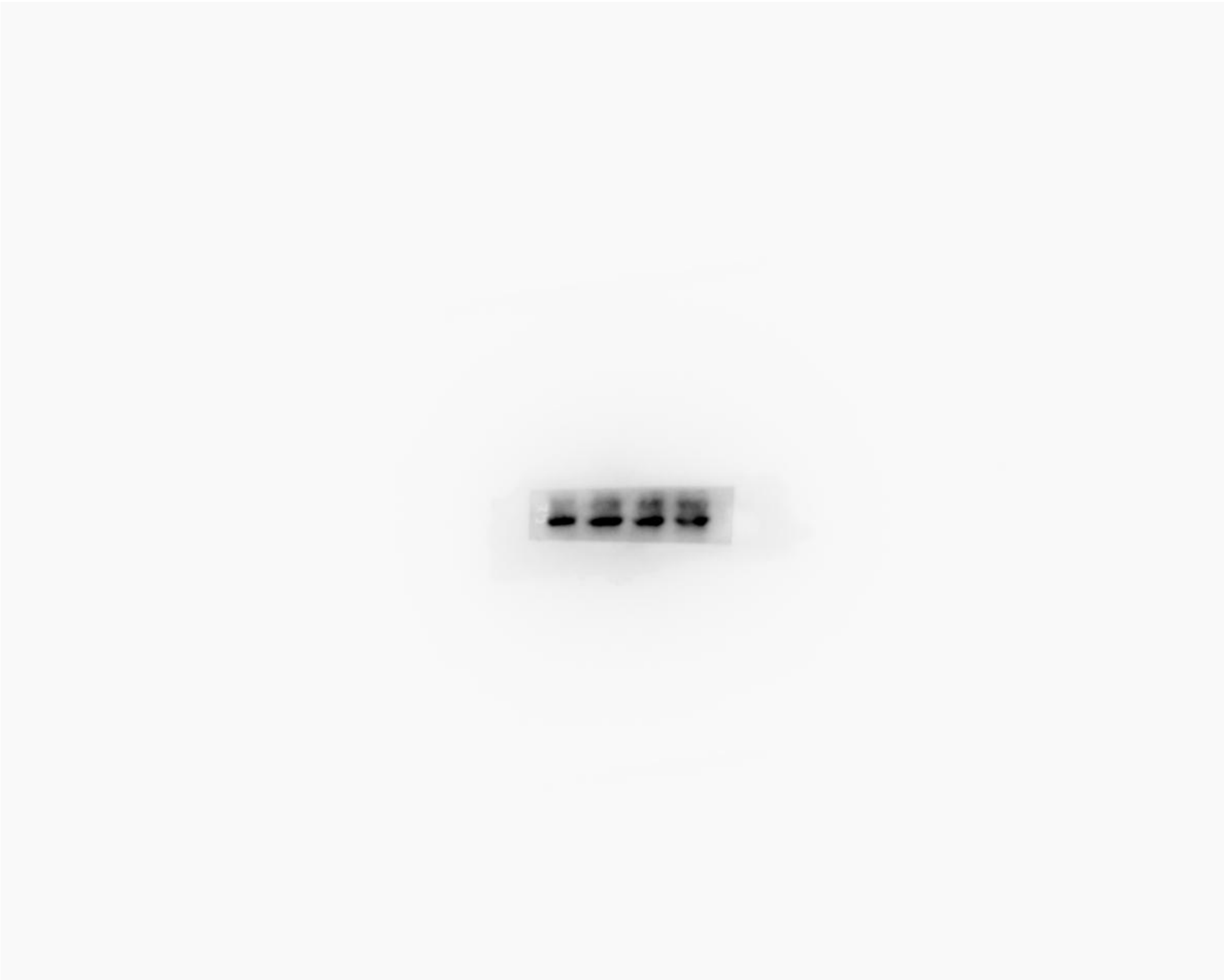

Fig4B

MED12

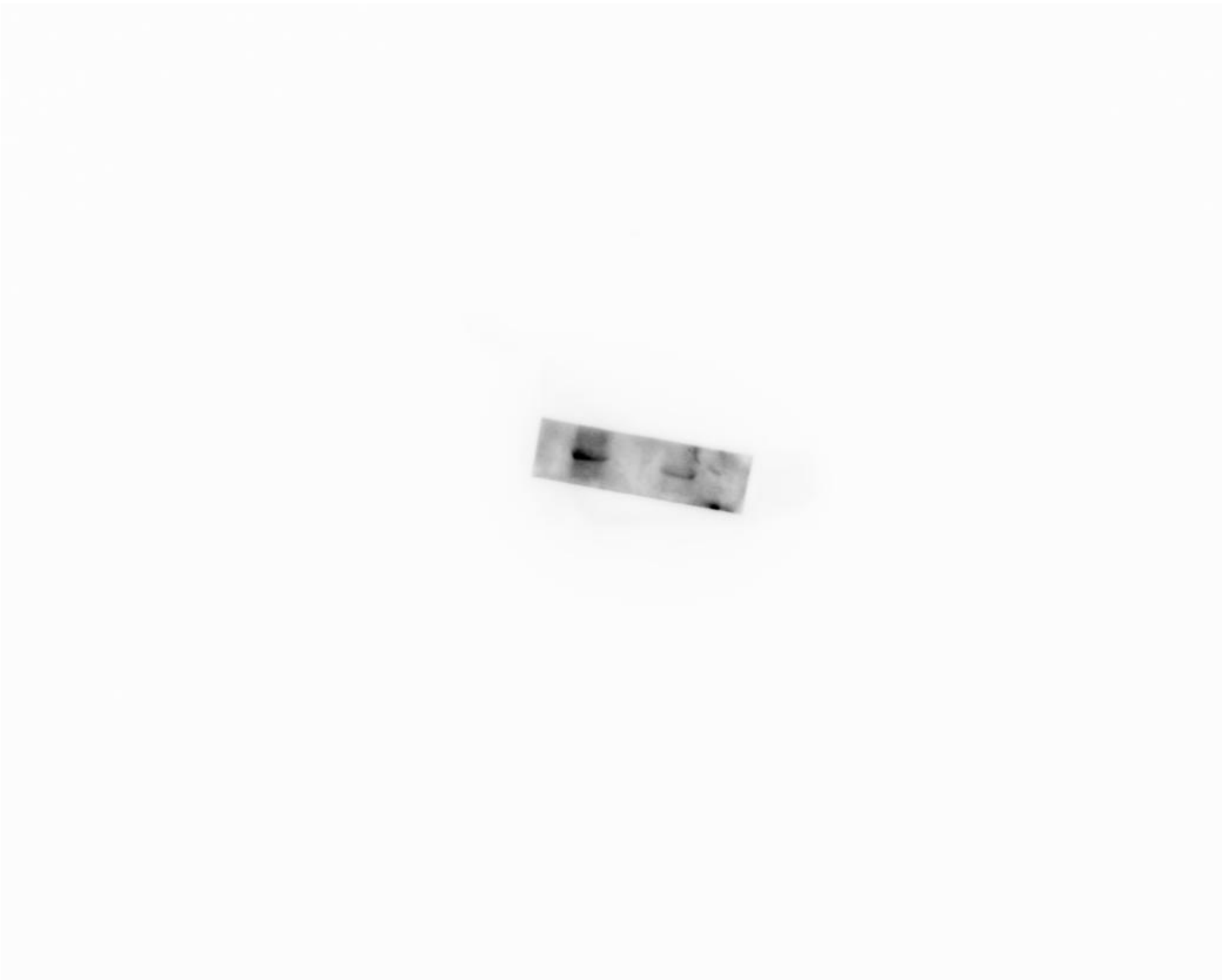

FBXO7

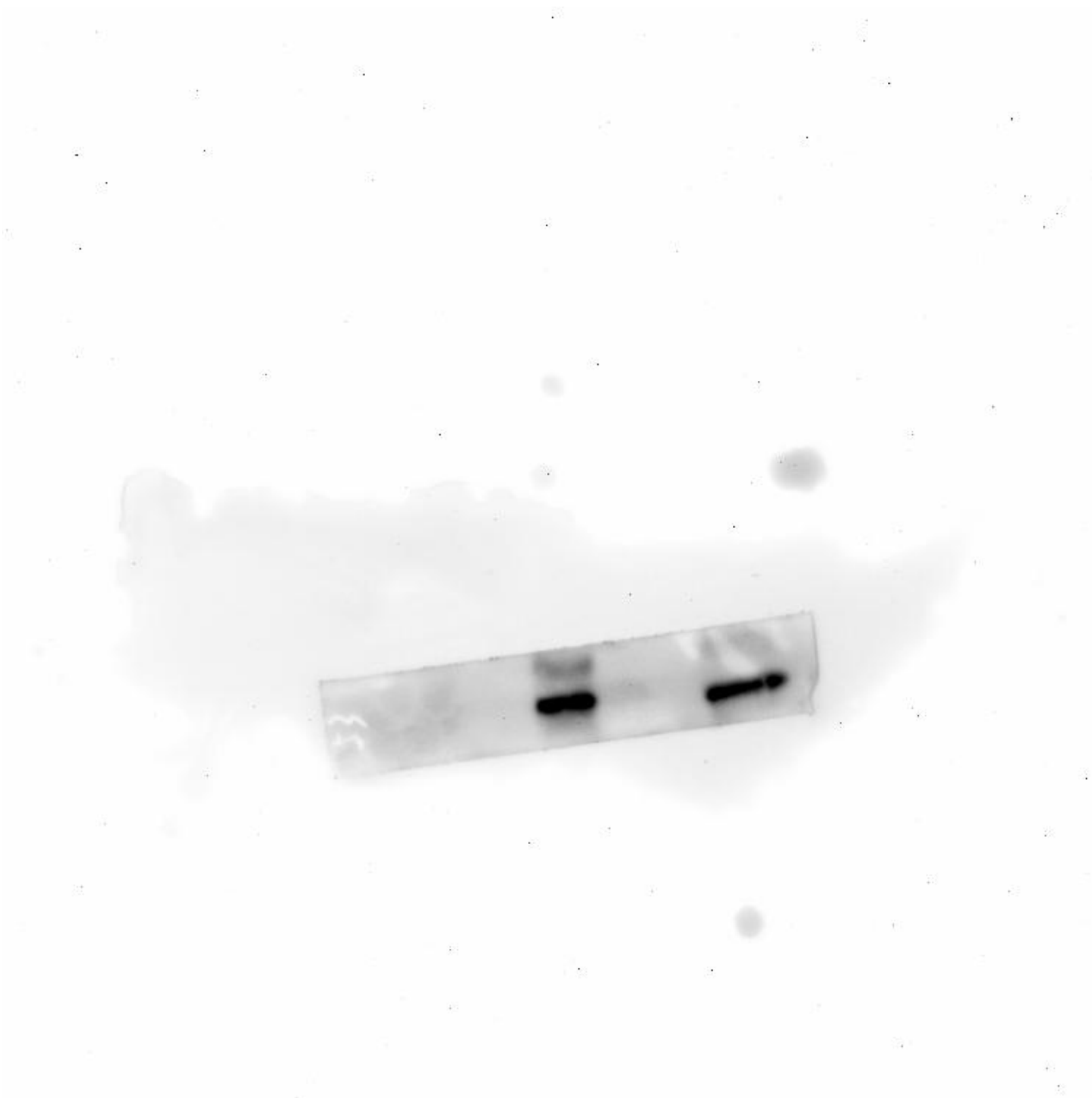

MED12

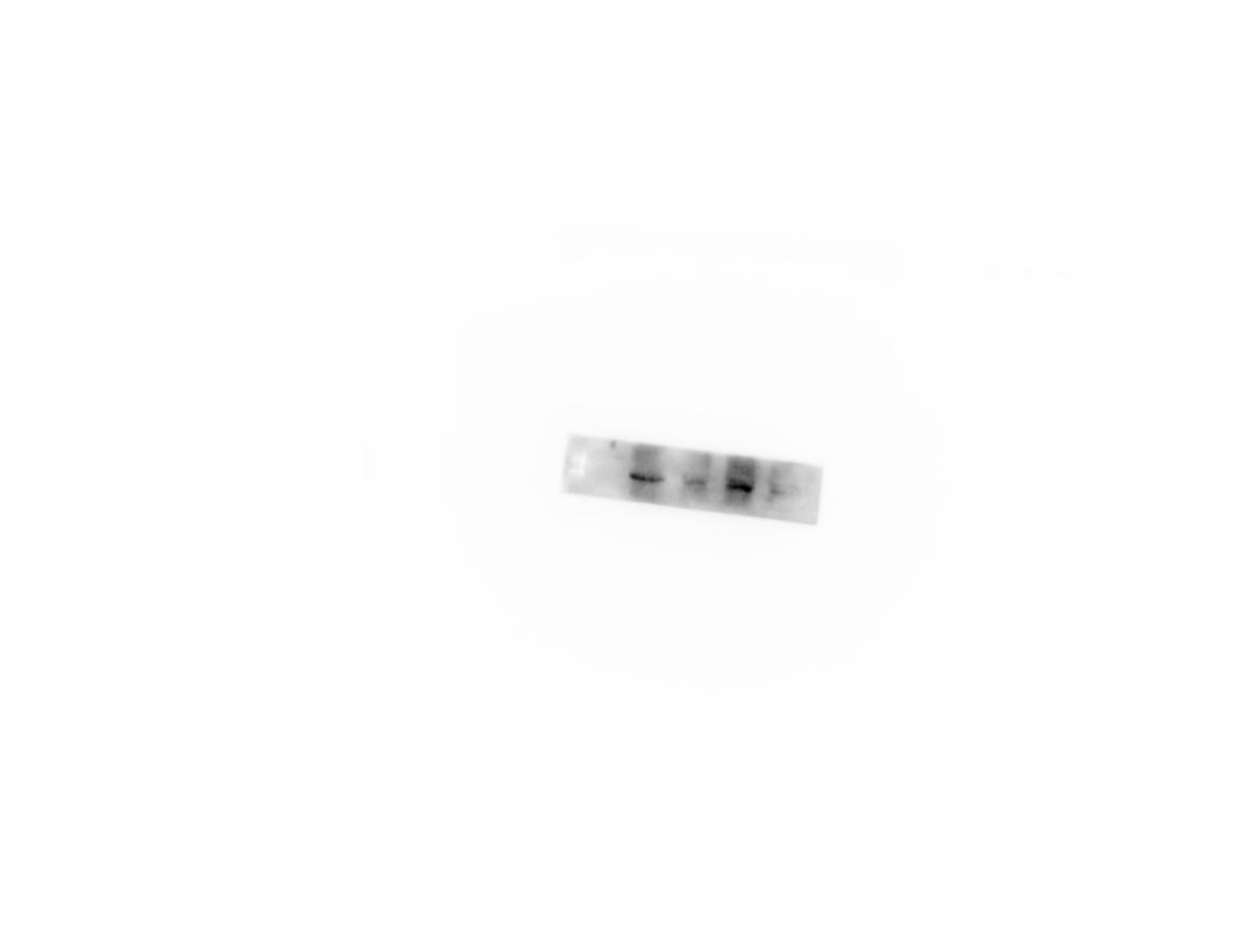

FBXO7

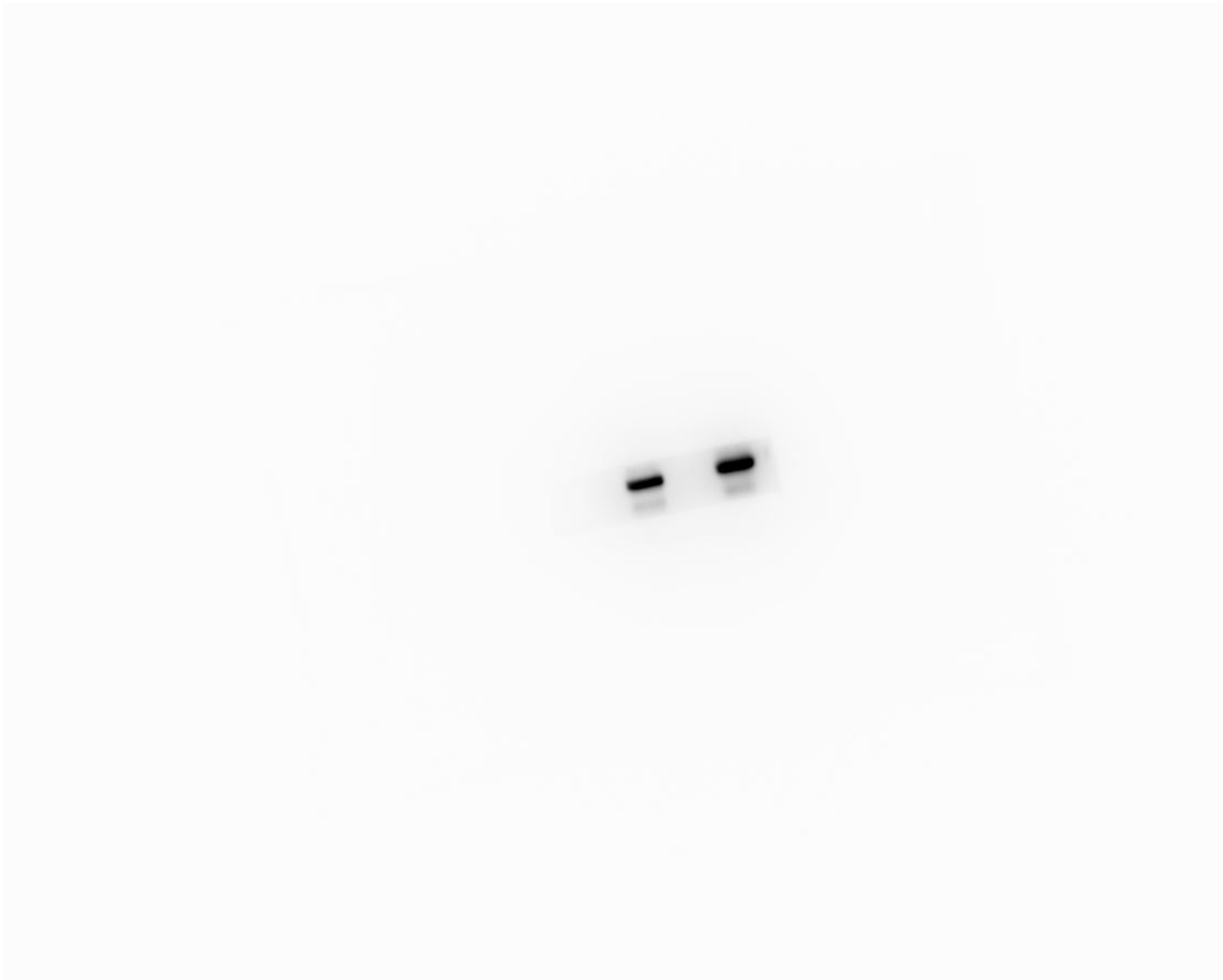

Fig4C

Flag-IP

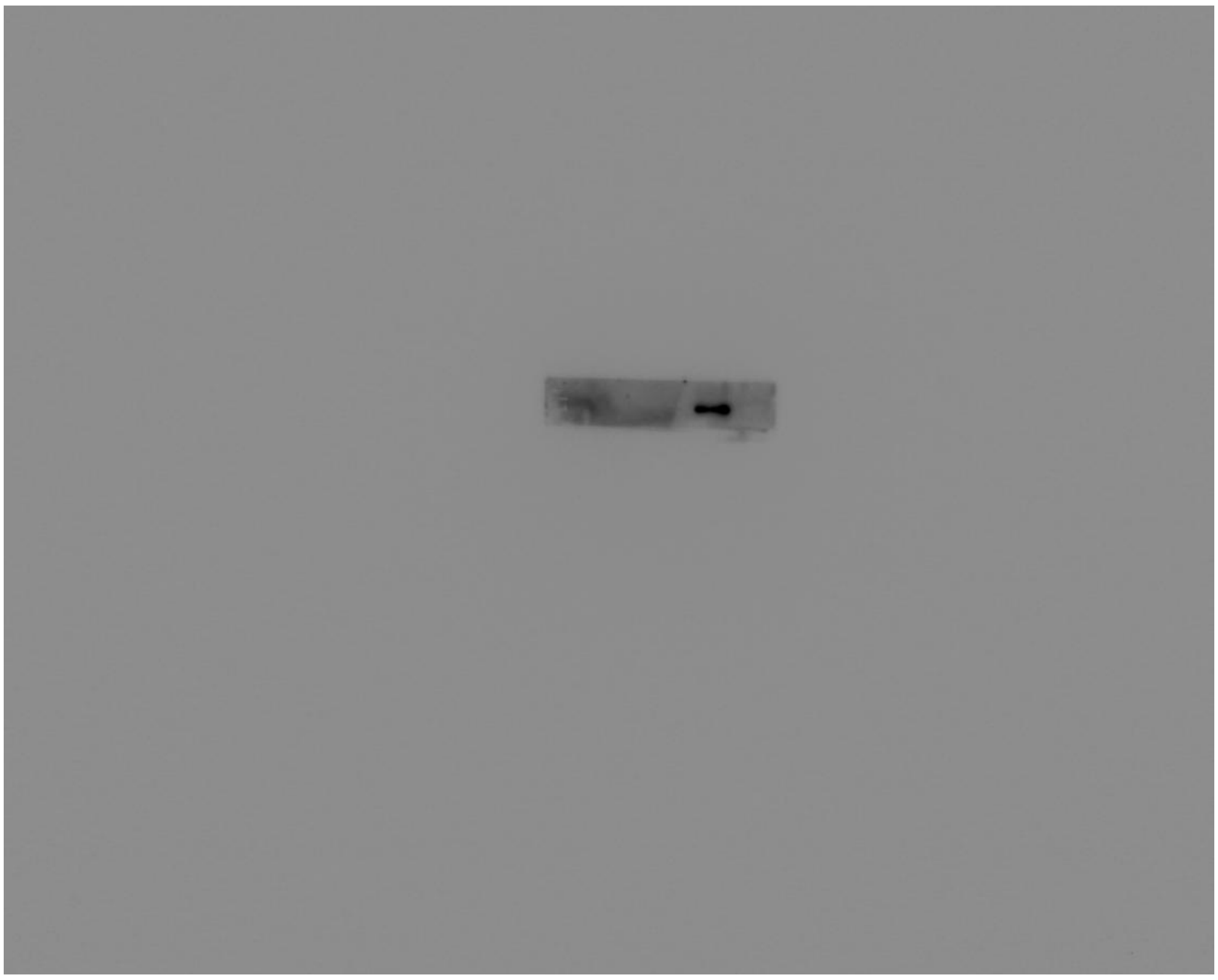

GFP-IP

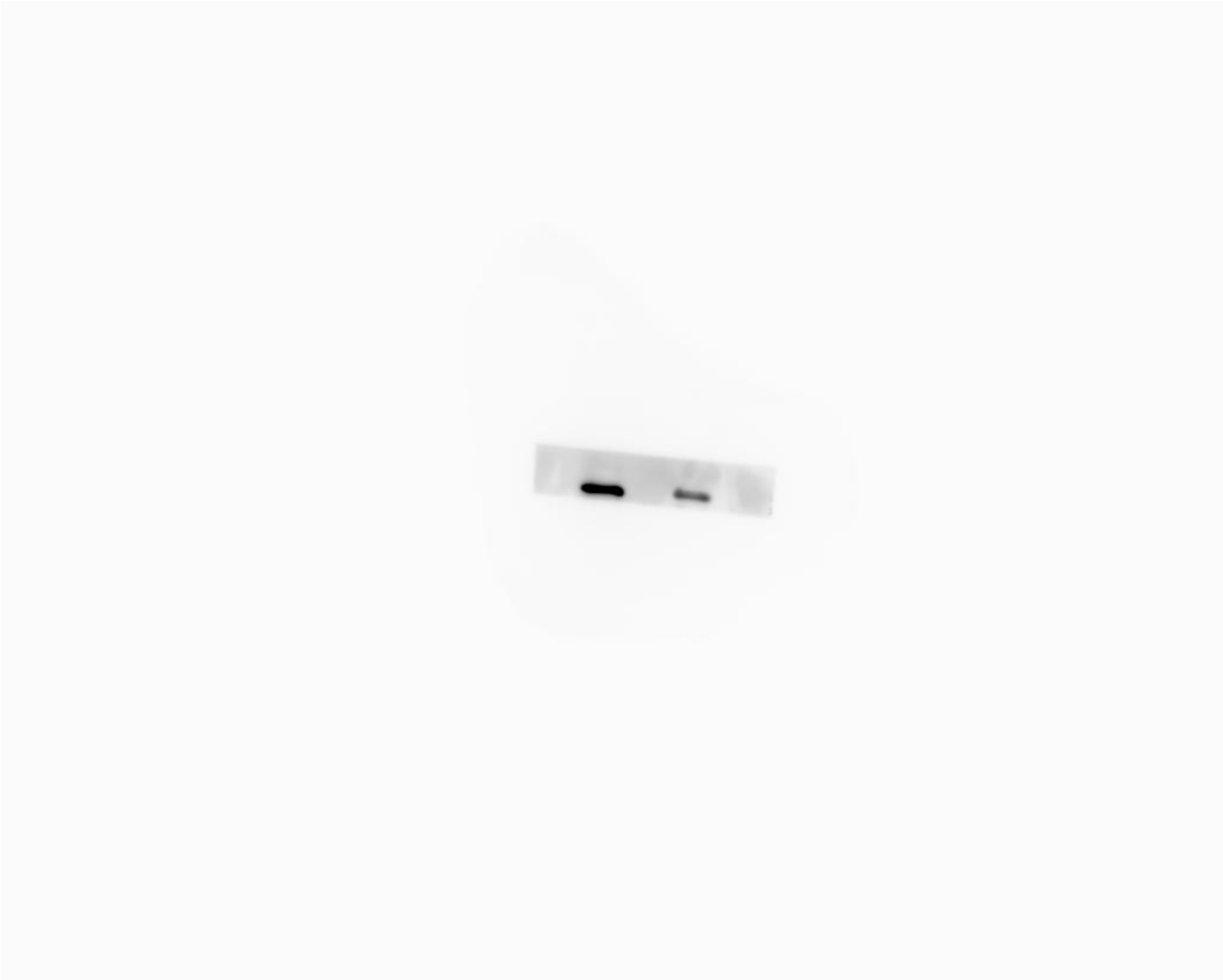

Flag-Input

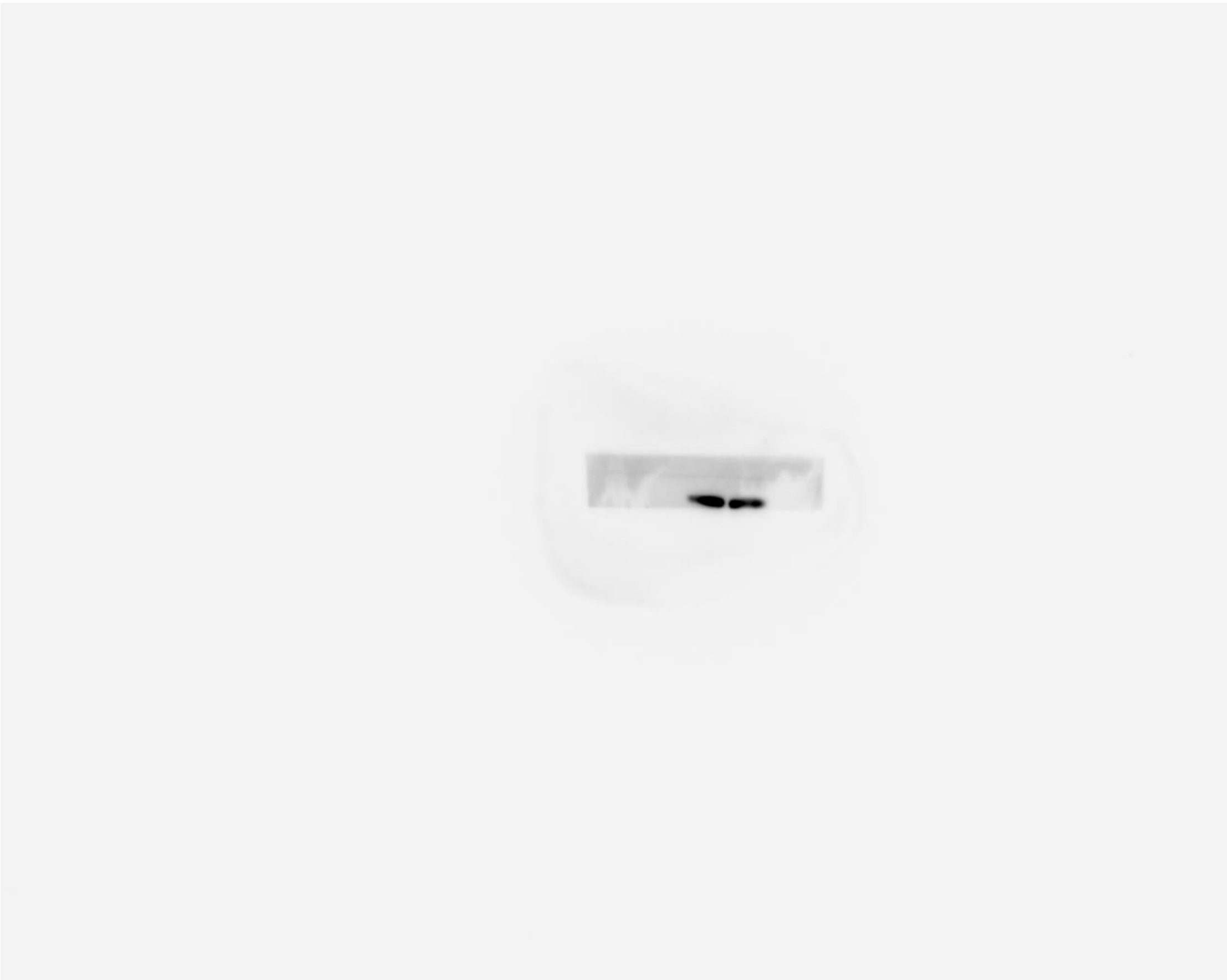

GFP-Input

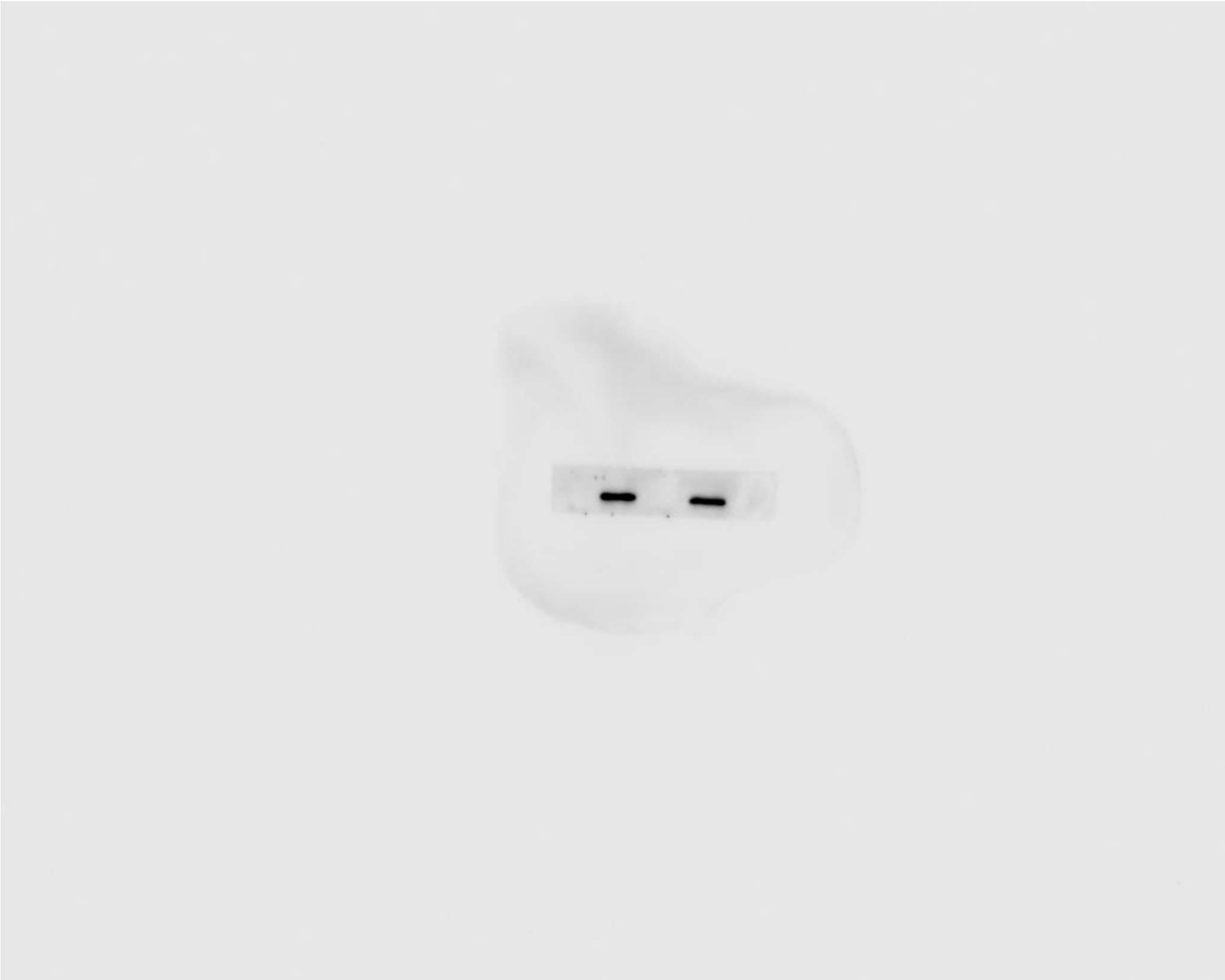

GAPDH-Input

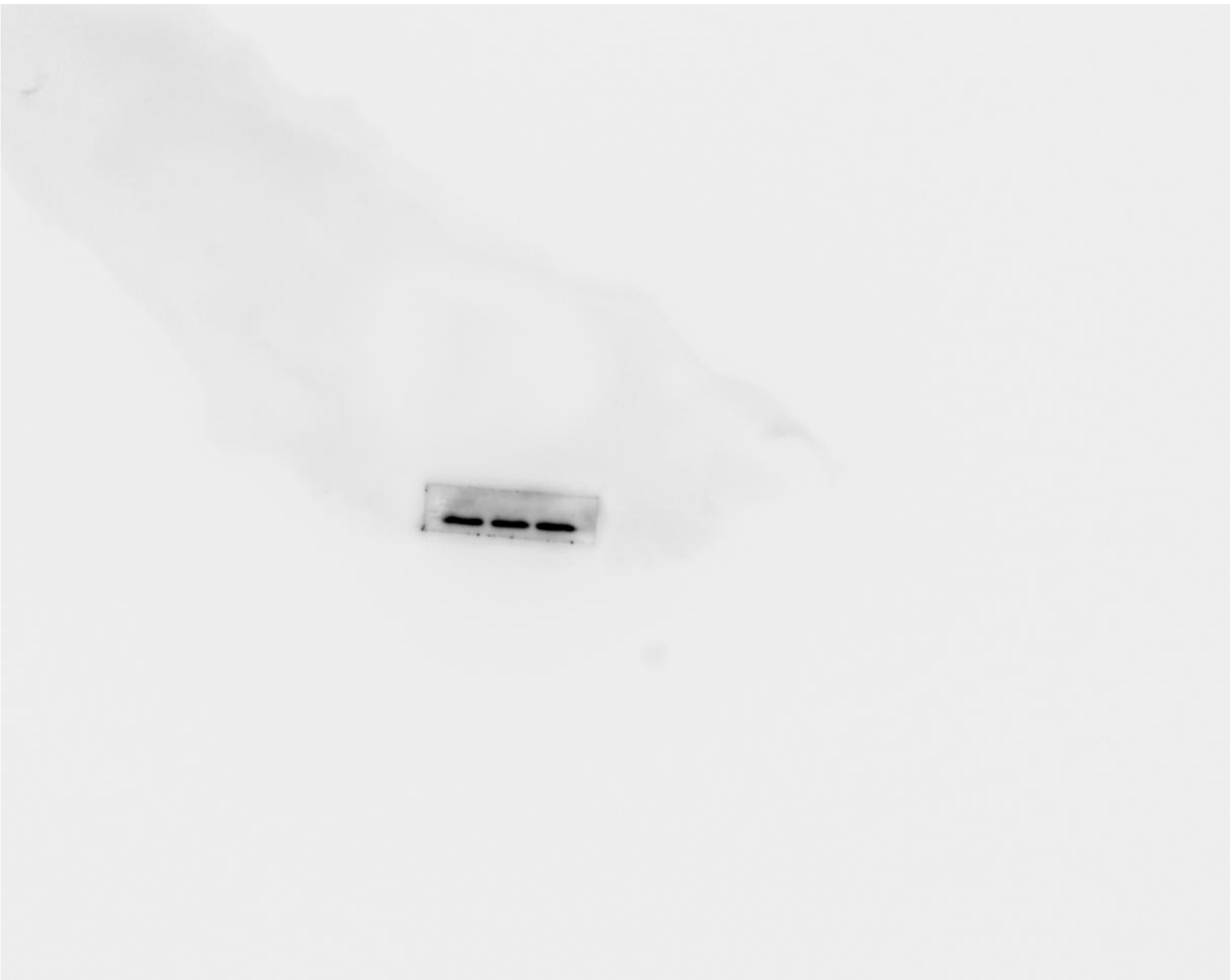

Fig4D

Flag-IP

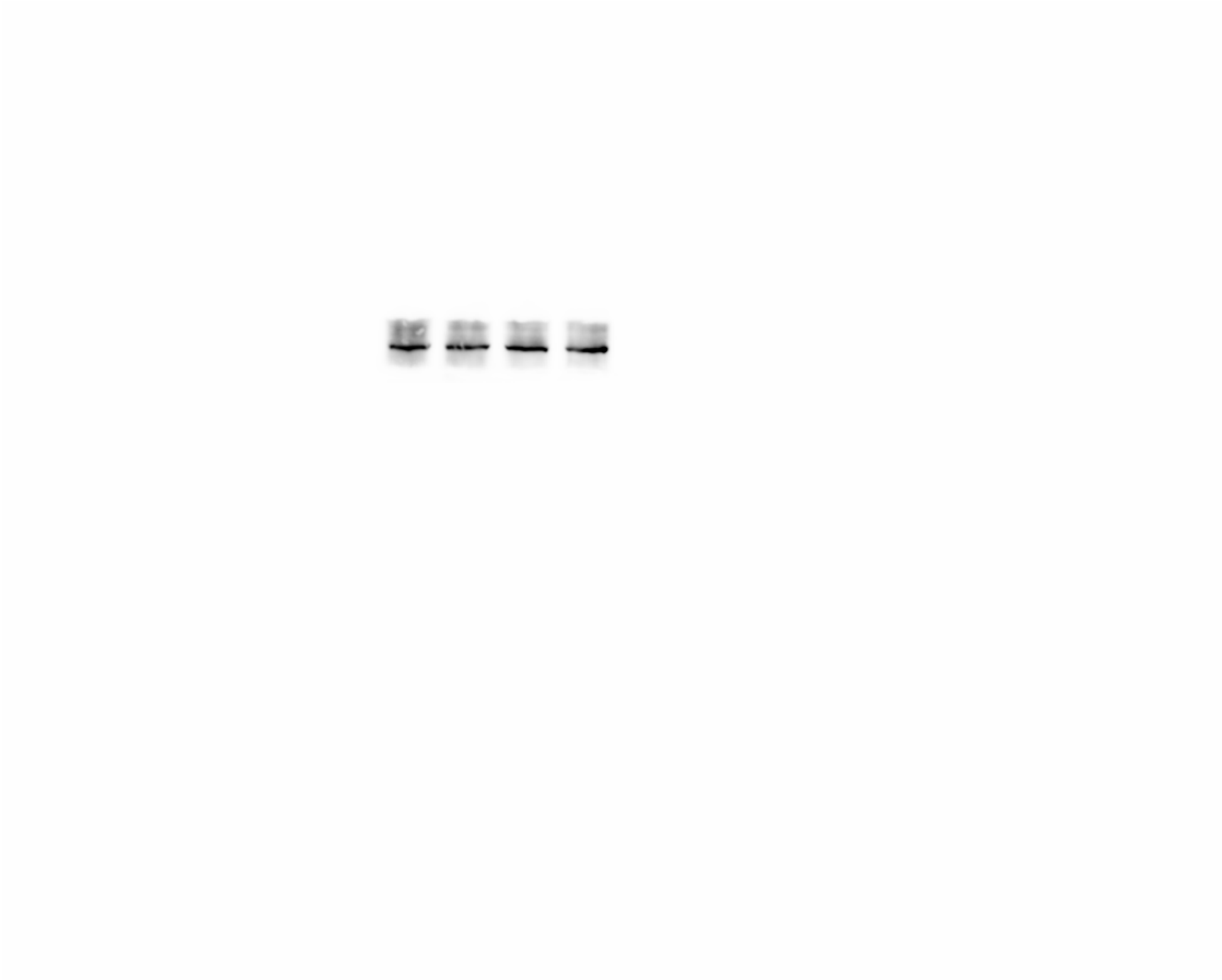

GFP-IP

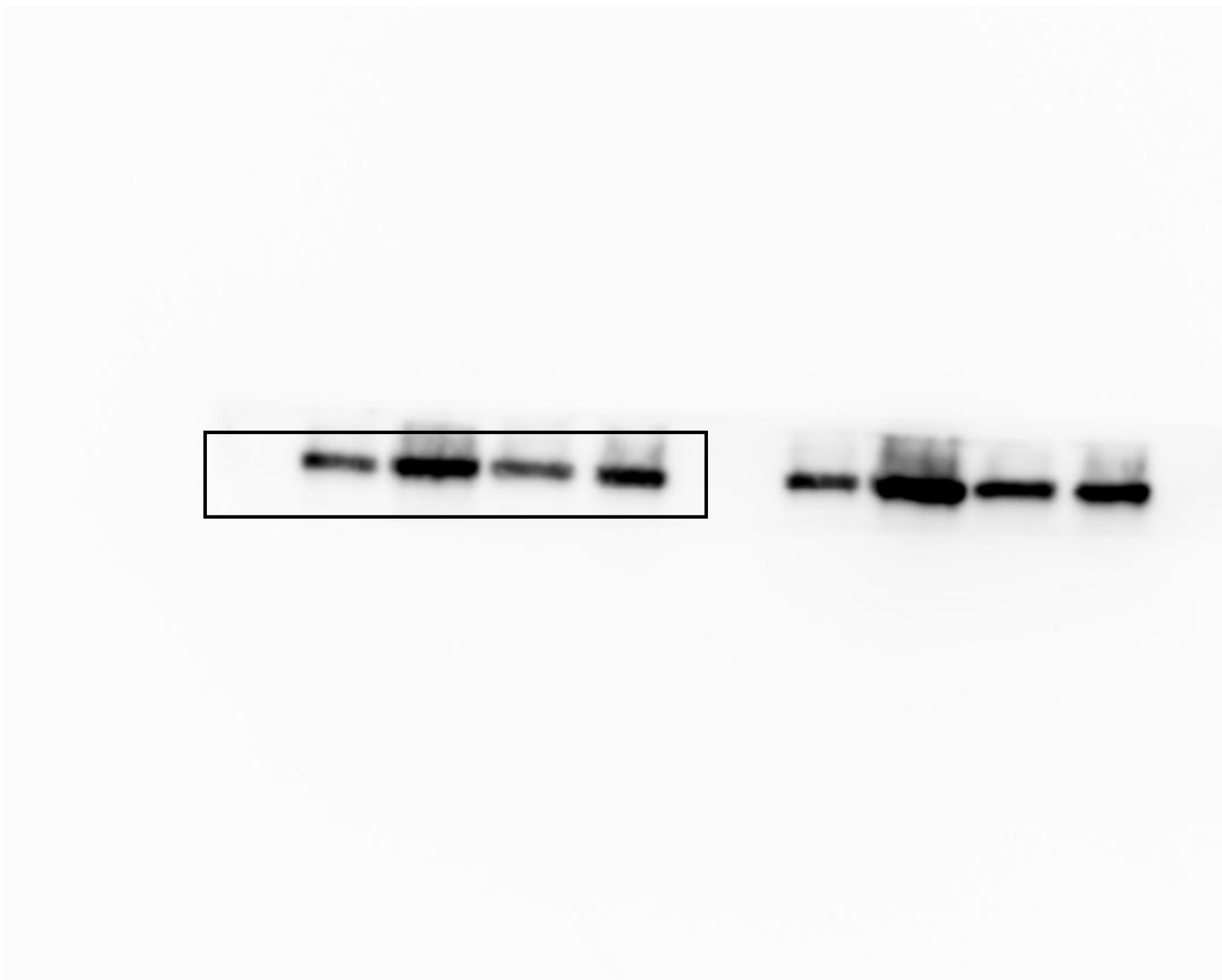

Flag-Input

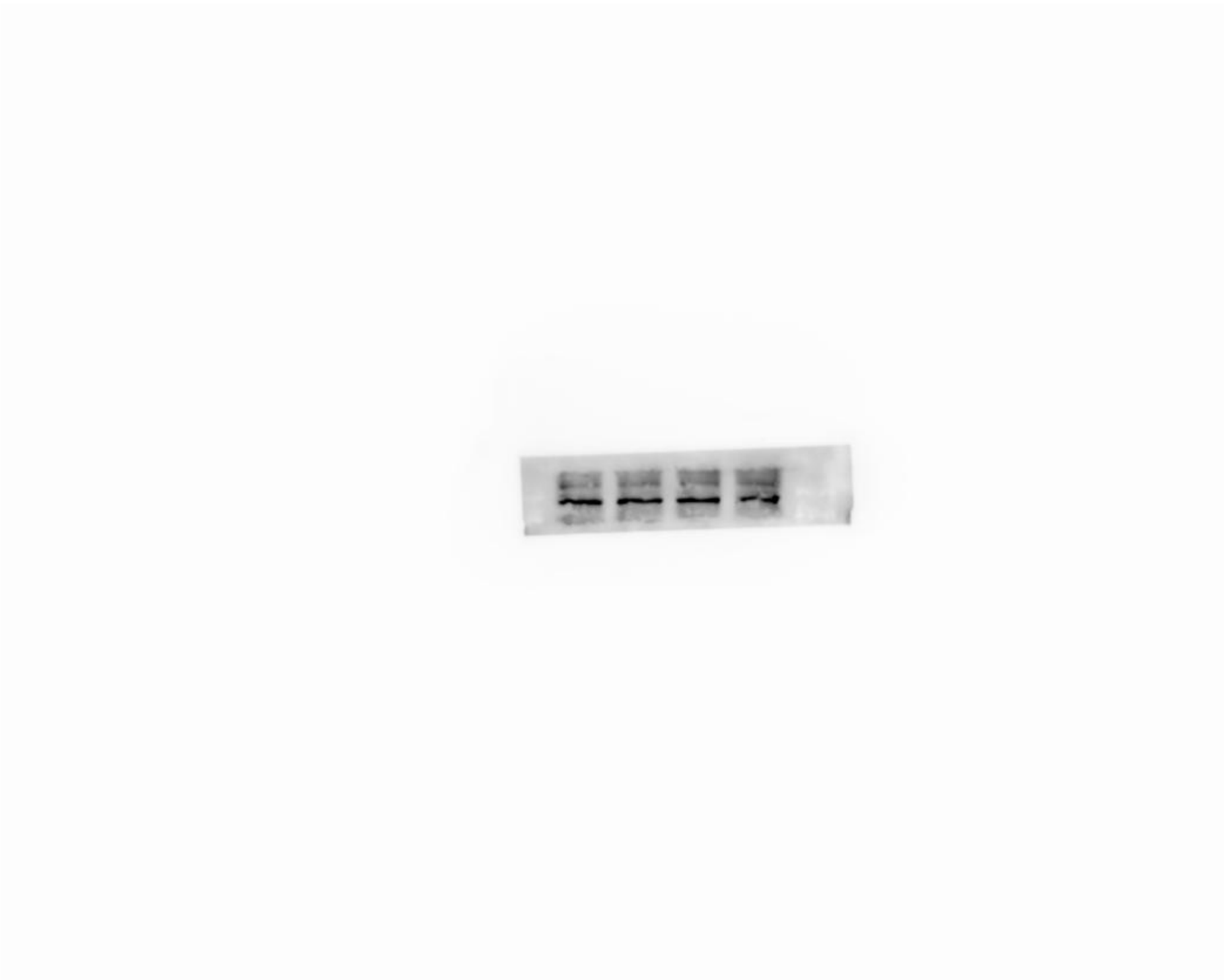

GFP-Input

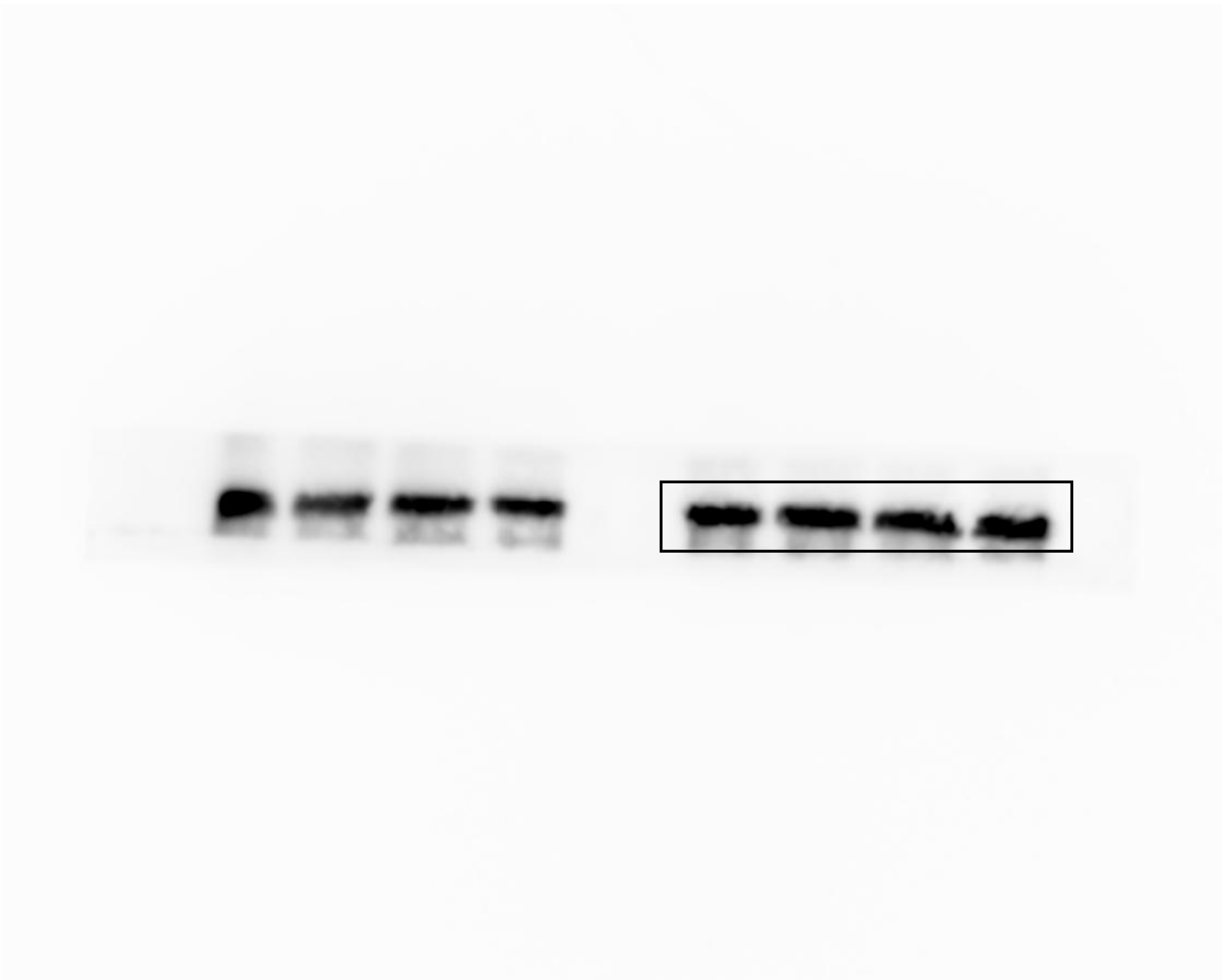

ACTIN-Input

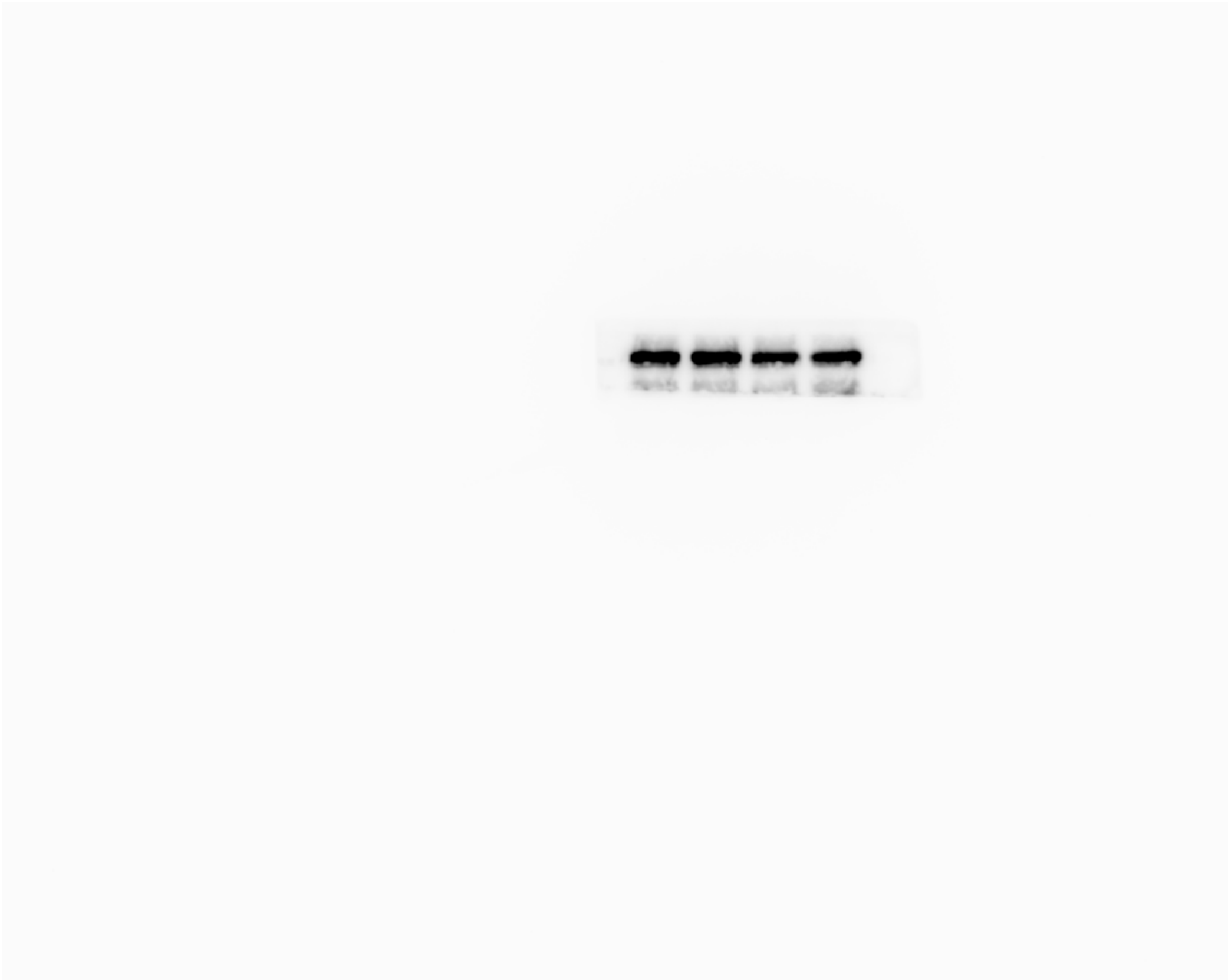

Fig4E

GFP-IP

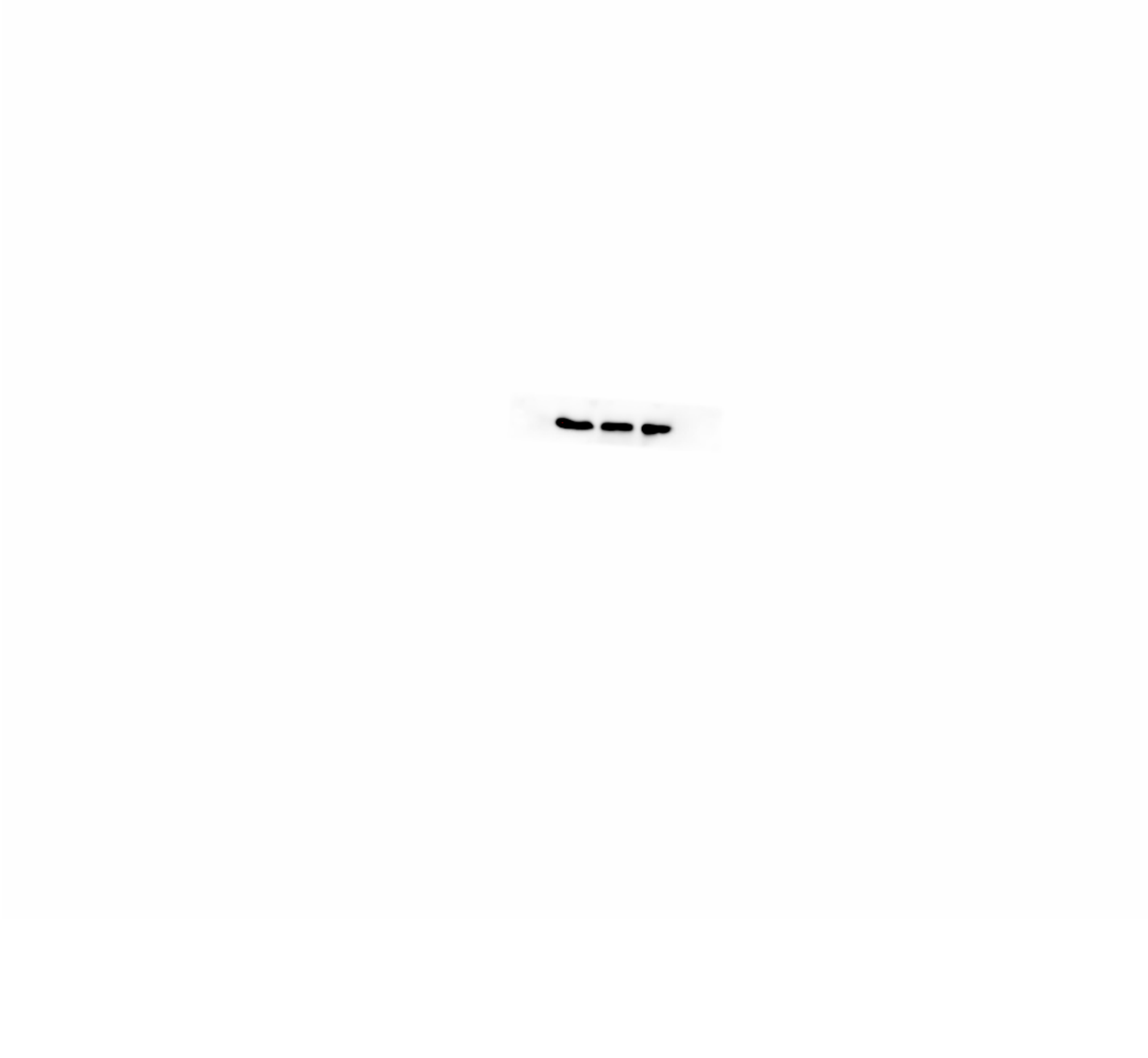

Flag-IP

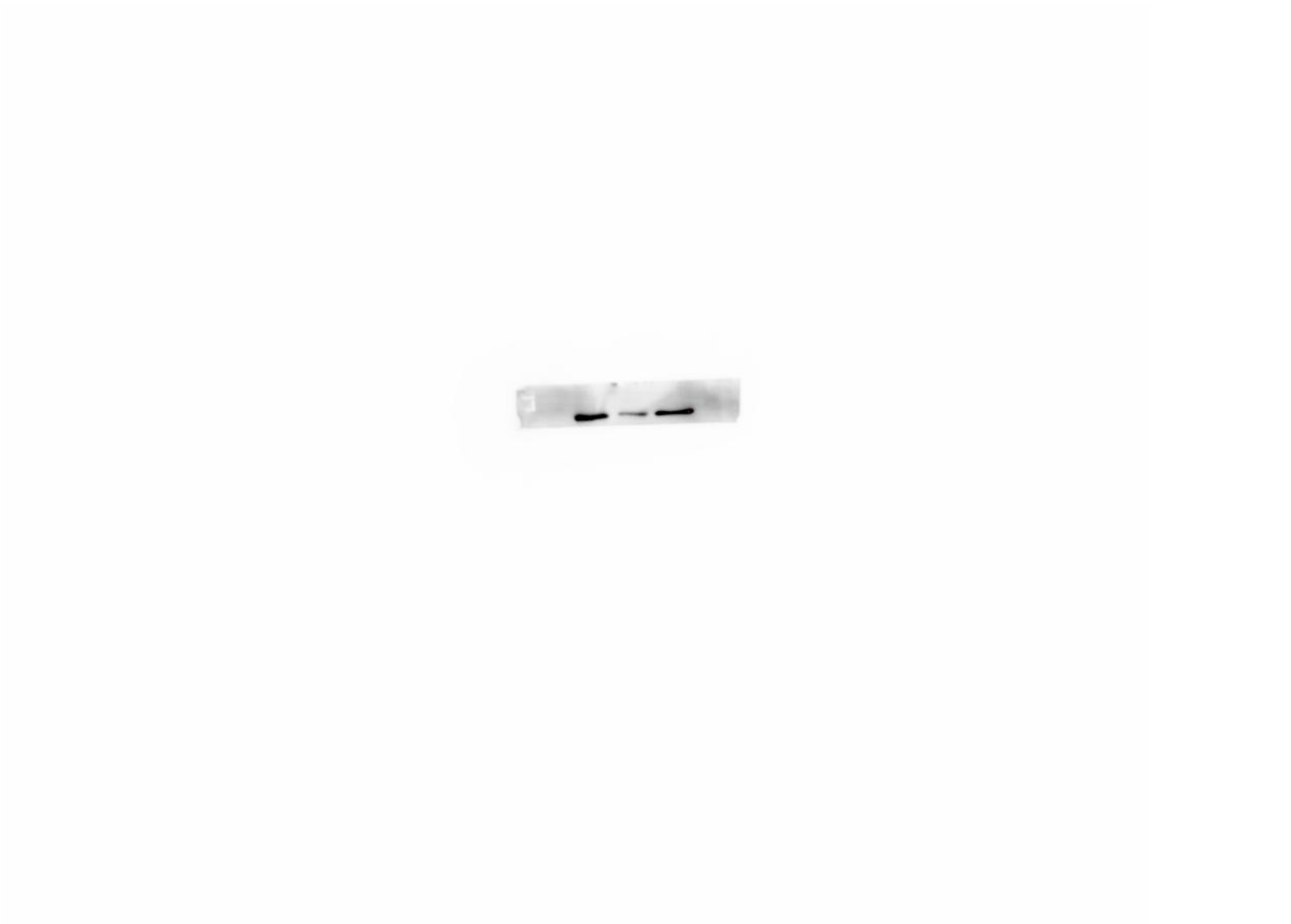

Flag-Input

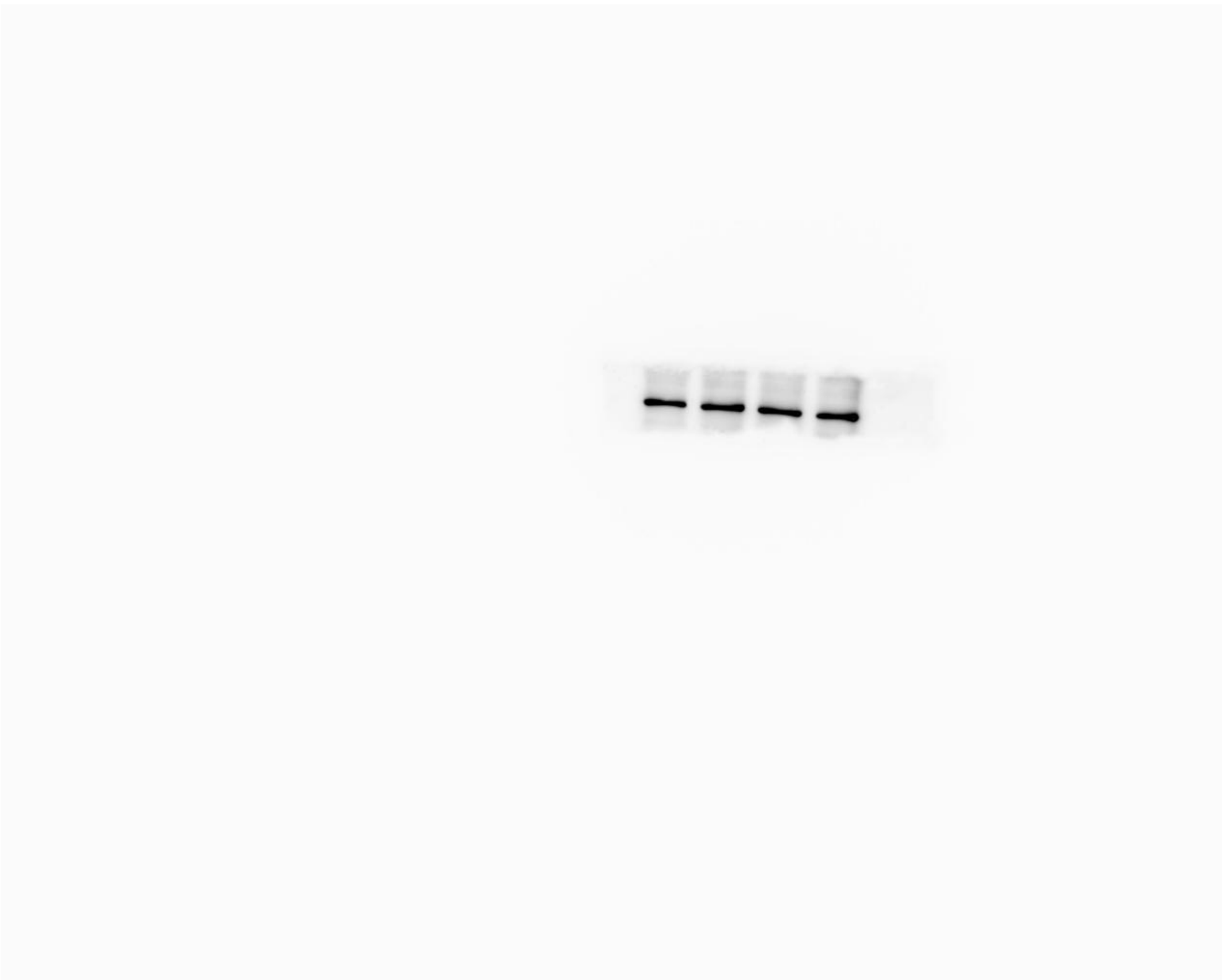

GFP-Input

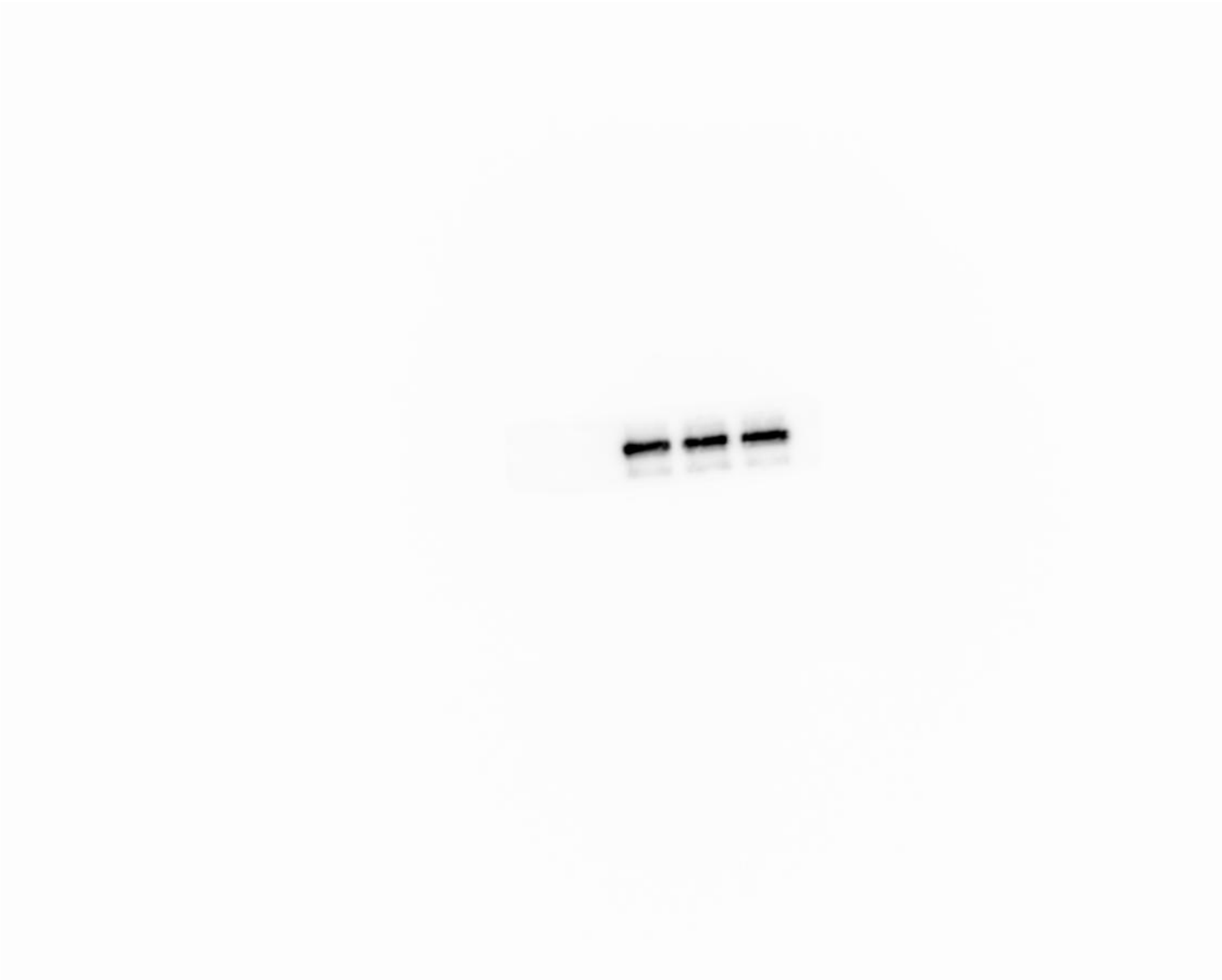

ACTIN-Input

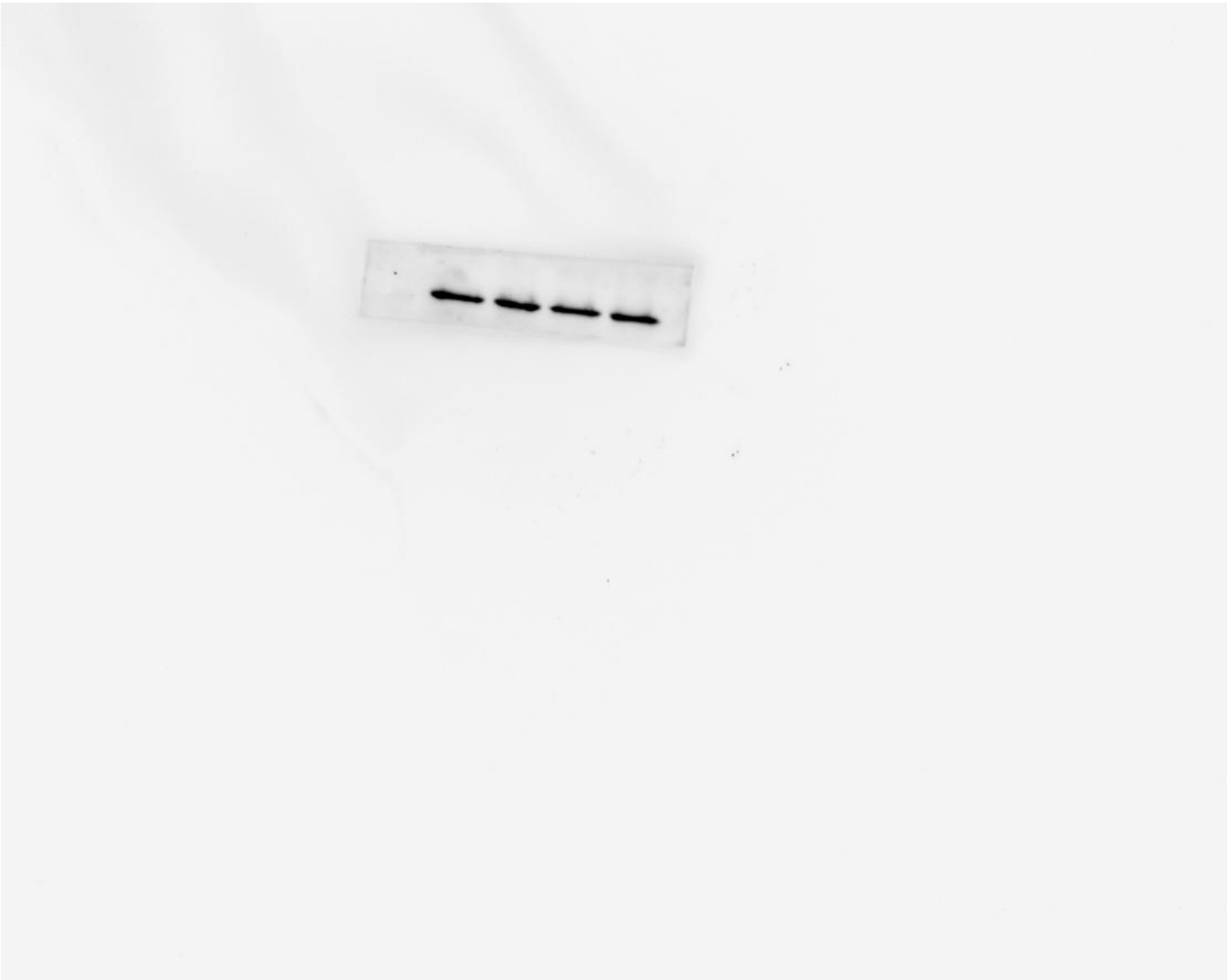

Fig4F

MED12

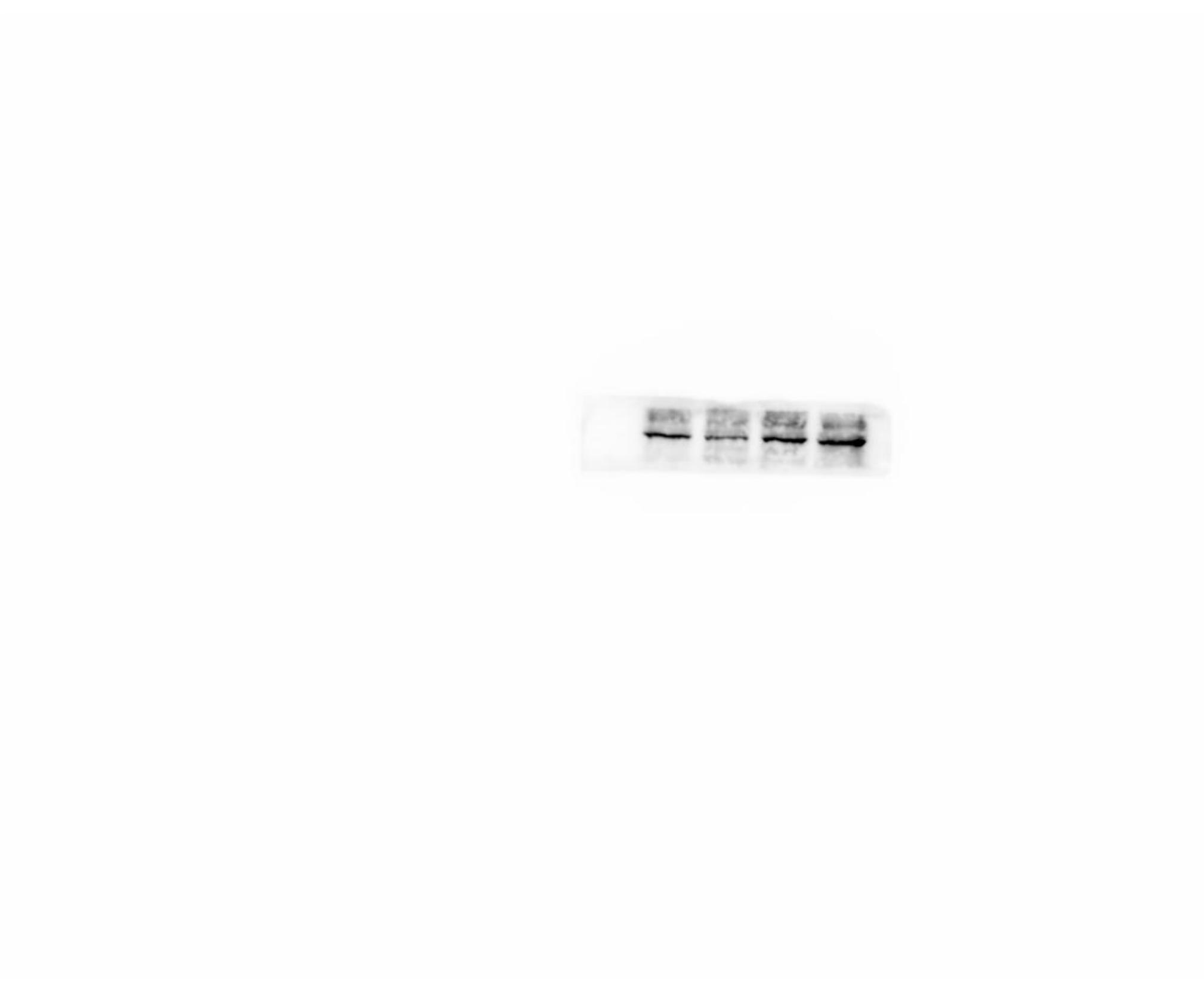

GFP

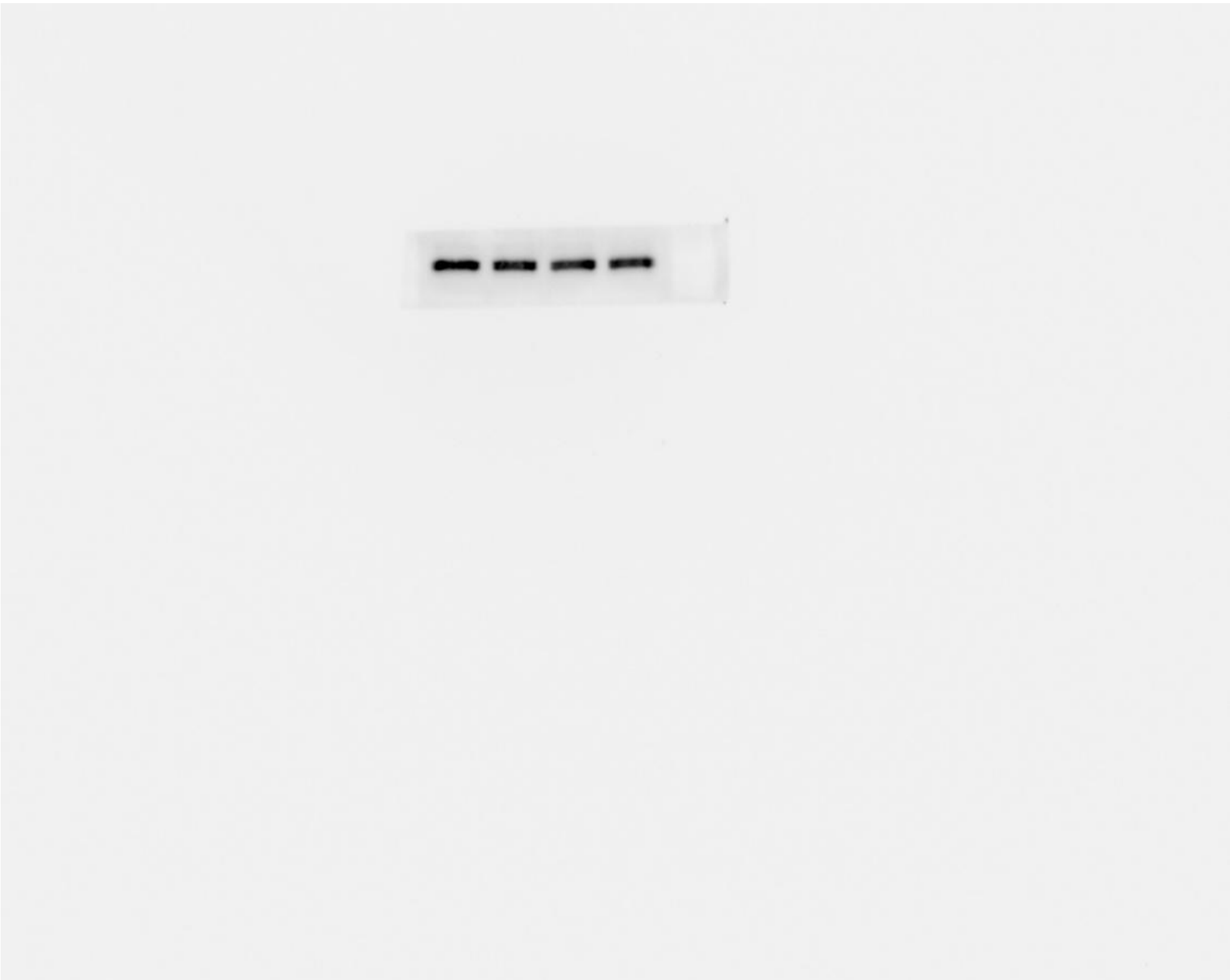

ACTIN

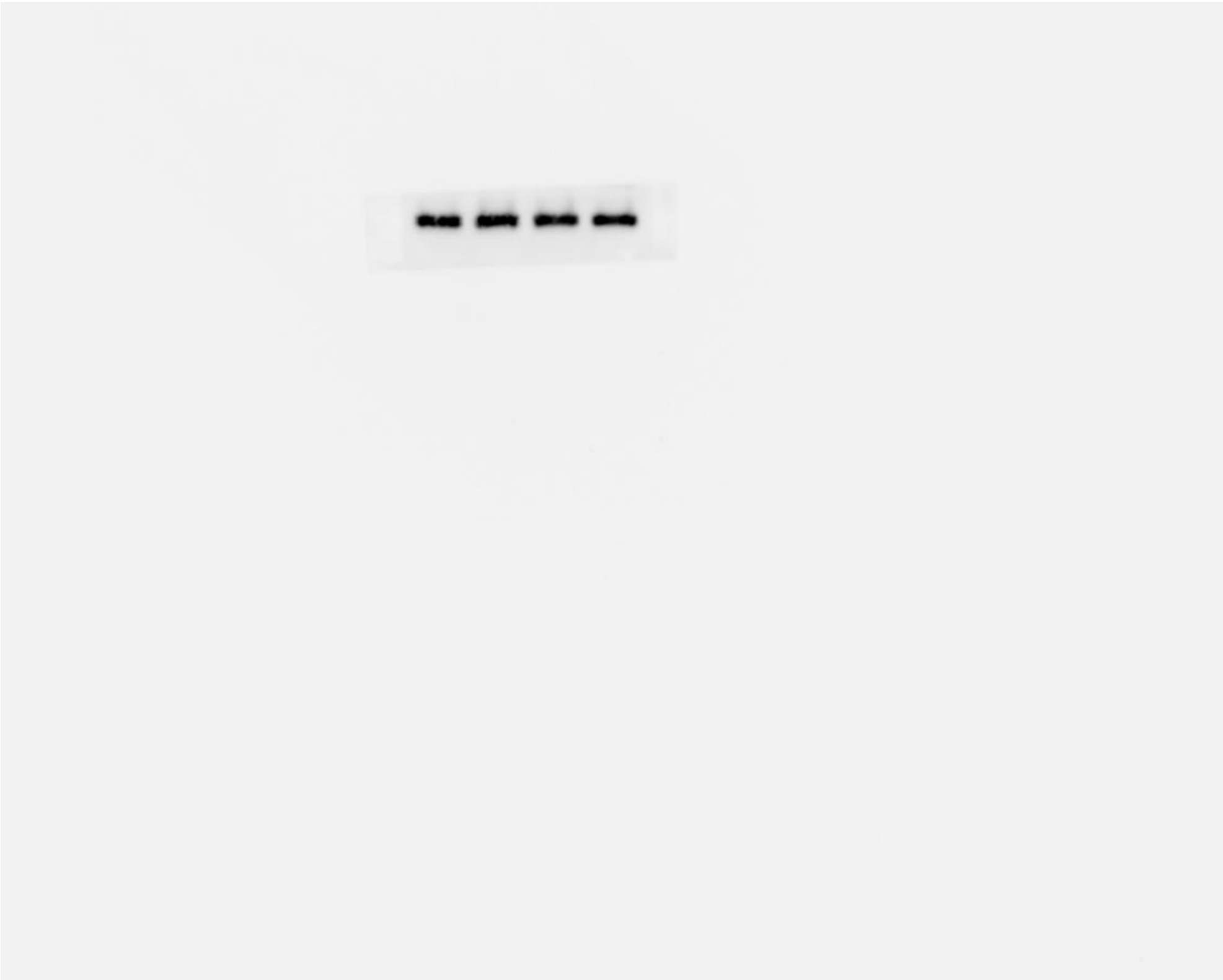

Fig4G

MED12

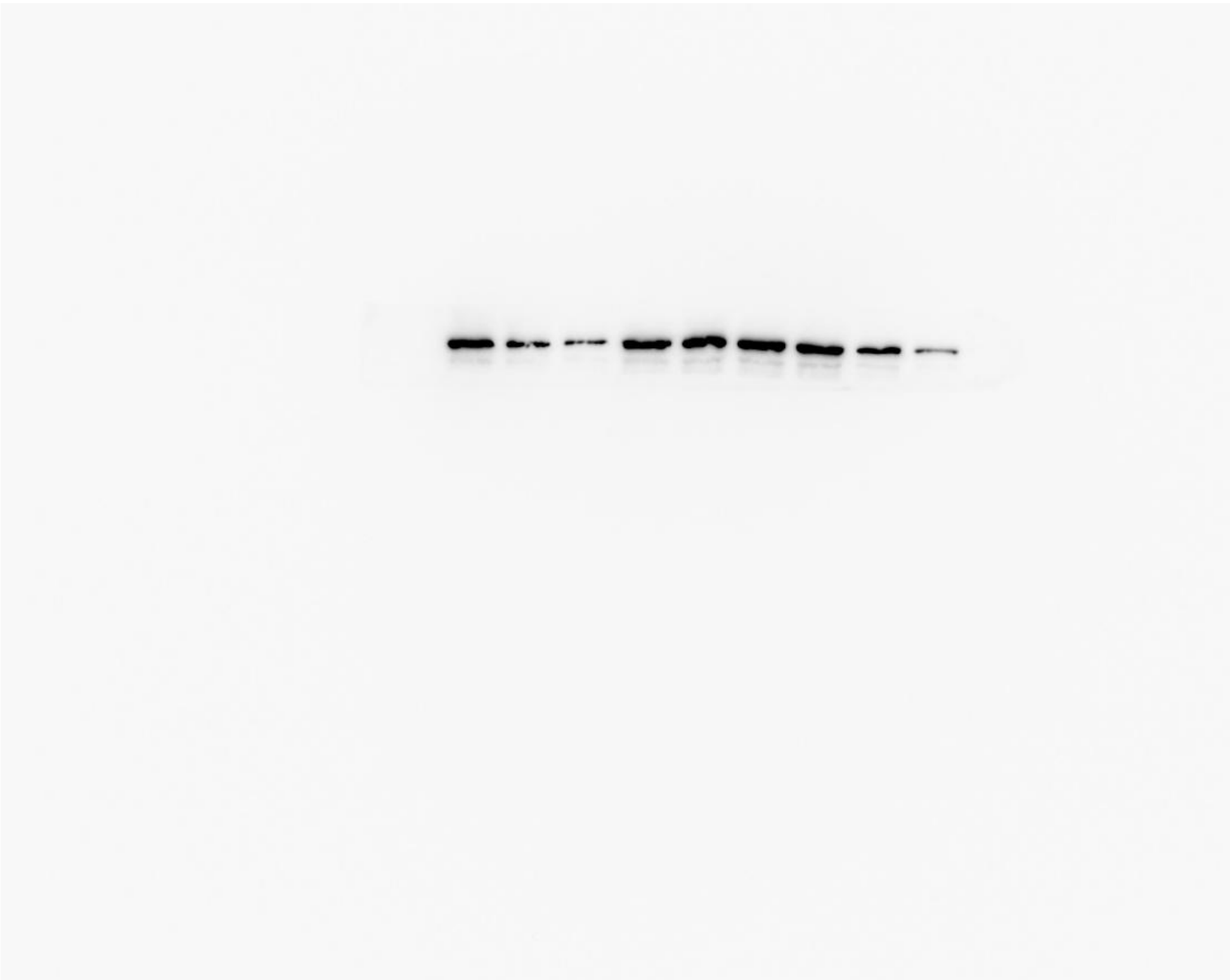

GFP

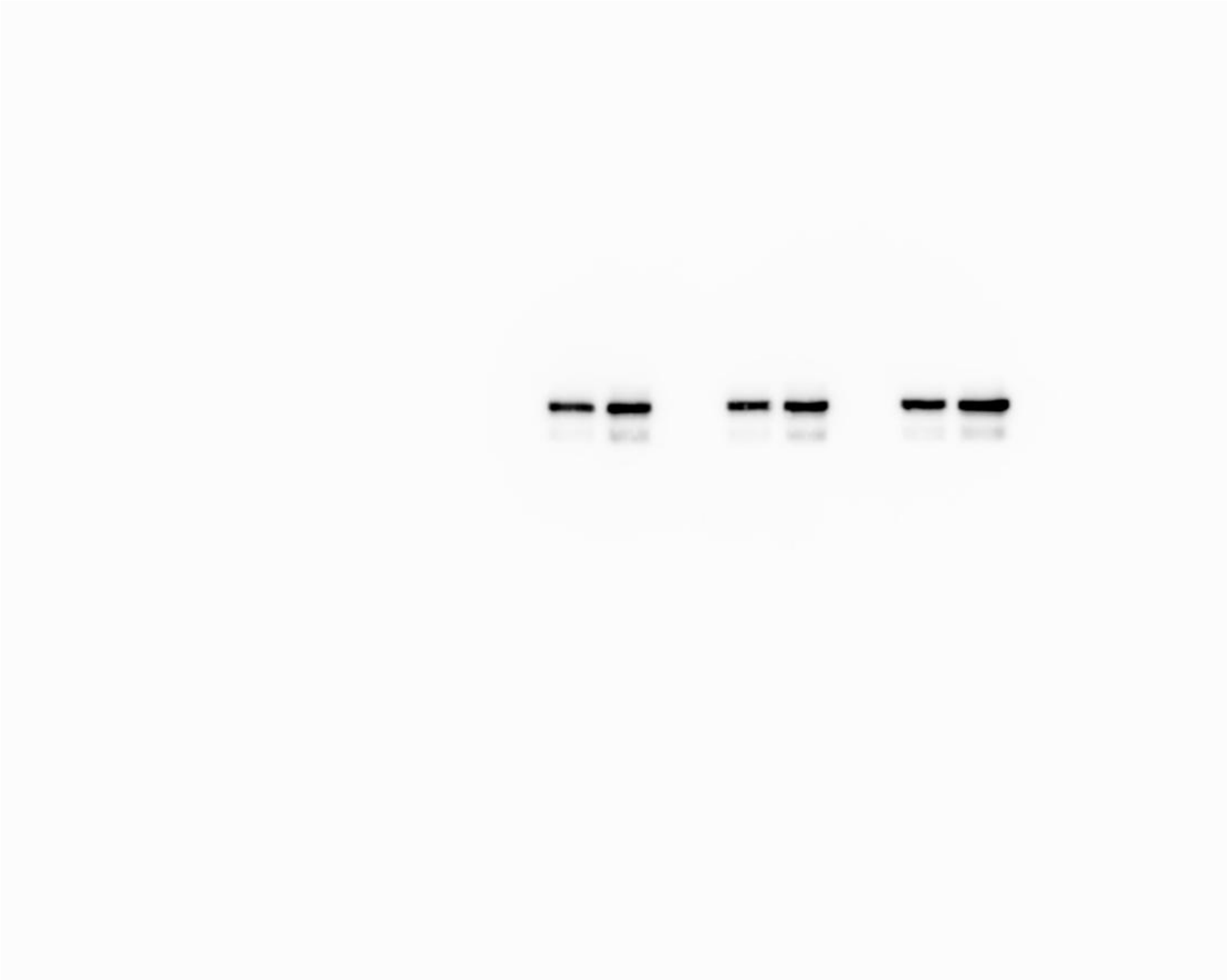

GAPDH

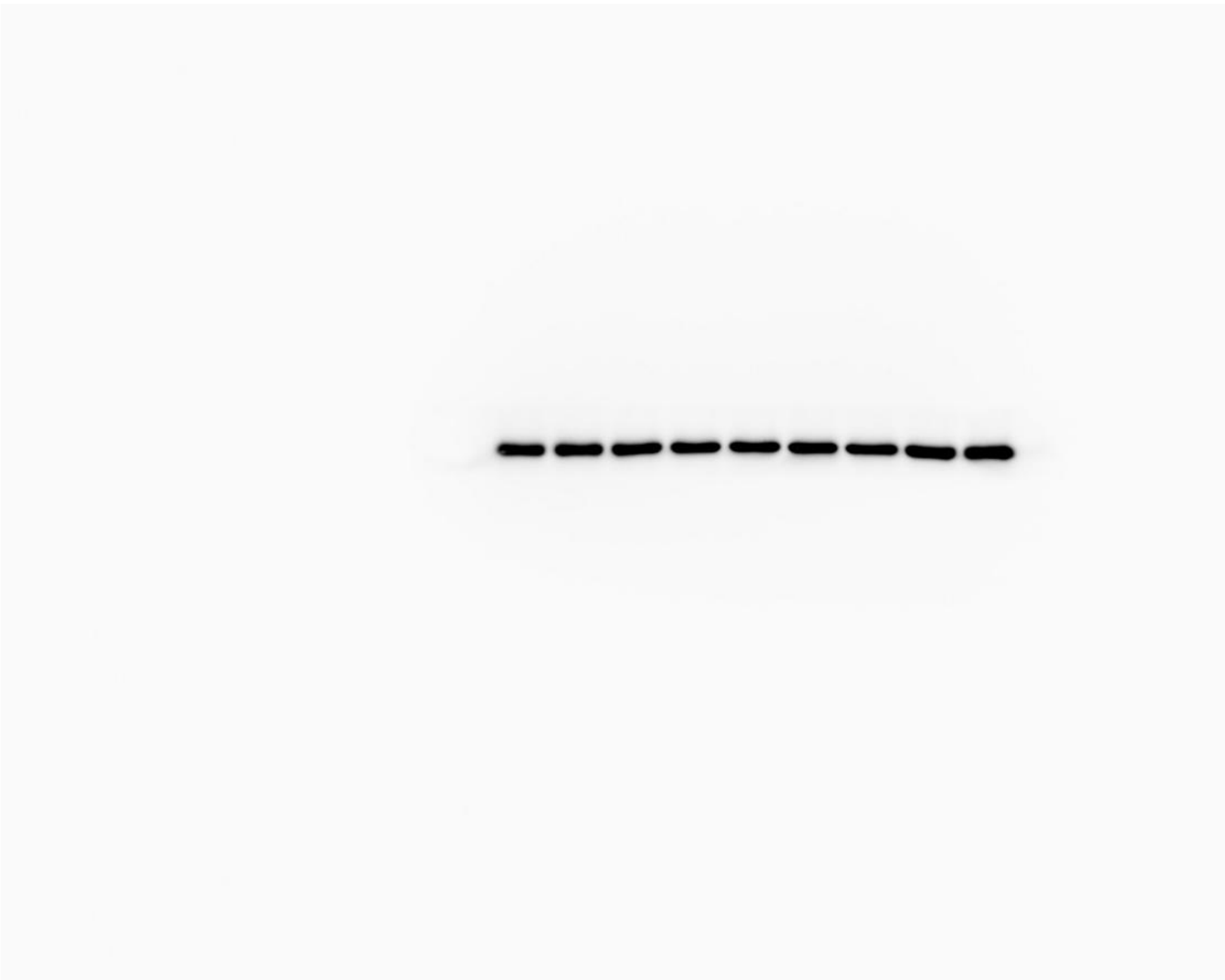

Fig5B

MED12

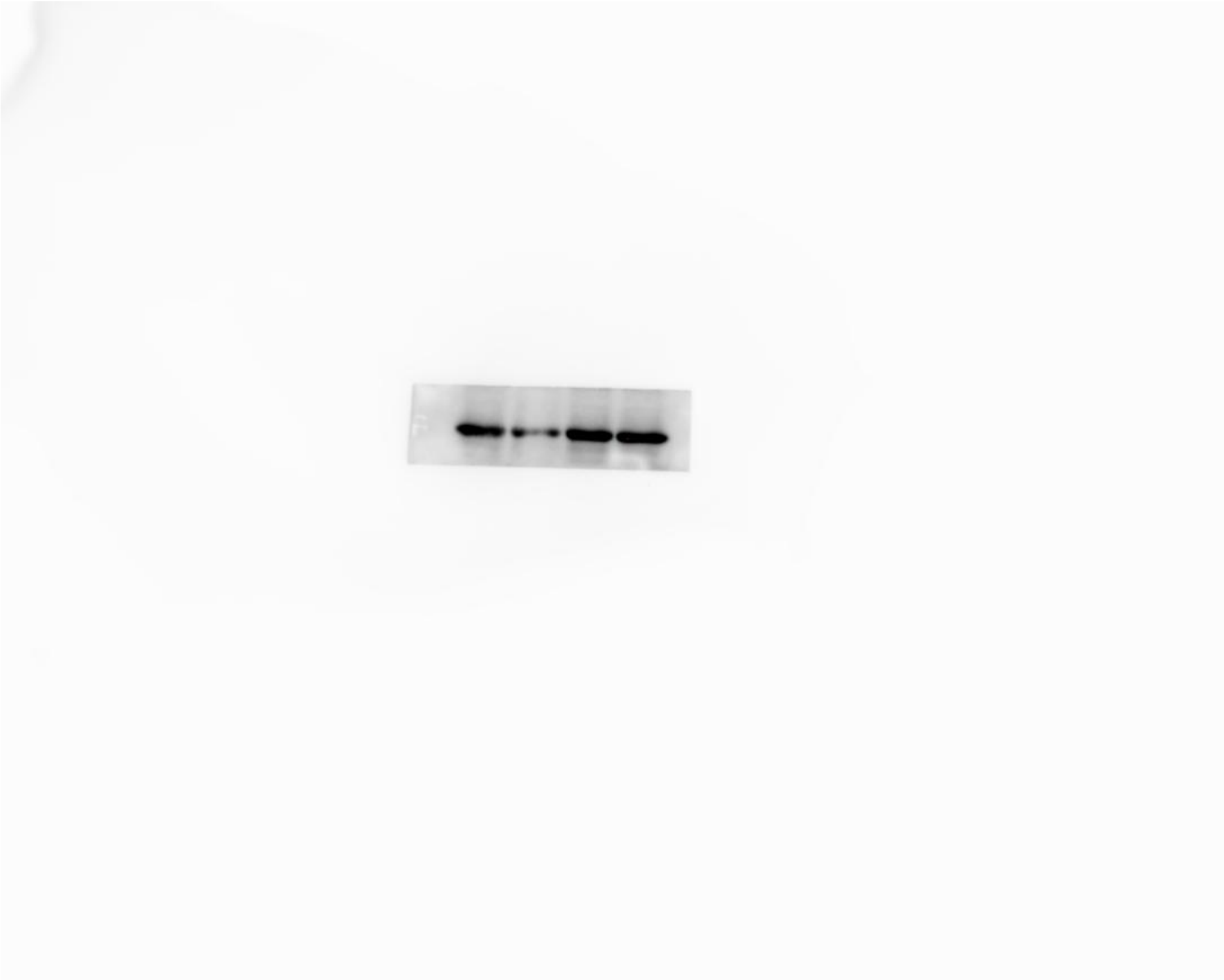

GFP

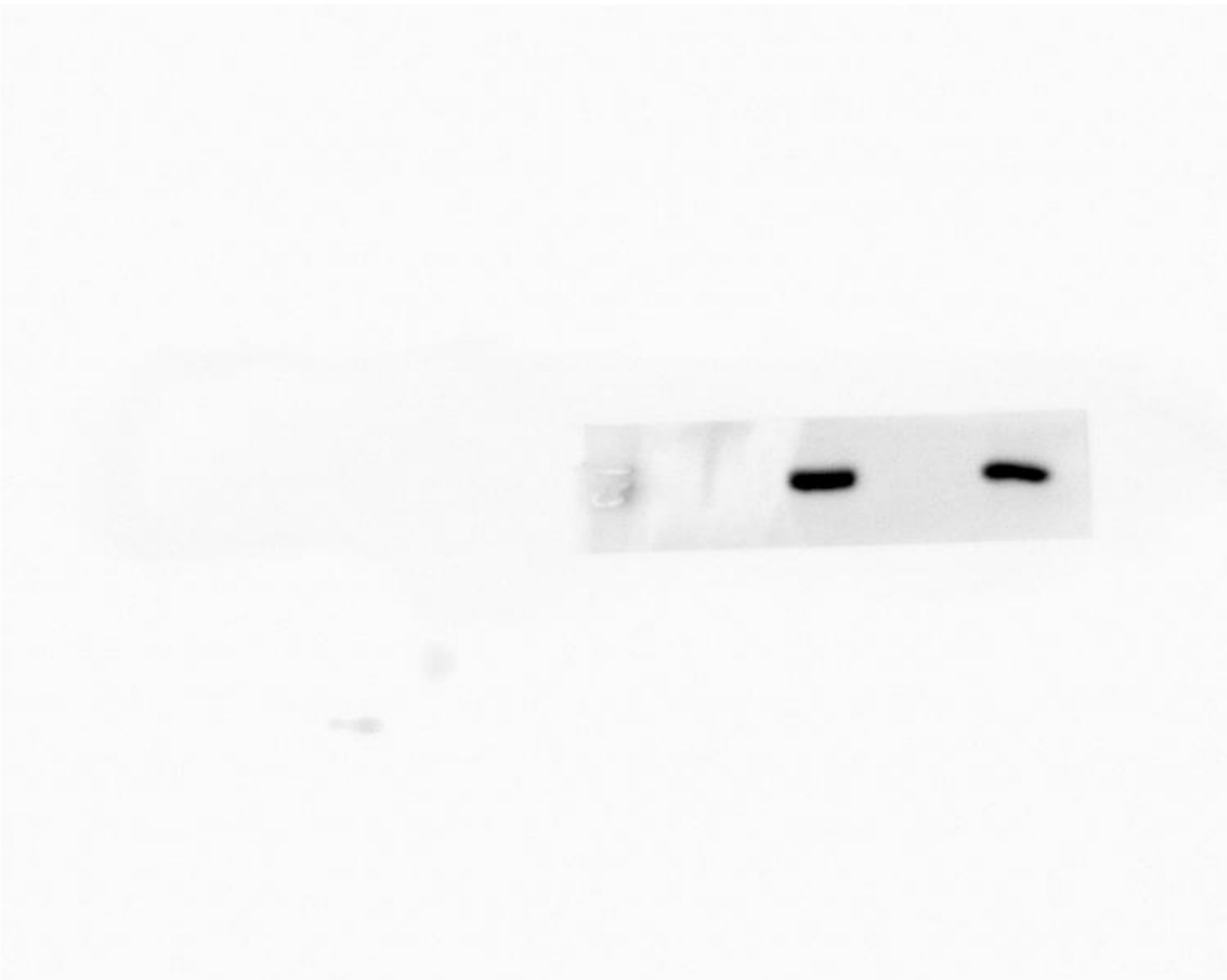

GAPDH

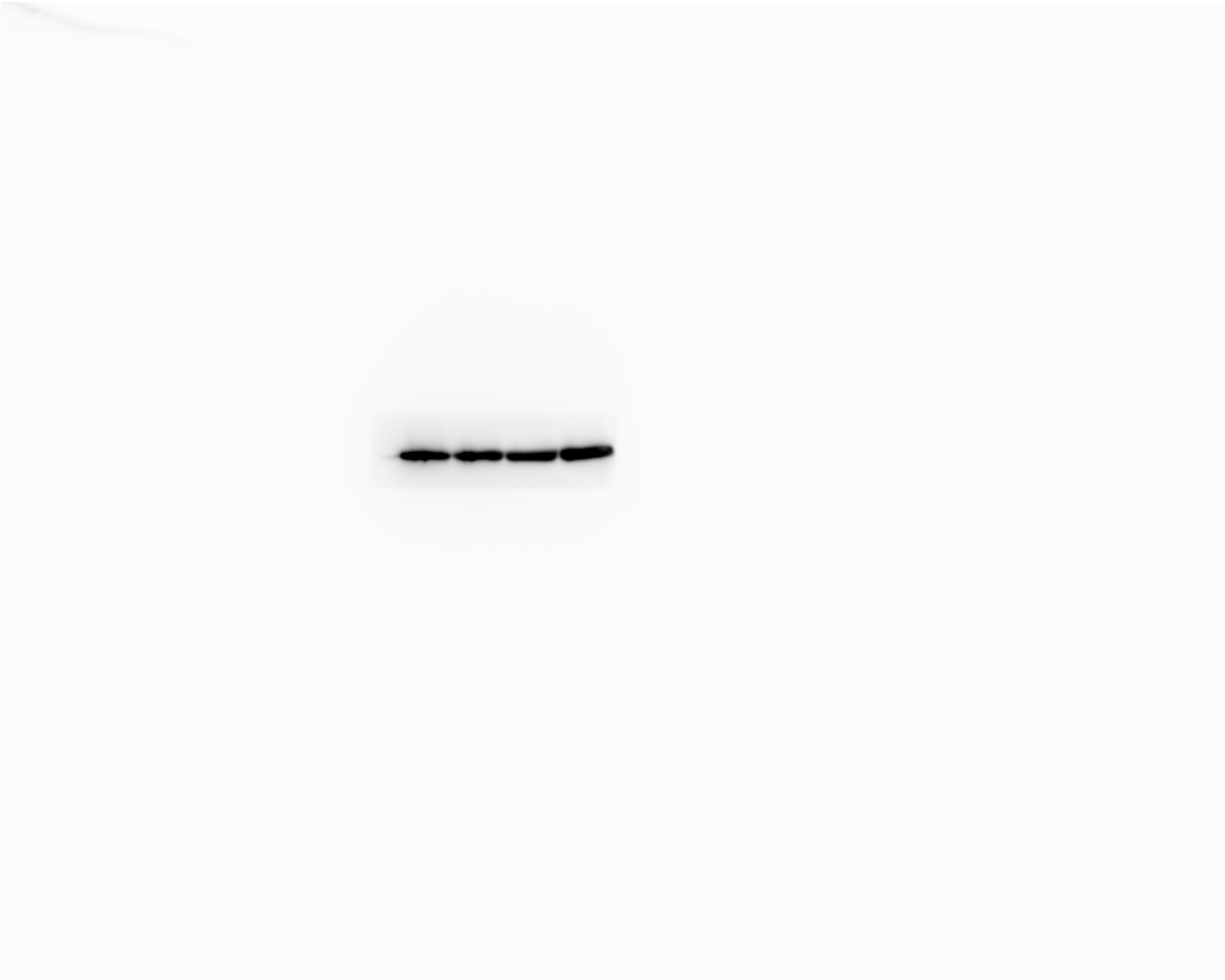

Fig5C

MED12

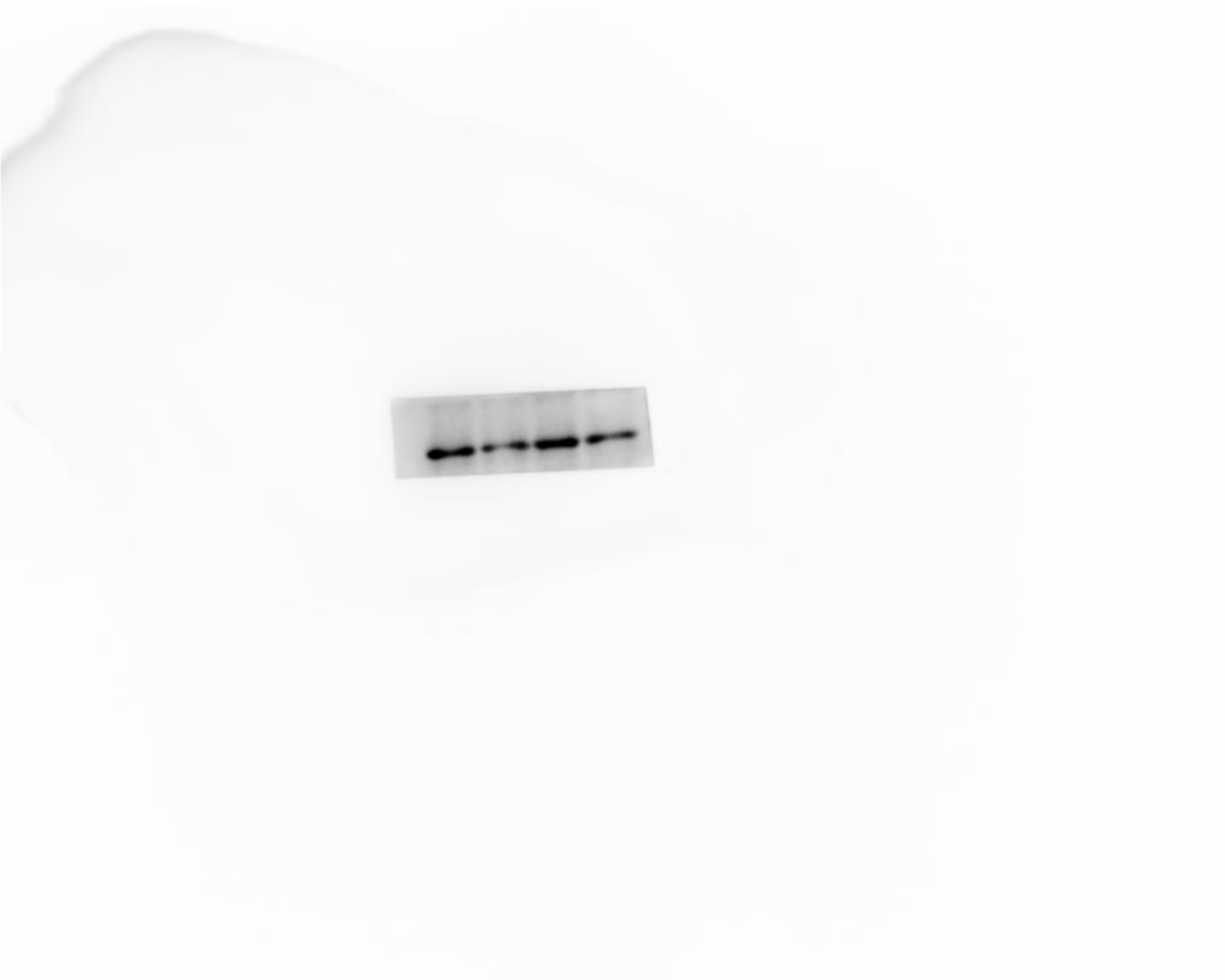

GFP

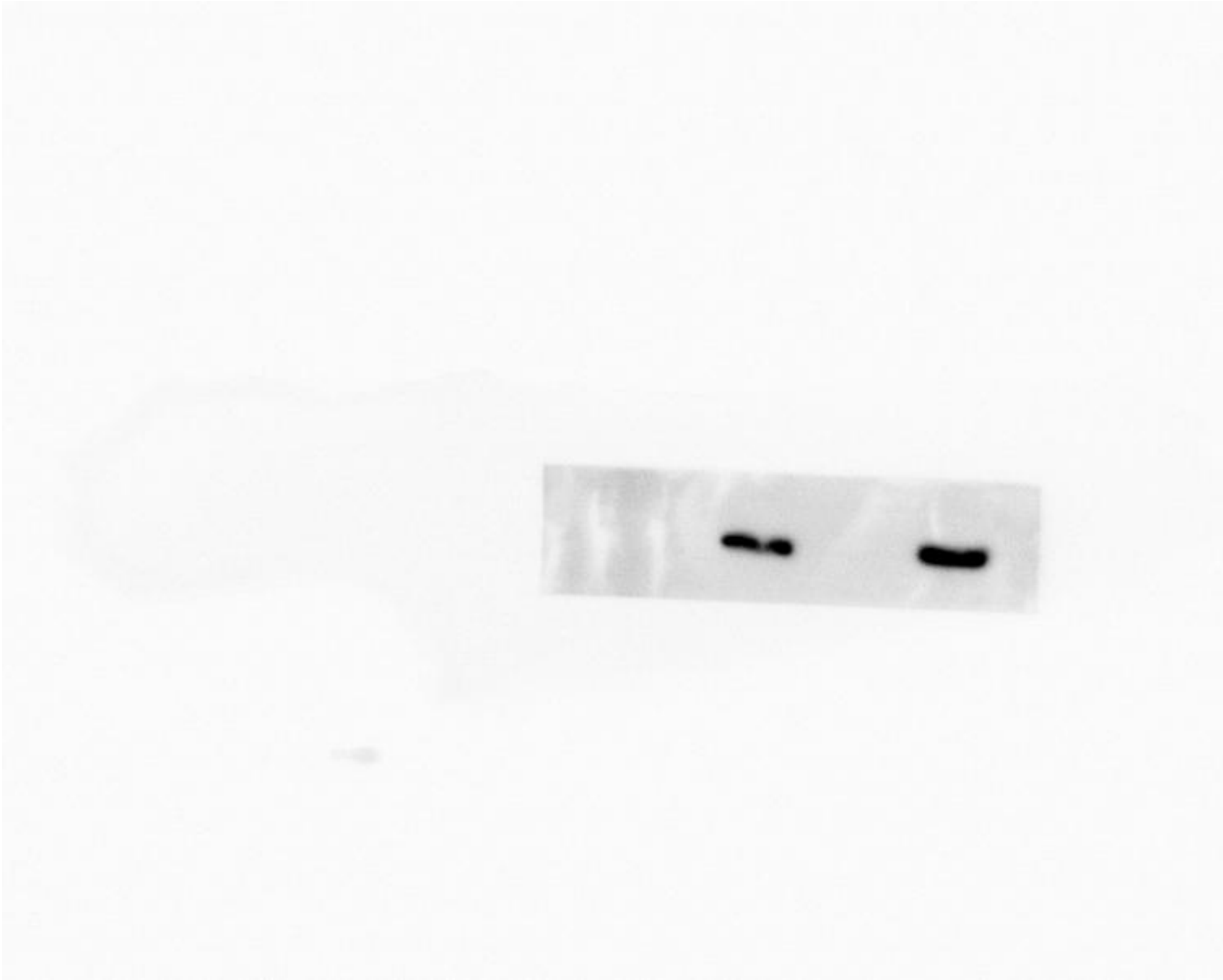

GAPDH

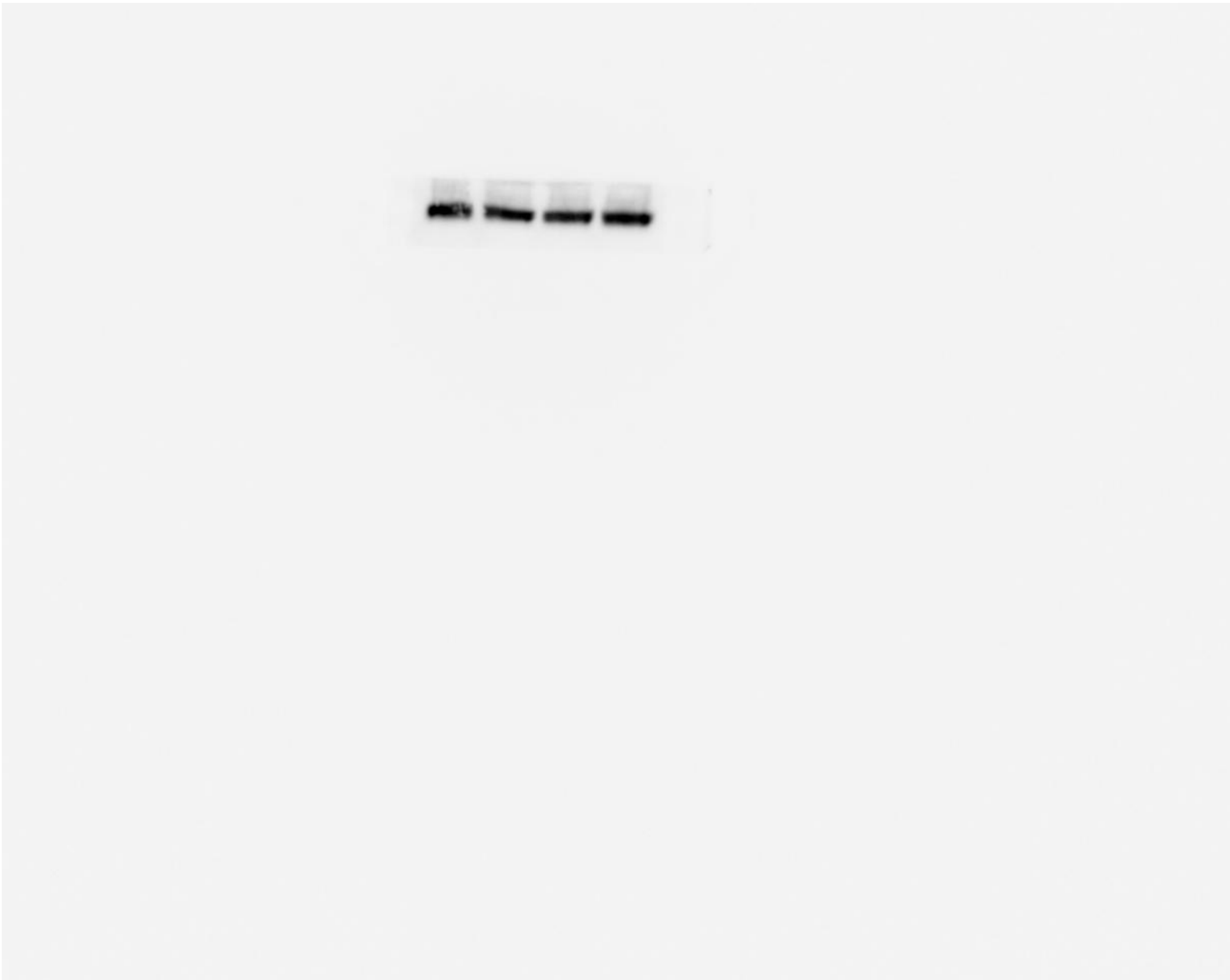

Fig5D

MED12

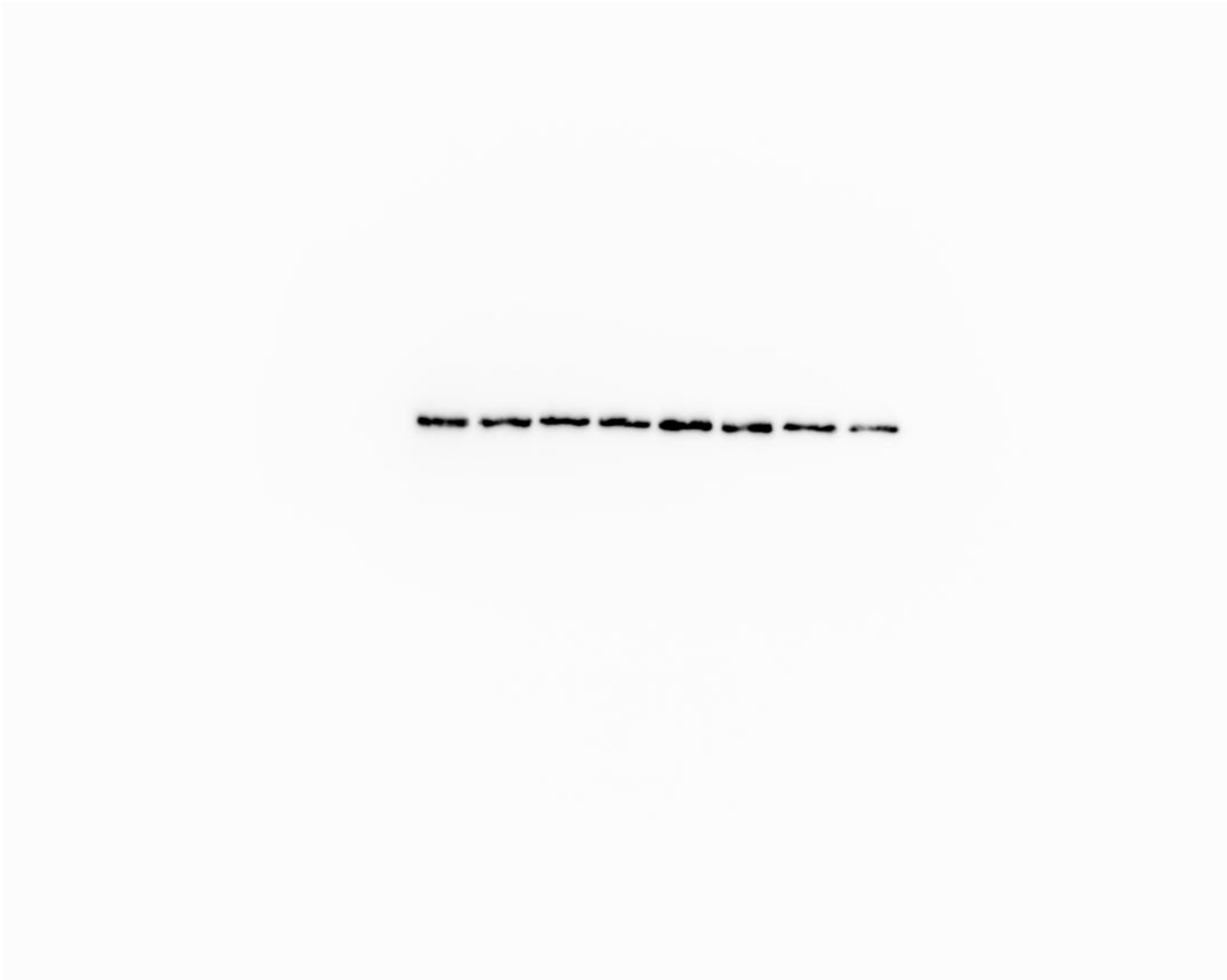

GAPDH

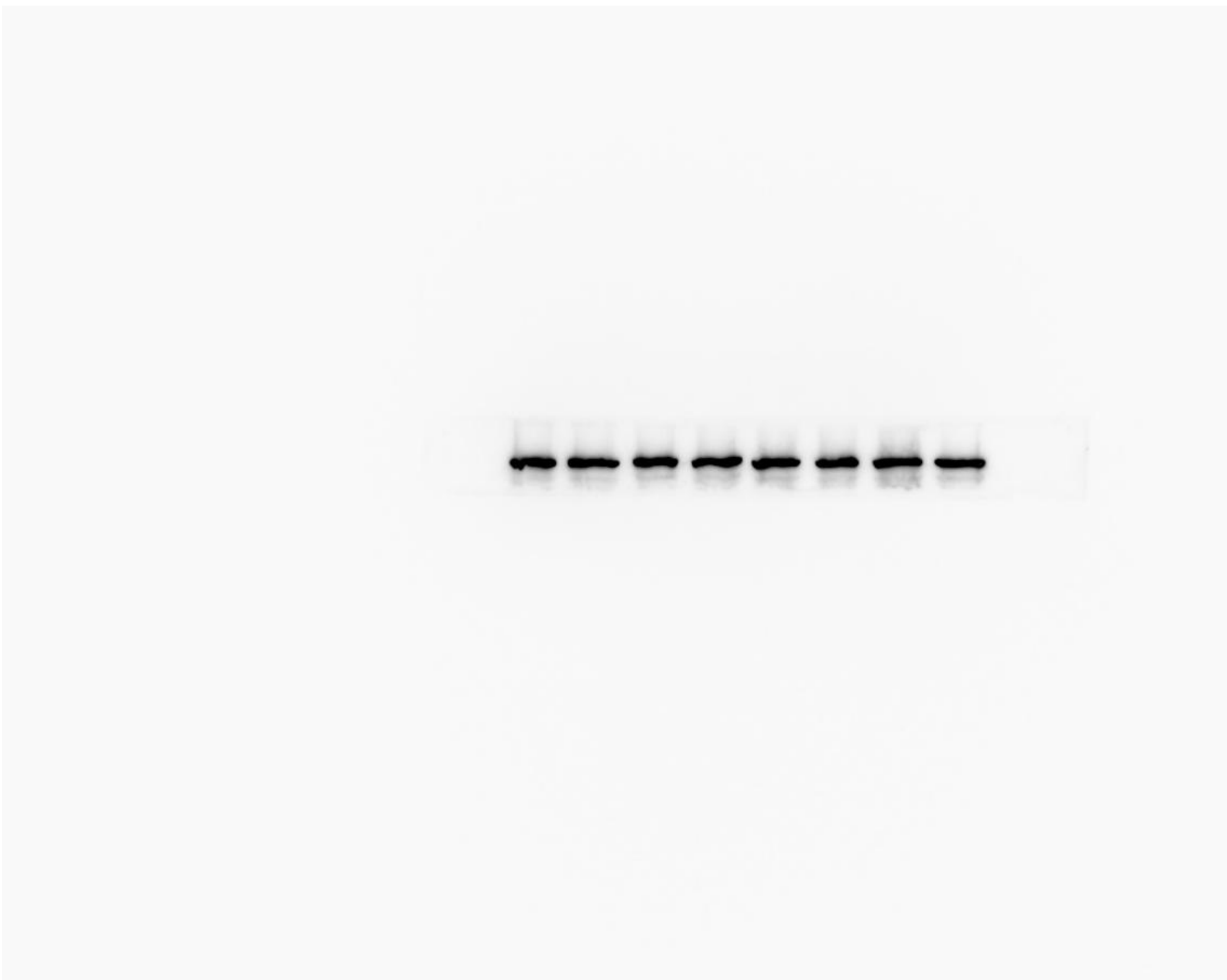

Fig5E

Ub-IP

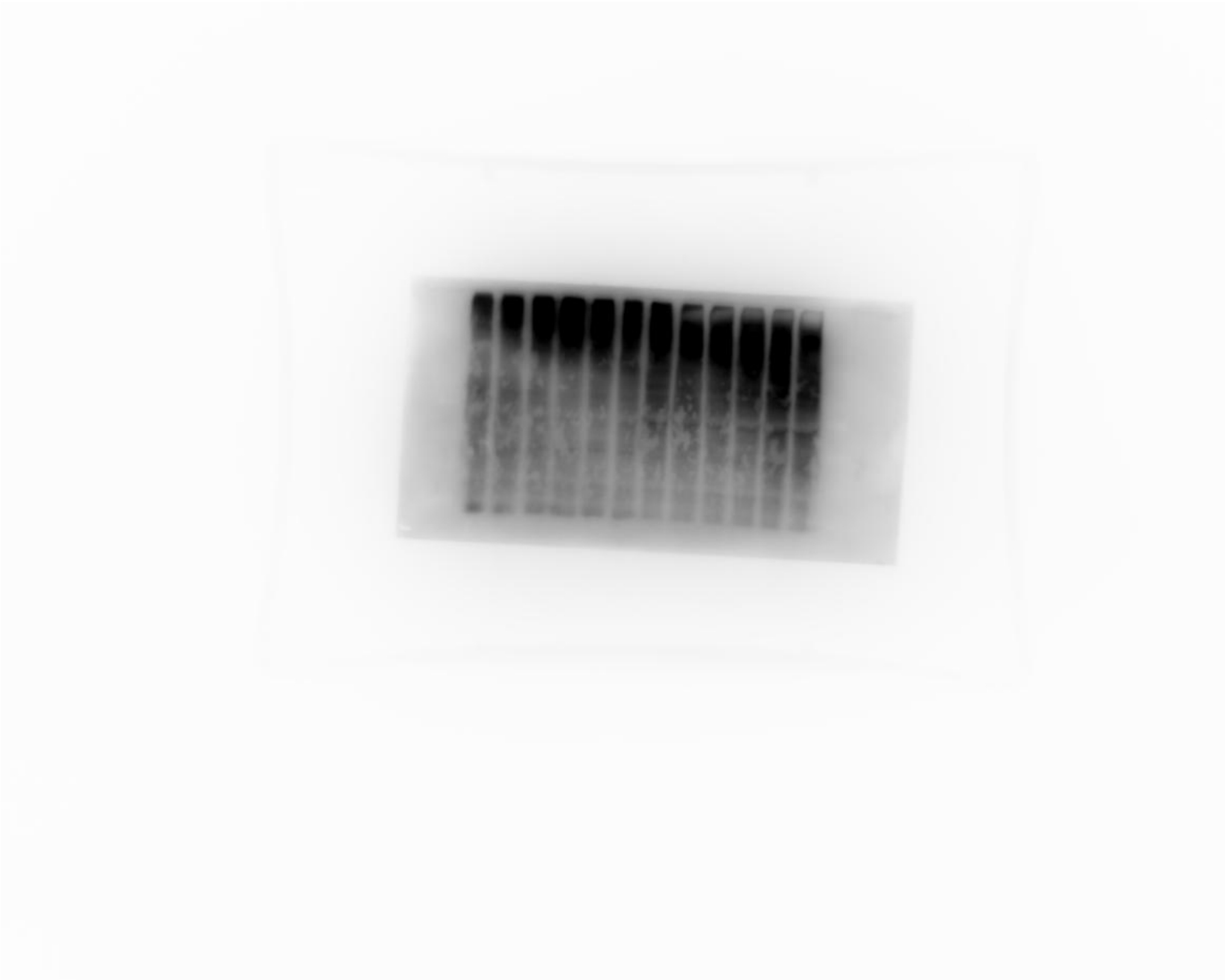

MED12-IP

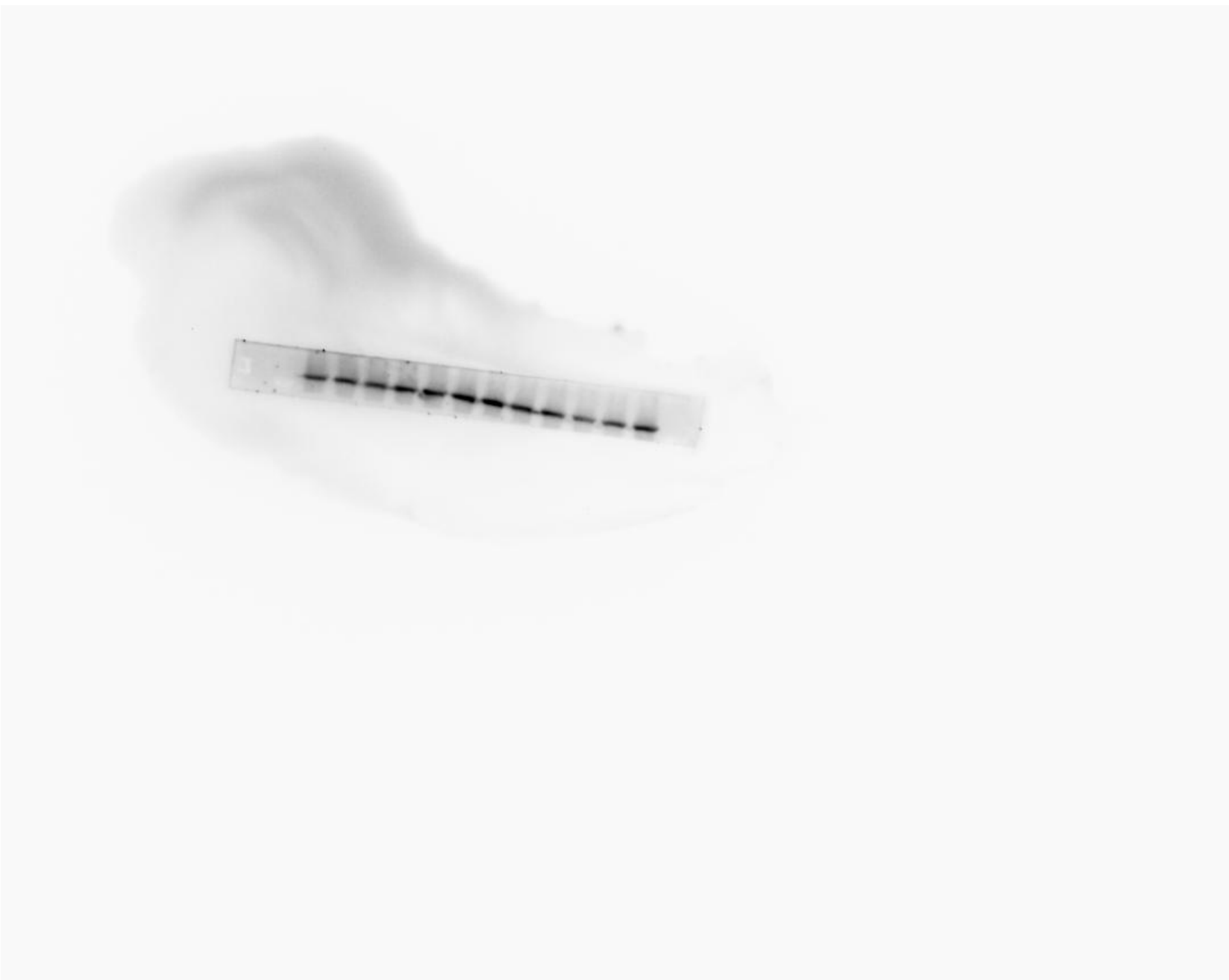

MED12-Input

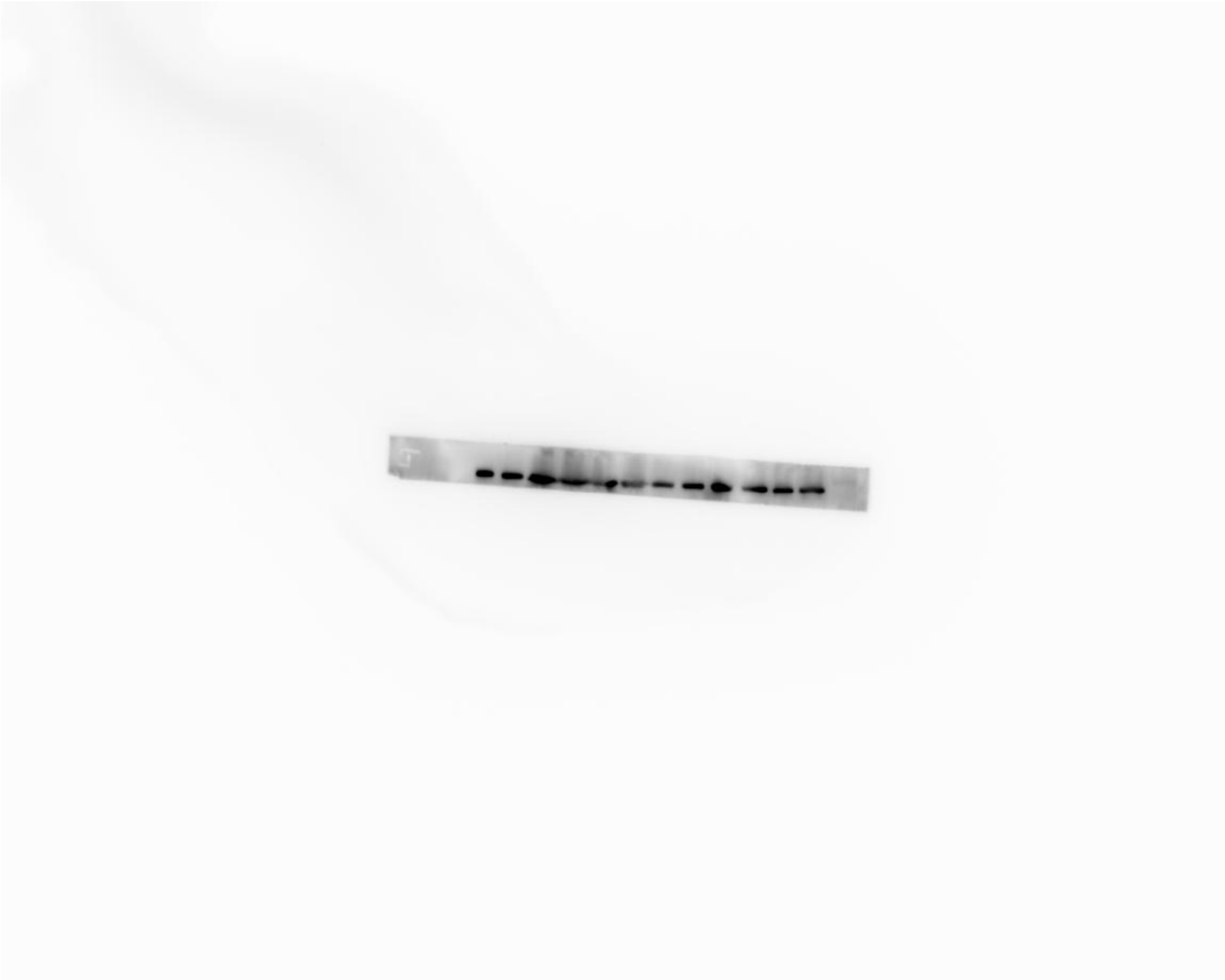

GAPDH

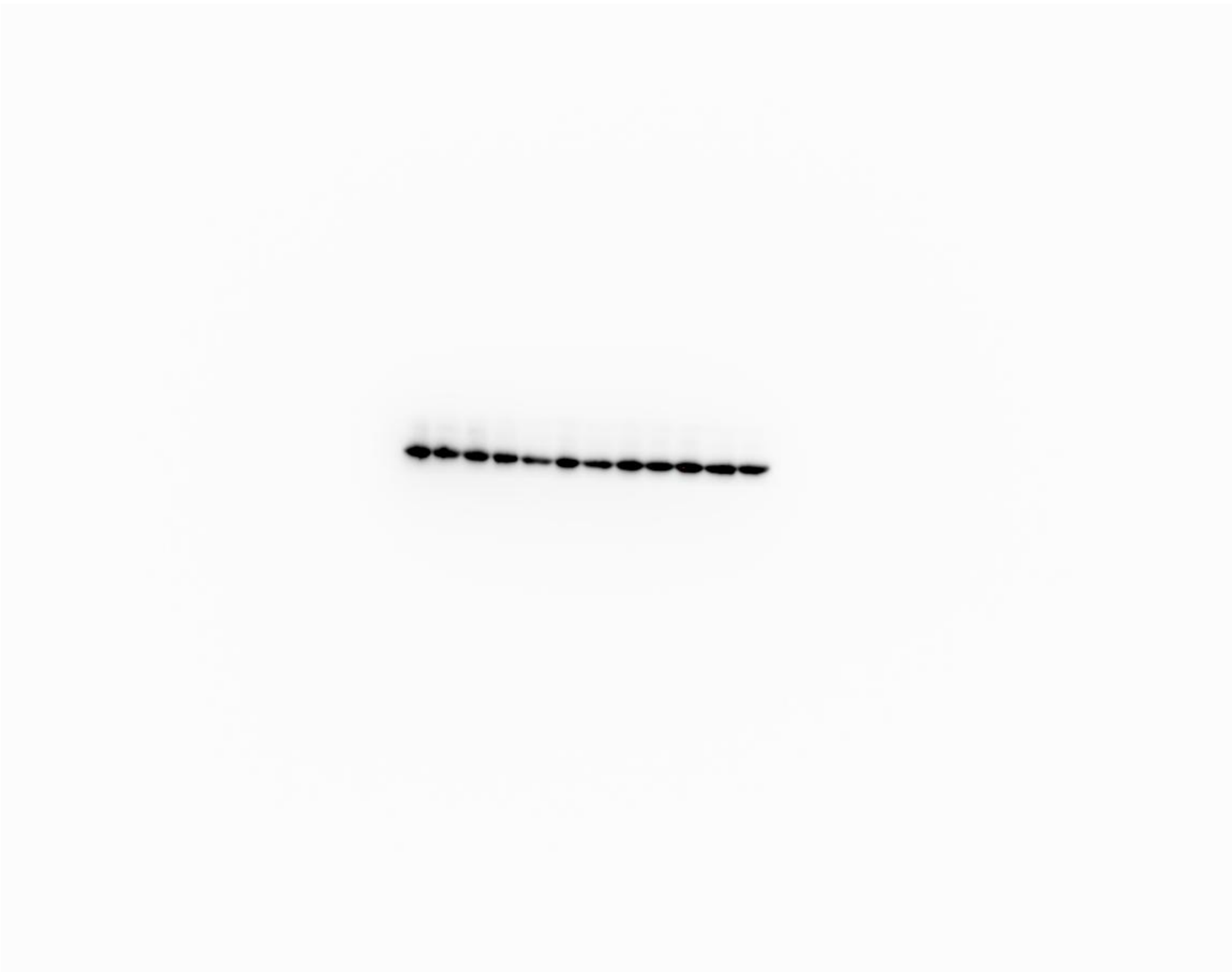

Fig5F

HA-Ub-IP

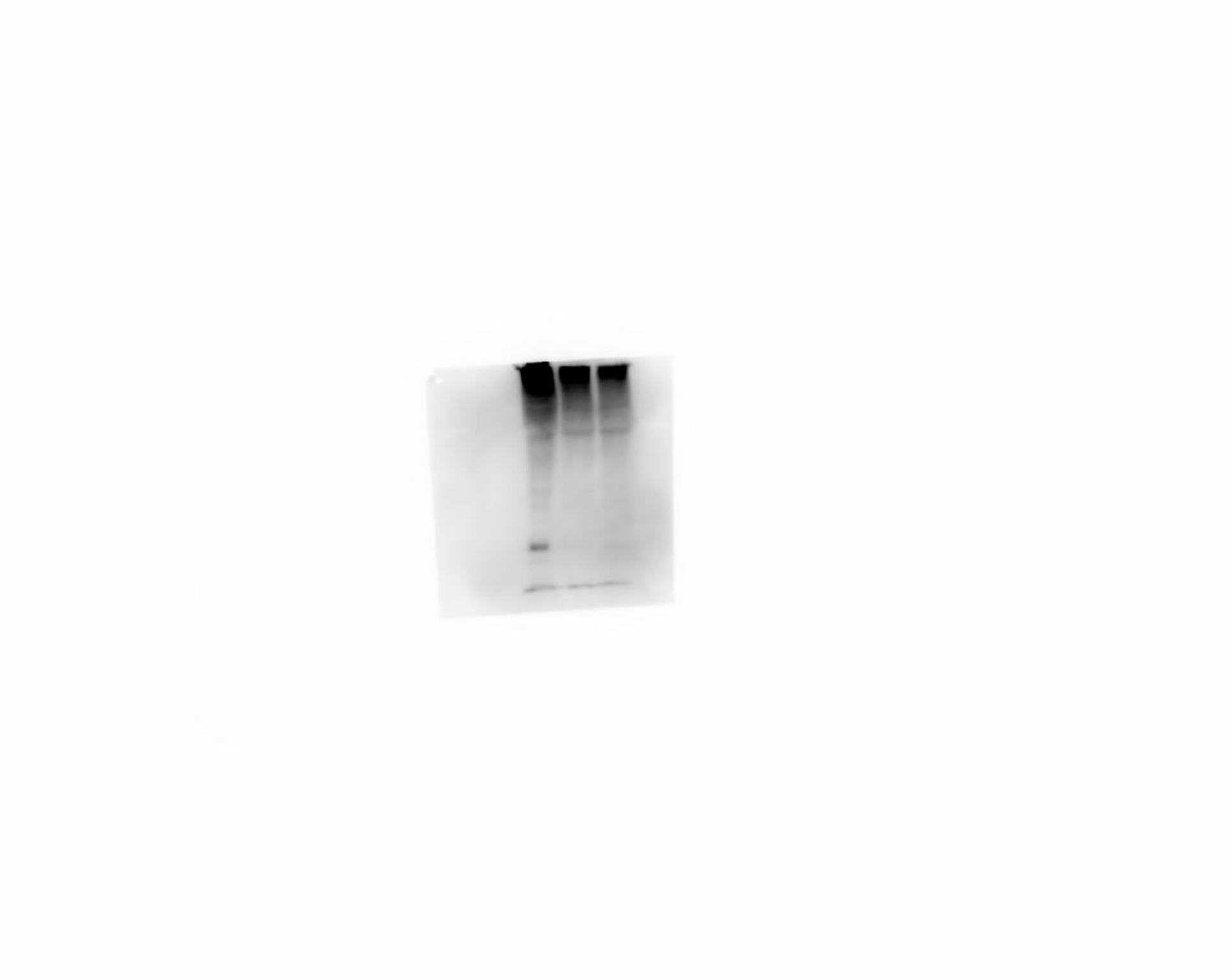

MED12-IP

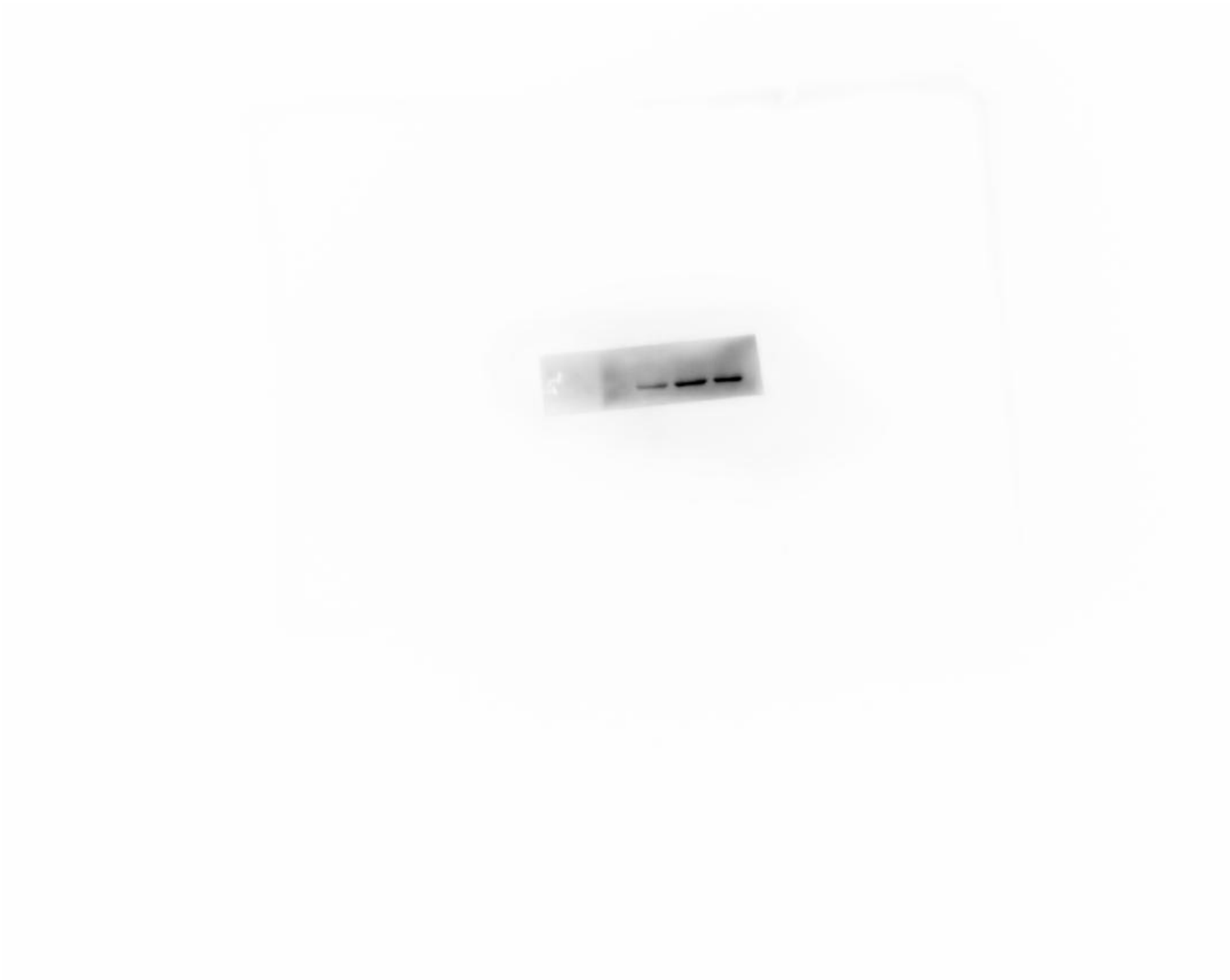

HA-Ub-Input

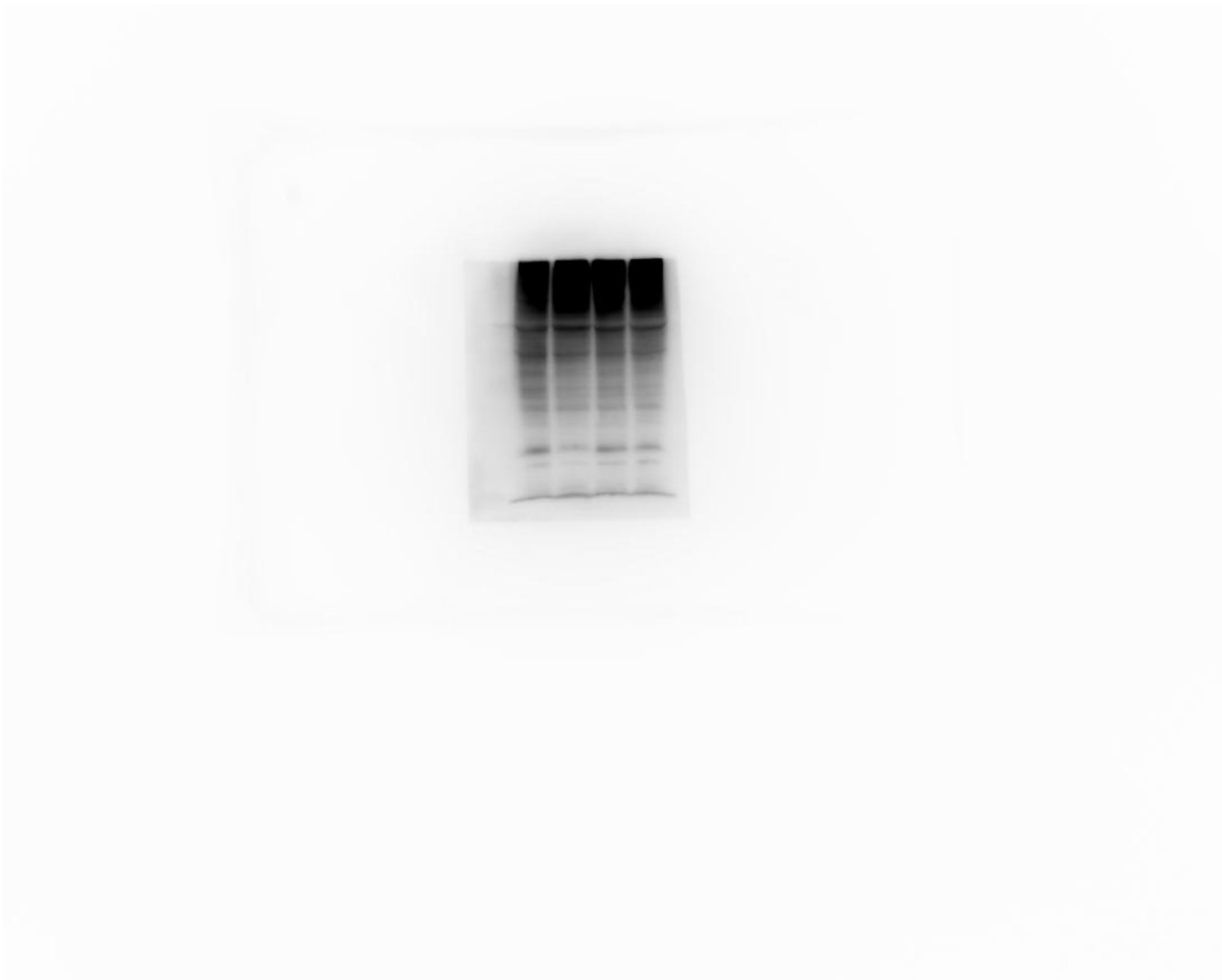

MED12-Input

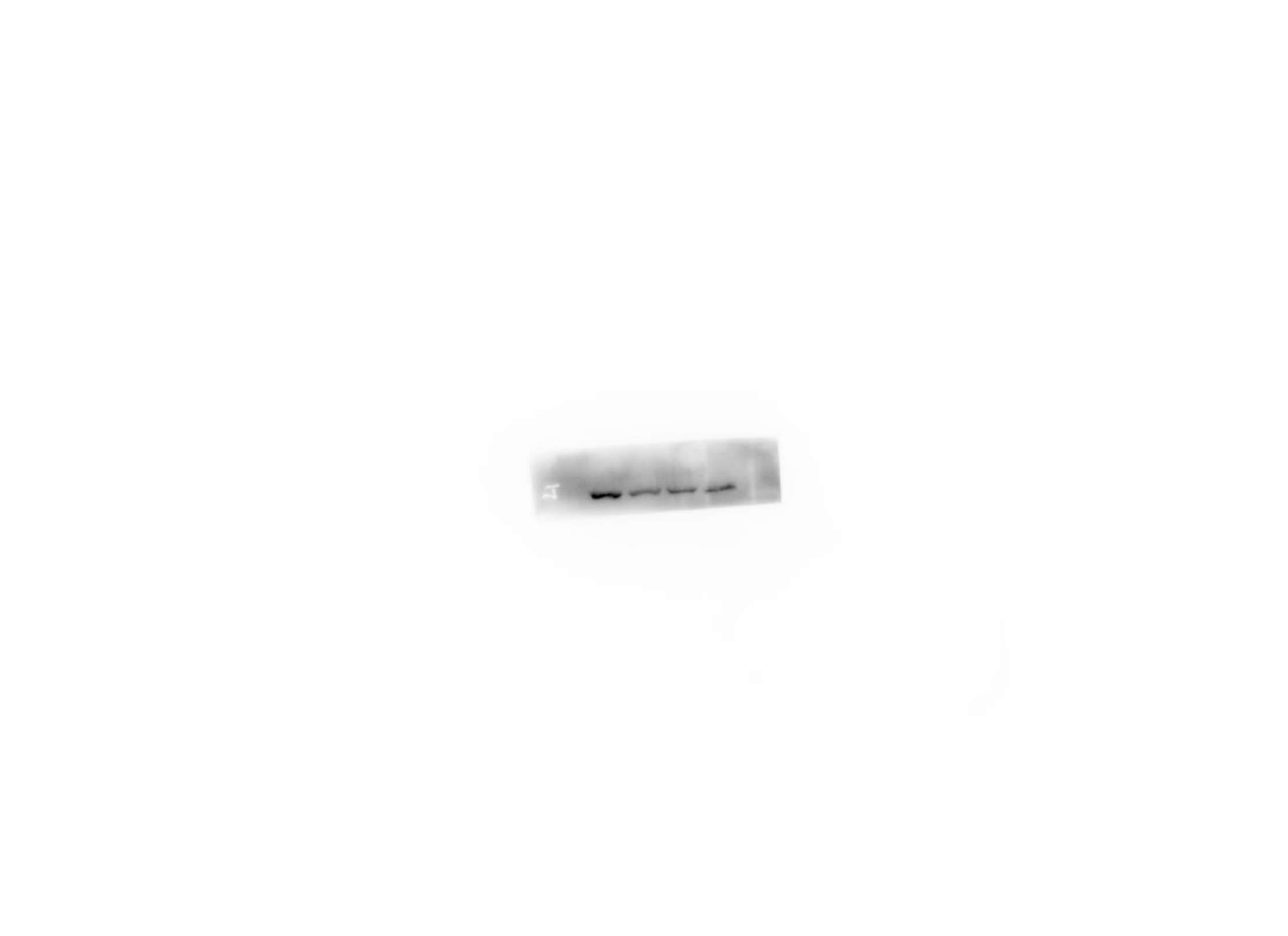

FBXO7-Input

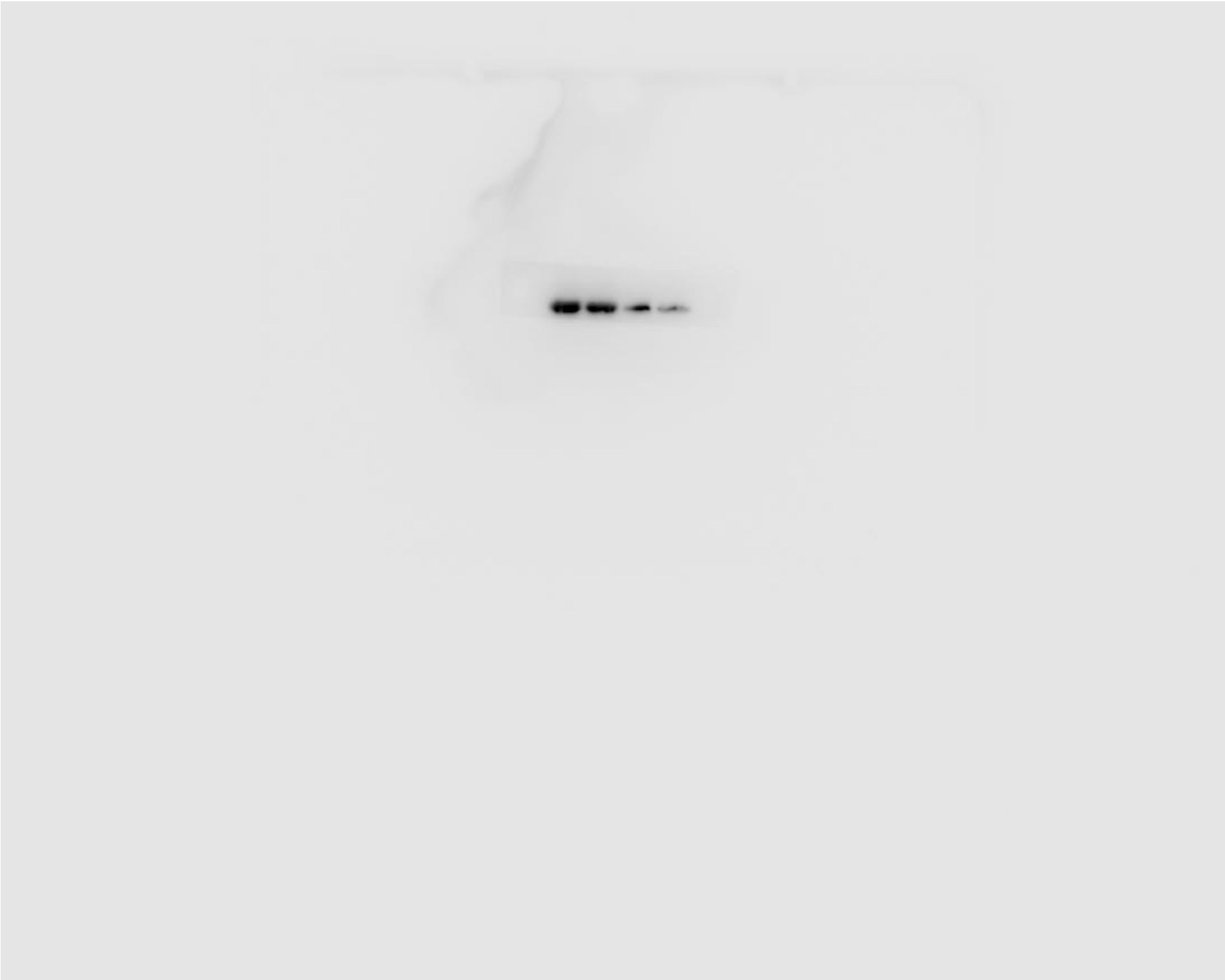

GAPDH

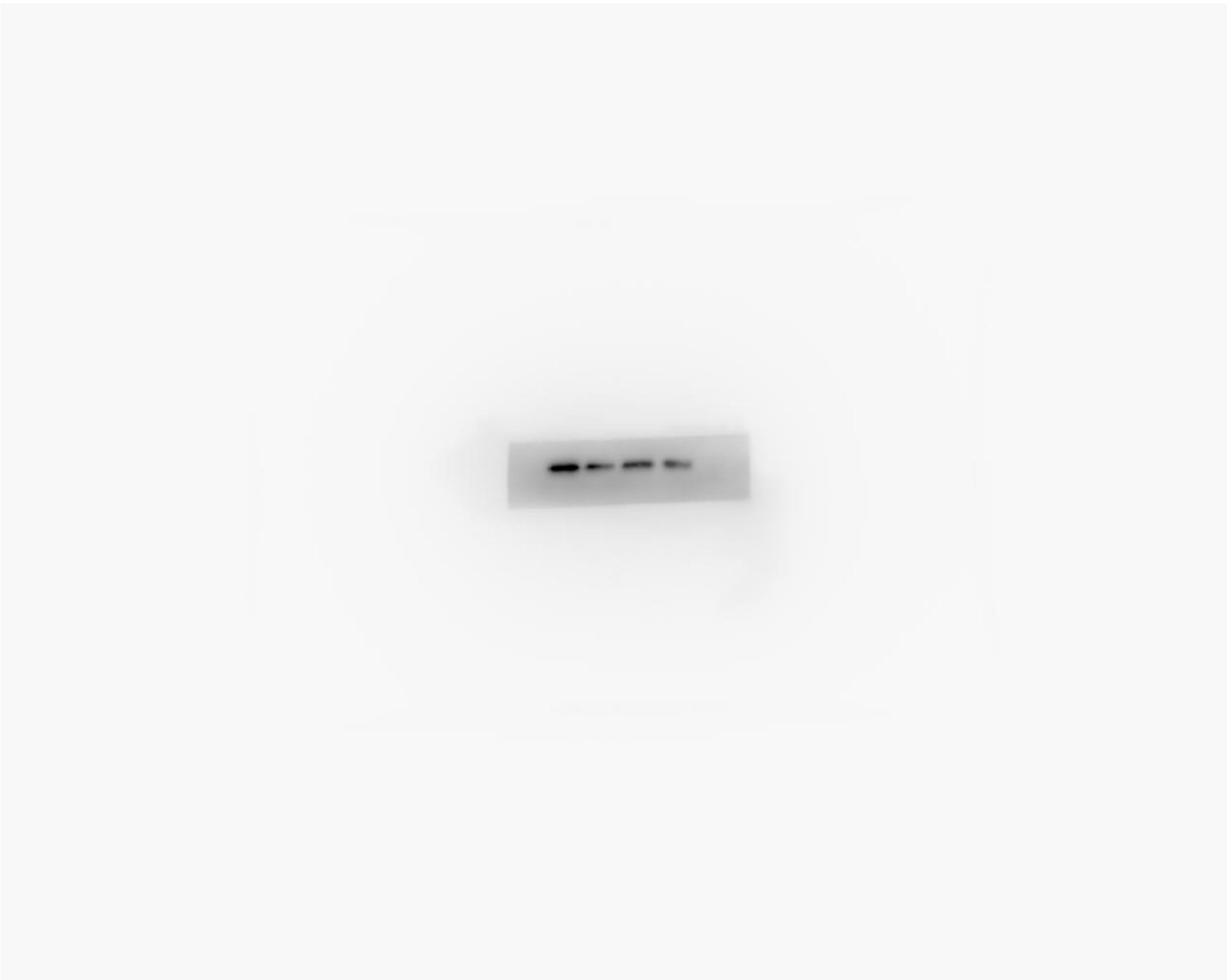

Fig5G

HA-Ub-IP

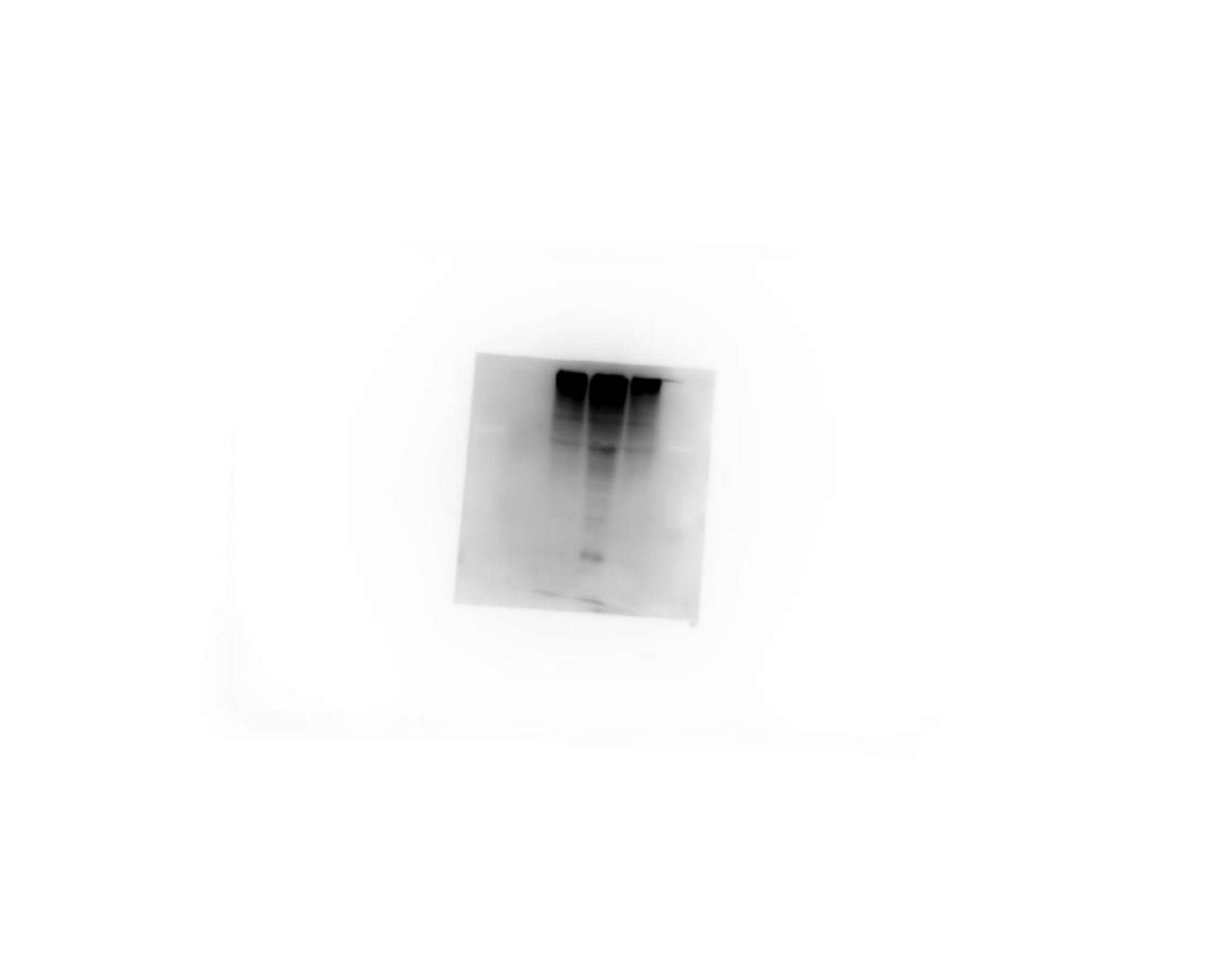

MED12-IP

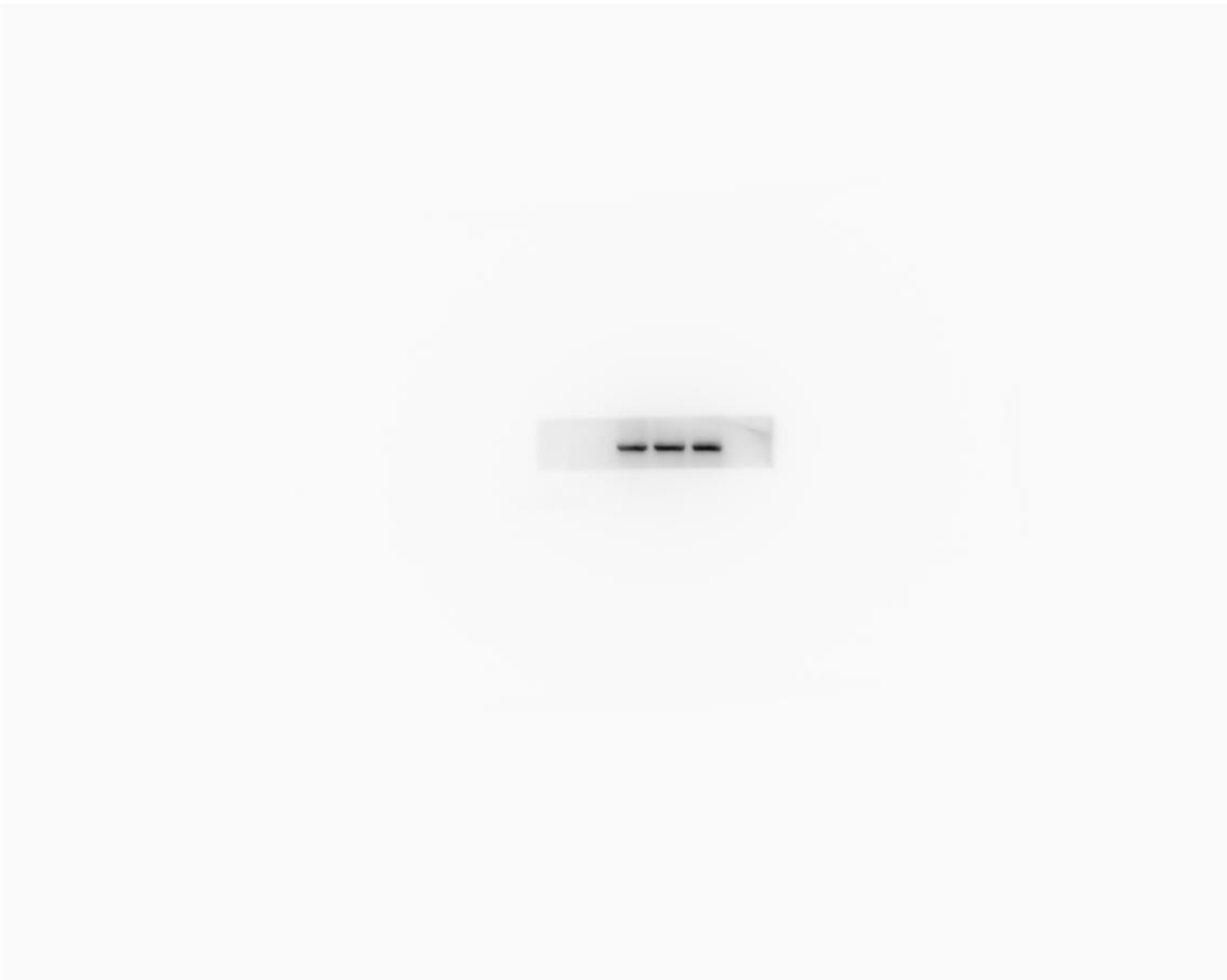

HA-Ub-Input

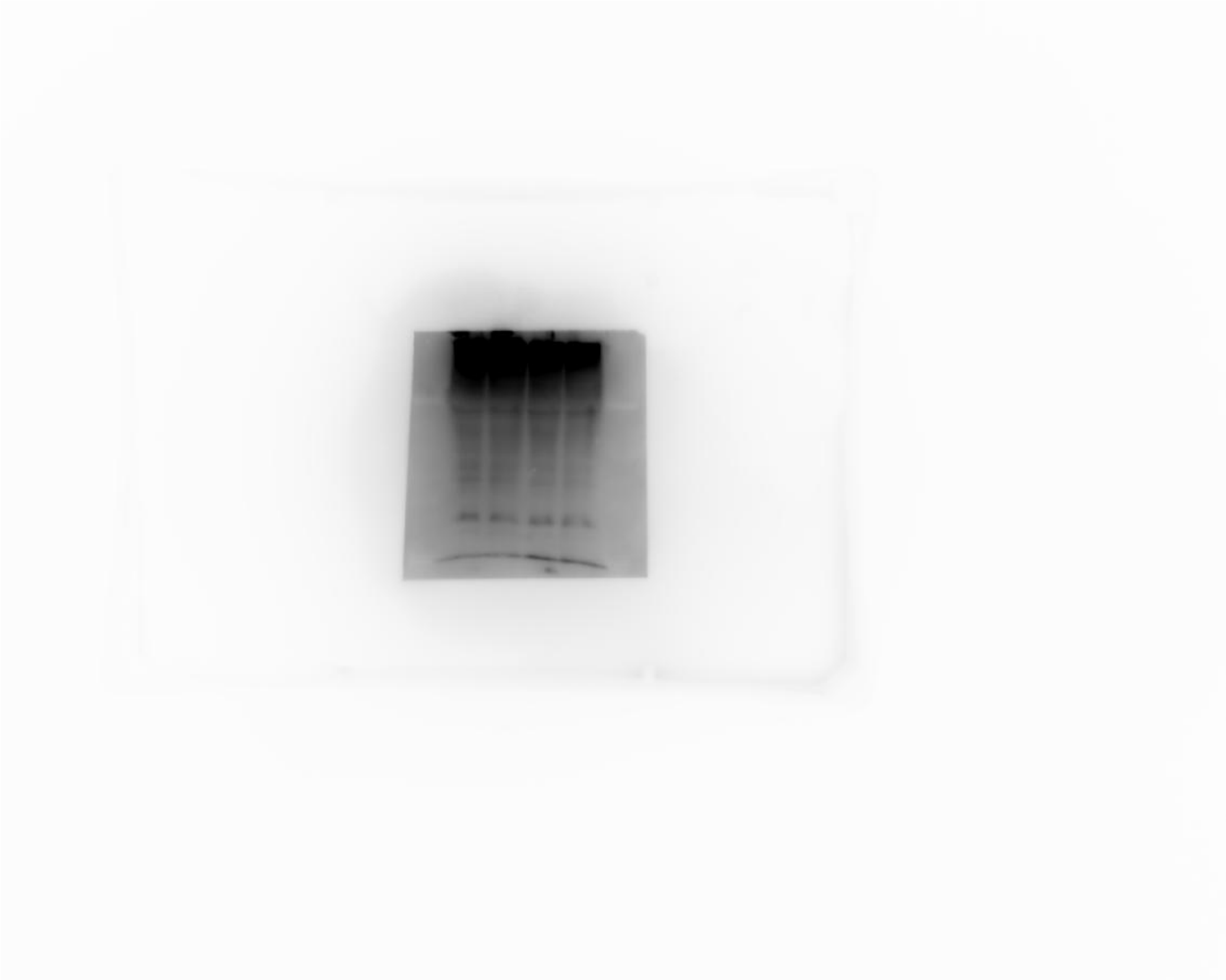

MED12-Input

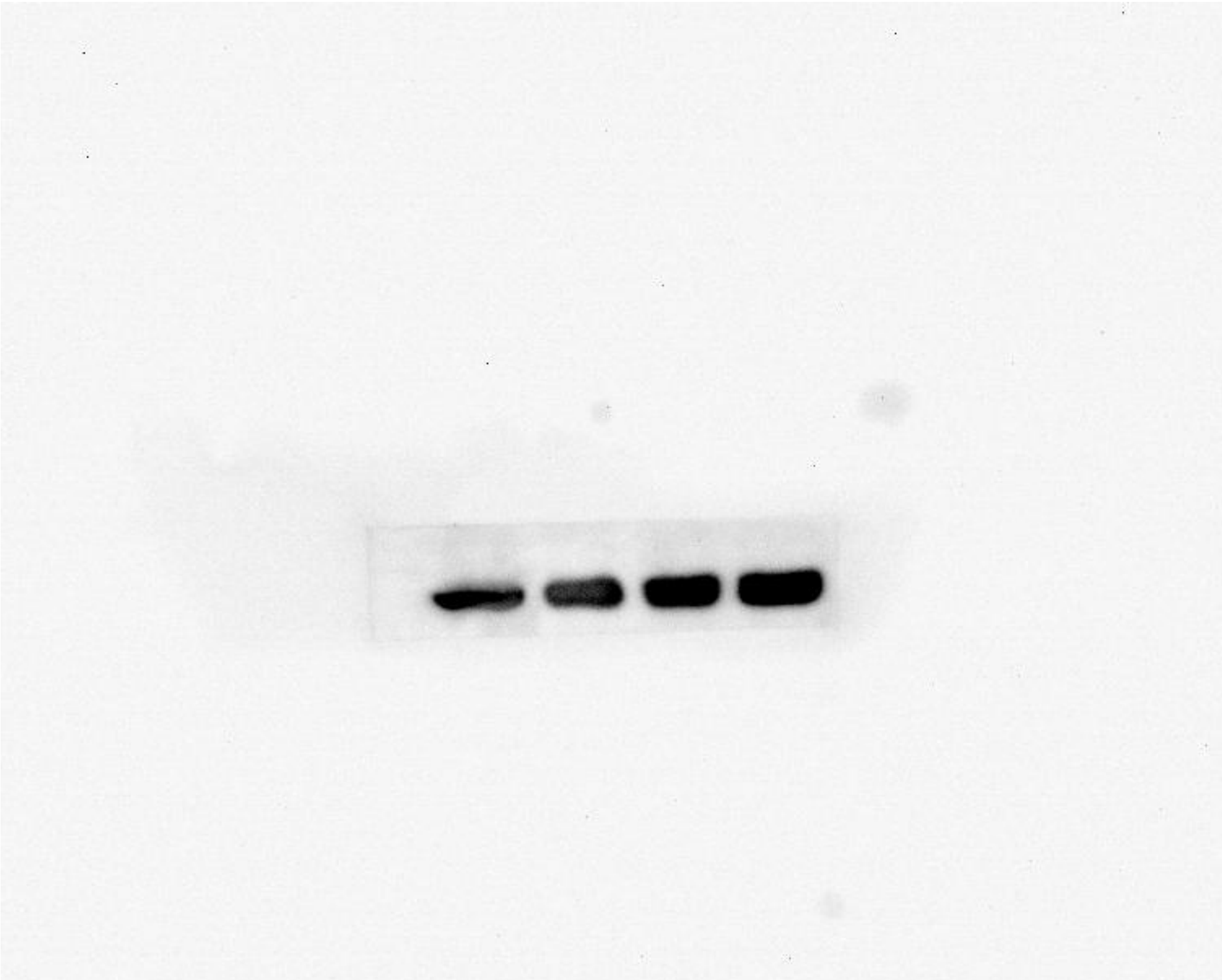

GFP-Input

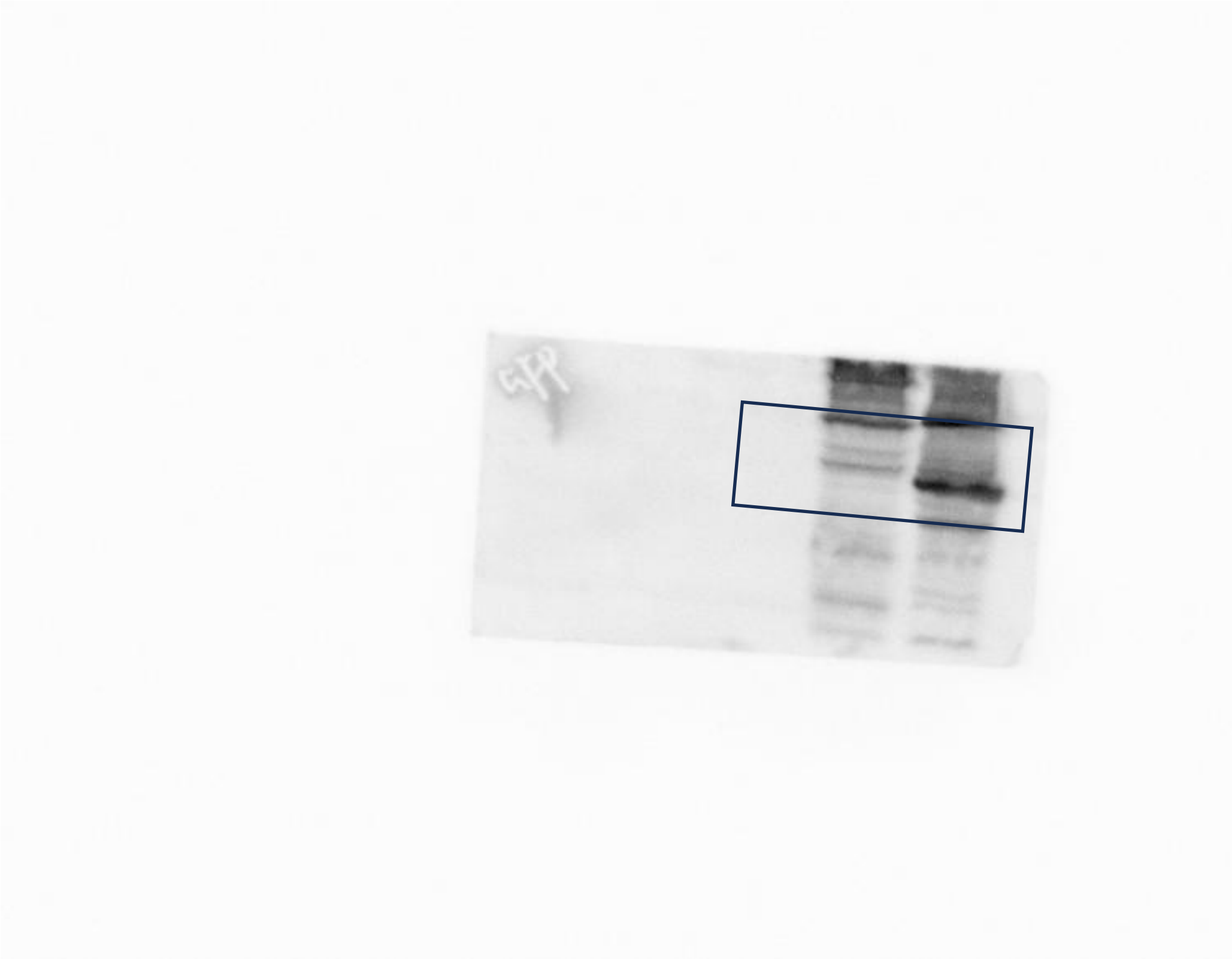

GAPDH

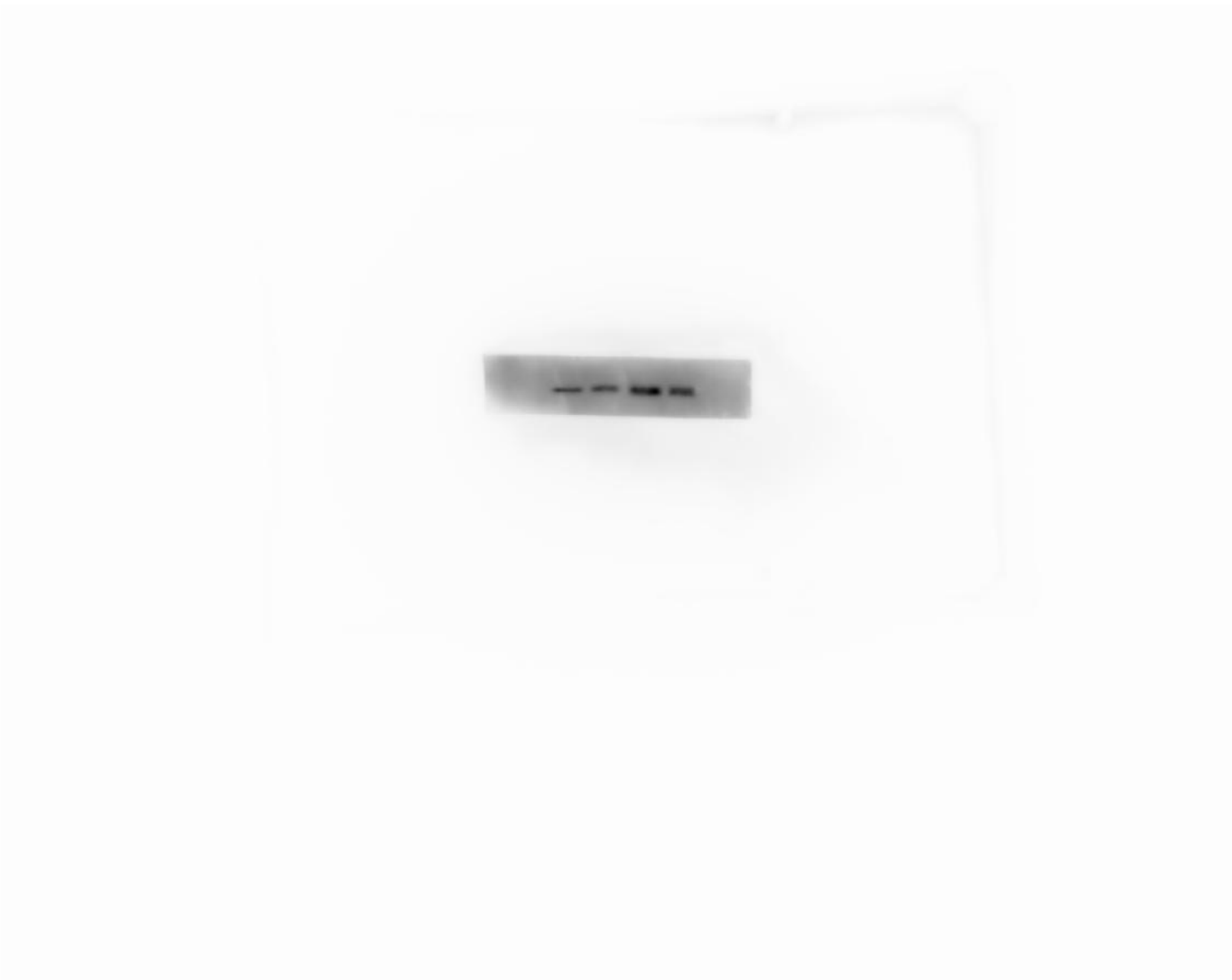

Fig5H

HA-Ub-IP

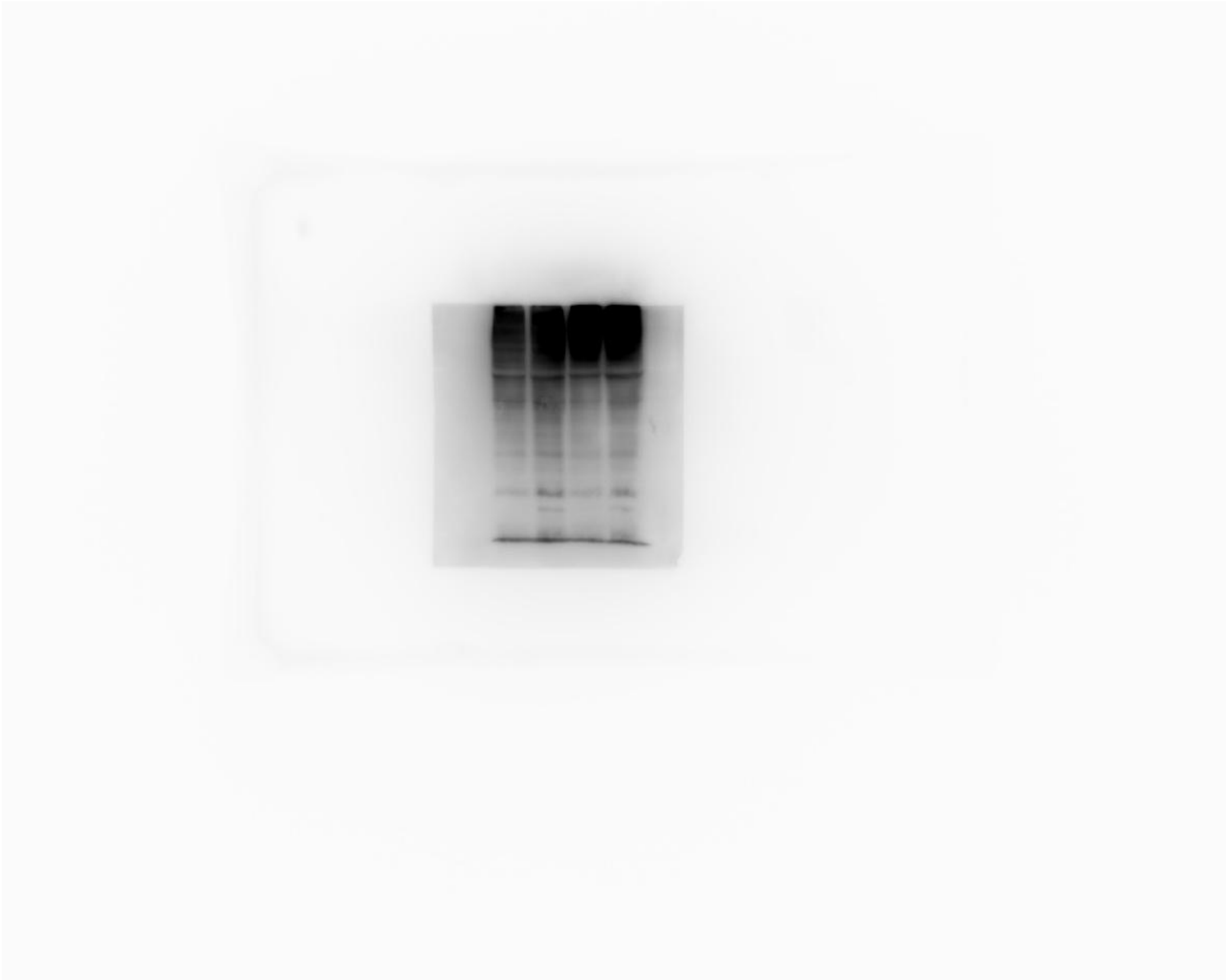

Flag-IP

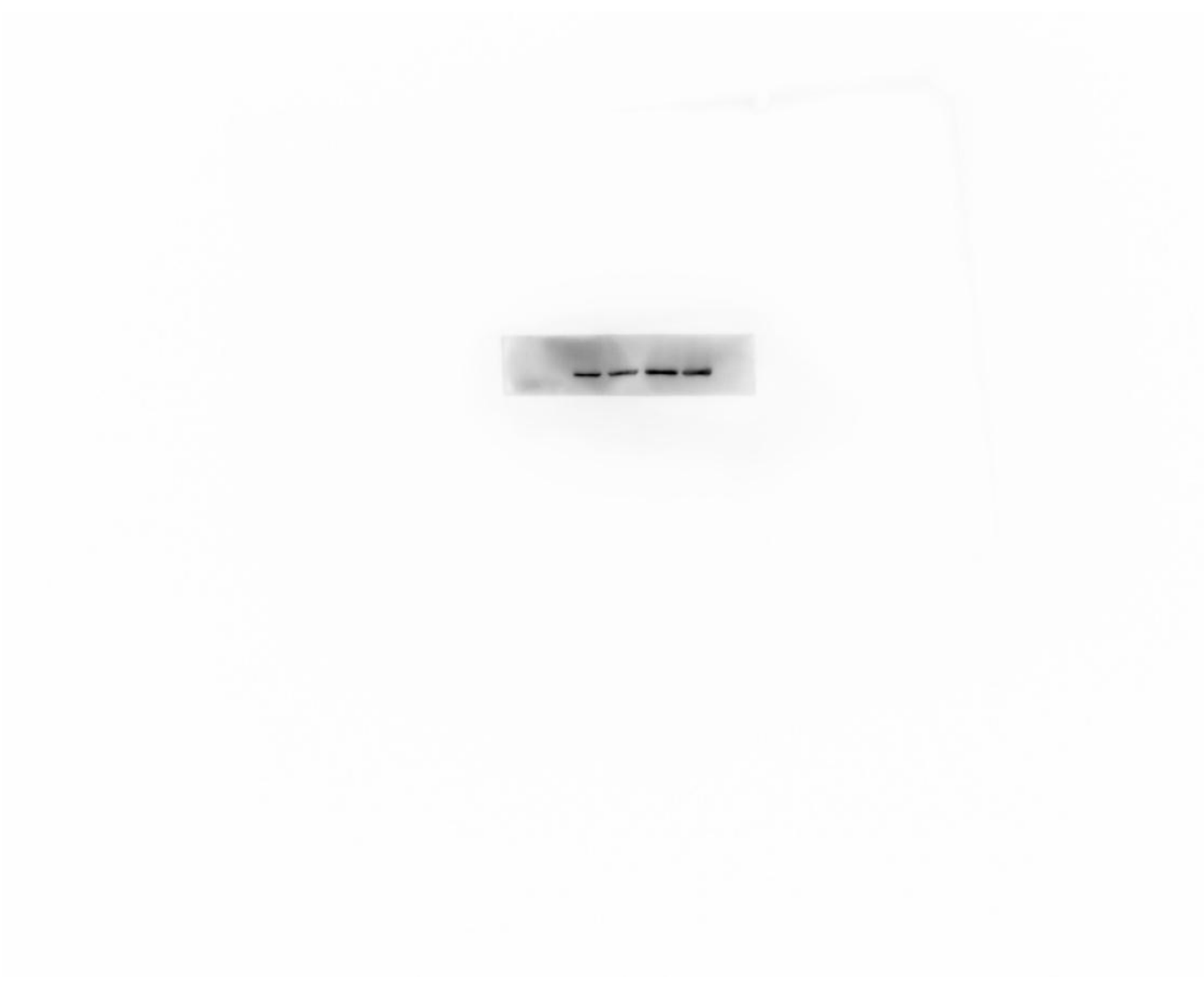

HA-Ub-Input

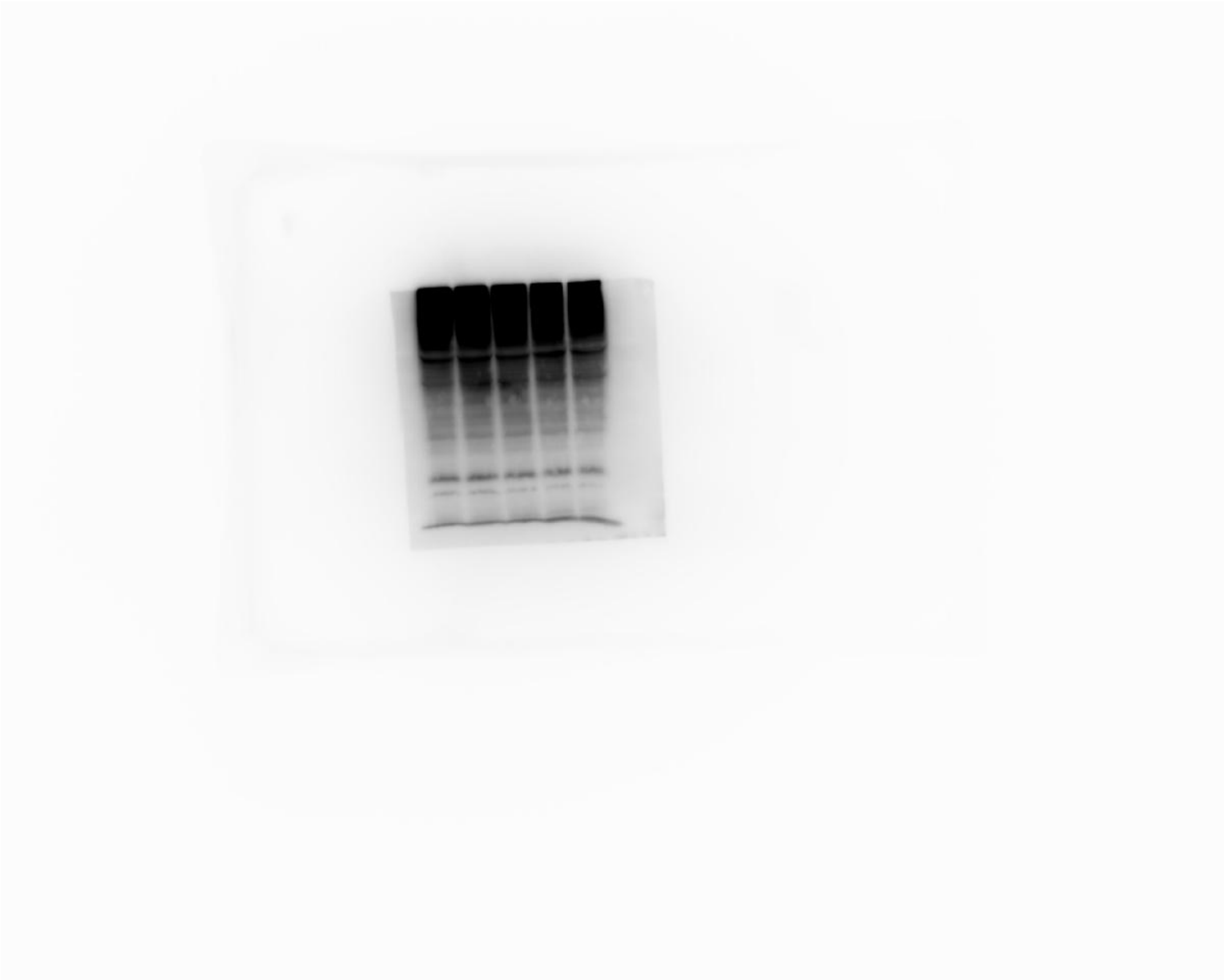

Flag-Input

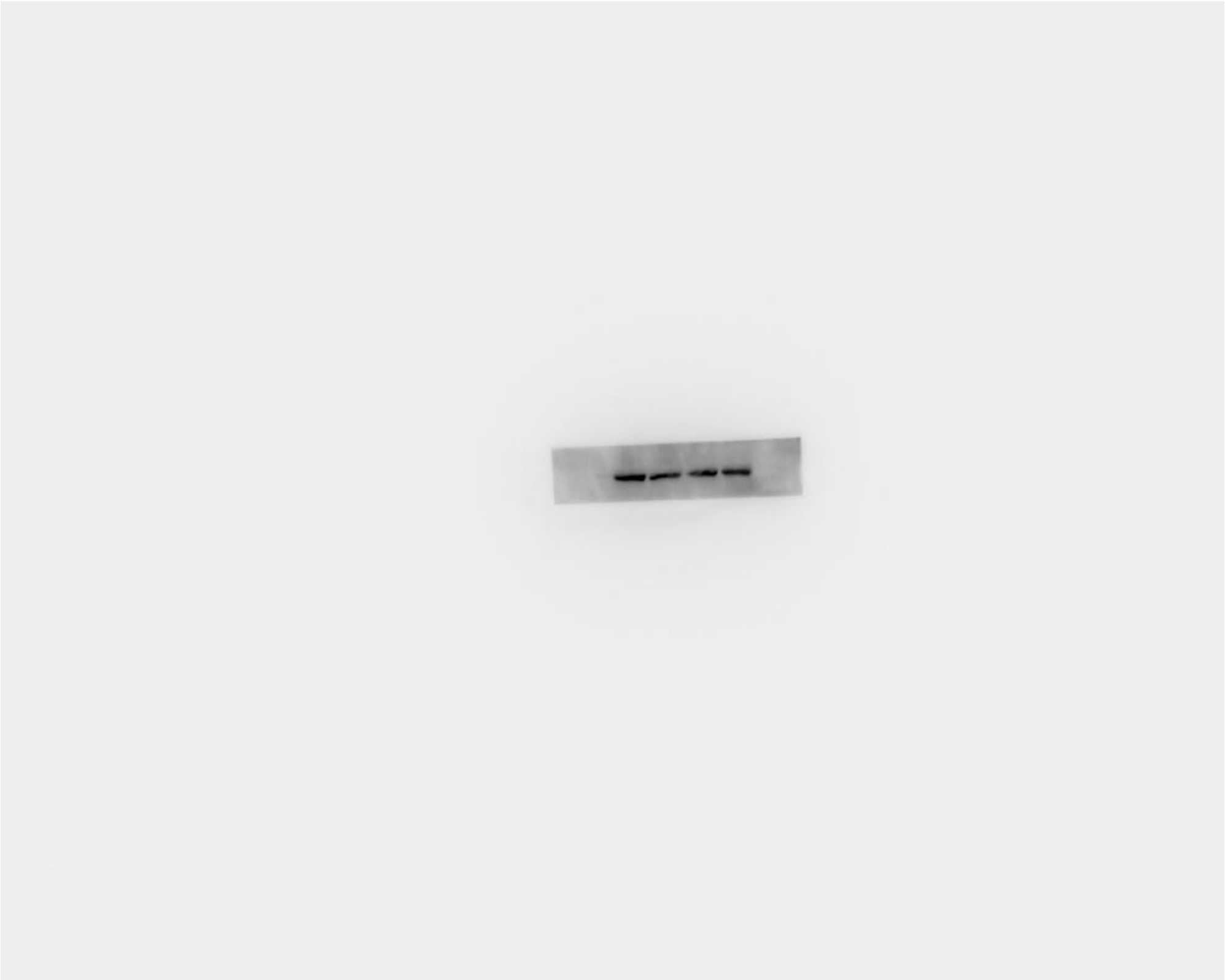

GFP

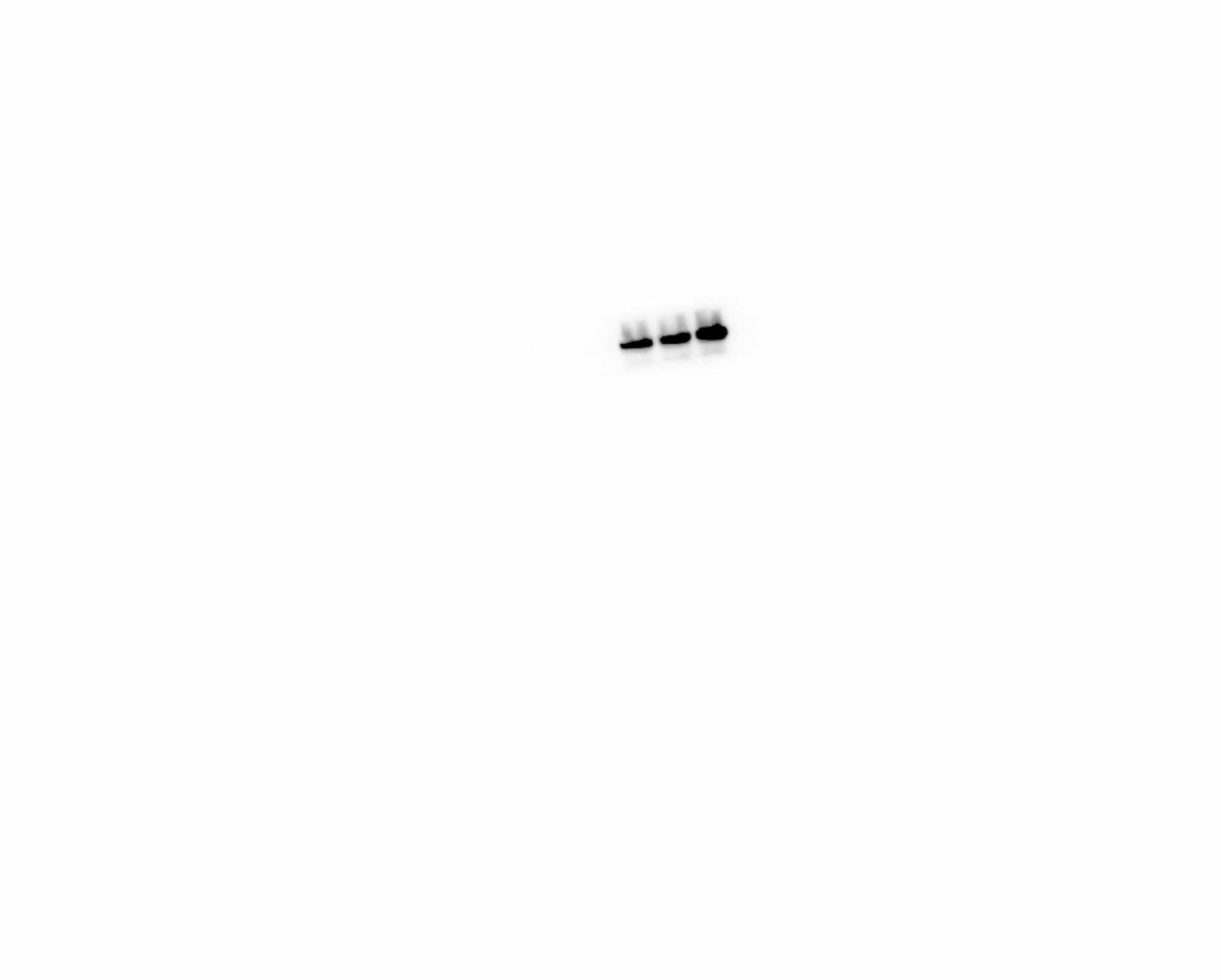

GAPDH

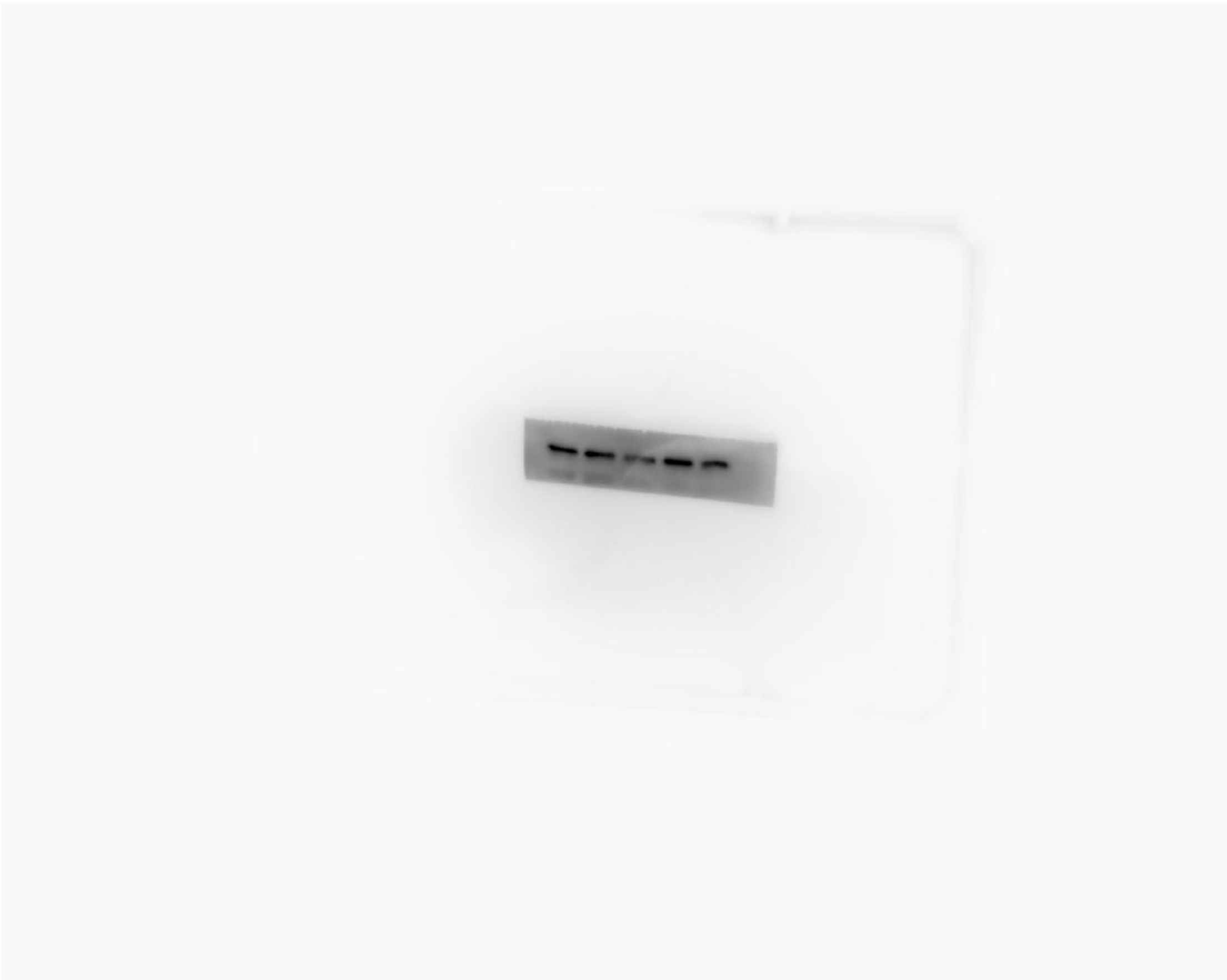

Fig5I

HA-Ub-IP

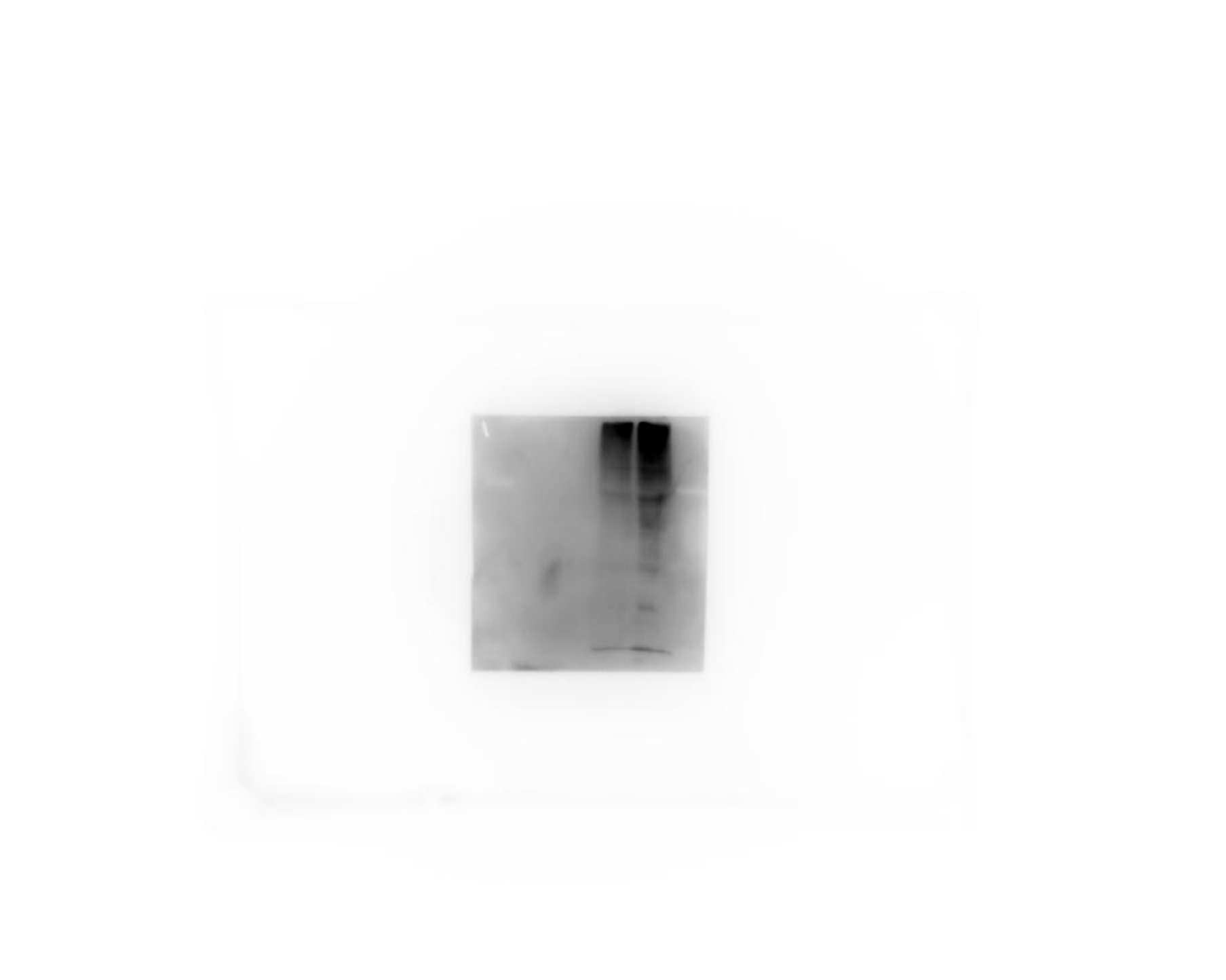

Flag-IP

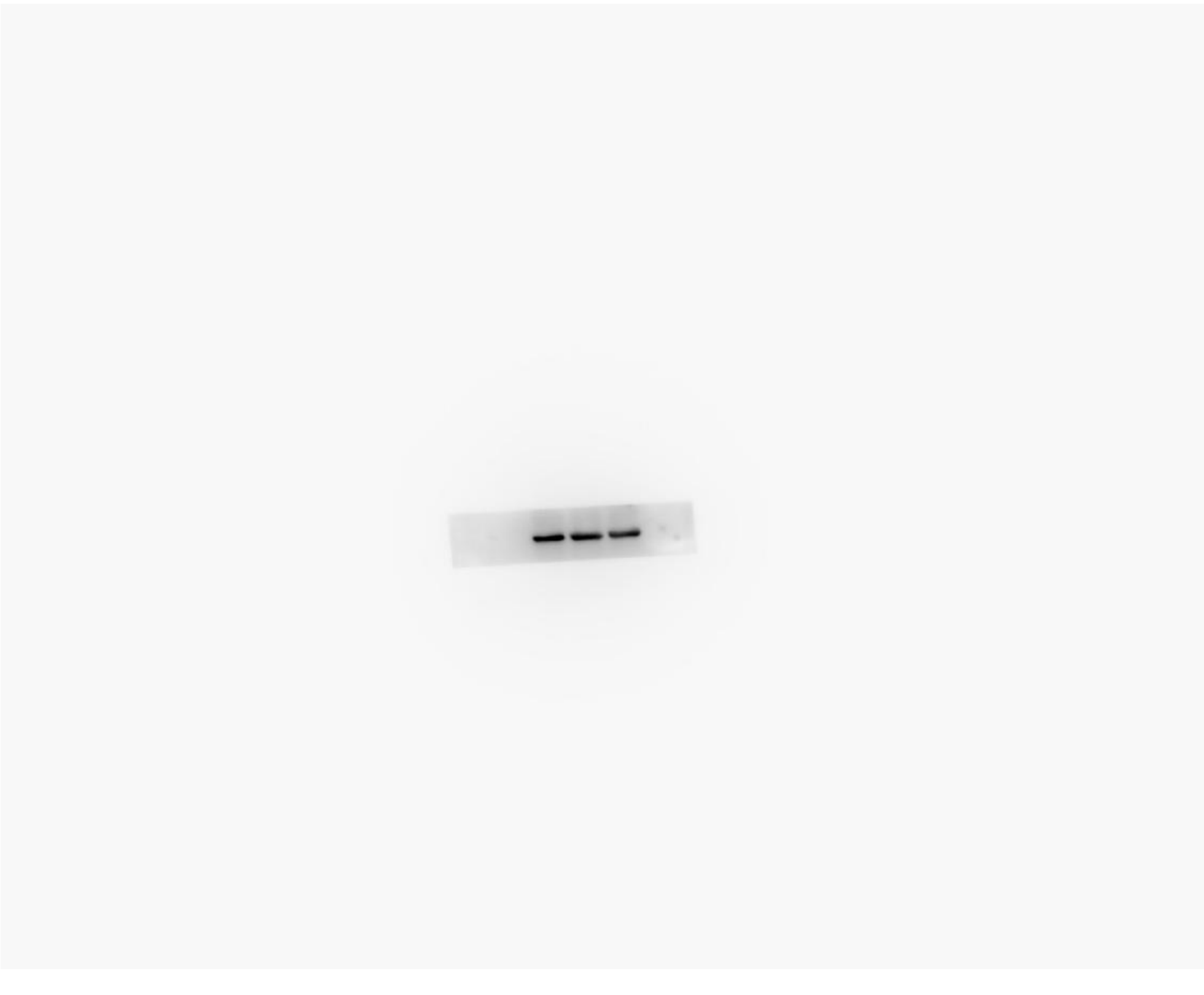

HA-Ub-Input

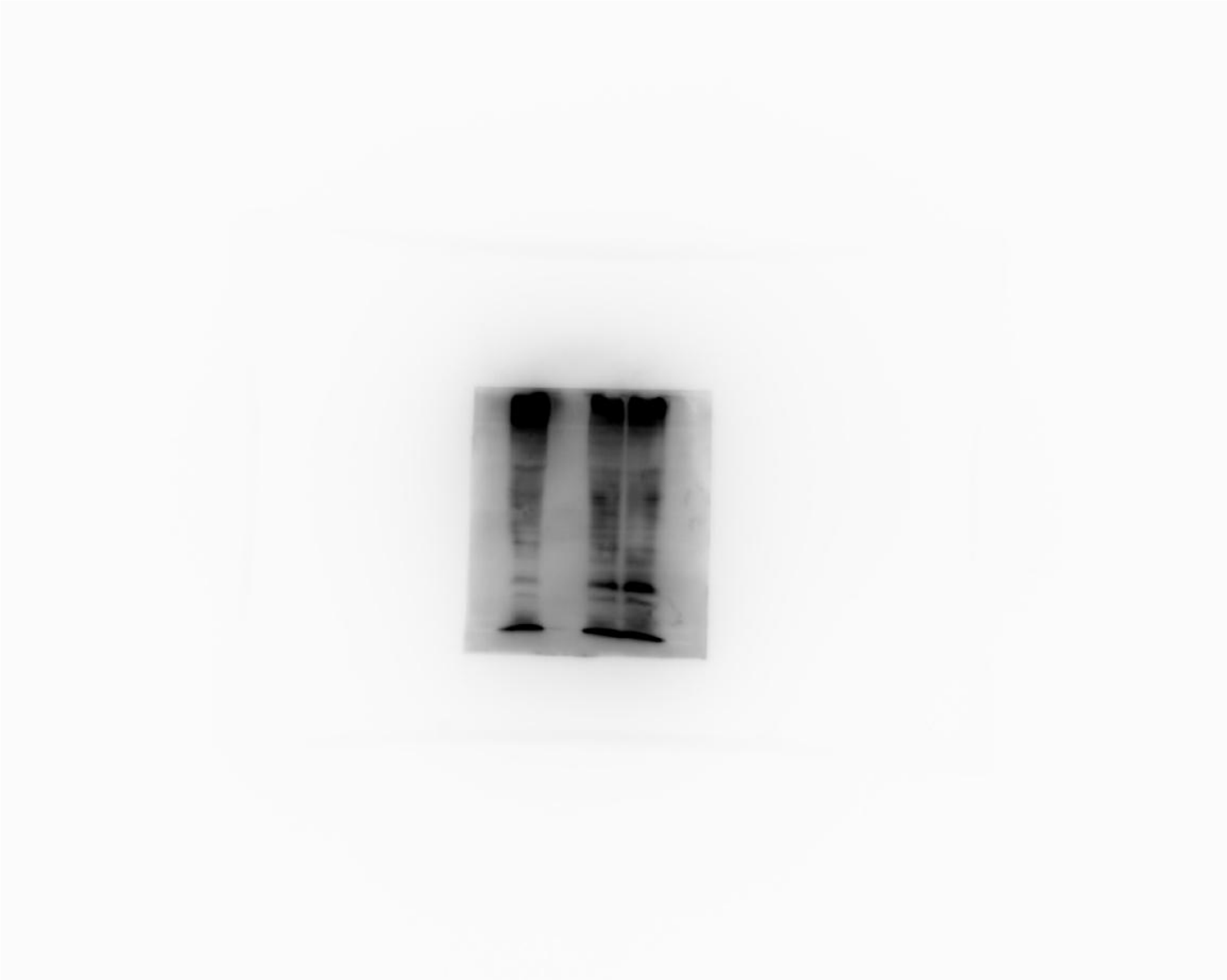

Flag-Input

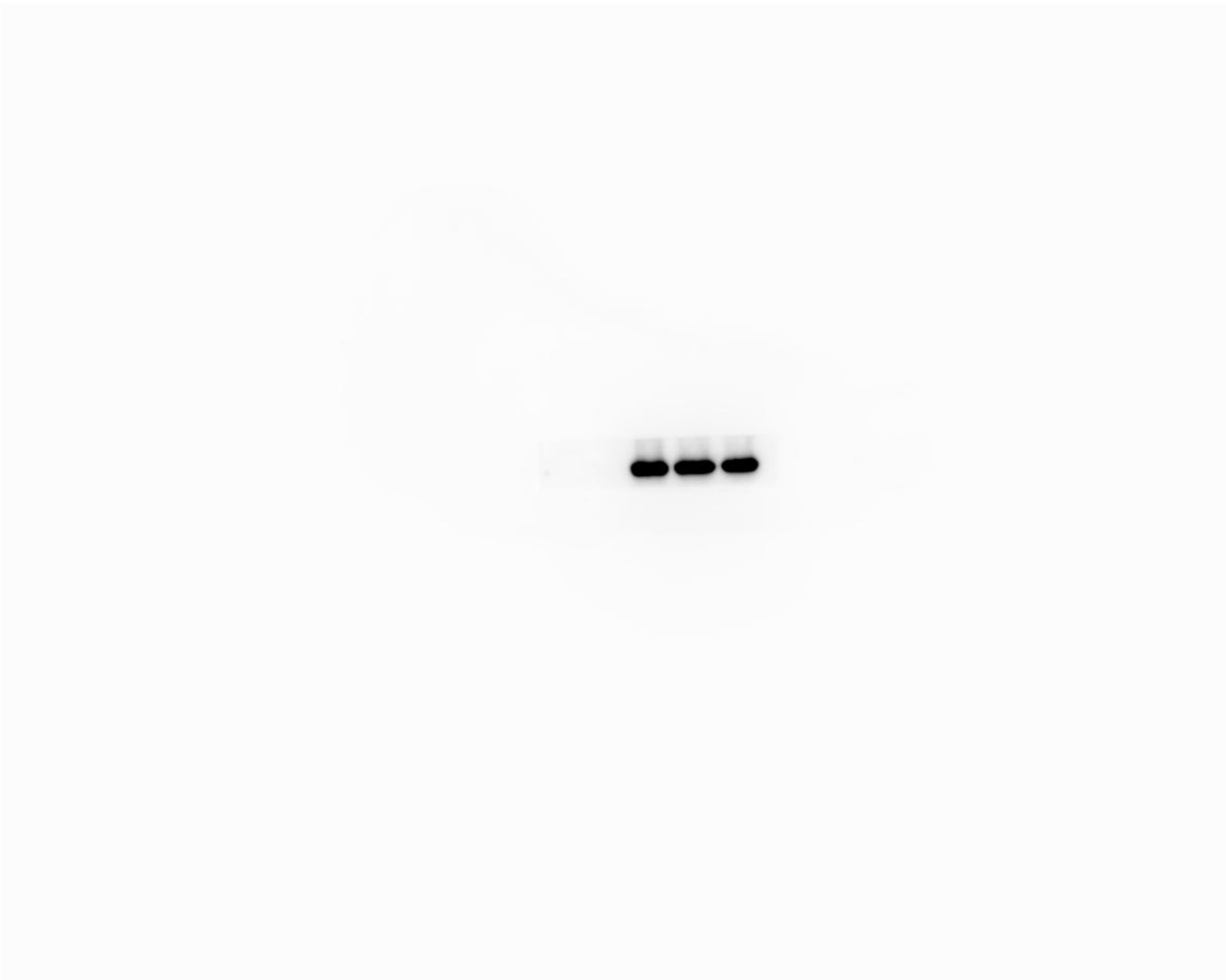

FBXO7

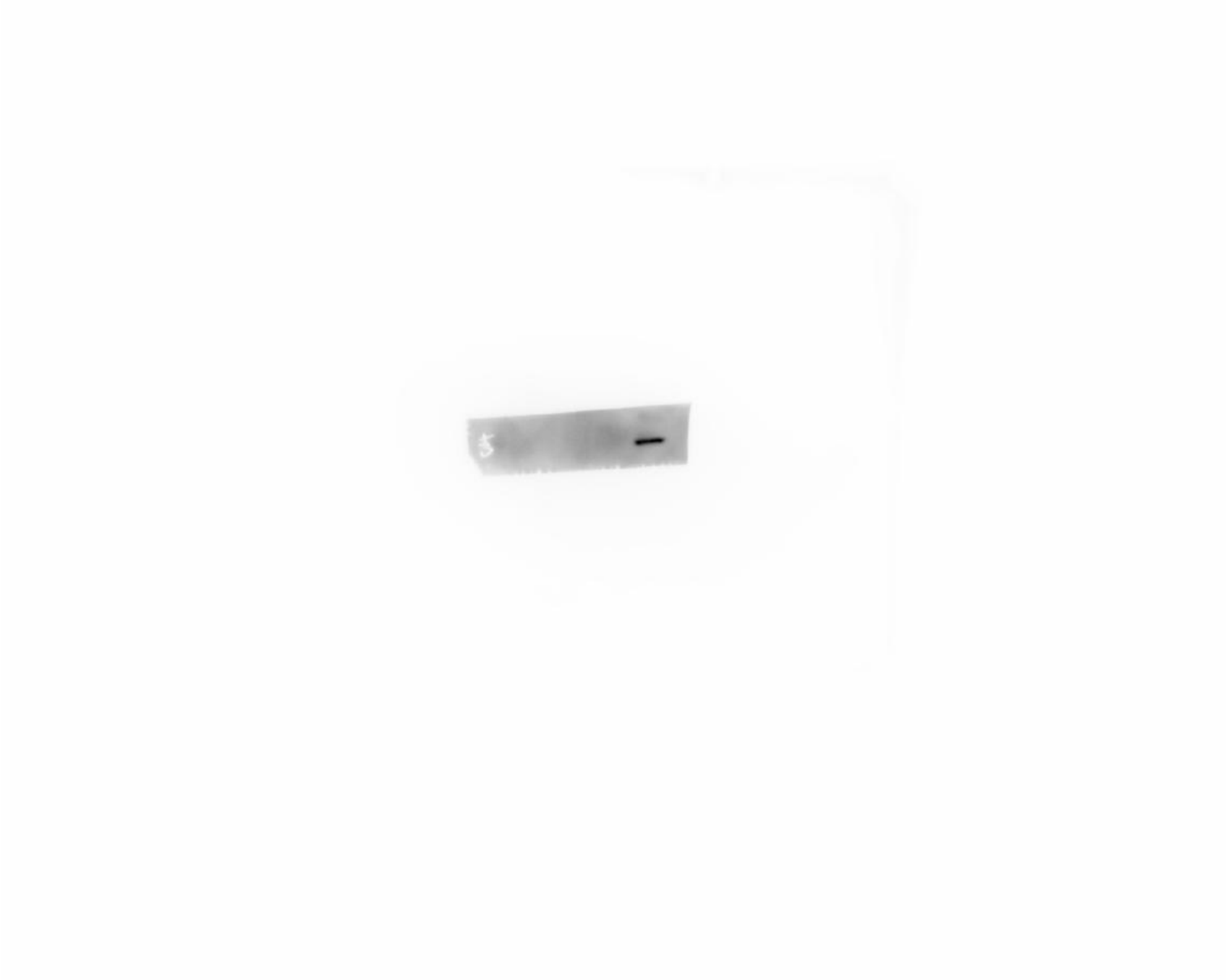

GAPDH

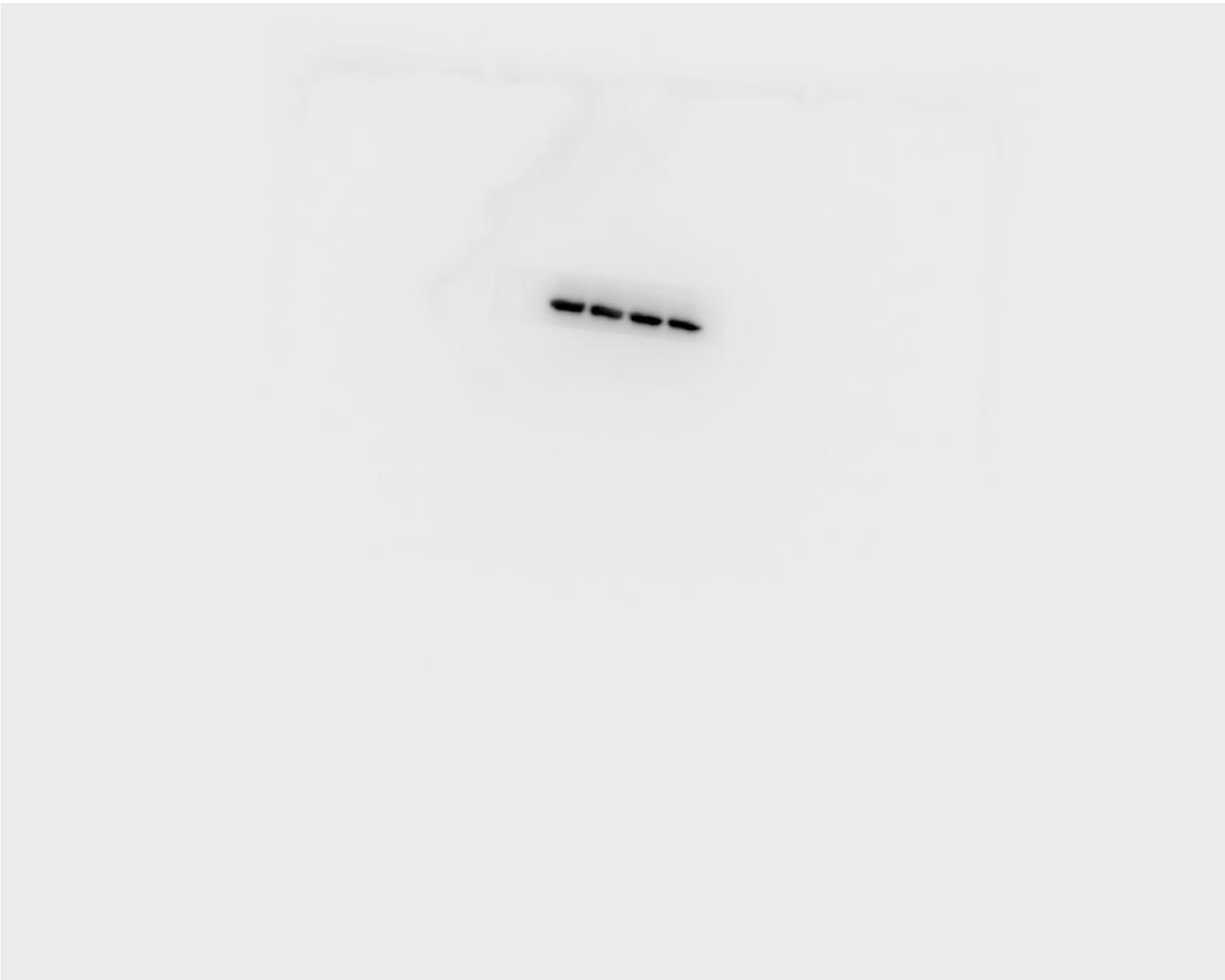

Fig5J

Ub-IP

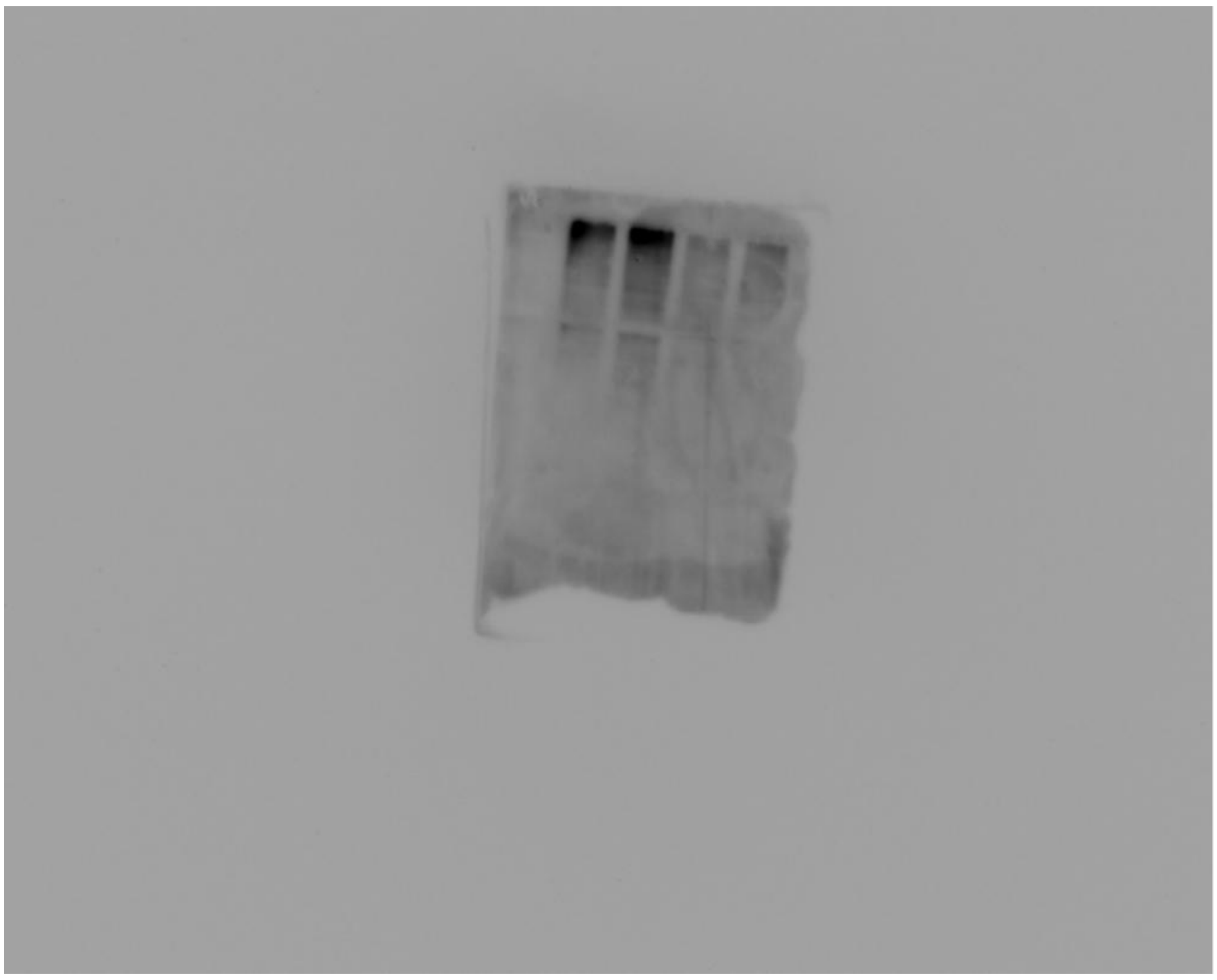

MED12-IP

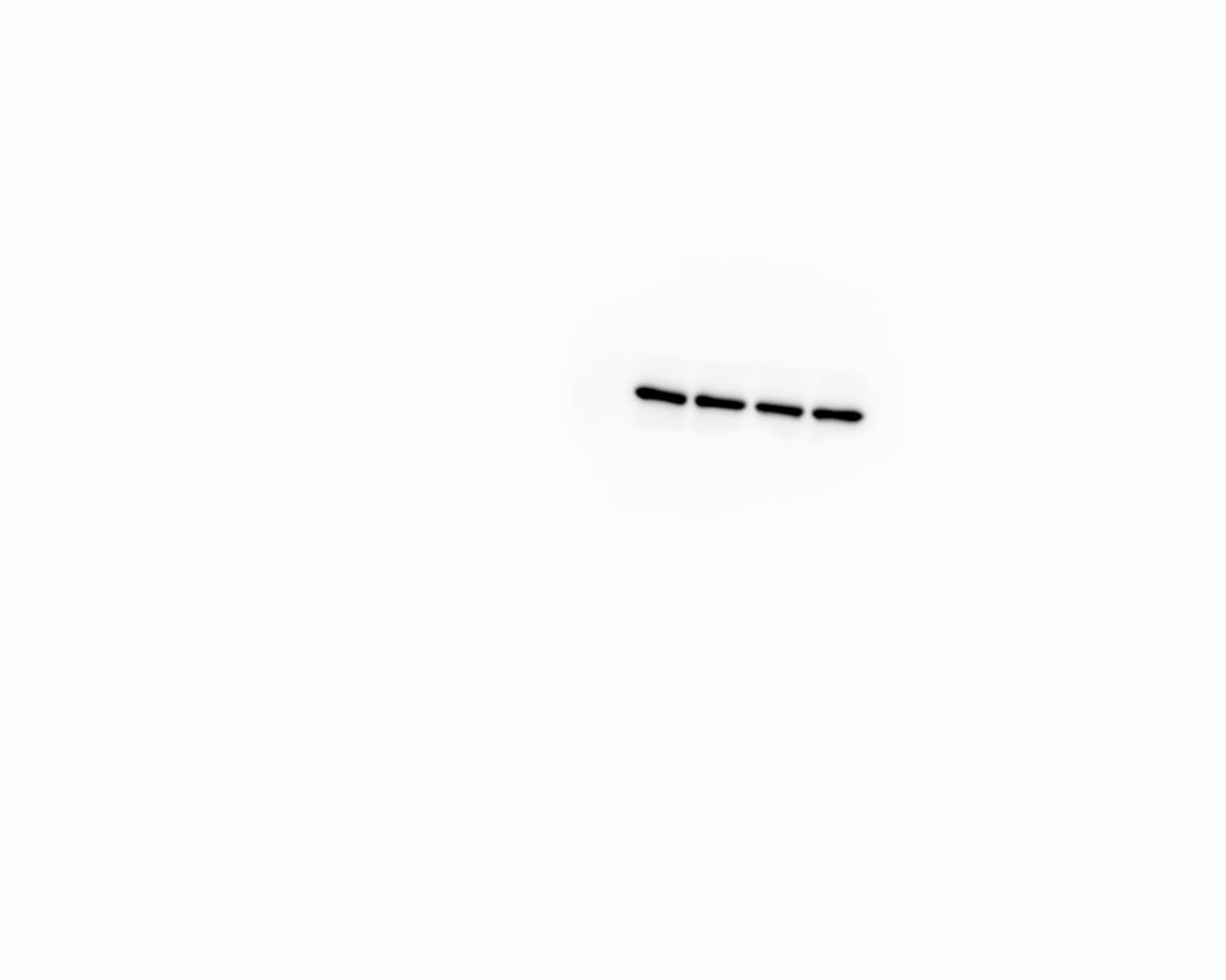

Ub-Input

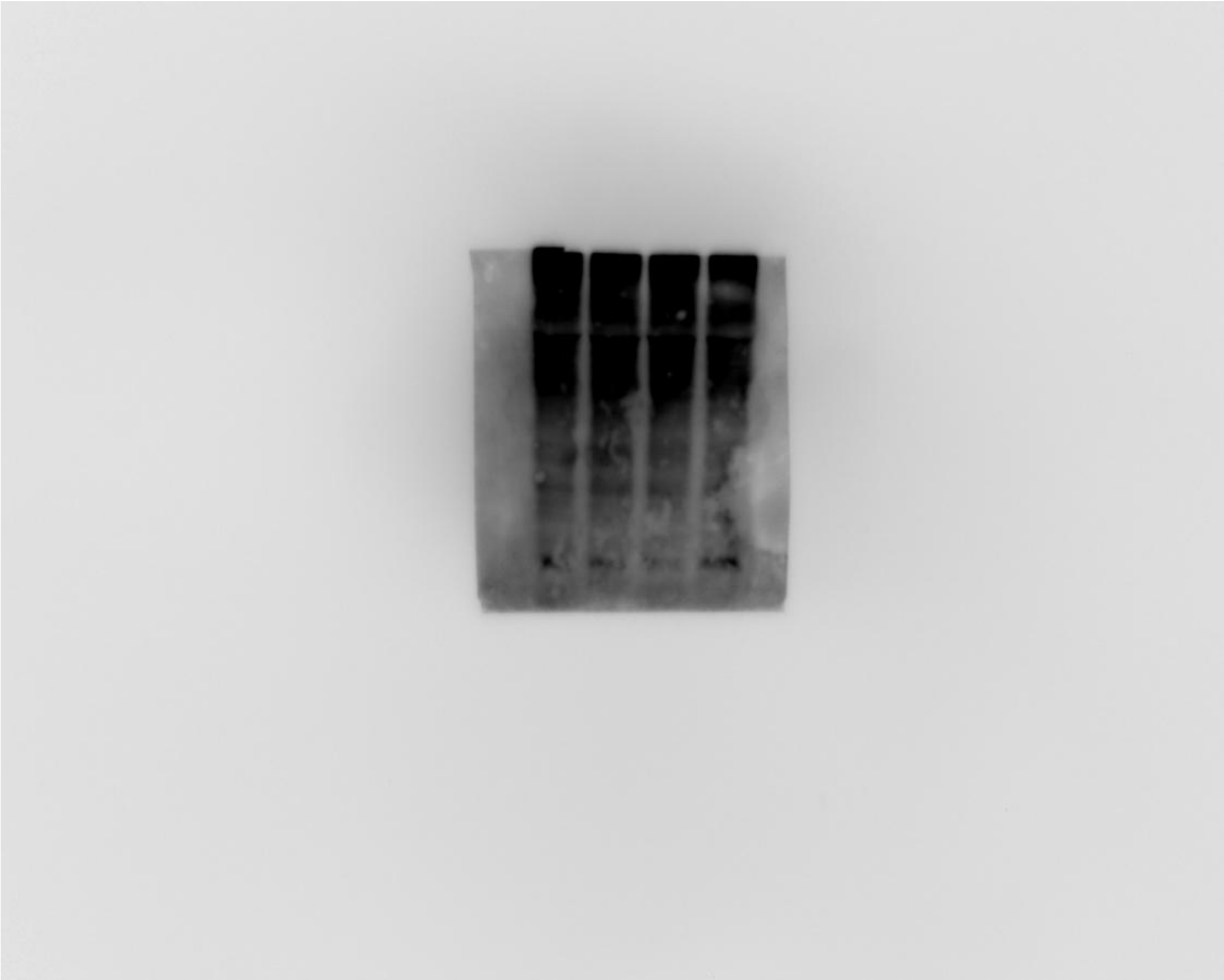

MED12-Input

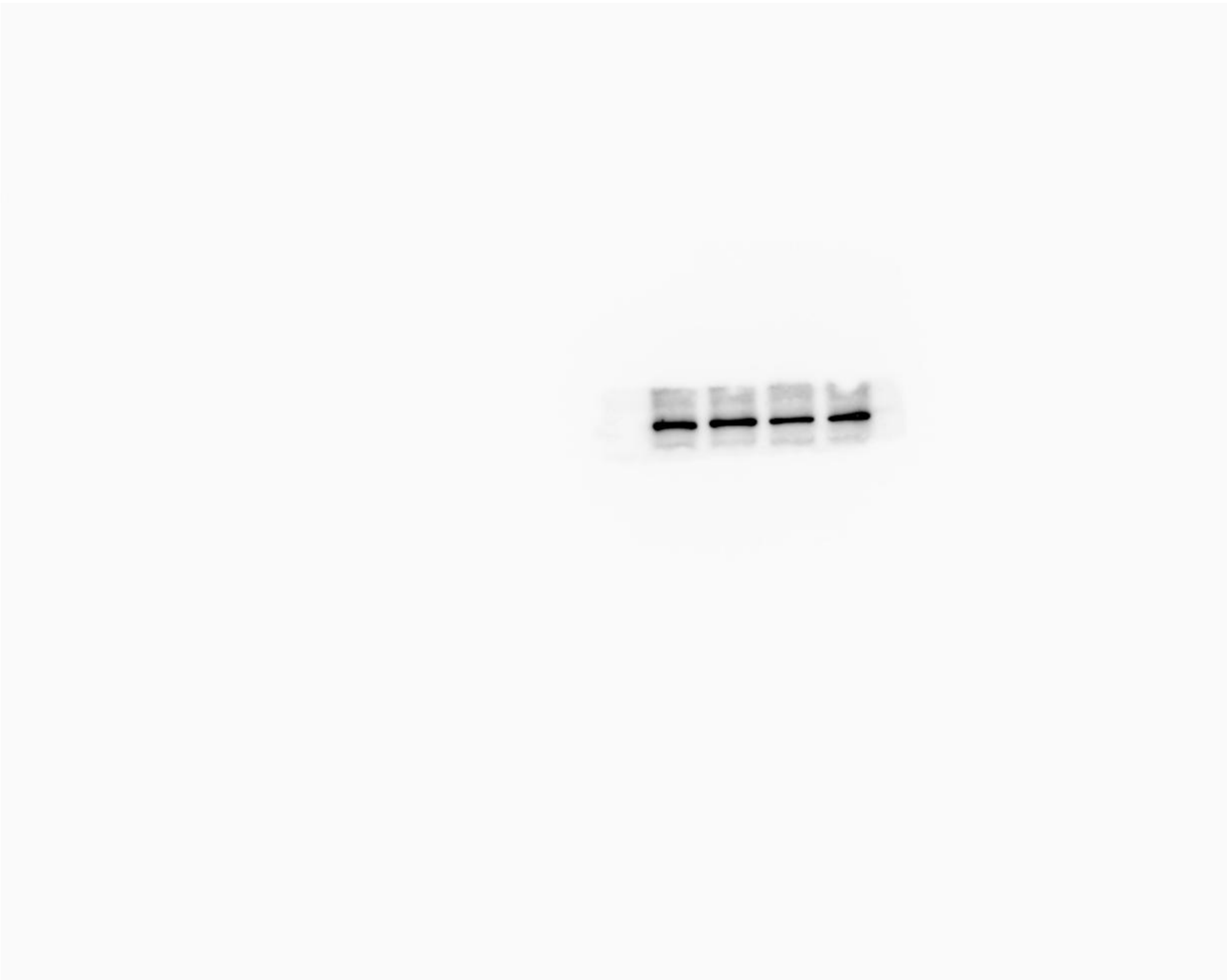

GFP-Input

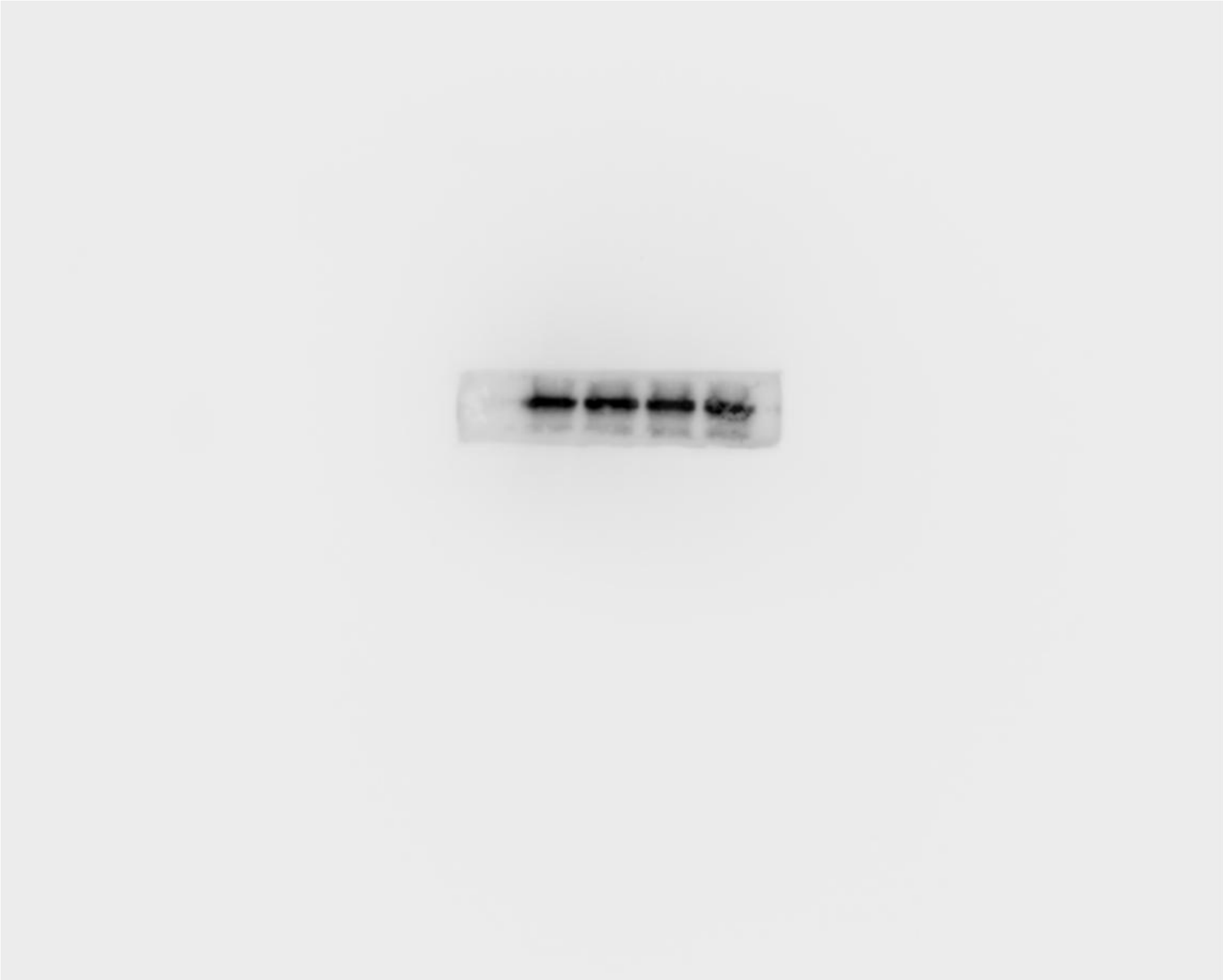

GAPDH

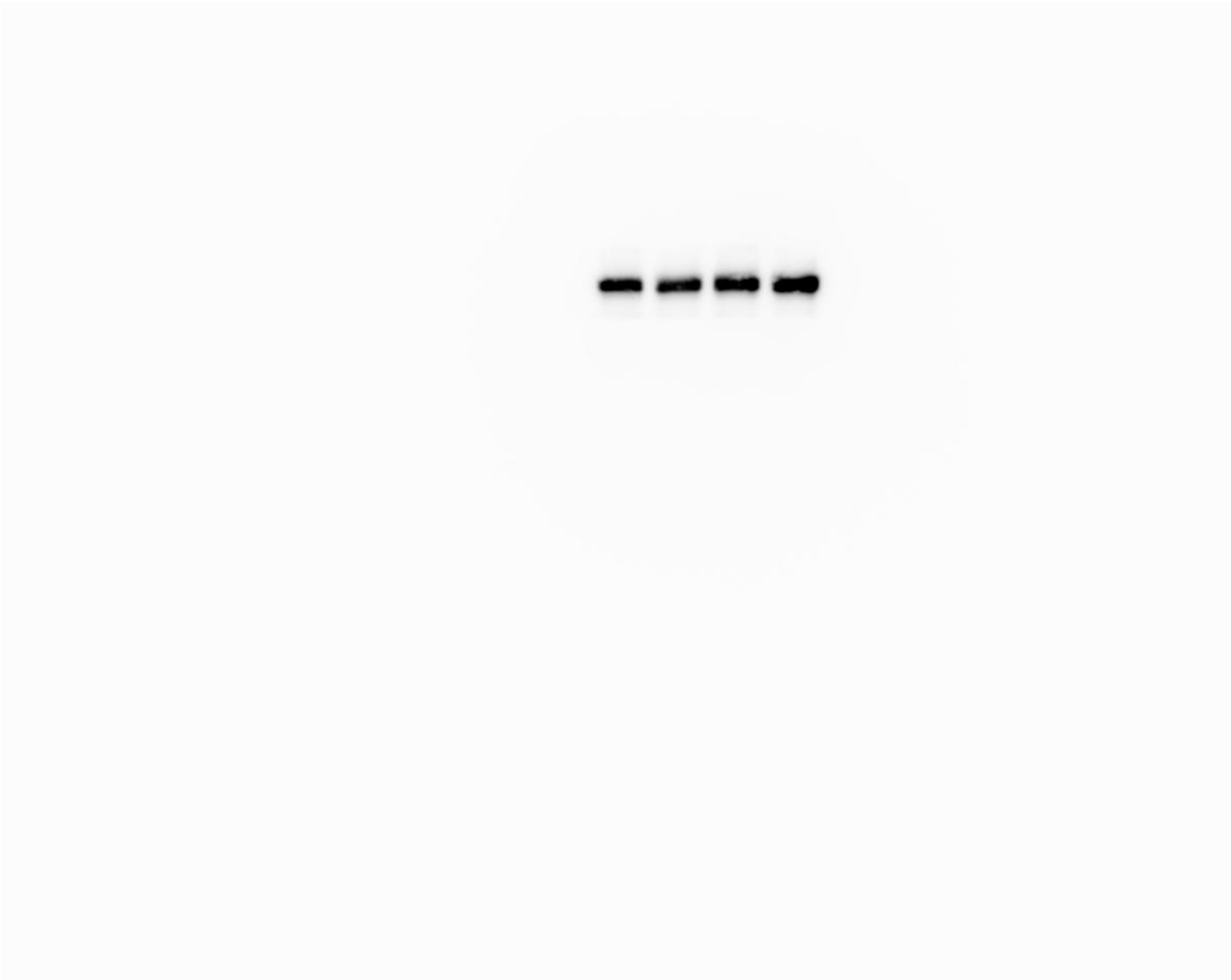

Fig5K

Ub-IP

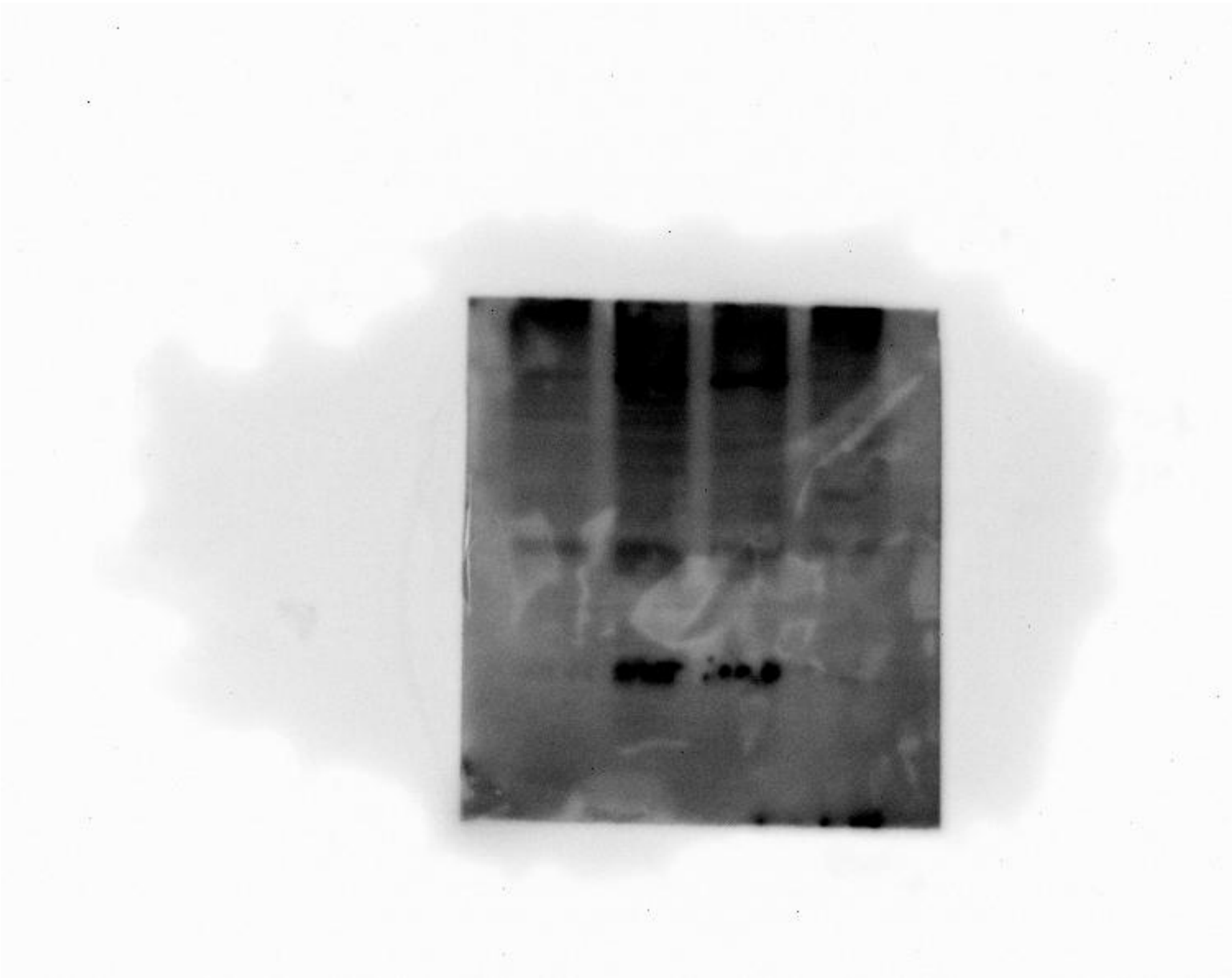

MED12-IP

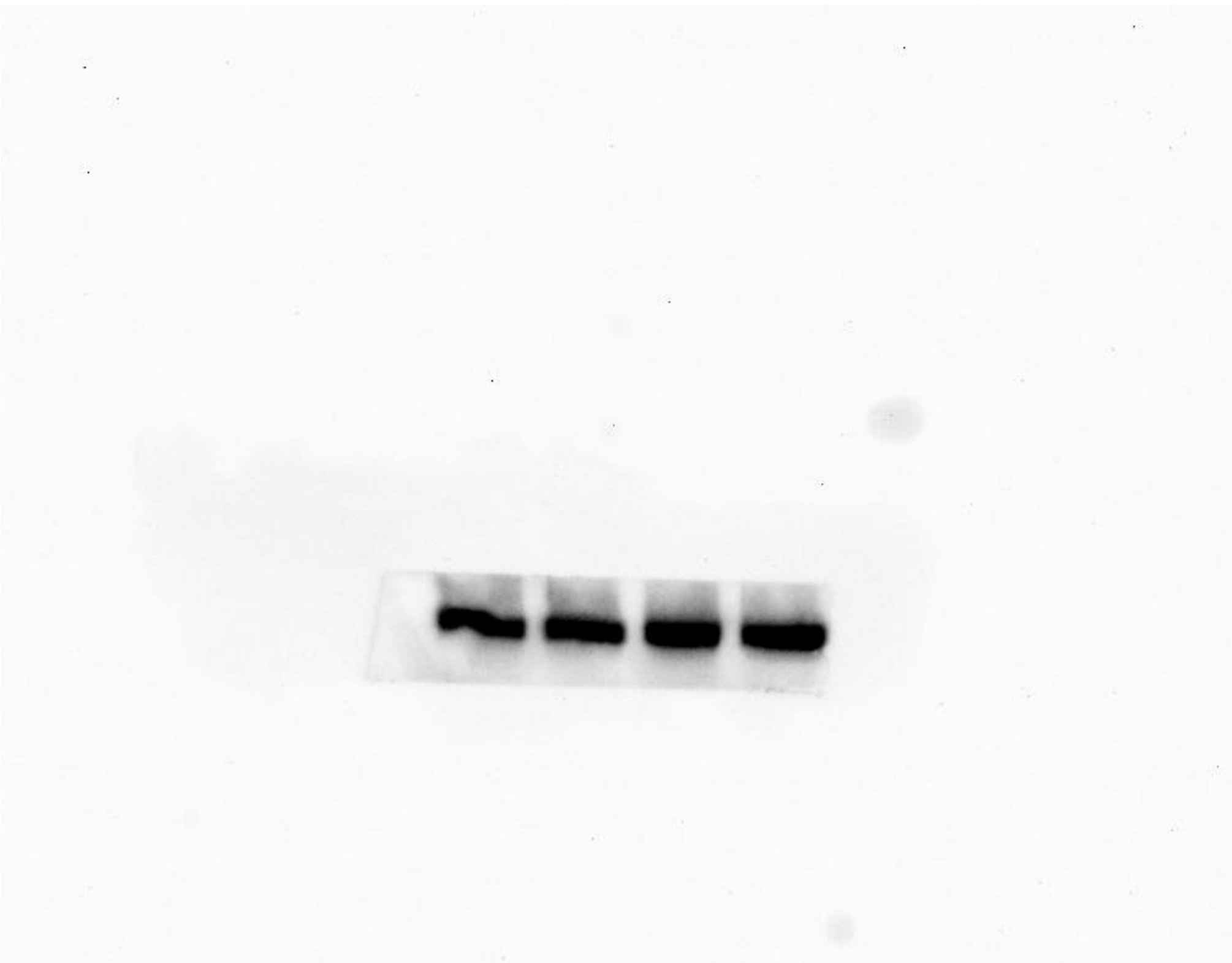

Ub-Input

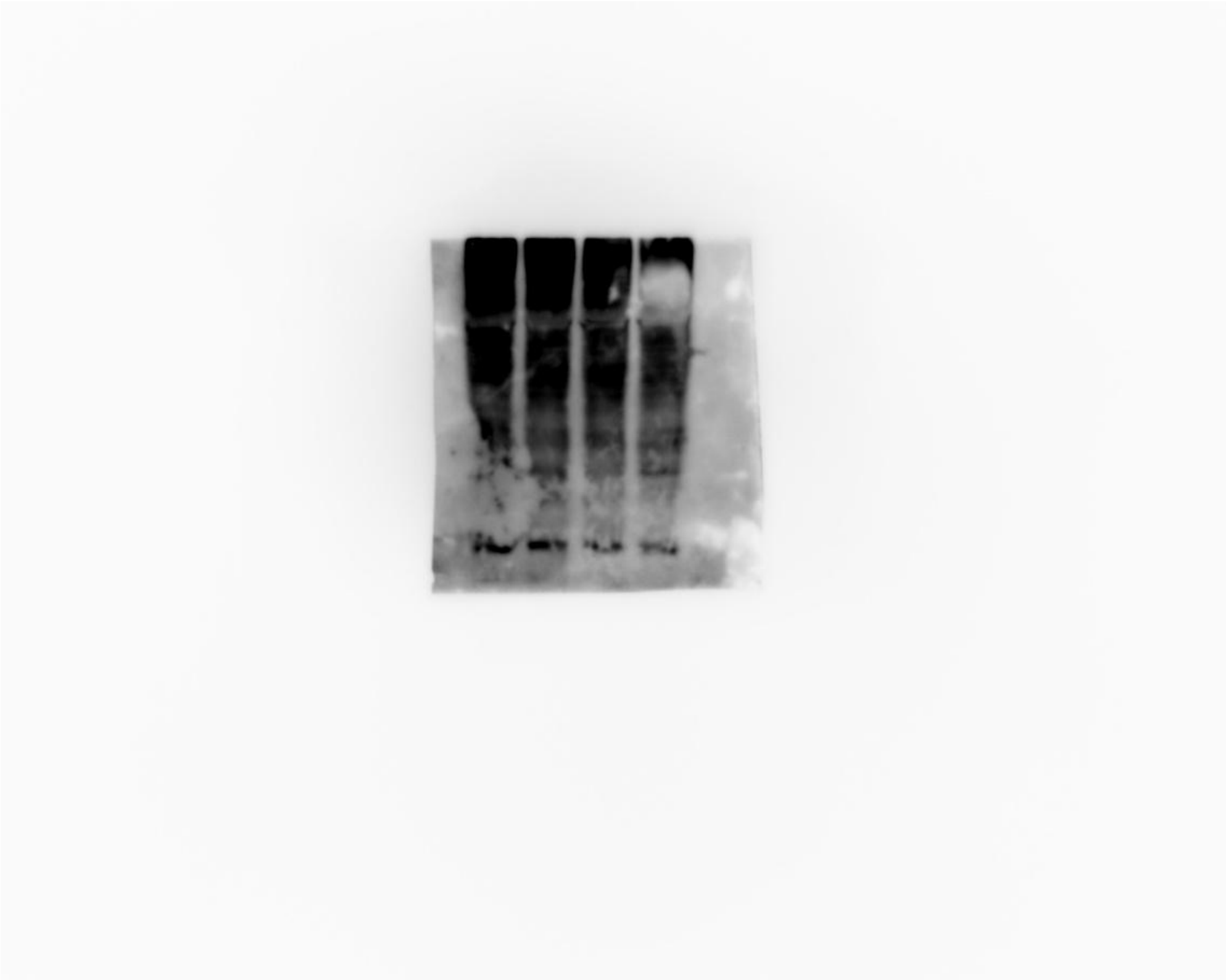

MED12-Input

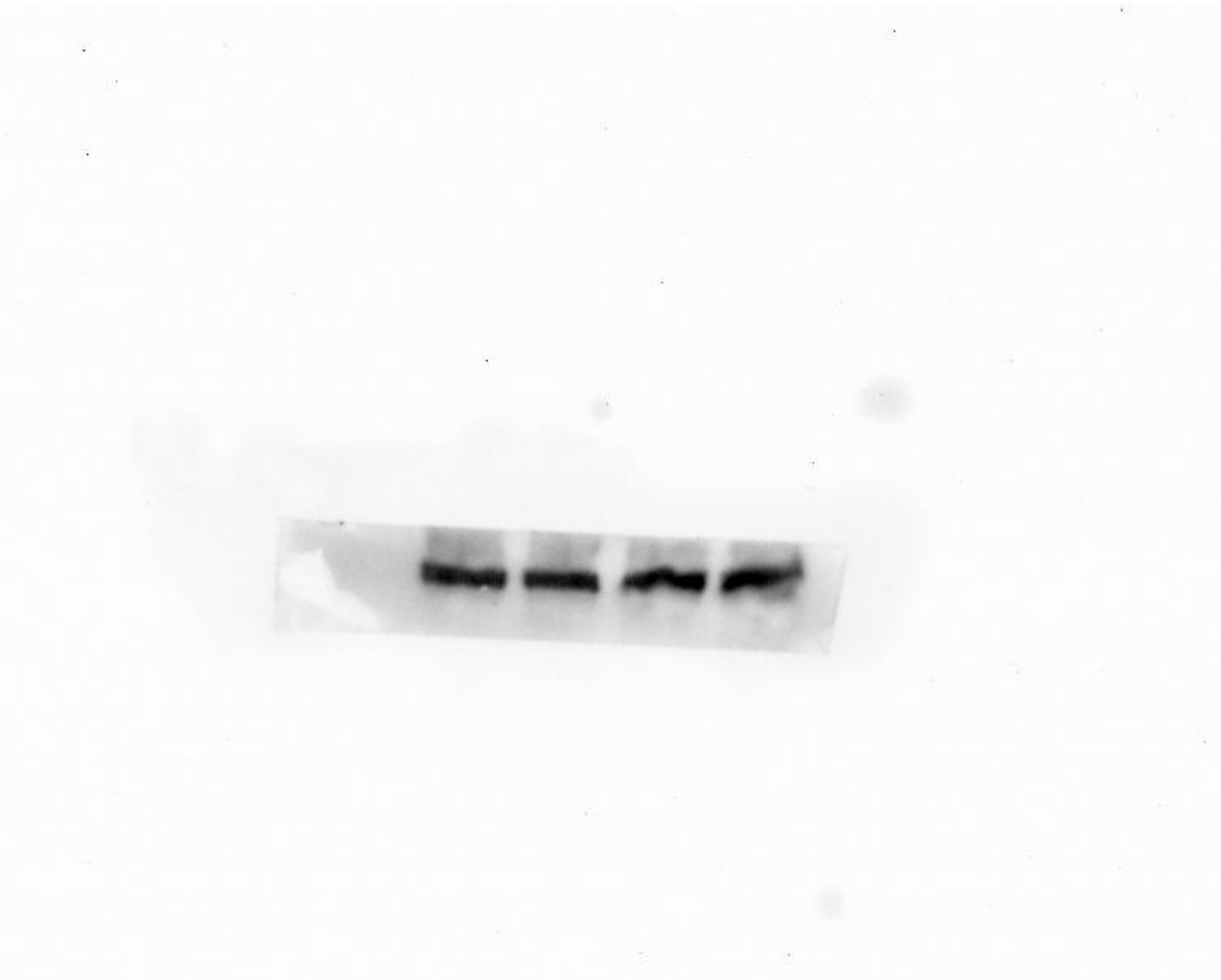

GFP-Input

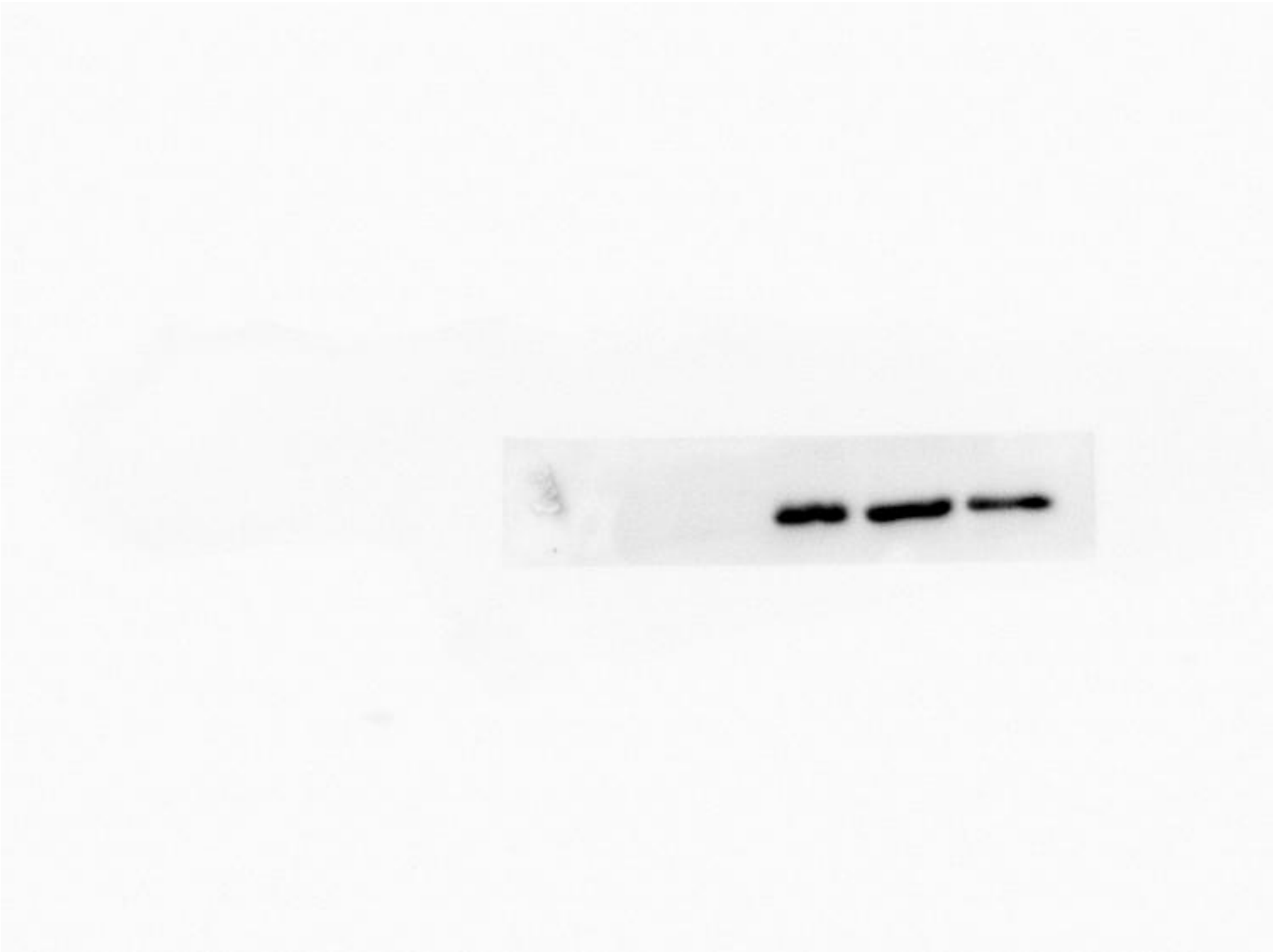

GAPDH

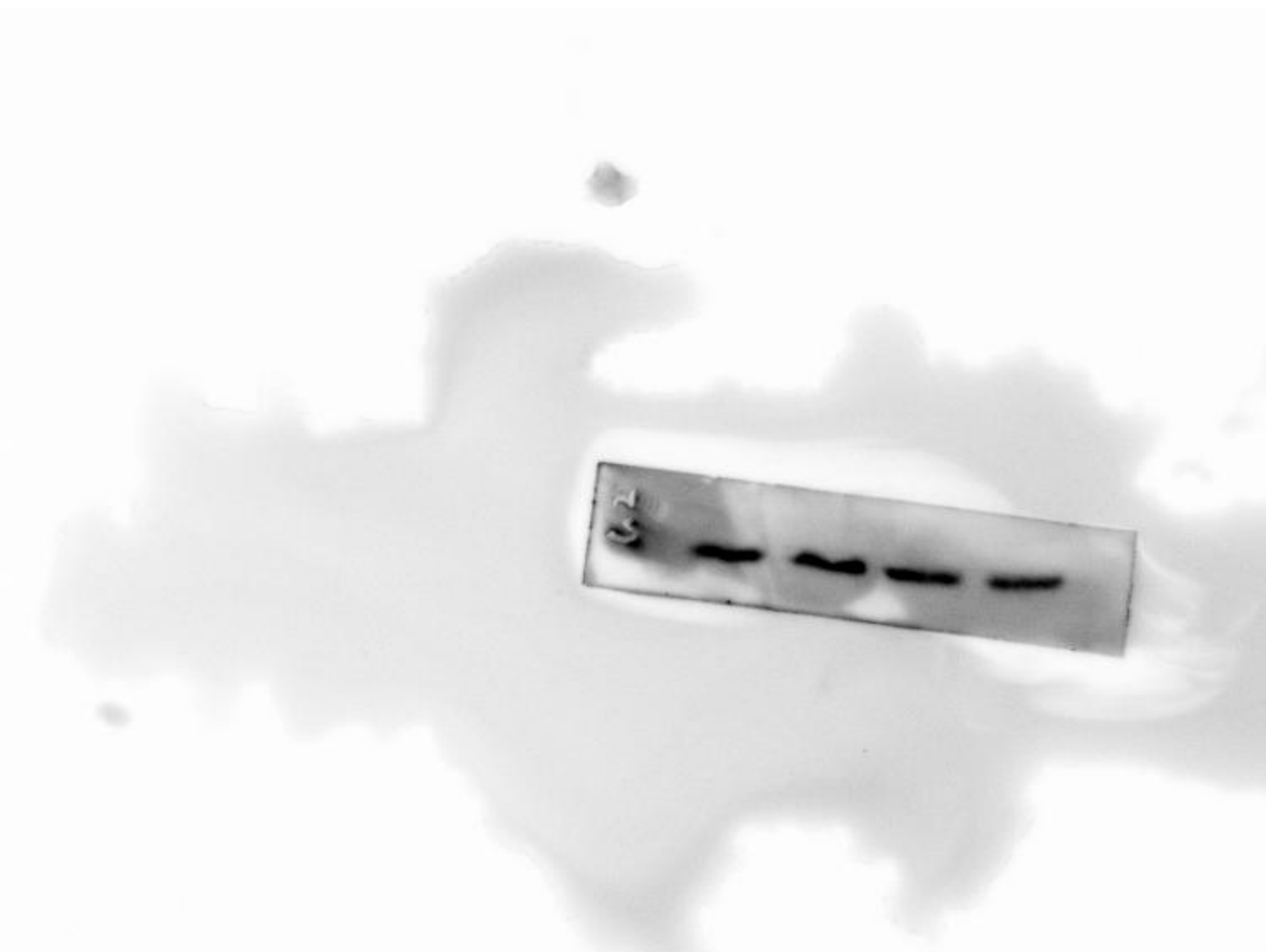

Fig5L

HA-Ub-IP

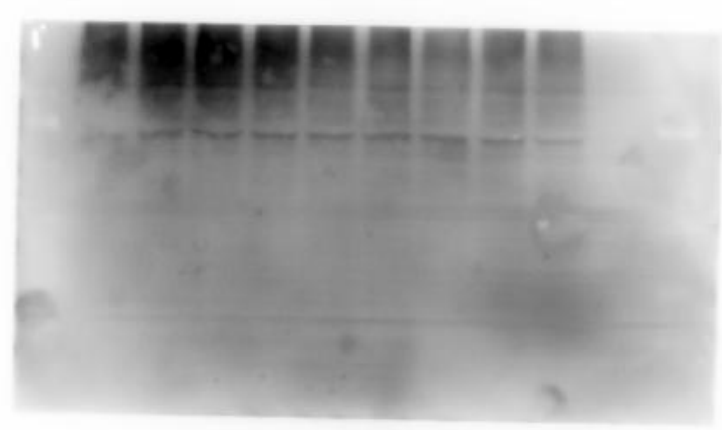

MED12-IP

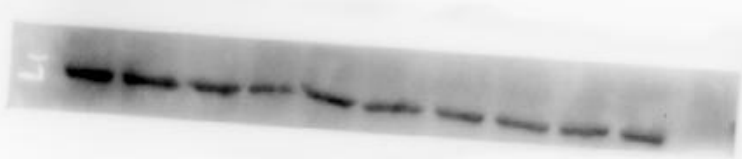

HA-Ub-IP

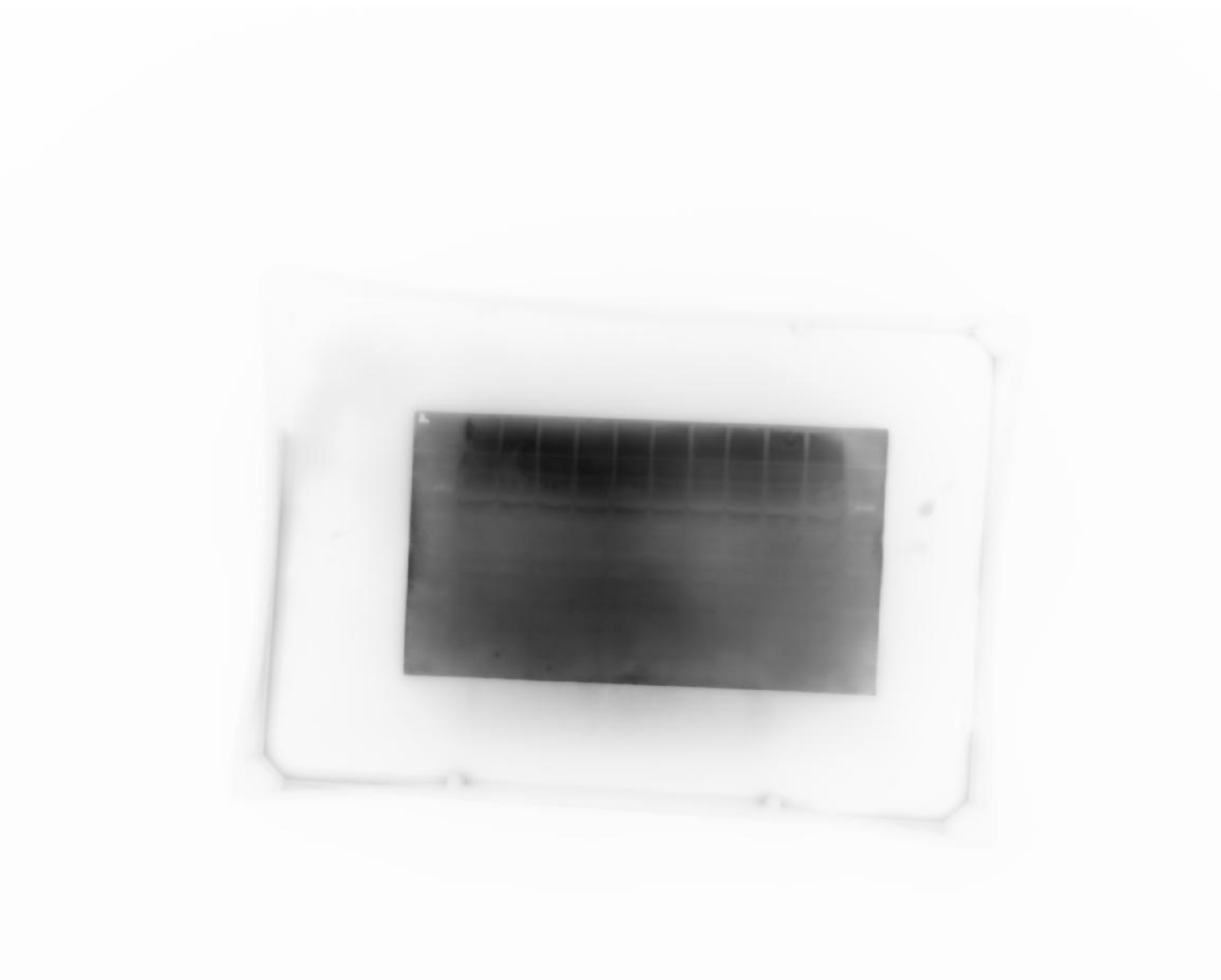

MED12-Input

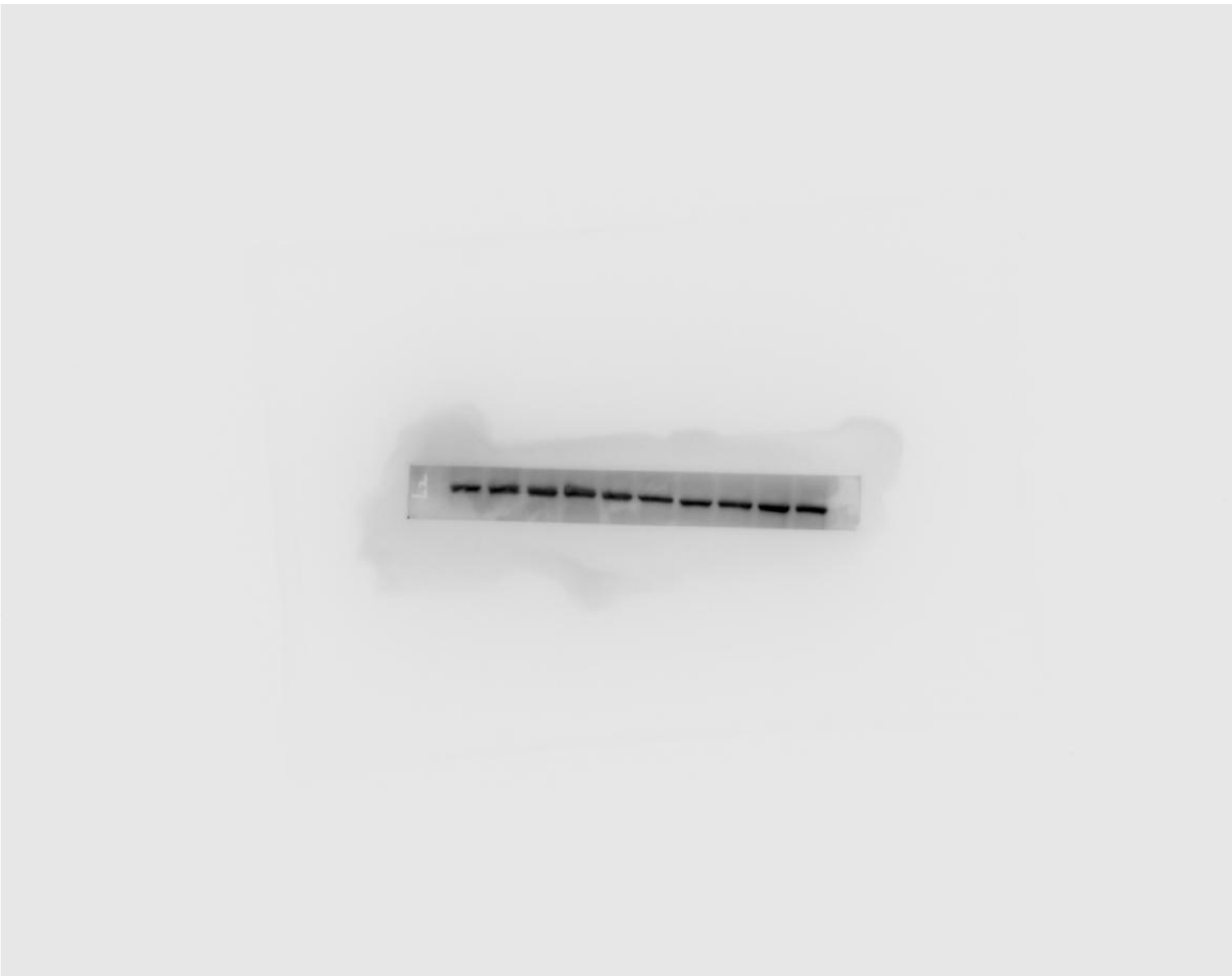

GAPDH

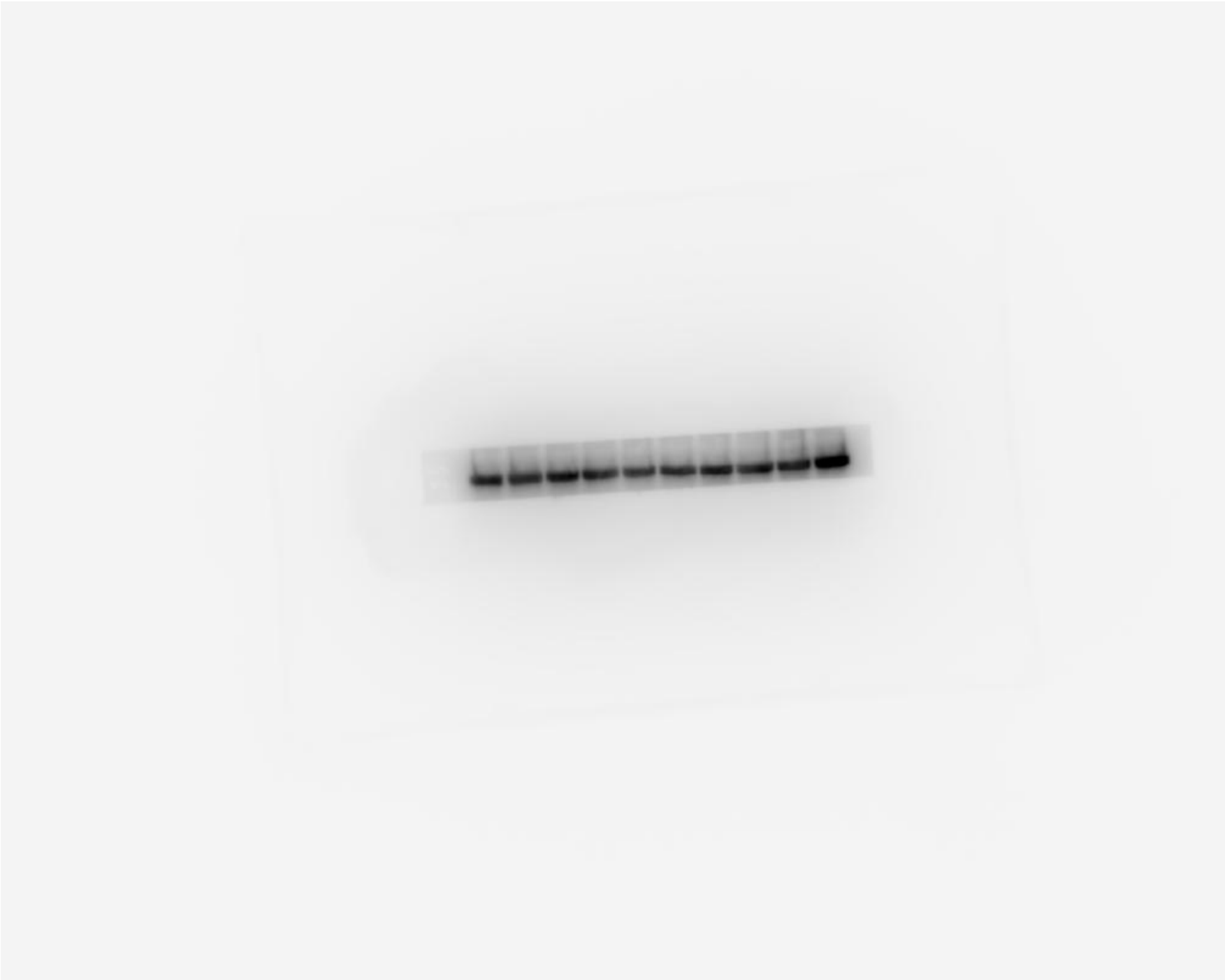

Fig5M

HA-Ub-IP

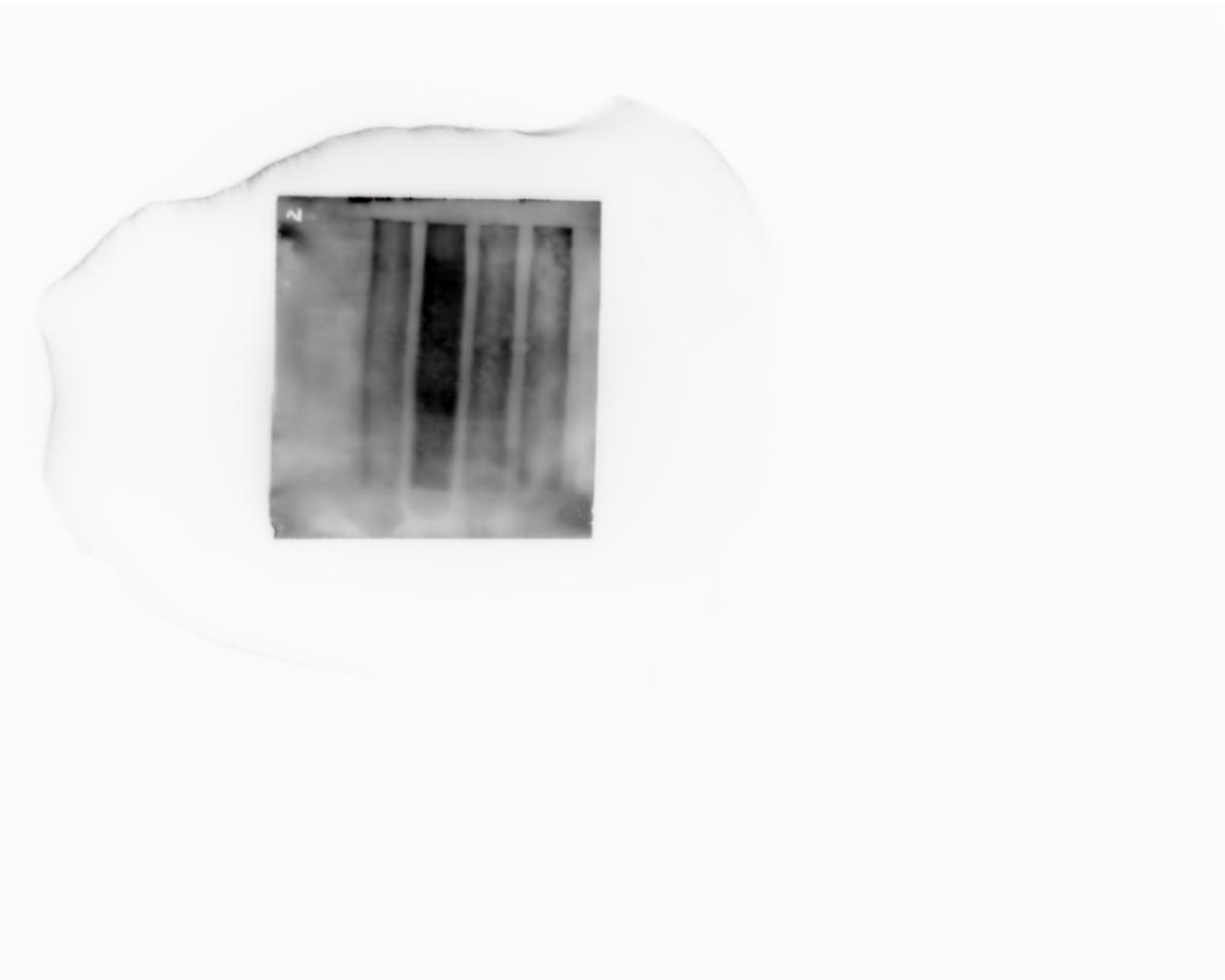

Flag-IP

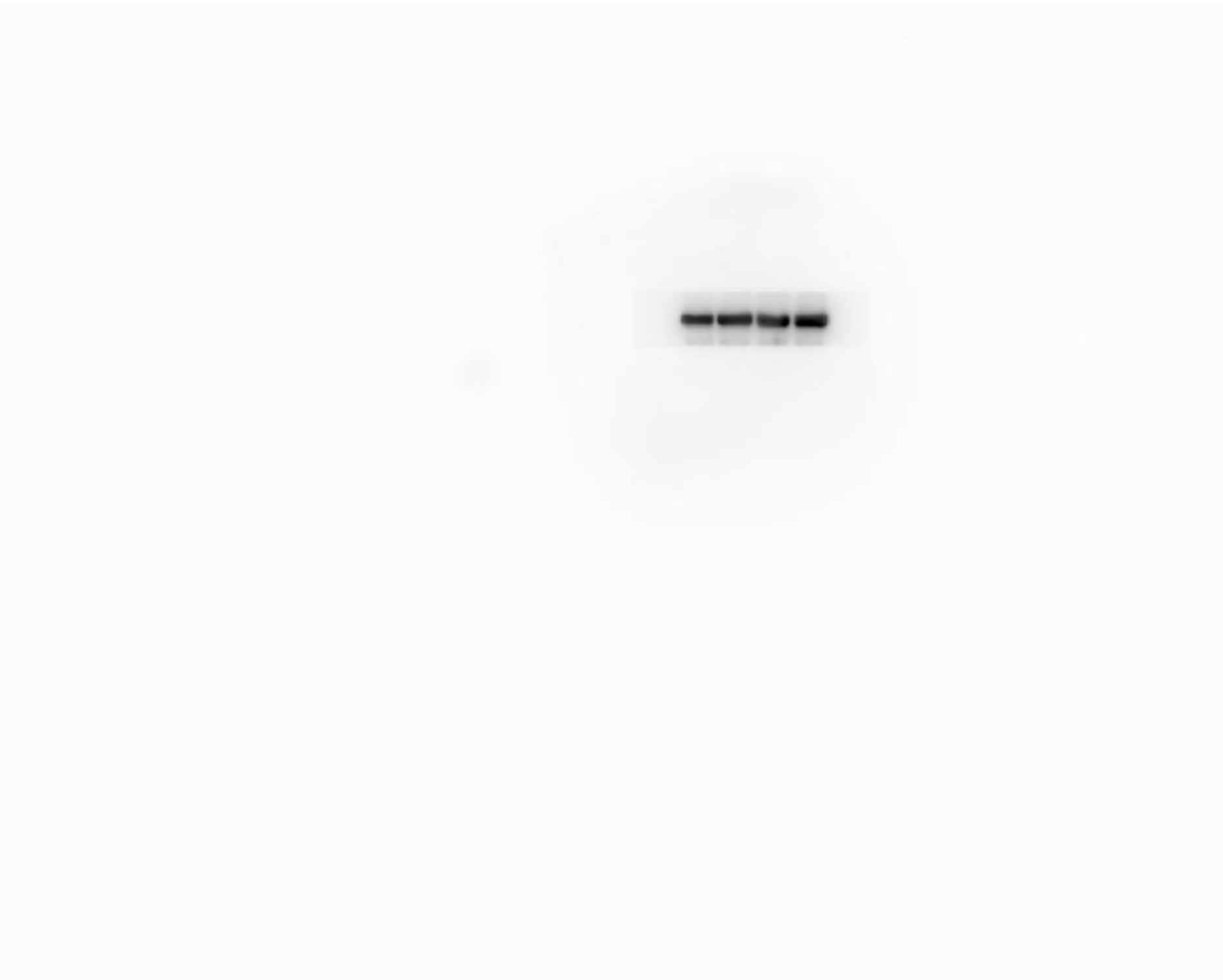

HA-Ub-Input

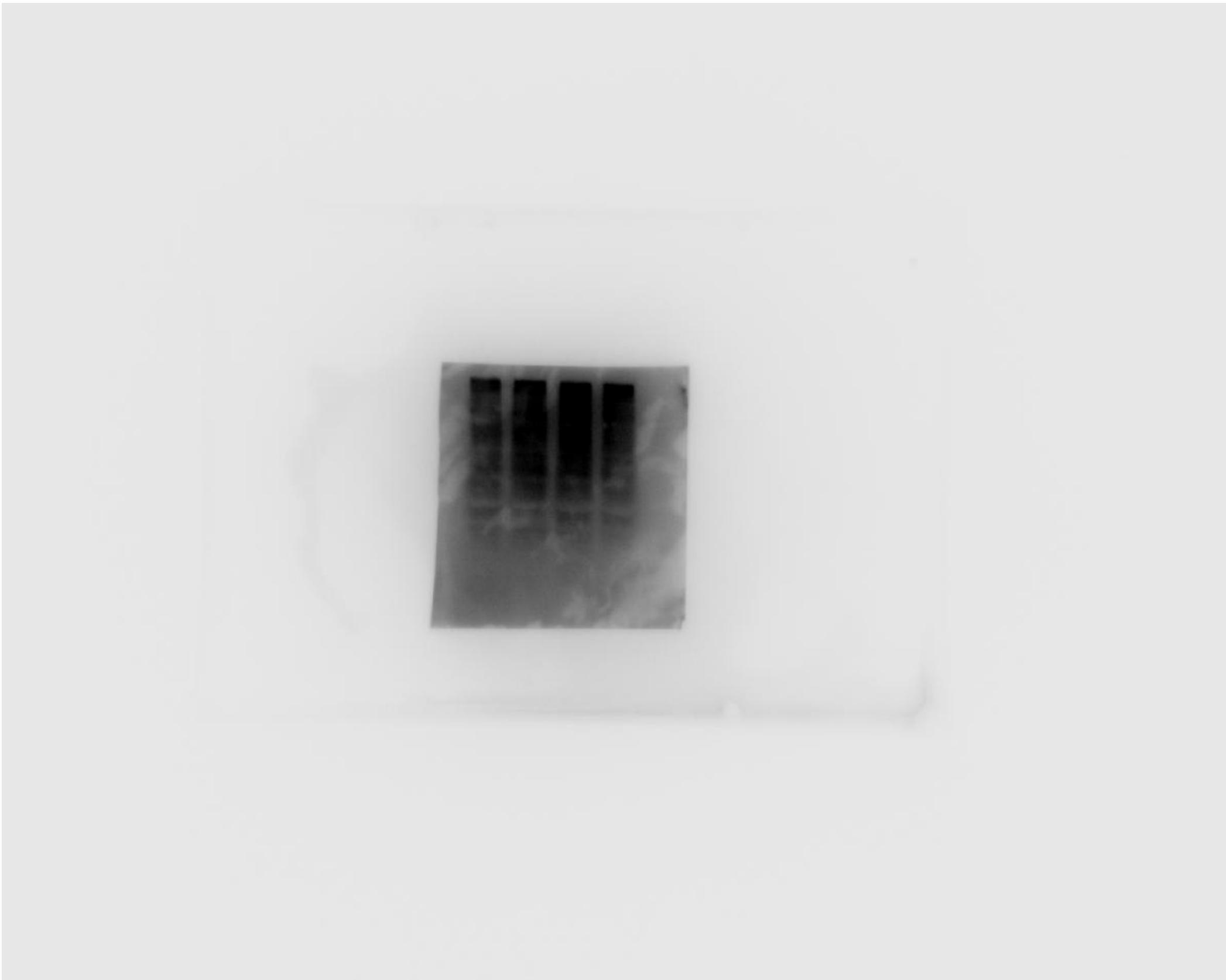

Flag-Input

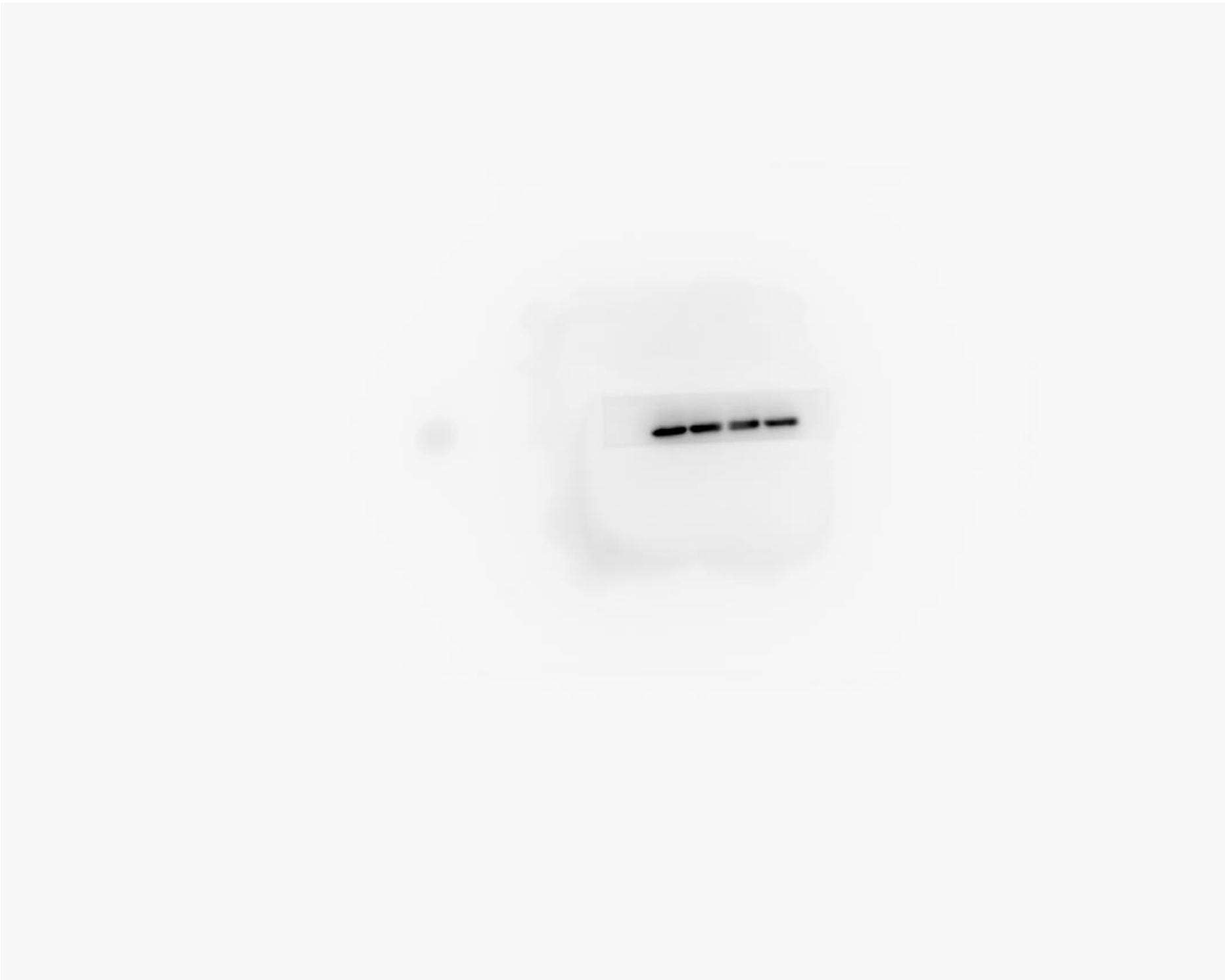

GFP-Input

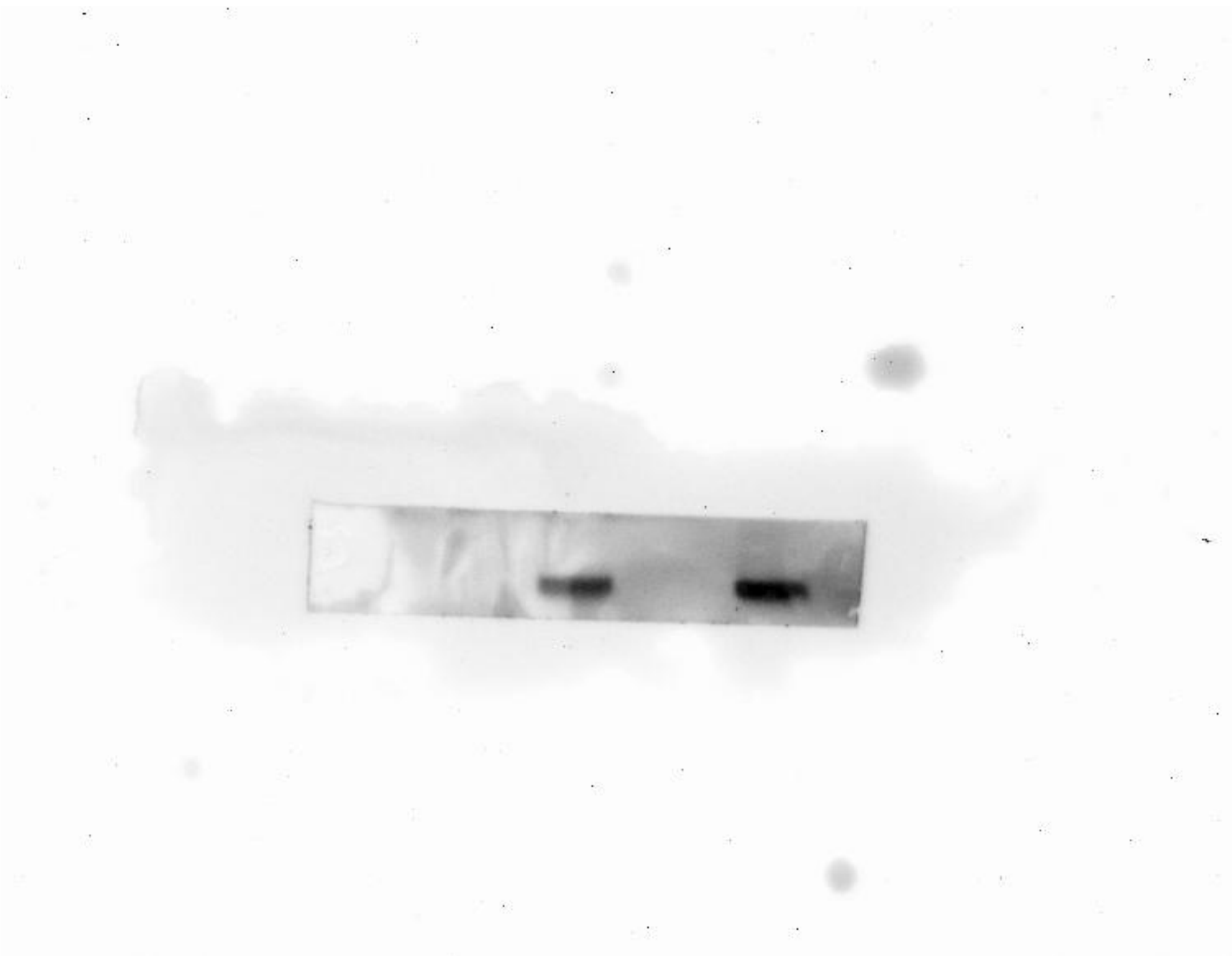

GAPDH

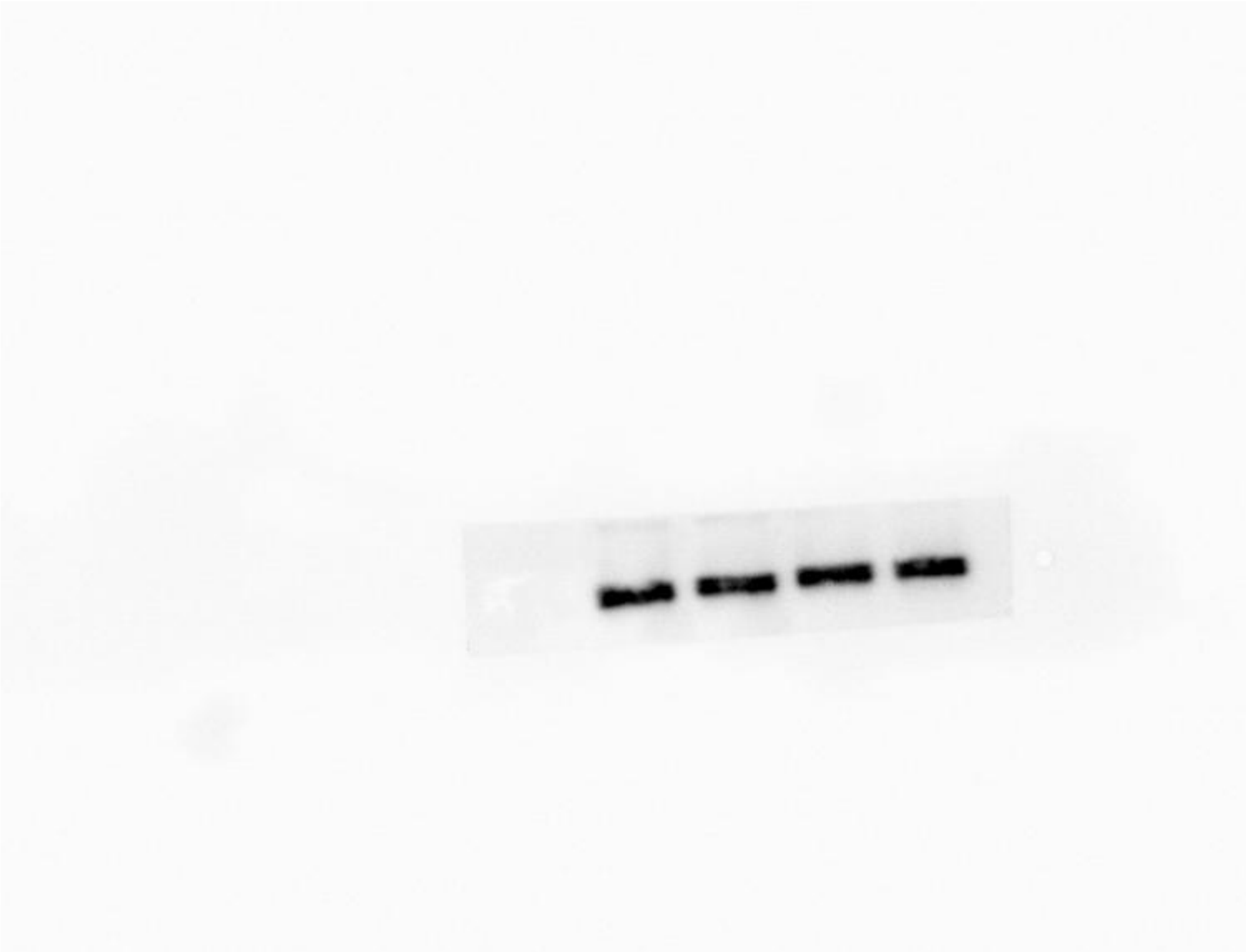

Fig6F

pp53

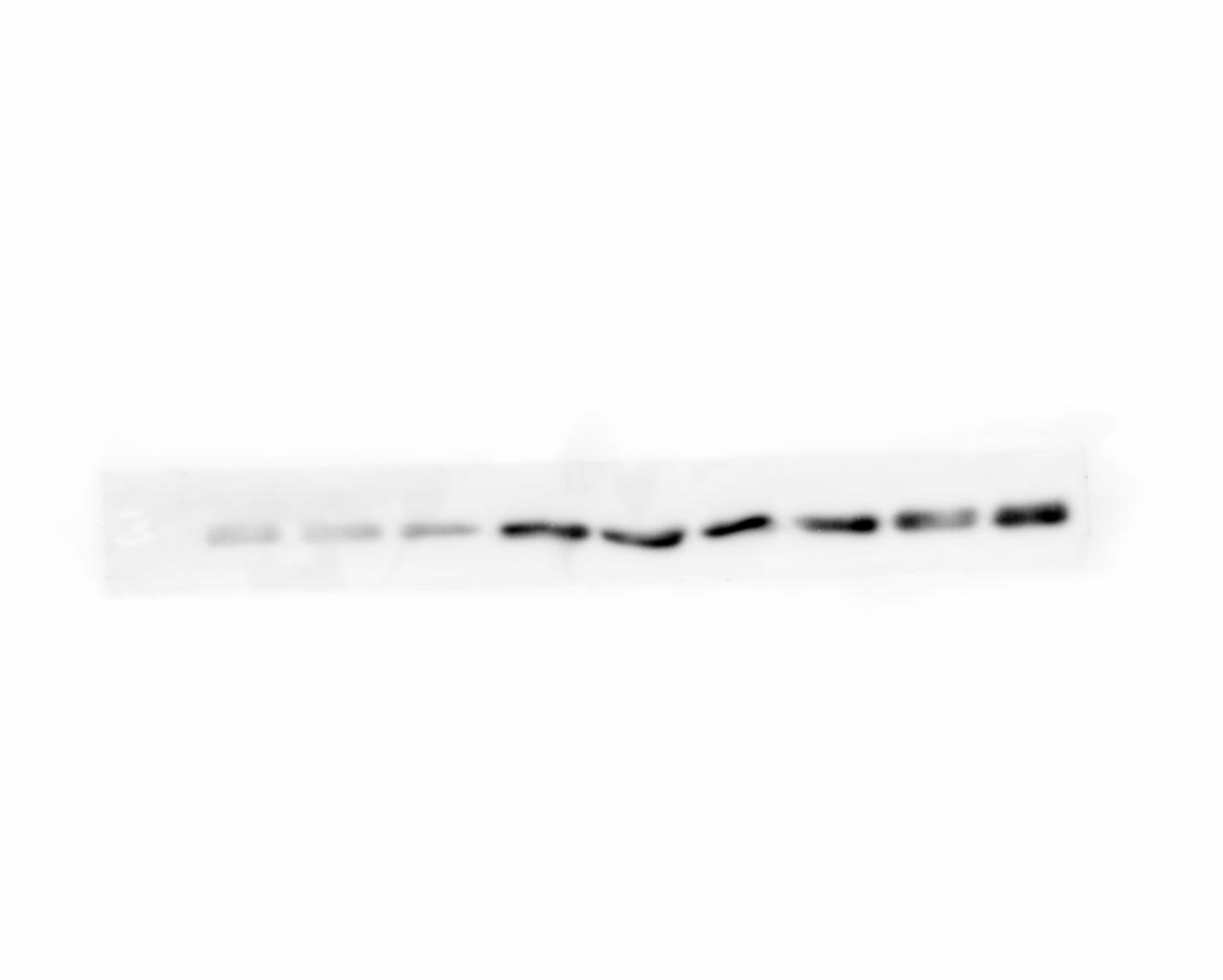

p53

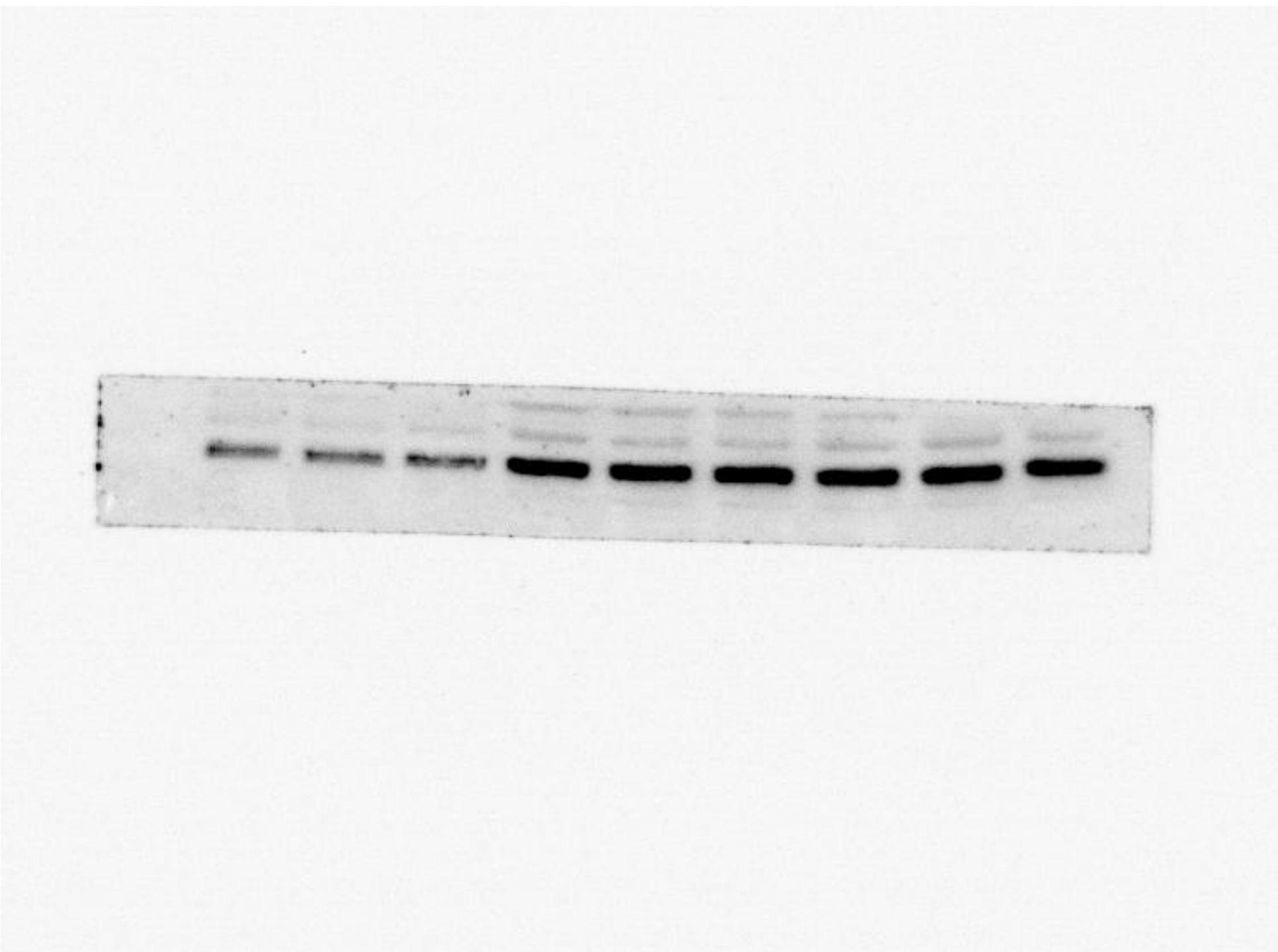

p21

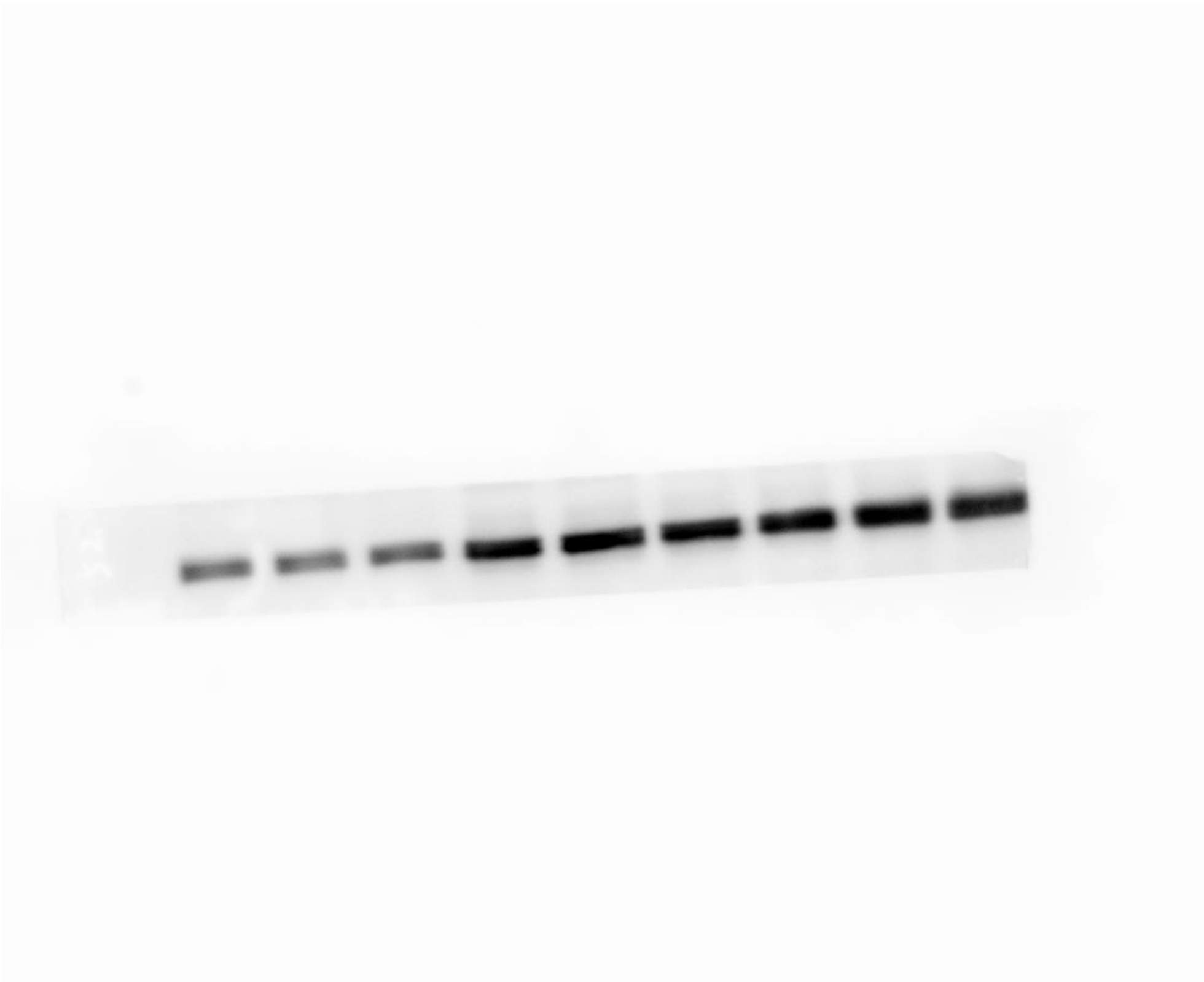

p16

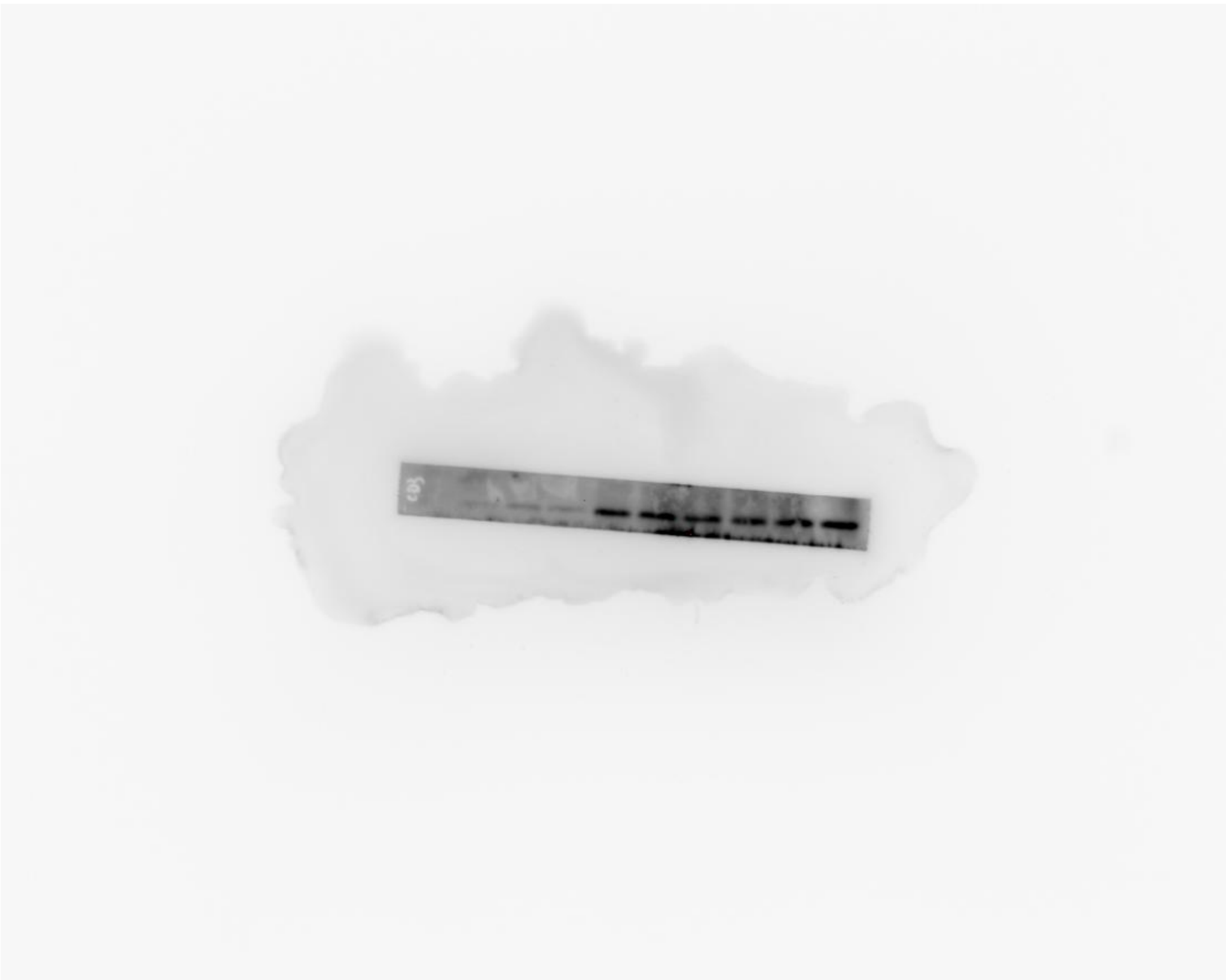

GAPDH

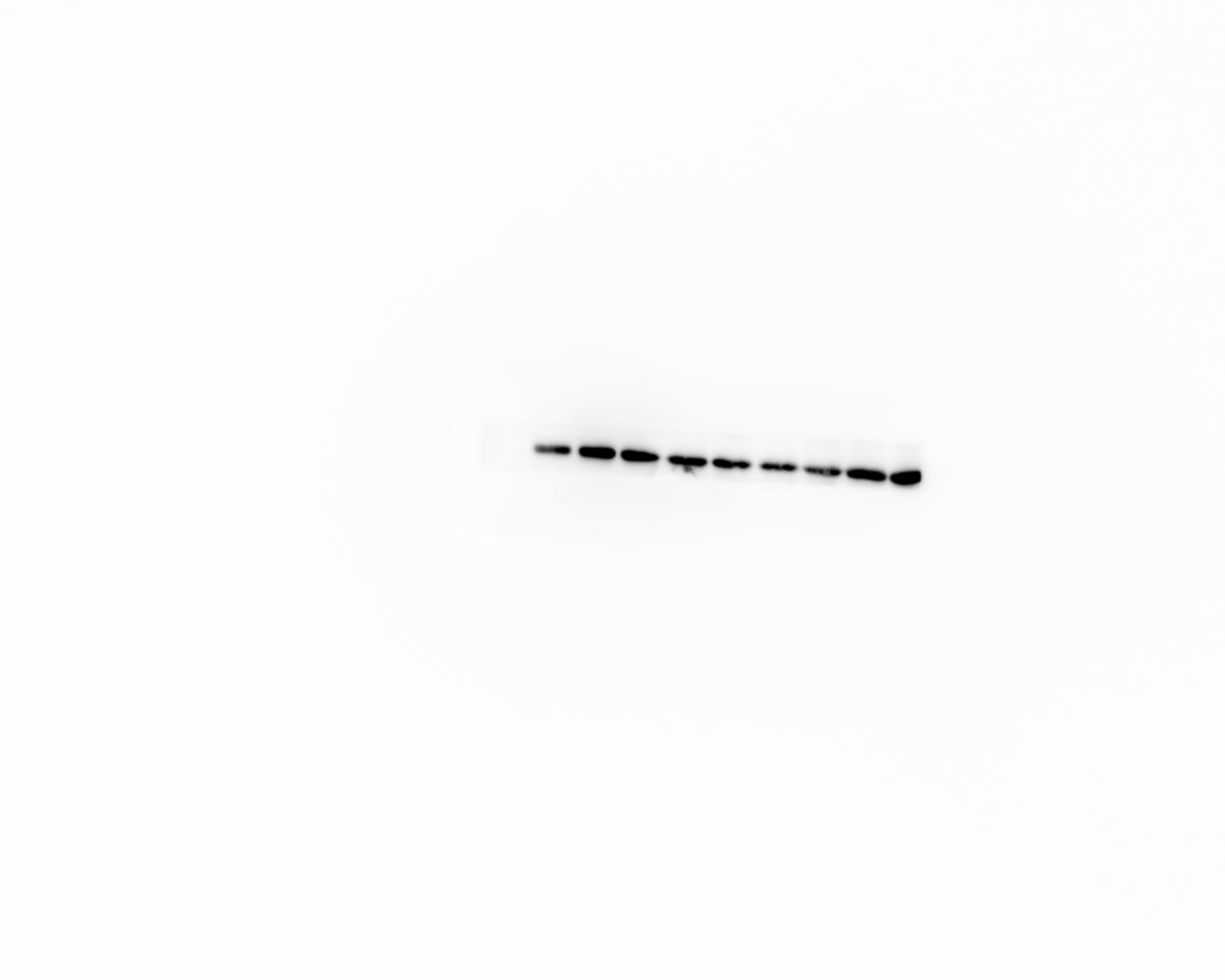

Fig6H

p-TBK1

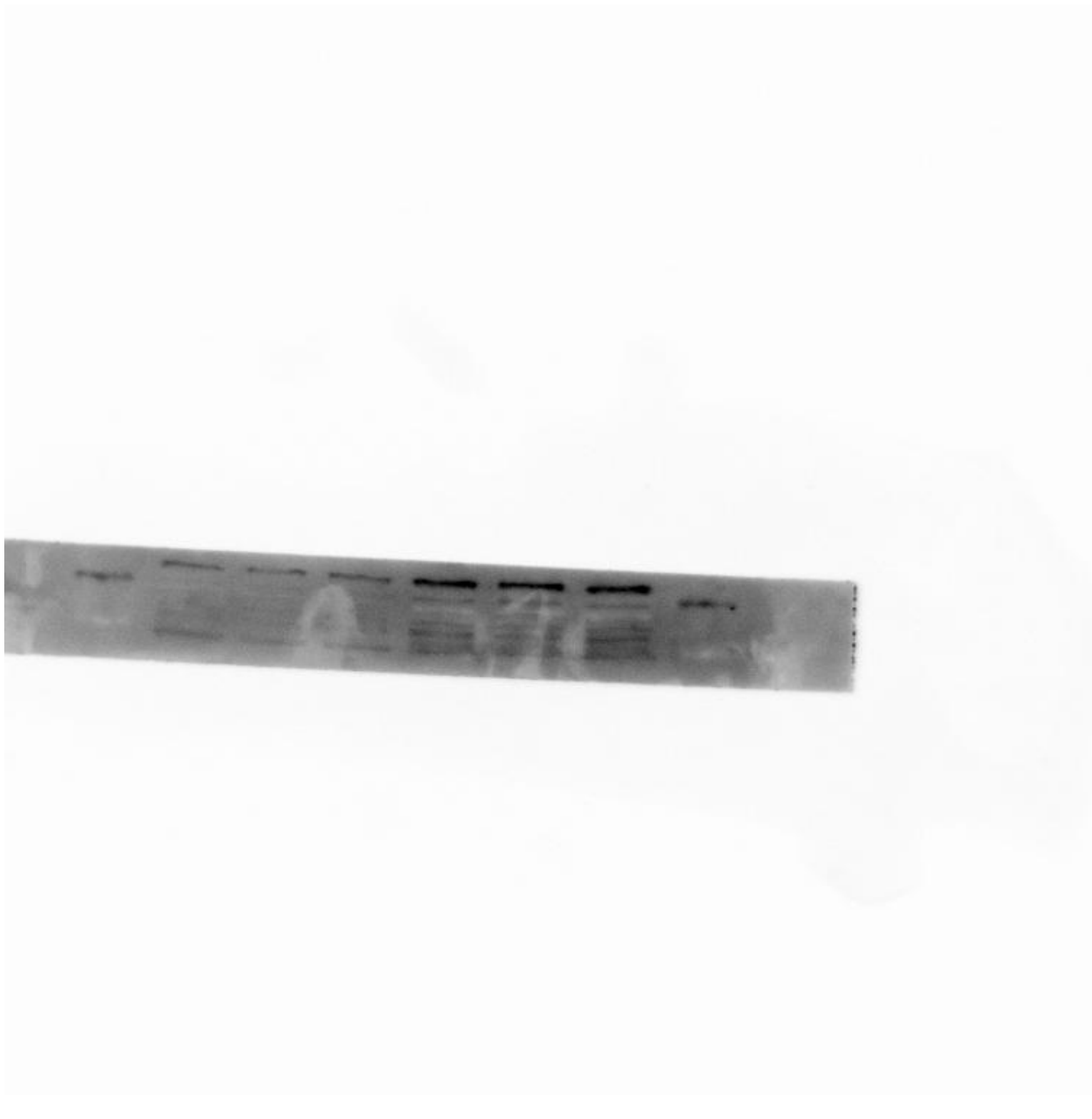

TBK1

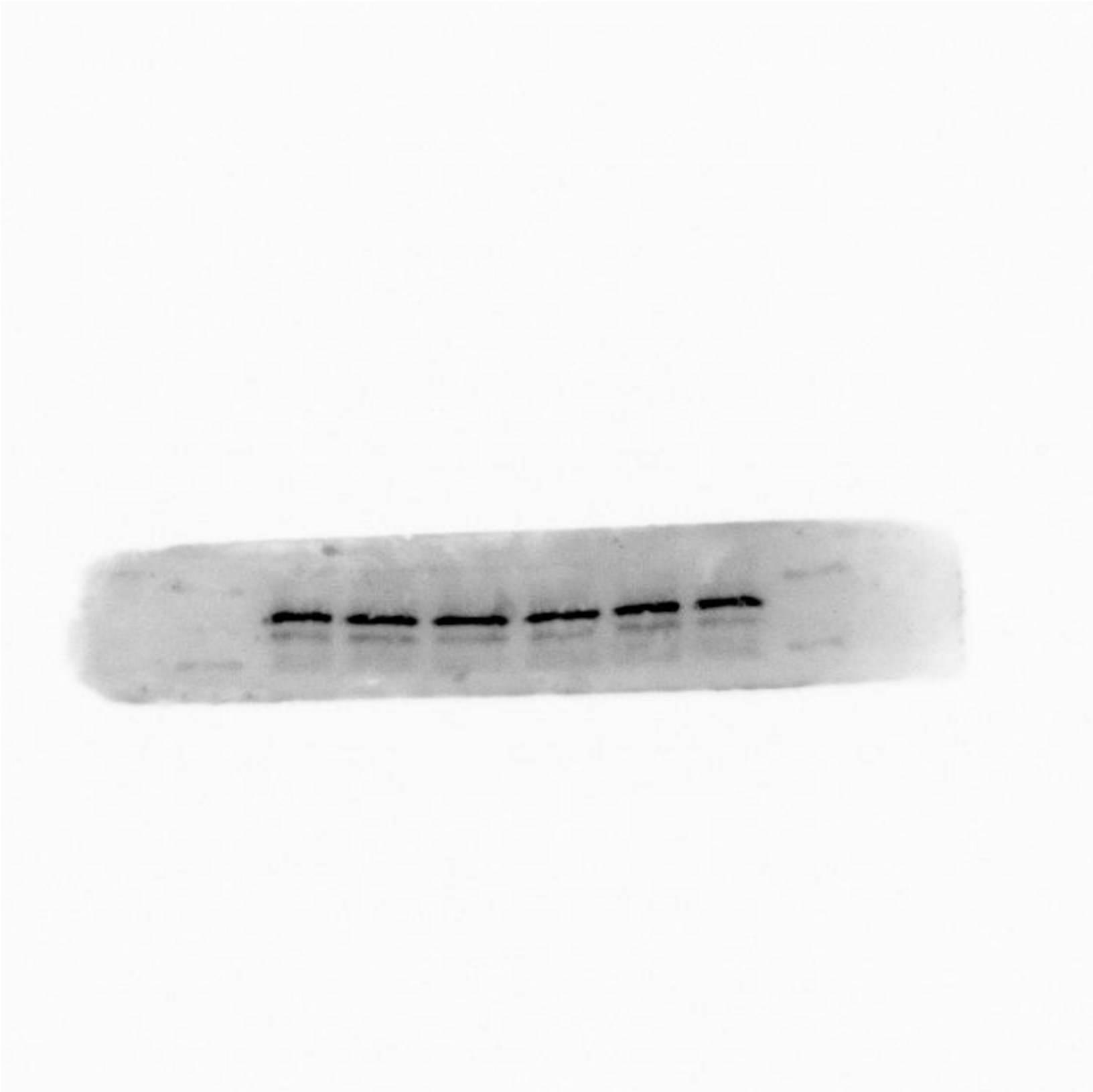

p-IRF3

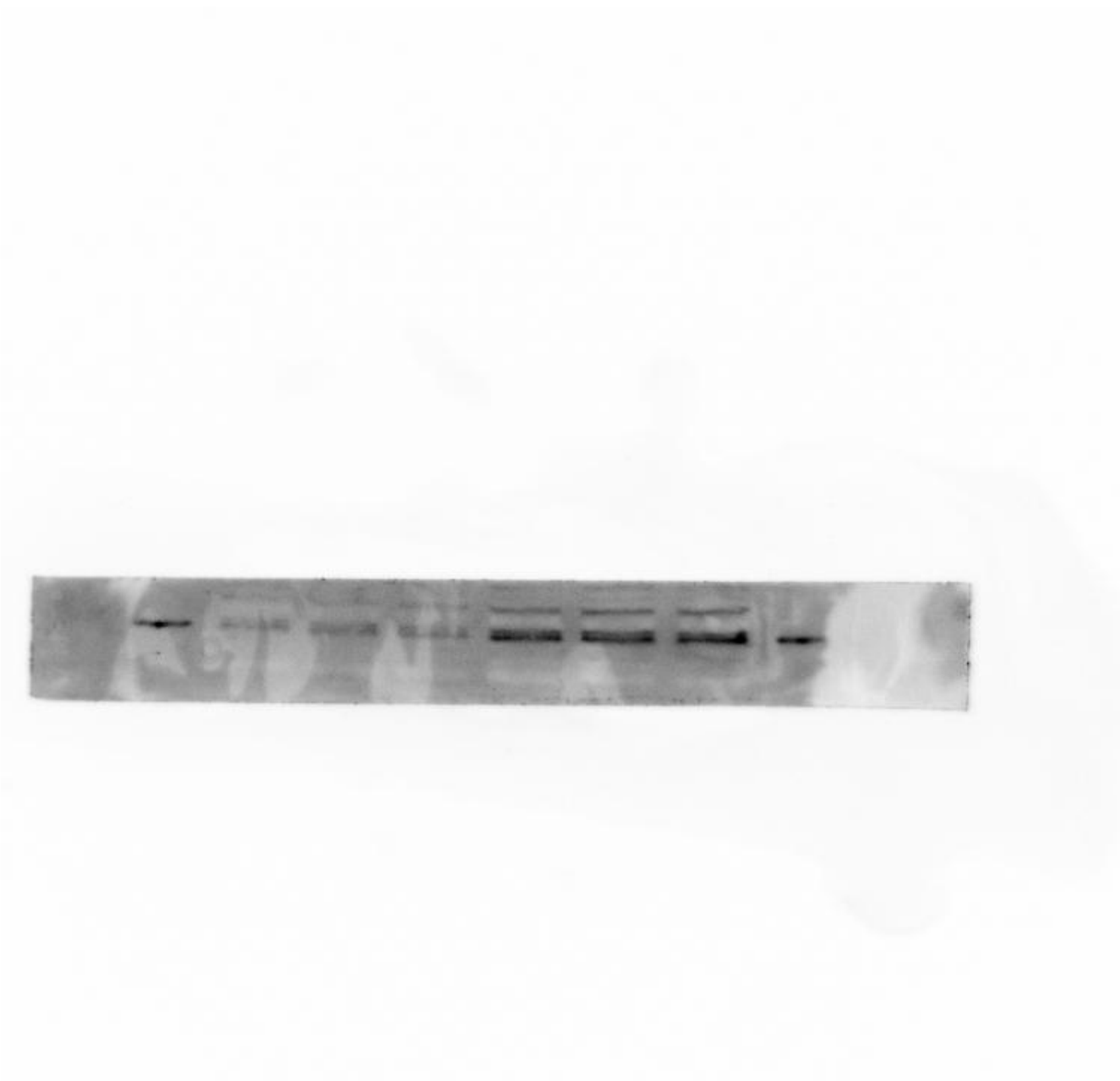

IRF3

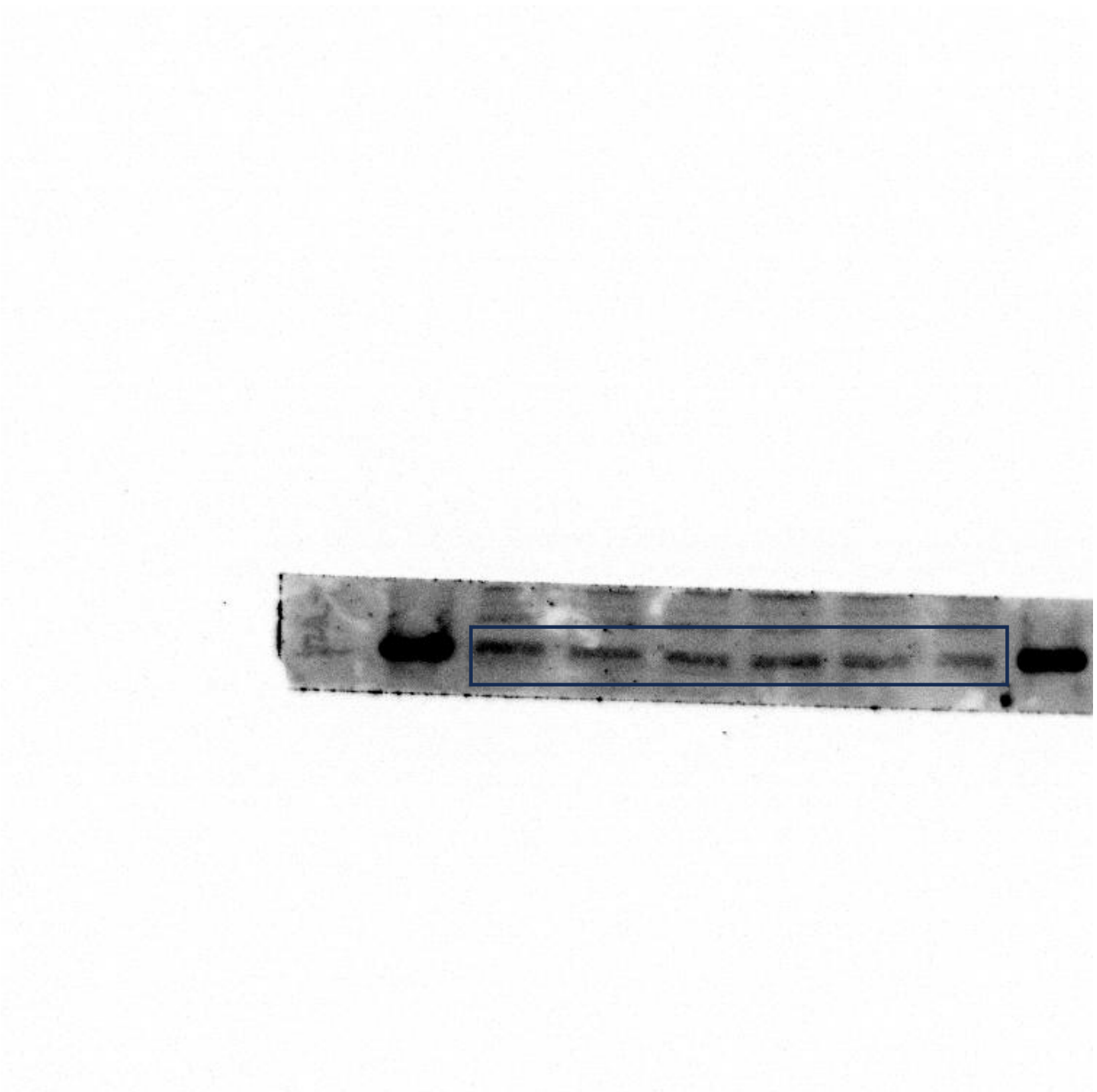

STING

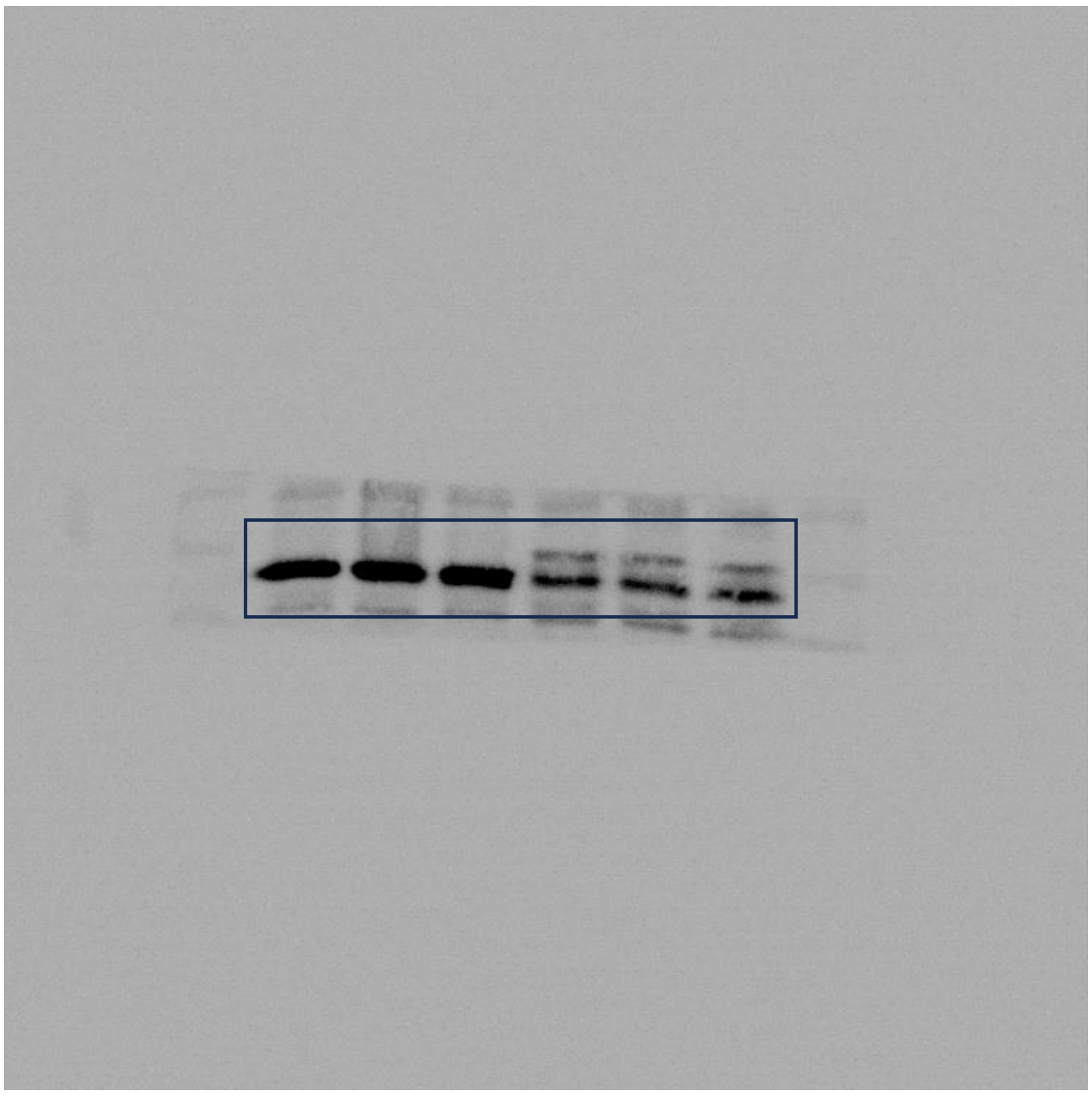

GAPDH

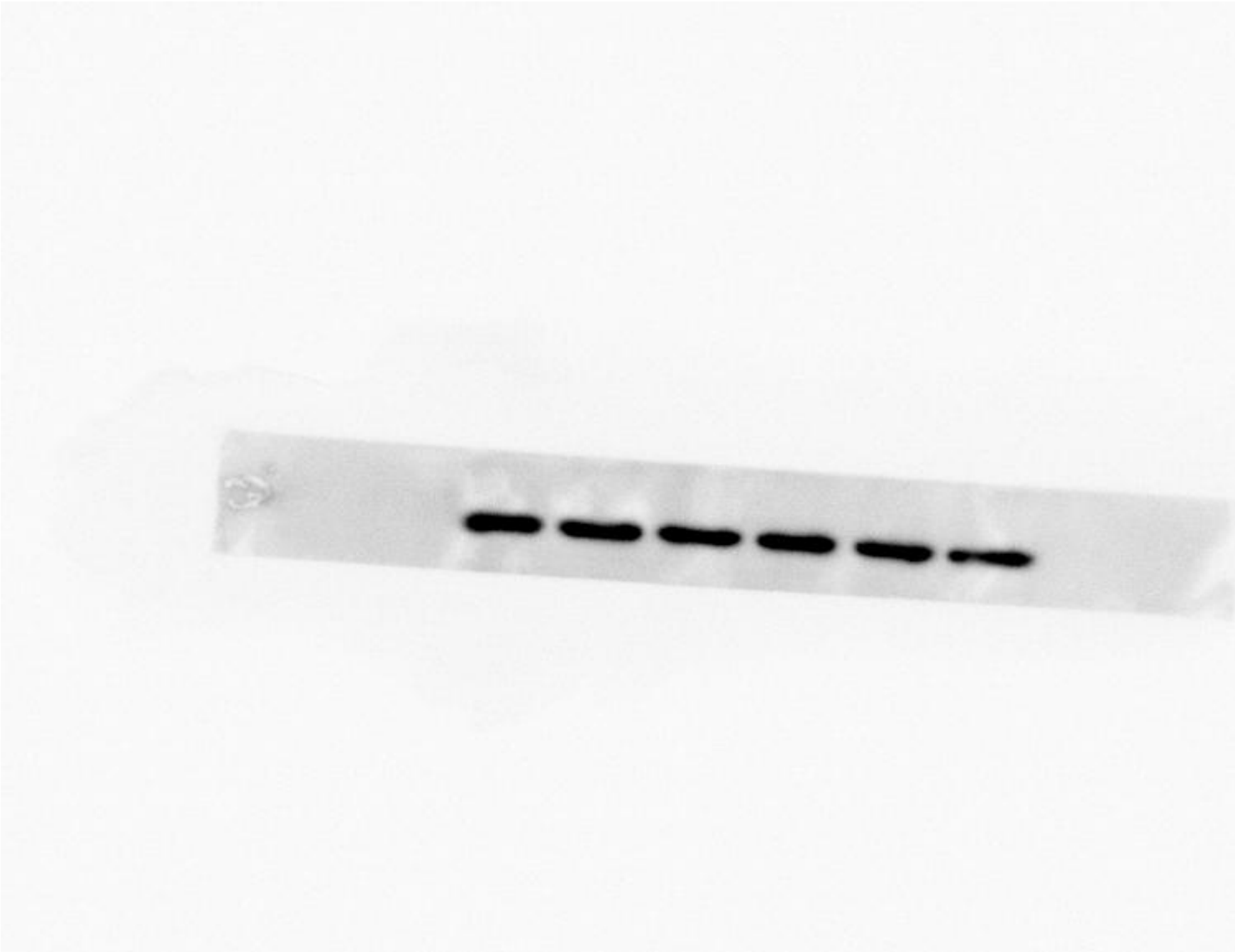

Fig7D

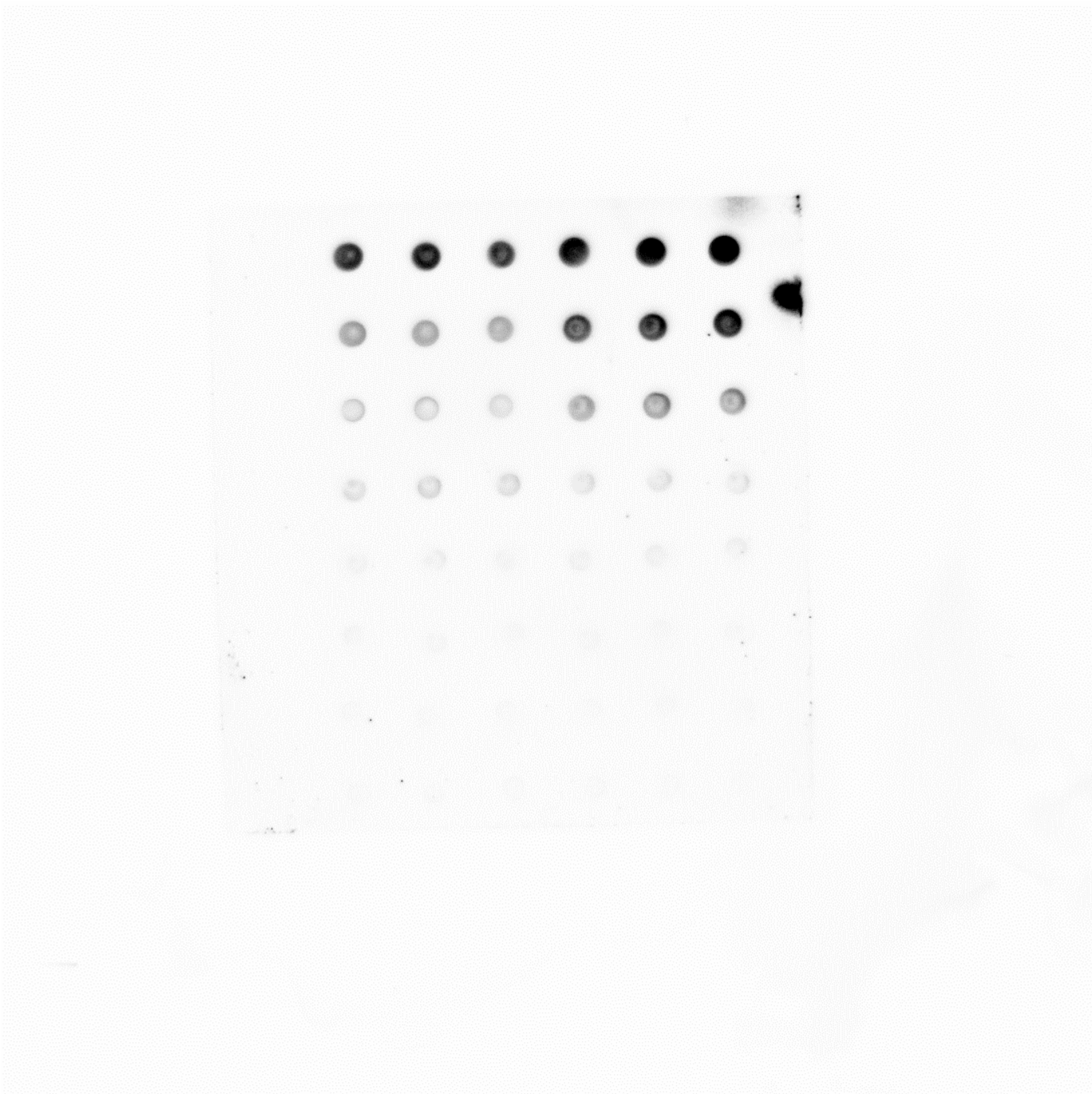

Fig8A

pp53

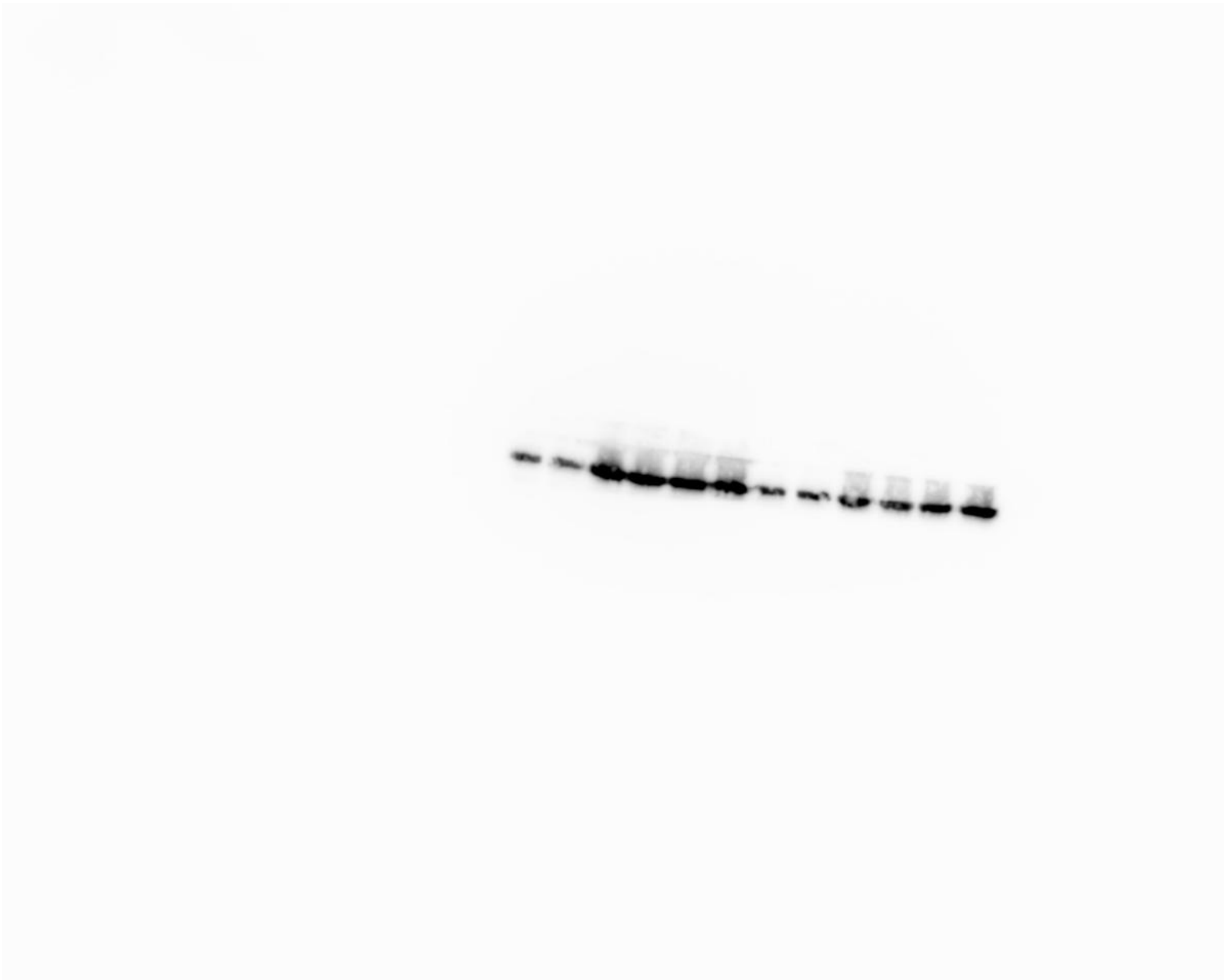

p53

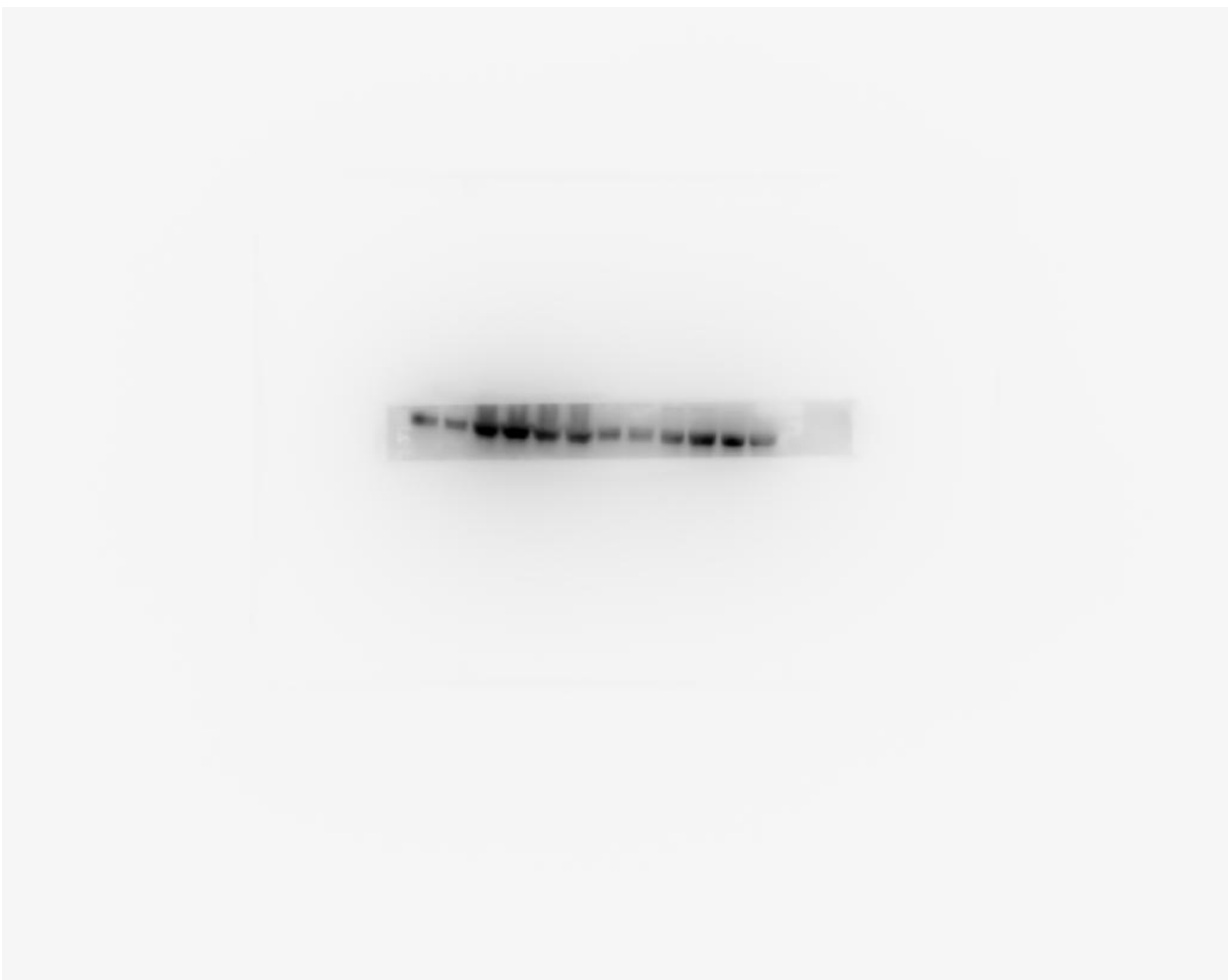

P21

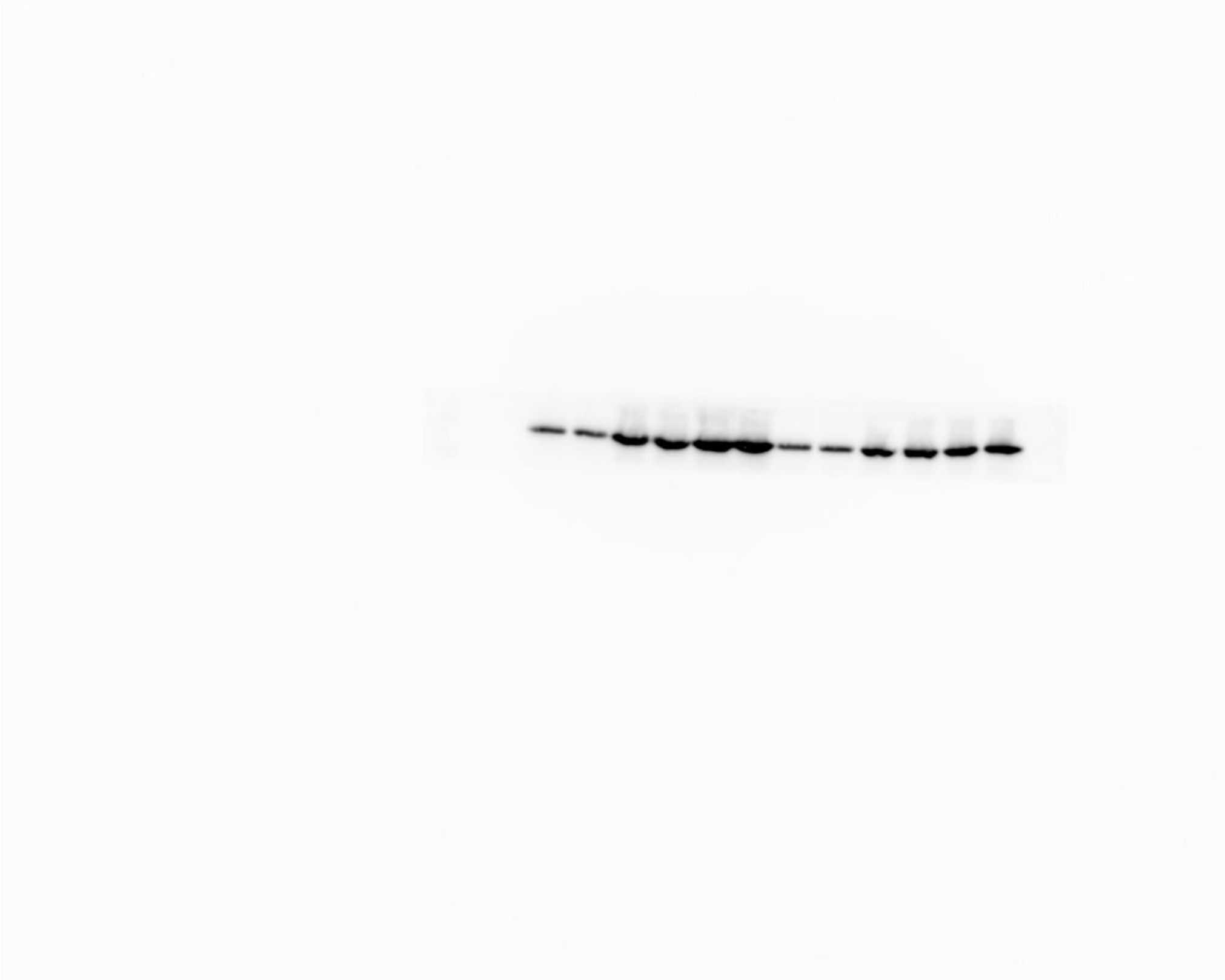

P16

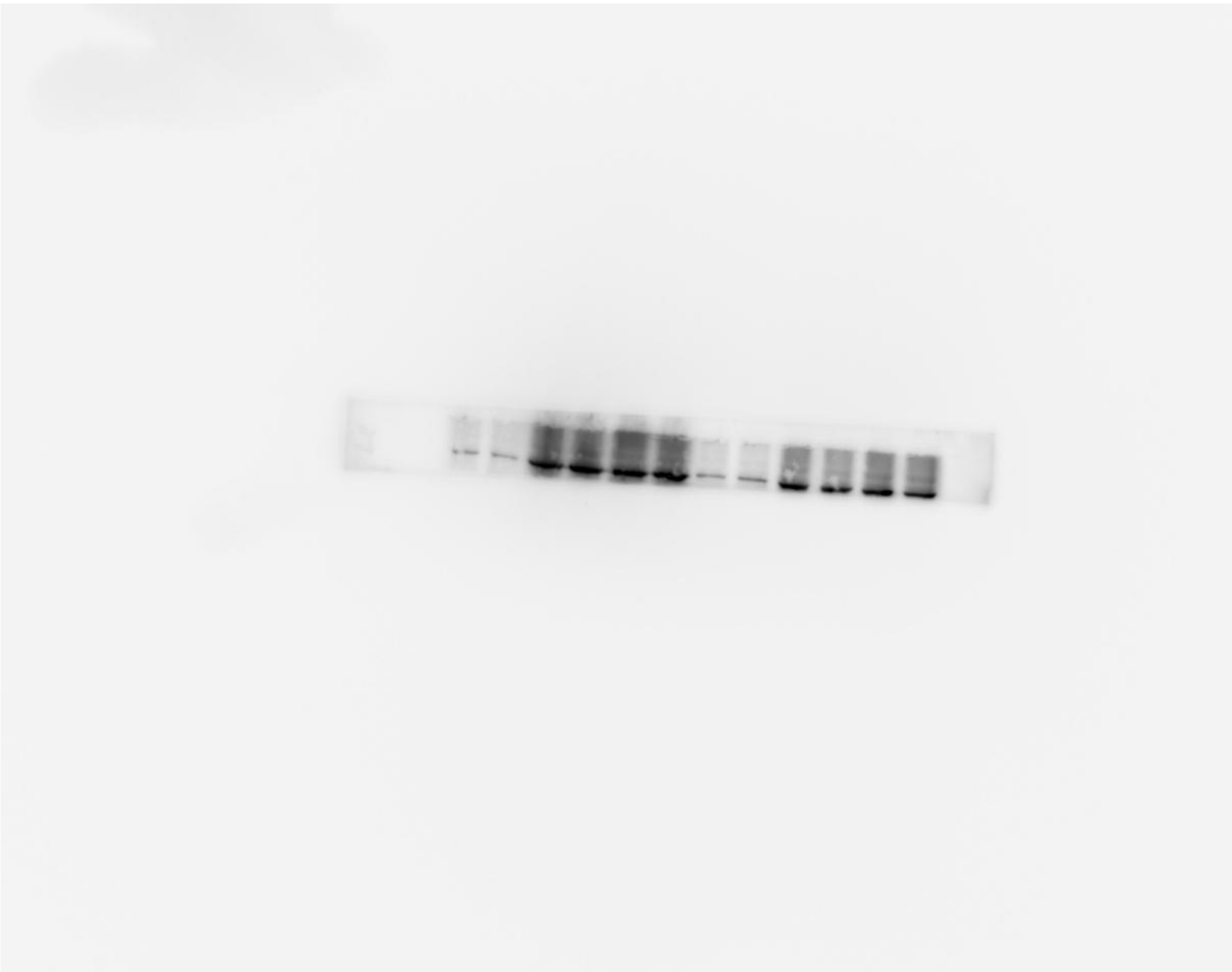

GAPDH

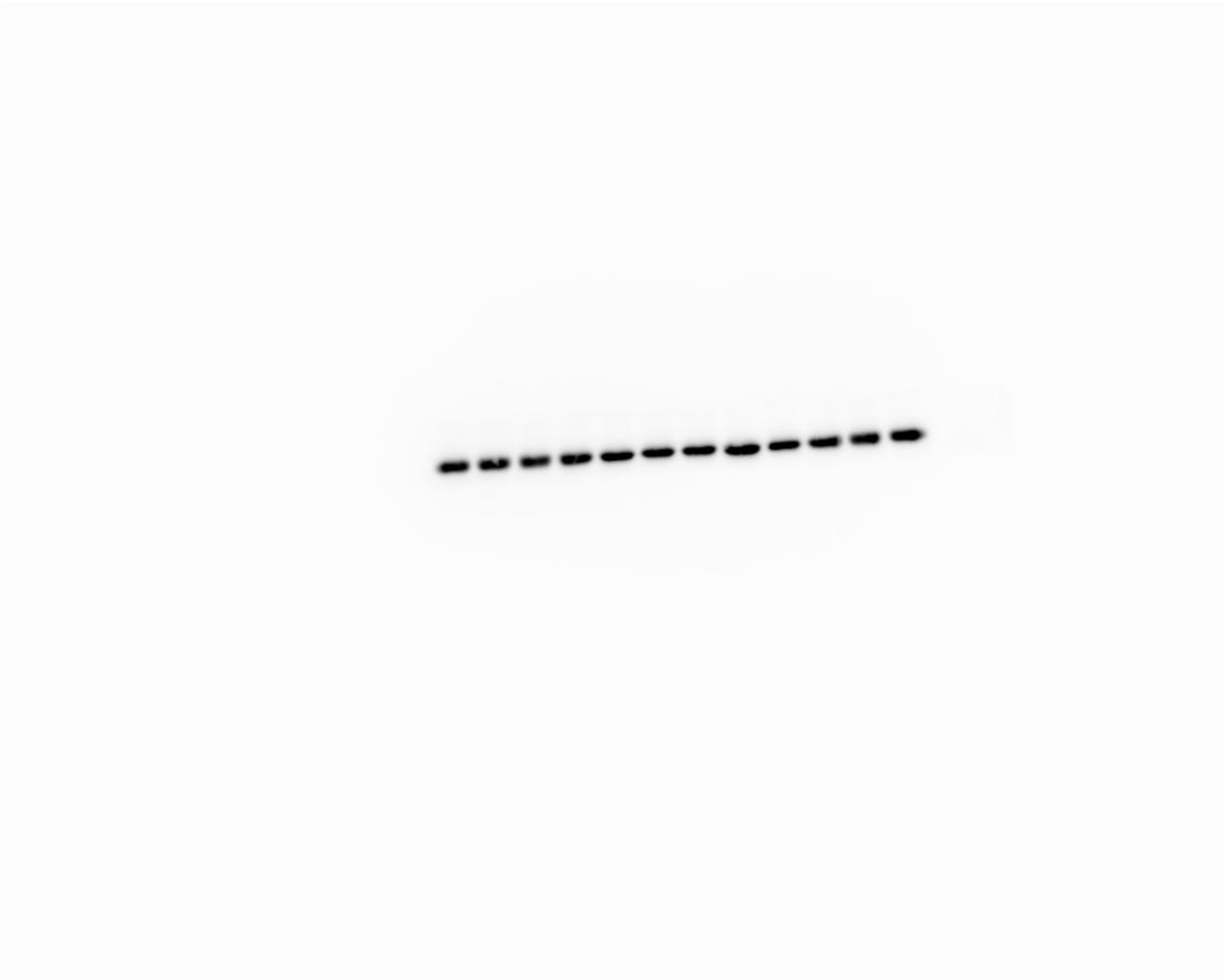

Fig8D

Flag-IP

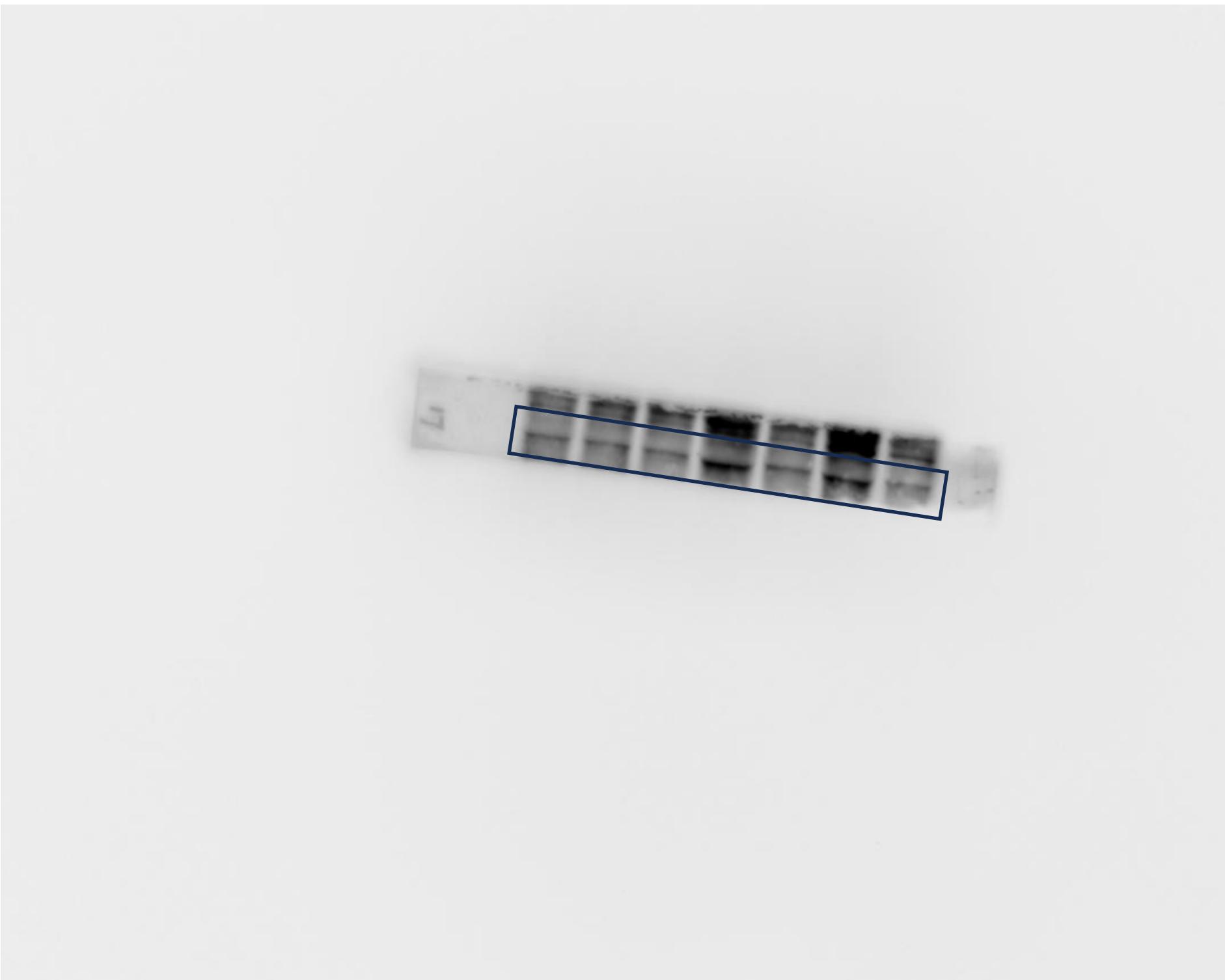

GFP

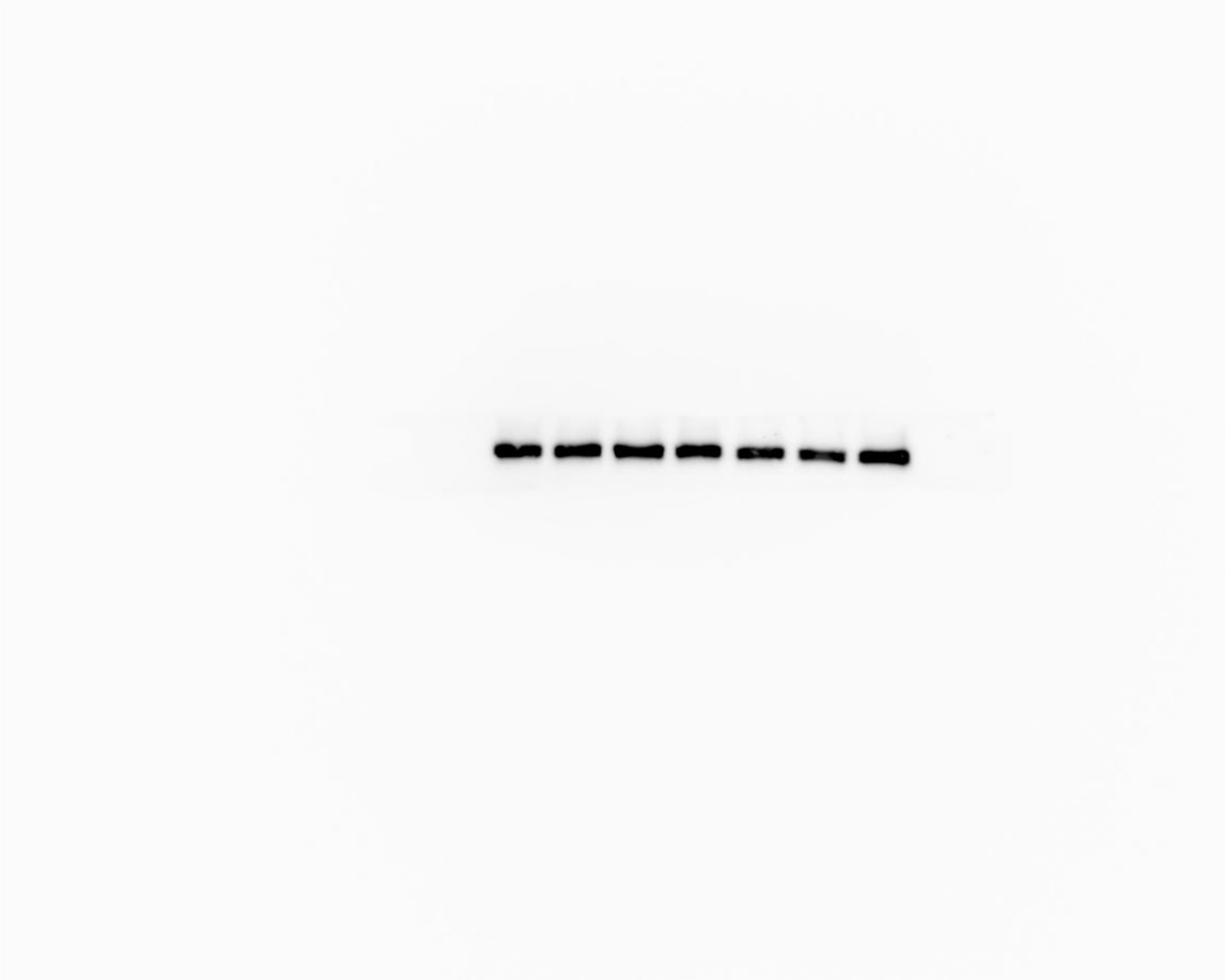

Flag-Input

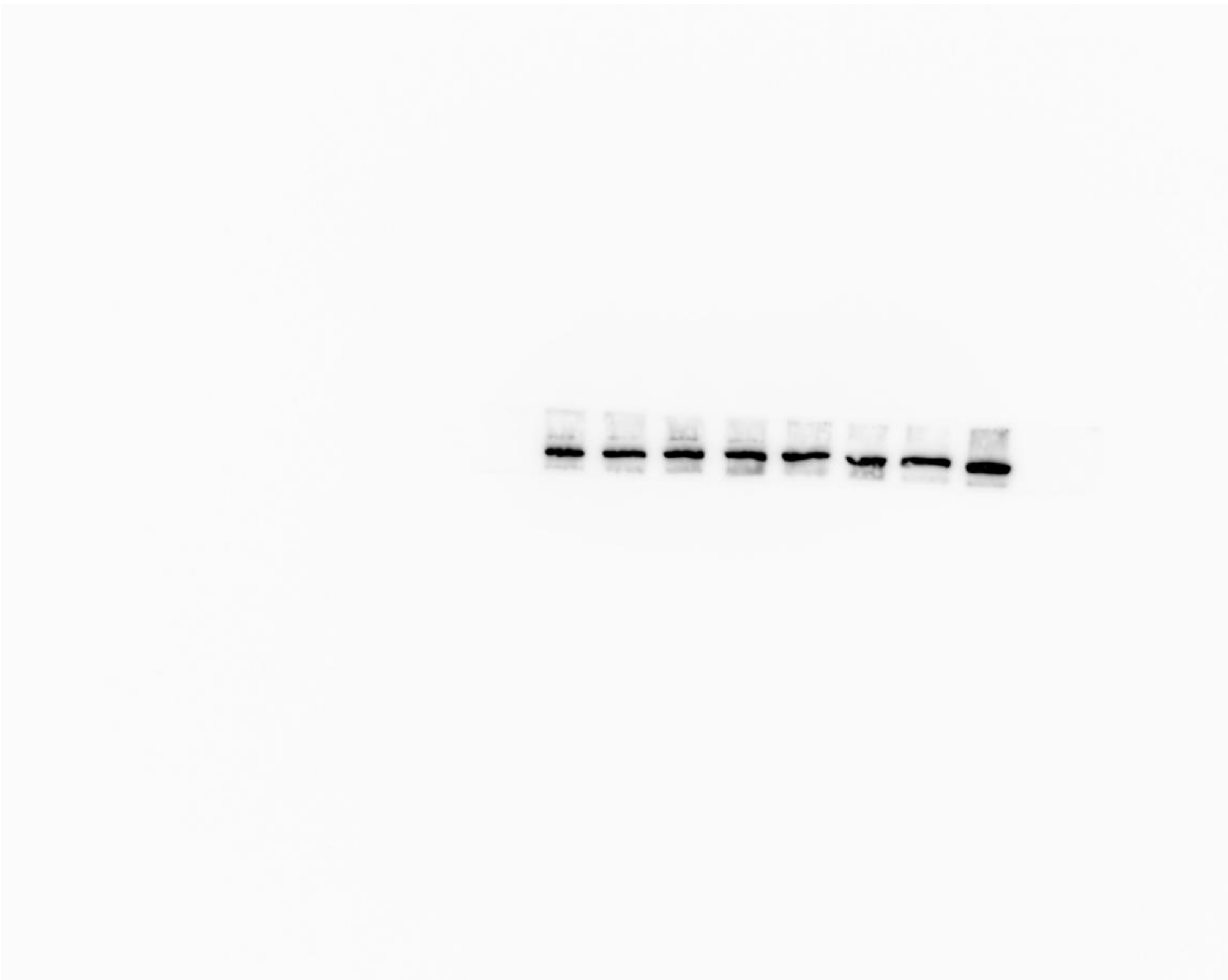

GAPDH

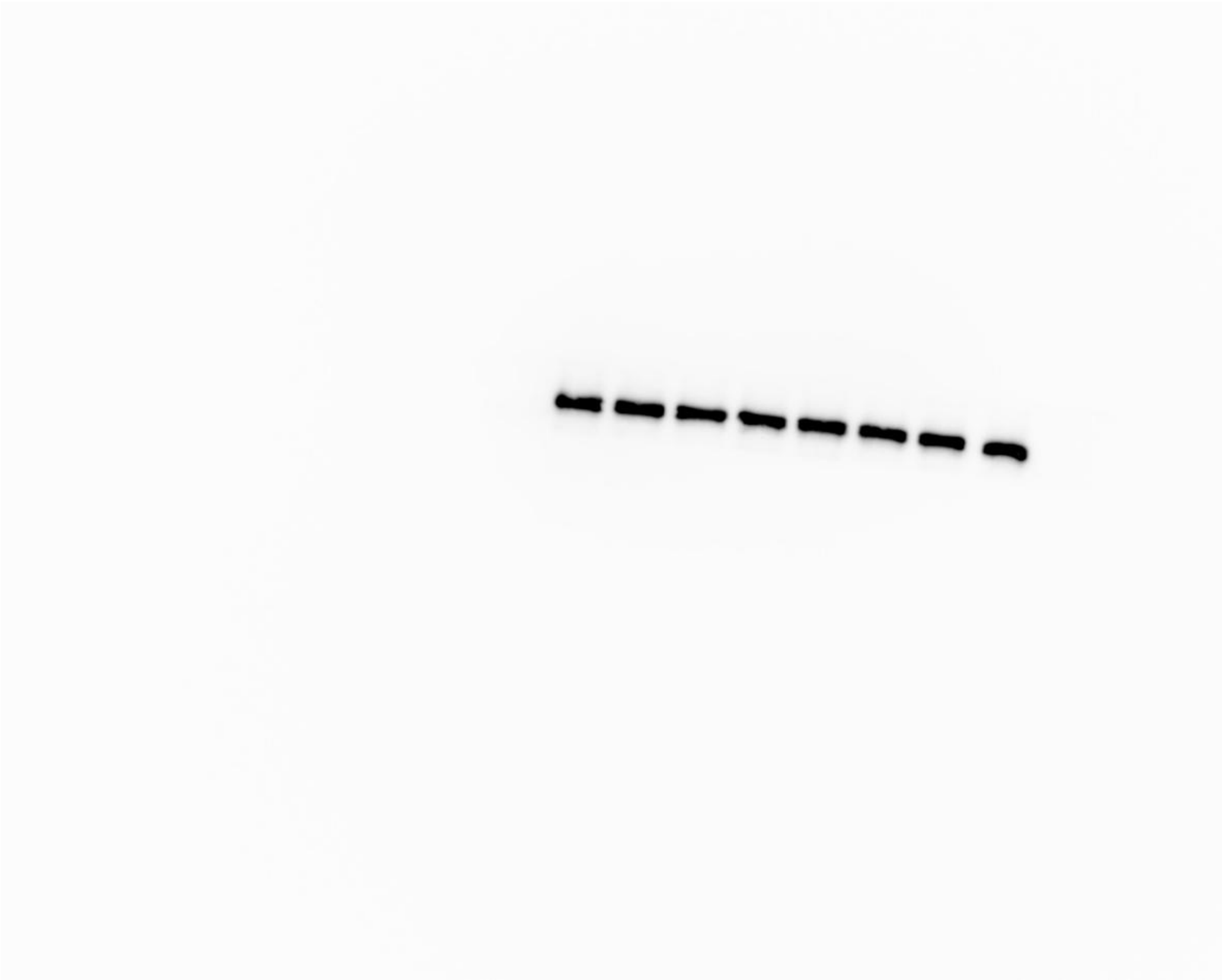

Fig8E

WT-MED12

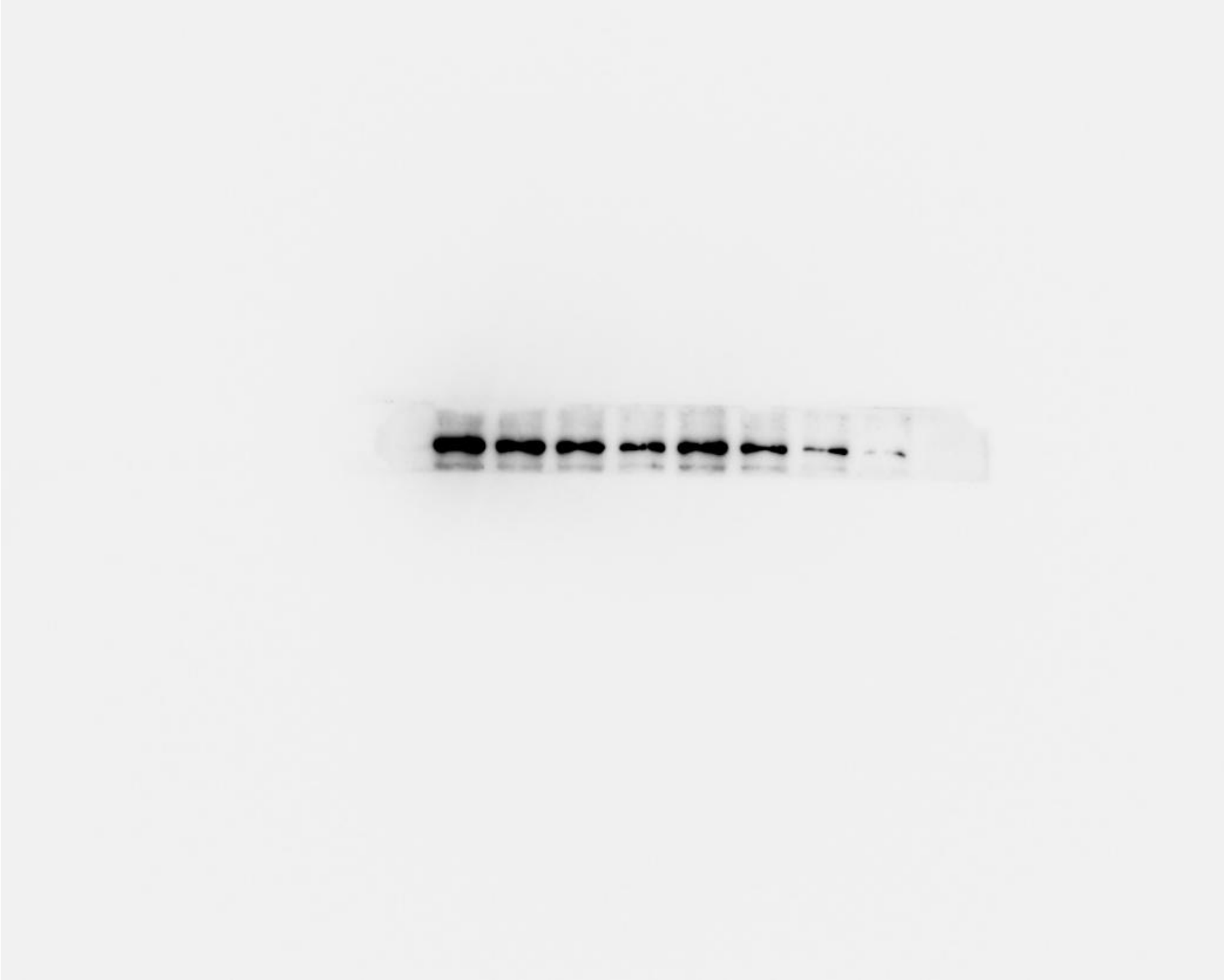

WT-GAPDH

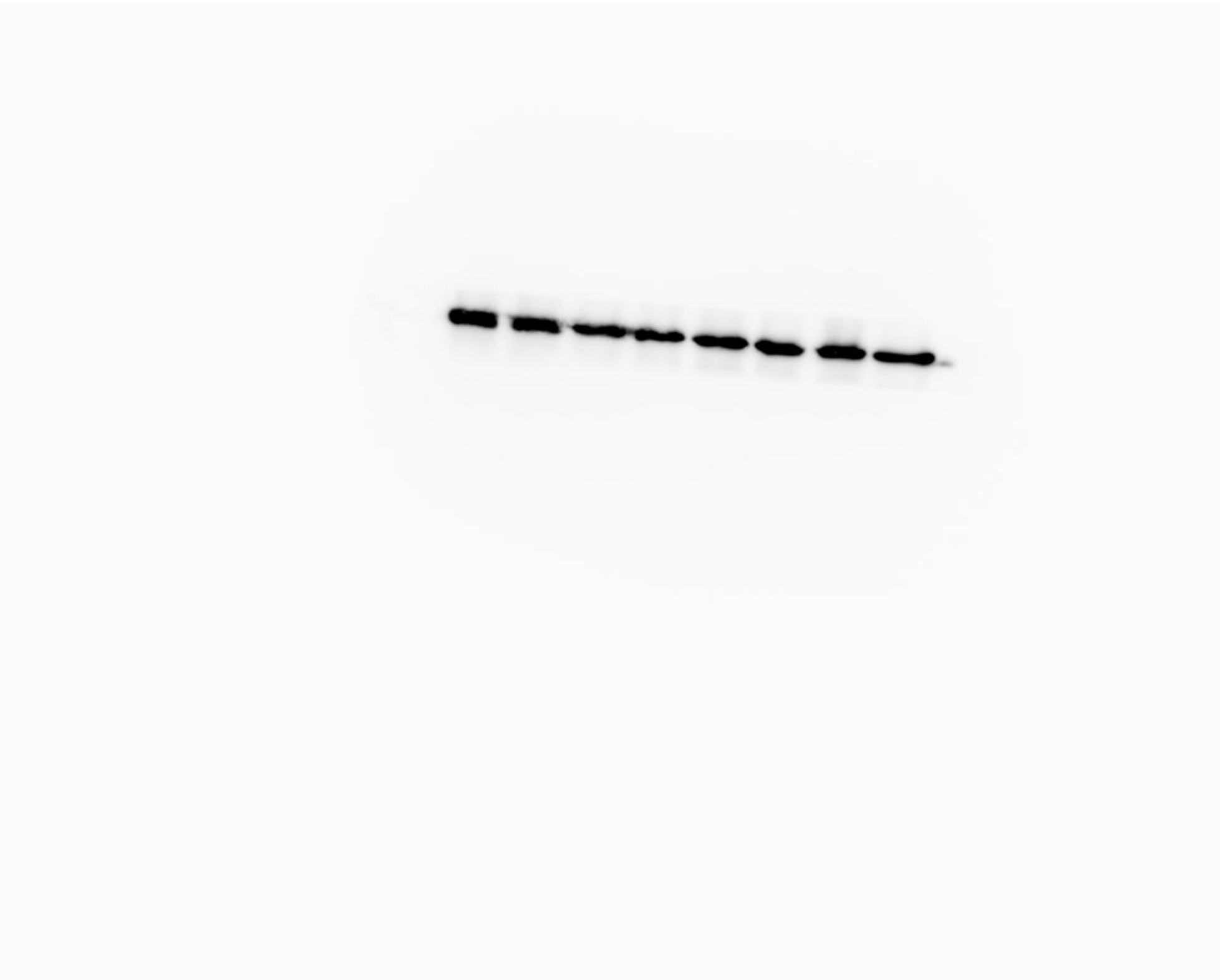

RF-MED12

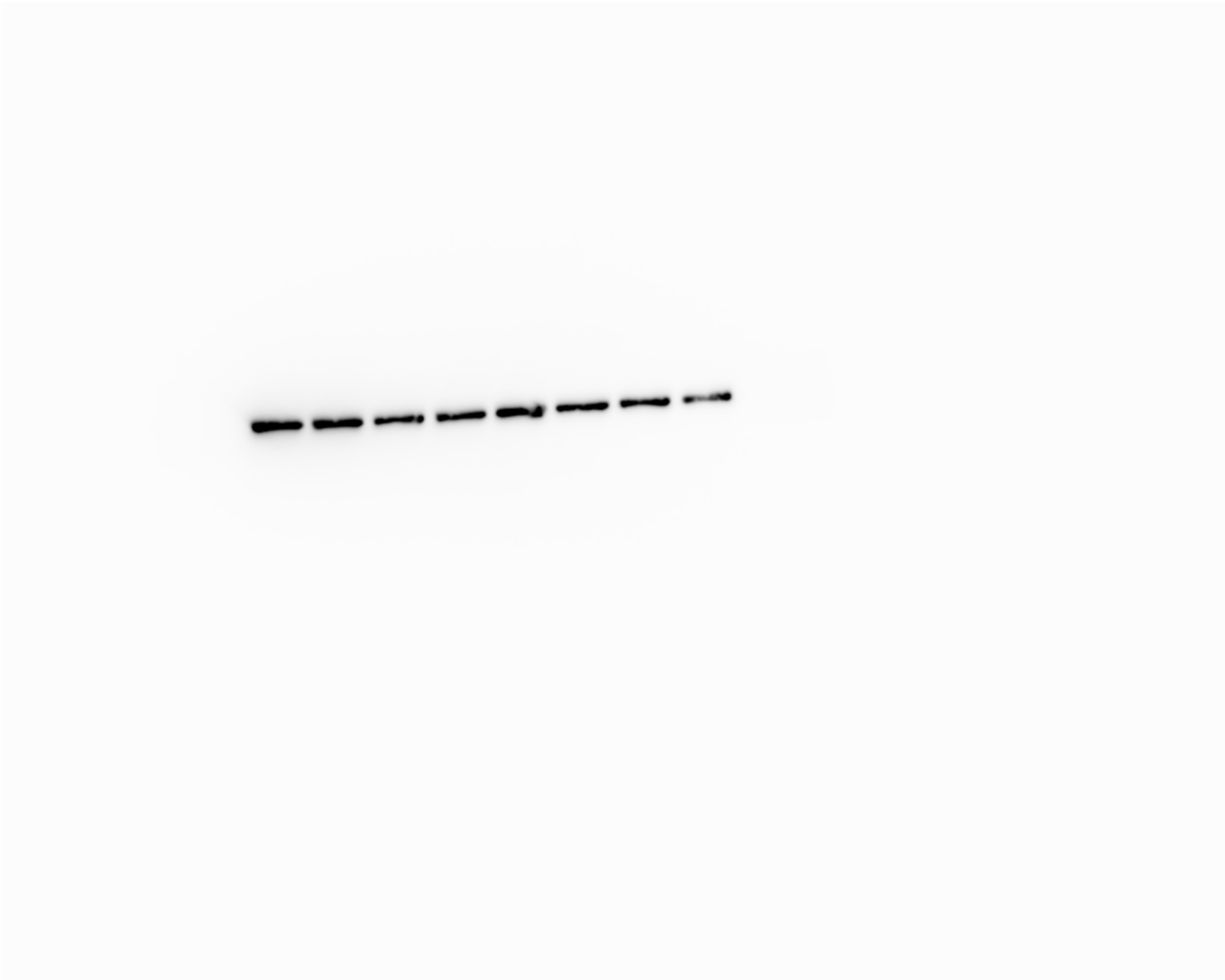

RF-GAPDH

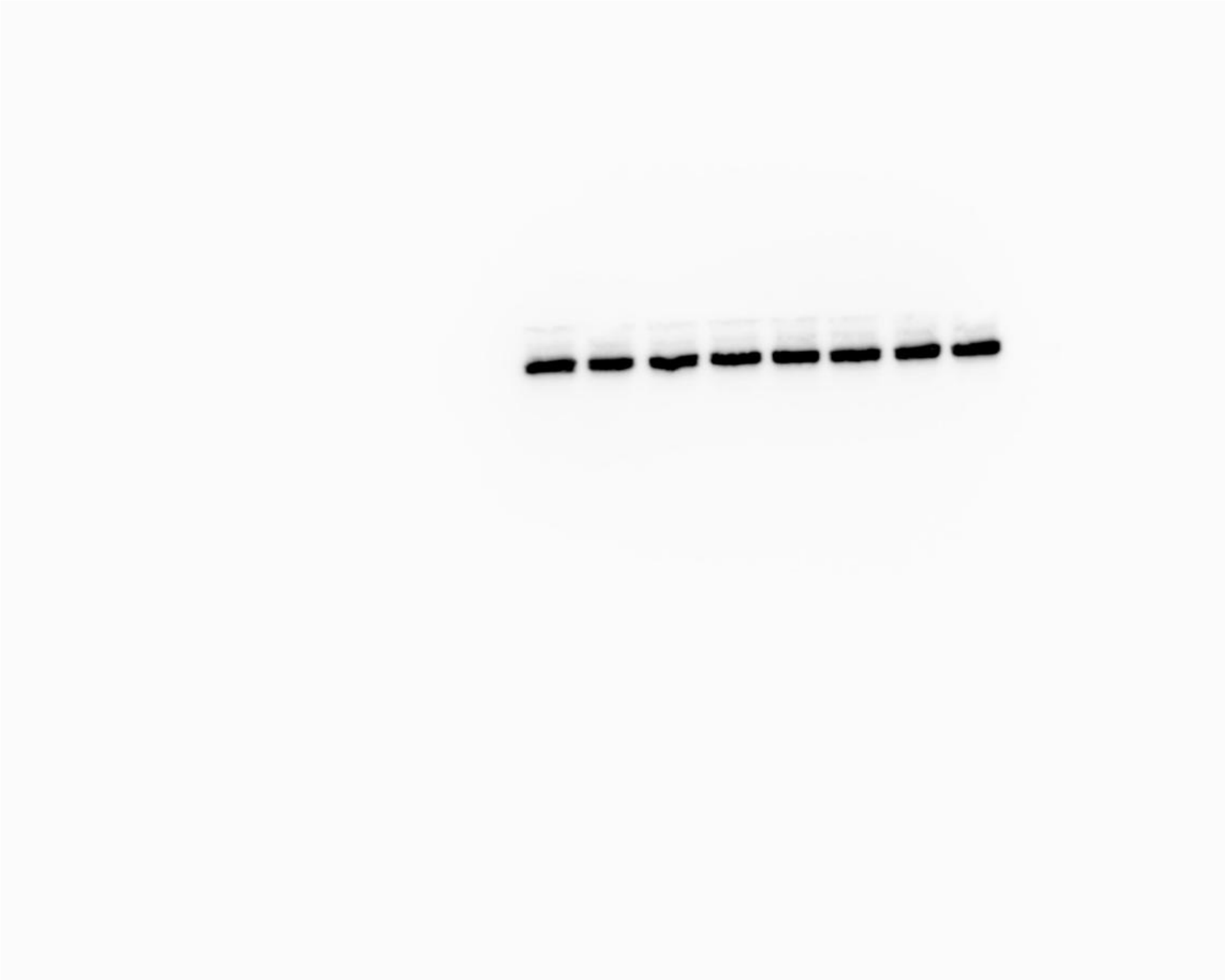

Fig8G

MED12-IP

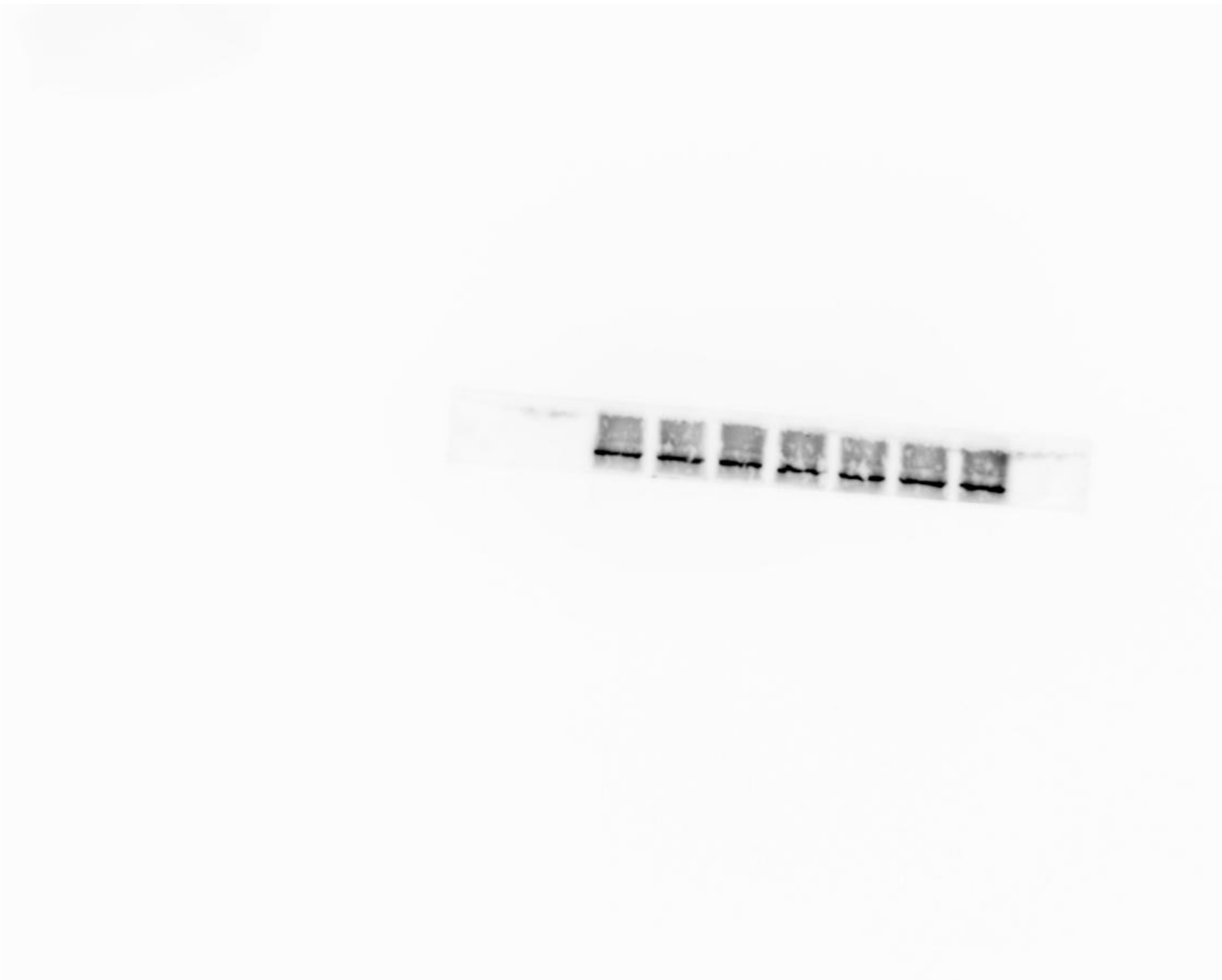

Ub-IP

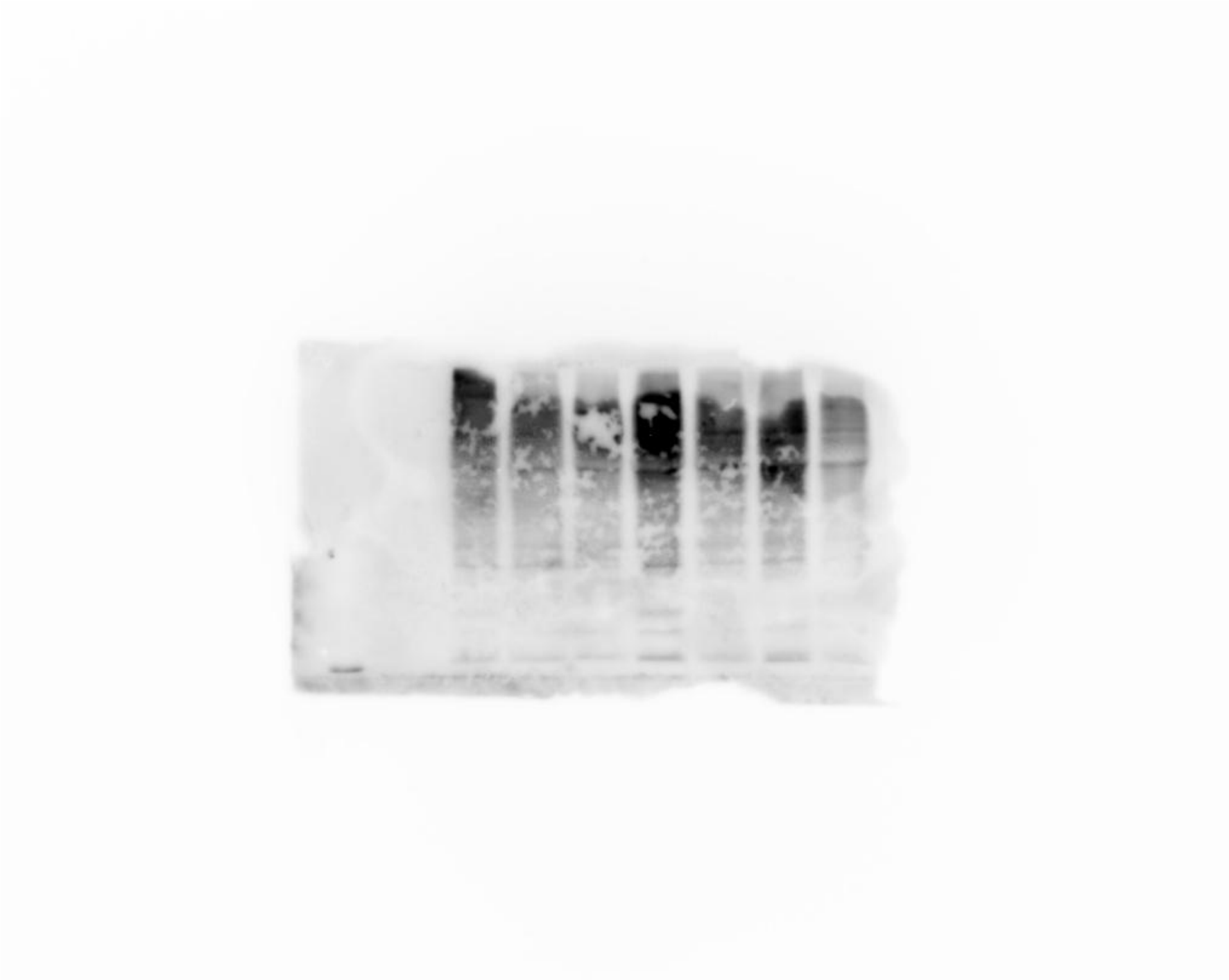

MED12-Input

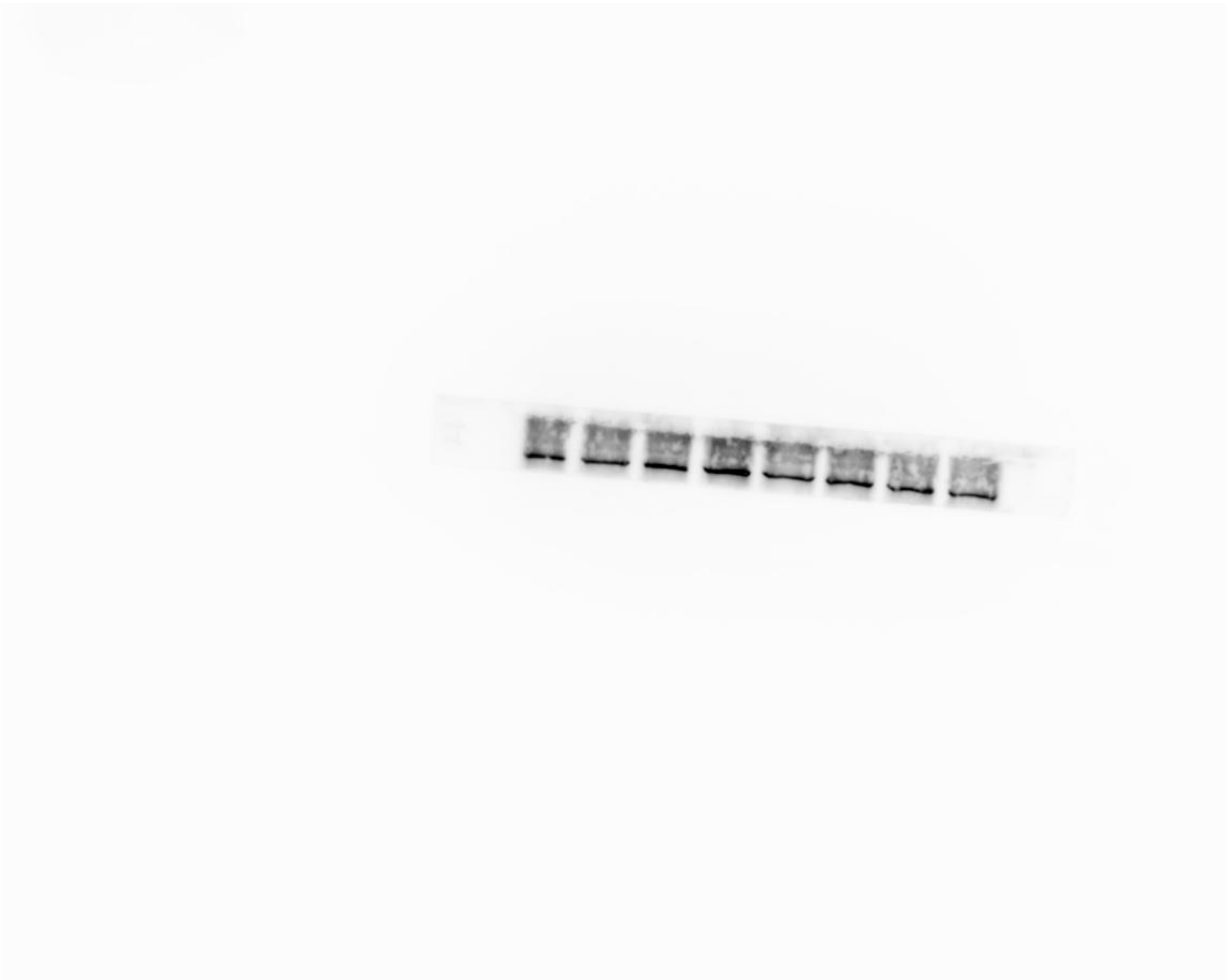

GAPDH

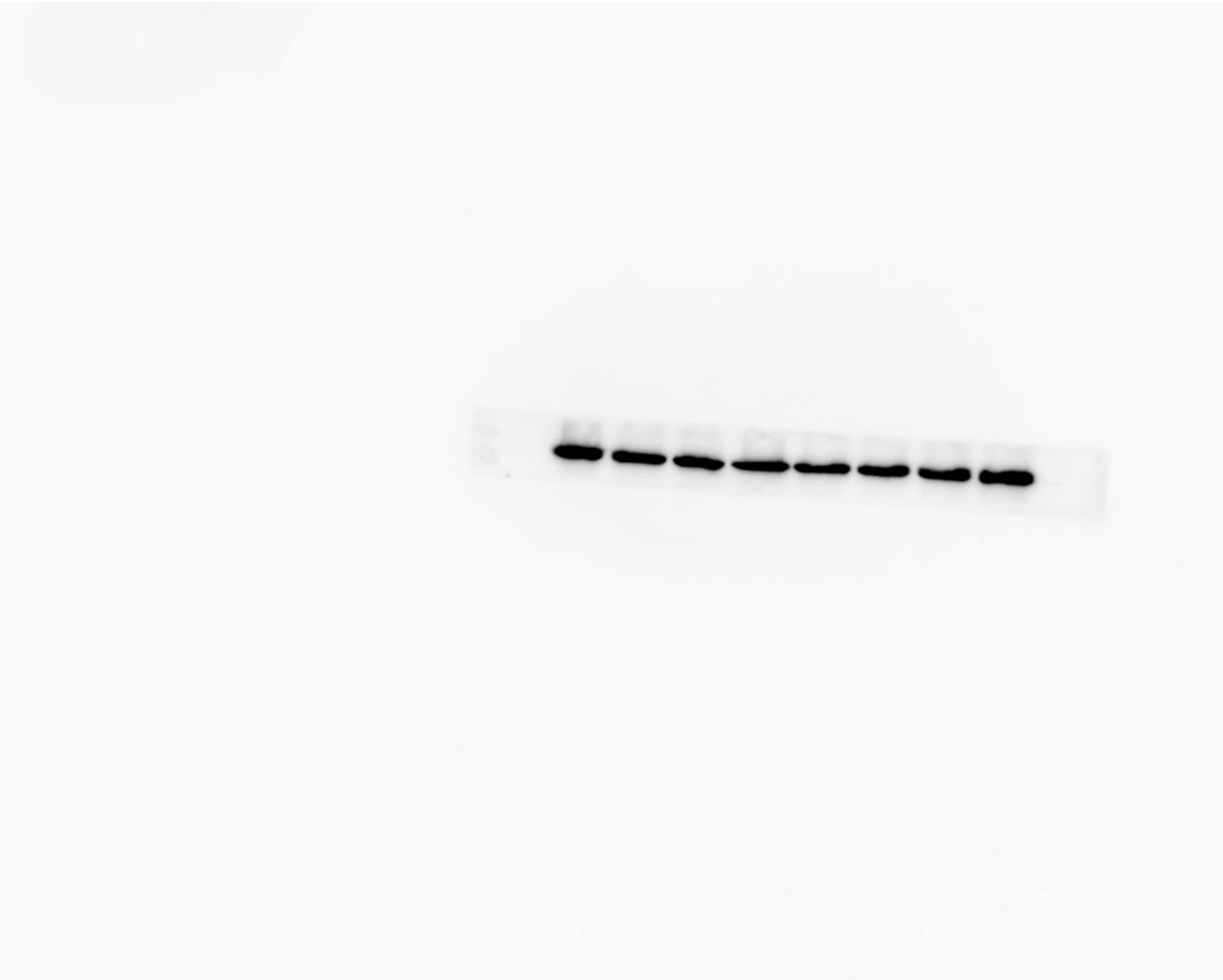

Fig8H

PP53

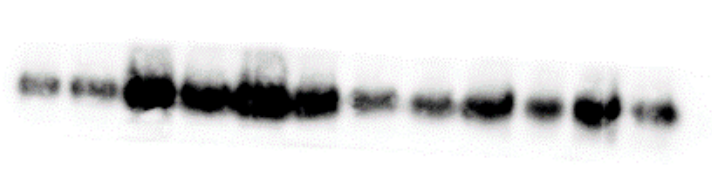

P53

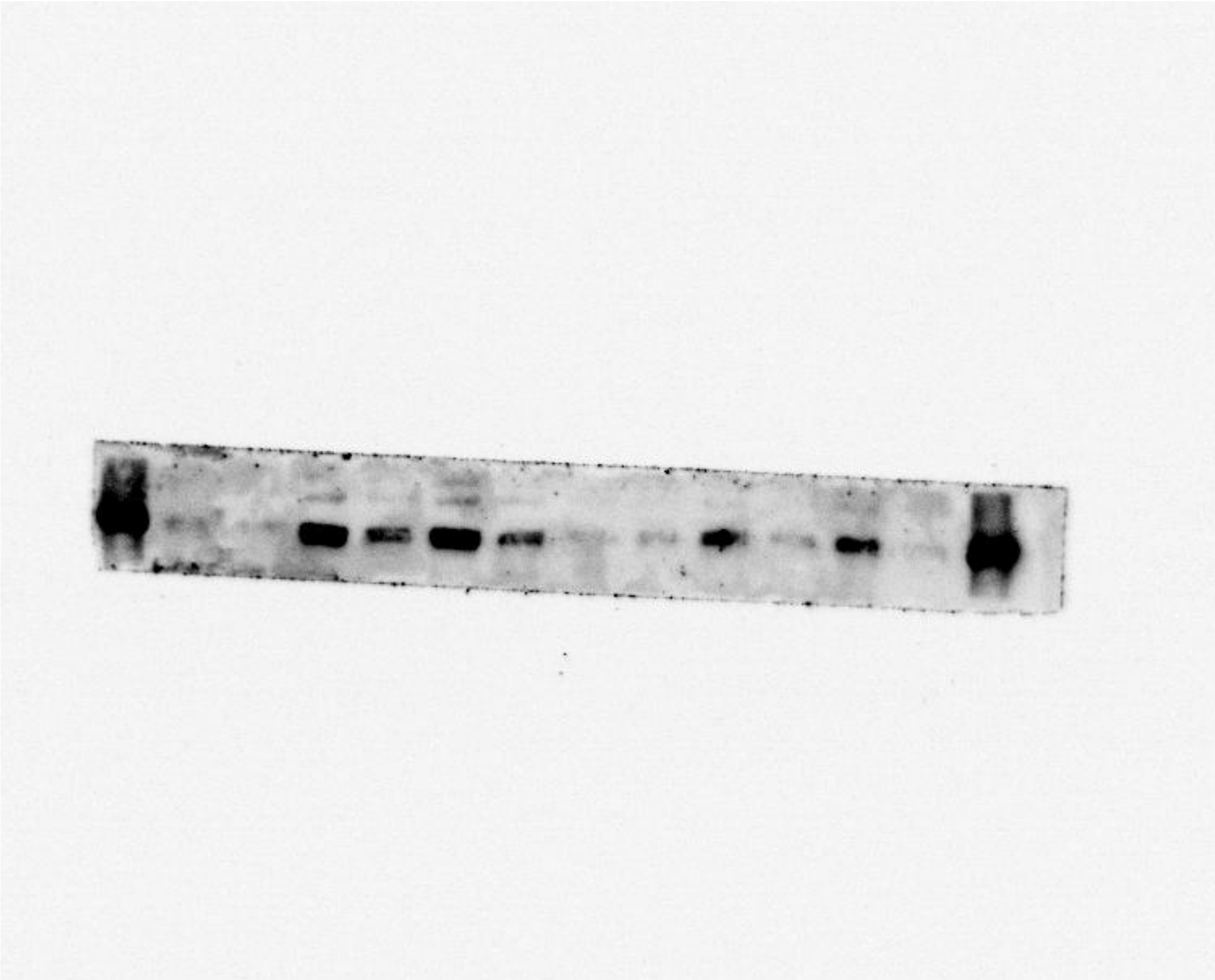

P21

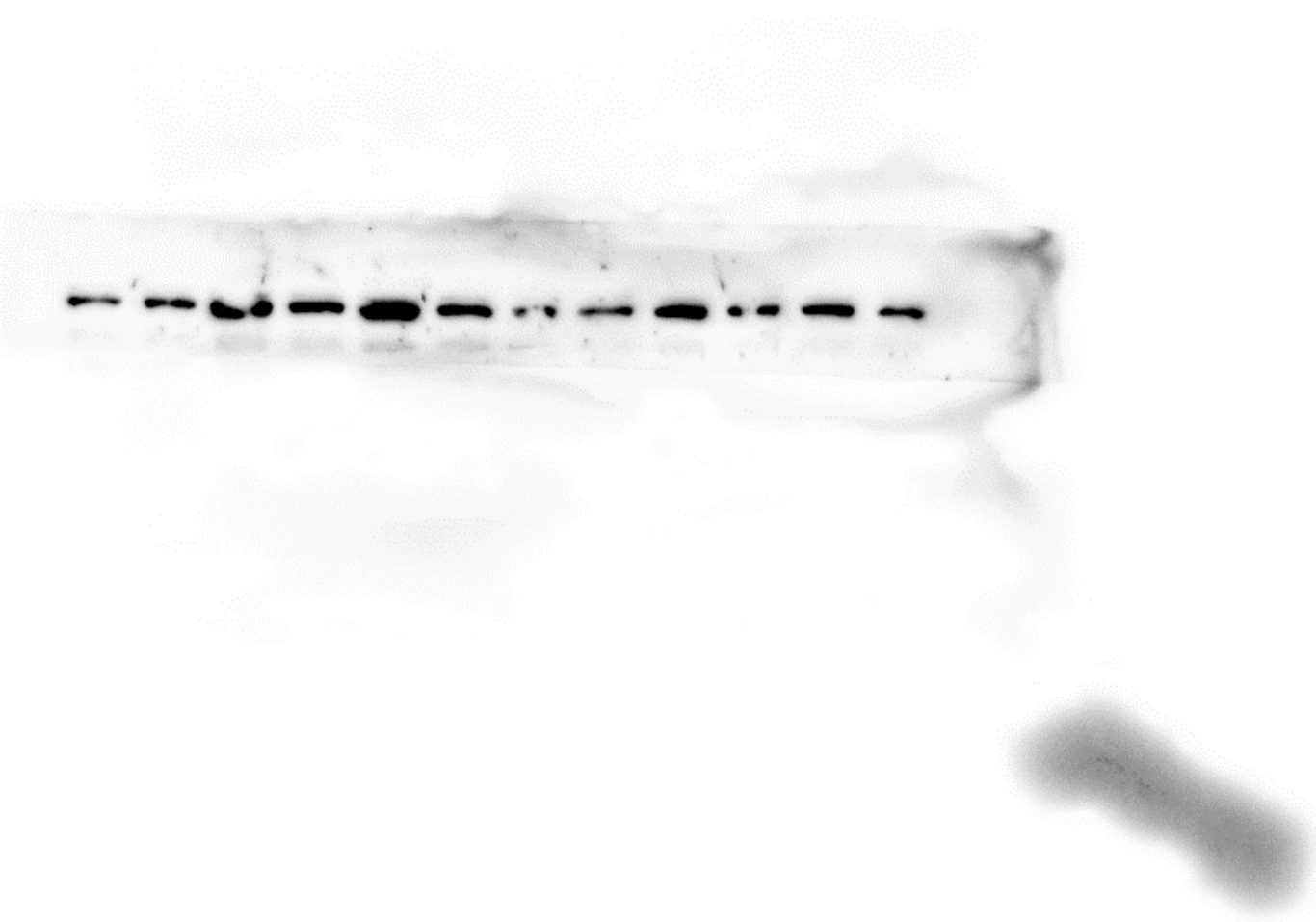

P16

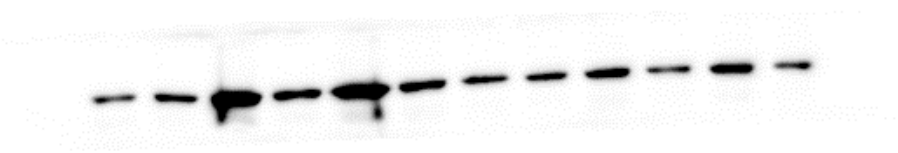

p-TBK1

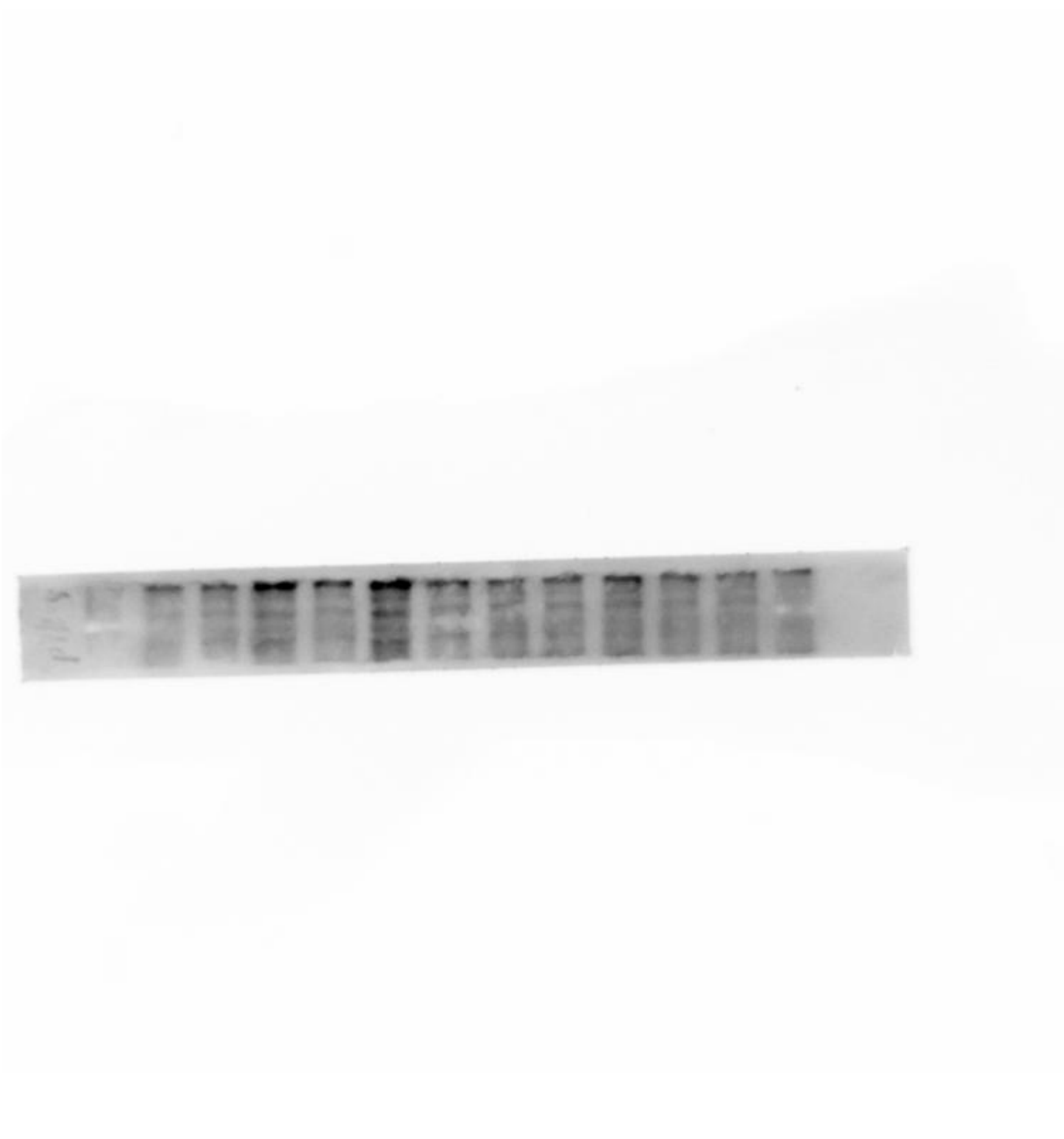

TBK1

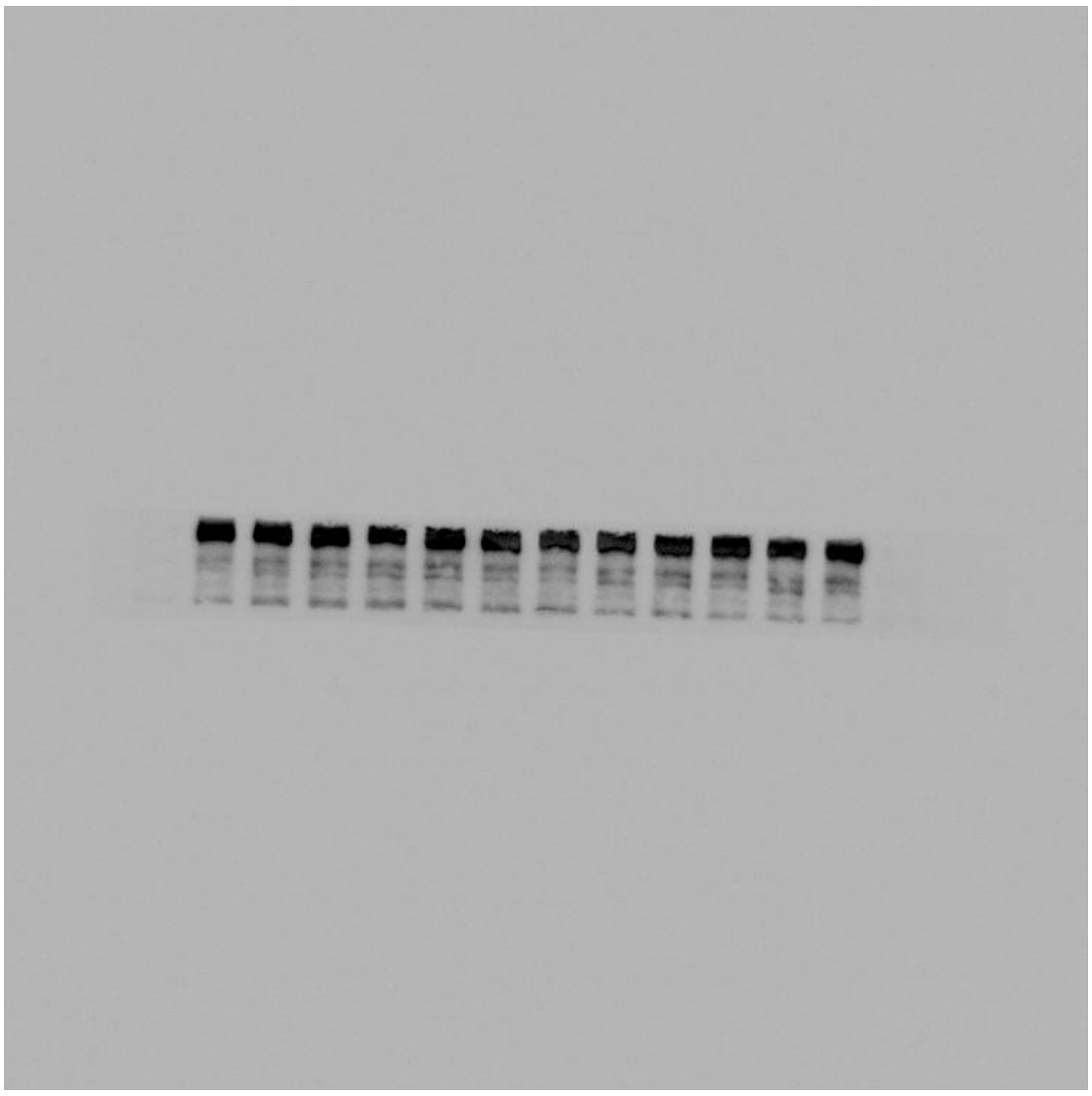

p-IRF3

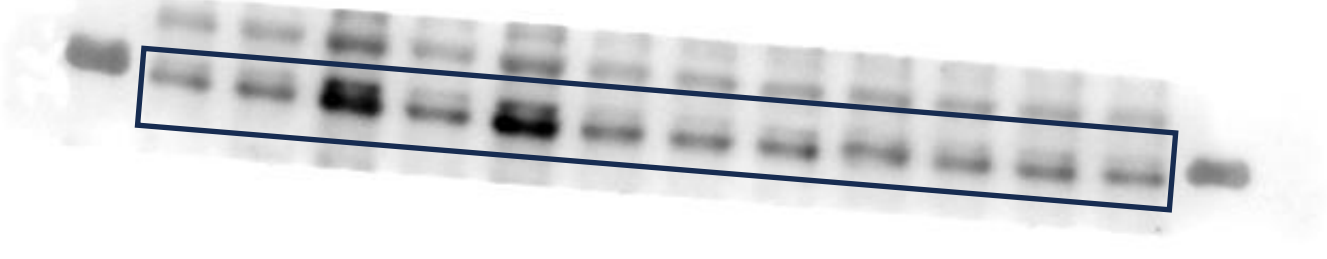

IRF3

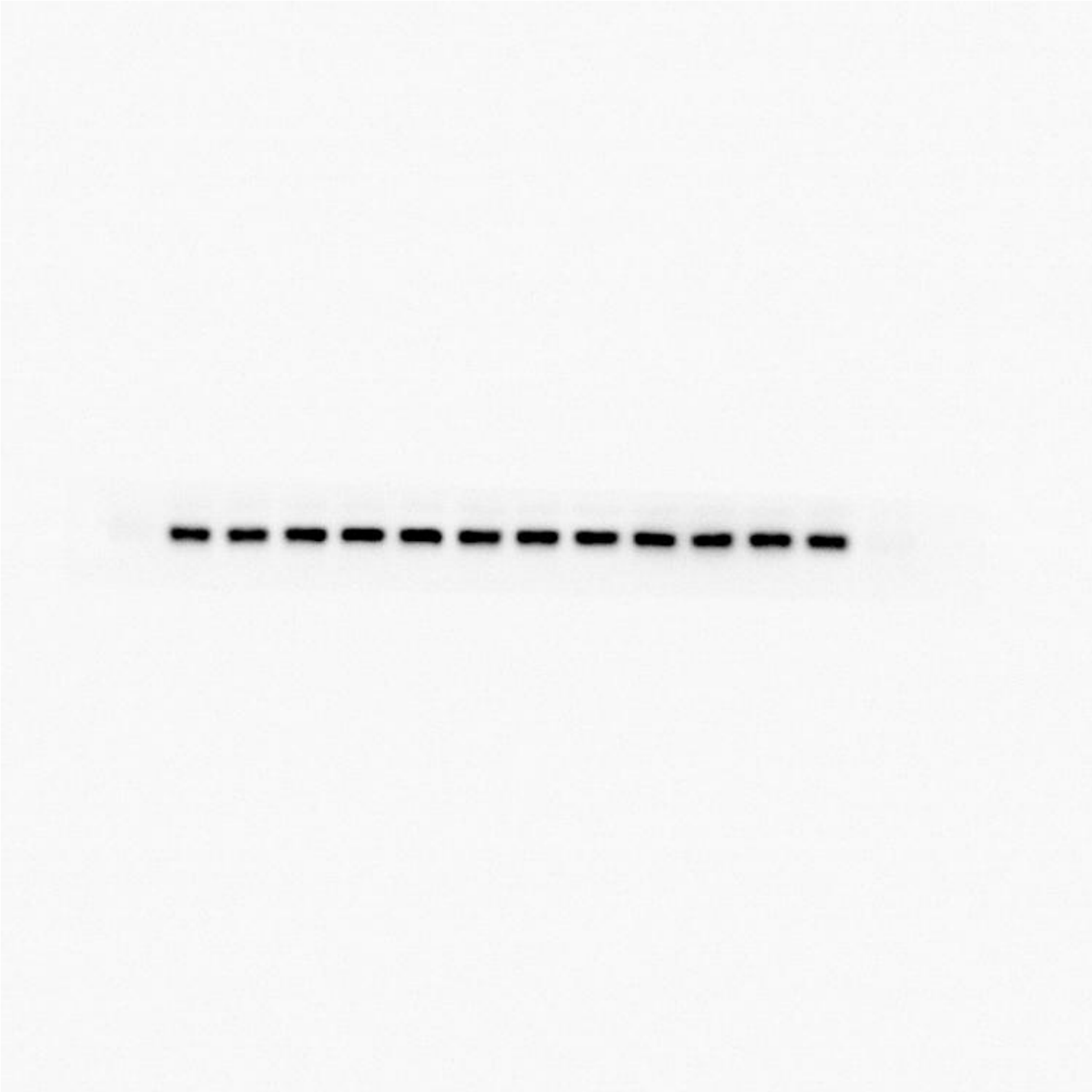

STING

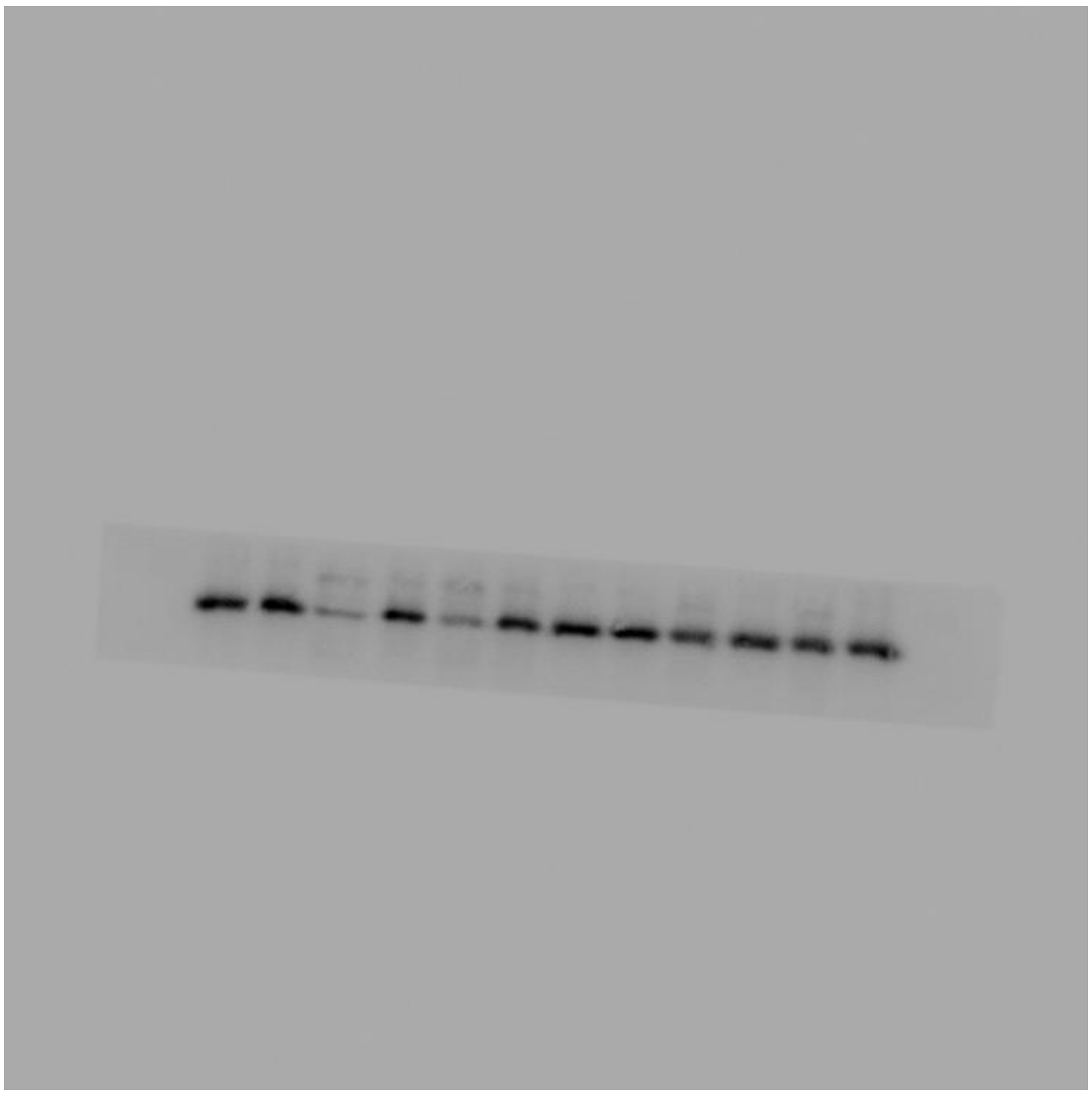

GAPDH

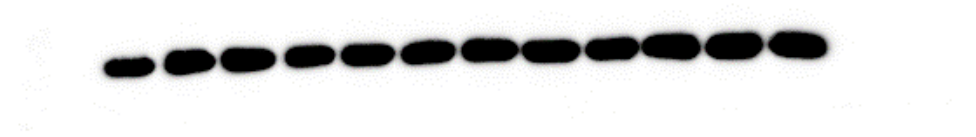

Fig9B

TSG101

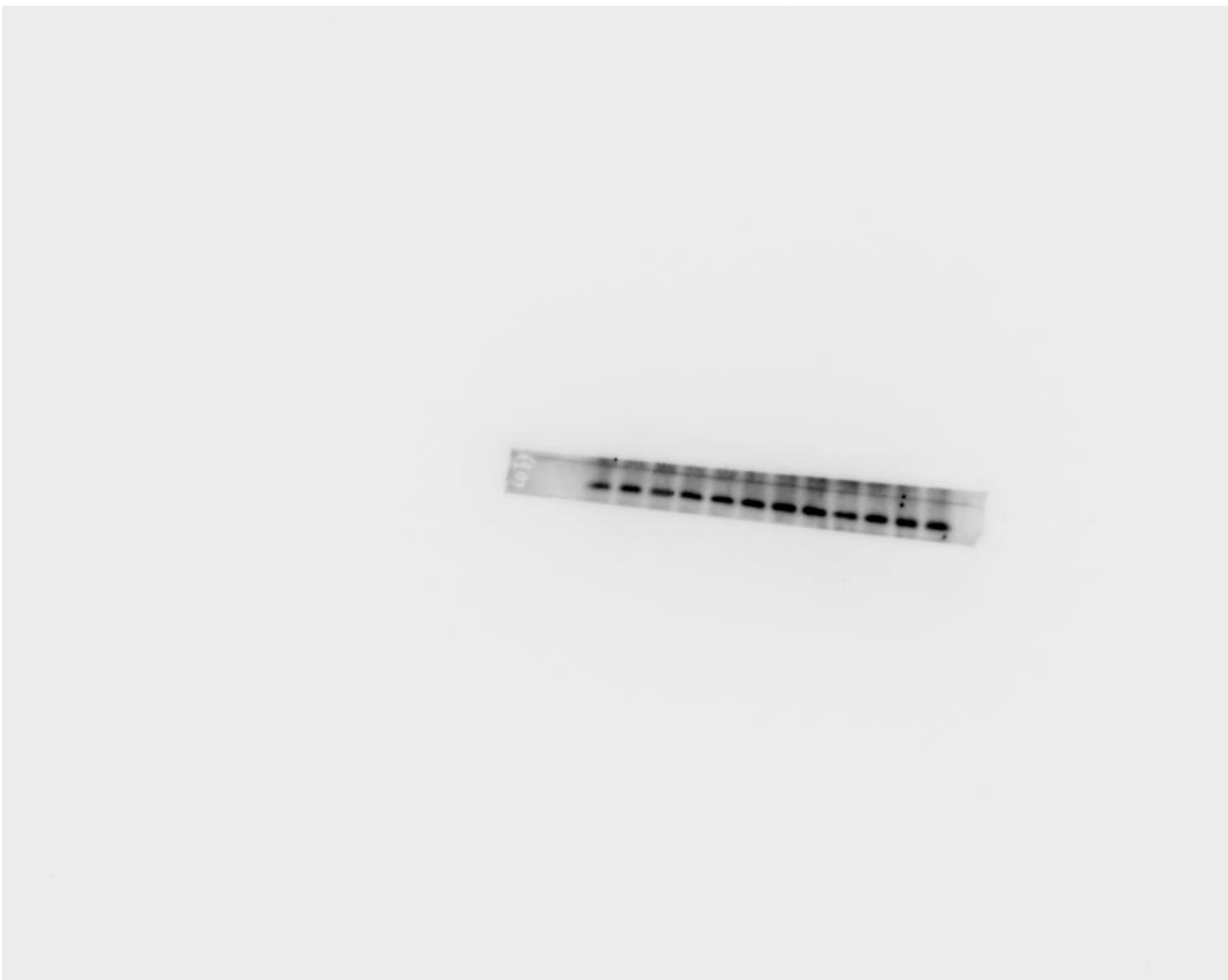

CD63

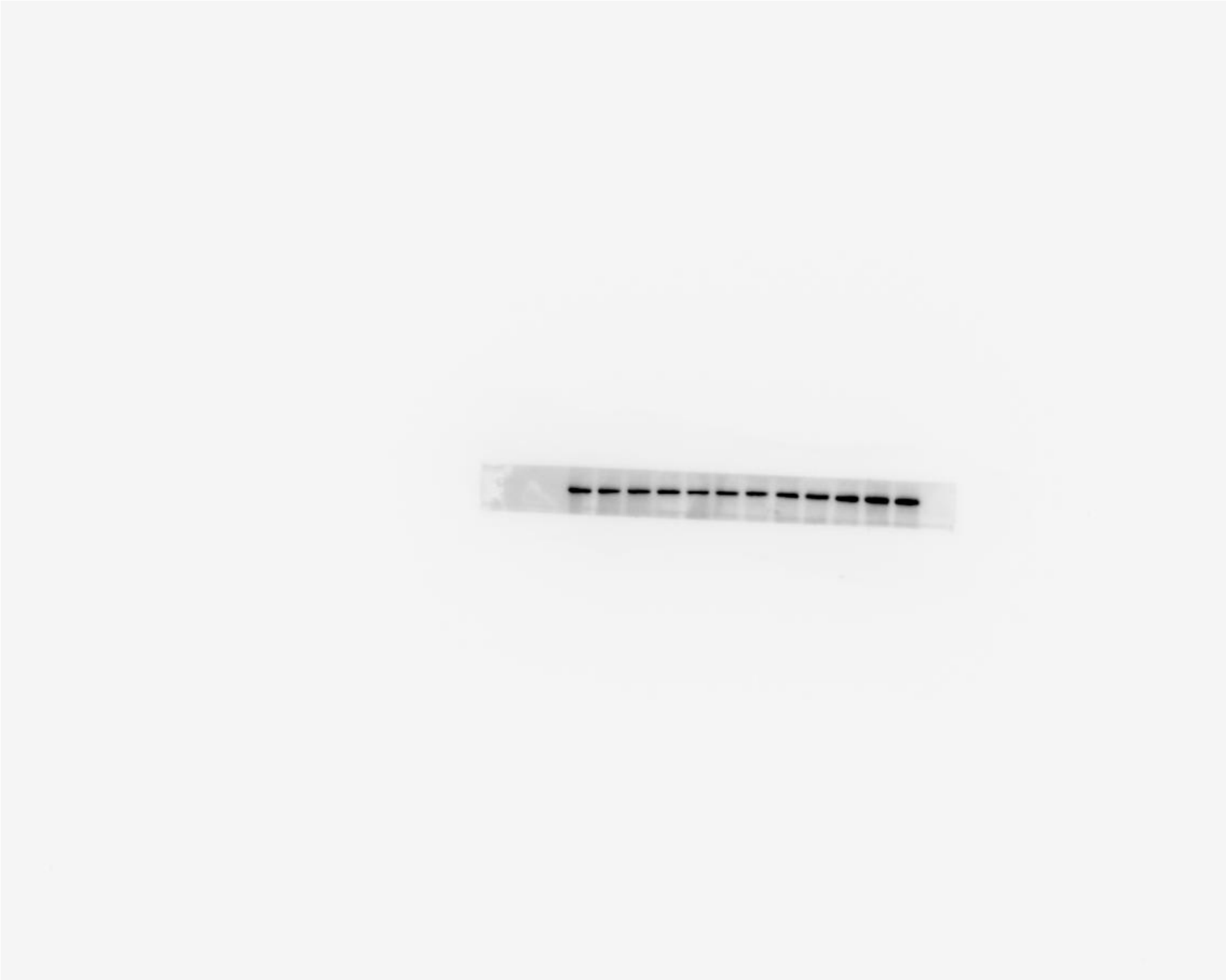

CD9

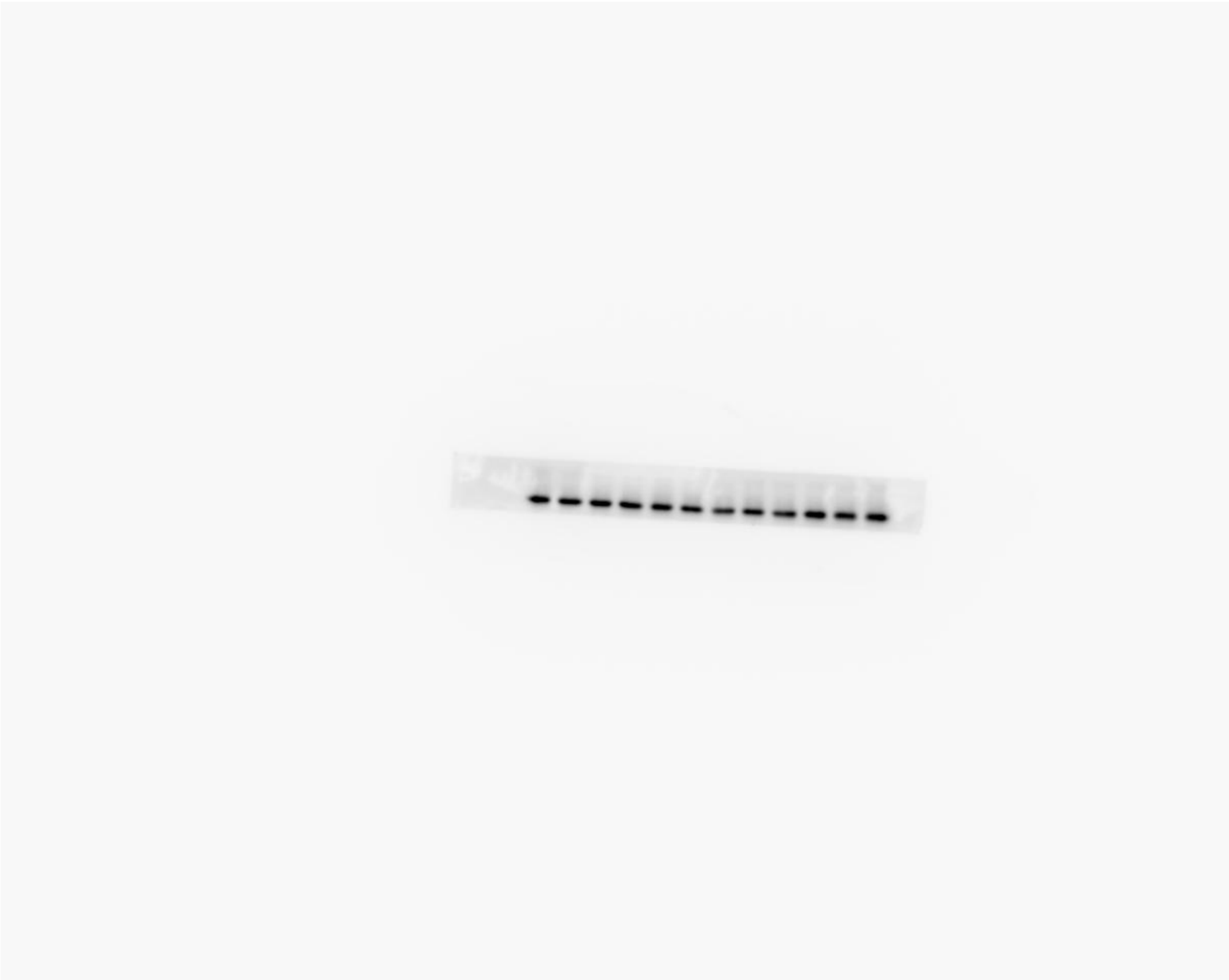

GAPDH

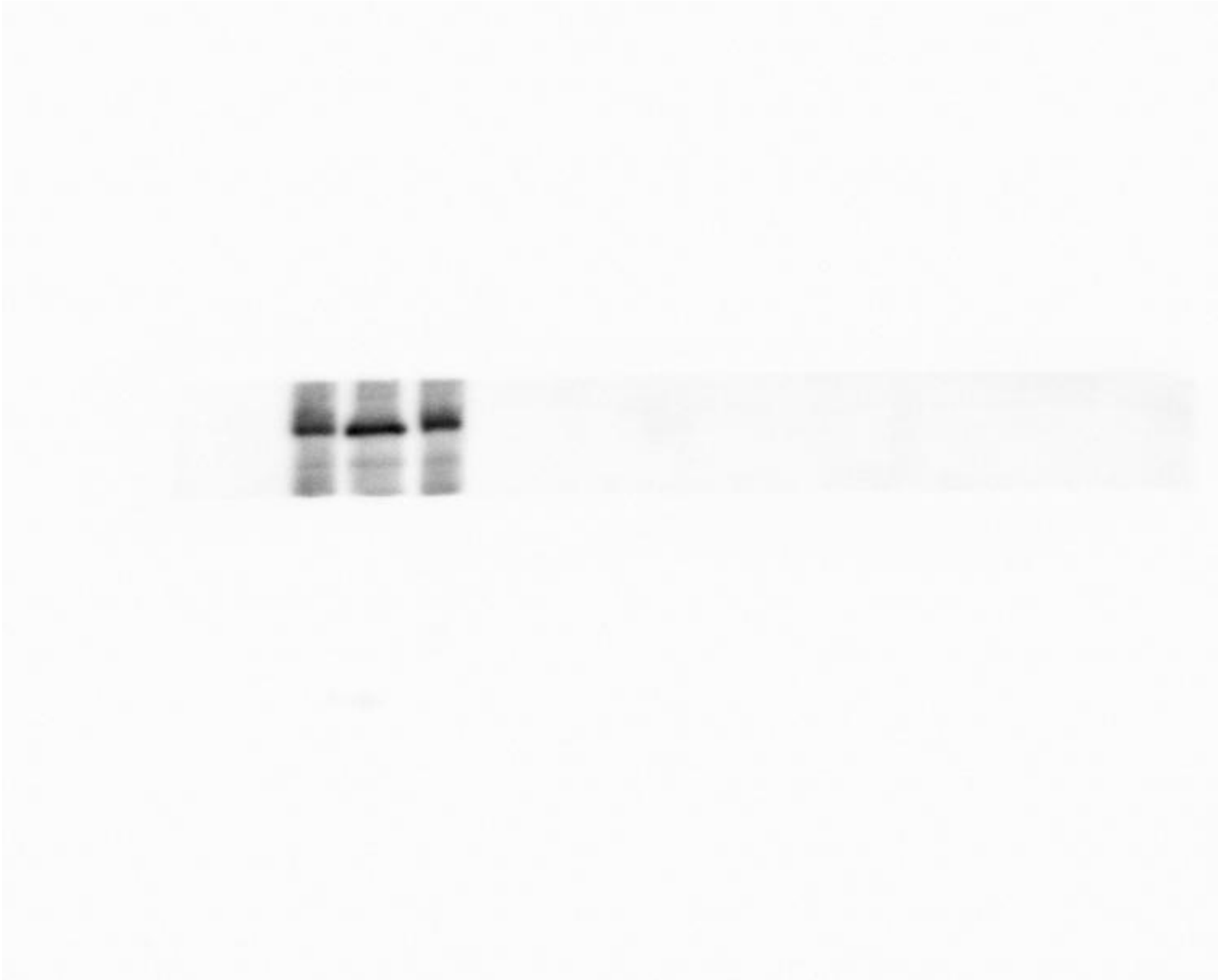

Fig9E

MED12

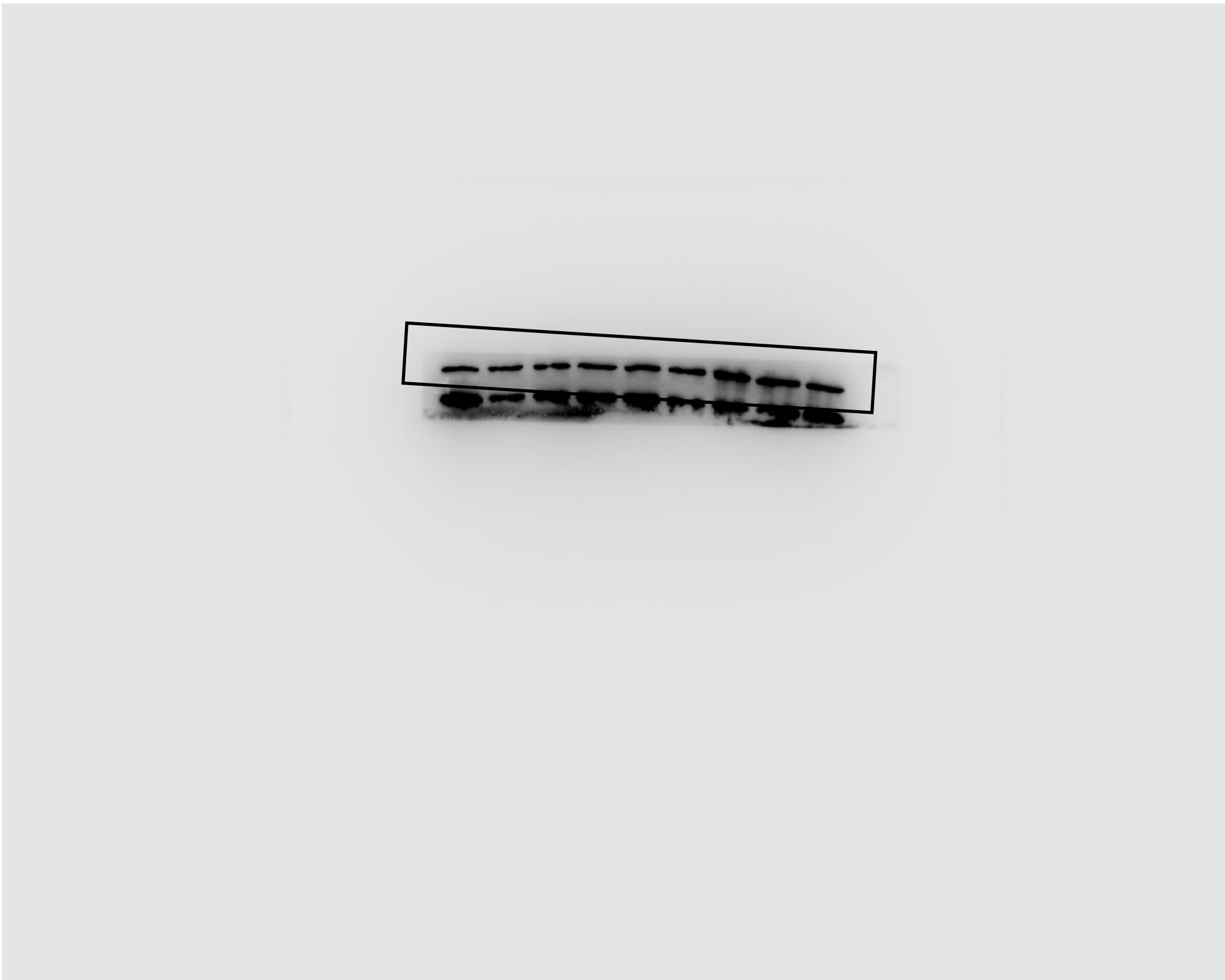

GAPDH

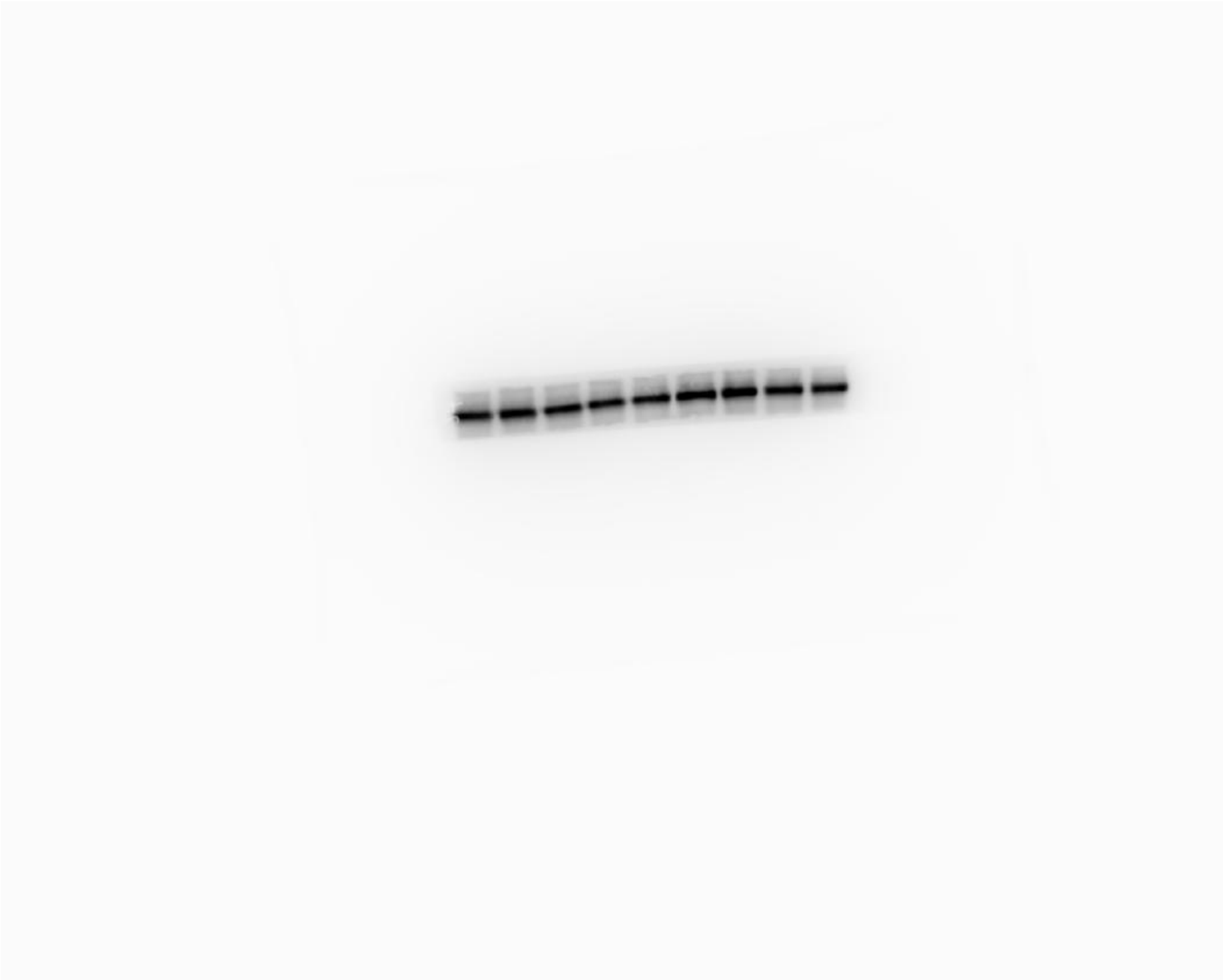

Fig9F

PP53

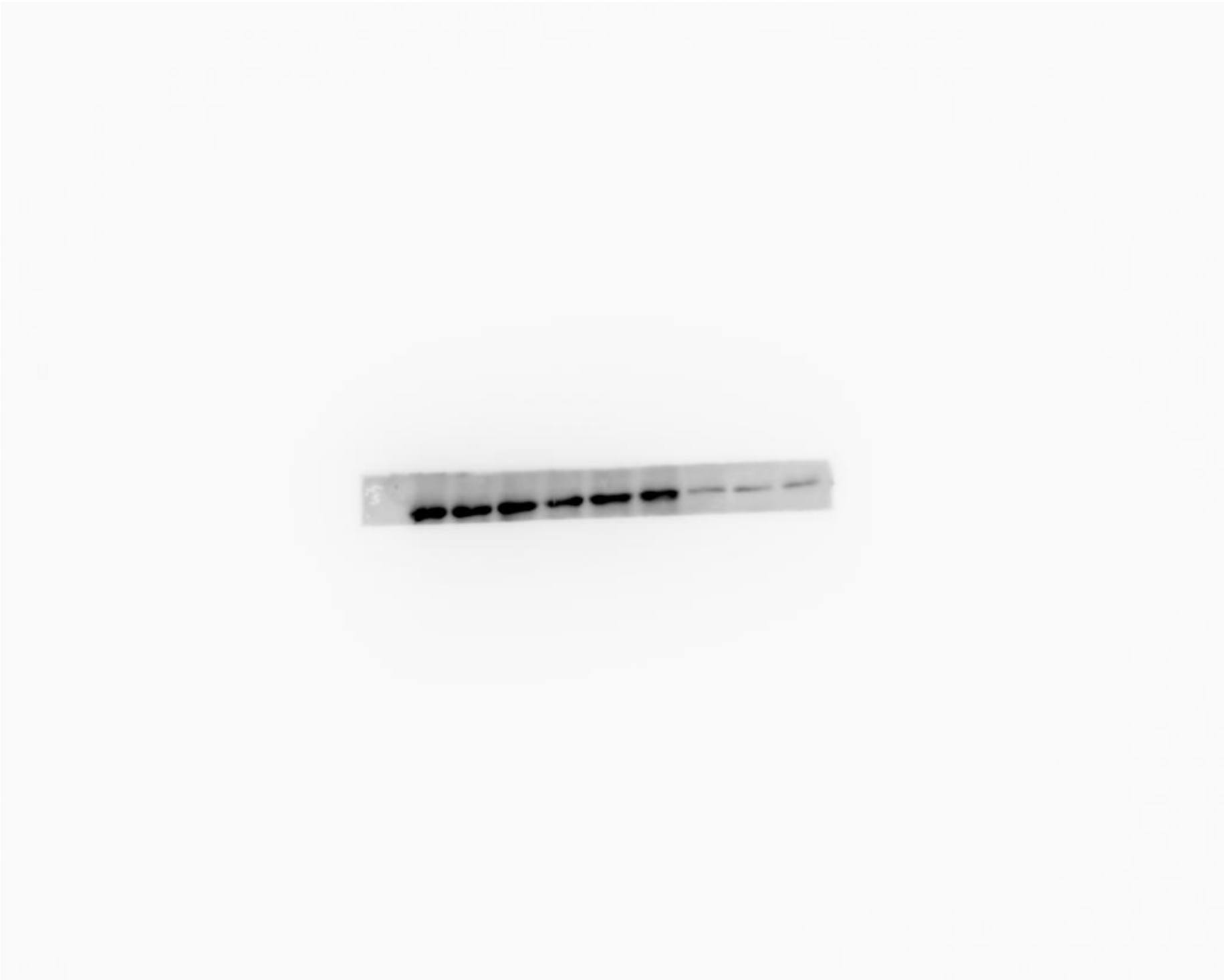

P53

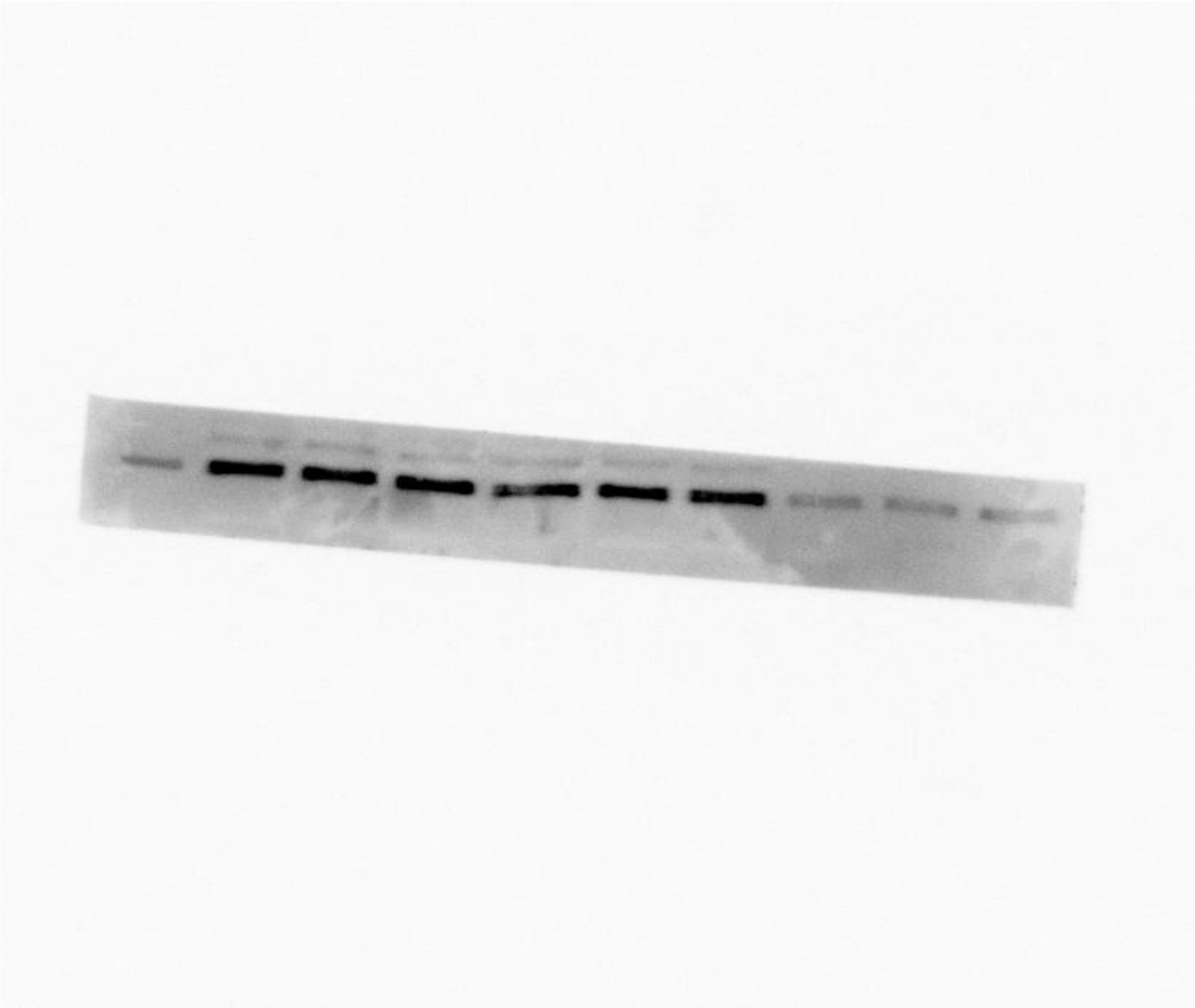

P21

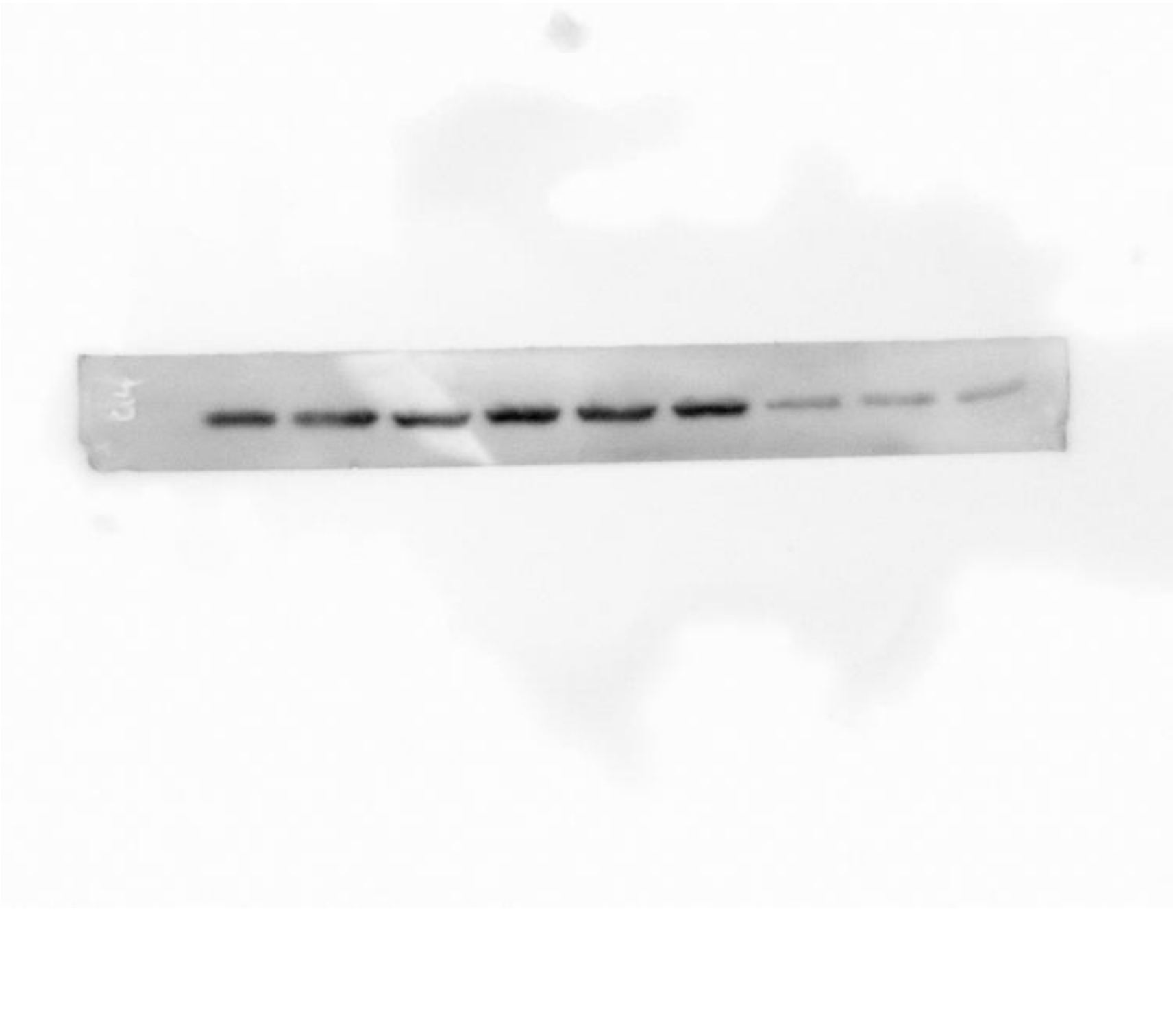

P16

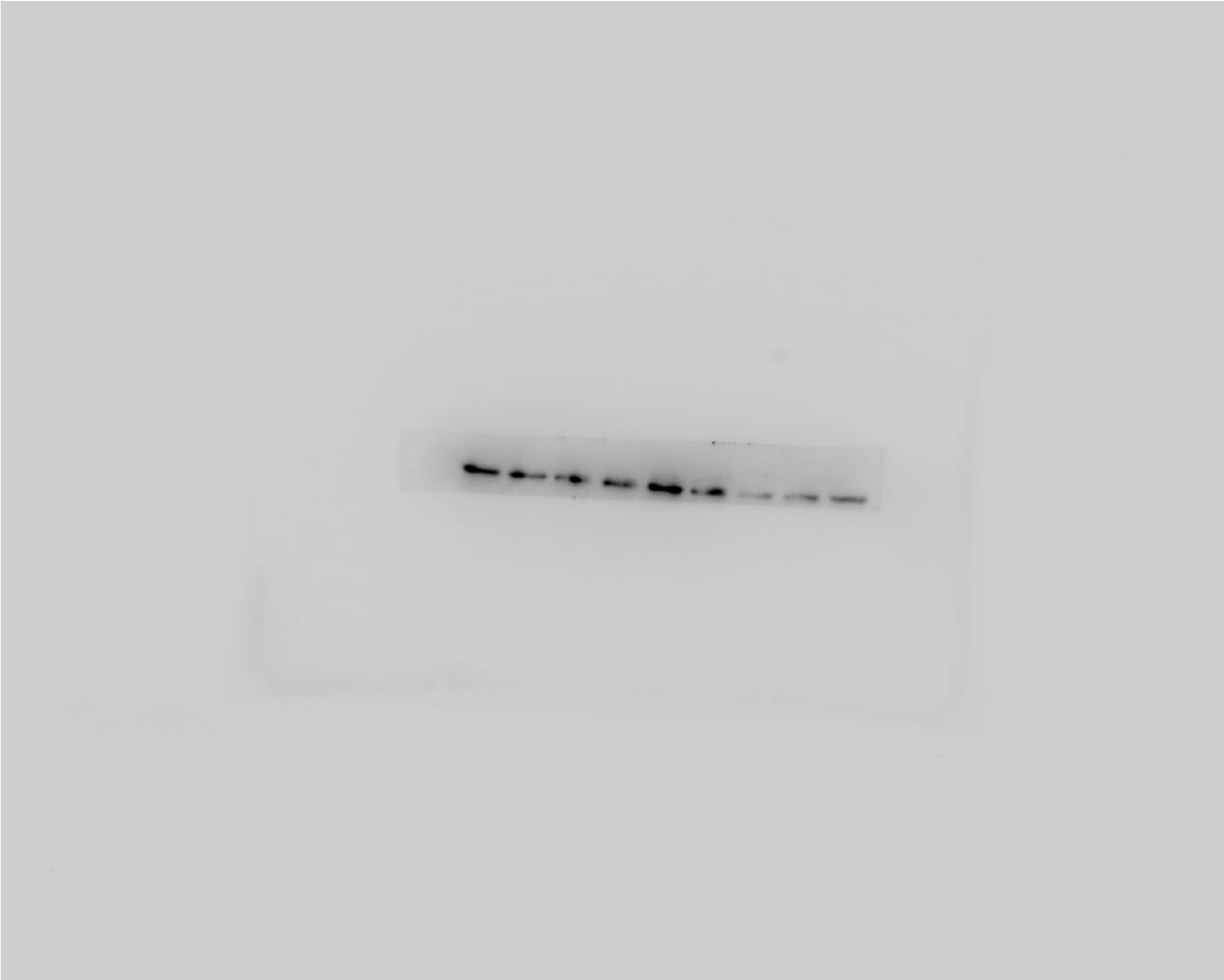

GAPDH

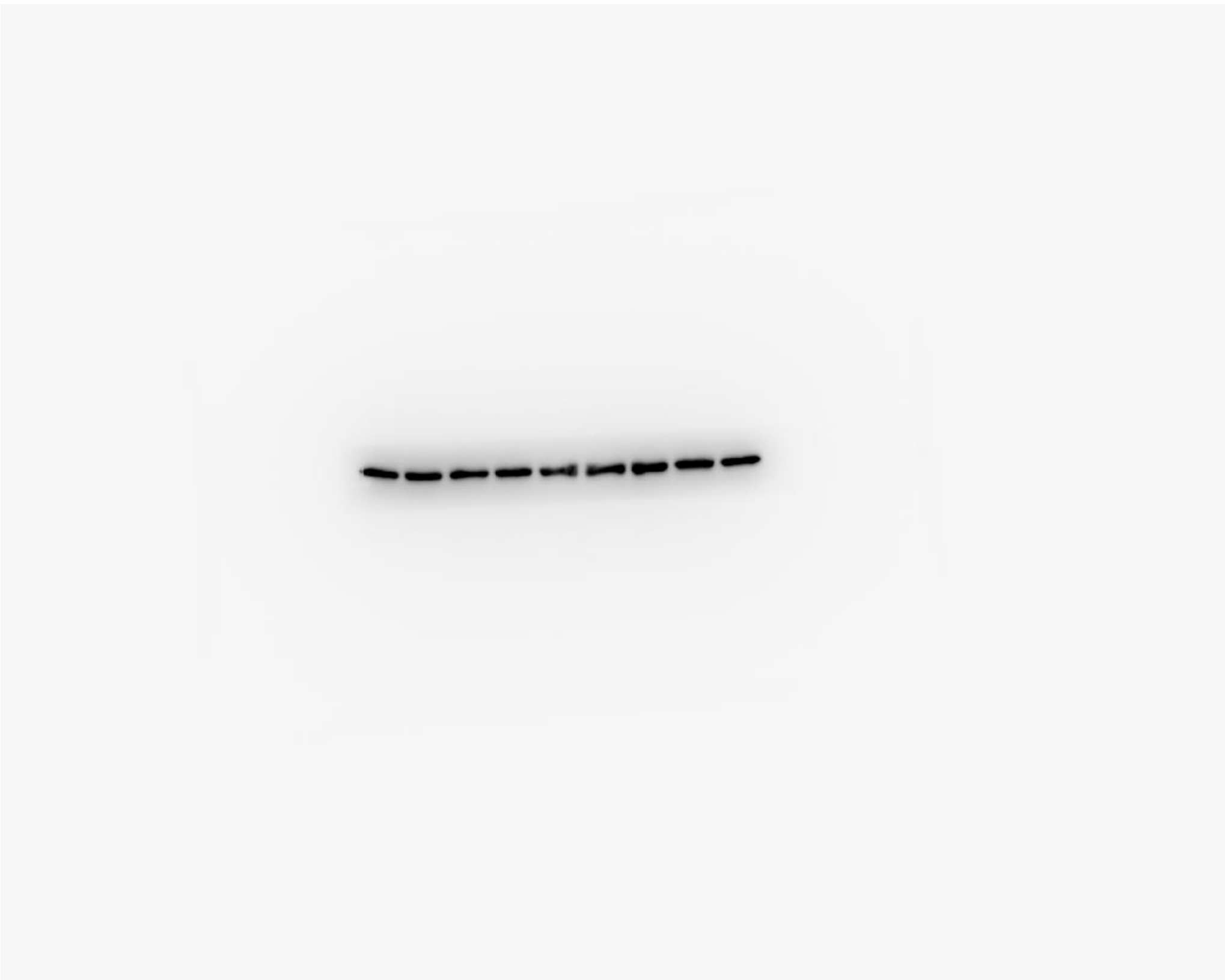

Fig9G

p-TBK1

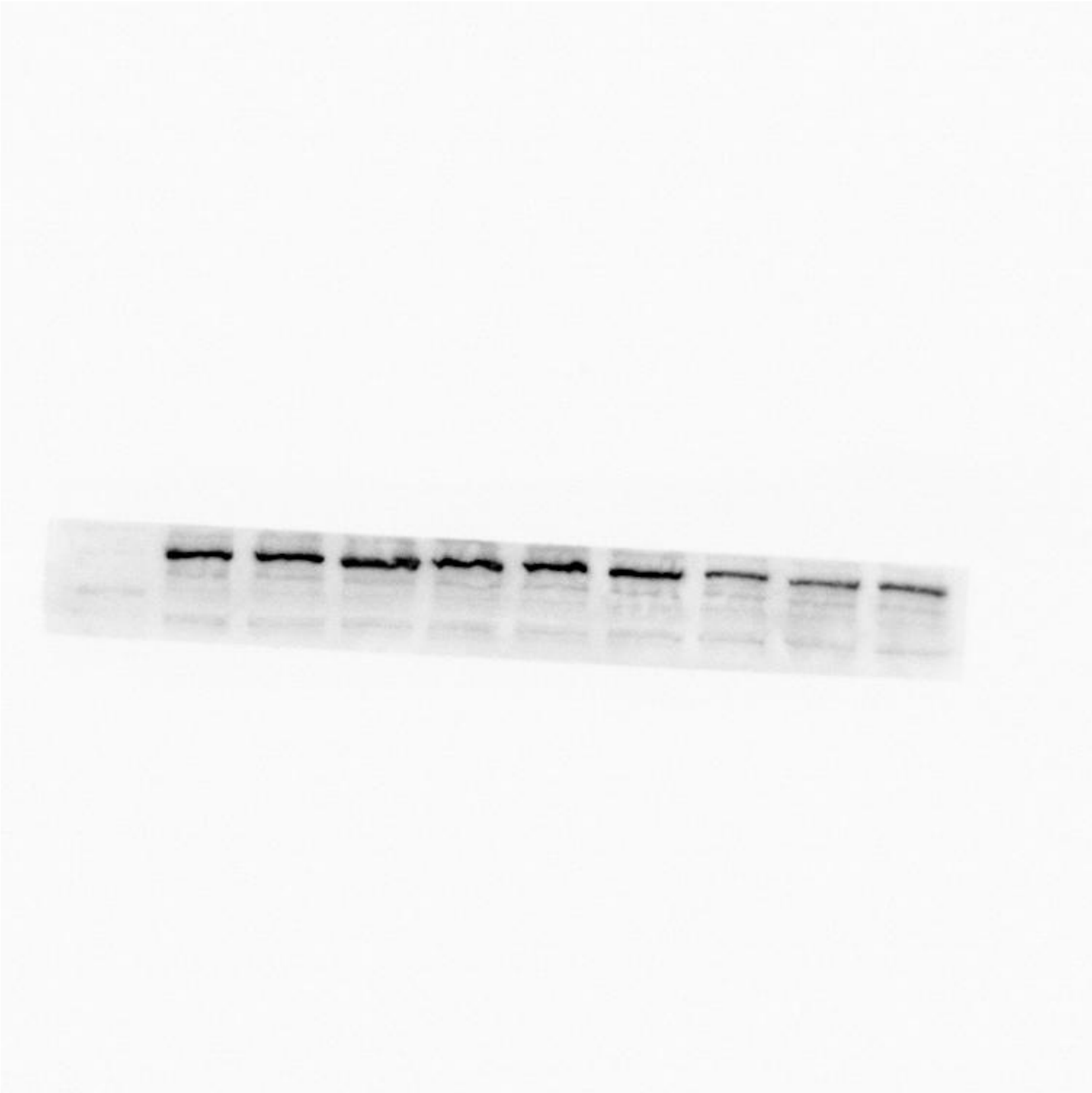

TBK1

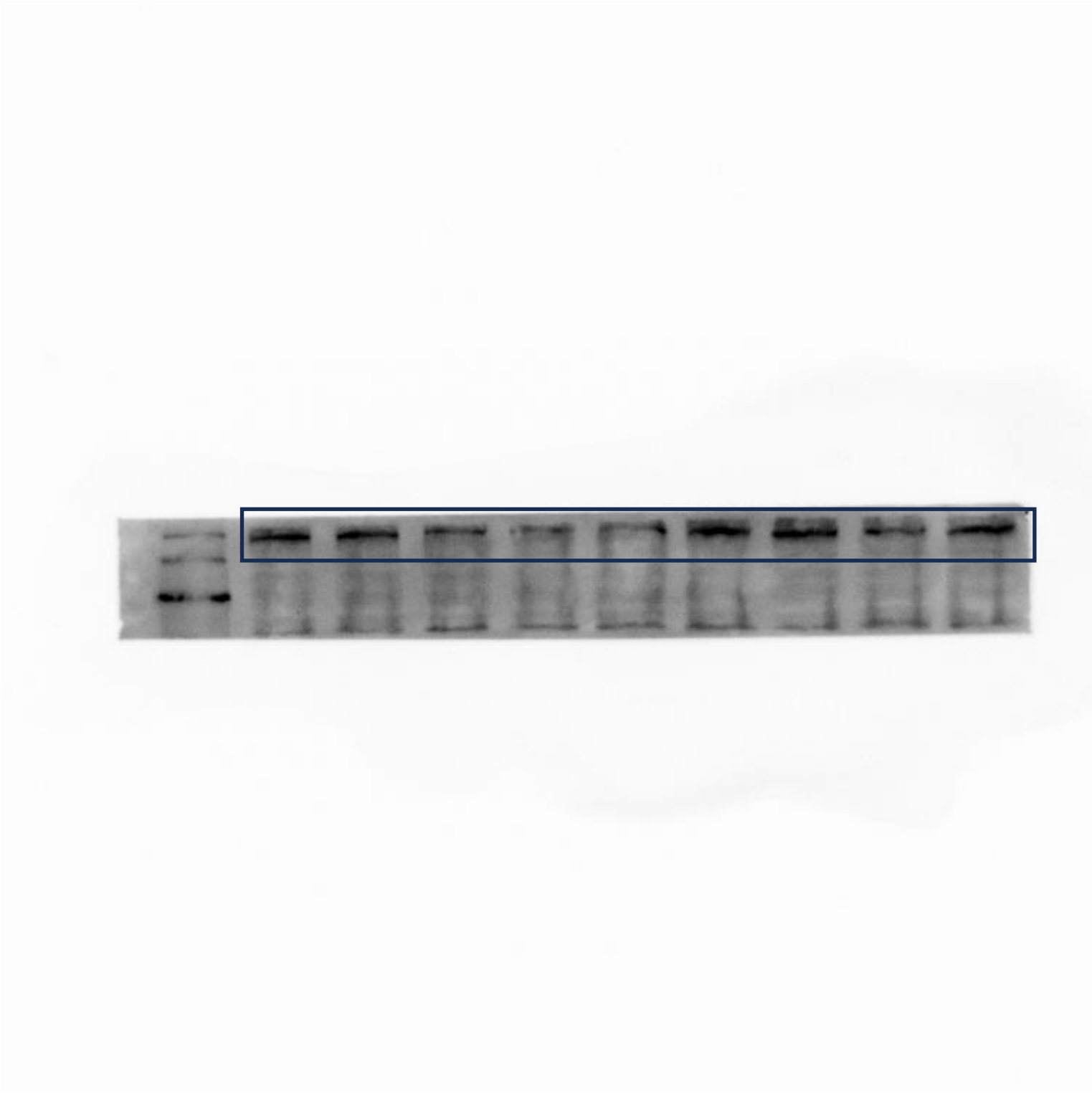

p-IRF3

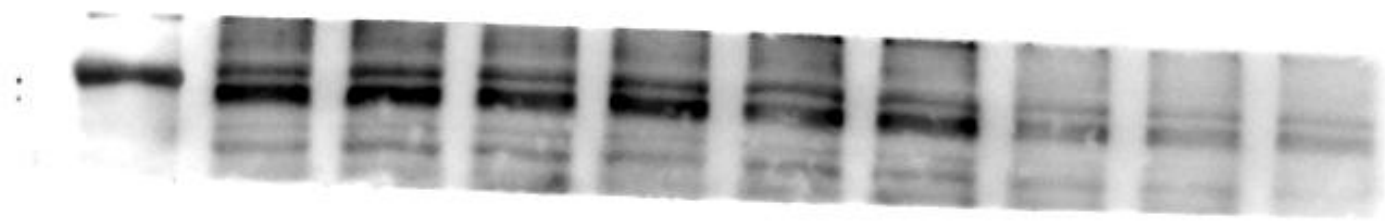

IRF3

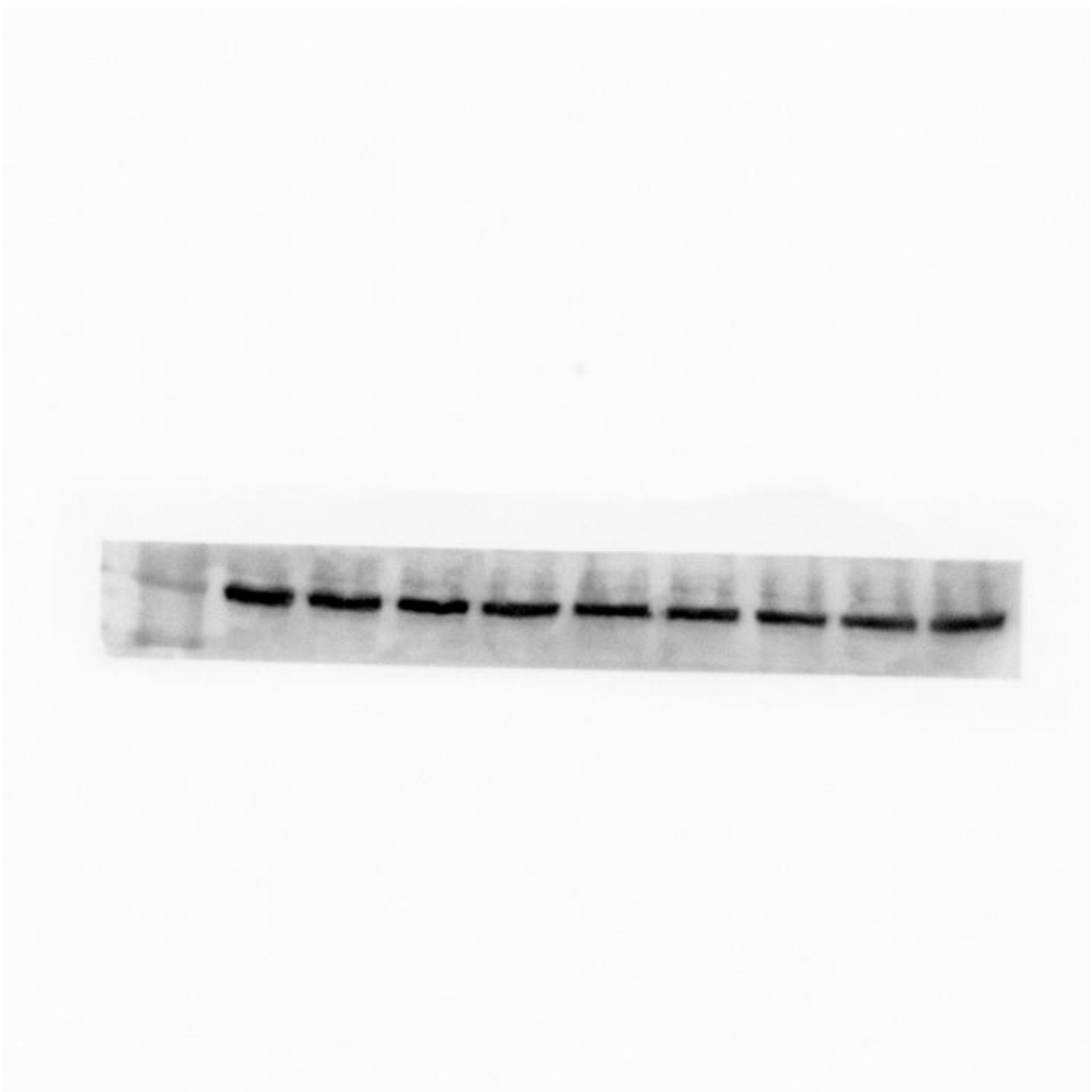

STING

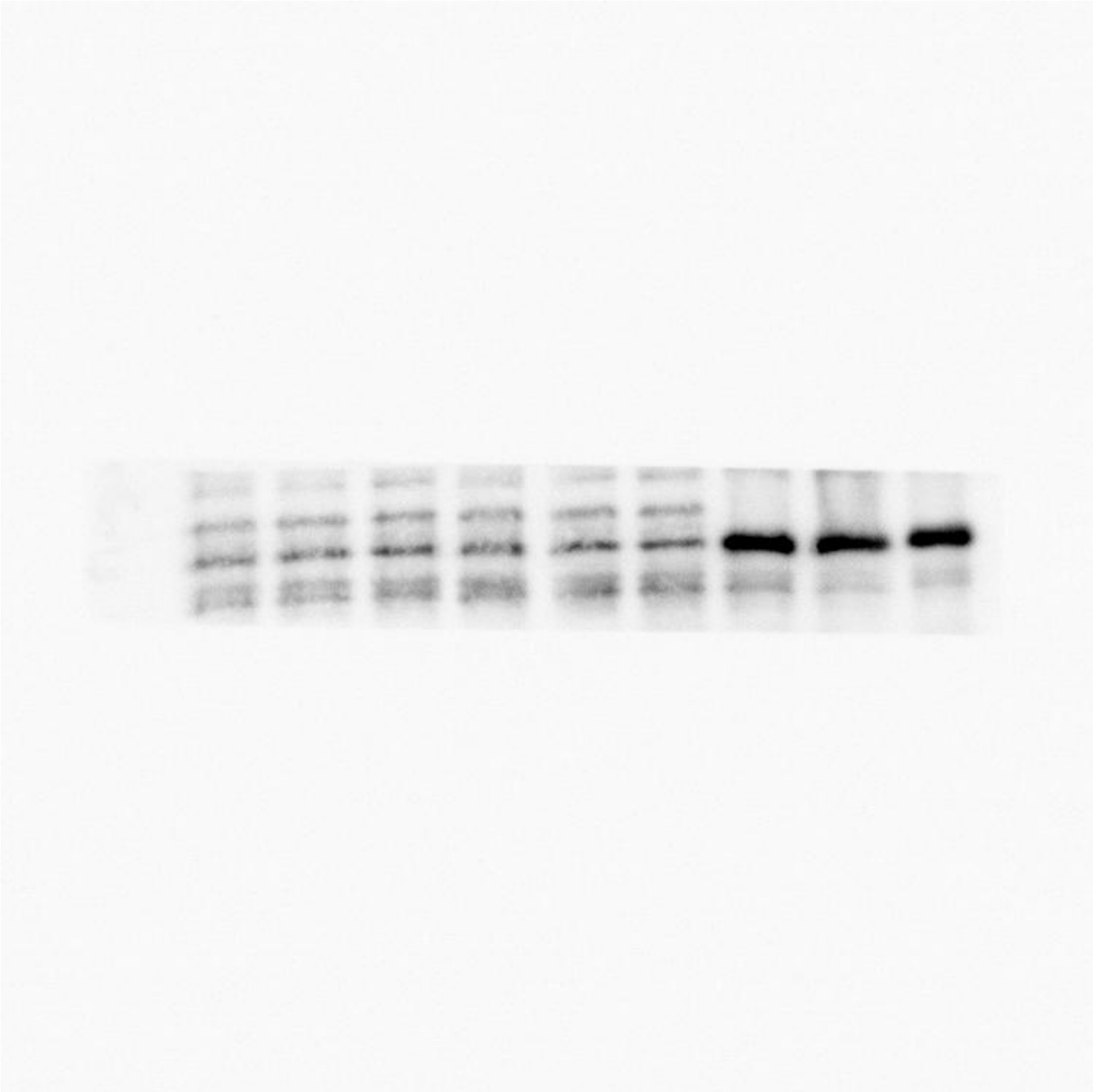

GAPDH

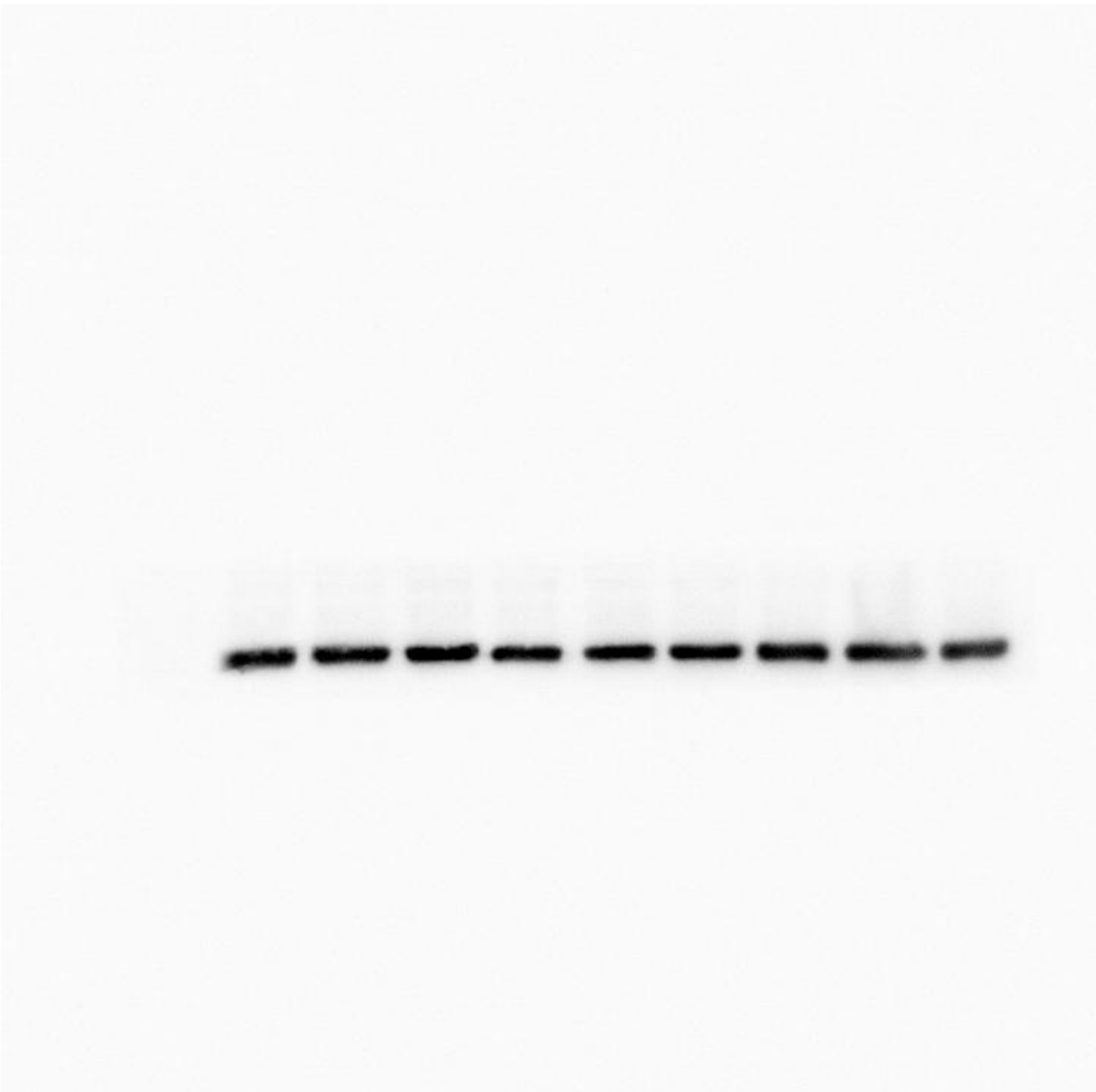

FigS1B

MMA-IP

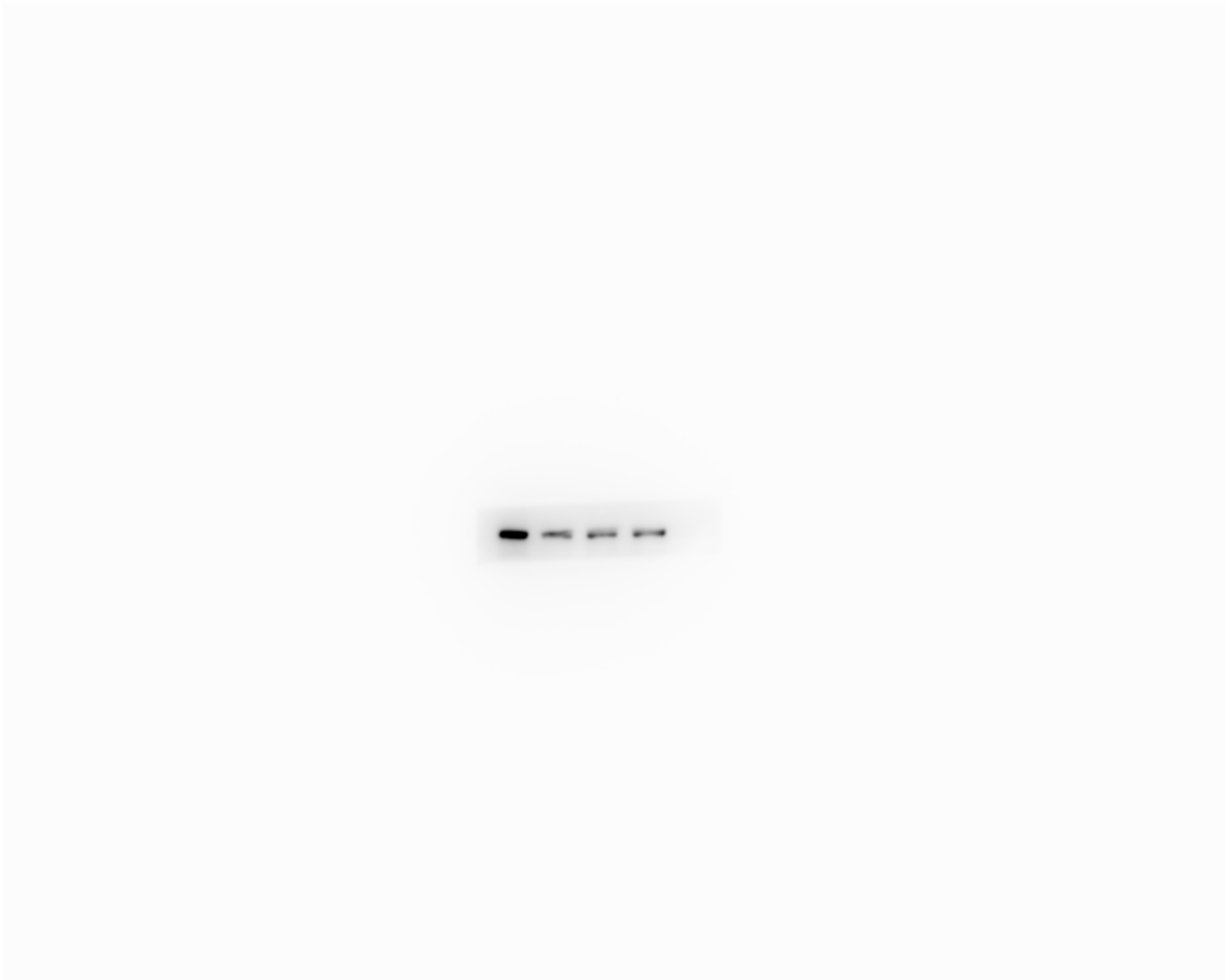

GFP-IP

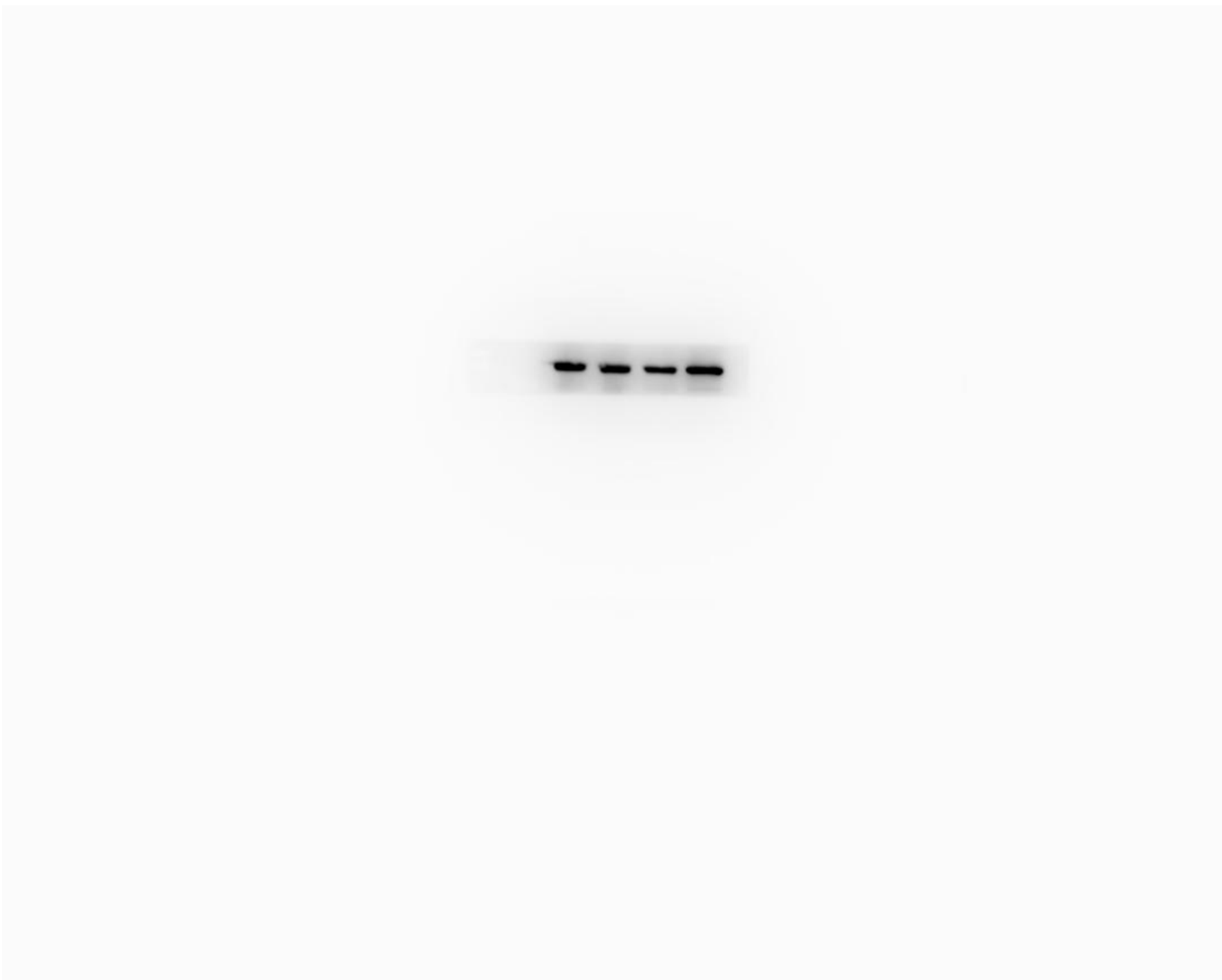

GFP-Input

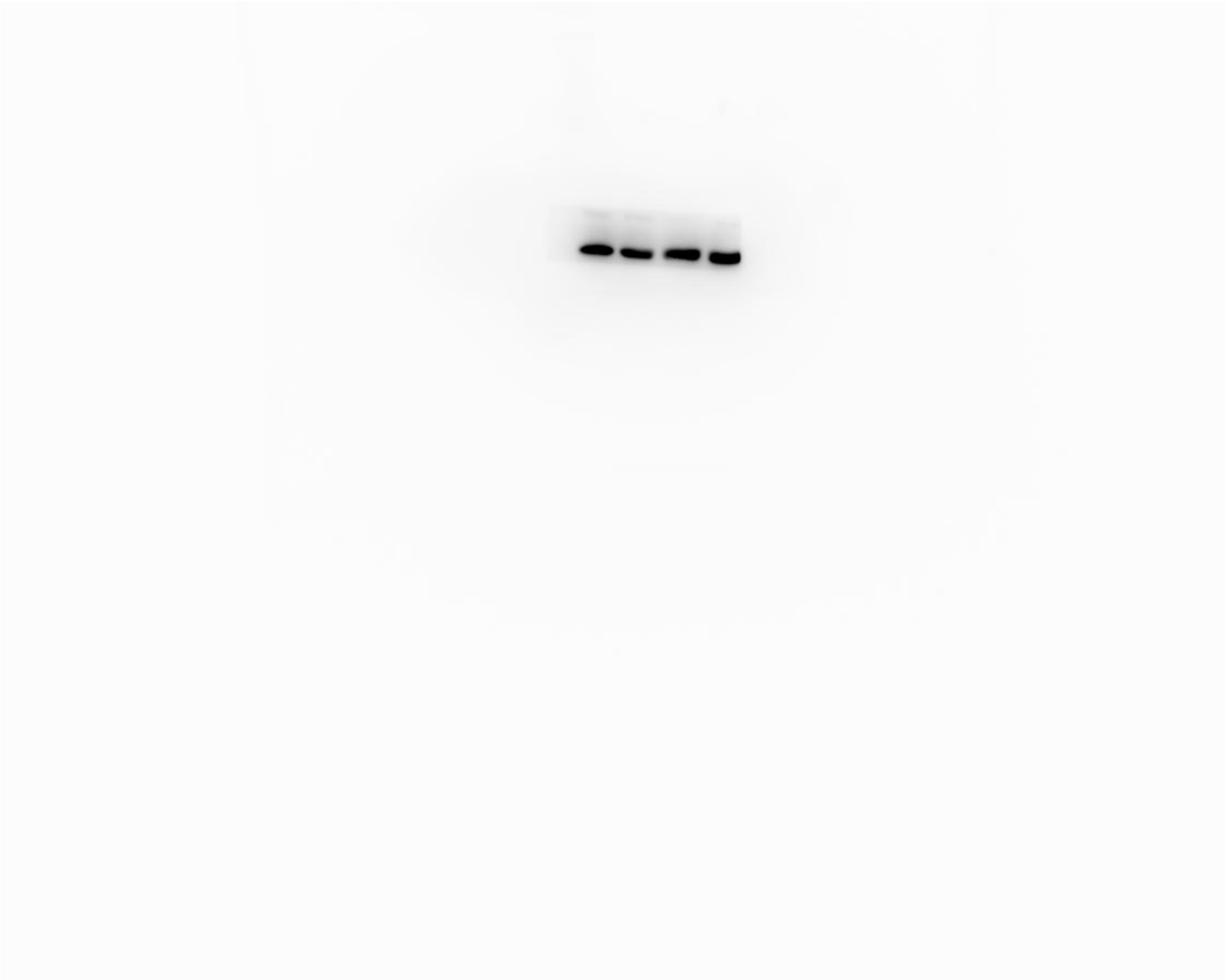

ACTIN

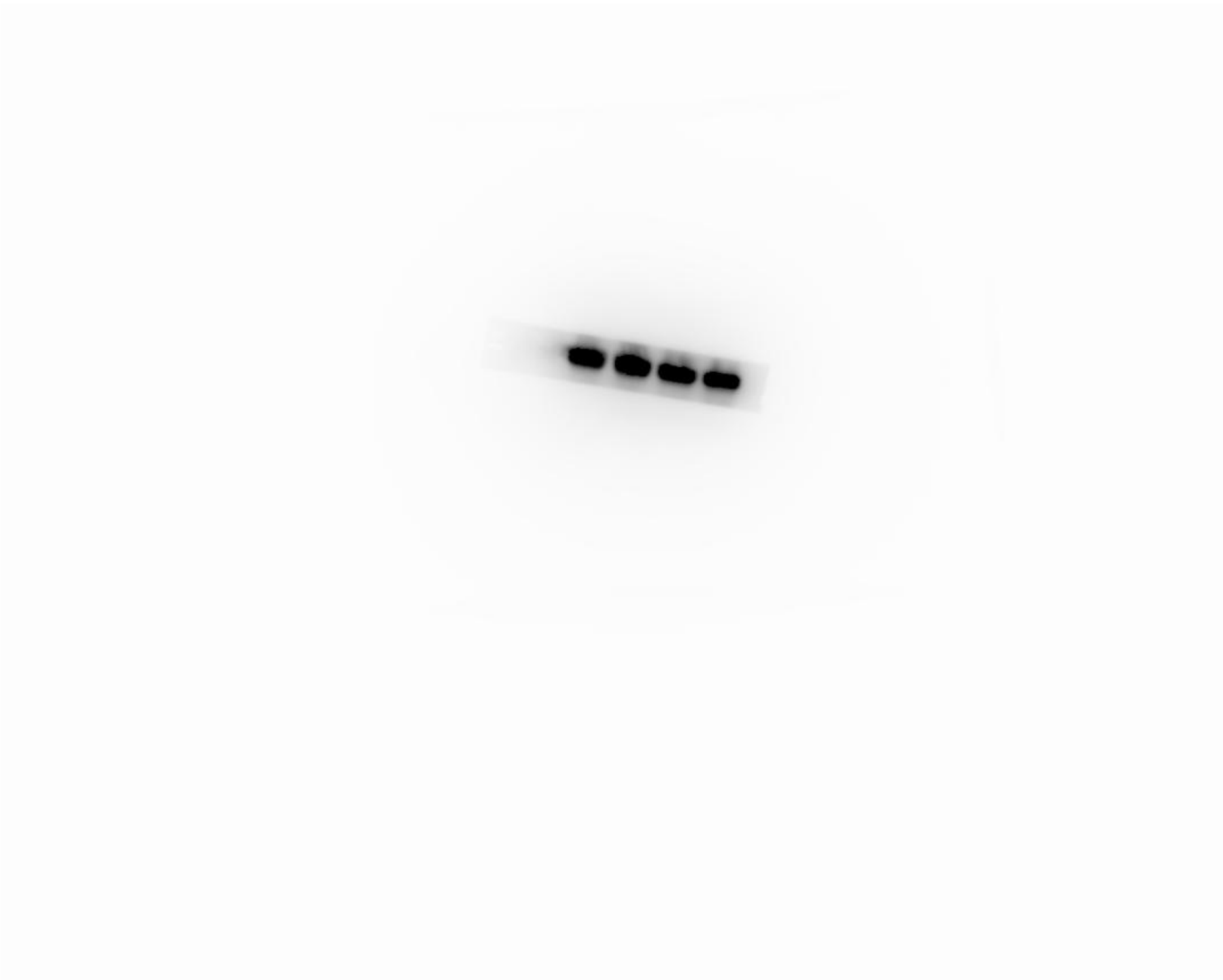

FigS1D

Human

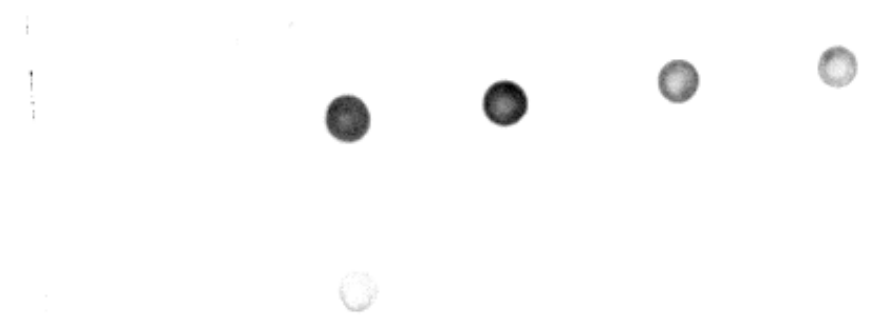

Rat

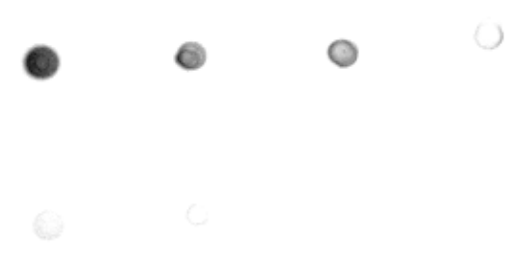

FigS1E

FBXO7me

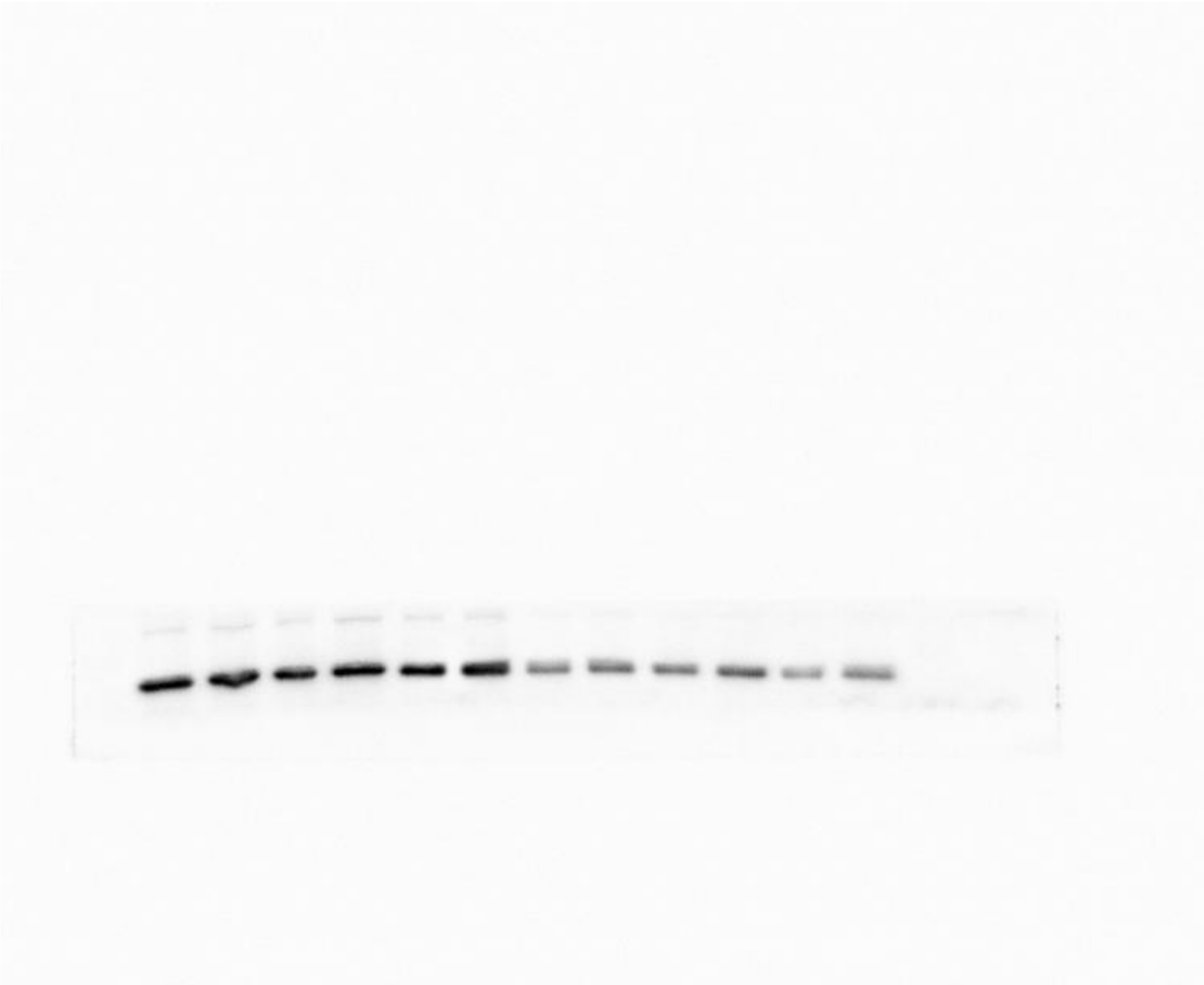

GAPDH

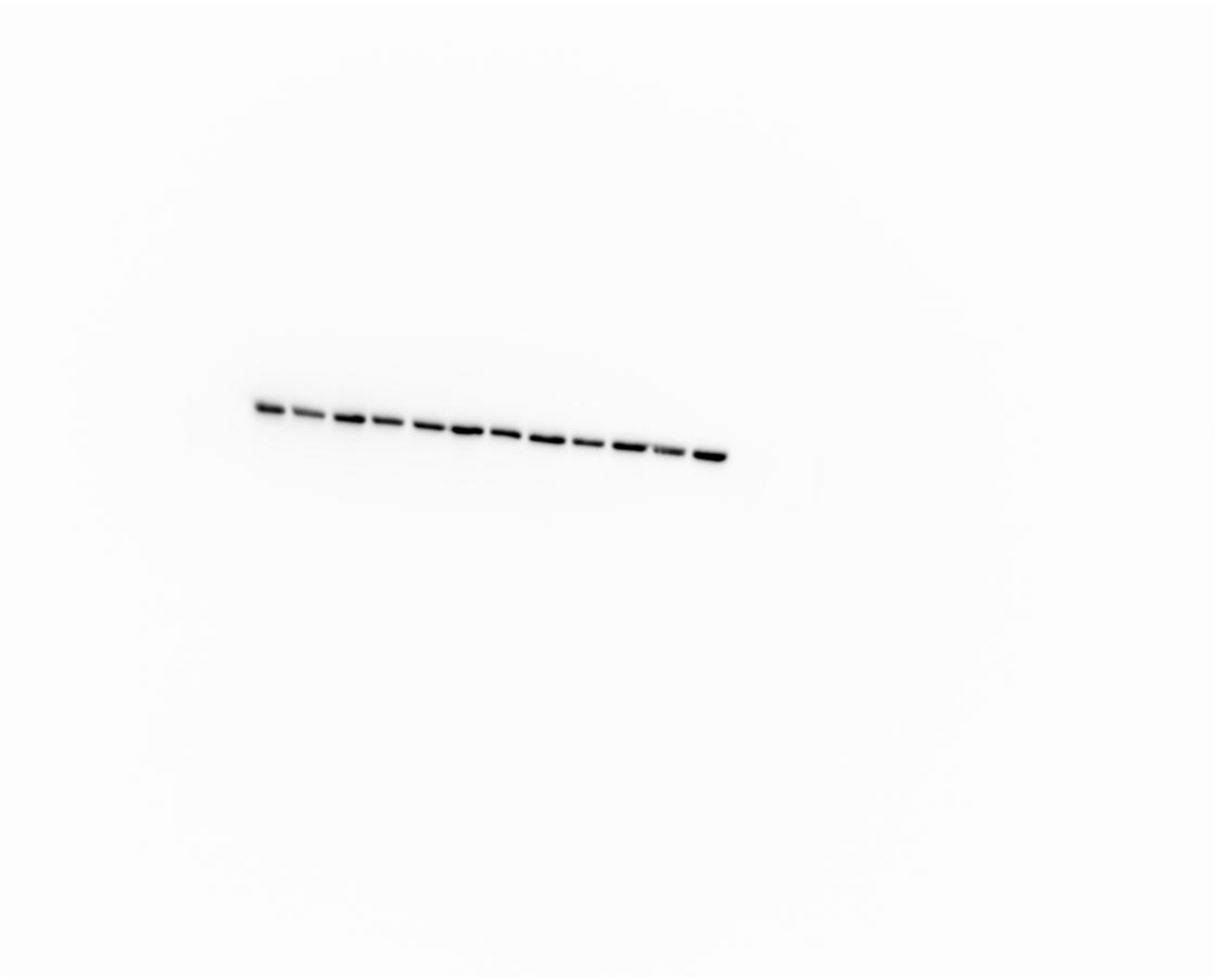

FigS2A

P53

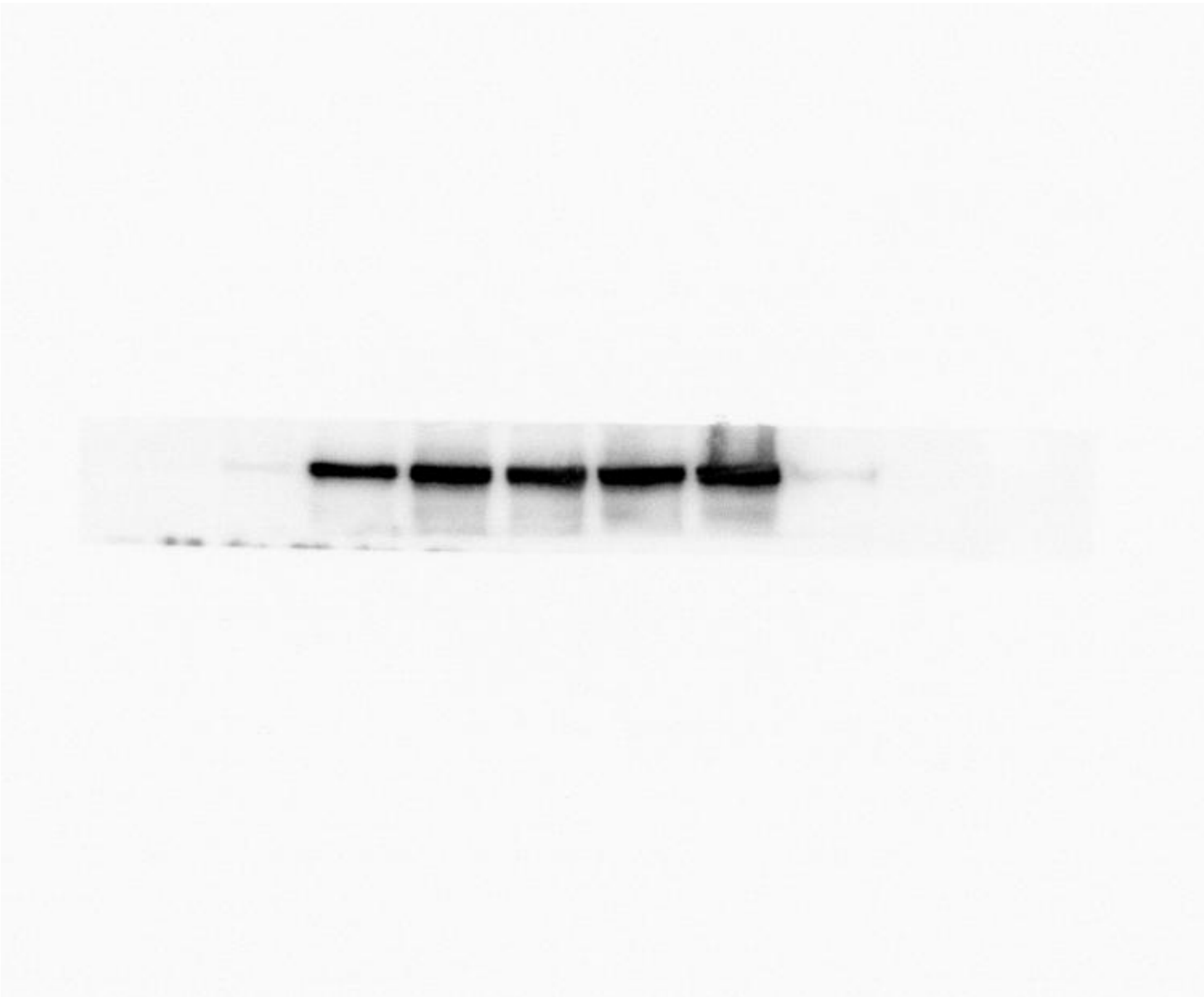

P21

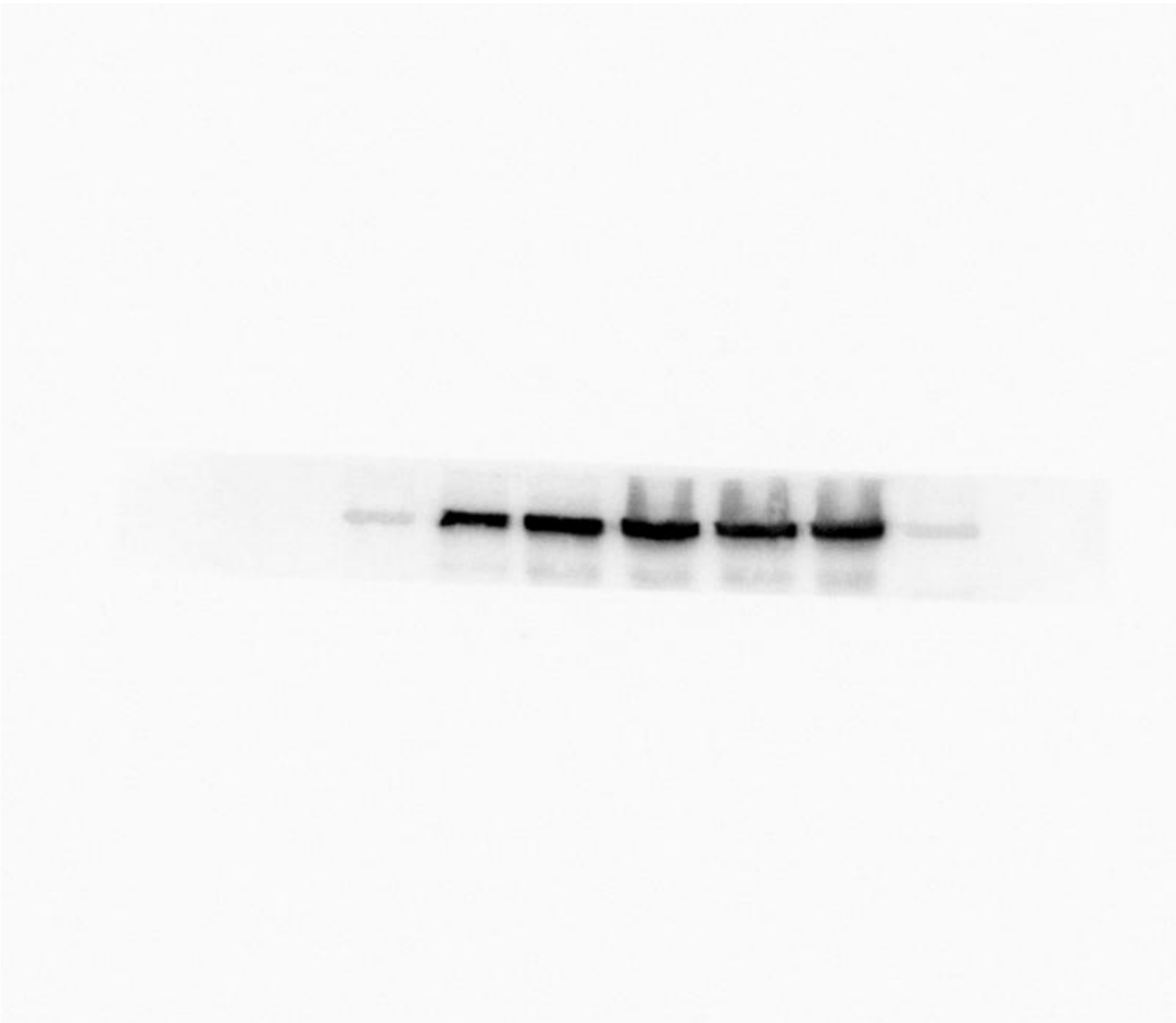

P16

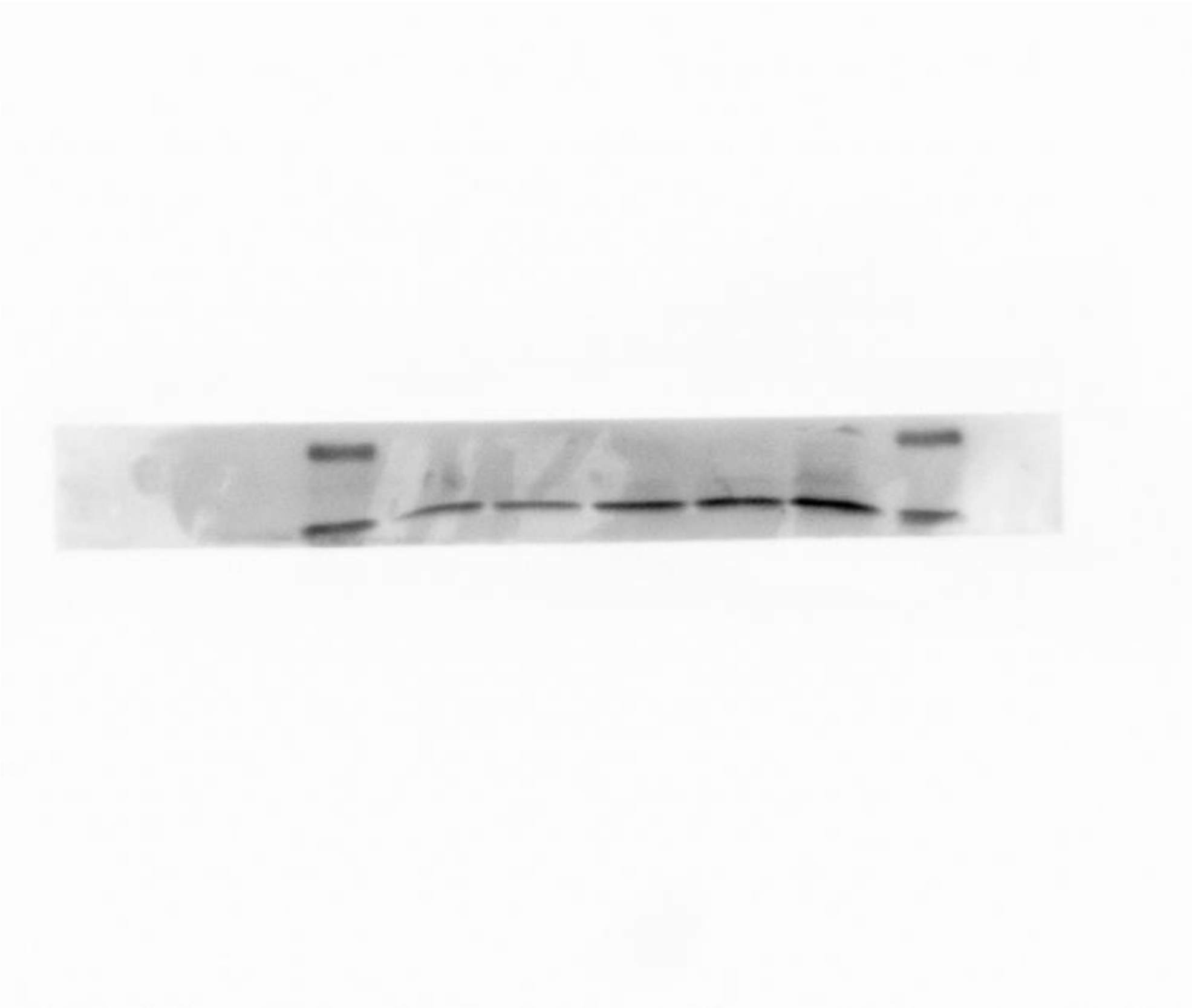

Tubulin

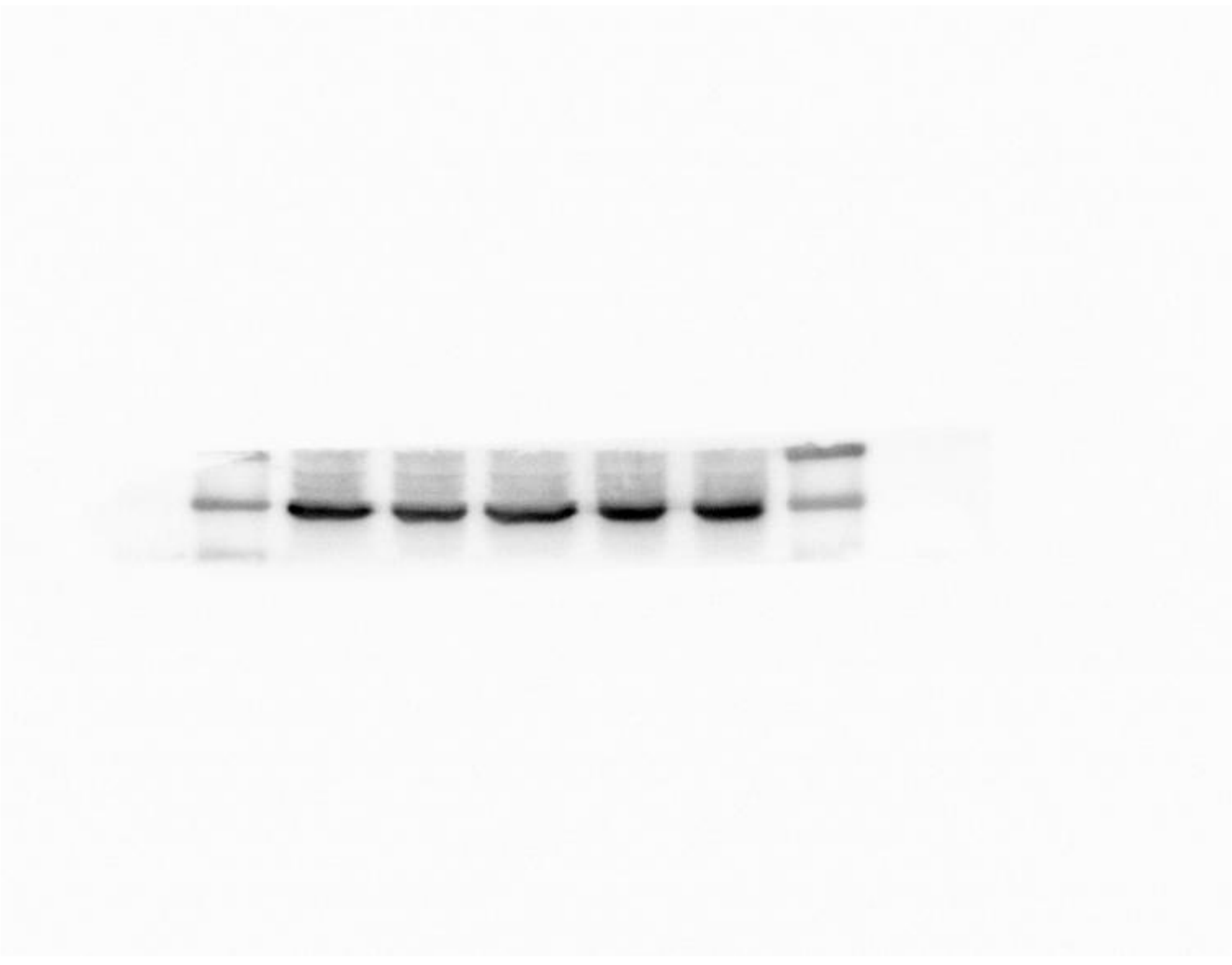

FigS2H

PP53

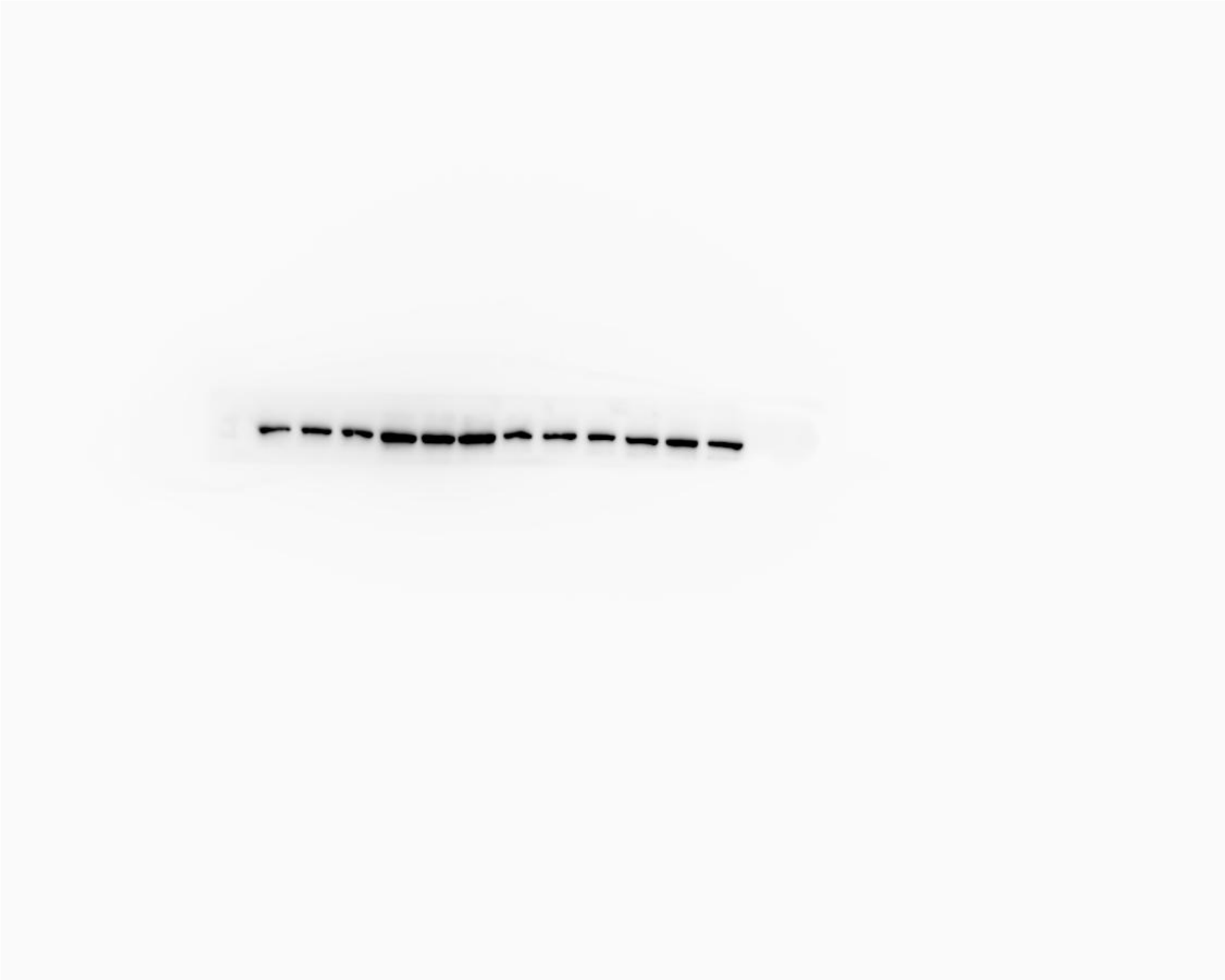

P53

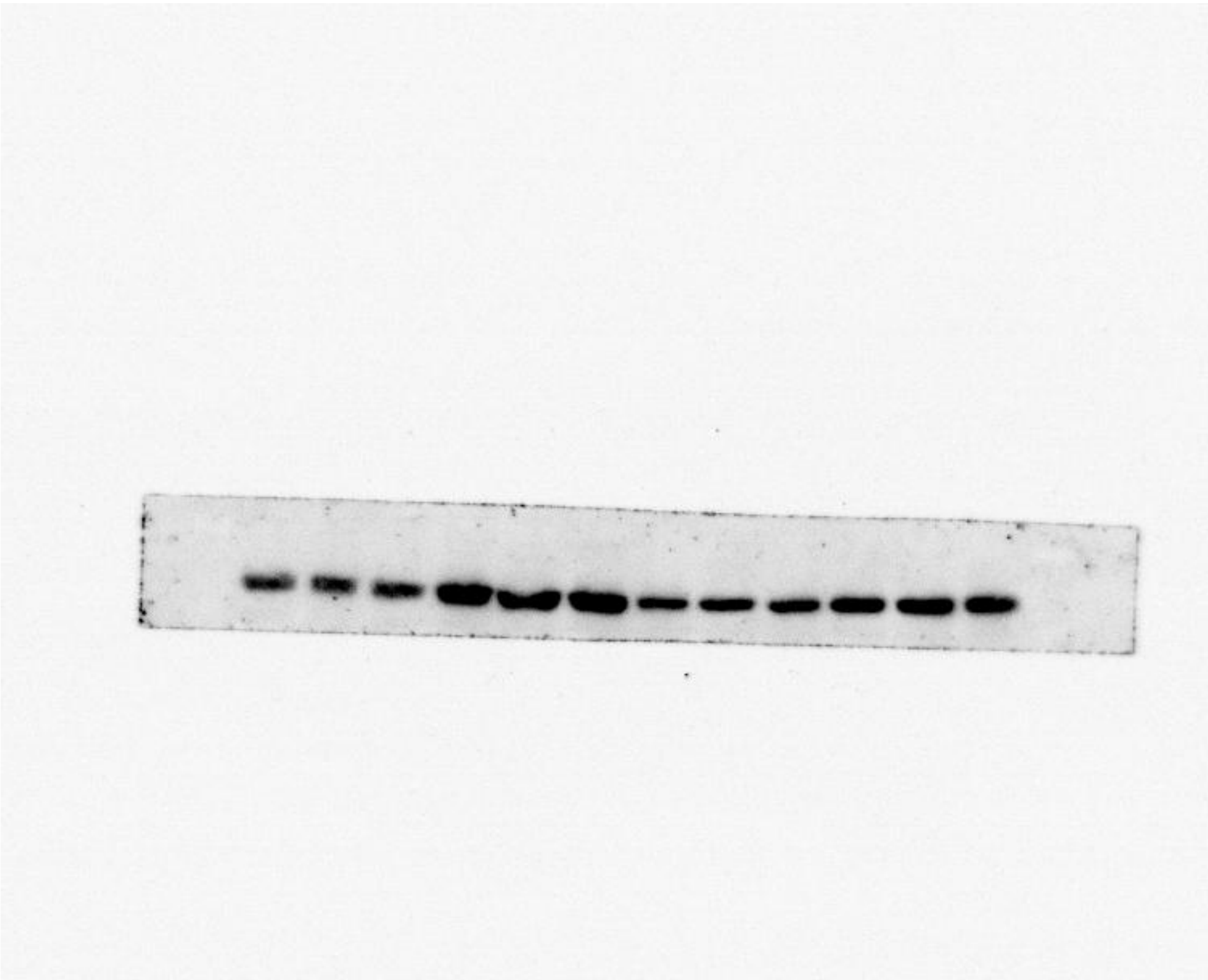

P21

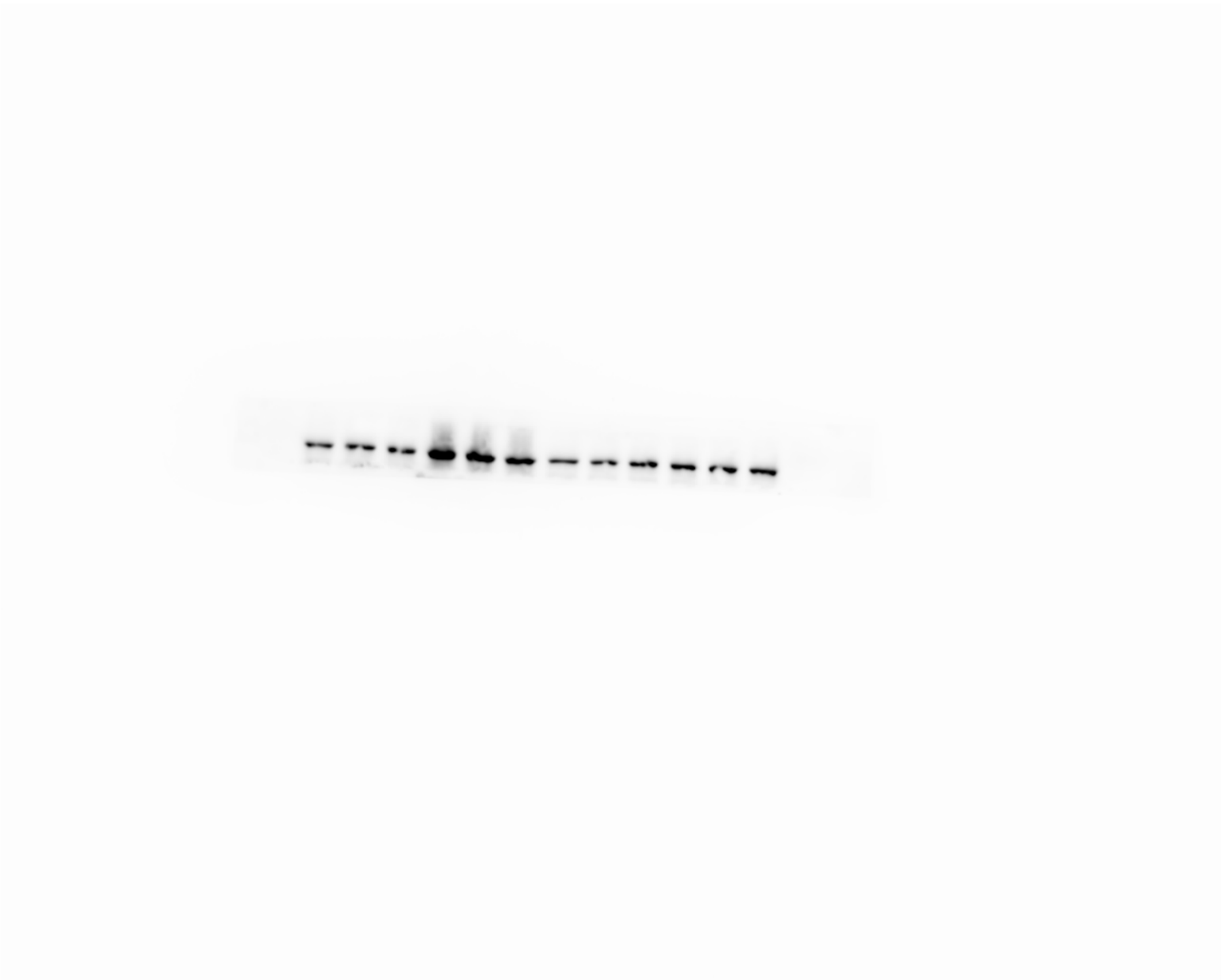

P16

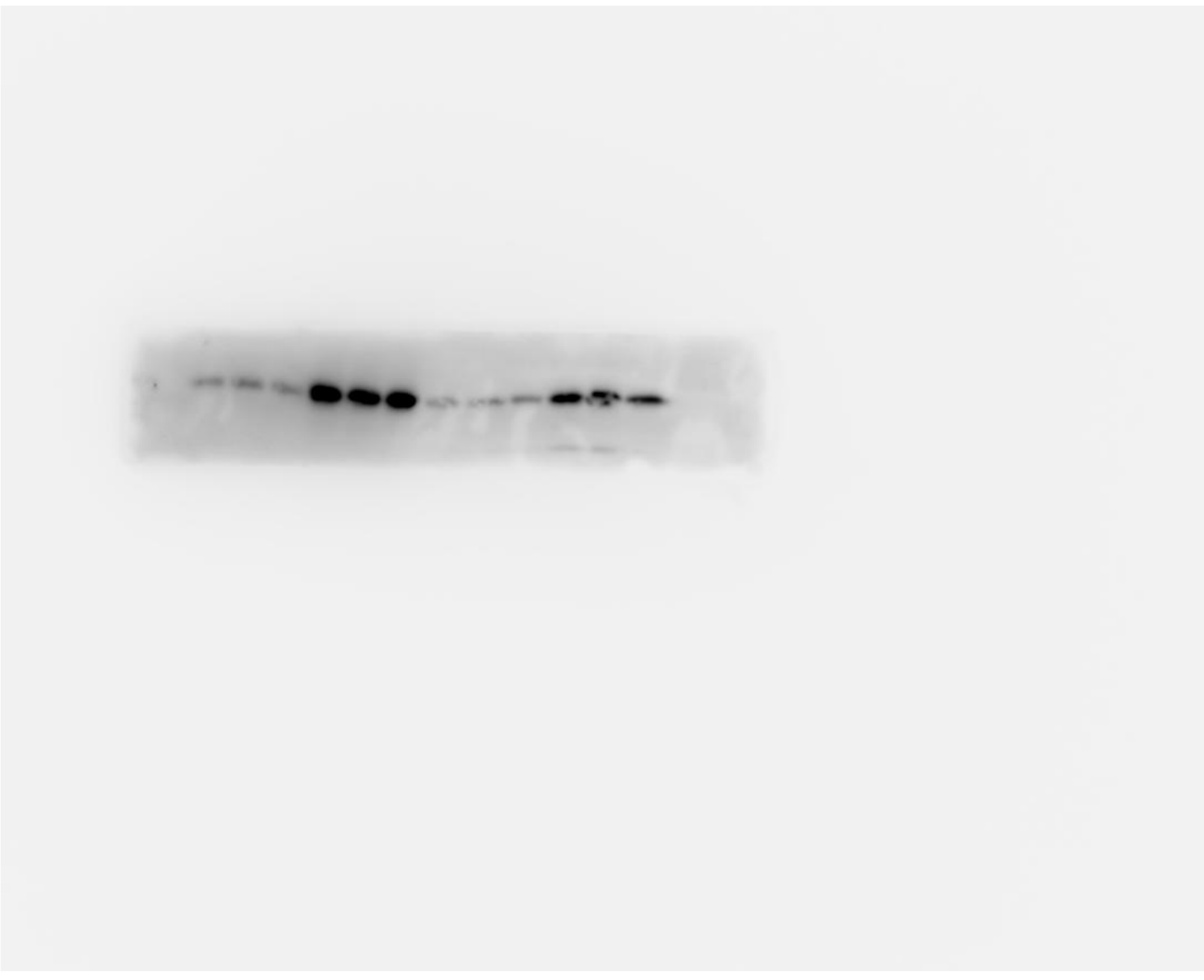

GAPDH

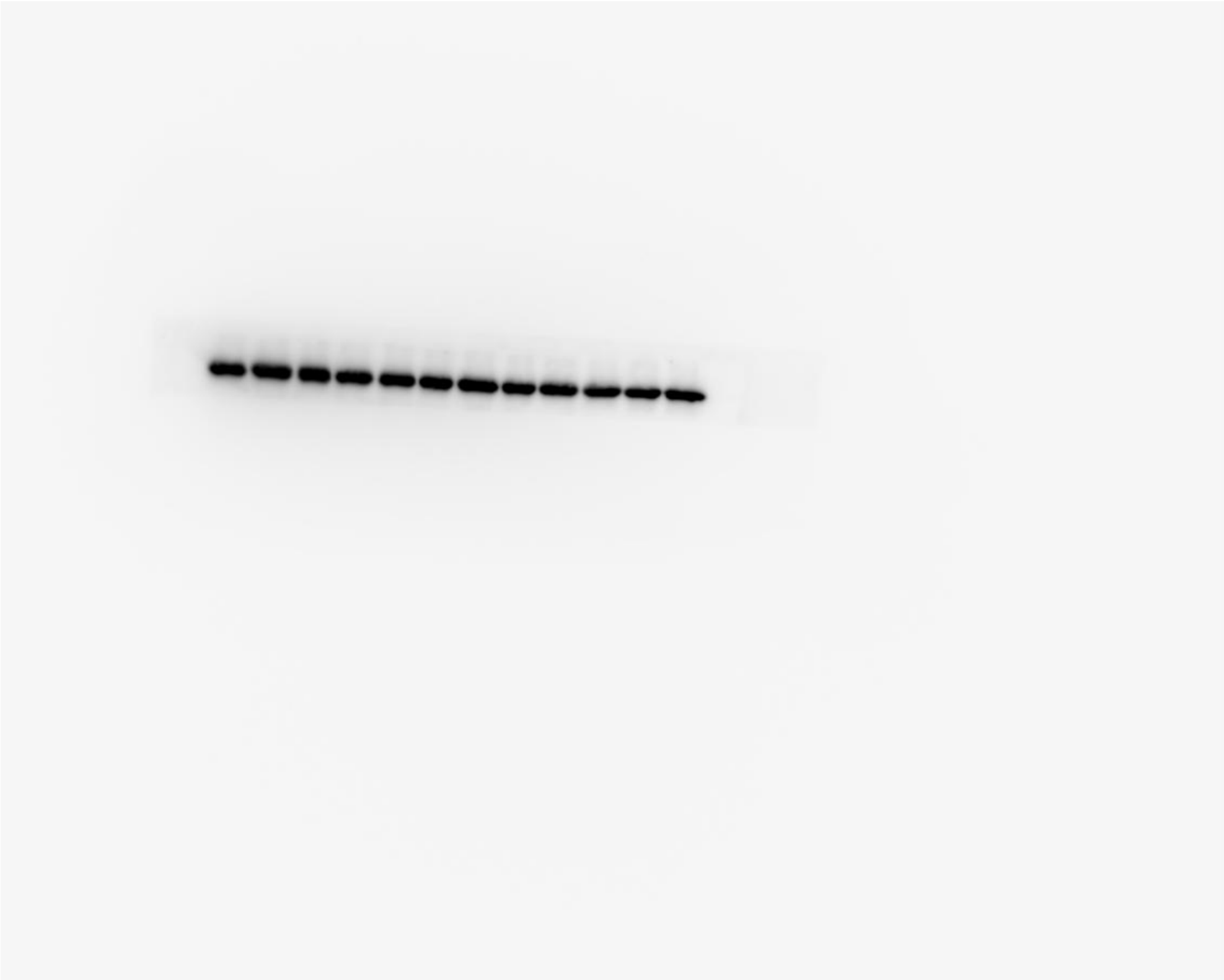

FigS3A

HA-IP

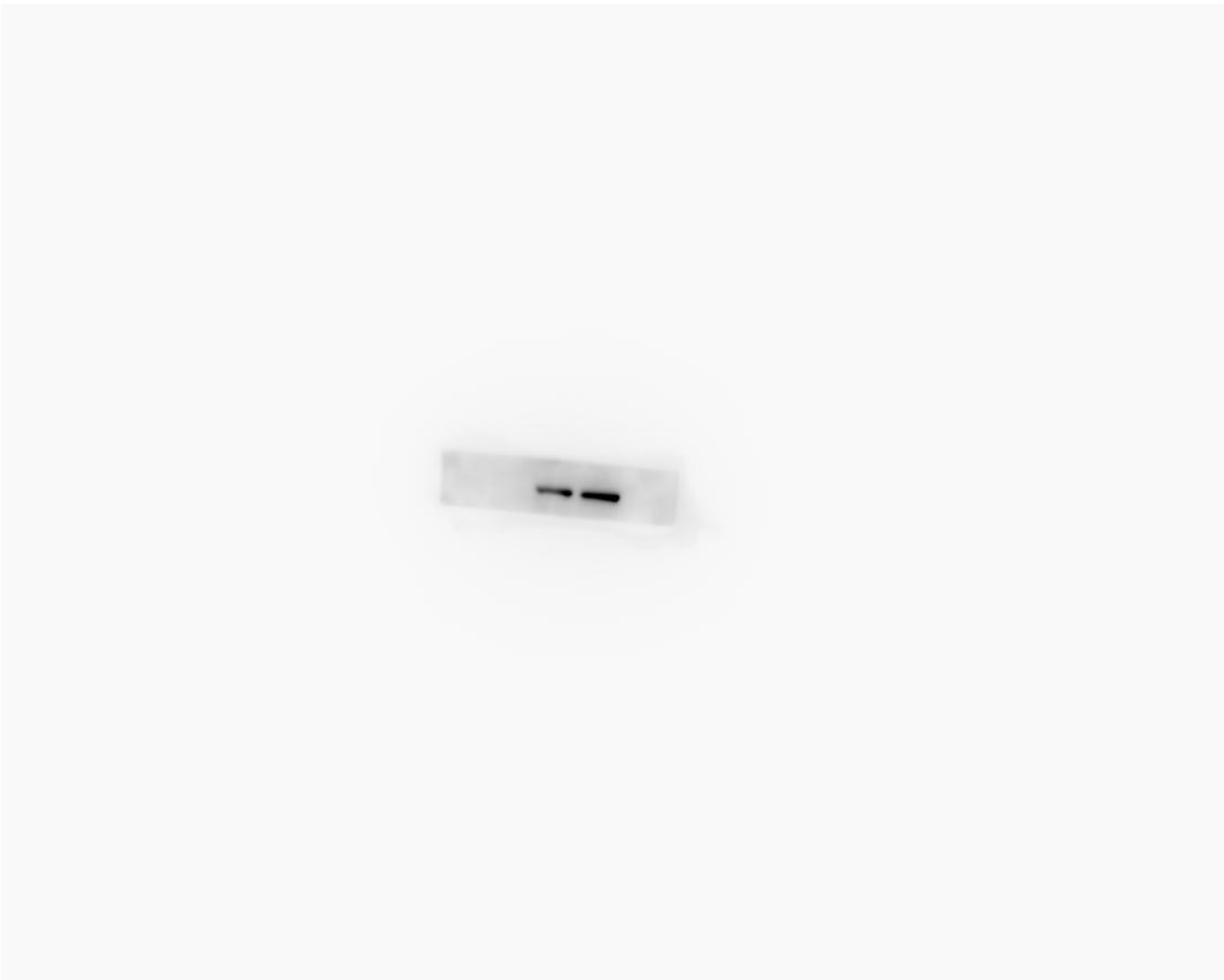

GFP-IP

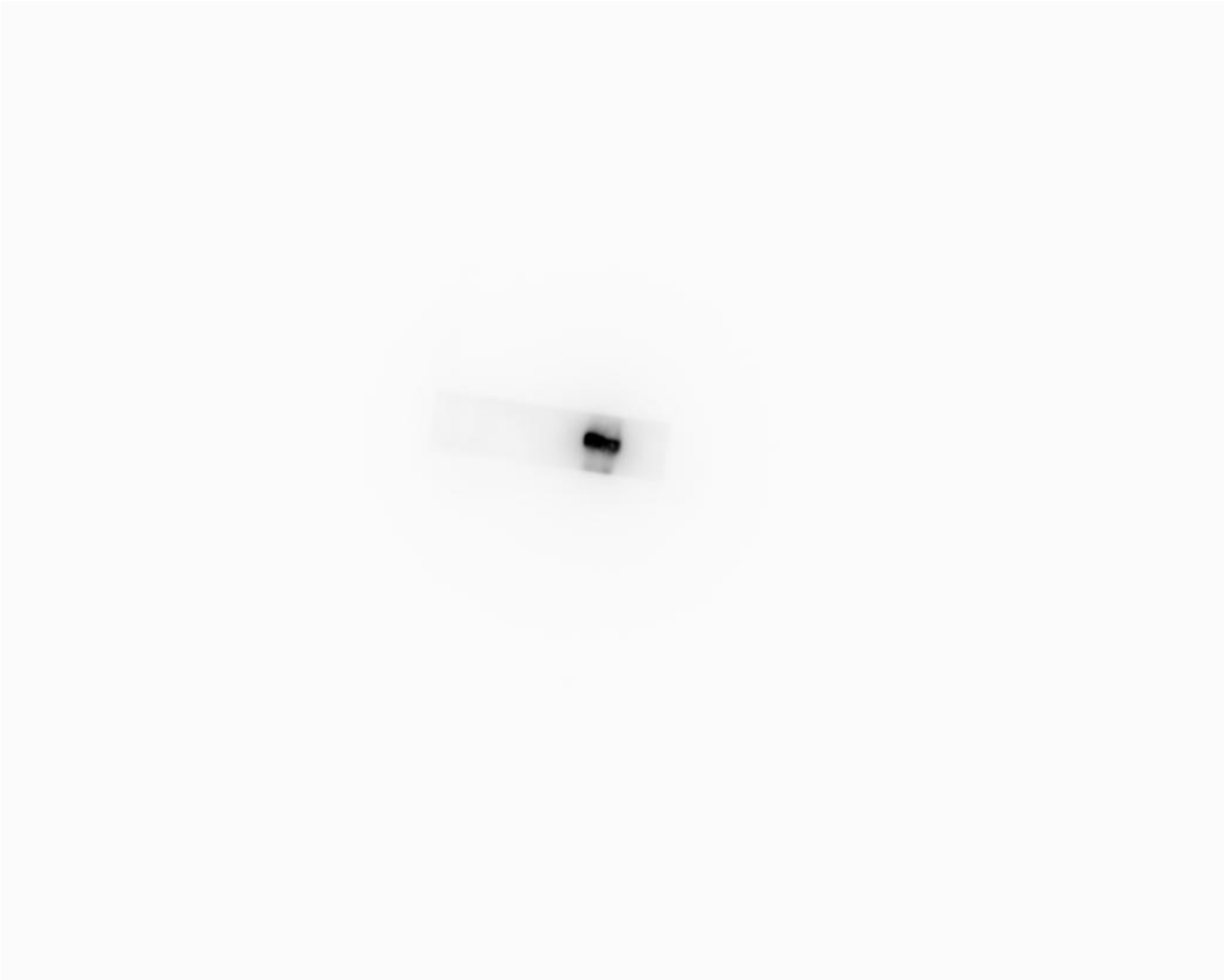

HA-Input

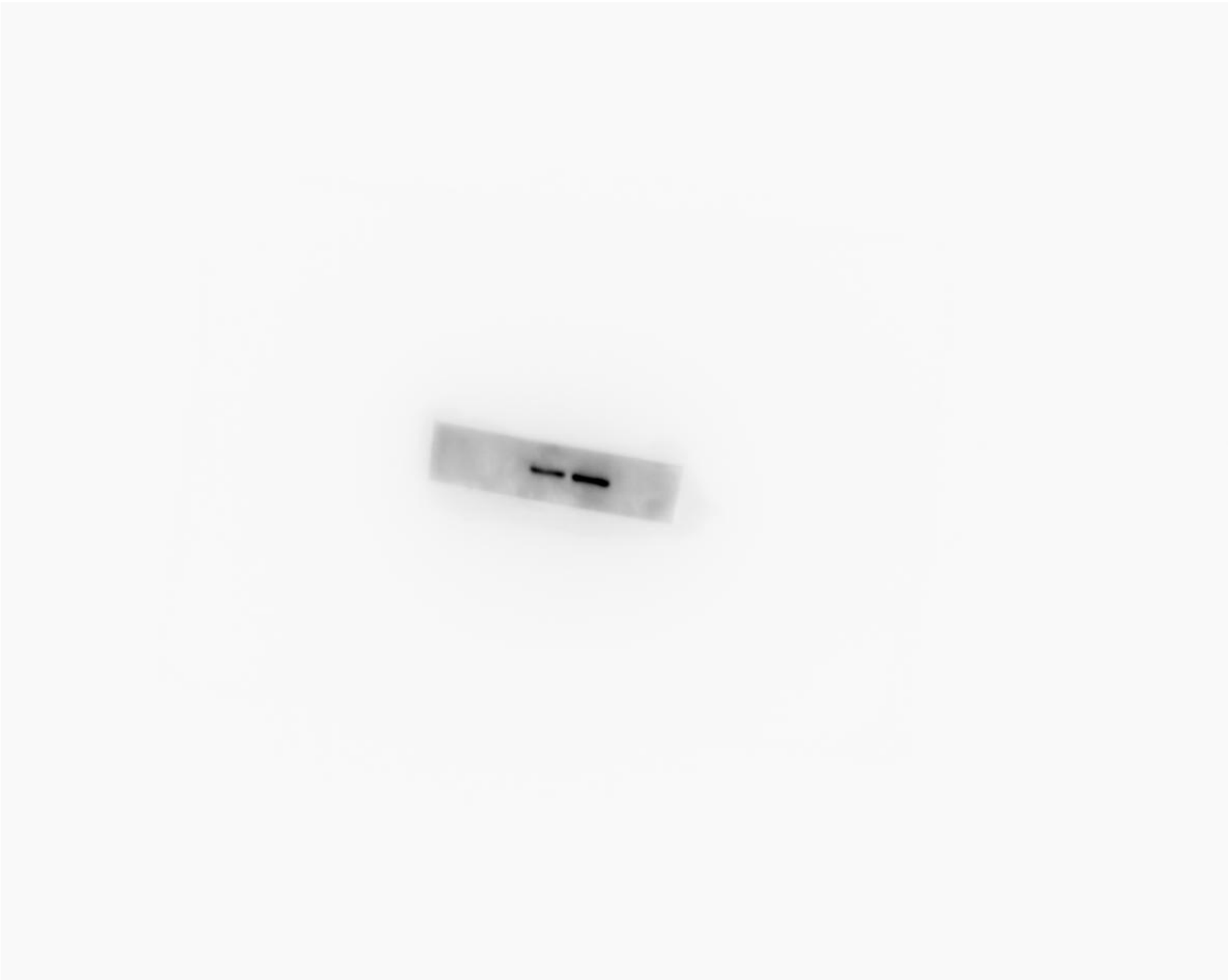

GFP-Input

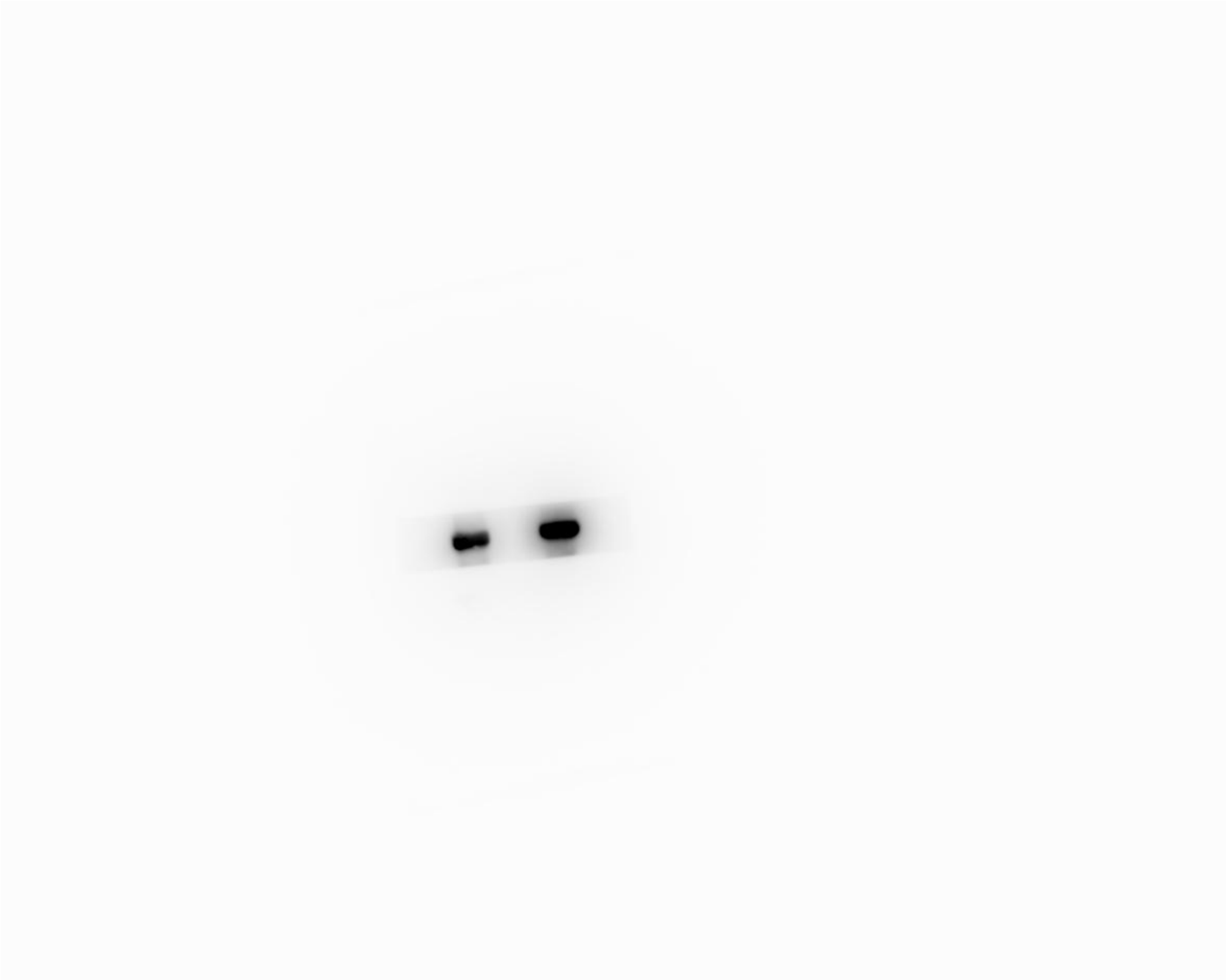

GAPDH

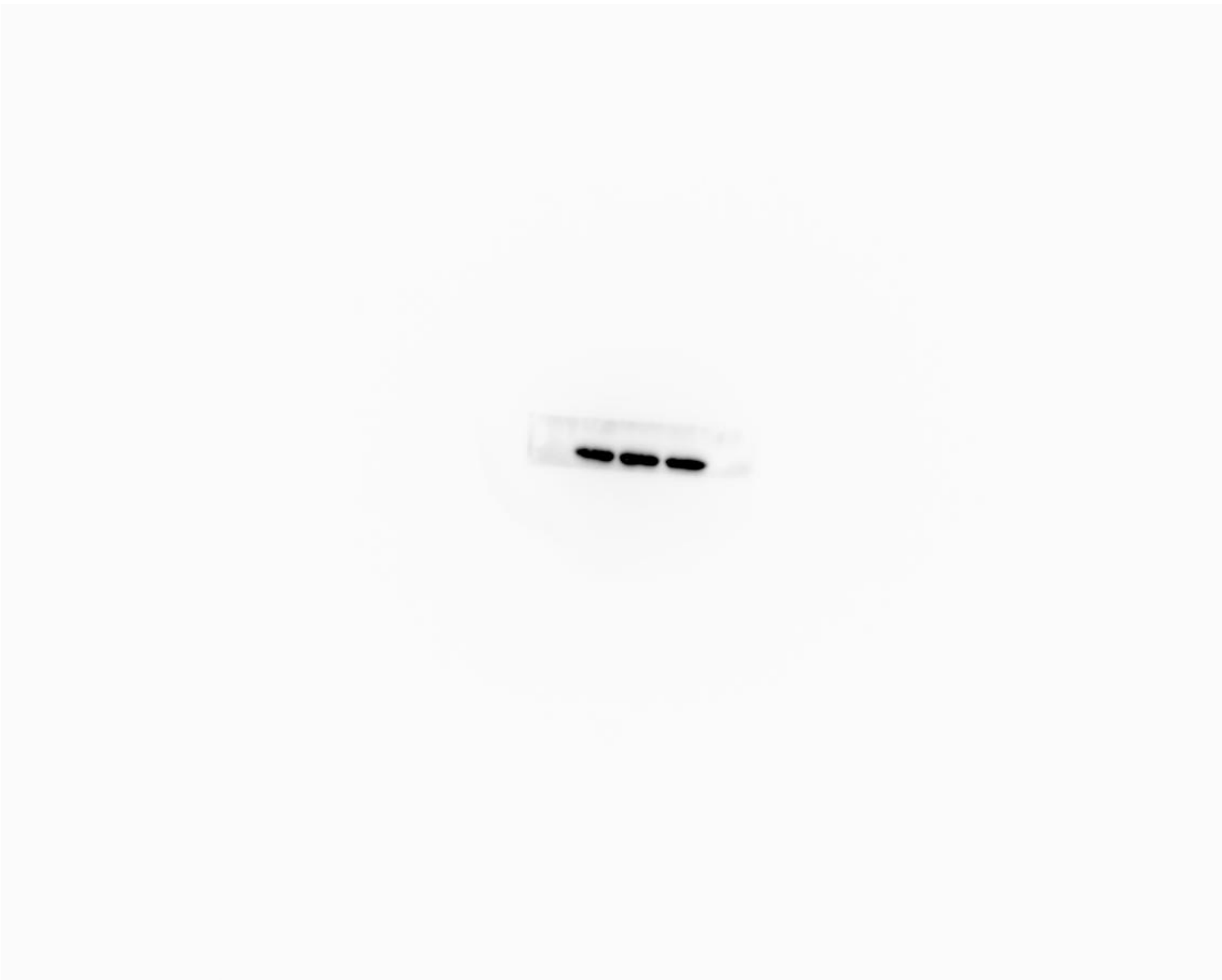

FigS3C

GFP-IP

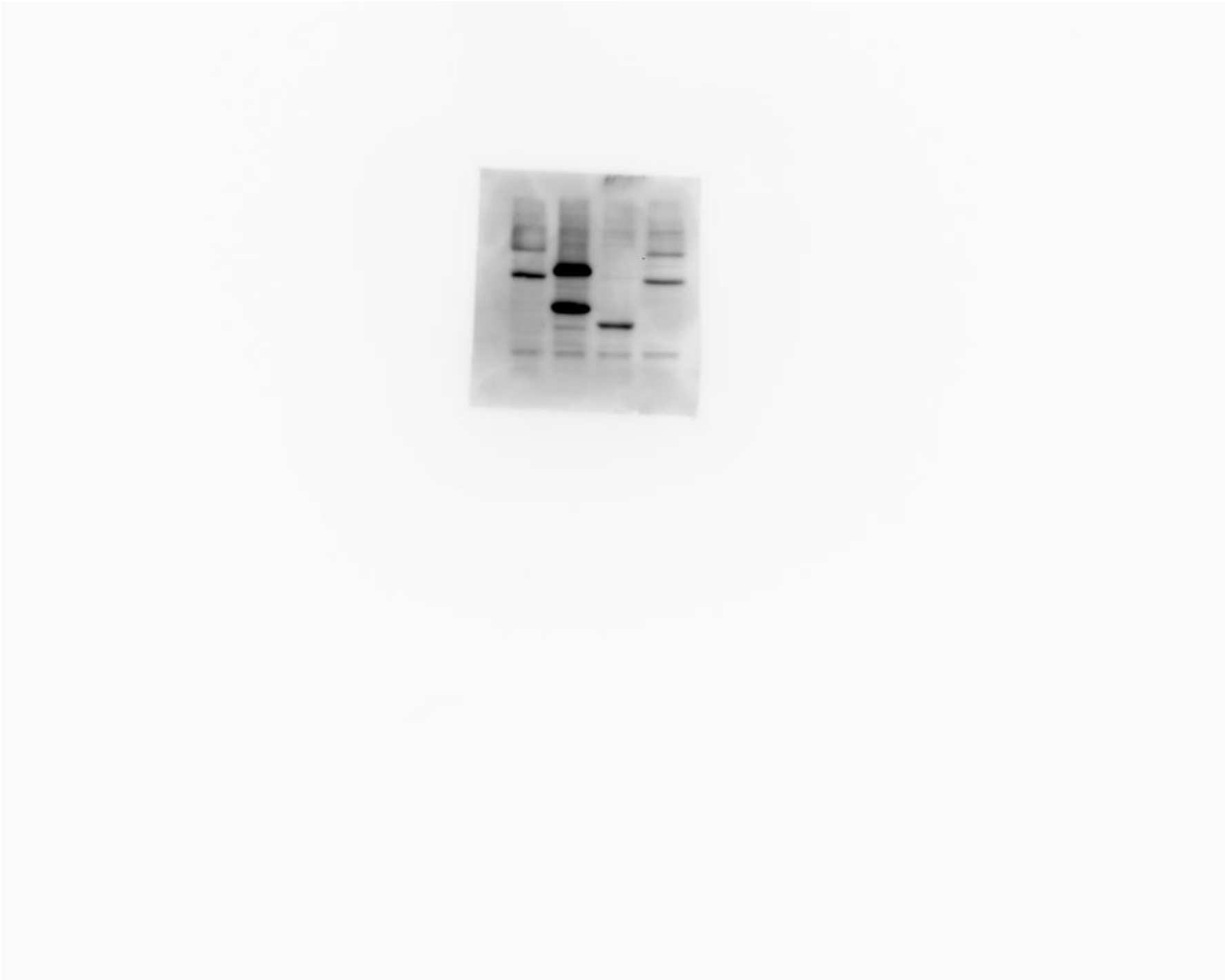

HA-IP

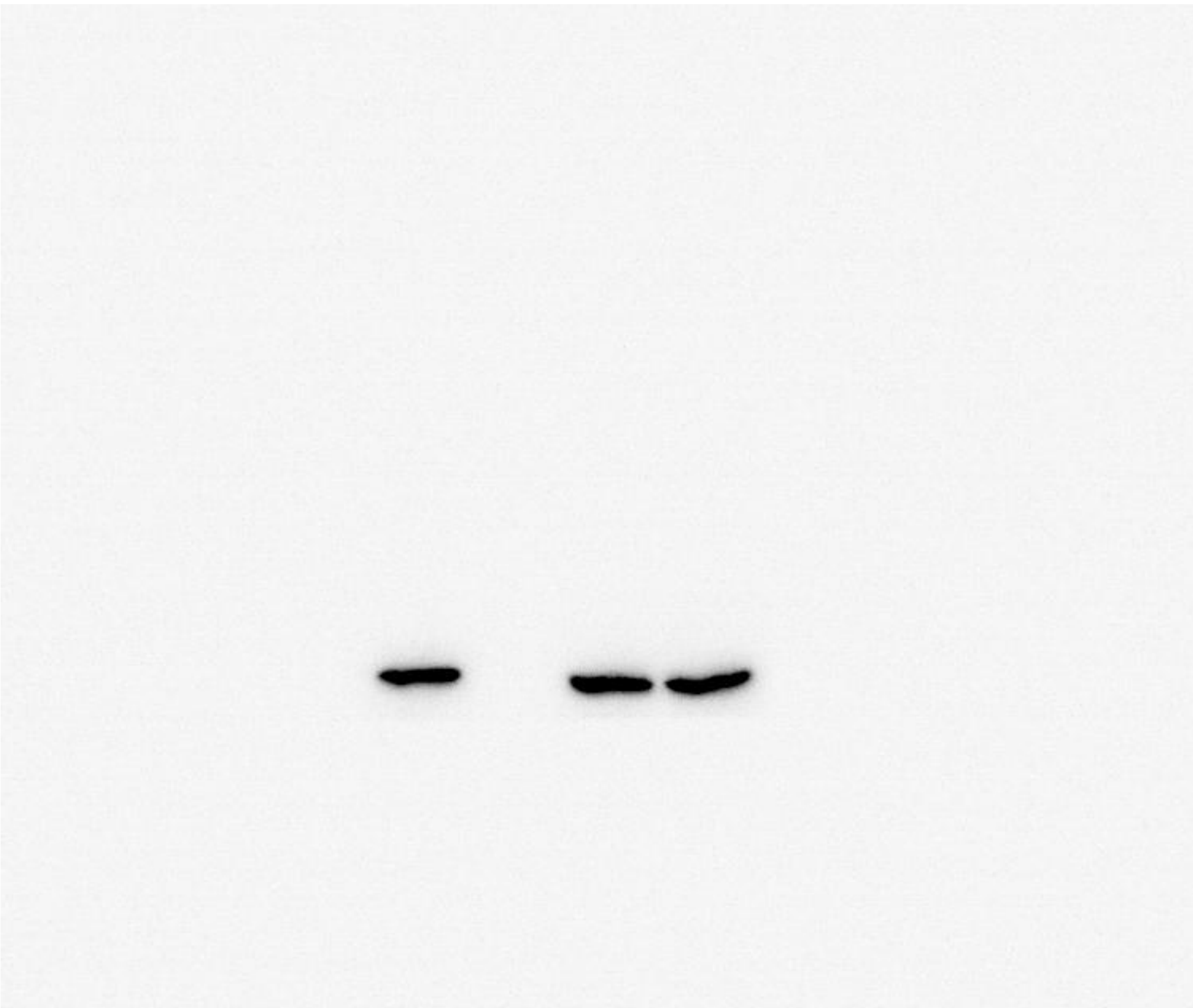

GFP-Input

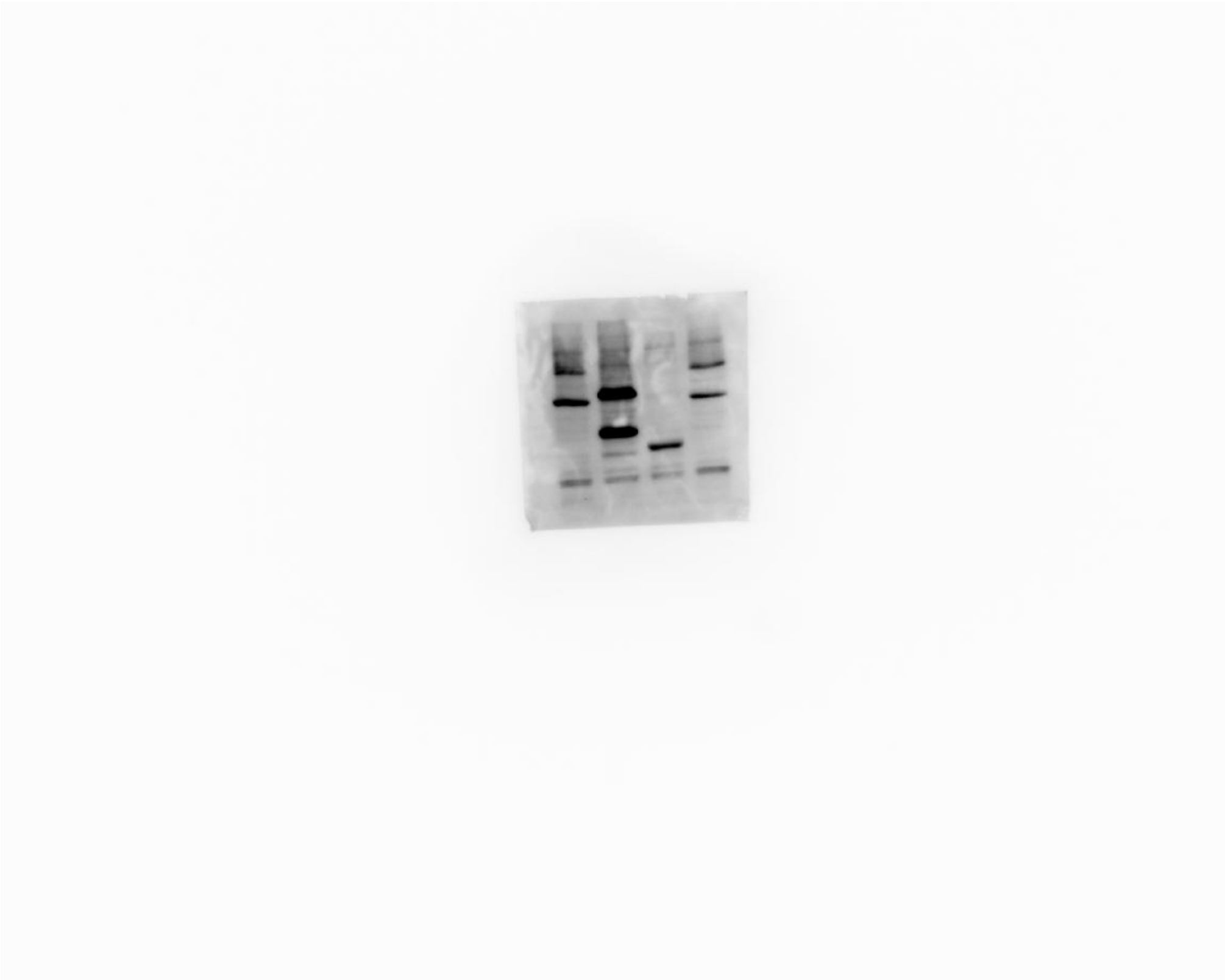

HA-Input

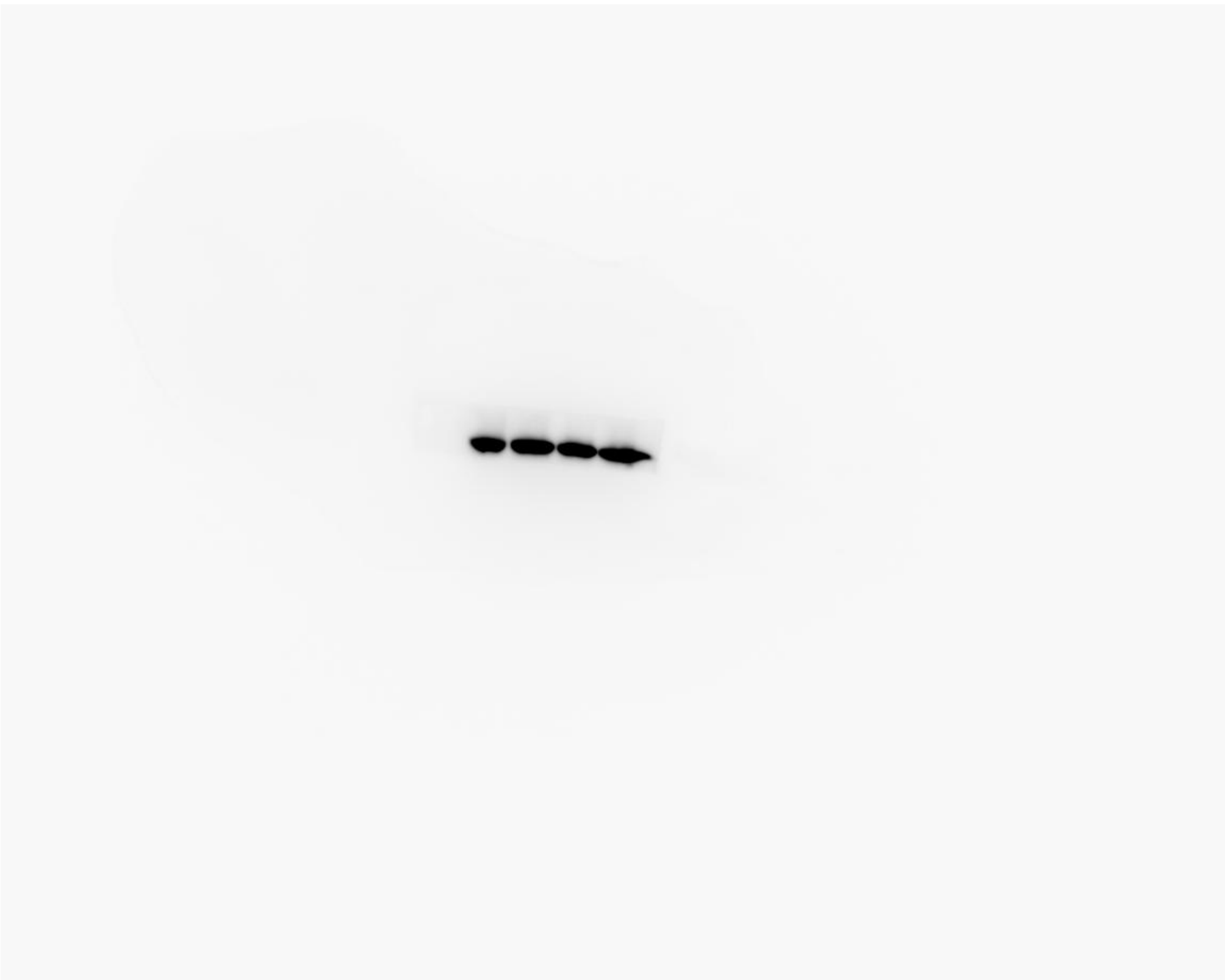

FigS3D

PRMT2

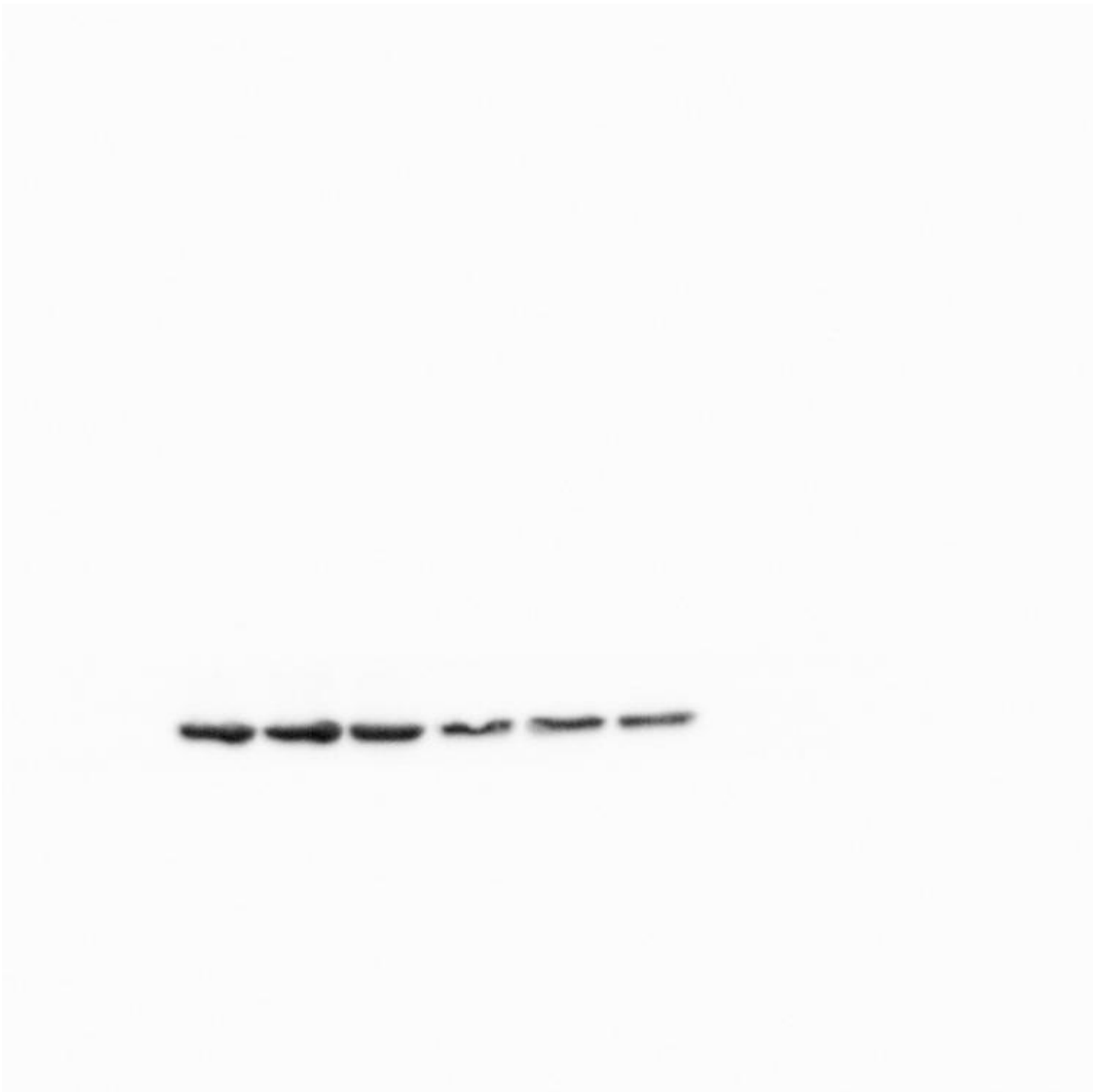

GAPDH

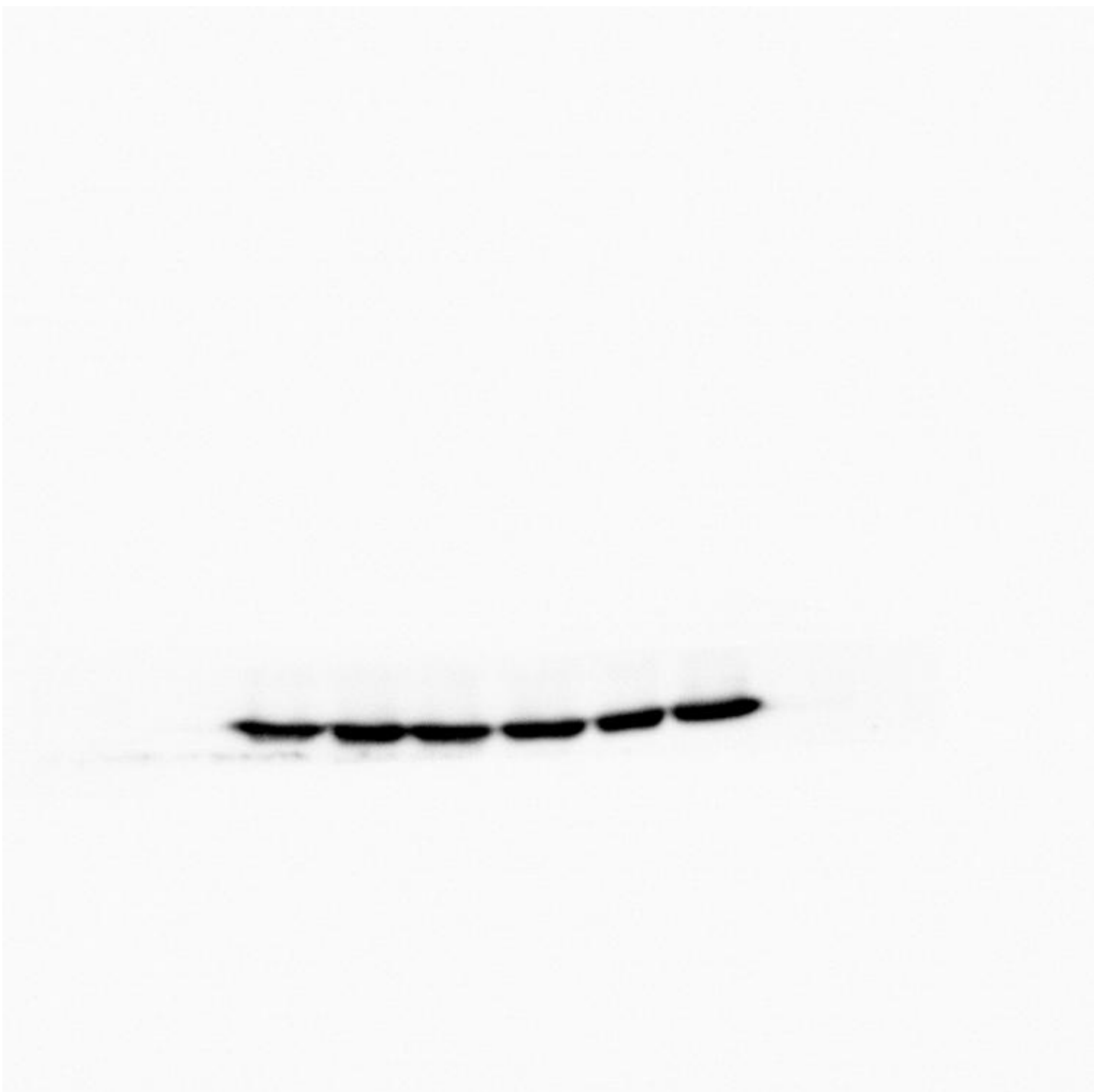

FigS3E

HIF1α

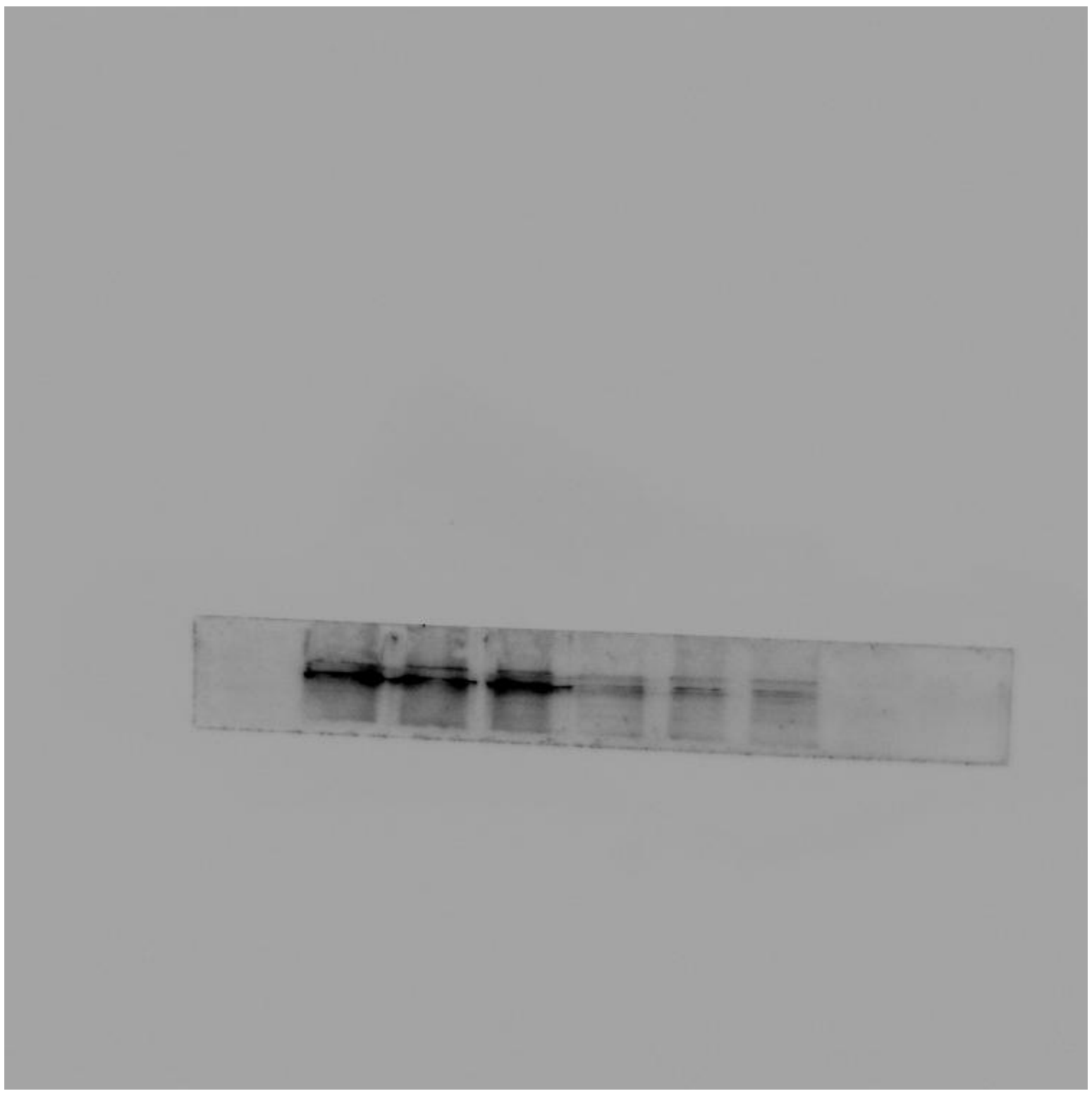

PRMT2

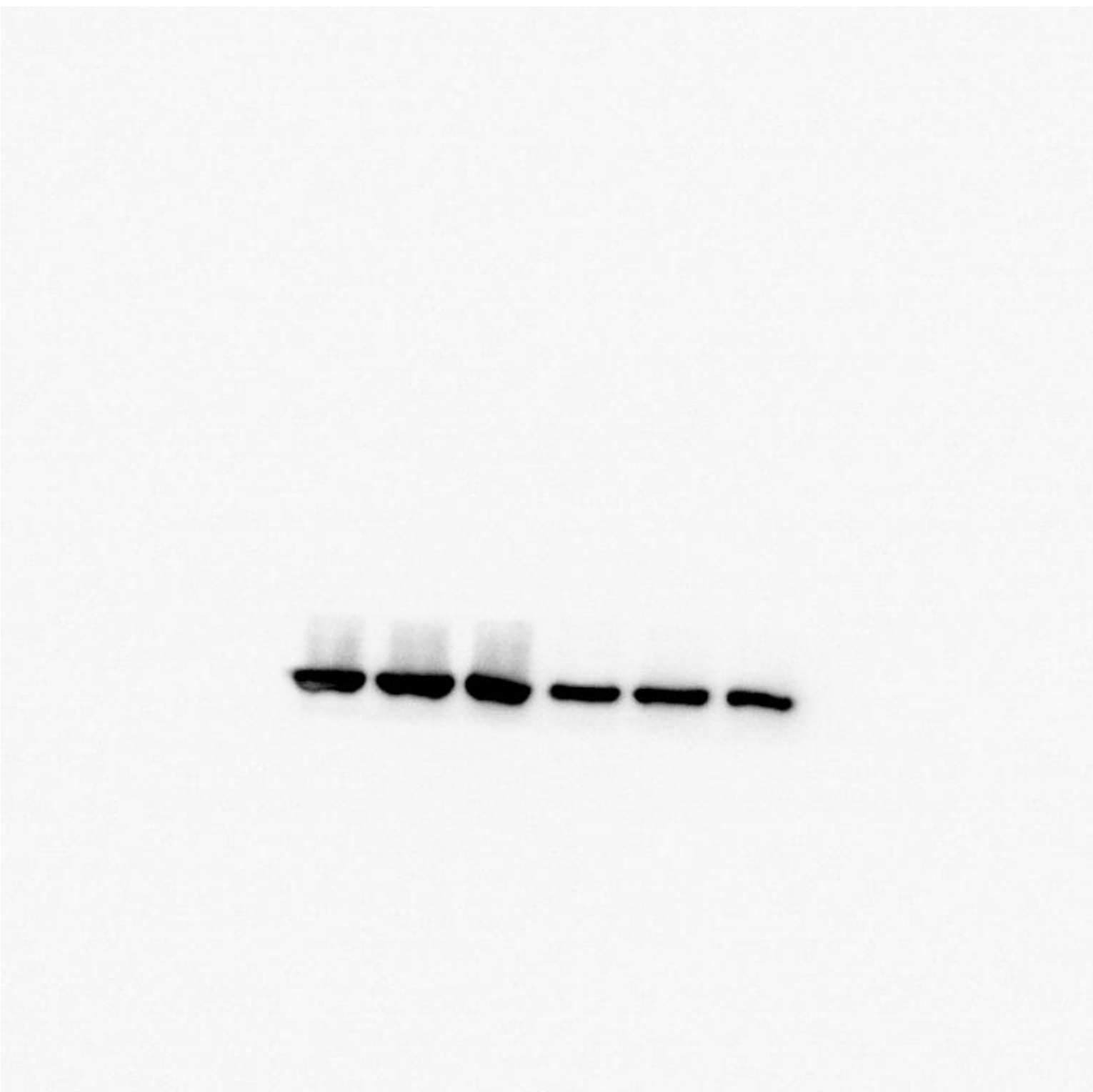

GAPDH

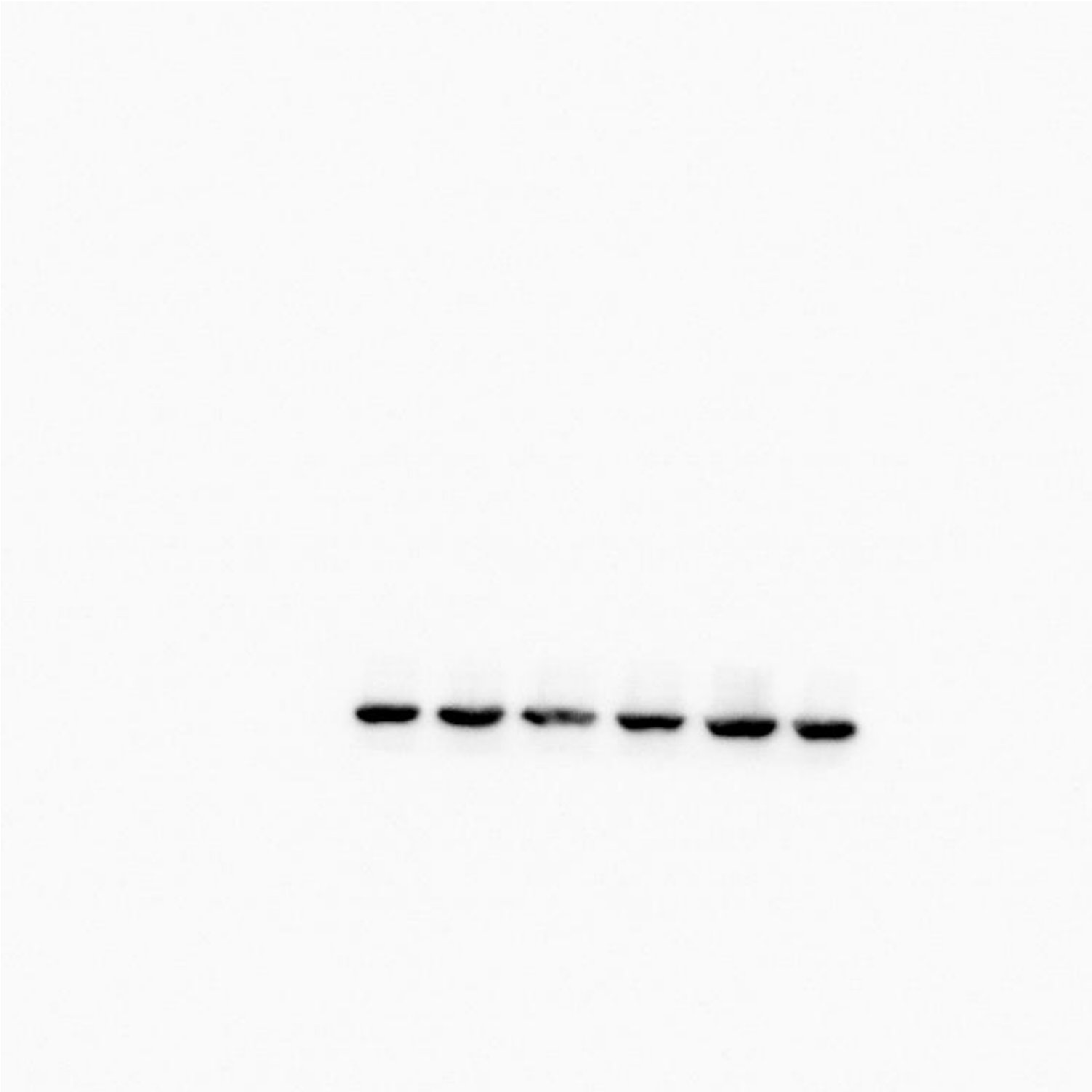

FigS3F

HIF1α

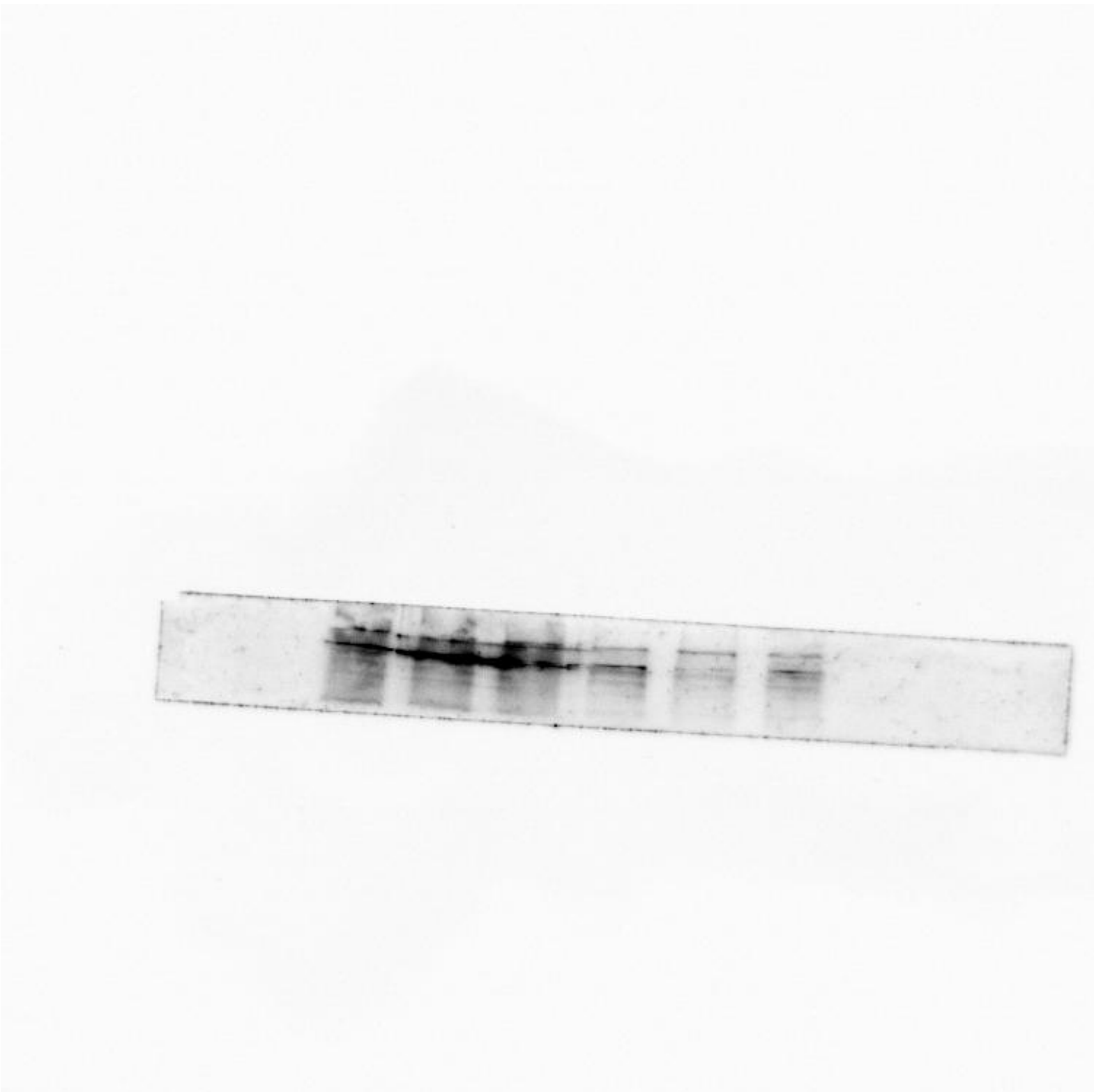

PRMT2

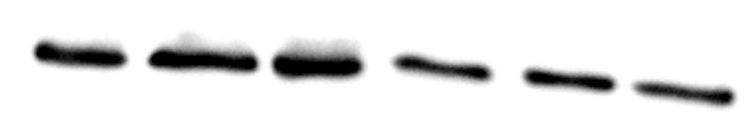

GPADH

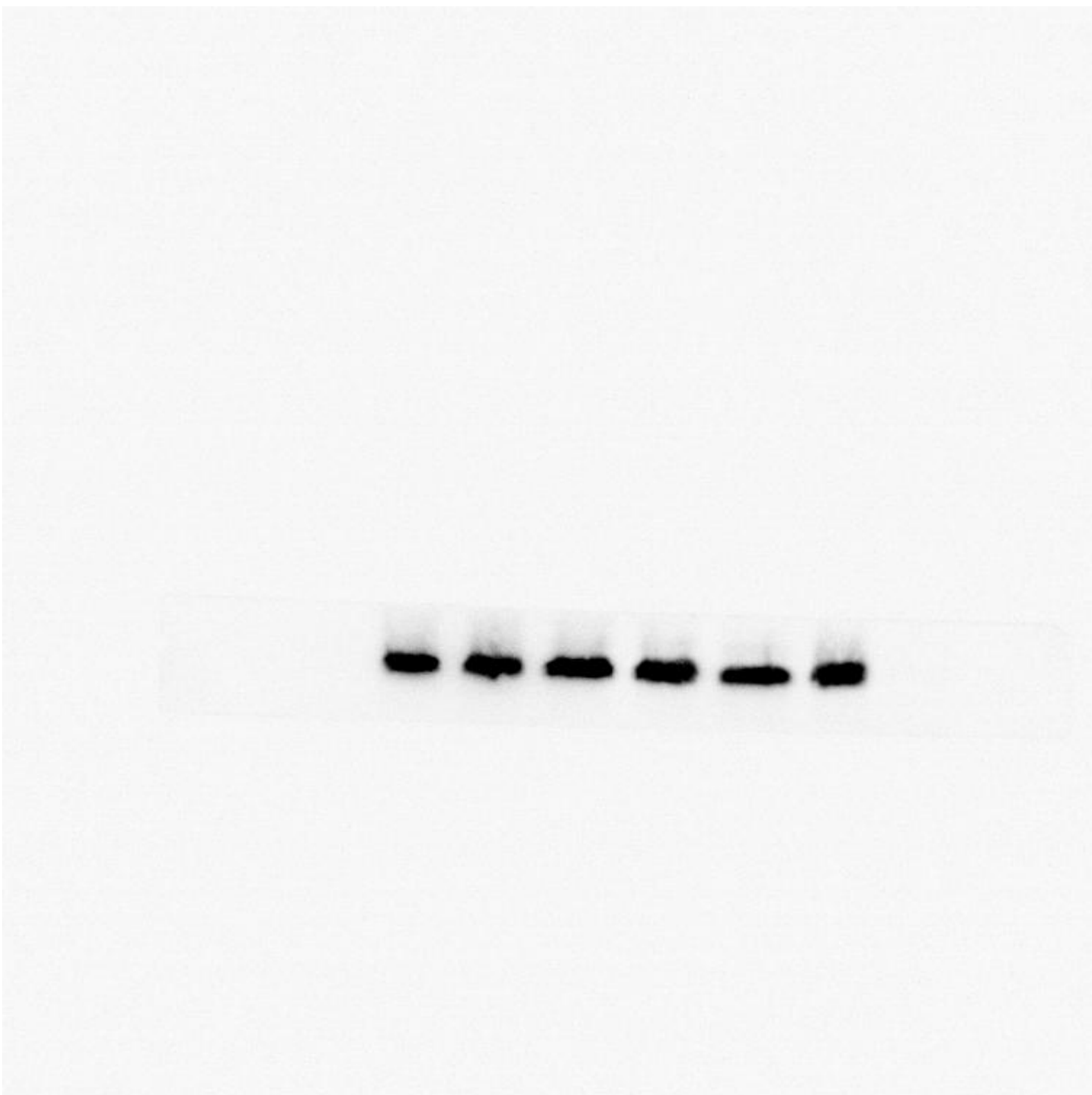

FigS3I

PP53

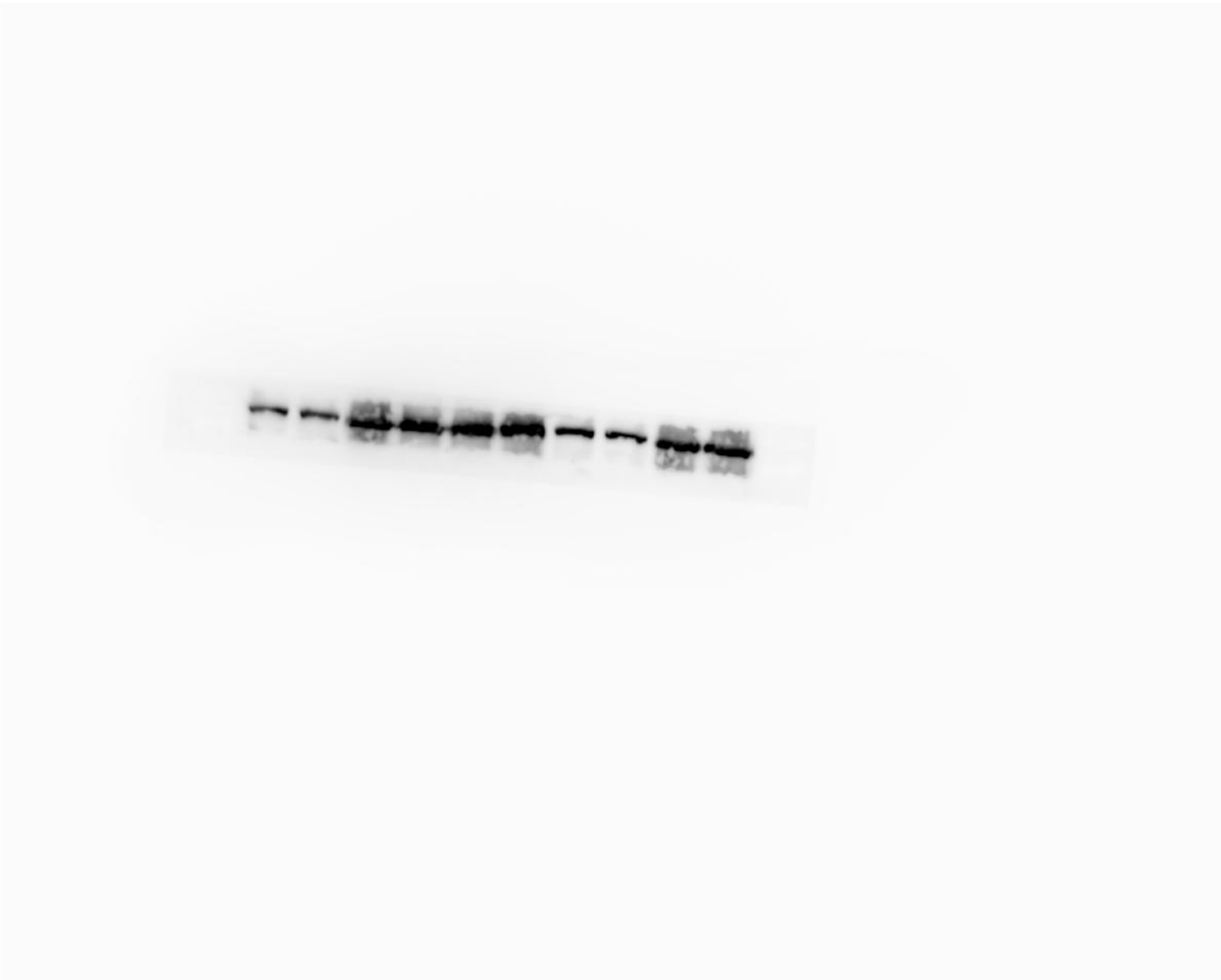

P53

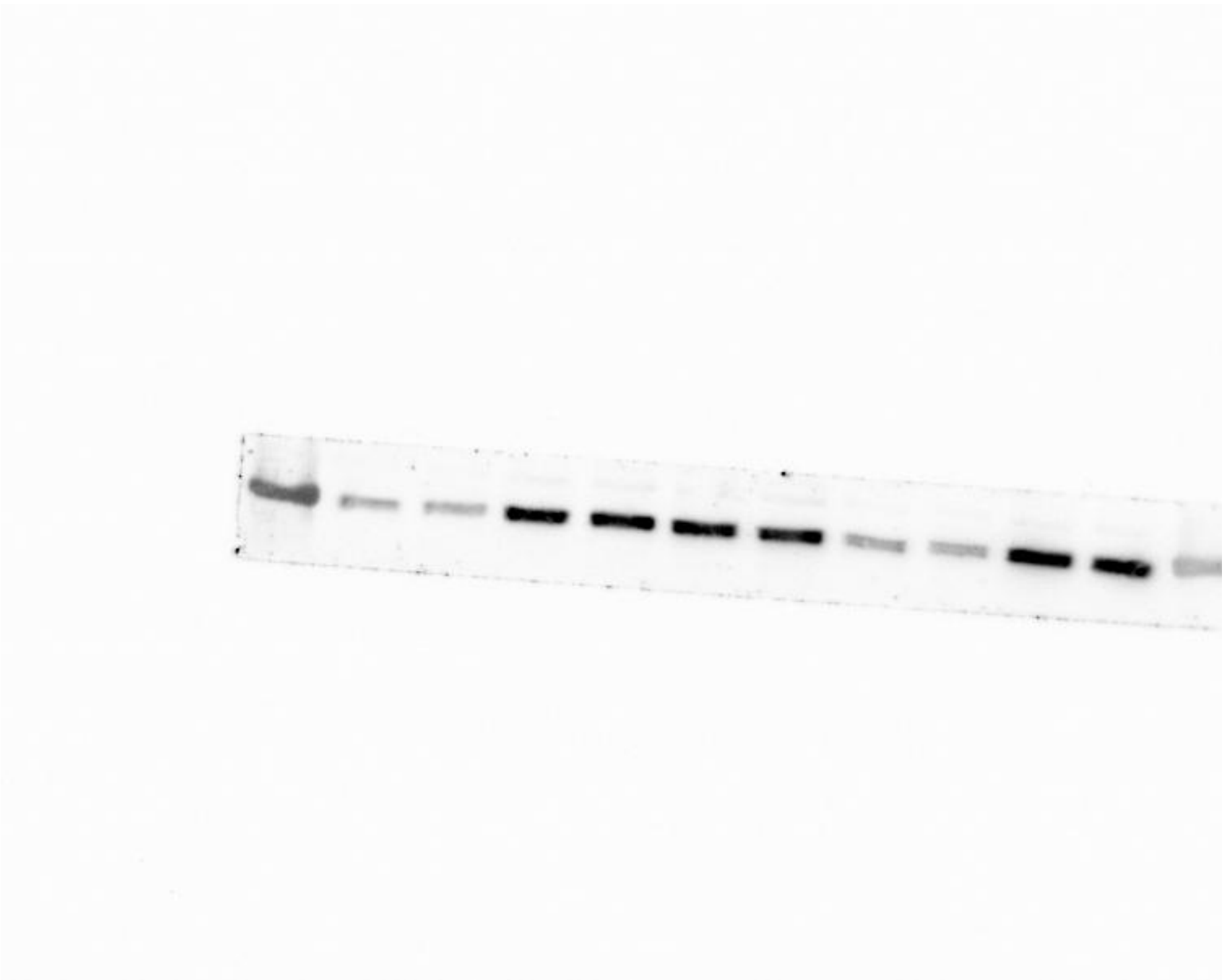

P21

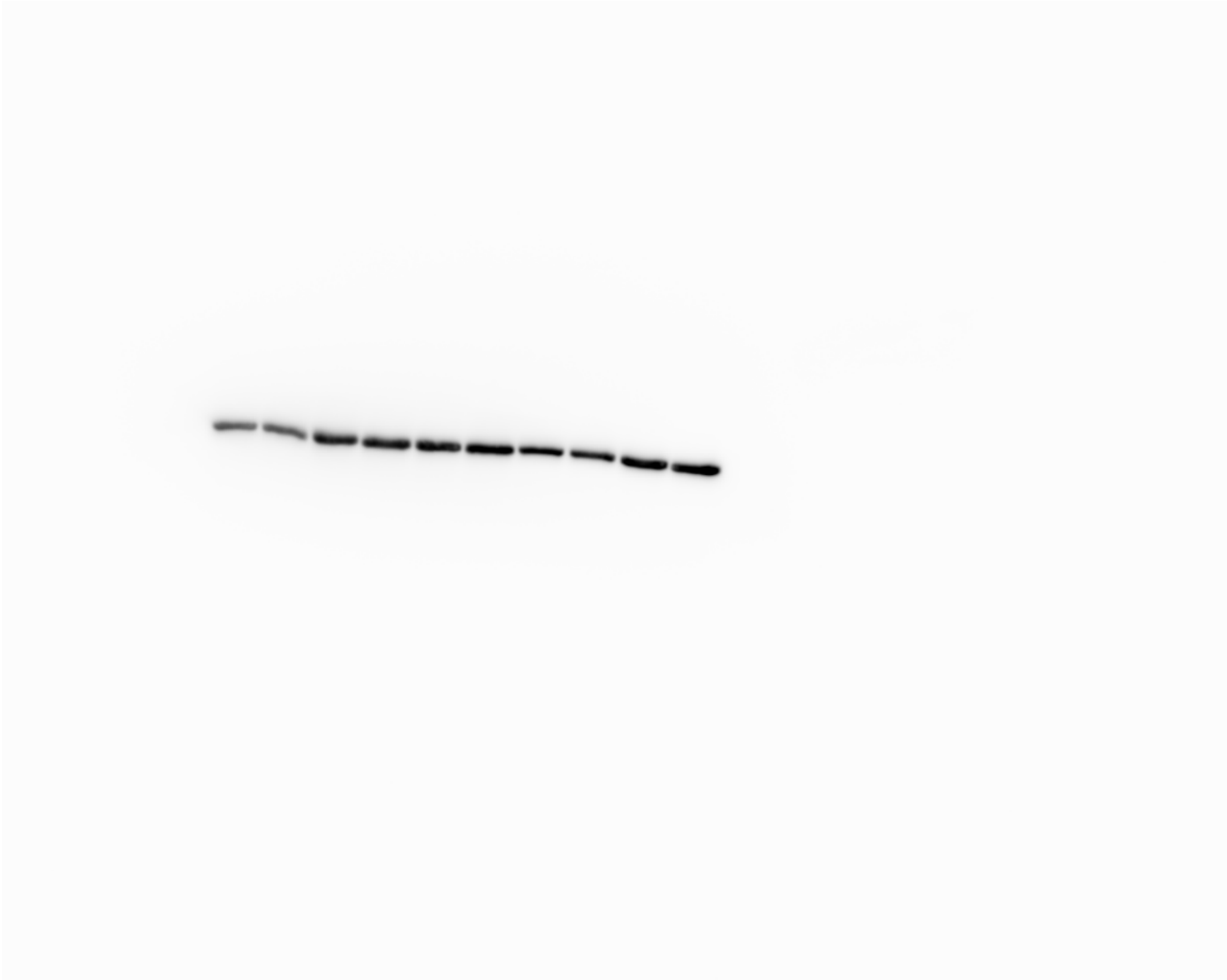

P16

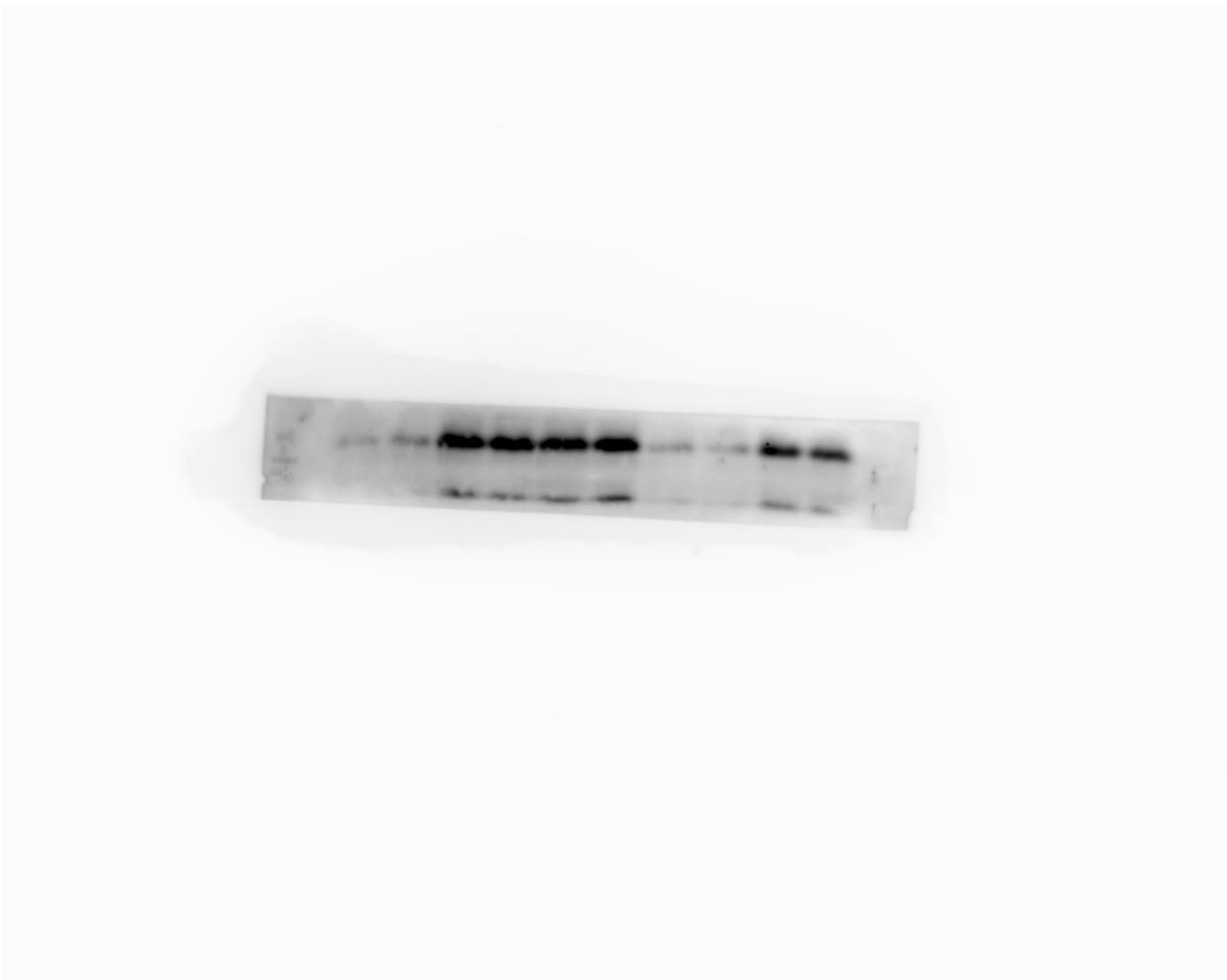

GAPDH

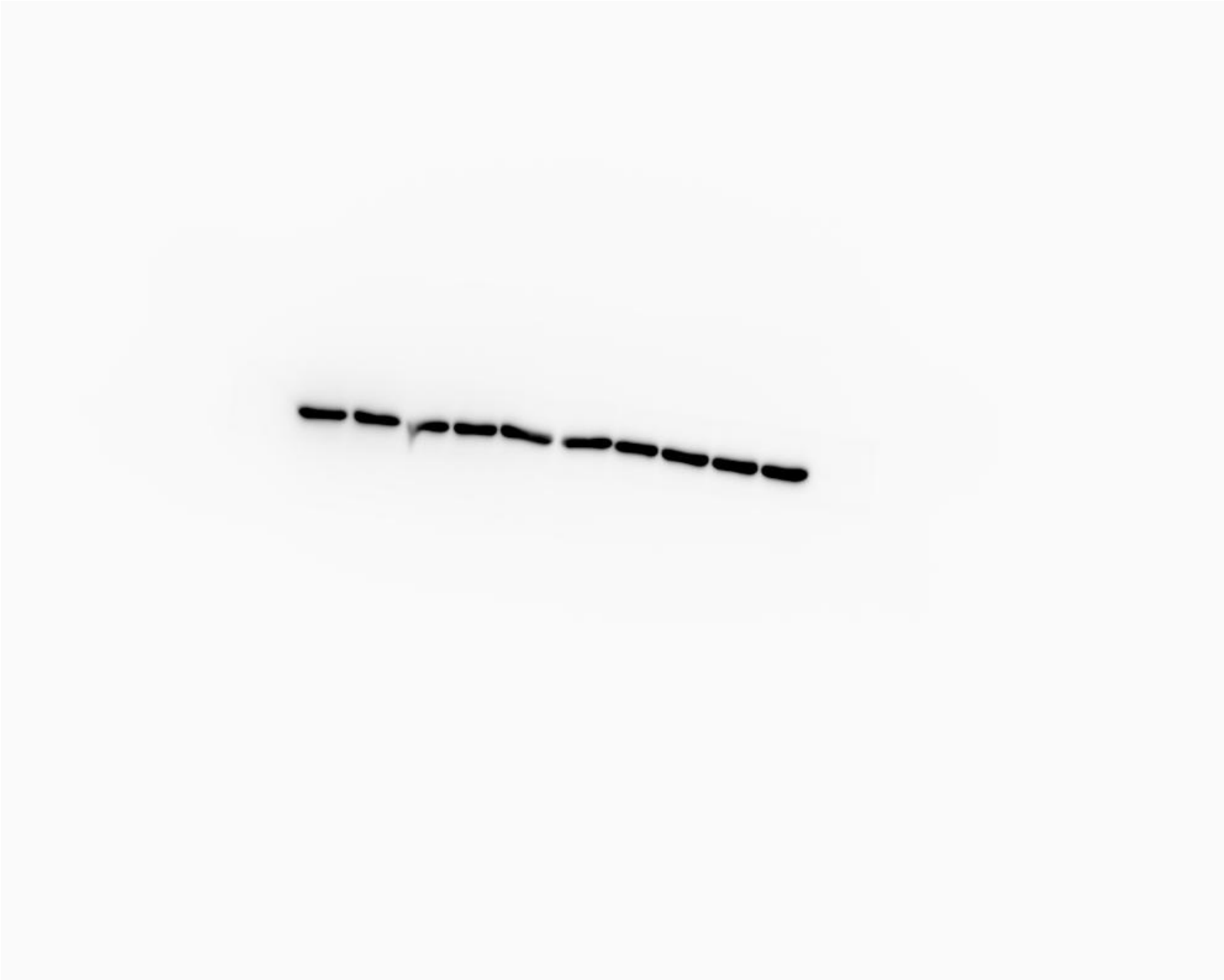

FigS5A

NUMA1

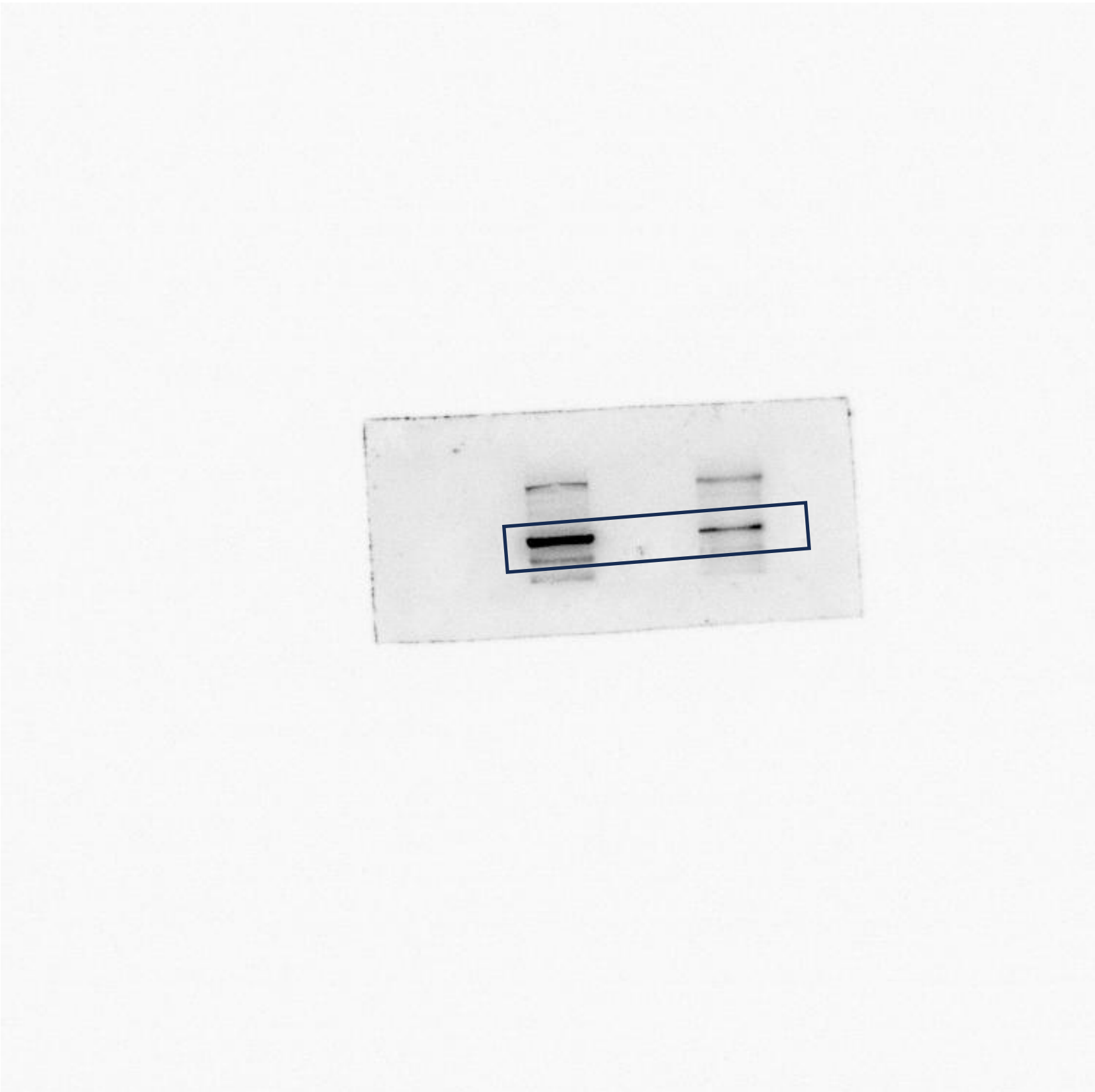

THRAP3

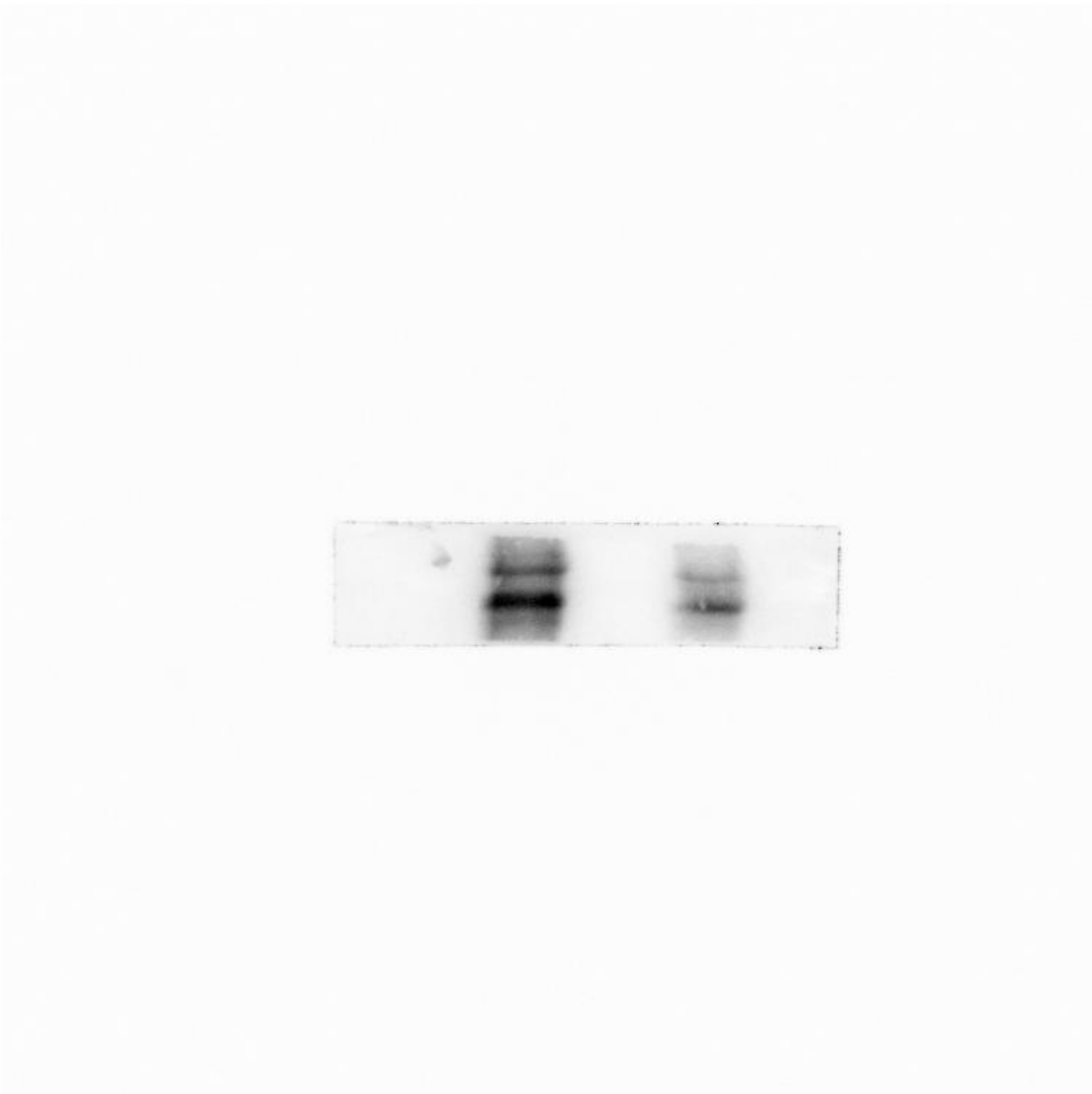

H1.2

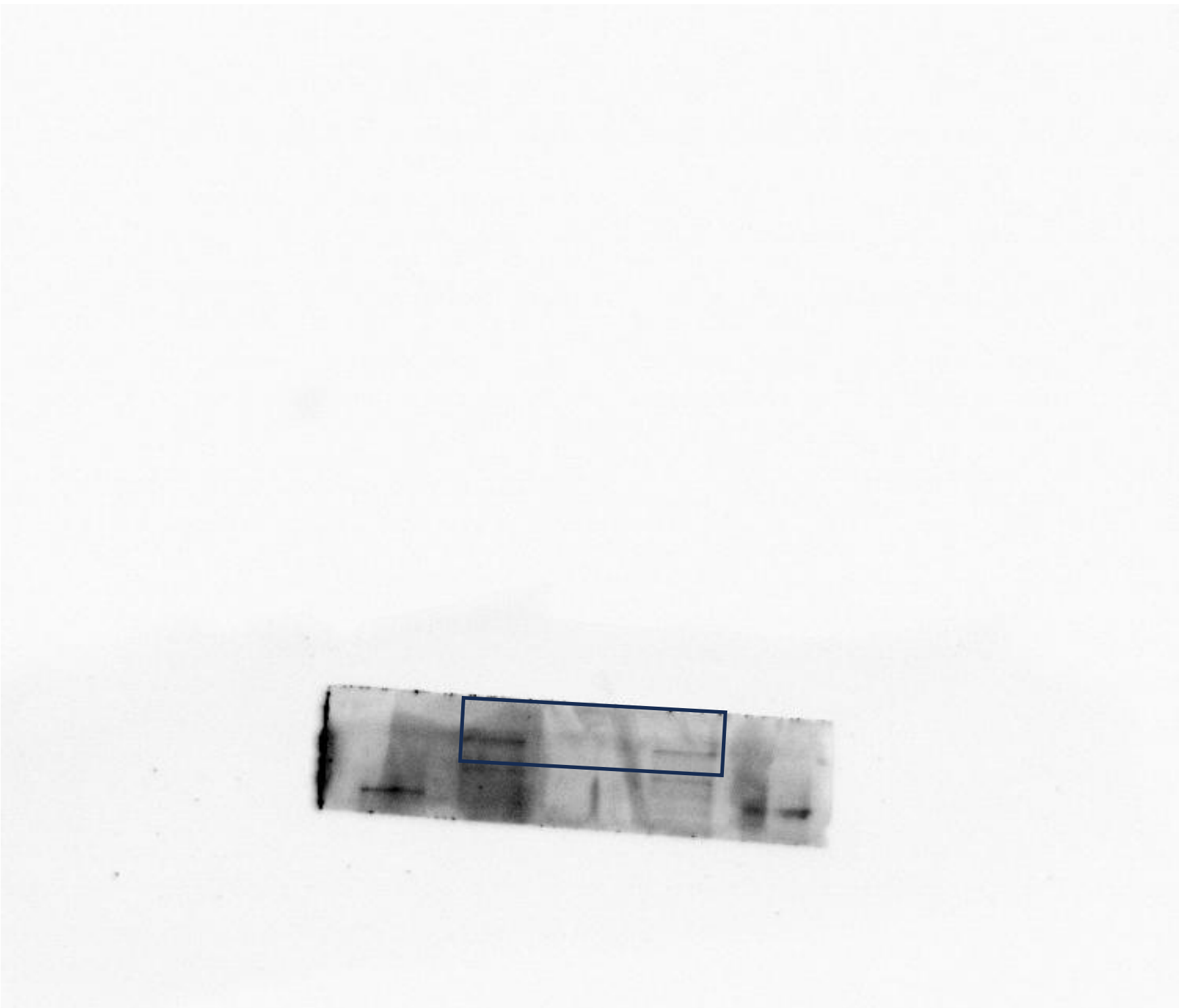

SEC23A

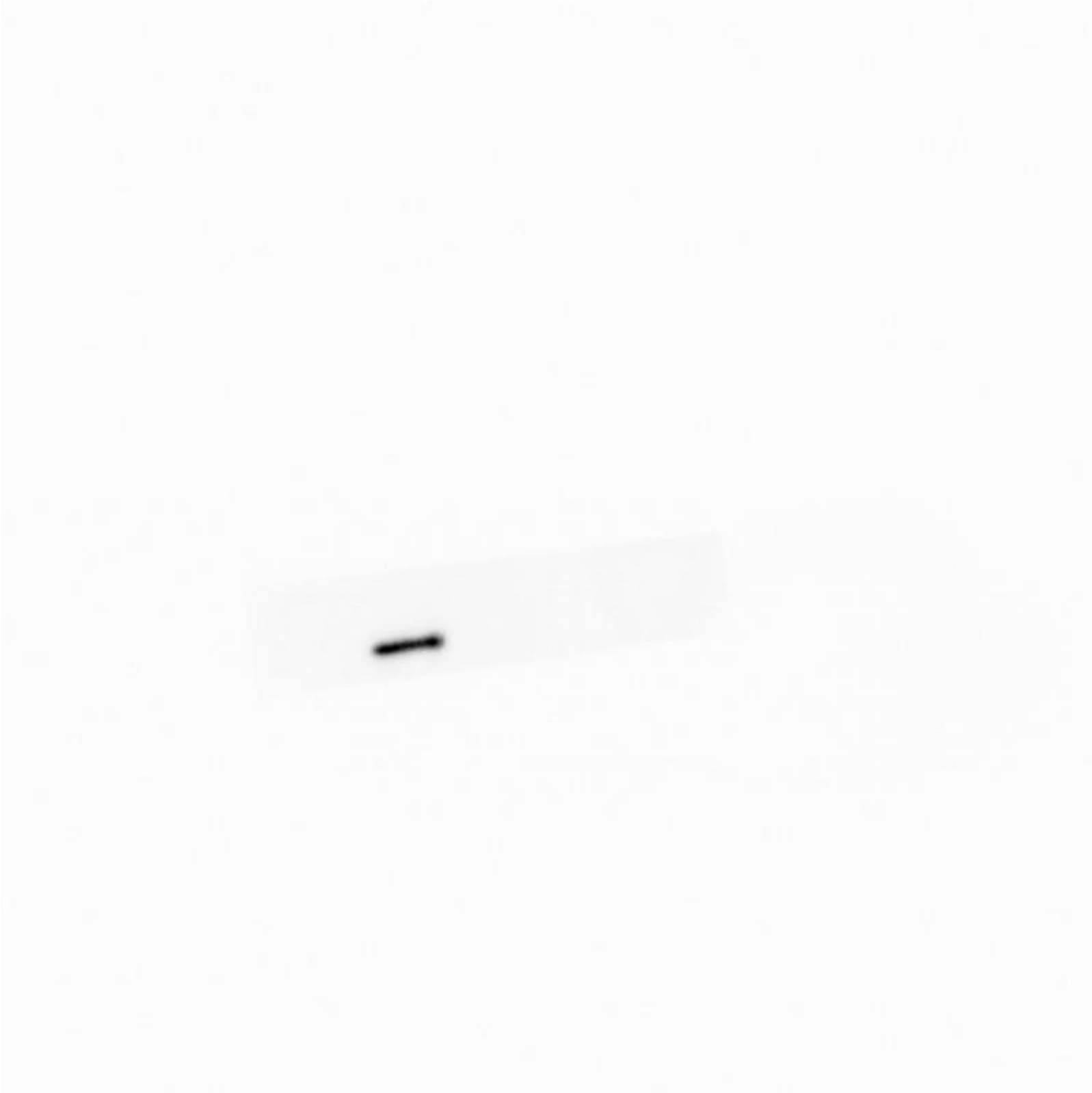

RPL29

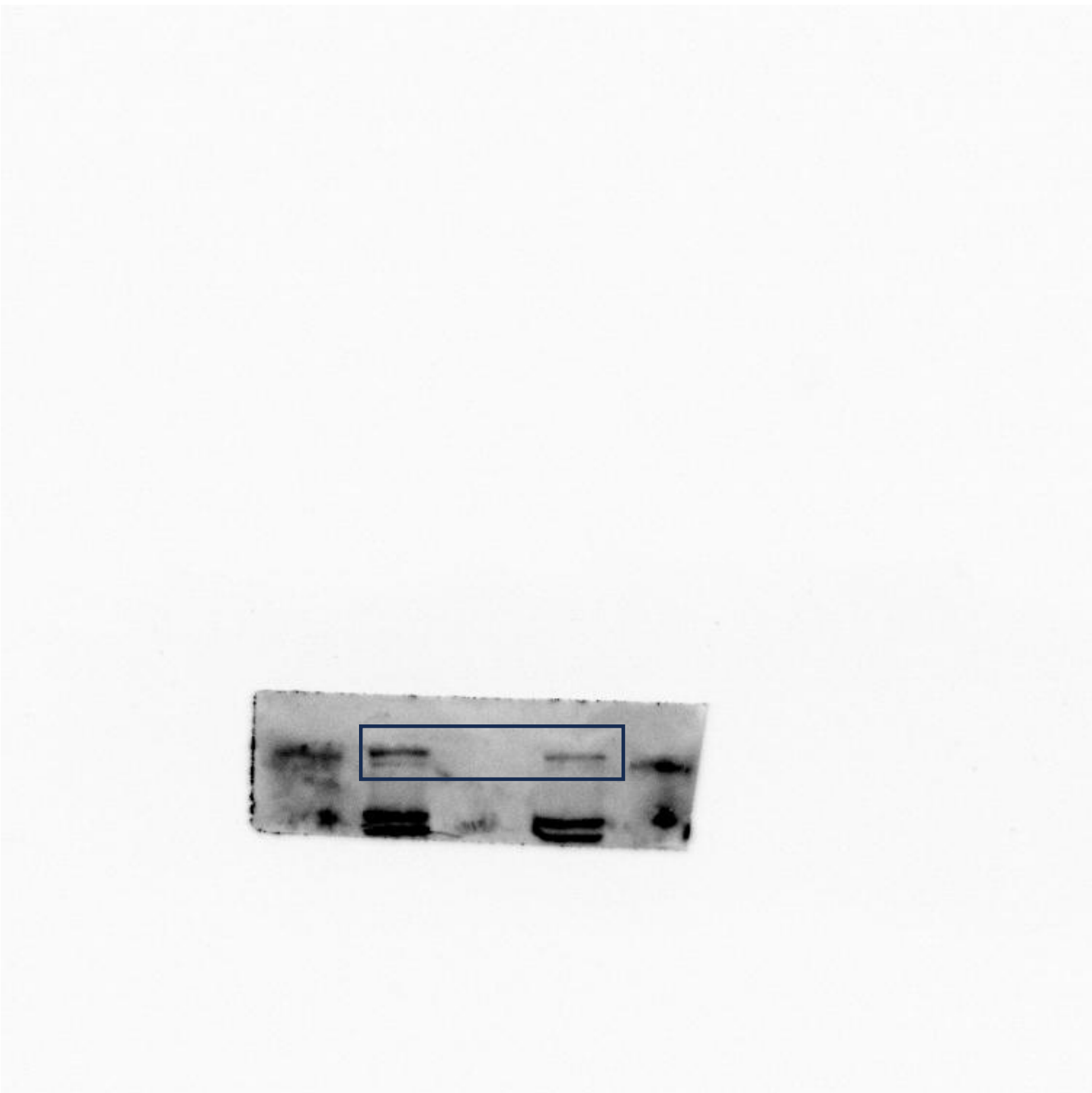

TRA2B

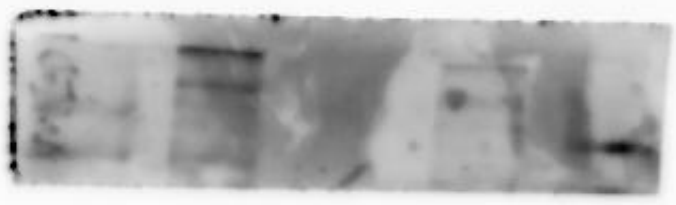

MED12

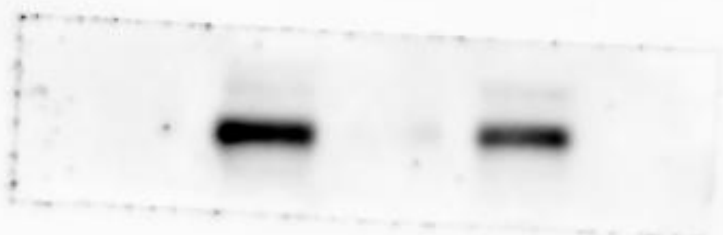

FBXO7

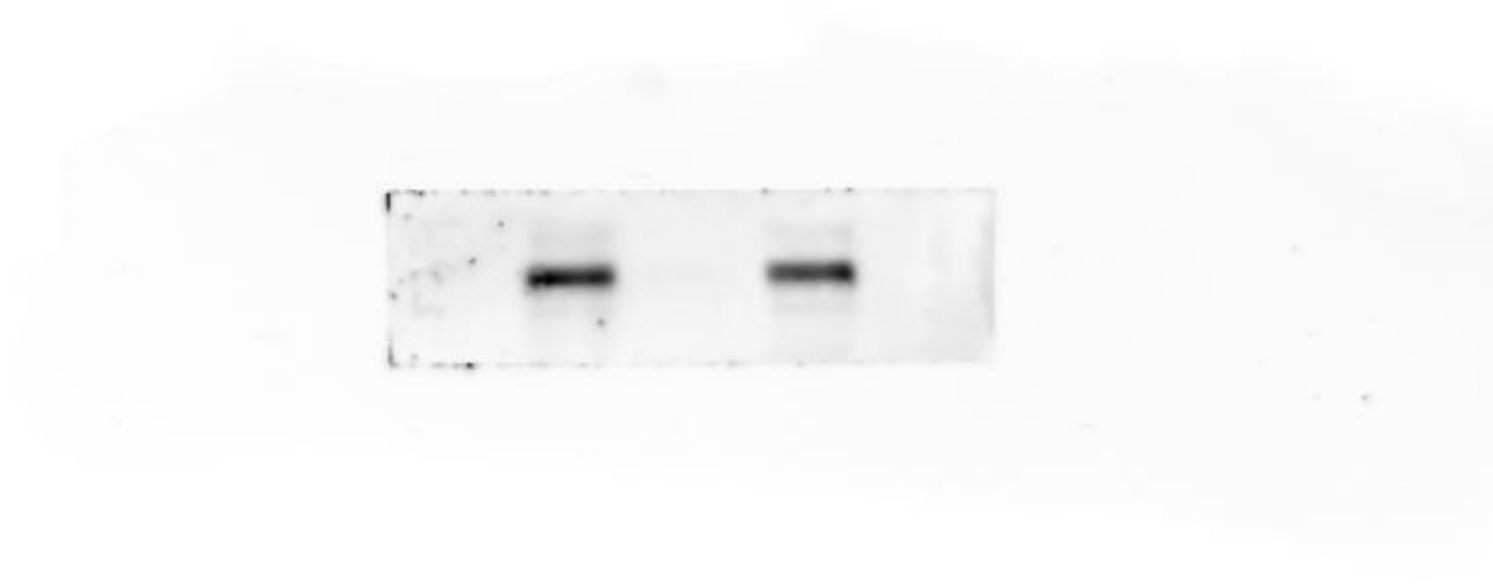

FigS5B

Flag-IP

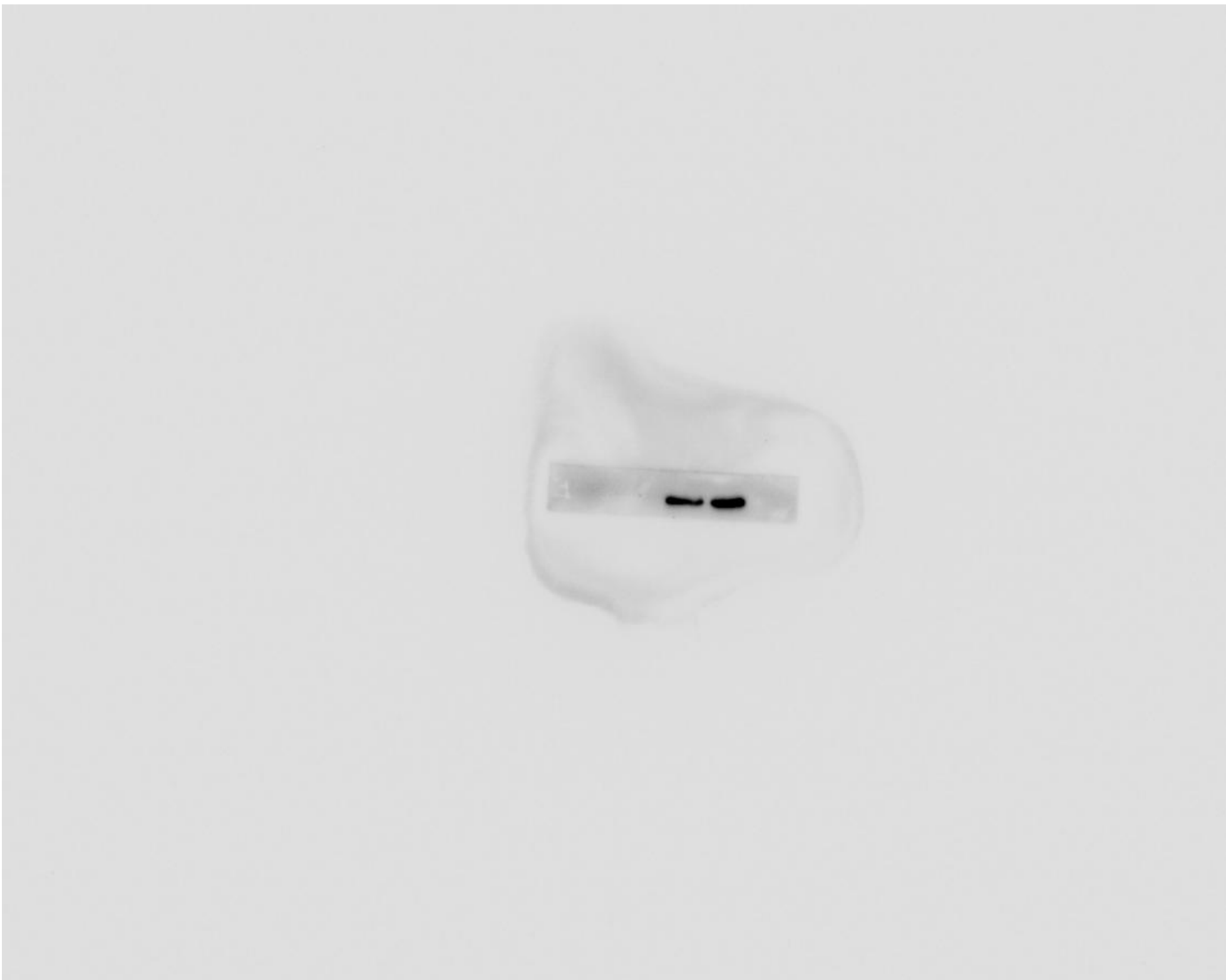

GFP-IP

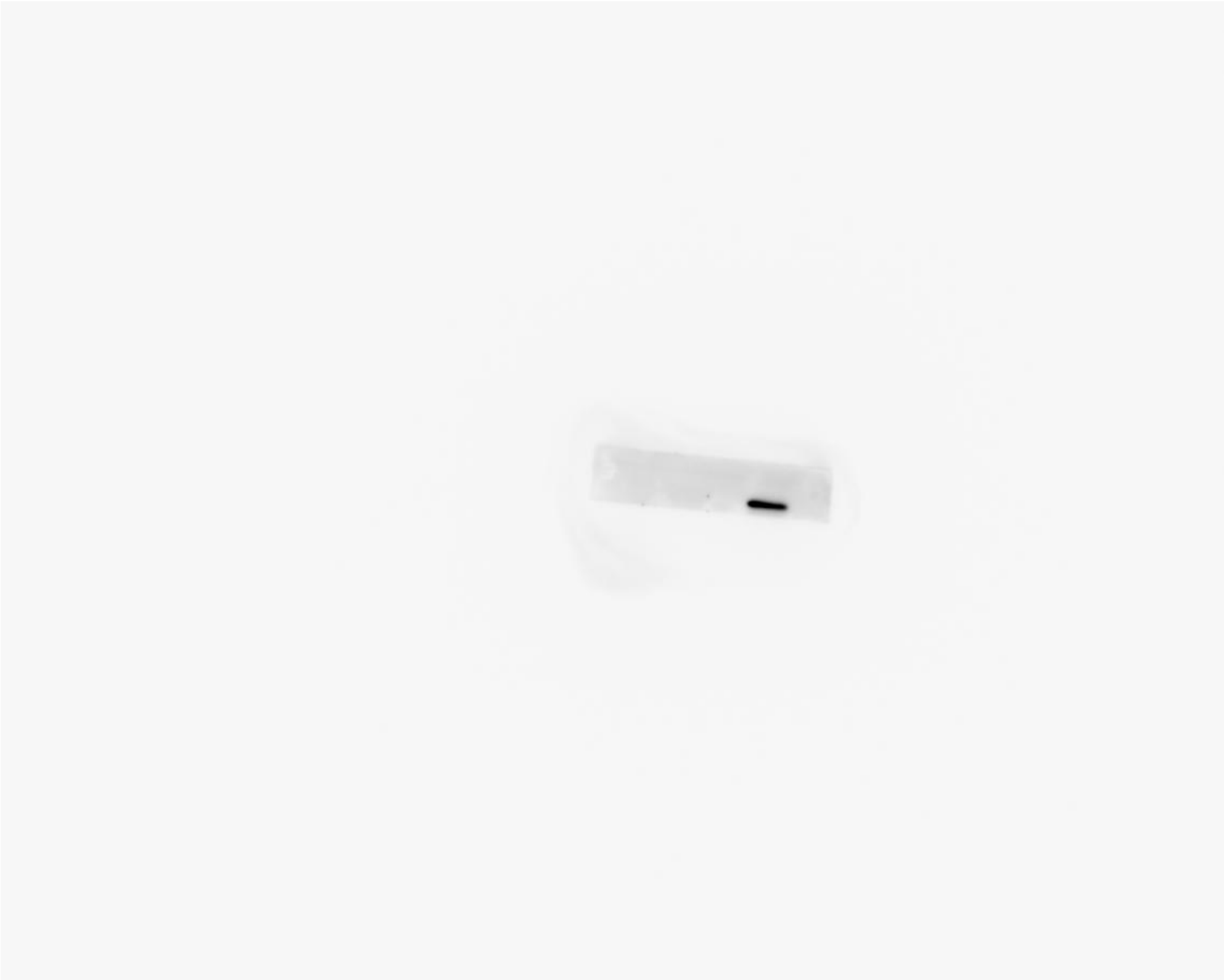

Flag-Input

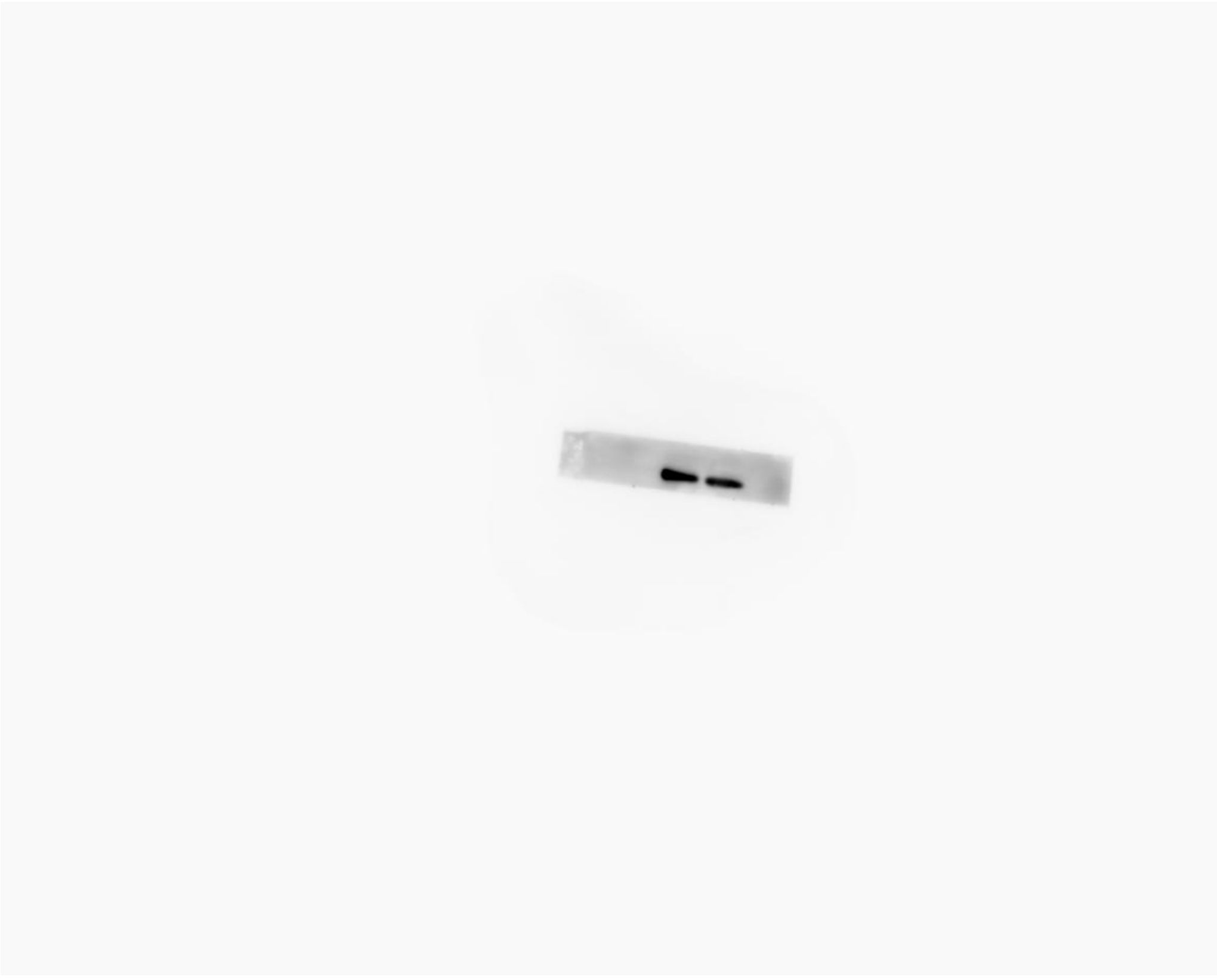

GFP-Input

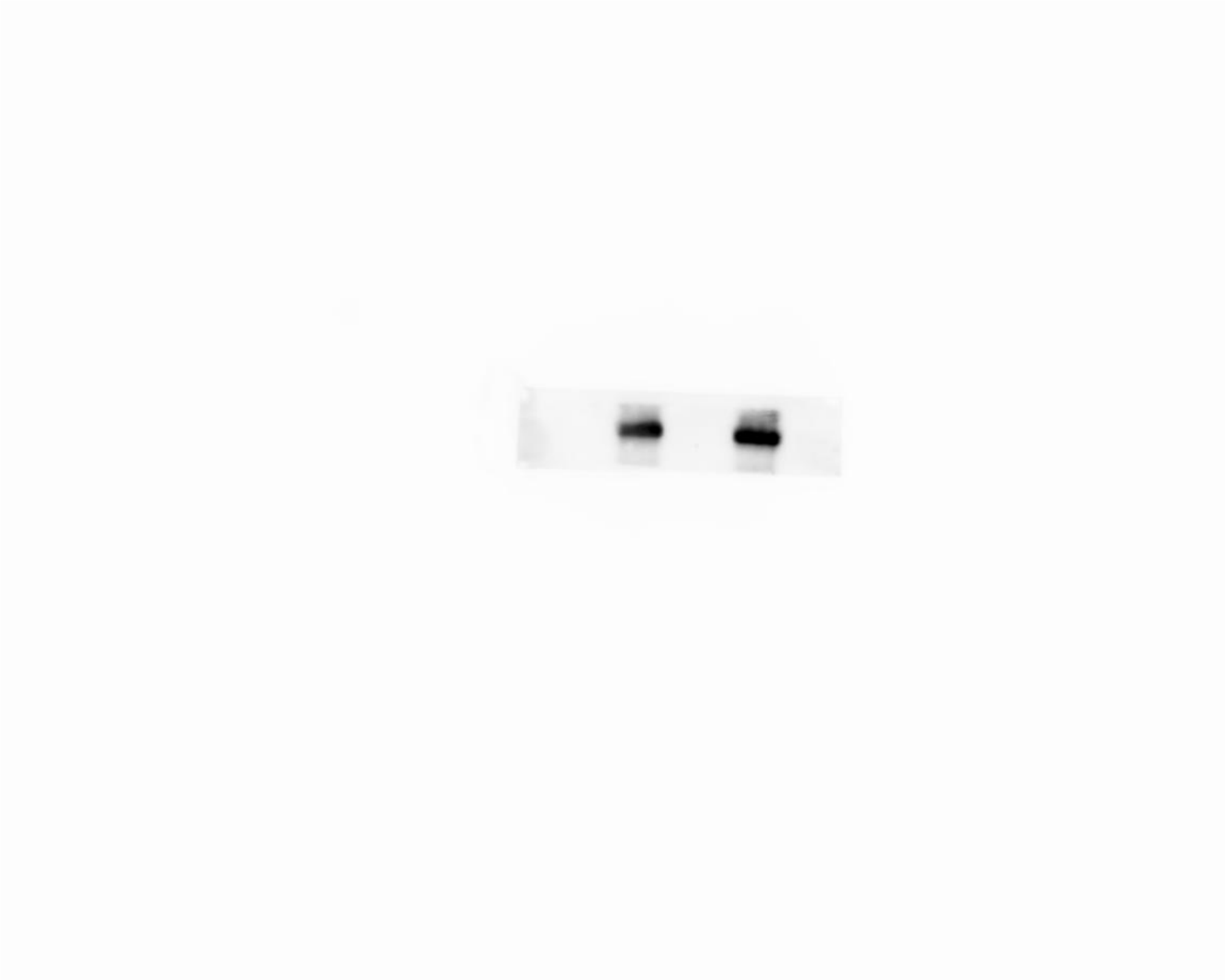

GAPDH

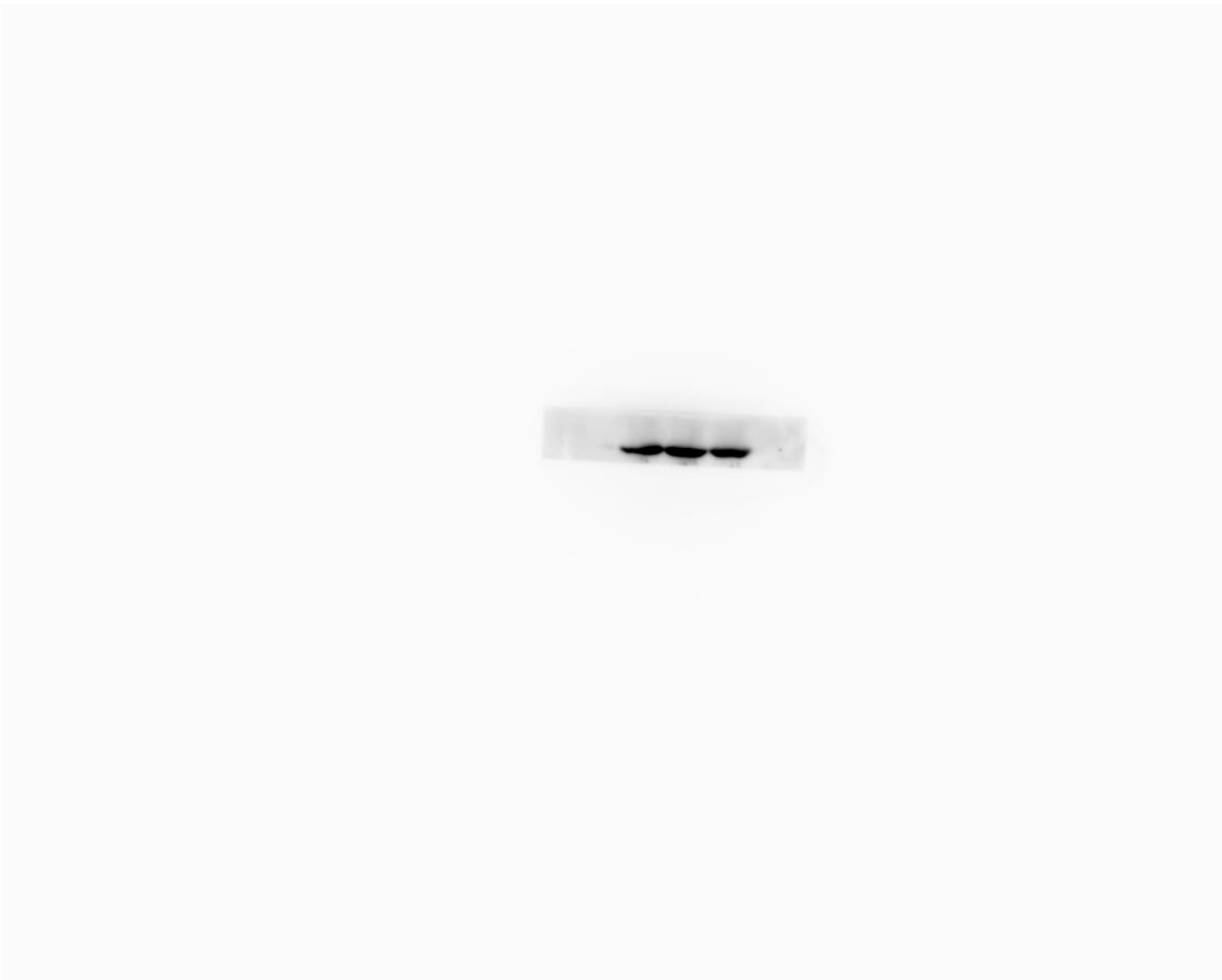

FigS5D

Flag-IP

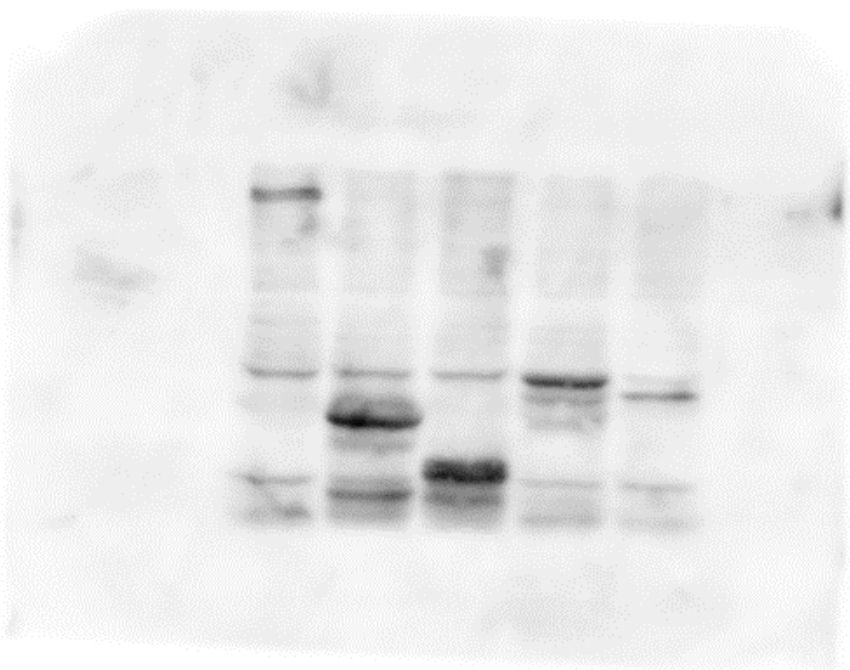

GFP-IP

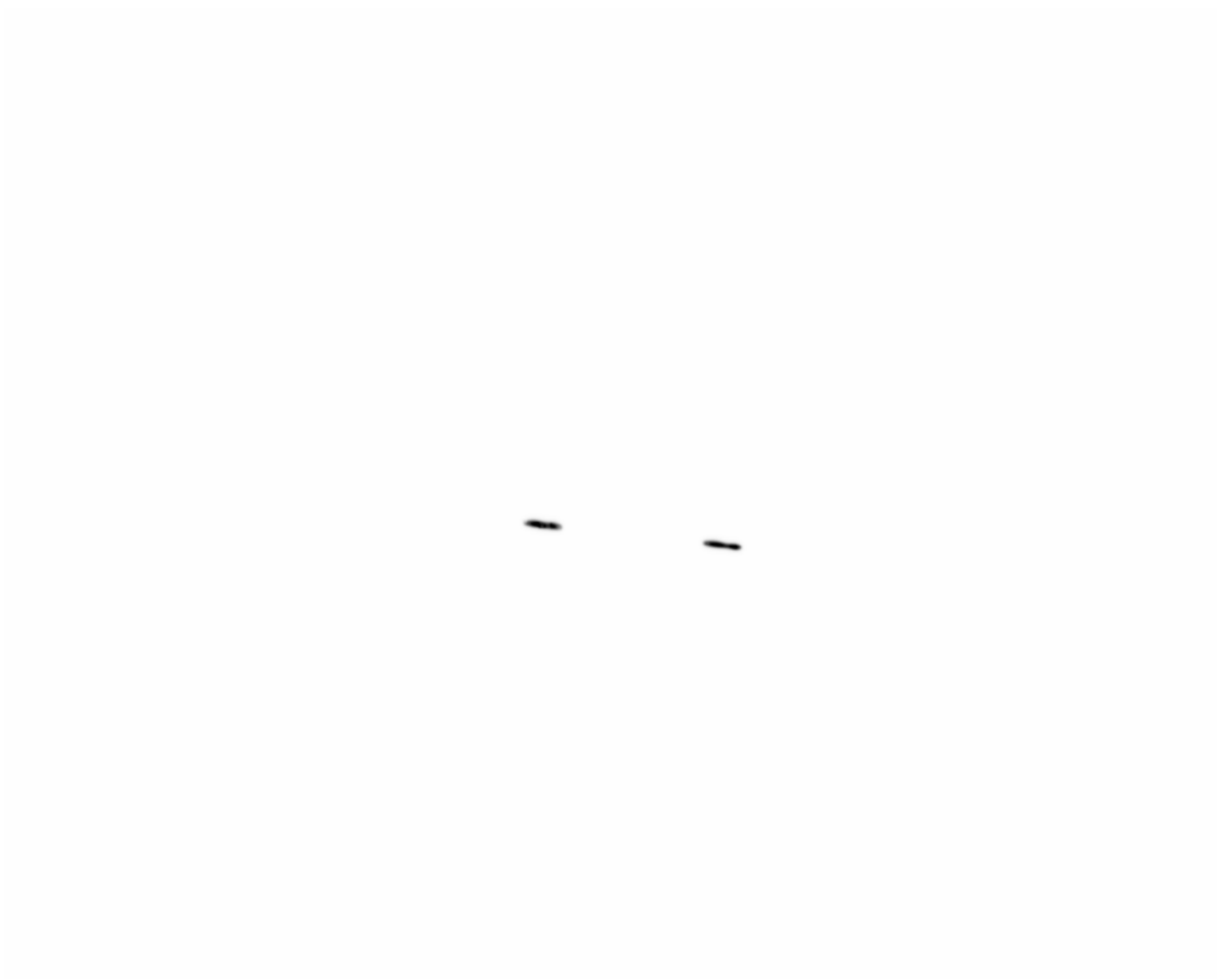

Flag-Input

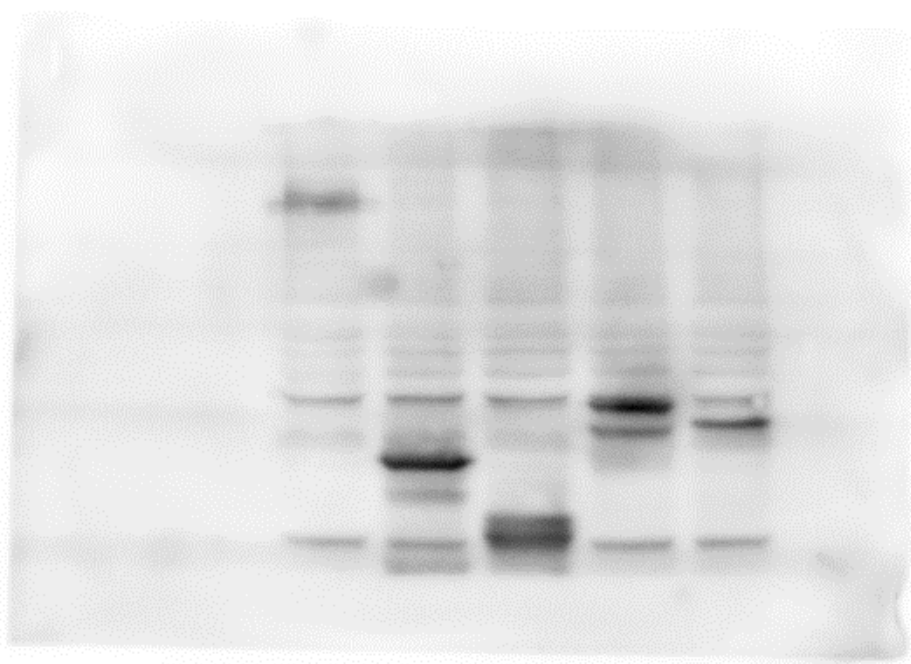

GFP-Input

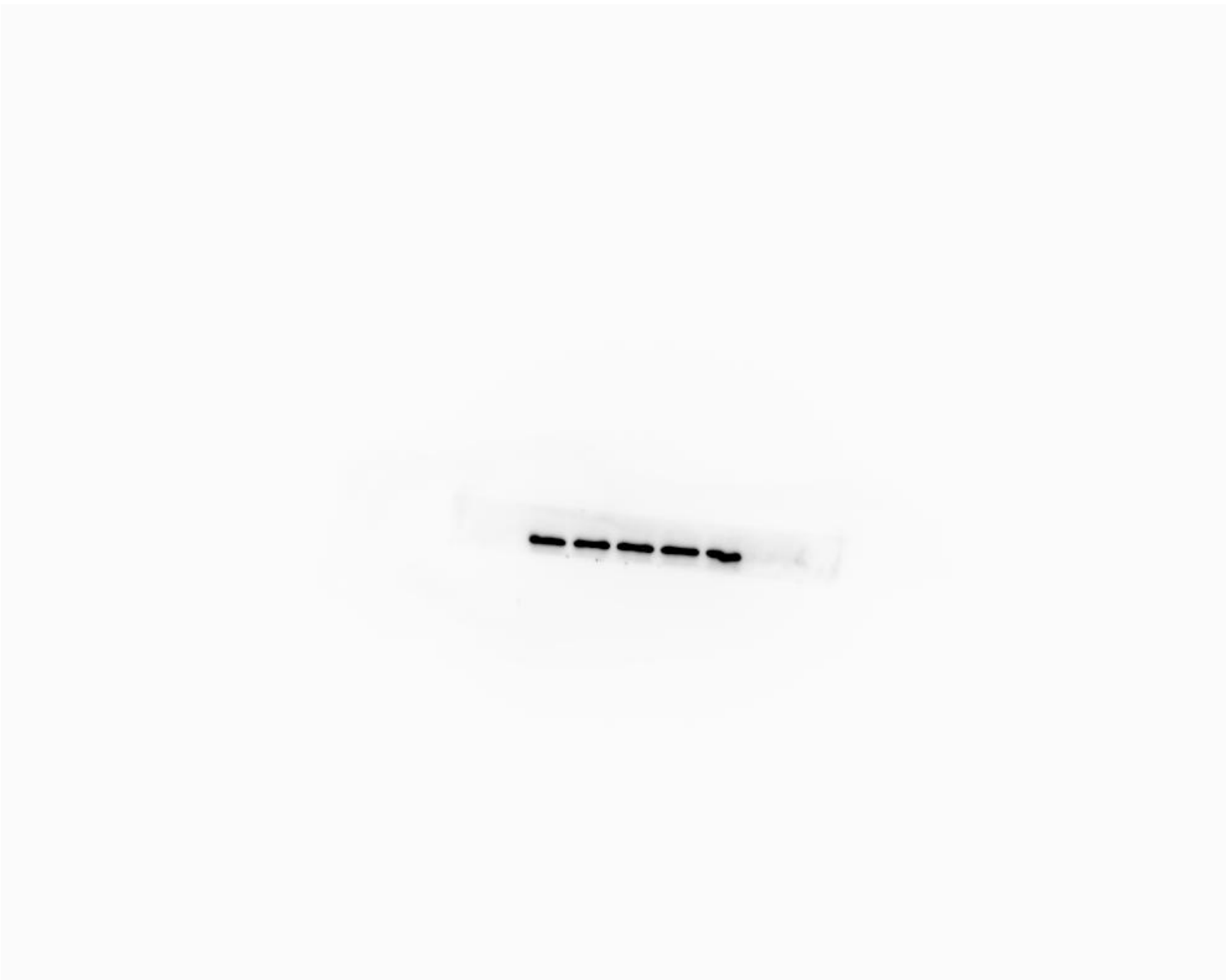

GAPDH

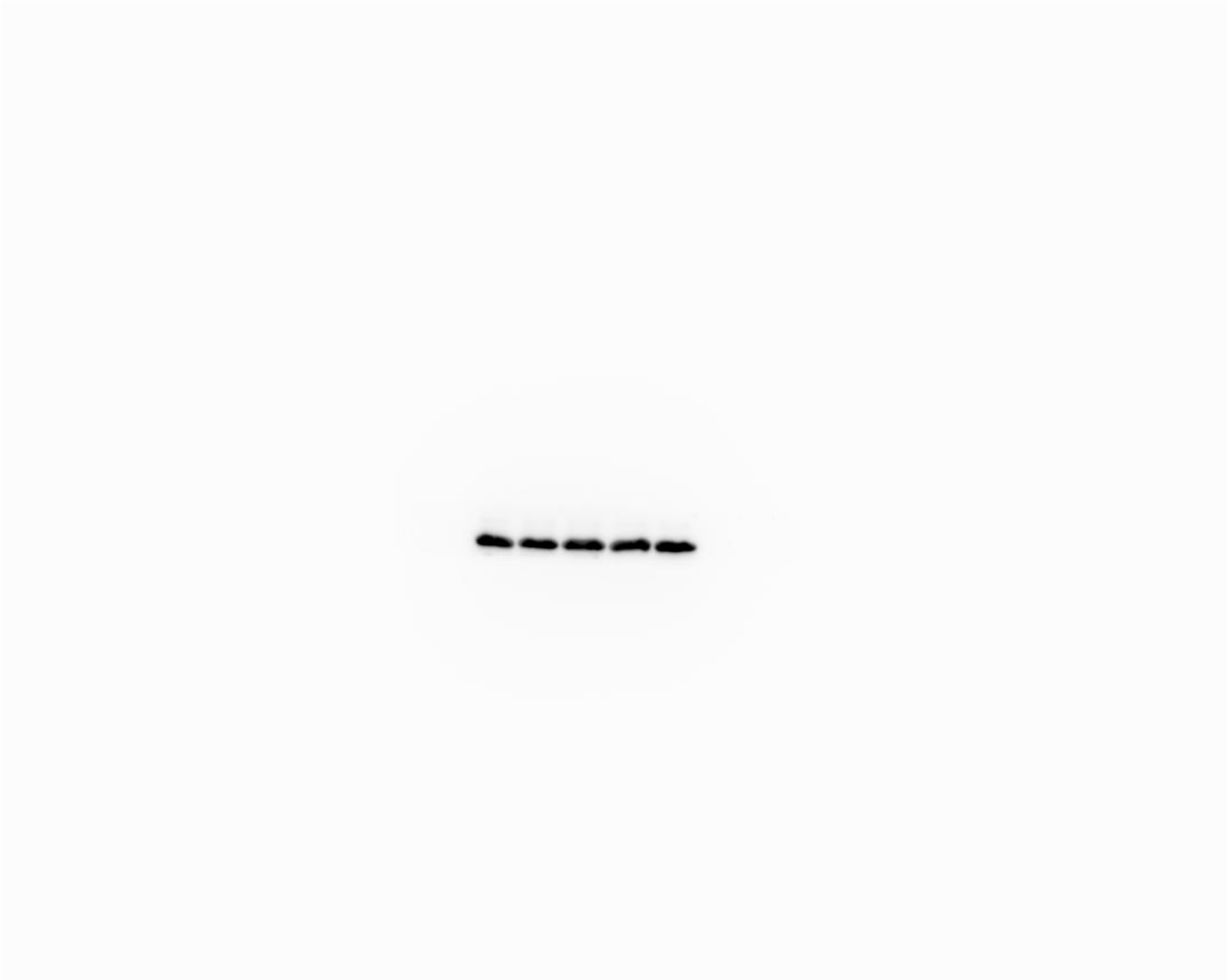

FigS5E

GFP-IP

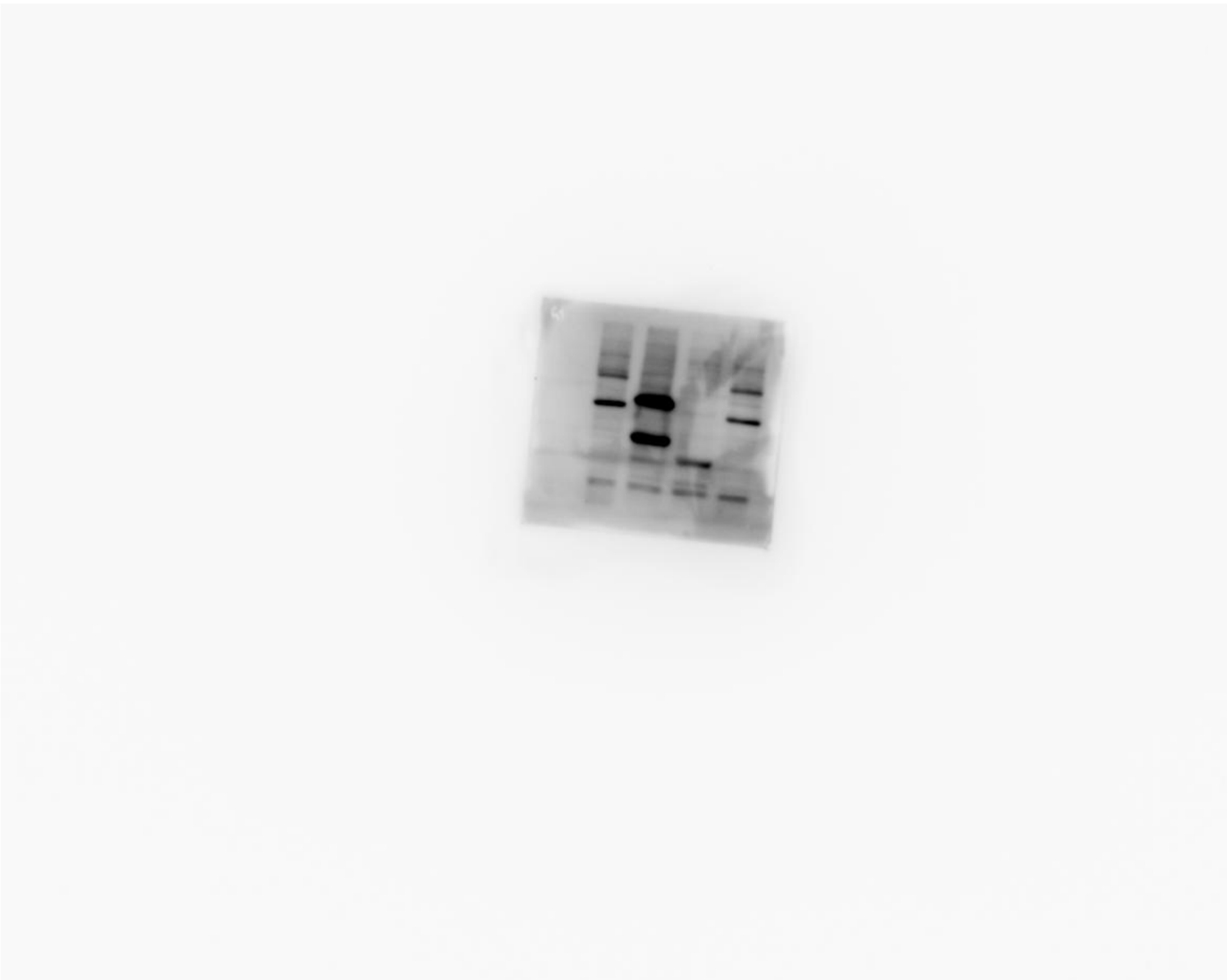

Flag-IP

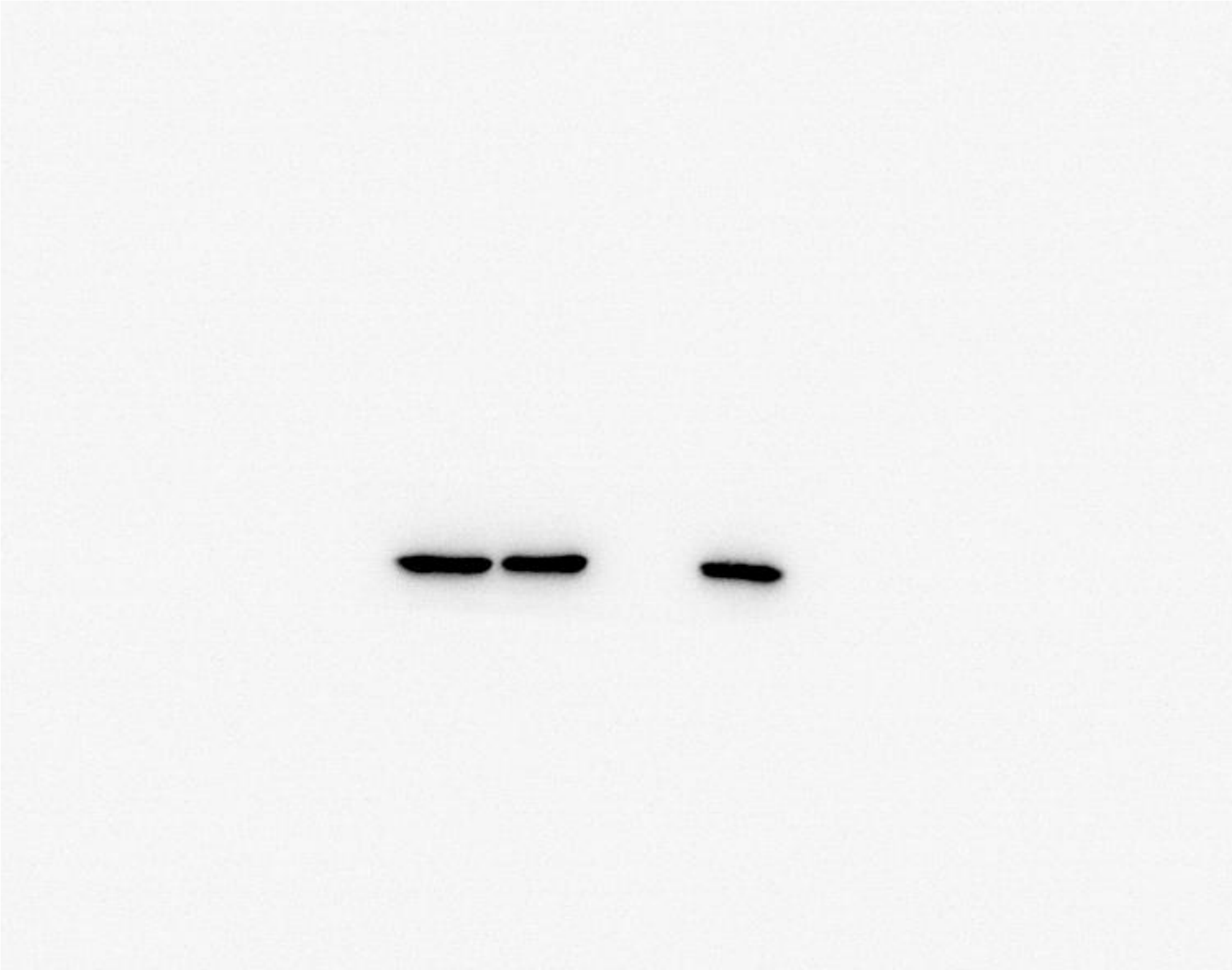

GFP-Input

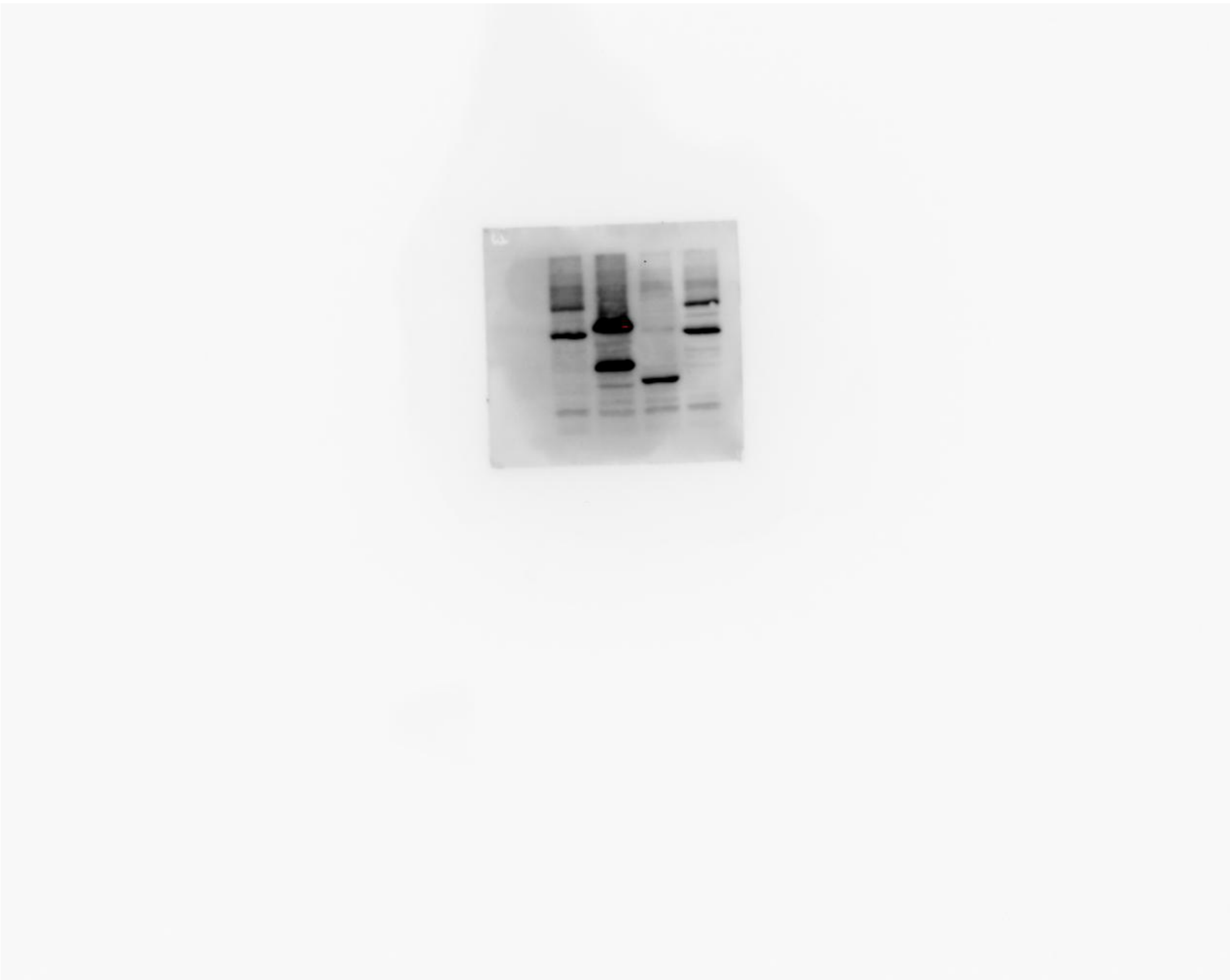

Flag-Input

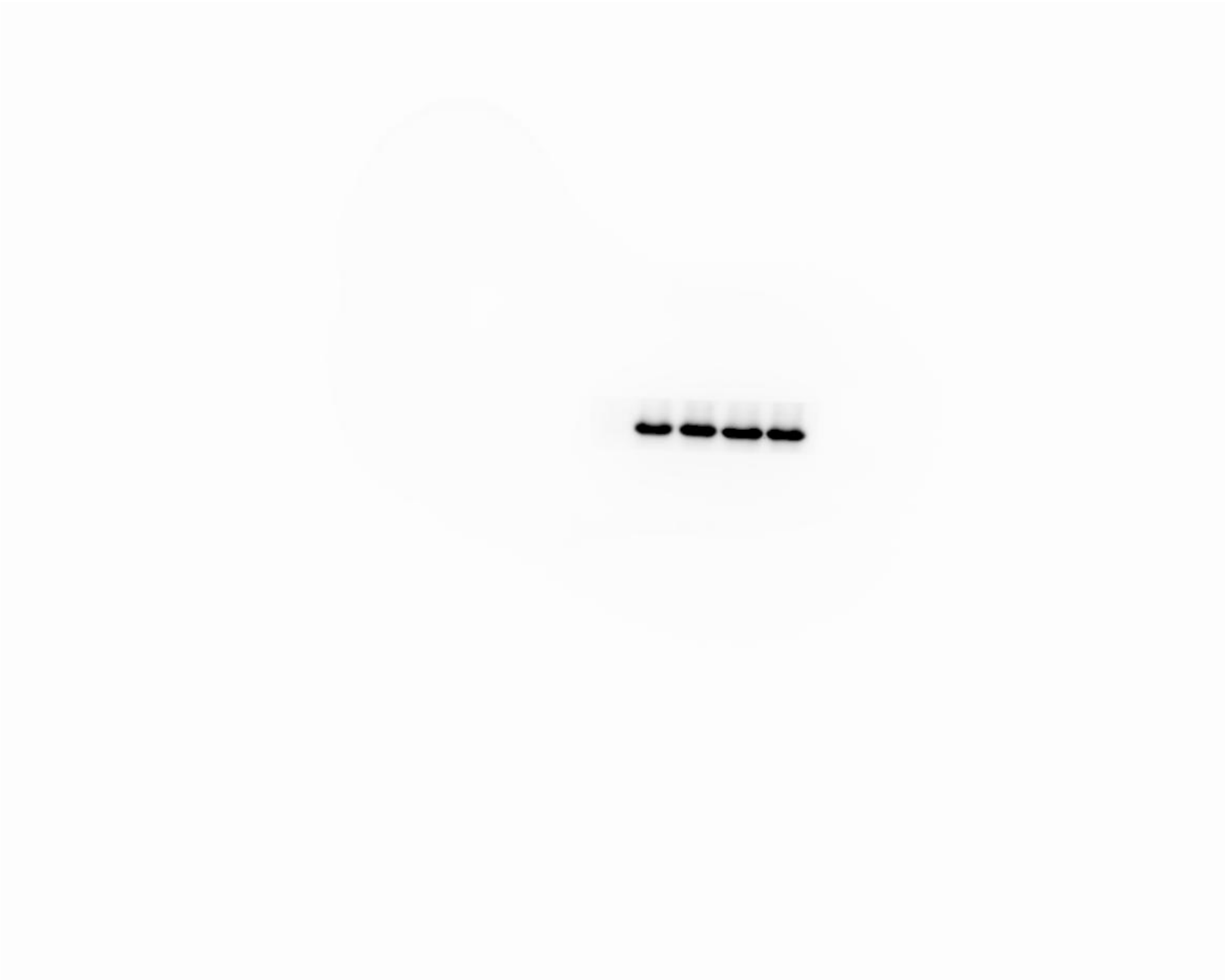

GAPDH

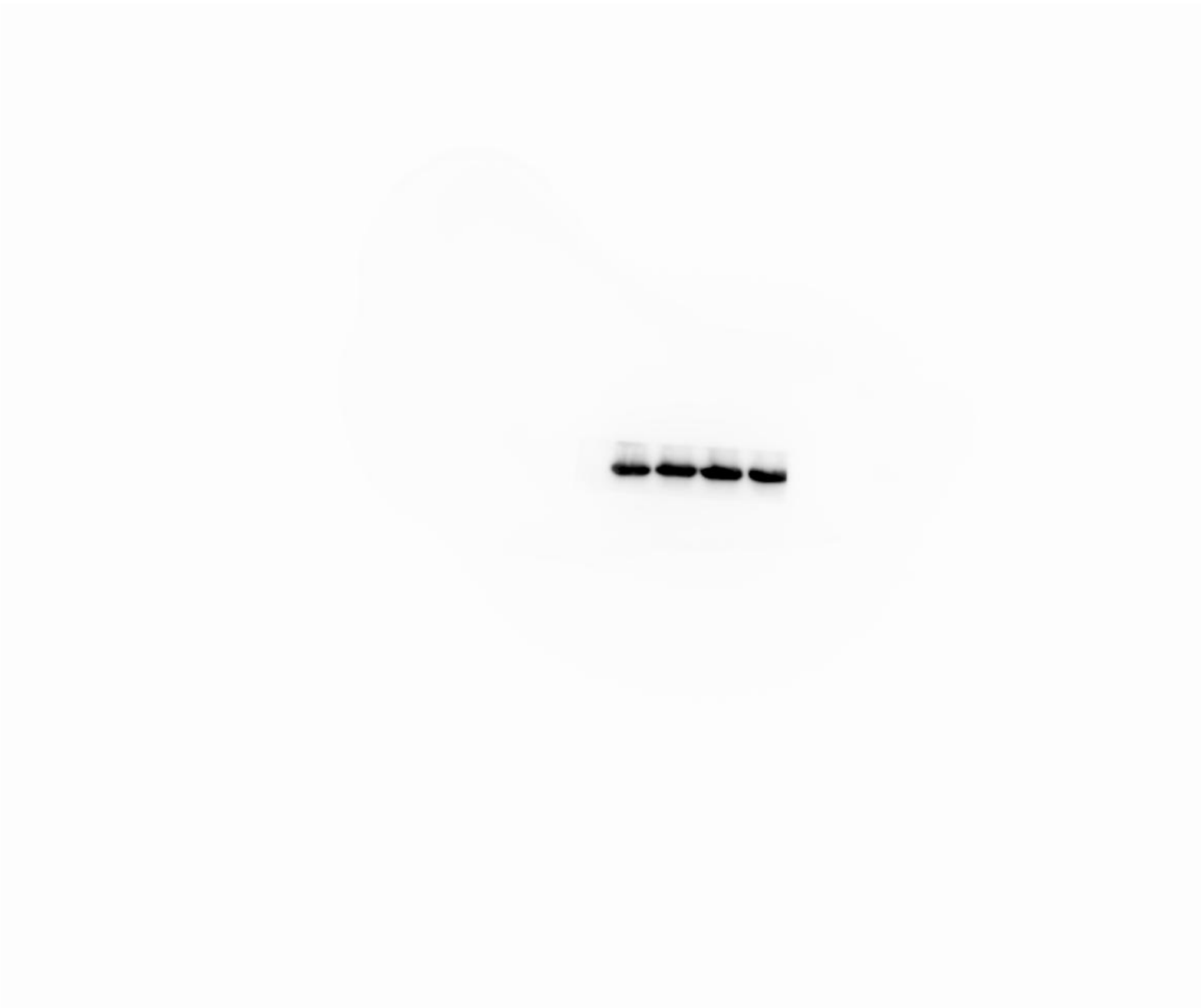

FigS5F

MED12

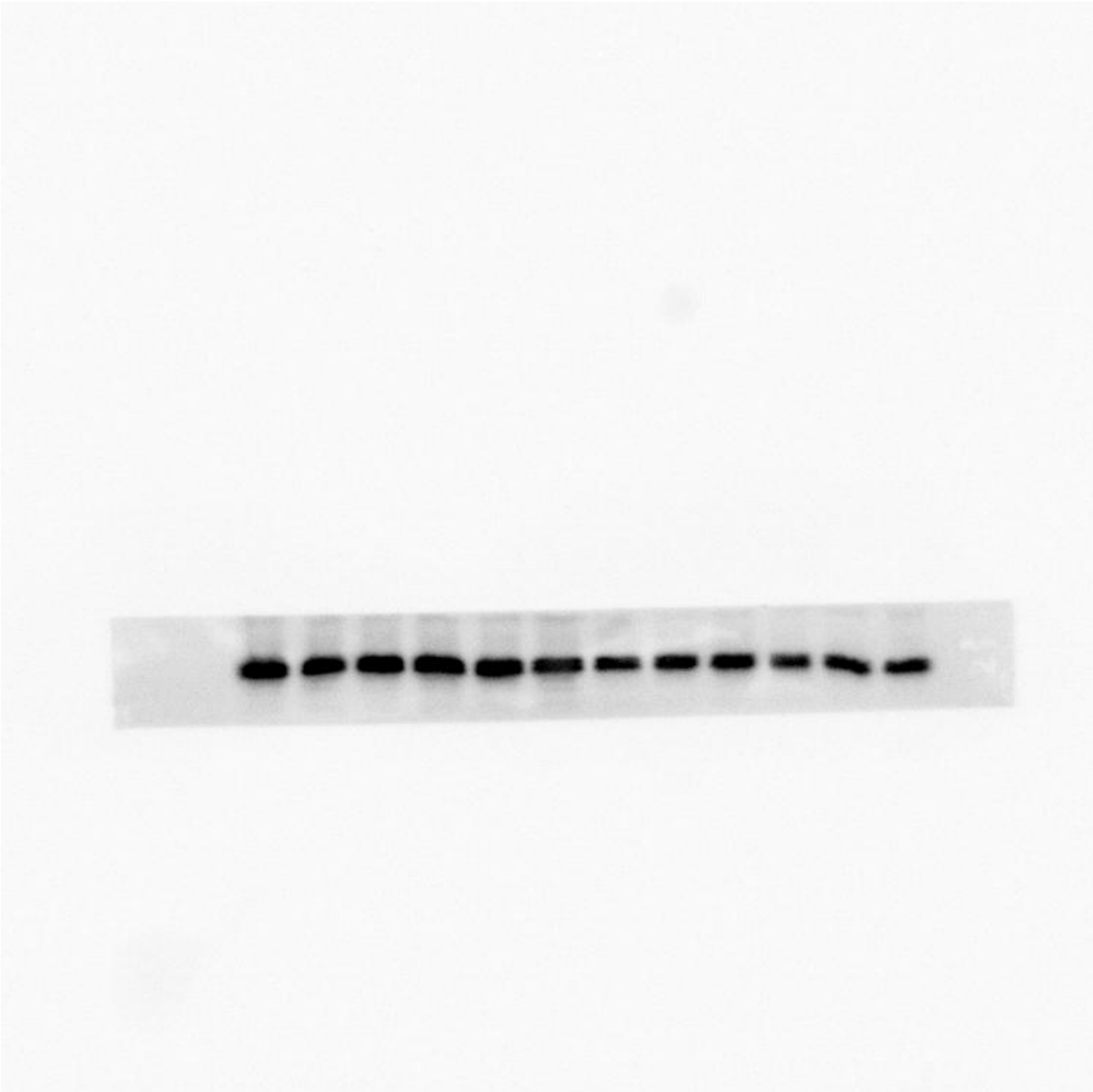

γH2A

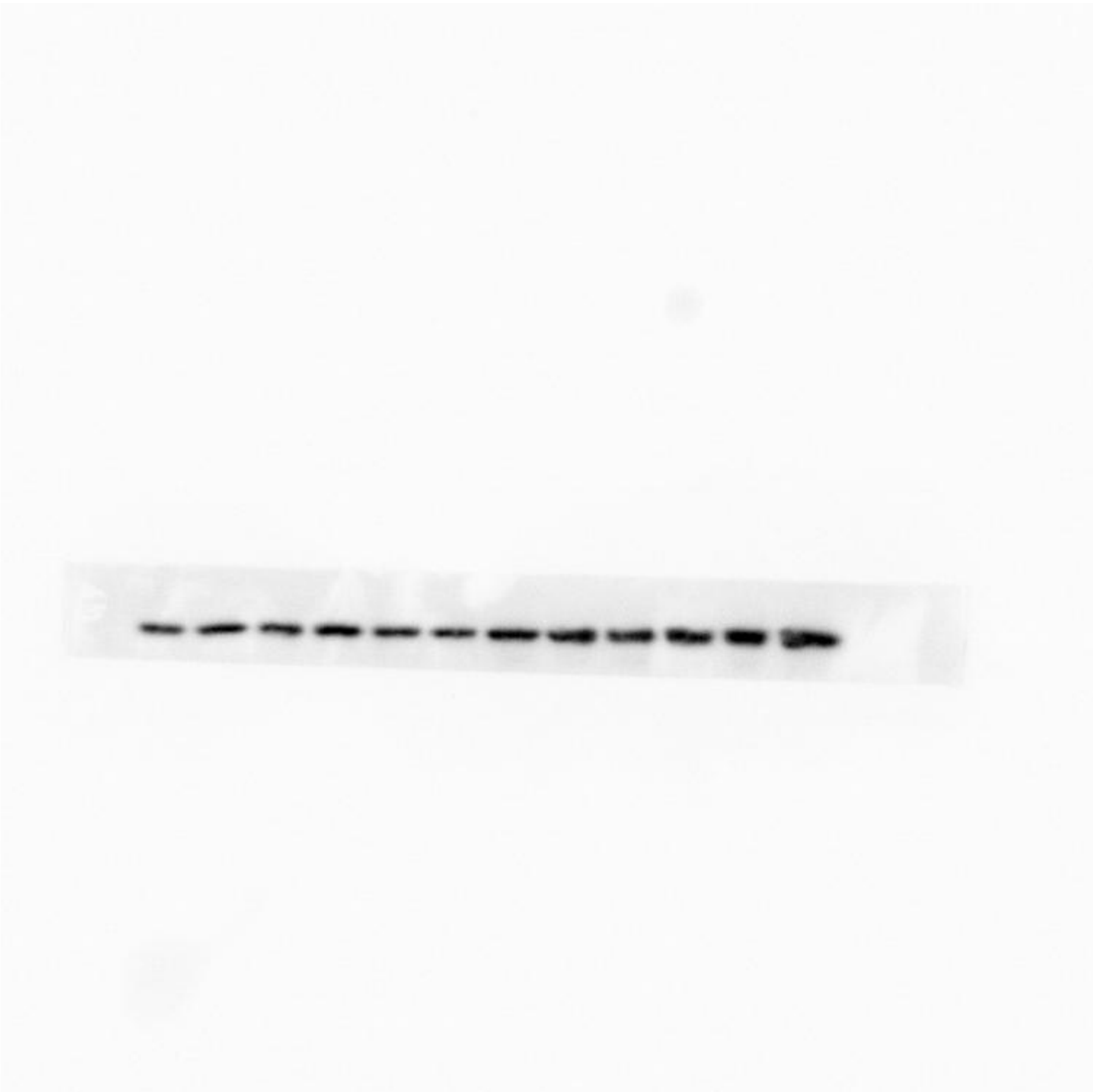

P21

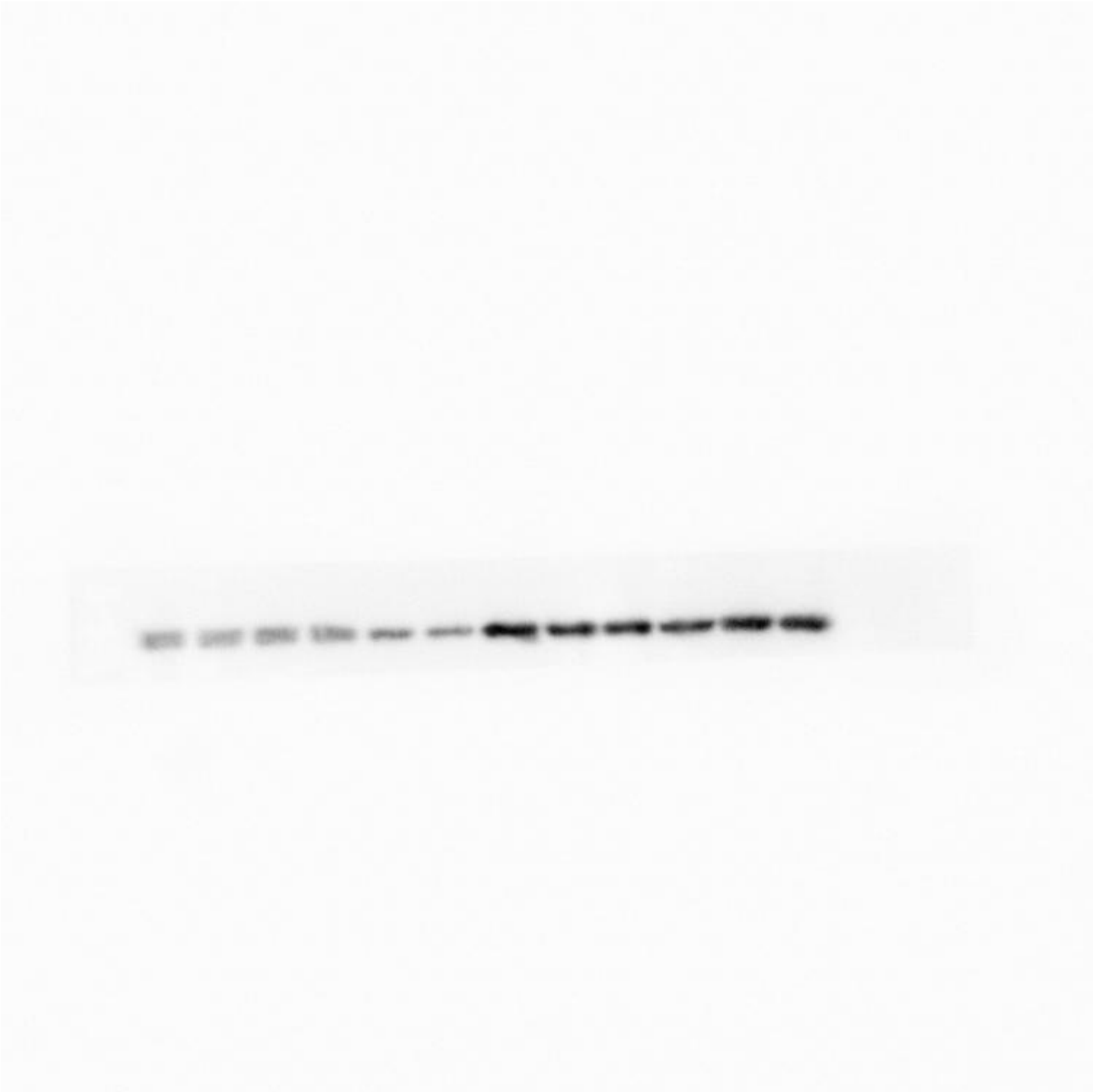

GAPDH

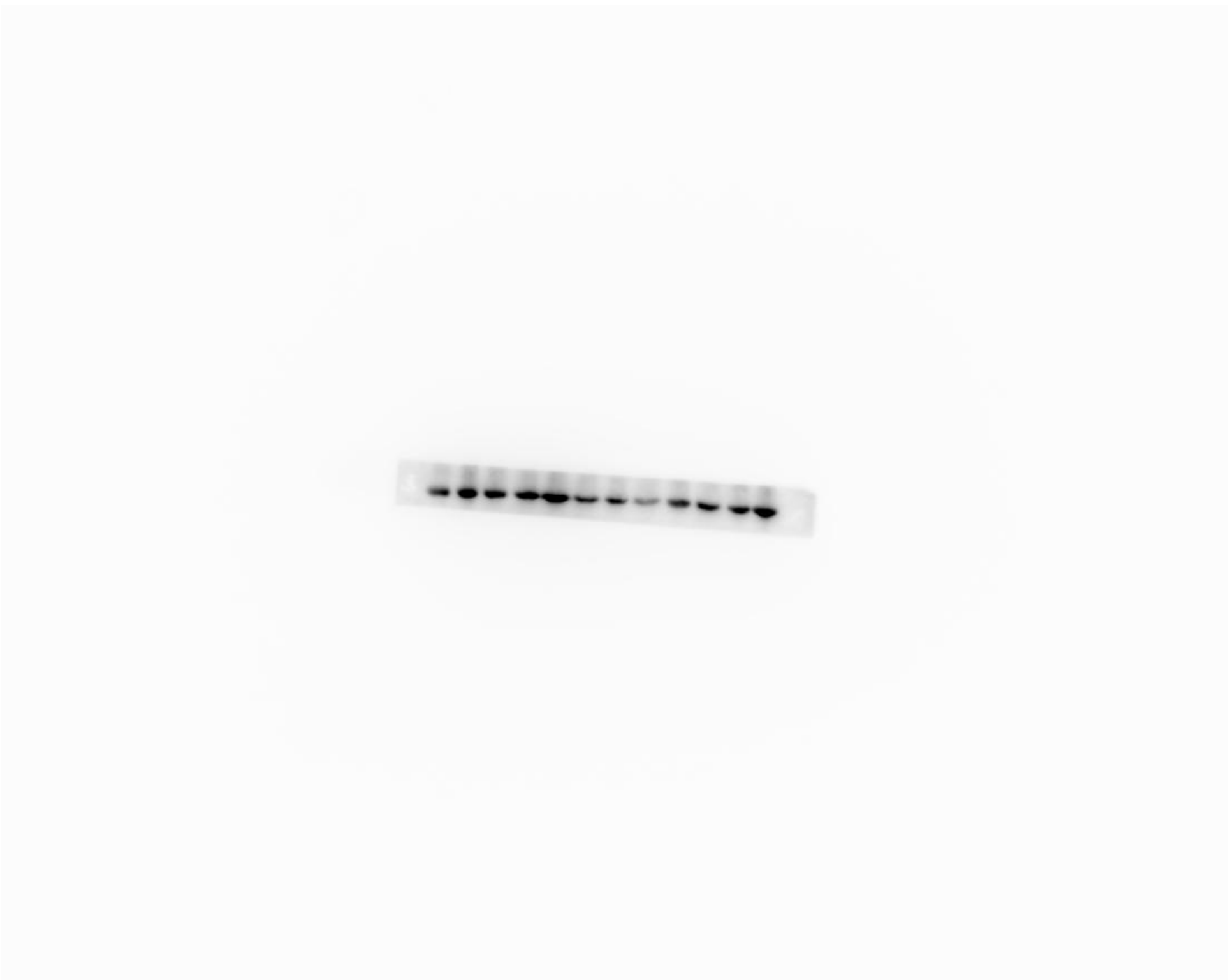

FigS5H

WT-MED12

WT-GAPDH

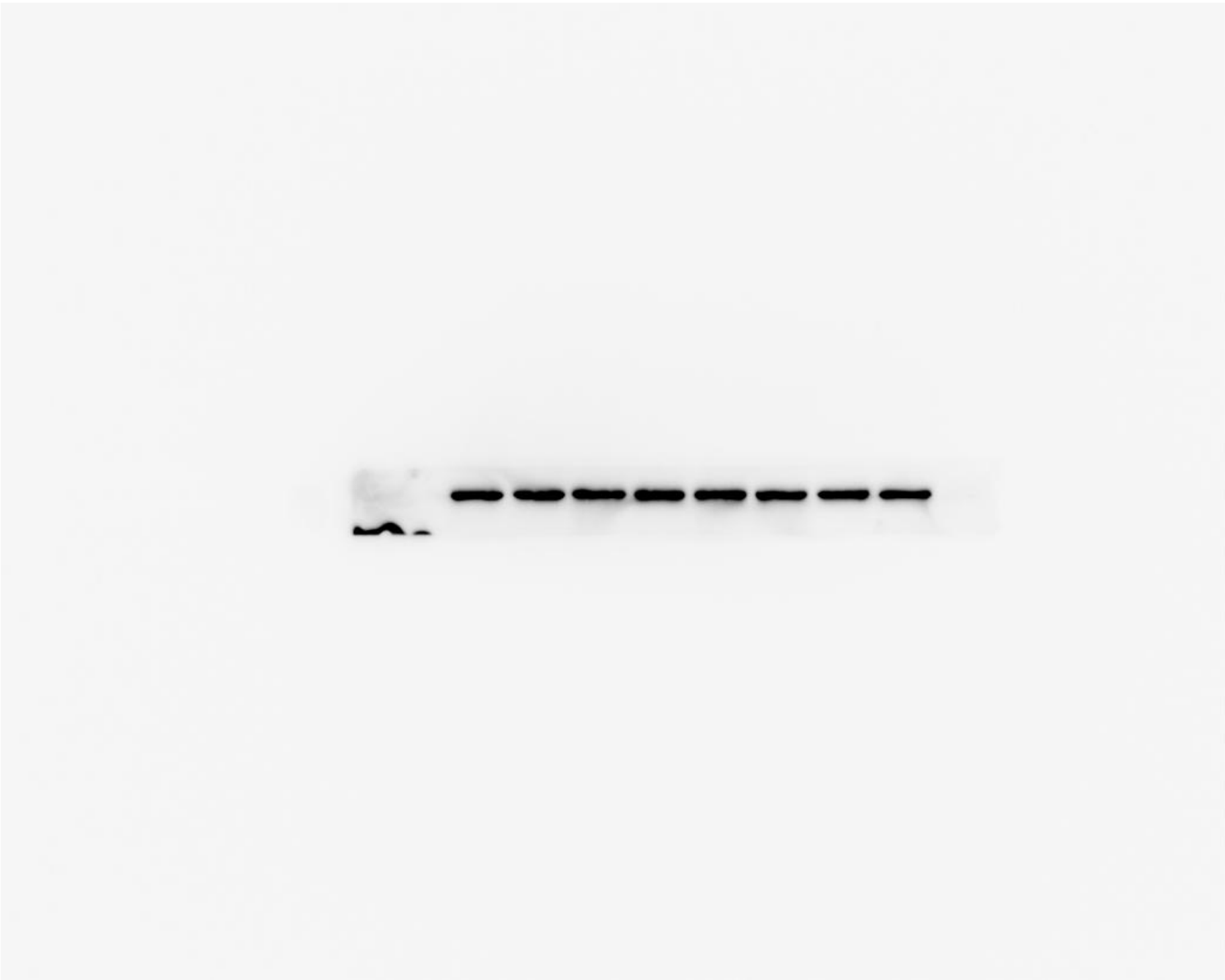

RF-MED12

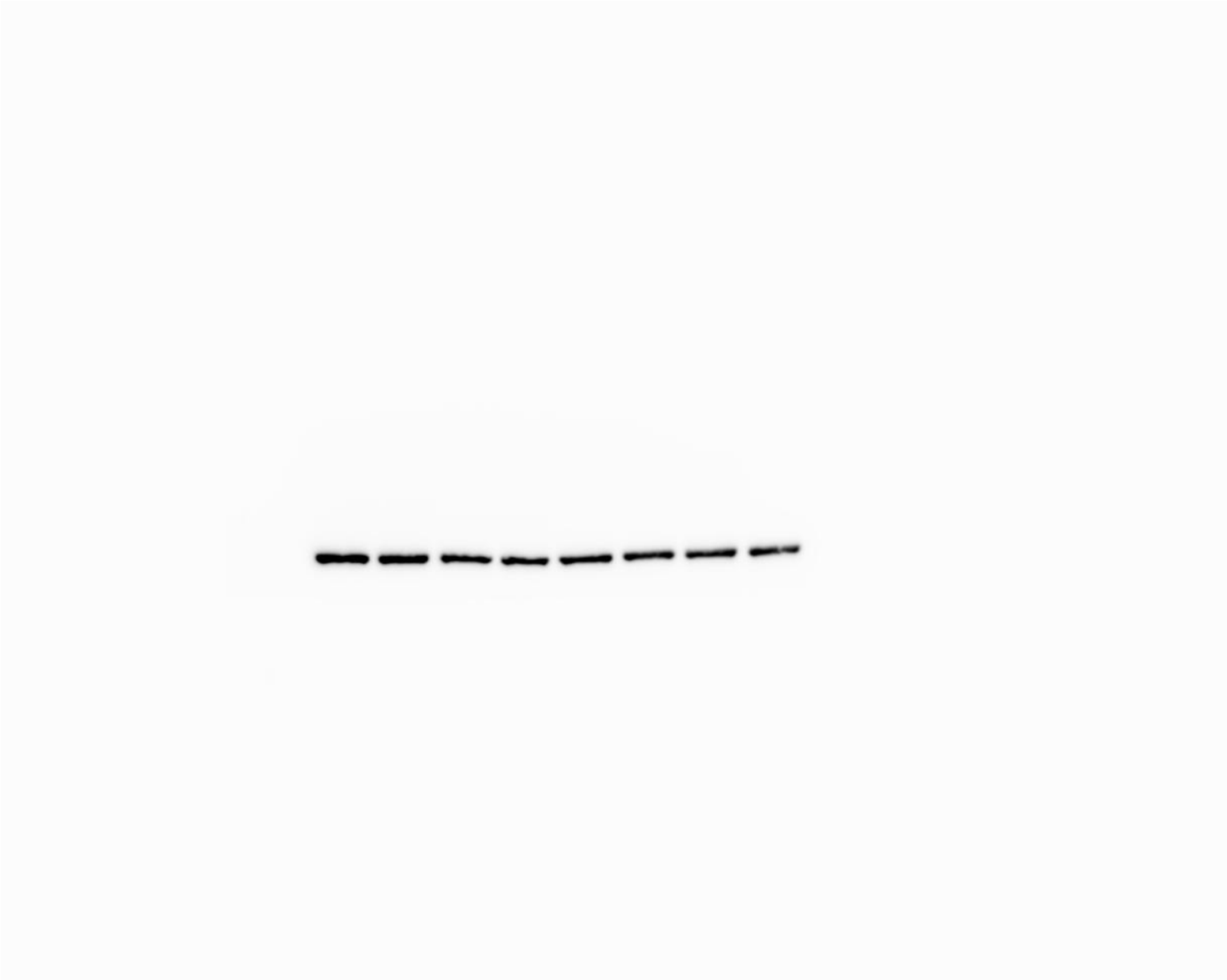

RF-GAPDH

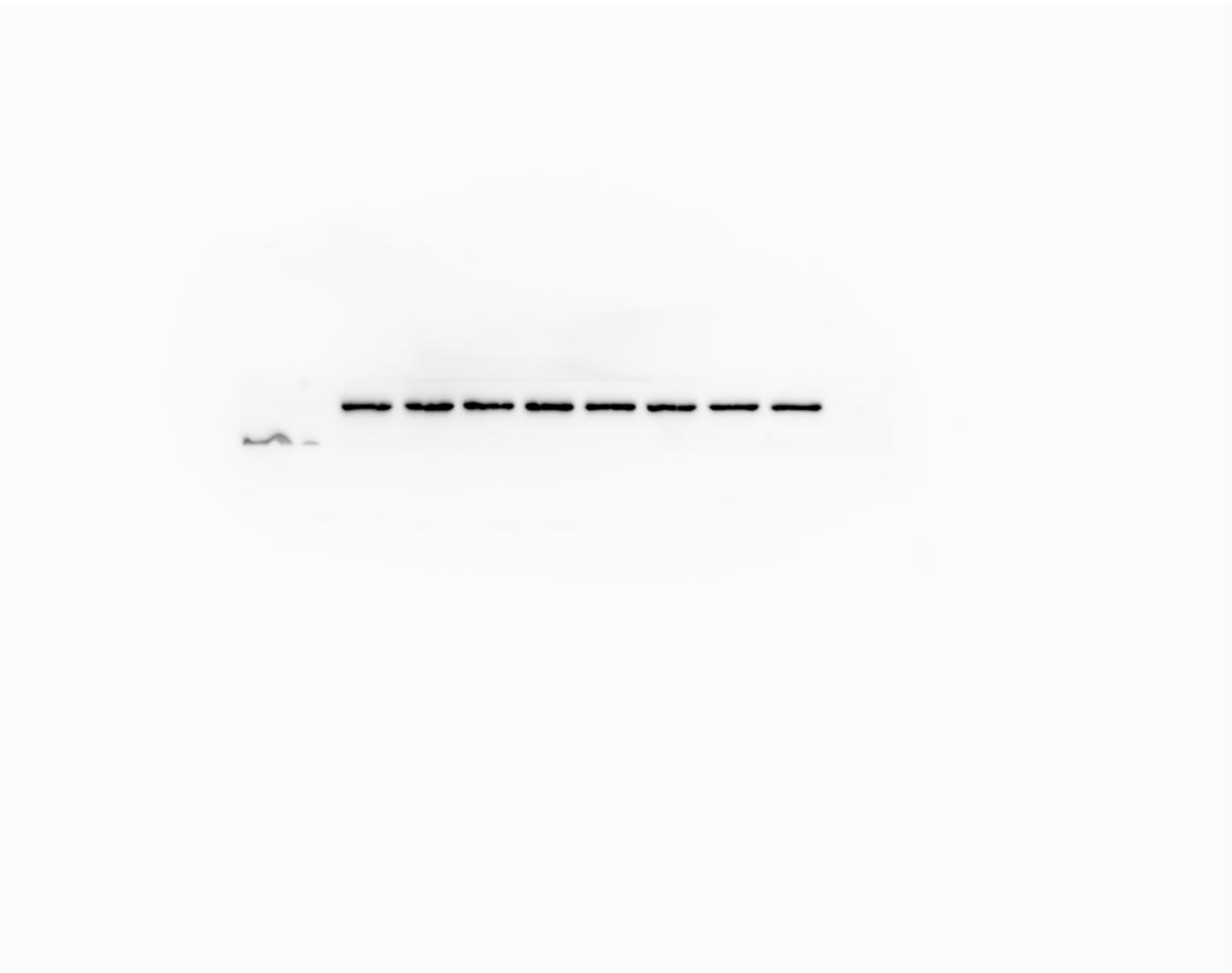

FigS7A

MED12

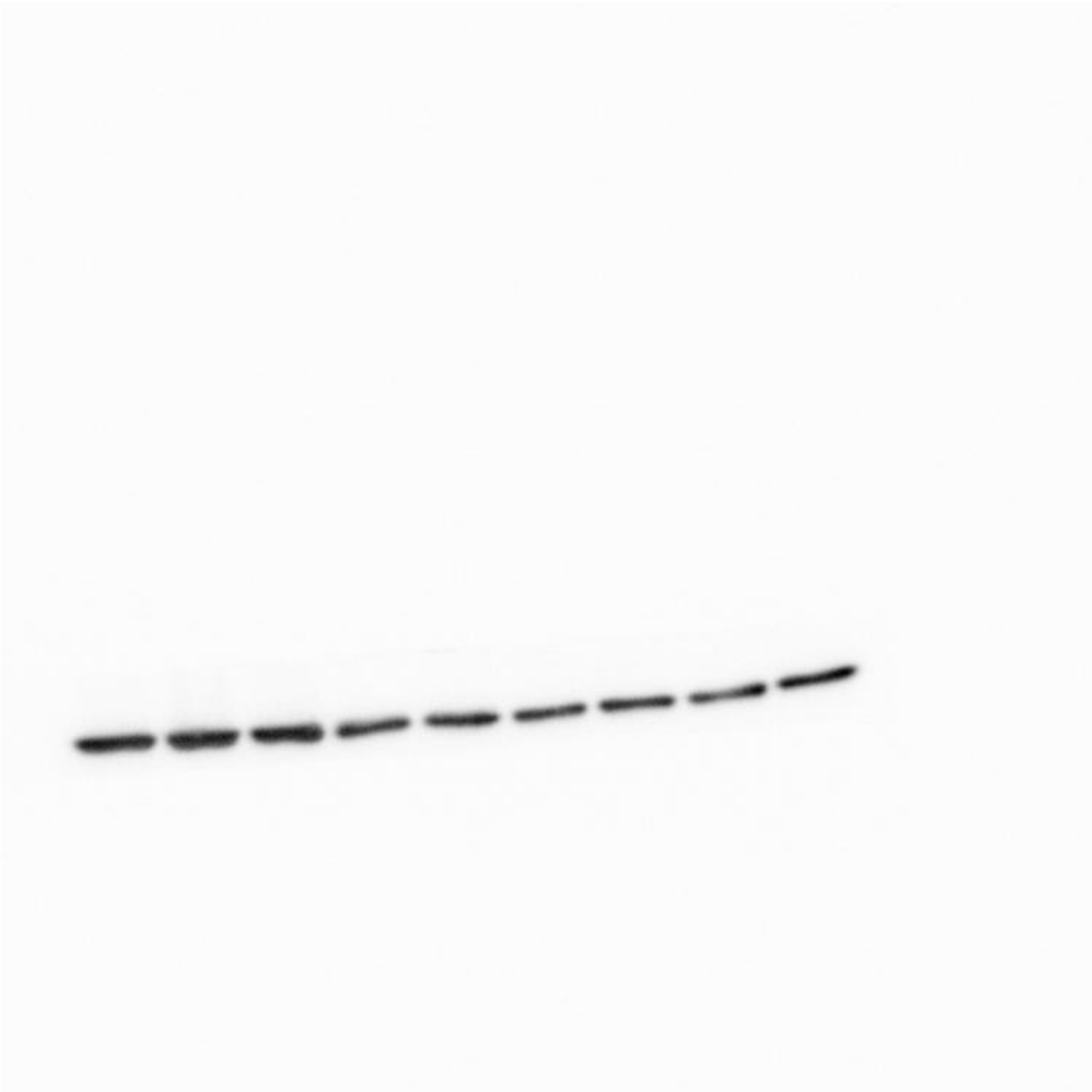

GAPDH

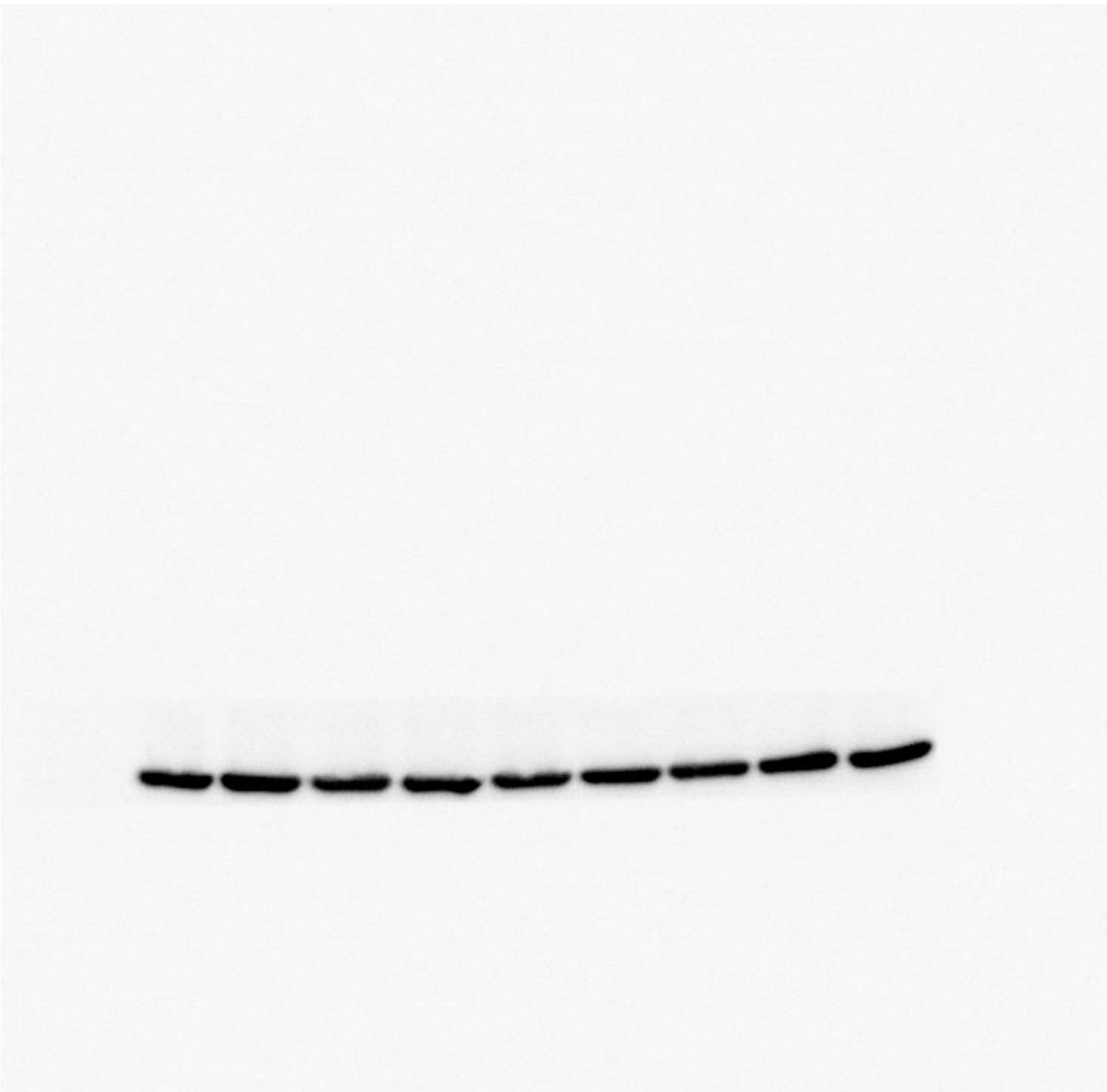

FigS7C

PP53

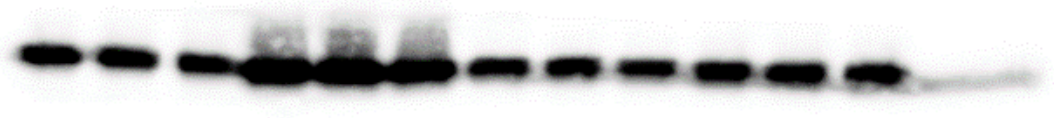

P53

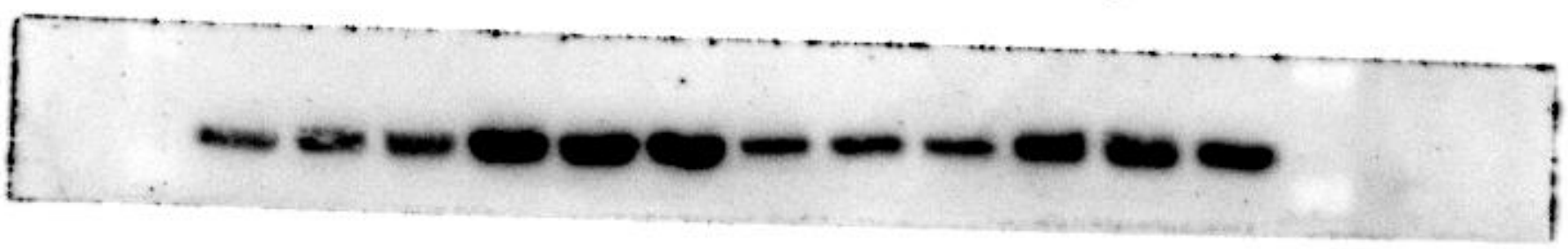

P21

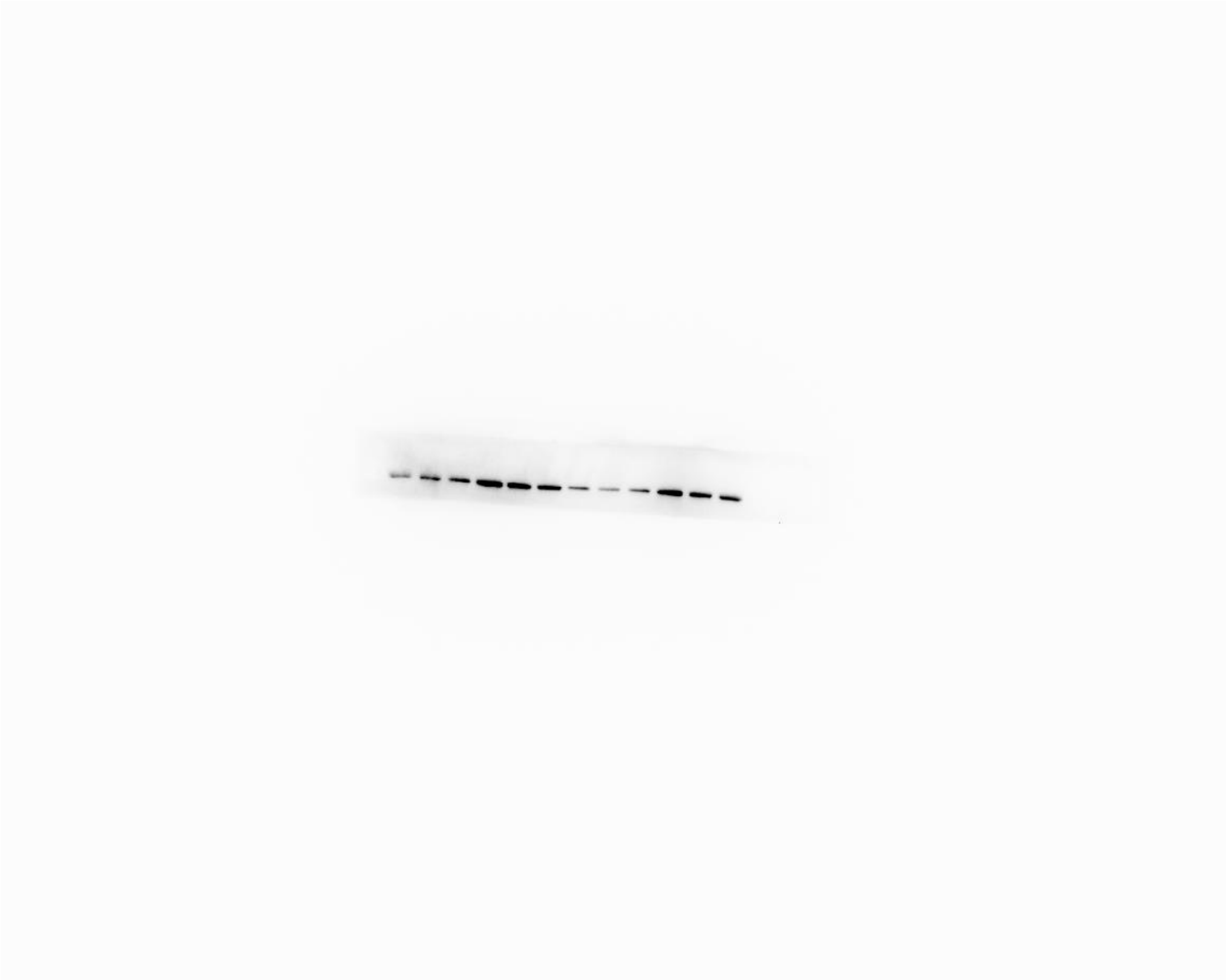

P16

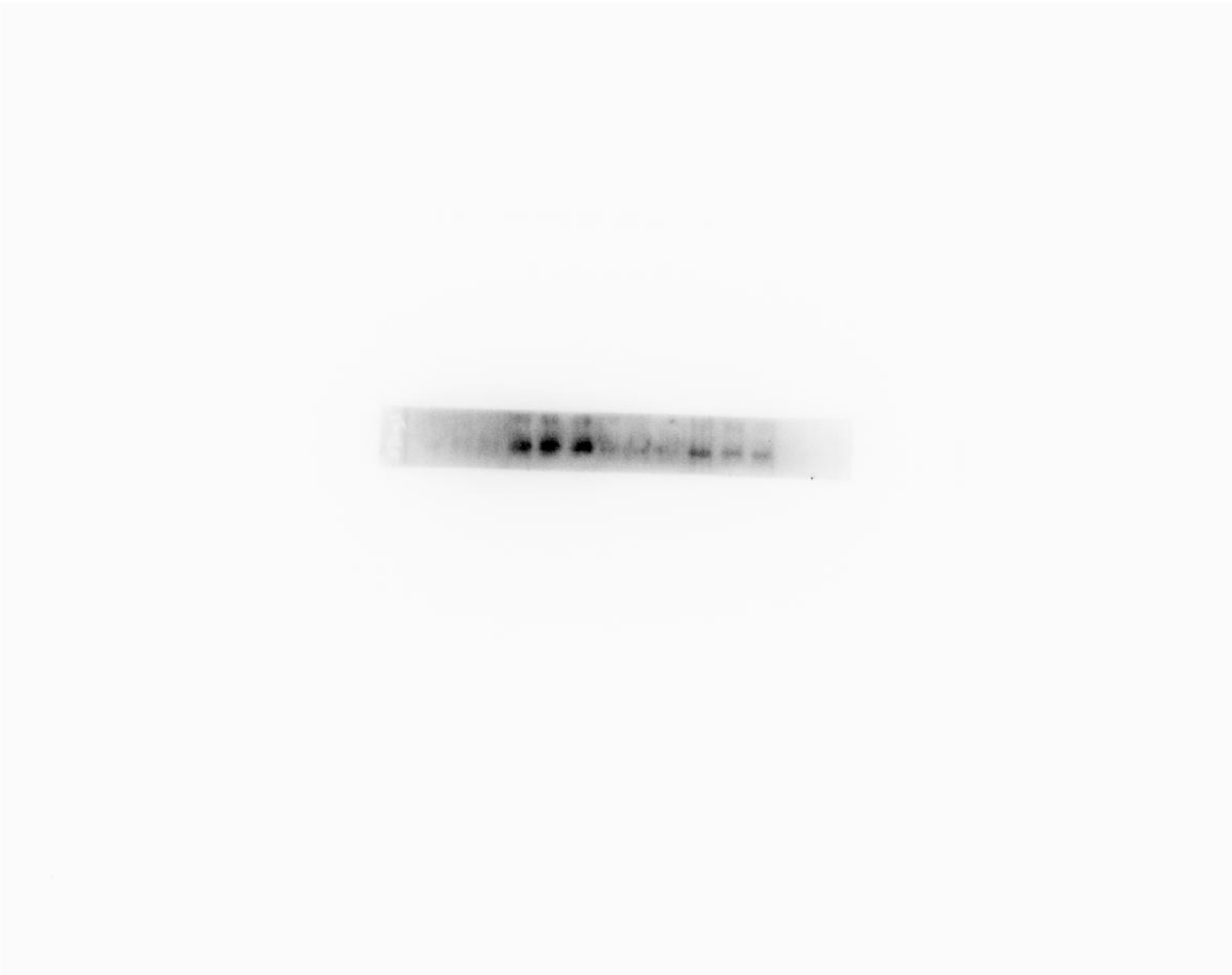

GAPDH

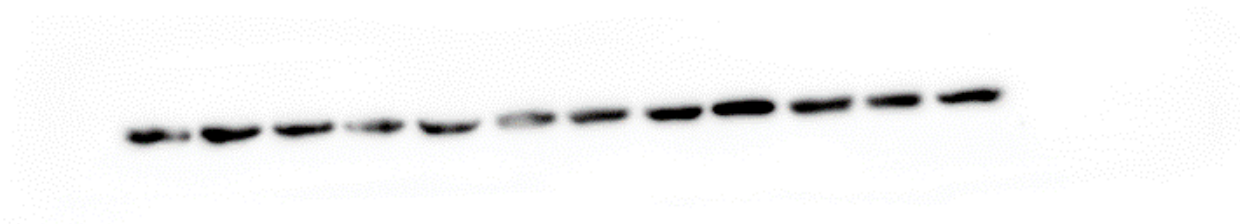

FigS8A

PP53

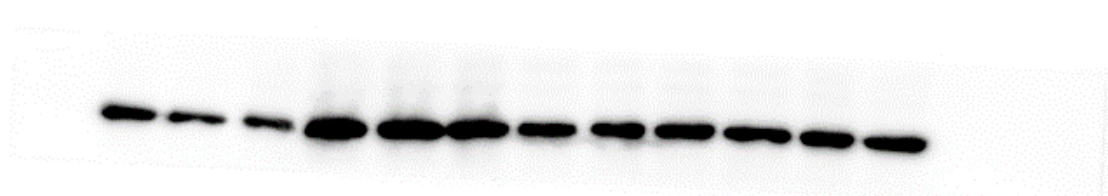

P53

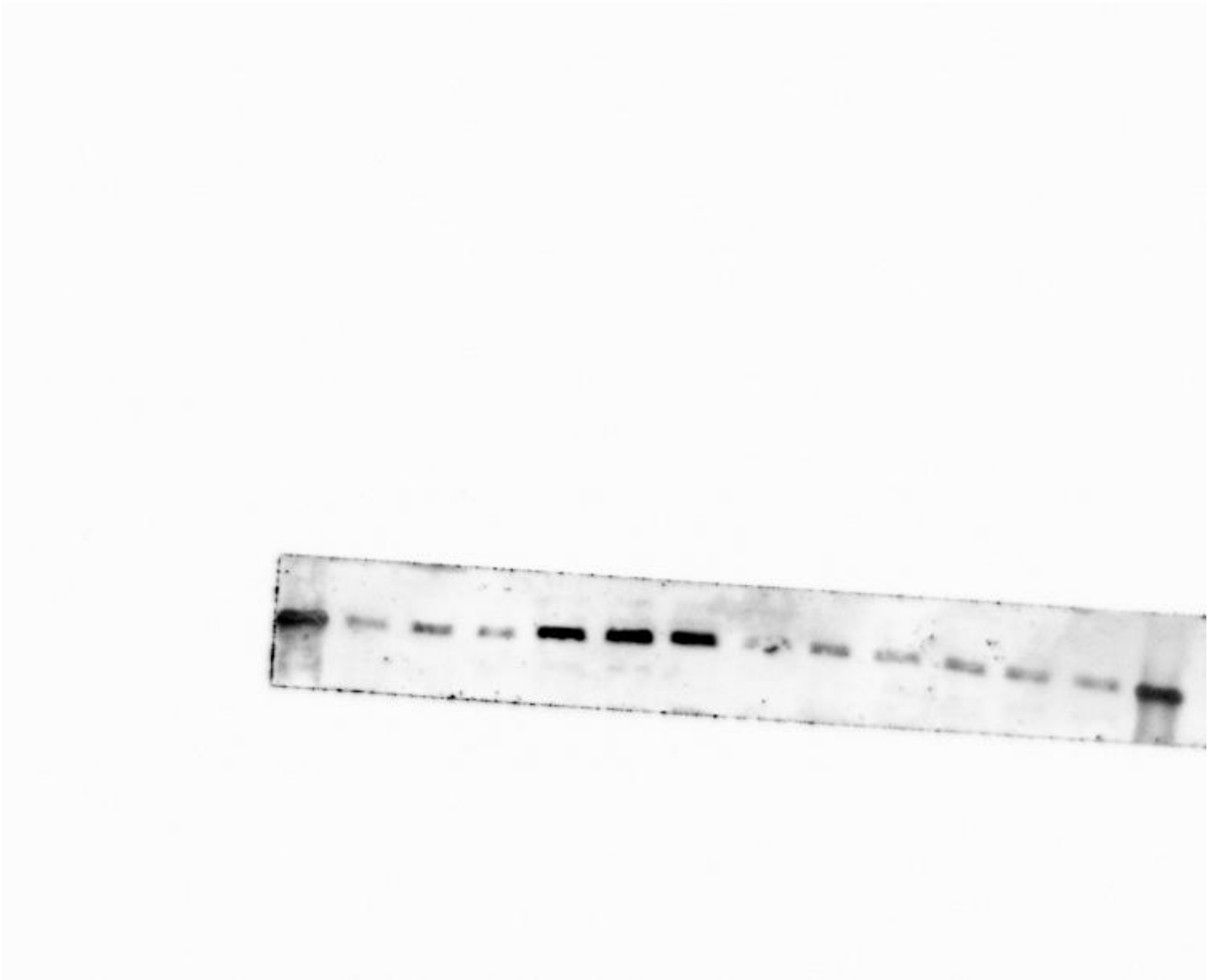

P21

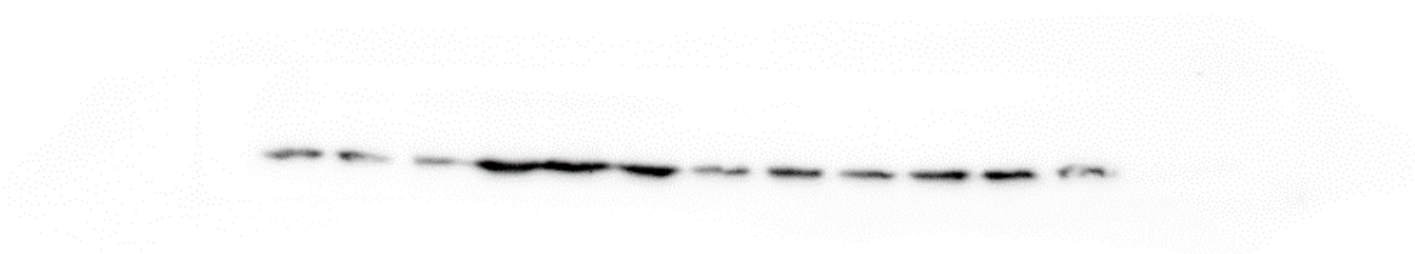

P16

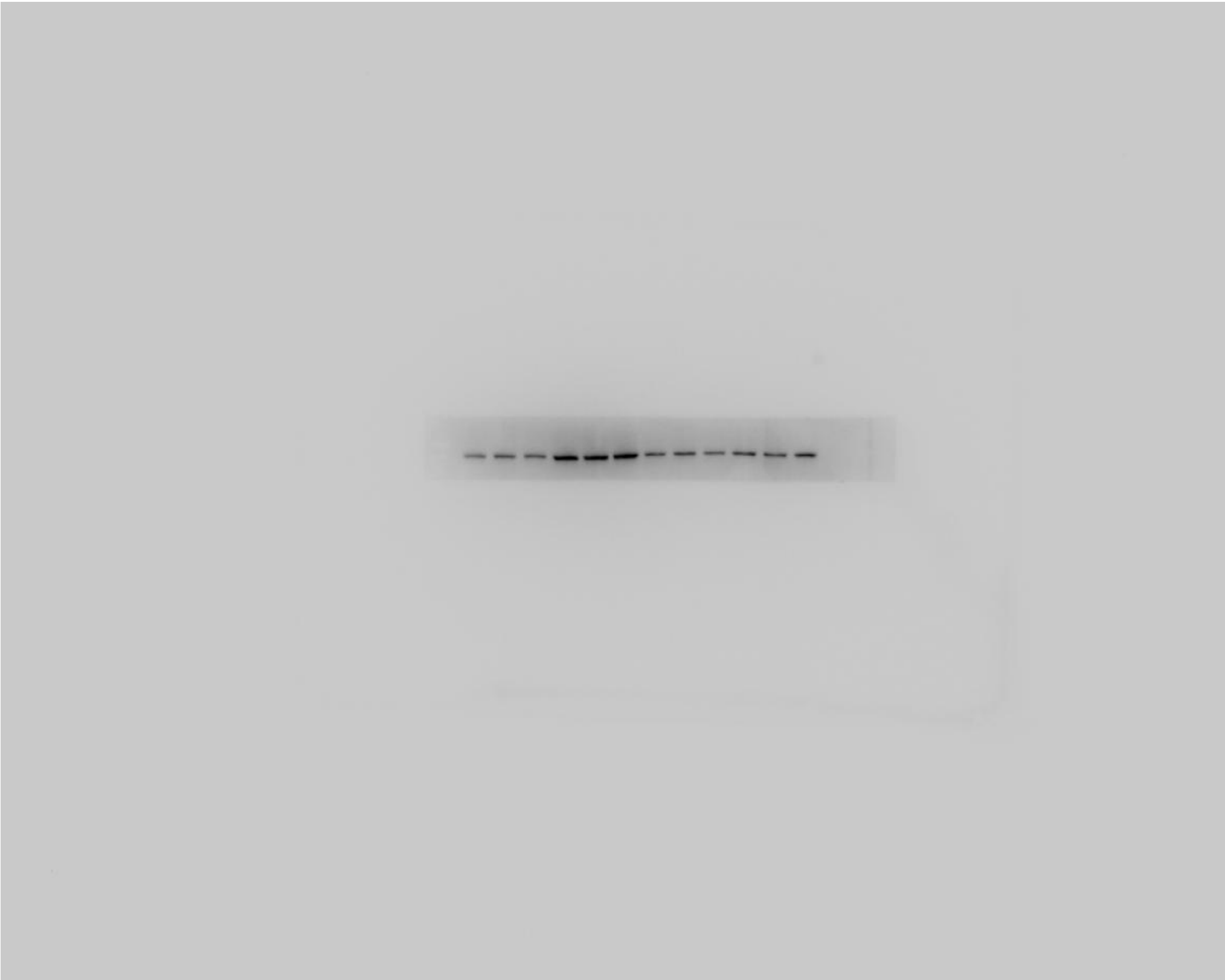

GAPDH

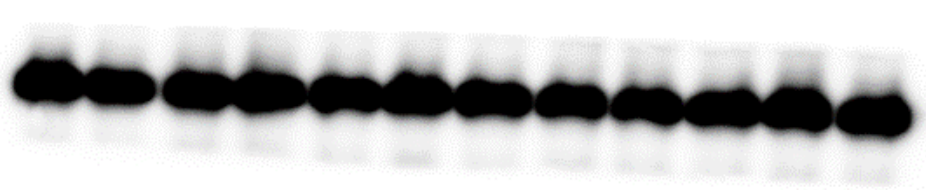

FigS8B

p-TBK1

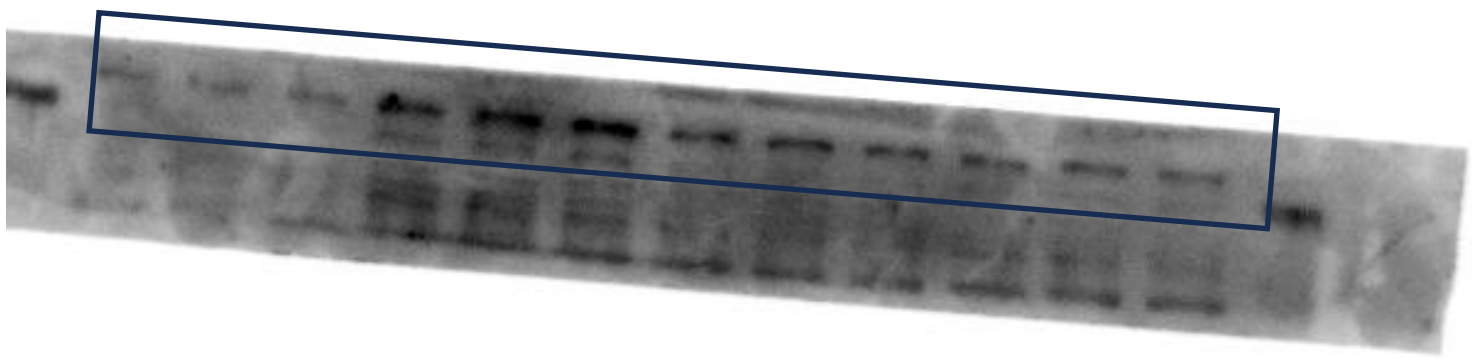

TBK1

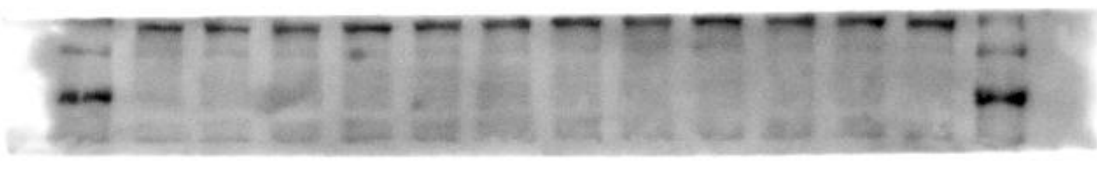

p-IRF3

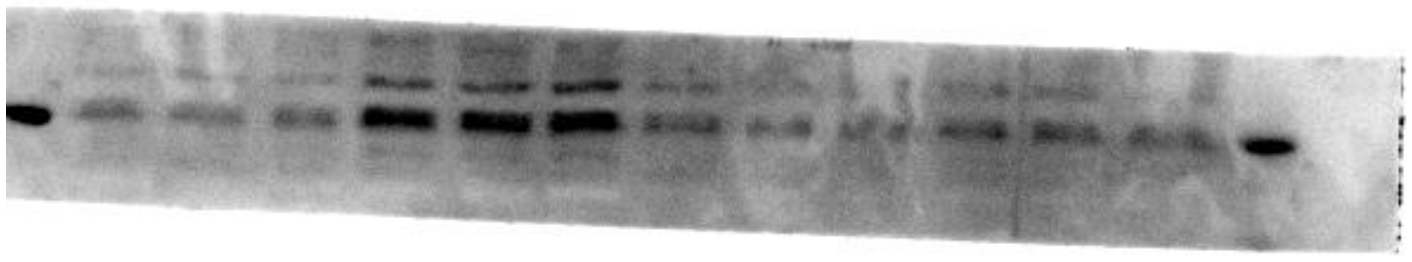

IRF3

STING

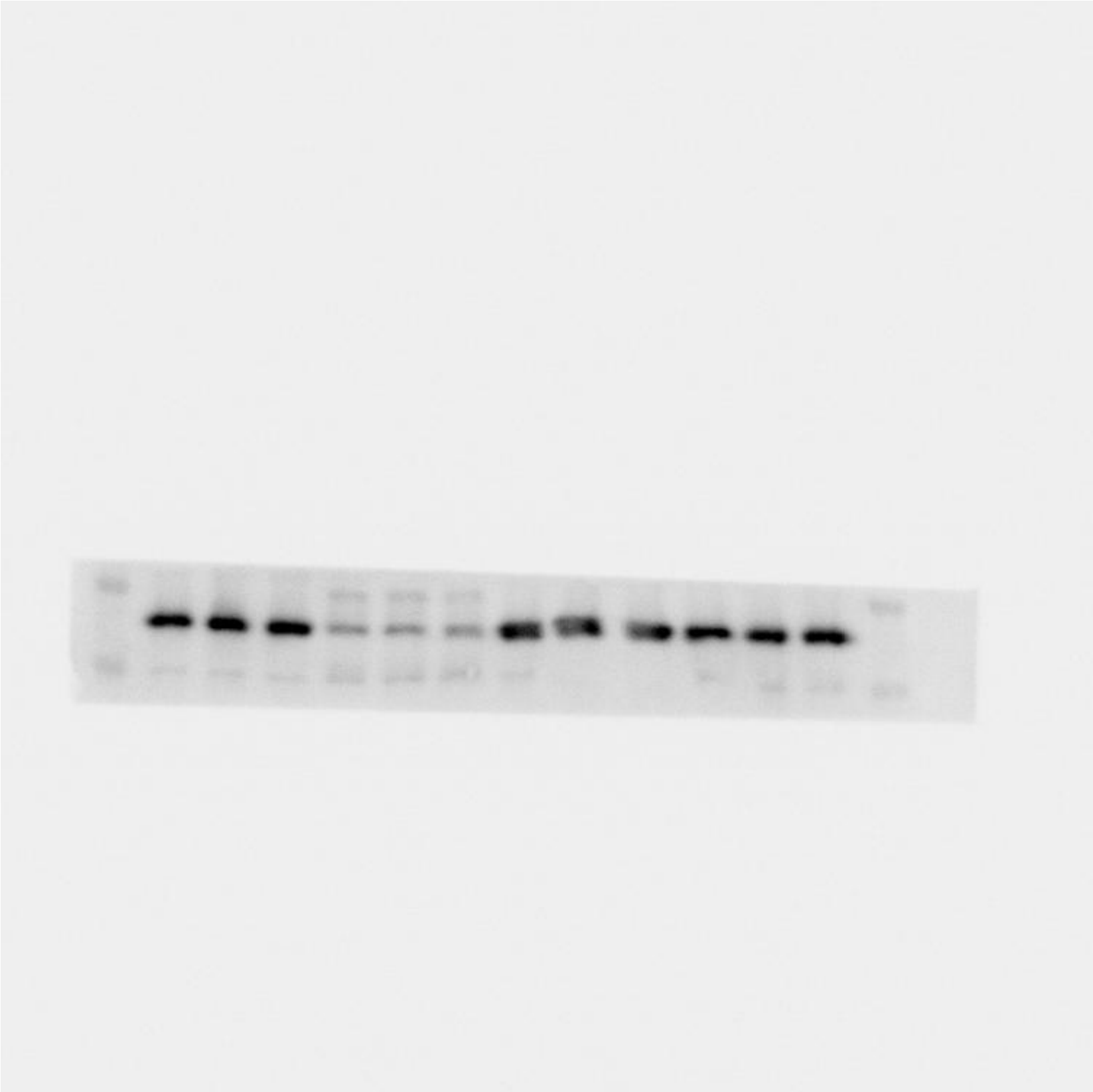

GAPDH

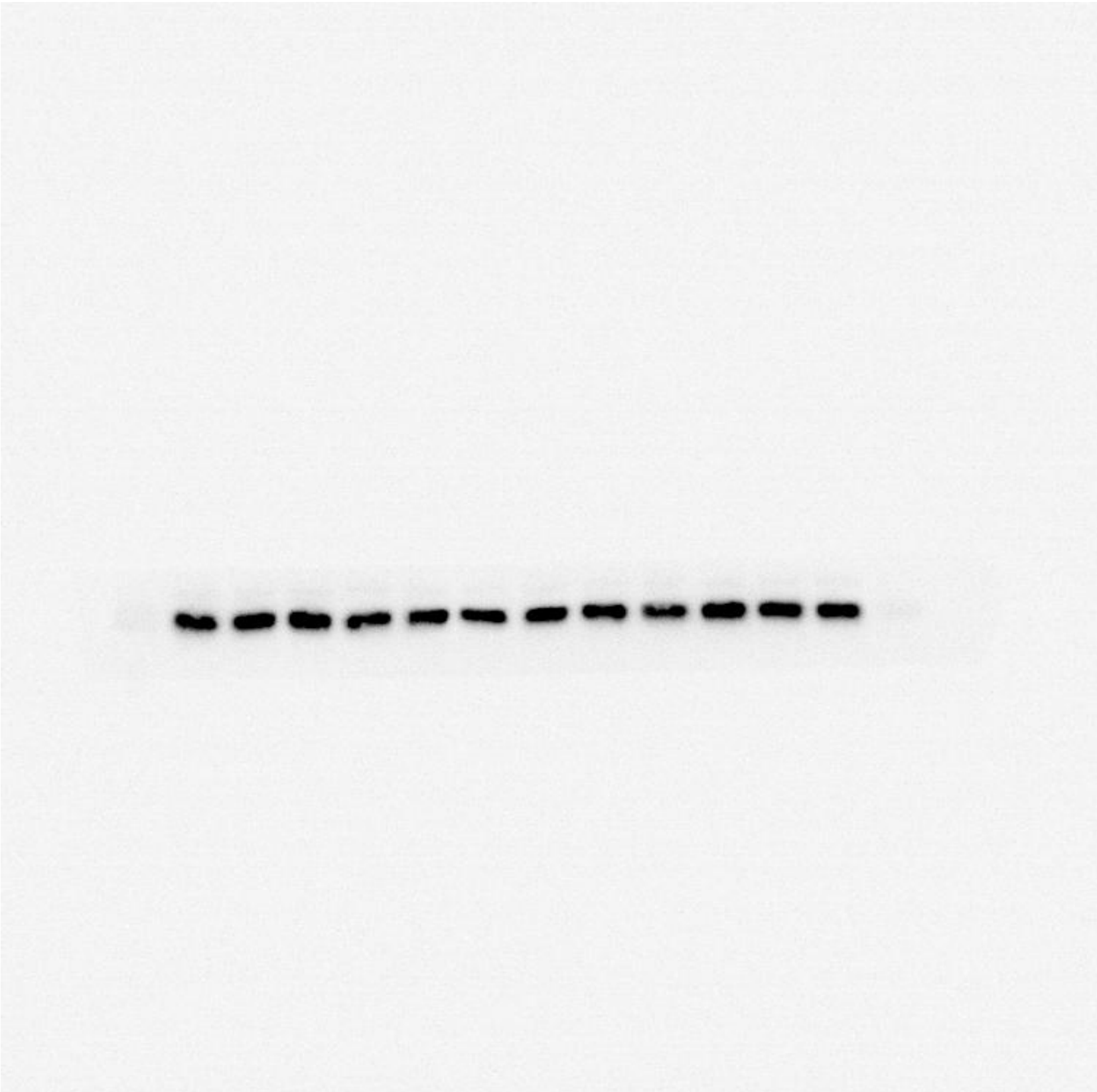

FigS9A

PRMT2

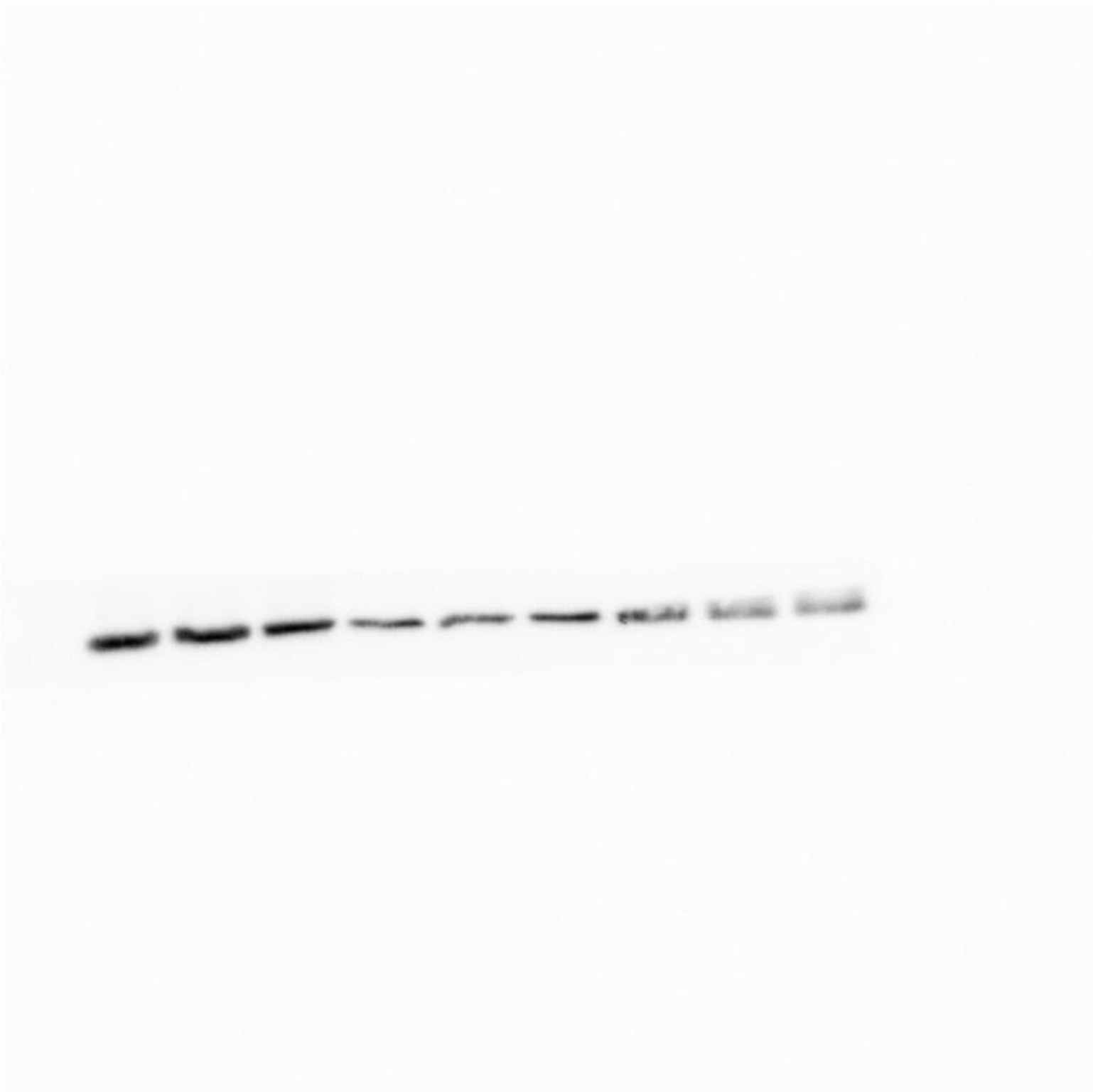

GAPDH

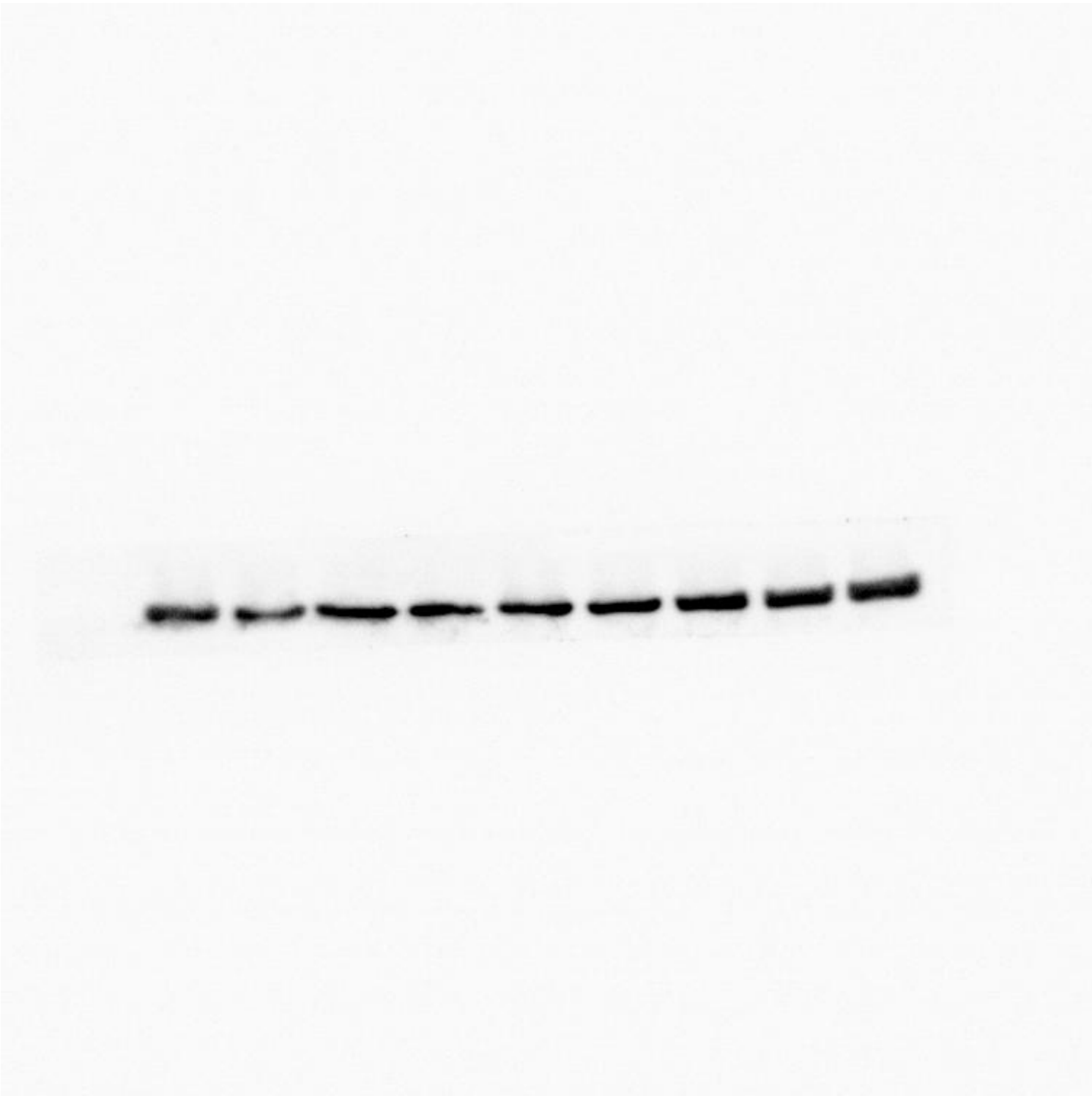

p-TBK1

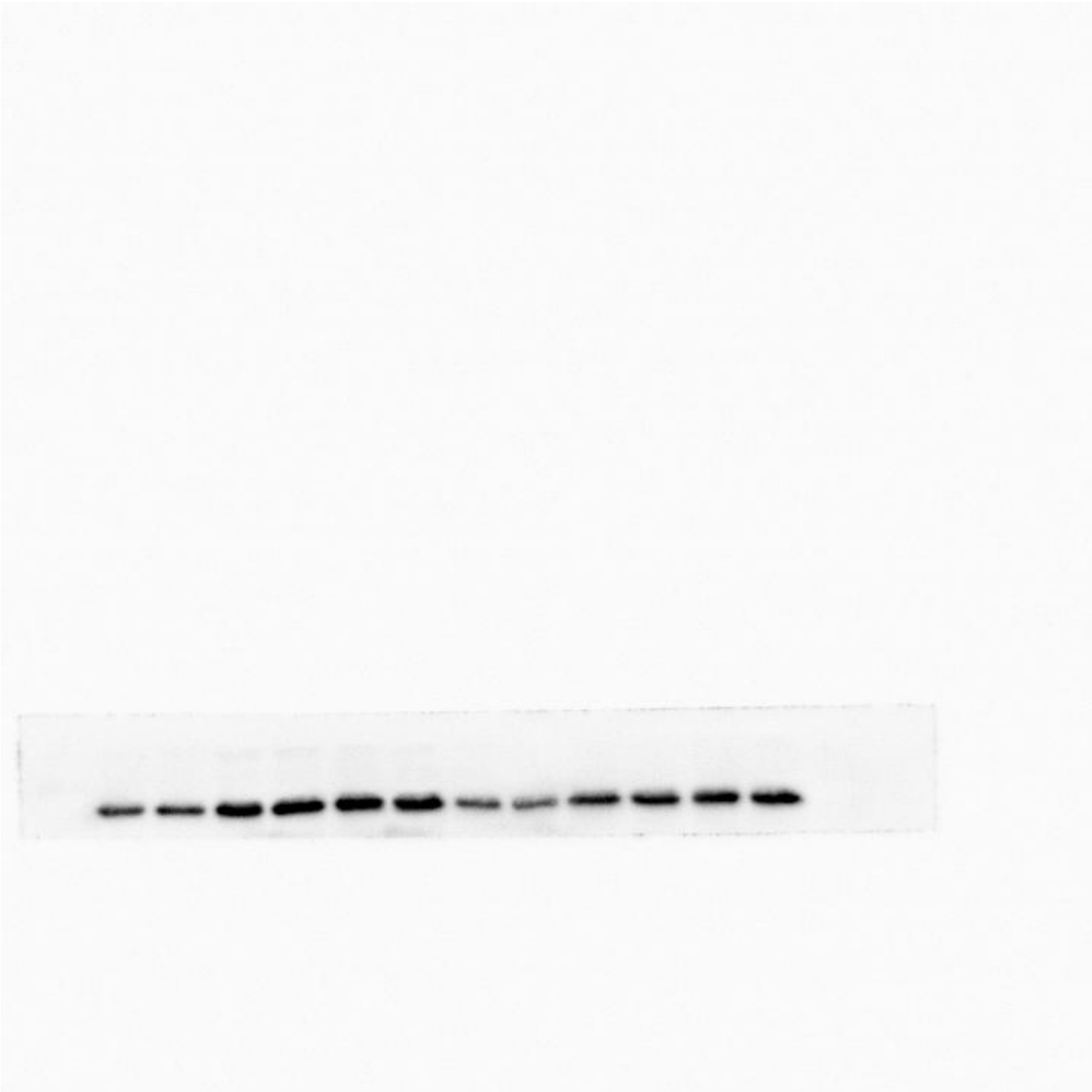

TBK1

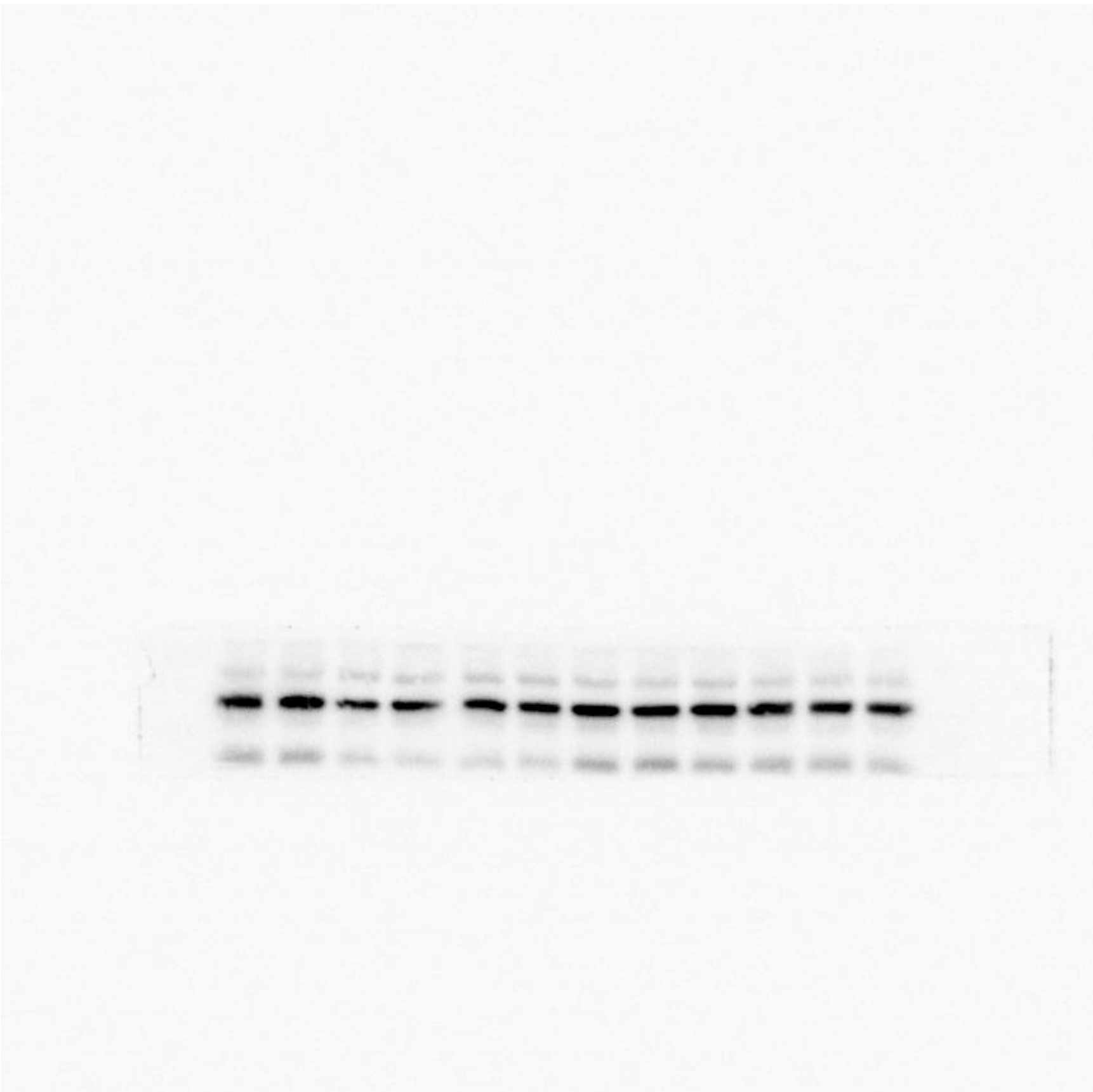

p-IRF3

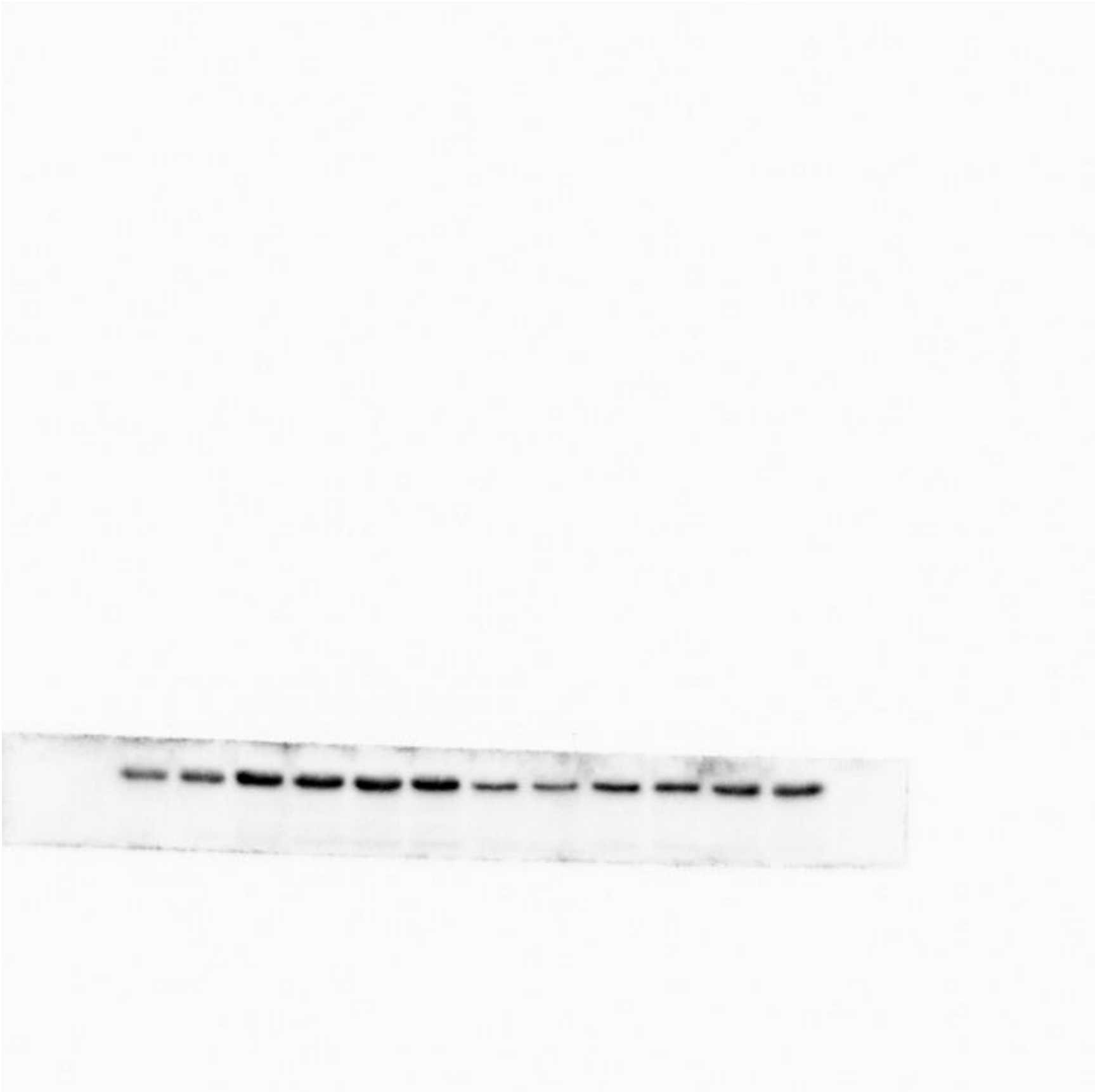

IRF3

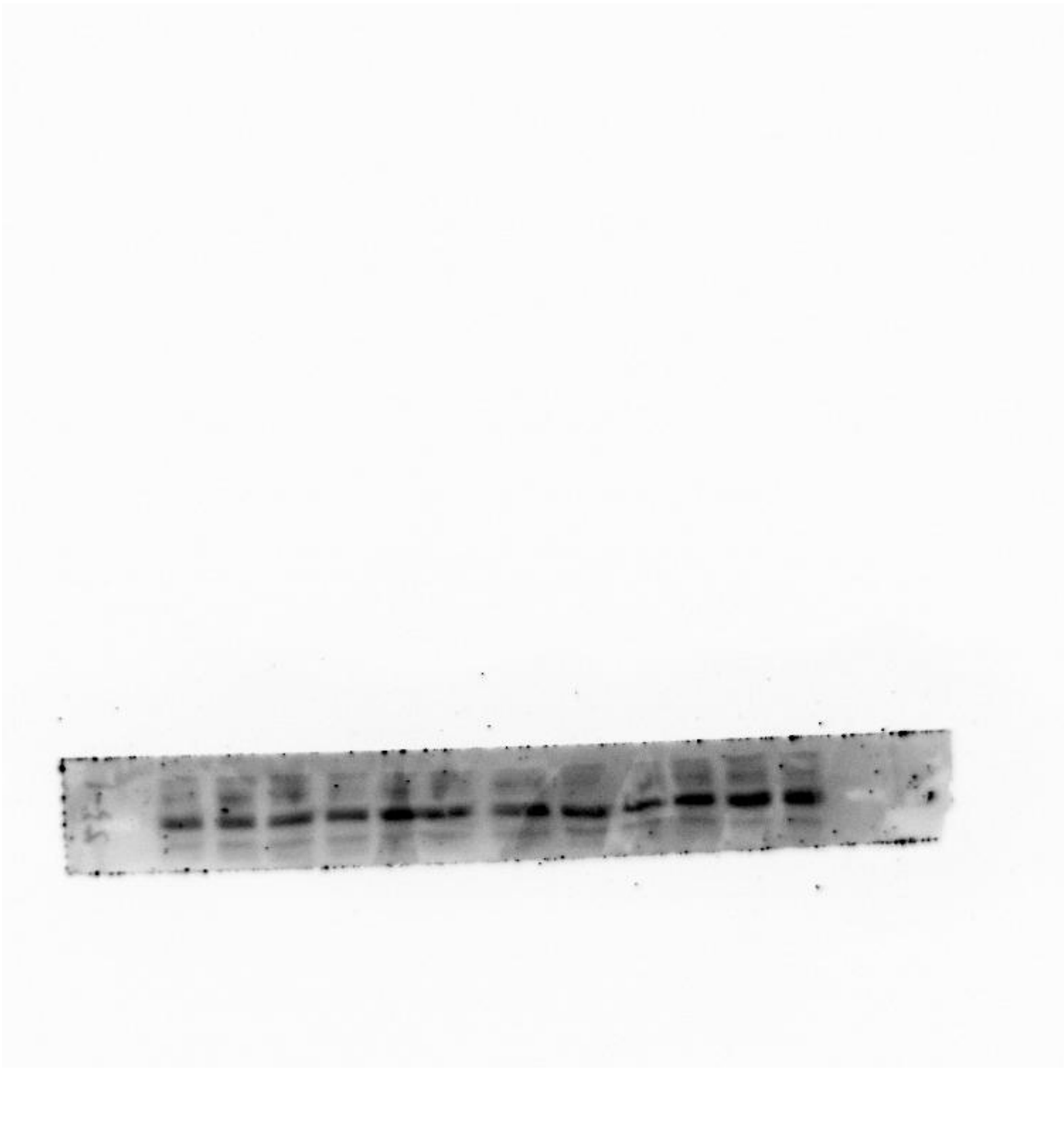

STING

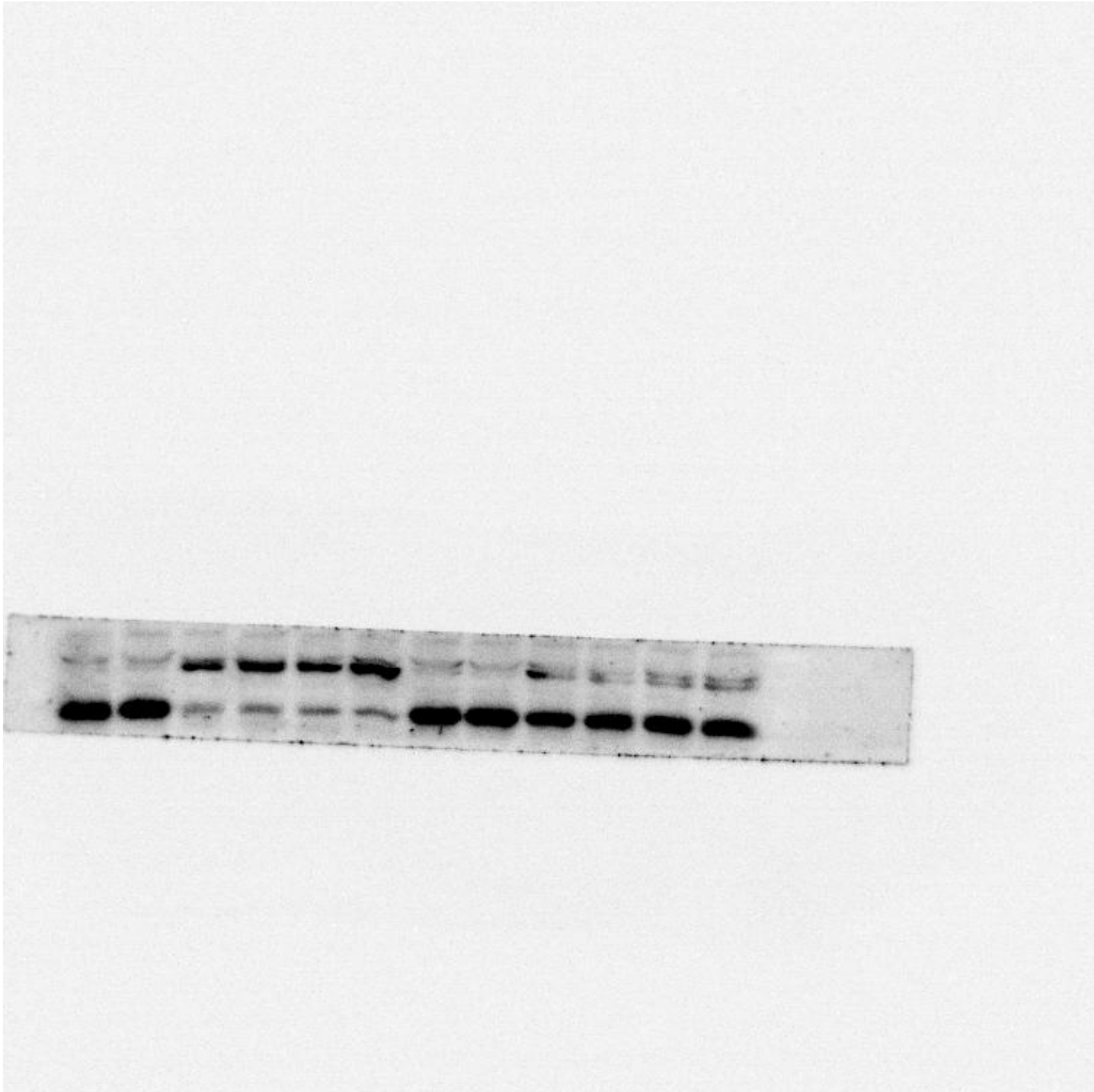

GAPDH

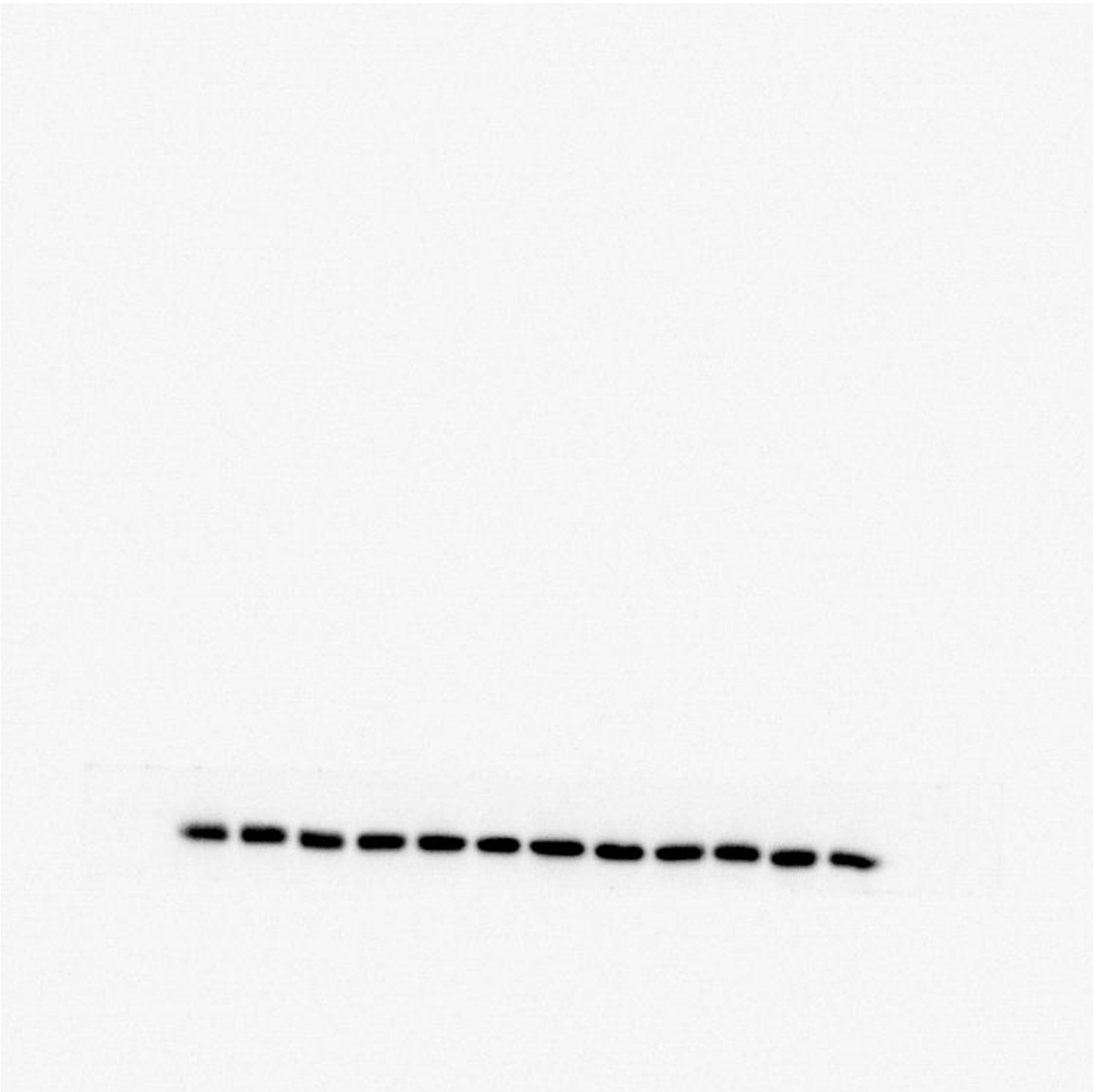

Supplement: Supplementary file 2 [file jci-136-197274-s186.pdf]
